# The burden of bacterial antimicrobial resistance in the WHO European region in 2019: a cross-country systematic analysis

**DOI:** 10.1016/S2468-2667(22)00225-0

**Published:** 2022-10-14

**Authors:** European Antimicrobial Resistance Collaborators, Tomislav Mestrovic, Gisela Robles Aguilar, Lucien R Swetschinski, Kevin S Ikuta, Authia P Gray, Nicole Davis Weaver, Chieh Han, Eve E Wool, Anna Gershberg Hayoon, Simon I Hay, Christiane Dolecek, Benn Sartorius, Christopher J L Murray, Isaac Yeboah Addo, Bright Opoku Ahinkorah, Ayman Ahmed, Mamoon A Aldeyab, Kasim Allel, Robert Ancuceanu, Anayochukwu Edward Anyasodor, Marcel Ausloos, Fabio Barra, Akshaya Srikanth Bhagavathula, Dinesh Bhandari, Sonu Bhaskar, Natália Cruz-Martins, Anna Dastiridou, Klara Dokova, Eleonora Dubljanin, Oyewole Christopher Durojaiye, Adeniyi Francis Fagbamigbe, Simone Ferrero, Peter Andras Gaal, Veer Bala Gupta, Vijai Kumar Gupta, Vivek Kumar Gupta, Claudiu Herteliu, Salman Hussain, Irena M Ilic, Milena D Ilic, Elham Jamshidi, Tamas Joo, André Karch, Adnan Kisa, Sezer Kisa, Tomislav Kostyanev, Hmwe Hmwe Kyu, Judit Lám, Graciliana Lopes, Alexander G Mathioudakis, Alexios-Fotios A Mentis, Irmina Maria Michalek, Mohammad Ali Moni, Catrin E Moore, Francesk Mulita, Ionut Negoi, Ruxandra Irina Negoi, Tamás Palicz, Adrian Pana, João Perdigão, Ionela-Roxana Petcu, Navid Rabiee, David Laith Rawaf, Salman Rawaf, Murad Ziyaudinovich Shakhmardanov, Aziz Sheikh, Luís Manuel Lopes Rodrigues Silva, Valentin Yurievich Skryabin, Anna Aleksandrovna Skryabina, Bogdan Socea, Andy Stergachis, Temenuga Zhekova Stoeva, Chandra Datta Sumi, Arulmani Thiyagarajan, Marcos Roberto Tovani-Palone, Metin Yesiltepe, Sojib Bin Zaman, Mohsen Naghavi

## Abstract

**Background:**

Antimicrobial resistance (AMR) represents one of the most crucial threats to public health and modern health care. Previous studies have identified challenges with estimating the magnitude of the problem and its downstream effect on human health and mortality. To our knowledge, this study presents the most comprehensive set of regional and country-level estimates of AMR burden in the WHO European region to date.

**Methods:**

We estimated deaths and disability-adjusted life-years attributable to and associated with AMR for 23 bacterial pathogens and 88 pathogen–drug combinations for the WHO European region and its countries in 2019. Our methodological approach consisted of five broad components: the number of deaths in which infection had a role, the proportion of infectious deaths attributable to a given infectious syndrome, the proportion of infectious syndrome deaths attributable to a given pathogen, the percentage of a given pathogen resistant to an antimicrobial drug of interest, and the excess risk of mortality (or duration of an infection) associated with this resistance. These components were then used to estimate the disease burden by using two counterfactual scenarios: deaths attributable to AMR (considering an alternative scenario where infections with resistant pathogens are replaced with susceptible ones) and deaths associated with AMR (considering an alternative scenario where drug-resistant infections would not occur at all). Data were solicited from a wide array of international stakeholders; these included research hospitals, surveillance networks, and infection databases maintained by private laboratories and medical technology companies. We generated 95% uncertainty intervals (UIs) for final estimates as the 25th and 975th ordered values across 1000 posterior draws, and models were cross-validated for out-of-sample predictive validity.

**Findings:**

We estimated 541 000 deaths (95% UI 370 000–763 000) associated with bacterial AMR and 133 000 deaths (90 100–188 000) attributable to bacterial AMR in the whole WHO European region in 2019. The largest fatal burden of AMR in the region came from bloodstream infections, with 195 000 deaths (104 000–333 000) associated with resistance, followed by intra-abdominal infections (127 000 deaths [81 900–185 000]) and respiratory infections (120 000 deaths [94 500–154 000]). Seven leading pathogens were responsible for about 457 000 deaths associated with resistance in 53 countries of this region; these pathogens were, in descending order of mortality, *Escherichia coli, Staphylococcus aureus, Klebsiella pneumoniae, Pseudomonas aeruginosa, Enterococcus faecium, Streptococcus pneumoniae*, and *Acinetobacter baumannii*. Methicillin-resistant *S aureus* was shown to be the leading pathogen–drug combination in 27 countries for deaths attributable to AMR, while aminopenicillin-resistant *E coli* predominated in 47 countries for deaths associated with AMR.

**Interpretation:**

The high levels of resistance for several important bacterial pathogens and pathogen–drug combinations, together with the high mortality rates associated with these pathogens, show that AMR is a serious threat to public health in the WHO European region. Our regional and cross-country analyses open the door for strategies that can be tailored to leading pathogen–drug combinations and the available resources in a specific location. These results underscore that the most effective way to tackle AMR in this region will require targeted efforts and investments in conjunction with continuous outcome-based research endeavours.

**Funding:**

Bill & Melinda Gates Foundation, Wellcome Trust, and Department of Health and Social Care using UK aid funding managed by the Fleming Fund.

## Introduction

Antimicrobial resistance (AMR) represents a salient challenge of our time, as the loss of effective antimicrobials might result in common infections becoming life threatening and hinder the ability to perform common surgical procedures and other medical treatments. The Sustainable Development Goals (SDGs) consider AMR to be both a global public health problem and a societal issue.^1^ The continuing emergence of AMR might impede progress on many of the SDGs.^1^ The *Review on Antimicrobial Resistance* estimated that AMR could result in the global loss of 10 million lives per year by 2050, with substantial economic ramifications.^2^ Although such forecasts have been criticised by some authors,^3^, ^4^ the first comprehensive global assessment of AMR burden in 2019^5^ estimated 4·95 million deaths associated with bacterial AMR and 1·27 million deaths attributable to bacterial AMR in that year alone, corroborating a dire trend.

**Research in context**

**Evidence before this study**

There is widespread consensus that antimicrobial resistance (AMR) represents an emerging and alarming threat to human health worldwide. The highly cited *Review on Antimicrobial Resistance* from 2016 concluded that as many as 10 million people could die annually from AMR by 2050. This concerning trend was recently confirmed by the 2019 global burden of AMR study, which showed that AMR is a leading cause of death around the world, particularly in low-resource settings. Through an in-depth search of the articles available in PubMed covering exposure to resistant bacterial agents (details presented in appendix 1 pp 7–10), we have retrieved many human-focused publications on AMR concentrating on Europe. The most noteworthy is a 2019 paper estimating the burden of infections caused by 16 pathogen–drug combinations from the European Antimicrobial Resistance Surveillance Network in countries of the EU and European Economic Area for the year 2015, measured in number of cases, deaths attributable to AMR, and disability-adjusted life-years. Since this 2019 paper did not cover all countries in Europe and provided estimates for only a limited number of pathogen–drug combinations, there is a need for more comparable, country-level information and a comprehensive and more timely pan-European overview.

**Added value of this study**

To our knowledge, this study provides the most comprehensive analysis of the burden of AMR in the WHO European region to date, providing estimates for 53 countries, 23 bacterial pathogens, and 88 pathogen–drug combinations in 2019. These estimates advance previous related work in the WHO European region in several ways. First, this study offers an expanded scope, including additional countries in Europe, and an increased number of pathogen–drug combinations. Second, we use the major methodological innovations put forth in the 2019 global burden of bacterial AMR study. Third, we draw on two different counterfactual scenarios, acknowledged previously in other studies, to describe the magnitude of the problem within the WHO European region. Fourth, this study allows for comparability with other causes of death since it builds on estimates of disease incidence, prevalence, and mortality from the Global Burden of Diseases, Injuries, and Risk Factors Study 2019.

**Implications of all the available evidence**

Our estimates indicate that bacterial AMR is a substantial problem in the WHO European region, with stark differences between subregions and specific countries. Such estimates of the impact of AMR on morbidity and mortality are indispensable for informing public health investment decisions for each country in this region. Additionally, highlighting specific pathogens and pathogen–drug combinations with the highest estimated burden might specifically inform policy targets and policy design—concentrating on expanding infection prevention and control programmes, patient awareness, and informing research priorities in the field of antibiotic and vaccine development.

Coordinated global-level, regional-level, and country-level strategies are necessary to attenuate the emergence and spread of AMR; however, considering different trends in various parts of the world,^5^ tailored and data-driven regional approaches will be needed for local policy decisions regarding laboratory capacity and data collection systems, infection prevention and control programmes, antimicrobial stewardship initiatives, and antibiotic and vaccine access and development. In 2017, the European Commission initiated a European One Health Action Plan against AMR;^6^ however, more concerted efforts are needed, as a 2022 report jointly published by the European Centre for Disease Prevention and Control (ECDC) and the WHO Regional Office for Europe showed that AMR is widespread in the WHO European region.^7^

Most AMR studies have focused on the EU and European Economic Area (EEA); hence, more comparable information and a comprehensive, pan-European overview is needed, especially if we aim to compare data before and during the COVID-19 pandemic in the future. Previous ECDC surveillance reports have also concentrated on invasive isolates (ie, from blood and cerebrospinal fluid) of eight bacterial species deemed of public health importance in Europe^8^, ^9^ and do not provide a full epidemiological picture. Although this is a multi-layered problem with many known downstream effects on mortality, length of hospital stay, and respective health-care costs,^2^, ^5^, ^10^, ^11^ many existing studies and reports have generally focused on only one measure of AMR burden.^12^

Cassini and colleagues^11^ went one step further by estimating the burden of AMR in EU and EEA countries in 2015 for eight bacterial pathogens and 16 pathogen–drug combinations by measuring the number of cases of all types of infections with resistant bacteria, the number of attributable deaths, and the number of disability-adjusted life-years (DALYs). After obtaining a global snapshot of AMR in 2019,^5^ we saw the need to summarise the burden for the countries of the WHO European region using our larger number of data sources and infectious syndromes, as well as implementing two counterfactual scenarios (previously acknowledged in the literature^5^, ^13^, ^14^) where drug-resistant infections are replaced by either susceptible infections or no infection in a scenario where drug resistance is eliminated. Consequently, this study presents the first regional-level and country-level estimates for the WHO European region of the burden of bacterial AMR in 2019, covering an extensive set of pathogens and pathogen–drug combinations with the use of consistent methods for both counterfactual scenarios. This manuscript was produced as part of the Global Burden of Diseases, Injuries, and Risk Factors Study (GBD) Collaborator Network and in accordance with the GBD Protocol.^15^

## Methods

### Overview and input data

This study extends the results of the 2019 global burden of AMR study^5^ and uses its methodological approach but provides more granular and country-specific estimates within the WHO European region. More specifically, we present here aggregated estimates for the entire WHO European region in 2019, as well as country-level estimates, based on the global estimation of all-age and age-specific deaths and DALYs (with DALYs calculated as the sum of years of life lost due to premature mortality and years of healthy life lost due to disability) for 204 countries and territories. In some analyses, countries belonging to the WHO European region were grouped in accordance with GBD regions (ie, western Europe, central Europe, eastern Europe, central Asia, and north Africa and the Middle East). Disease burdens associated with and attributable to AMR were estimated for 12 major infectious syndromes and one residual category (previously described in the 2019 global burden of AMR study^5^), 23 bacterial pathogens, and 88 pathogen–drug combinations (appendix 1 pp 21–22).

Our input data consisted of 471 million individual records or isolates covering 7585 study-location-years obtained from surveillance systems, hospital systems, systematic literature reviews, and other sources (appendix 1 pp 10–27). From this global input data, our models were heavily informed by the wealth of data from Europe (appendix 1 pp 6–7). All data inputs for our models were empirical data (ie, not modelled estimates), except for a custom meta-analysis of vaccine probe data, which we used to estimate the fraction of pneumonia caused by *Streptococcus pneumoniae*. We used clinical breakpoints and methods from the Clinical and Laboratory Standards Institute (CLSI) as guidance for classification of isolates into categories of susceptible or resistant.^16^

Our overall approach can be divided into five broad components: the number of deaths in which infection was implicated, the proportion of infectious deaths attributable to a given infectious syndrome, the proportion of infectious syndrome deaths attributable to a given pathogen, the percentage of a given pathogen resistant to an antimicrobial drug of interest, and the excess risk of death or duration of an infection associated with this resistance.

We followed GATHER^17^ and PRISMA^18^ guidelines (appendix 1 pp 61–84).

### Estimation steps

In our approach, ten estimation steps took place within the aforementioned five broad modelling components (appendix 1 pp 10–30). In estimation steps one and two, we defined the number of deaths in which infection was implicated by using GBD 2019 cause of death estimates^19^ to determine the number of deaths by age, sex, and location for which either the underlying cause of death was of infectious origin or for which the pathway to death included sepsis. By estimating AMR, we considered infectious syndromes that played a role in the pathway of sepsis deaths, some of which might not have been the underlying cause of death. In estimation steps three and four, we used multiple data sources to estimate pathogen distribution for each infectious syndrome for deaths and incident cases for each age, sex, and location. In estimation steps five, six, and seven, we used data from millions of isolates to estimate the prevalence of phenotypic resistance by country for each of 88 pathogen–drug combinations (appendix 1 pp 20–25). In estimation steps eight and nine, we estimated the relative risk of death for a resistant infection compared with that of a drug-sensitive infection for each pathogen–drug combination using data from 164 sources encompassing 511 870 patients with known outcome and resistance information; from this global input, the primary emphasis was on European sources (appendix 1 pp 6–7). Availability of input data for these nine estimation steps is documented in appendix 1 (p 42).

To generate burden estimates of multiple pathogen–drug combinations that were mutually exclusive within a pathogen (and could thus be added), we introduced a population-attributable fraction (PAF) for each resistance profile with resistance to at least one drug. This metric considers prevalence of resistance, excess risk, and a redistribution of burden to each antibiotic on the basis of the respective excess risk.

In estimation step ten, we computed two counterfactual scenarios to quantify the benefit of eliminating drug-resistant infections, estimating the drug-resistant burden as deaths and DALYs directly attributable to bacterial AMR on the basis of the counterfactual of drug-sensitive infection, and deaths and DALYs associated with bacterial AMR on the basis of the counterfactual of no infection (appendix 1 pp 27–30).

### AMR burden calculation approach

In a scenario in which drug-resistant infections are replaced with drug-susceptible ones, we considered the excess risk of resistance, known as the attributable to AMR counterfactual scenario. Deaths attributable to AMR were calculated by multiplying the number of deaths for each underlying cause by the fraction of these deaths in which infection was implicated, followed by multiplying the fraction of infectious deaths attributable to each infectious syndrome. This was then multiplied by the fraction of infectious syndrome deaths attributable to each pathogen and by the PAF for each location-year and pathogen–drug combination.

Under the no-infection counterfactual scenario, infections that are resistant would not occur; this is also termed the associated with AMR scenario. Calculations here closely follow the process described for the attributable to AMR counterfactual, except the PAF is replaced with the prevalence of resistance for each location-year and pathogen–drug combination. We used a similar approach to calculate DALYs for both counterfactual scenarios (appendix 1 pp 27–30).

### Modelling tools and framework

Details on our modelling approach can be found in the 2019 global burden of AMR study^5^ and in appendix 1 of this paper (pp 10–30). Briefly, for estimation steps three and four, we used the Bayesian meta-regression tool MR-BRT to estimate case-fatality rates (CFRs) as a function of the Healthcare Access and Quality Index and various bias covariates. We used multinomial estimation with partial and composite observations to incorporate heterogeneous data in the estimation of pathogen distributions for each infectious syndrome. In estimation steps five, six, and seven, we used a two-stage spatiotemporal modelling framework to estimate the prevalence of resistance in each pathogen–drug combination.

Given the relationship between antibiotic consumption levels and AMR rates,^20^ we modelled antibiotic consumption at the national level to use as a covariate in the stage one models of prevalence of resistance, using an ensemble spatiotemporal Gaussian process regression model to combine antibiotic usage estimates with pharmaceutical sales data for low-income and middle-income countries. In cross-country comparisons, the indicator metric “defined daily doses per 1000 inhabitants per day” was used to report antibiotic consumption in the community and within the hospital setting (in accordance with the WHO Anatomical Therapeutic Chemical classification) by providing a rough estimate of the proportion of the population treated with antimicrobials on a daily basis. We additionally pursued a correlation analysis of mortality rates attributable to AMR with antibiotic consumption rates in defined daily doses, as well as with the Socio-demographic Index (SDI). We used MR-BRT and a two-stage nested mixed effects meta-regression model in the estimation of both relative risk of death and excess risk of hospital stay for each pathogen–drug combination (appendix 1 pp 23–24). The software used for these analyses is described in appendix 1 (pp 23–24).

### Uncertainty analysis

Consistent with GBD methods,^19^ we propagated uncertainty from each step of the analysis into the final estimates of deaths and infections attributable to and associated with drug resistance by taking the 25th and 975th of 1000 draws from the posterior distribution of each quantity of interest. The models were cross-validated for out-of-sample predictive validity.

### Role of the funding source

The funders of the study had no role in study design, data collection, data analysis, data interpretation, or the writing of the report.

## Results

### AMR burden by infectious syndromes in the WHO European region

We estimated 1·2 million (95% uncertainty interval [UI] 0·9–1·7) deaths in 2019 involving one of 11 infectious syndromes (ie, those with lethal outcomes) as an underlying or an intermediate cause of death in the WHO European region. Of these, we focused on 0·9 million (0·6–1·3) deaths that were caused by both susceptible and resistant bacterial agents to estimate AMR burden. The largest fatal burden in the region came from bloodstream infections (319 000 deaths [169 000–548 000] or 34%), followed by respiratory infections (231 000 deaths [186 000–293 000] or 25%) and peritoneal and intra-abdominal infections (197 000 deaths [126 000–287 000] or 21%; appendix 1 p 48). Together, these three infectious syndromes accounted for 80% of the fatal bacterial infection burden in the region. The highest mortality rates per 100 000 population were observed in eastern Europe (19·9 [13·1–28·5] attributable to AMR and 74·0 [48·8–105·6] associated with AMR) and central Europe (16·6 [10·5–25·0] attributable and 68·0 [43·2–100·9] associated). In comparison, western Europe had 11·7 deaths per 100 000 (8·0–16·6) attributable to AMR and 52·5 deaths per 100 000 (37·0–73·0) associated with AMR (appendix 1 p 39).

In the WHO European region, we estimated the total burden of AMR mortality in 2019 to be 541 000 deaths (95% UI 370 000–763 000) associated with AMR and 133 000 deaths (90 100–188 000) attributable to AMR (table 1). Among 319 000 deaths involving bloodstream infections, 195 000 (104 000–333 000) were associated with and 47 200 (24 700–79 300) were attributable to any resistant pathogen–drug combination. Likewise, among 197 000 deaths involving peritoneal and intra-abdominal infections, 127 000 (81 900–185 000) were associated with and 31 200 (19 900–45 600) were attributable to any resistant pathogen–drug combination. Together with respiratory infections (which accounted for 120 000 deaths [94 500–154 000] associated with and 28 500 deaths [21 200–38 500] attributable to AMR), these syndromes accounted for 80·5% of deaths attributable to and 81·7% of deaths associated with AMR in the WHO European region in 2019 (table 1).

**Table 1:** Overall AMR burden by infectious syndrome in the WHO European region in 2019

	**Associated with AMR**				**Attributable to AMR**			
	Deaths		DALYs		Deaths		DALYs	
	Counts	Rate, per 100 000 population	Counts	Rate, per 100 000 population	Counts	Rate, per 100 000 population	Counts	Rate, per 100 000 population
BSI	195 000 (104 000–333 000)	20·9 (11·1–35·8)	4 180 000 (2 270 000–6 870 000)	448·3 (243·5–737·3)	47 200 (24 700–79 300)	5·1 (2·7–8·5)	1 020 000 (566 000–1 670 000)	109·4 (60·7–178·9)
Bacterial skin infections	14 300 (5700–30 000)	1·5 (0·6–3·2)	258 000 (106 000–557 000)	27·7 (11·3–59·8)	3080 (1180–6700)	0·3 (0·1–0·7)	56 000 (22 100–124 000)	6·0 (2·4–13·3)
Bone and joint infections	1530 (477–3500)	0·2 (0·1–0·4)	31 100 (9210–72 100)	3·3 (1·0–7·7)	342 (104–817)	0 (0–0·1)	7020 (2050–16 700)	0·8 (0·2–1·8)
CNS infections*	2130 (1380–3640)	0·2 (0·1–0·4)	90 500 (59 400–148 000)	9·7 (6·4–15·8)	504 (322–902)	0·1 (0–0·1)	21 100 (13 600–34 900)	2·3 (1·5–3·7)
Cardiac infections	20 000 (13 200–29 400)	2·1 (1·4–3·2)	354 000 (242 000–525 000)	38·0 (25·9–56·3)	4670 (3030–7 000)	0·5 (0·3–0·8)	83 600 (55 000–127 000)	9·0 (5·9–13·6)
Diarrhoea	649 (361–1090)	0·1 (0–0·1)	42 200 (24 400–69 200)	4·5 (2·6–7·4)	145 (78–252)	0 (0–0)	7780 (4290–13 000)	0·8 (0·5–1·4)
Gonorrhoea and chlamydia	..	..	2420 (1380–3870)	0·3 (0·1–0·4)	..	..	243 (70–492)	0 (0–0·1)
Intra-abdominal infections	127 000 (81 900–185 000)	13·7 (8·8–19·8)	2 860 000 (1 800 000–4 200 000)	307·2 (193·3–451·0)	31 200 (19 900–45 600)	3·3 (2·1–4·9)	708 000 (438 000–1 050 000)	76·0 (47·0–113·1)
LRI and thorax infections	120 000 (94 500–154 000)	12·9 (10·1–16·6)	2 760 000 (2 240 000–3 460 000)	296·0 (240·8–370·9)	28 500 (21 200–38 500)	3·1 (2·3–4·1)	656 000 (502 000–855 000)	70·4 (53·8–91·8)
Tuberculosis	11 800 (9150–15 000)	1·3 (1·0–1·6)	501 000 (390 000–626 000)	53·7 (41·8–67·2)	5670 (2190–9510)	0·6 (0·2–1·0)	219 000 (86 500–365 000)	23·5 (9·3–39·2)
Typhoid, paratyphoid, and iNTS	67 (37–121)	0 (0–0)	3130 (1340–6470)	0·3 (0·1–0·7)	13 (4–31)	0 (0–0)	629 (144–1670)	0·1 (0–0·2)
UTI	48 700 (35 600–68 000)	5·2 (3·8–7·3)	833 000 (600 000–1 190 000)	89·4 (64·4–127·2)	11 500 (8310–16 800)	1·2 (0·9–1·8)	201 000 (143 000–297 000)	21·6 (15·3–31·9)
All infectious syndromes	541 000 (370 000–763 000)	58·1 (39·7–81·9)	11 900 000 (8 190 000–16 700 000)	1278·5 (879·0–1794·0)	133 000 (90 100–188 000)	14·3 (9·7–20·2)	2 980 000 (2 020 000–4 210 000)	319·8 (216·5–451·8)

Data are estimates (95% uncertainty interval). Estimates were aggregated across drugs, accounting for the co-occurrence of resistance to multiple drugs. For gonorrhoea and chlamydia, we did not estimate the fatal burden, thus only the DALY burden is presented. AMR=antimicrobial resistance. BSI=bloodstream infections. DALYs=disability-adjusted life-years. LRI=lower respiratory infections. iNTS=invasive non-typhoidal salmonellae. UTI=urinary tract infections.

* Includes meningitis.

### AMR burden by species and pathogen–drug combinations in the WHO European region

**Figure 1:**
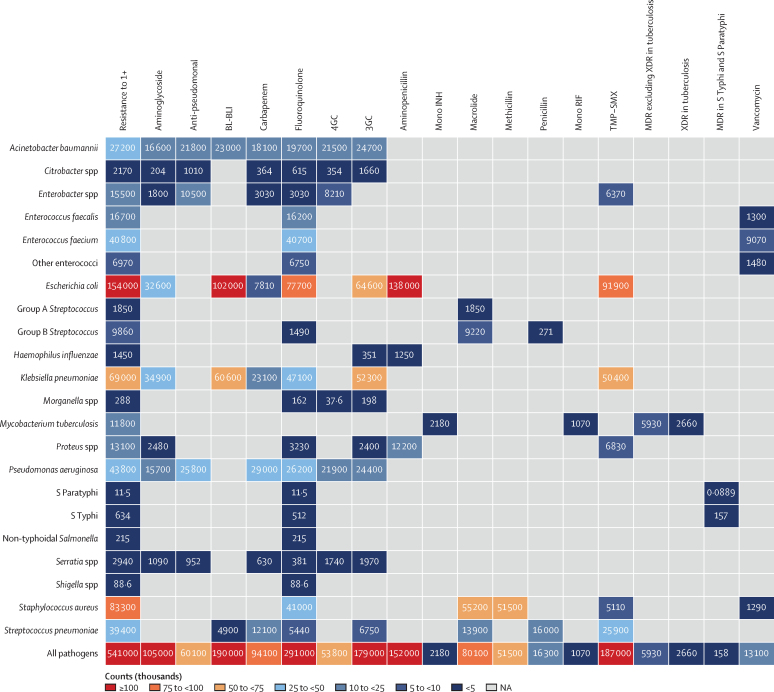
Heatmap representing death counts associated with antimicrobial resistance by pathogen–drug combination in the WHO European region in 2019 Group A *Streptococcus* pertains to *Streptococcus pyogenes*, and Group B *Streptococcus* pertains to *Streptococcus agalactiae*. Some counts are rounded to the nearest hundred. 3GC=third-generation cephalosporins. 4GC=fourth-generation cephalosporins. Anti-pseudomonal=anti-pseudomonal penicillin or β-lactamase inhibitors. BL-BLI=β-lactam or β-lactamase inhibitors. MDR=multidrug resistance. Mono INH=isoniazid mono-resistance. Mono RIF=rifampicin mono-resistance. NA=not applicable. Resistance to 1+=resistance to one or more drugs. S Paratyphi=*Salmonella enterica* serotype Paratyphi. S Typhi=*S enterica* serotype Typhi. TMP-SMX=trimethoprim-sulfamethoxazole. XDR=extensive drug resistance.

**Figure 2:**
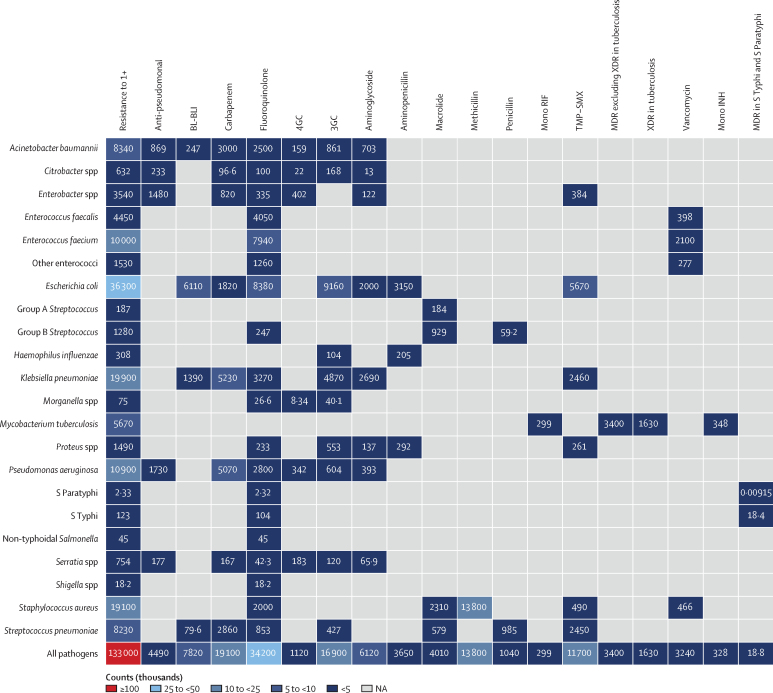
Heatmap representing death counts attributable to antimicrobial resistance by pathogen–drug combination in the WHO European region in 2019 Group A *Streptococcus* pertains to *Streptococcus pyogenes*, and Group B *Streptococcus* pertains to *Streptococcus agalactiae*. Some counts are rounded to the nearest hundred. 3GC=third-generation cephalosporins. 4GC=fourth-generation cephalosporins. Anti-pseudomonal=anti-pseudomonal penicillin or β-lactamase inhibitors. BL-BLI=β-lactam or β-lactamase inhibitors. MDR=multidrug resistance. Mono INH=isoniazid mono-resistance. Mono RIF=rifampicin mono-resistance. NA=not applicable. Resistance to 1+=resistance to one or more drugs. S Paratyphi=*Salmonella enterica* serotype Paratyphi. S Typhi=*S enterica* serotype Typhi. TMP-SMX=trimethoprim-sulfamethoxazole. XDR=extensive drug resistance.

In 2019, seven pathogens were each responsible for more than 25 000 deaths associated with AMR in the WHO European region: *Escherichia coli* (153 982 deaths), *Staphylococcus aureus* (83 325 deaths), *Klebsiella pneumoniae* (68 994 deaths), *Pseudomonas aeruginosa* (43 801 deaths), *Enterococcus faecium* (40 800 deaths), *S pneumoniae* (39 383 deaths), and *Acinetobacter baumannii* (27 206 deaths; figure 1). Deaths attributable to AMR are presented in figure 2. Together, these seven pathogens were responsible for 112 784 deaths attributable to and 457 491 deaths associated with AMR in the region. Table 2 shows the overall AMR burden by specific pathogens.

**Table 2:** Overall AMR burden by pathogen in the WHO European region in 2019

	**Associated with AMR**				**Attributable to AMR**			
	Deaths		DALYs		Deaths		DALYs	
	Counts	Rate, per 100 000 population	Counts	Rate, per 100 000 population	Counts	Rate, per 100 000 population	Counts	Rate, per 100 000 population
*Acinetobacter baumannii*	27 200 (15 100–45 700)	2·9 (1·6–4·9)	573 000 (319 000–955 000)	61·5 (34·3–102·5)	8340 (4430–14 100)	0·9 (0·5–1·5)	177 000 (94 400–301 000)	19·0 (10·1–32·3)
*Citrobacter* spp	2170 (1240–3630)	0·2 (0·1–0·4)	51 100 (28 600–87 000)	5·5 (3·1–9·3)	632 (322–1140)	0·1 (0–0·1)	14 900 (7430–27 500)	1·6 (0·8–3·0)
*Enterobacter* spp	15 500 (10 300–22 100)	1·7 (1·1–2·4)	354 000 (230 000–509 000)	38·0 (24·7–54·7)	3540 (2220–5220)	0·4 (0·2–0·6)	80 500 (49 200–120 000)	8·6 (5·3–12·8)
*Enterococcus faecalis*	16 700 (9950–26 300)	1·8 (1·1–2·8)	383 000 (229 000–587 000)	41·1 (24·5–63·0)	4450 (2180–7790)	0·5 (0·2–0·8)	101 000 (49 600–177 000)	10·9 (5·3–19·0)
*Enterococcus faecium*	40 800 (25 800–60 900)	4·4 (2·8–6·5)	883 000 (544 000–1 320 000)	94·8 (58·4–142·1)	10 000 (4850–17 300)	1·1 (0·5–1·9)	218 000 (105 000–376 000)	23·4 (11·2–40·4)
*Escherichia coli*	154 000 (101 000–225 000)	16·5 (10·9–24·1)	3 010 000 (1 980 000–4 370 000)	323·1 (212·1–468·9)	36 300 (22 900–55 300)	3·9 (2·5–5·9)	719 000 (452 000–1 090 000)	77·2 (48·5–117·0)
Group A *Streptococcus*	1850 (953–3420)	0·2 (0·1–0·4)	44 000 (24 000–78 200)	4·7 (2·6–8·4)	187 (20–644)	0 (0–0·1)	4150 (547–15 600)	0·4 (0·1–1·7)
Group B *Streptococcus*	9860 (6490–14 700)	1·1 (0·7–1·6)	254 000 (170 000–365 000)	27·2 (18·3–39·2)	1280 (330–3260)	0·1 (0–0·4)	31 400 (8990–83 900)	3·4 (1·0–9·0)
*Haemophilus influenzae*	1450 (1160–1800)	0·2 (0·1–0·2)	34 300 (27 500–42 600)	3·7 (3·0–4·6)	308 (119–527)	0 (0–0·1)	7580 (3380–12 700)	0·8 (0·4–1·4)
*Klebsiella pneumoniae*	69 000 (47 000–98 600)	7·4 (5·0–10·6)	1 540 000 (1 040 000–2 180 000)	165·0 (111·6–234·3)	19 900 (12 700–29 700)	2·1 (1·4–3·2)	450 000 (288 000–670 000)	48·3 (30·9–71·9)
*Morganella* spp	288 (192–432)	0 (0–0)	4650 (3020–7100)	0·5 (0·3–0·8)	75 (38–132)	0 (0–0)	1200 (623–2140)	0·1 (0·1–0·2)
*Mycobacterium tuberculosis*	11 800 (9150–15 000)	1·3 (1·0–1·6)	501 000 (390 000–626 000)	53·7 (41·8–67·2)	5670 (2190–9510)	0·6 (0·2–1·0)	219 000 (86 500–365 000)	23·5 (9·3–39·2)
*Neisseria gonorrhoeae*	..	..	2420 (1380–3870)	0·3 (0·1–0·4)	..	..	243 (70–492)	0 (0–0·1)
Non-typhoidal *Salmonella*	215 (109–369)	0 (0–0)	9600 (3020–22 500)	1·0 (0·3–2·4)	45 (6–109)	0 (0–0)	1290 (205–3190)	0·1 (0–0·3)
Other enterococci	6970 (4270–10 700)	0·7 (0·5–1·1)	145 000 (87 200–225 000)	15·6 (9·4–24·1)	1530 (549–2790)	0·2 (0·1–0·3)	31 700 (11 300–57 300)	3·4 (1·2–6·1)
*Proteus* spp	13 100 (8760–18 500)	1·4 (0·9–2·0)	239 000 (157 000–342 000)	25·7 (16·8–36·7)	1490 (782–2510)	0·2 (0·1–0·3)	27 500 (14 300–46 400)	3 (1·5–5·0)
*Pseudomonas aeruginosa*	43 800 (29 900–61 500)	4·7 (3·2–6·6)	959 000 (650 000–1 350 000)	102·9 (69·7–145·2)	10 900 (6870–16 600)	1·2 (0·7–1·8)	241 000 (151 000–366 000)	25·9 (16·2–39·3)
*Salmonella enterica* serotype Paratyphi	11 (3–18)	0 (0–0)	323 (132–536)	0 (0–0·1)	2 (0–4)	0 (0–0)	64 (11–140)	0 (0–0)
*Salmonella enterica* serotype Typhi	634 (265–1360)	0·1 (0–0·1)	25 300 (11 100–50 800)	2·7 (1·2–5·5)	123 (24–311)	0 (0–0)	4920 (990–12 100)	0·5 (0·1–1·3)
*Serratia* spp	2940 (1770–4520)	0·3 (0·2–0·5)	77 000 (46 600–119 000)	8·3 (5·0–12·7)	754 (405–1270)	0·1 (0–0·1)	19 700 (10 600–33 000)	2·1 (1·1–3·5)
*Shigella* spp	88 (33–194)	0 (0–0)	6510 (2390–13 700)	0·7 (0·3–1·5)	18 (2–48)	0 (0–0)	1030 (180–2590)	0·1 (0–0·3)
*Staphylococcus aureus*	83 300 (60 000–114 000)	8·9 (6·4–12·3)	1 710 000 (1 200 000–2 360 000)	183·1 (128·3–253·6)	19 100 (10 600–31 100)	2·0 (1·1–3·3)	396 000 (218 000–653 000)	42·5 (23·4–70·0)
*Streptococcus pneumoniae*	39 400 (30 800–50 300)	4·2 (3·3–5·4)	1 110 000 (883 000–1 400 000)	119·4 (94·8–150·8)	8230 (5440–11 500)	0·9 (0·6–1·2)	230 000 (153 000–319 000)	24·7 (16·4–34·3)
All pathogens	541 000 (370 000–763 000)	58·1 (39·7–81·9)	11 900 000 (8 190 000–16 700 000)	1278·5 (879·0–1794·0)	133 000 (90 100–188 000)	14·3 (9·7–20·2)	2 980 000 (2 020 000–4 210 000)	319·8 (216·5–451·8)

Data are estimates (95% uncertainty interval). Some counts are rounded to the nearest hundred. Estimates were aggregated across drugs, accounting for the co-occurrence of resistance to multiple drugs. For *N gonorrhoeae*, we did not estimate the fatal burden, thus only the DALY burden is presented. Group A *Streptococcus* pertains to *Streptococcus pyogenes*, and Group B *Streptococcus* pertains to *Streptococcus agalactiae*. AMR=antimicrobial resistance. DALYs=disability-adjusted life-years.

In a cross-country comparison of the overall resistance burden of leading pathogens, Russia (the most populous country in the region) had the highest mortality counts for strains of *E coli* resistant to any of the antibiotics studied, with 8551 deaths (95% UI 4988–13 737) attributable to and 31 977 deaths (20 183–47 980) associated with AMR. This was followed by *K pneumoniae* (5865 deaths [3836–8647] attributable to and 16 496 deaths [10 845–23 828] associated with AMR) and *S aureus* (3014 deaths [1549–5010] attributable to and 11 237 deaths [7445–16 184] associated with AMR; appendix 2 p 66, appendix 3 p 40).

More comparisons can be made with the use of crude and age-standardised mortality rates per 100 000 population. The highest crude mortality rates for *E coli* resistant to any antibiotic were seen in Bulgaria, with mortality rates per 100 000 of 7·29 (95% UI 4·01–12·13) attributable to and 29·29 (16·51–46·72) associated with AMR. Bulgaria also had the highest crude mortality rates for resistant *K pneumoniae*, with 4·59 deaths (2·63–7·46) attributable to and 14·70 deaths (8·85–22·95) associated with AMR, per 100 000 population. The highest crude mortality rates for resistant *S aureus* were observed in Portugal, with 5·65 deaths (3·33–8·52) attributable to and 24·39 deaths (19·26–30·91) associated with AMR, per 100 000 population (appendix 1 pp 46–47).

By contrast, the lowest crude mortality rates for *E coli* were observed in Iceland for deaths attributable to AMR (1·70 per 100 000 [95% UI 1·07–2·61]) and in Türkiye for deaths associated with AMR (7·04 per 100 000 [4·56–10·66]). For *S aureus*, the lowest crude mortality rates were observed in Sweden for deaths both attributable to (0·45 per 100 000 [0·28–0·70]) and associated with AMR (2·10 per 100 000 [1·51–2·87). For *K pneumoniae*, the lowest crude mortality rates were observed in Switzerland for deaths both attributable to (0·44 per 100 000 [0·28–0·68]) and associated with AMR (2·00 per 100 000 [1·40–2·81]; appendix 1 pp 46–47).

The highest age-standardised mortality rates associated with AMR for *E coli, K pneumoniae,* and *S aureus* were observed in Uzbekistan, with 16·2 deaths (for *E coli*), 13·6 deaths (for *K pneumoniae*), and 12·8 deaths (for *S aureus*) per 100 000. Uzbekistan also had the highest age-standardised mortality rates per 100 000 attributable to AMR for *E coli* (4·1 deaths) and *K pneumoniae* (3·7 deaths), whereas Türkiye had the highest AMR-associated *S aureus* mortality, with 2·9 deaths per 100 000. The lowest age-standardised mortality rates per 100 000 population for *E coli, K pneumoniae,* and *S aureus* were observed in Finland (4·2 deaths associated with and 0·9 deaths attributable to AMR for *E coli*), Switzerland (0·9 deaths associated with and 0·2 deaths attributable to AMR for *K pneumoniae*), and Sweden (0·9 deaths associated with and 0·2 deaths attributable to AMR for *S aureus*).

Regarding specific pathogen–drug combinations, methicillin-resistant *S aureus* was the leading combination in 27 countries (51% of the whole region) for deaths attributable to AMR, followed by *E faecium* resistant to fluoroquinolones in eight countries, and *E coli* resistant to third-generation cephalosporins in six countries (appendix 1 pp 31–34). Aminopenicillin-resistant *E coli* predominated as the leading pathogen–drug combination in 47 countries (89% of the region) for deaths associated with AMR (appendix 1 pp 31–34). A similar distribution was observed when DALYs for pathogen–drug combinations and both counterfactuals were compared (appendix 1 pp 35–38).

### AMR burden in the WHO European region by countries and age groups

**Figure 3:**
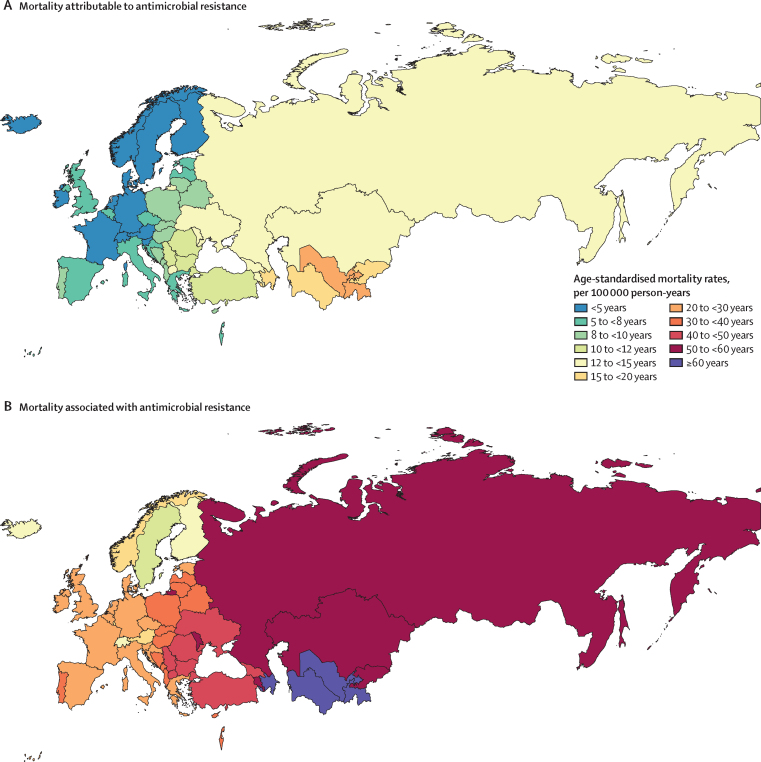
Cross-country comparison of age-standardised mortality rates per 100 000 person-years for deaths attributable to (A) and associated with (B) antimicrobial resistance in the WHO European region in 2019

**Figure 4:**
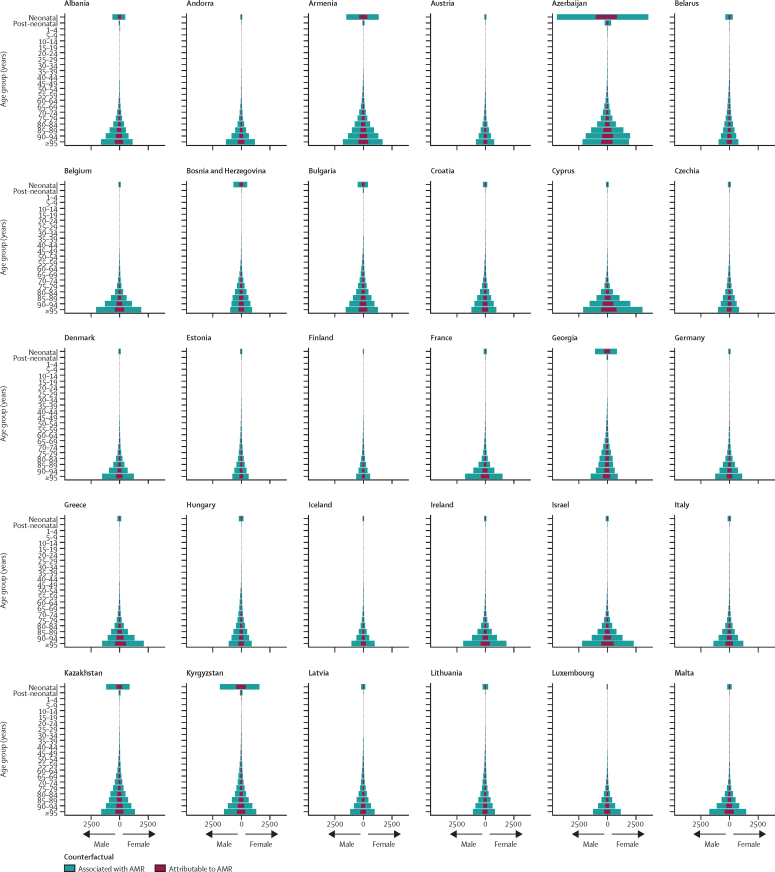

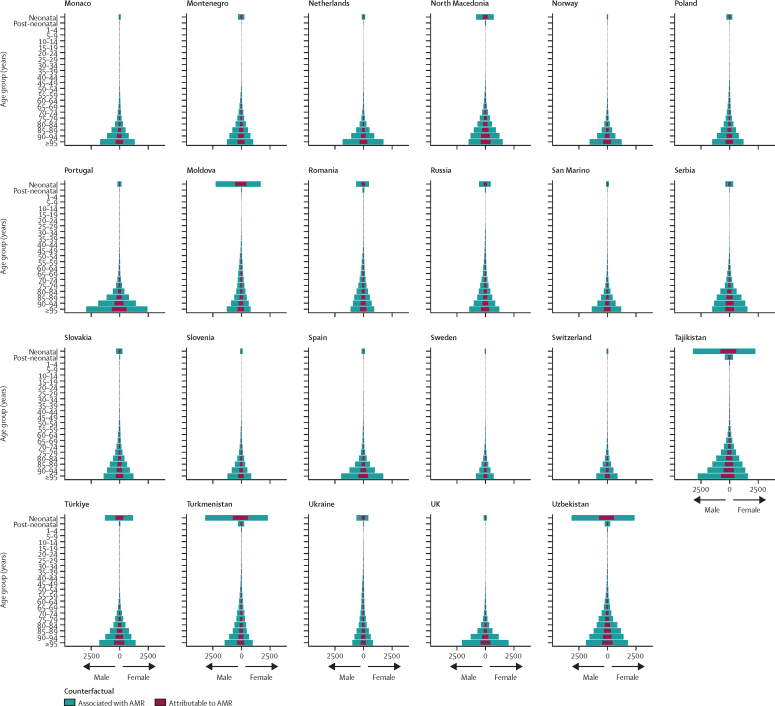
Age-specific mortality rates for deaths attributable to and deaths associated with AMR per 100 000 person-years by age group and country in 2019 AMR=antimicrobial resistance.

Accounting for age-standardised mortality rates per 100 000 person-years, a large part of the burden in the WHO European region was concentrated in countries of central Asia (which are considered by WHO as part of this region; figure 3). Five countries had an age-standardised mortality rate attributable to AMR larger than 15 deaths per 100 000 in 2019: in descending order, Tajikistan, Uzbekistan, Azerbaijan, Turkmenistan, and Kyrgyzstan (figure 3). Apart from Kyrgyzstan, these countries also had an age-standardised mortality rate associated with AMR larger than 60 deaths per 100 000, while the rest of the countries in the region fluctuated between 10 and 60 deaths per 100 000 (figure 3). The three countries with the lowest mortality rates per 100 000 were Switzerland (2·9 deaths attributable to and 13·1 deaths associated with AMR), Finland (2·8 deaths attributable to and 13·1 deaths associated with AMR), and Sweden (2·5 deaths attributable to and 11·8 deaths associated with AMR). Figure 4 shows a detailed breakdown of age-standardised mortality rates by age groups in each country of the region. Most deaths related to AMR—both attributable and associated—occurred among those aged 5 years or older, especially in older age groups. However, we also observed that in Azerbaijan, Kyrgyzstan, Moldova, Tajikistan, Turkmenistan, and Uzbekistan, the mortality rate among neonates exceeded that of older age groups.

**Figure 5:**
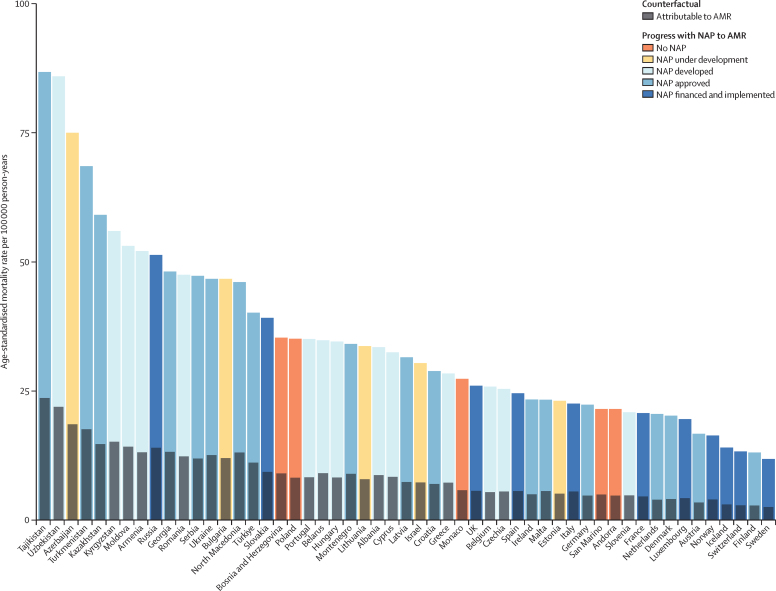
Age-standardised mortality rate associated with and attributable to AMR in relation to the status of NAPs for the countries in the WHO European region Rates per 100 000 person-years associated with AMR are coloured according to NAP status, while rates attributable to AMR are grey. The category “no NAP” also includes countries that did not provide data about its status. NAP data was acquired from the 2020–21 Country Self-assessment Survey responses.^21^ Estimates were aggregated across drugs, accounting for the co-occurrence of resistance to multiple drugs. AMR=antimicrobial resistance. NAP=national AMR action plan.

The countries in the region with developed, approved, financed, and implemented national AMR action plans^7^ were all situated in the lower 50th percentile of age-standardised mortality rates attributable to and associated with AMR, except Russia and Slovakia. By contrast, most countries with developed, but not yet approved national action plans had higher age-standardised mortality rates (figure 5). We observed a positive correlation between crude mortality rates associated with AMR and antibiotic consumption rates in defined daily doses per 1000 inhabitants per day for all antimicrobials or antimicrobial groups, except for trimethoprim–sulfamethoxazole. For example, the use of macrolides, one of the most widely prescribed groups of broad-spectrum antibacterials, had a moderate positive relationship (correlation coefficient *r*=0·48 [95% CI 0·24–0·67]; appendix 2 p 8). Additional figures are also available in appendix 1 (pp 46–55).

A correlation between crude mortality rates attributable to AMR and the SDI revealed a negative relationship (*r*=–0·49 [−0·67 to −0·26]; appendix 1 p 55). In other words, nations with a lower social development status (with implications for their health-care provision) generally had a larger AMR mortality burden (appendix 1 p 55). We observed a similar pattern when deaths associated with AMR were considered (appendix 1 pp 46–55).

## Discussion

To our knowledge, this study is the first comprehensive report of the burden of deaths both attributable to and associated with AMR for an extensive list of pathogens and pathogen–drug combinations for the entire WHO European region. In comparison with recently published global estimates,^5^ we have shown that the AMR burden in this region (with approximately 12% of the world's population) makes up 10·5% of the total estimated 1·27 million global deaths attributable to and 10·9% of the 4·95 million global deaths associated with AMR. Considering the estimated AMR burden within the landscape of infectious deaths in the WHO European region, we observe the substantial role that resistant microorganisms have in excess mortality; if all drug-resistant infections were replaced by no infection, 541 000 deaths might have been prevented in the WHO European region in 2019, whereas if all drug-resistant infections were replaced by drug-susceptible infections, 133 000 deaths might have been prevented.

Where specific pathogens are concerned, we have shown that *E coli* and *S aureus* present the largest burden of AMR in the WHO European region, which is consistent with results by Cassini and colleagues.^11^ These microbial species are recognised by WHO as priority pathogens,^22^ and their dominance is similar across the world.^5^ Methicillin-resistant *S aureus* and *E coli* resistant to third-generation cephalosporins are integral parts of the first SDG indicator for AMR (3.d.2), which was proposed in 2019 and will be further informed by studies akin to ours. A notable difference with previous European results is that our results indicate a larger burden of aminopenicillin-resistant *E coli* than that which is third-generation cephalosporin resistant, whereas Cassini and colleagues found the reverse*.*^11^ A large burden due to *K pneumoniae* in the region should be noted as well, reaching almost 20 000 attributable and almost 70 000 associated deaths. This is in line with other studies highlighting this pathogen as the fastest growing AMR threat in Europe in terms of human morbidity and mortality,^11^ fuelled primarily by intra-hospital and inter-hospital transmission.^23^

Patient exposure to antimicrobial agents is one of the most important factors in the selection of drug-resistant bacterial mutants. Generally, a positive correlation between use and resistance was evident for the WHO European region as a whole for most antimicrobials, and the relationship was usually the strongest for countries in western and central Europe. One of the exceptions was trimethoprim–sulfamethoxazole, but studies have already shown that variations in its use are not a major factor in determining the prevalence of resistance among several species (such as *E coli*), and that resistance rates to this antimicrobial can remain high despite decades of reduced consumption—primarily due to resistance co-selection in bacteria driven by genomically colocalised and yet laterally transferable resistance determinants, low fitness cost, and other mechanisms.^24^ We should also consider the unavailability of certain antimicrobials in some countries, different prescribing patterns (and sometimes treatment algorithms), as well as differences in the rate of change in classes of antibiotics consumed.^25^ Studies have also shown that the consumption of highly available cephalosporins, linked to the emergence of extended spectrum β-lactamase-producing Gram-negative bacteria,^26^ increased swiftly in low-income and middle-income countries, while at the same time declining in high-income countries.^26^

We used the SDI as a summary of overall development to determine underlying disparities in AMR burden between countries. Our results generally indicate that higher SDI scores translated to lower AMR mortality rates. Of course, AMR burden is dependent on the infectious burden in our study, which strongly correlates with SDI, so infection prevention in general might influence this result. Additionally, improved sanitation and hygiene, higher provider-to-patient ratio, and lower transmission of multidrug-resistant microorganisms in health-care institutions play an important role,^27^ as do differences in primary care provision across countries. A 2015 study assessing health-care spending in Europe showed that increased expenditure on private health care was associated with higher levels of AMR.^28^ However, for any steadfast conclusions, we would have to know how much data derive from private health care compared with state health care across Europe.

Although the availability of national action plans does not guarantee that specific strategies against AMR are used or enforced, we have seen that, in general, countries with such plans had lower rates of resistance burden (except for some outliers such as Russia). Hence, further efforts should be made to swiftly develop and implement national action plans in countries where this process is lagging. Prioritising dedication to and sustainability of these efforts and setting transparent review schedules (as delineated in the WHO Implementation Handbook and guidelines^29^) are key to ensuring the standardisation of these plans for easy comparability between countries of the WHO European region. Because lack of training and AMR awareness is identified in national action plans of some high-income countries (eg, Finland, France, and Sweden),^30^ it is important to address such gaps, thereby enhancing surveillance and antimicrobial stewardship endeavours to preserve (and even further improve) favourable AMR outcomes in these countries. Further analysis is warranted to appraise whether national action plans objectives can be seen as an indication of a country's existing activities or progress towards achieving targets to reduce AMR.

Compared with some other regions around the world, and notwithstanding differences among countries, surveillance of AMR in Europe is rather stringent. AMR and its consequences are already listed as a special health issue in different European Commission decisions on serious cross-border threats to health and on the communicable diseases and related special health issues to be covered by epidemiological surveillance.^31^, ^32^ For the WHO European region, there are two principal AMR surveillance sources that complement each other: the European Antimicrobial Resistance Surveillance Network (EARS-Net), which collects data from countries within the EU–EEA; and the Central Asian and European Surveillance of Antimicrobial Resistance network, which collects data from countries and areas within the WHO European region not included in EARS-Net (primarily eastern Europe and central Asia).^7^ Information from these surveillance systems is also reported to the WHO Global Antimicrobial Resistance Surveillance System.^7^

These sources represent the backbone of ECDC reports, as well as a 2022 joint ECDC-WHO–Europe report,^7^ but do not link to patients' hospital records or outcome. However, these sources were still used in our analysis to inform pathogen distribution, component models of prevalence of resistance, and data processing. ECDC reports are also based on antimicrobial susceptibility testing results from invasive isolates (from blood or cerebrospinal fluid) of eight bacterial pathogens relevant for public health in Europe: *E coli, K pneumoniae, P aeruginosa, Acinetobacter* spp, *S pneumoniae, S aureus, Enterococcus faecalis,* and *E faecium*.^8^, ^9^ This does not include *Mycobacterium tuberculosis* and several WHO priority pathogens^22^ that we have estimated in our study (such as *Salmonella* spp, *Shigella* spp, and *Neisseria gonorrhoeae*).

Cassini and colleagues provided estimates at the EU level for 16 pathogen–drug combinations in 2015.^11^ Here, we produced estimates for 11 of these 16 combinations; we did not estimate multidrug resistance in *P aeruginosa* or *A baumannii* due to our approach to multidrug-resistant infections (appendix 1 pp 20–25), and we did not estimate colistin resistance in *E coli, P aeruginosa,* or *A baumannii* because of data paucity on colistin resistance, which is an issue in the WHO European region in general.^8^ For the 11 pathogen–drug combinations that overlap, Cassini and colleagues estimated approximately 30 000 deaths and 796 000 DALYs caused by resistance in the EU in 2015. For these same 11 pathogen–drug combinations, Murray and colleagues^5^ estimated 23 100 deaths and 393 000 DALYs attributable to bacterial AMR for western and central Europe together; using the same modelling approach, in this Article, we have estimated 49 350 deaths and 1 073 000 DALYs attributable to bacterial AMR for these 11 combinations for the whole WHO European region in 2019. Although these results cannot be compared directly due to differing geographies and the use of a single-metric approach by Cassini and colleagues (versus our two counterfactuals), it is useful for contextualising the data and in refining the approach for future iterations of regional-level analyses.

When considering the downstream policy implications of this research, crucial methodological differences between our approach and the publication by Cassini and colleagues^11^ should be taken into account, as they can yield a rather divergent set of estimates. In our study, we have relied on both attributable and associated AMR burden to provide a scale of the upper and lower bounds of this issue. Importantly, due to our different numbers of data sources, larger number of pathogen–drug combinations, and specific methodological differences (appendix 1 pp 6–30), the use of the method devised by Cassini and colleagues^11^ might not result in death and DALY estimates between our two bounds of attributable or associated AMR burden. Therefore, any interpretation of estimates should address the main methodological approaches of different research groups for transparency.

Additionally, the work of Cassini and colleagues^11^ excluded *E coli* isolates that are resistant to colistin or carbapenem from their consideration of *E coli* resistant to third-generation cephalosporins. Conversely, our method included all isolates resistant to third-generation cephalosporins within the associated with AMR counterfactual for this pathogen–drug combination, regardless of co-resistance. Therefore, our estimates are higher than those of Cassini and colleagues for the associated with AMR counterfactual scenario. Furthermore, estimates by Cassini and colleagues^11^ were based on the incidence of each pathogen–drug combination calculated from ECDC data by using in-country expert opinion of population coverage; although most of the countries have mandatory reporting, this might overestimate coverage, possibly leading to underestimation of incidence and deaths.

Differences in the interpretation guidelines used for antimicrobial susceptibility testing should also be considered. Starting with the data collected in 2019, EARS-Net now accepts only antimicrobial susceptibility results generated with the use of European Committee on Antimicrobial Susceptibility Testing clinical breakpoints and methods.^8^, ^33^ This ensures compliance with the EU case definition for AMR and better comparability in the long run,^32^ but it also results in a lower number of participating laboratories (which was evident in the ECDC report for 2019). In this study, we have classified resistance by use of the most recent CLSI guidelines,^16^ based on minimum inhibitory concentrations provided in the data; in instances when such minimums were unavailable, we deferred to laboratory interpretation to classify the isolates. All isolates belonging to the intermediate resistance category in our study were classified as resistant.

This study has several limitations. Alongside the scarcity of data linking laboratory results to outcomes such as death, data paucity on the pathogen distribution by infectious syndrome and the prevalence of resistance data for key pathogen–drug combinations were also an issue, particularly for Moldova and all central Asian countries. Moreover, countries with low SDI might have less robust surveillance systems, as well as inadequate laboratory support, potentially resulting in an underestimation of mortality associated with and attributable to drug resistance in these countries. Nonetheless, our estimates are informed by data from all countries, and when data for a particular country were missing, estimates relied on regional patterns, covariates, and out-of-sample predictive validity. However, although the relative risk for each pathogen–drug combination can differ in accordance with infection, health-care access, age, and perhaps sex, data scarcity did not allow us to calculate the relative risk by this level of detail, necessitating our use of global relative risks.^5^

There are potential sources of bias and misclassification when combining and standardising data from a wide variety of providers, especially when dealing with mixed-classification or unclassifiable facilities or when delineating community-acquired from health-care-associated infections; however, this issue was of lower magnitude in comparison with our previous global estimation process. Although we have separate categories for community-acquired and hospital-acquired cases in our CFR models for lower respiratory and urogenital infections, we note the shortage of empirical data from community settings for other infectious syndromes. We are also cognisant that misattribution of burden might have occurred by relying on hospitalised patient data for CFR estimation, and that there might be some degree of overestimation by including intermediate antimicrobial susceptibility profiles as resistant. Selection bias in passive microbial surveillance data is also a potential issue, specifically if cultures for microbiological appraisal are not routinely drawn.^5^ Finally, further work will be needed to reconcile different approaches in the analysis of antimicrobial susceptibility testing reports, but also to account for the changing nature of breakpoint interpretation guidelines.^34^ The disaggregation of data in accordance with ethnicity was not pursued due to data scarcity. Regarding sex, the used algorithm assumes that the age-sex pattern of the death or case rate for a given infectious syndrome or pathogen is inherent to the pathology of the disease and is thus constant across location and year. Details on how the algorithm was applied have been previously published.^5^

Notwithstanding these limitations, our analysis represents the most comprehensive investigation of bacterial AMR burden in the WHO European region to date, reflecting the widest and currently best available range of data, as well as the use of models that have been implemented and honed specifically for incorporating disparate data sources for the GBD analysis. We echo other studies that highlight crucial AMR data gaps in some parts of the world,^35^ which will be of utmost importance going forward to further refine these estimates.

The high levels of resistance for several important bacterial pathogens and pathogen–drug combinations, together with the accompanying mortality and DALY burden shown in this study, show that bacterial AMR remains a serious challenge and a salient threat to public health in the WHO European region. Going forward, we recognise the need for improved evaluation criteria, the implementation of more usable data, novel strategies for data preparation, and the incorporation of new systematic literature reviews. Additionally, the specific behavioural traits of antibiotic prescribers, users, and providers require further investigation, as they are crucial for developing appropriate antimicrobial stewardship measures. Our research has provided a detailed description of the current situation; now, we need a benchmark for evaluating various mitigation strategies and pinpointing areas where policy impact can make a difference.

## Data sharing

This study follows the Guidelines for Accurate and Transparent Health Estimates Reporting. To download the data used in these analyses, please visit the Global Health Data Exchange (https://ghdx.healthdata.org/record/ihme-data/who-european-region-bacterial-antimicrobial-resistance-burden-estimates-2019).

## Declaration of interests

RA reports consulting fees from AbbVie; and payment or honoraria for lectures, presentations, speaker's bureaus, manuscript writing, or educational events from AbbVie, B Braun, Sandoz, and Laropharm, all outside the submitted work. CH and AP report grants from the Romanian National Authority for Scientific Research and Innovation, CNDS-UEFISCDI, for project number PN-III-P4-ID-PCCF-2016-0084 and project number PN-III-P2-2.1-SOL-2020-2-0351, outside the submitted work. CH also reports grants from the Romanian Ministry of Research Innovation and Digitalization MCID for project number ID-585-CTR-42-PFE-2021, outside the submitted work. A-FAM reports grants or contracts from MilkSafe, research co-financed by the EU European Regional Development Fund and Greek national funds through the operational programme Competitiveness, Entrepreneurship and Innovation, under the call RESEARCH–CREATE–INNOVATE (T2EDK-02222), as well as from ELIDEK (Hellenic Foundation for Research and Innovation, MIMS-860); stocks in a family winery; and other financial or non-financial interests in the BGI Group as a scientific officer, all outside of the submitted work. BS reports grants or contracts from the Fleming Fund; and leadership or fiduciary role in other board, society, committee or advocacy group, paid or unpaid, with the GBD Scientific Council and WHO RGHS, all outside the submitted work. AS reports support for the present manuscript from the Bill & Melinda Gates Foundation as payment to their institution, and paid participation on a Scientific Advisory Board for Vivli, outside the submitted work. All other authors declare no competing interests.
