# Supplementary material for: The burden of bacterial antimicrobial resistance in the WHO European region in 2019: a cross-country systematic analysis
Source: Lancet Public Health. 2022 Oct 14;7(11):e897–913. doi: 10.1016/S2468-2667(22)00225-0 (PMC9630253; doi:10.1016/S2468-2667(22)00225-0)
Supplement: Supplementary appendix 1 [file mmc1.pdf]

THE LANCET
Infectious Diseases

Supplementary appendix 1

This appendix formed part of the original submission and has been peer reviewed. We post it as supplied by the authors.

Supplement to: European Antimicrobial Resistance Collaborators. The burden of bacterial antimicrobial resistance in the WHO European region in 2019: a cross-country systematic analysis. *Lancet Infect Dis* 2022; published online Oct 13. https://doi.org/10.1016/S2468-2667(22)00225-0.

Appendix: supplementary methods and results to "The burden of antimicrobial resistance in the WHO European Region in 2019: a cross-country systematic analysis"

This appendix provides further methodological details and supplementary figures/tables for "*The burden of antimicrobial resistance in the WHO European Region in 2019: a cross-country systematic analysis*". Parts of the appendix are taken directly from the appendix of the paper "*Global burden of bacterial antimicrobial resistance in 2019: a systematic analysis*",[1] which is also referenced throughout the text.

Table of Contents

Section 1: Abbreviations 4

Section 2: Data sources...6

Section 2.1: Data sources for the WHO European Region..6

Section 2.2: Literature review details...7

Section 2.2.1: Maternal sepsis, neonatal sepsis, and LRI aetiology..7

Section 2.2.2: Urinary tract infections aetiology 8

Section 2.2.3: Skin infections aetiology...8

Section 2.2.4: Intra-abdominal infection aetiology 8

Section 2.2.5: Bone and joint infections aetiology...8

Section 2.2.6: Meningitis infection aetiology 8

Section 2.2.7: Relative risk studies for specific drug-bug combinations 8

Section 2.2.8: Prevalence of resistance for specific organisms..9

Section 2.3: Exclusion criteria for literature reviews 9

Section 3: Supplementary methods: a summary of the estimation process 10

Section 3.1: GBD 2019 framework..10

Section 3.2: Deaths where infection plays a role and infectious syndrome estimation 10

Section 3.2.1: Input data 10

Section 3.2.2: Data processing and mapping 10

Section 3.2.3: Intermediate cause and infectious syndrome mapping hierarchy with modelling pathways 11

Section 3.2.4: First pathway – deaths where infection plays a role 12

Section 3.2.5: Second pathway – fraction of deaths where infection plays a role by infectious syndrome in each GBD cause 12

Section 3.2.6: Model validation...13

Section 3.3: Case fatality ratios..13

Section 3.3.1: Input data 13

Section 3.3.2: Models ran for each infectious syndrome 14

Section 3.3.3: Modelling framework 14

Section 3.3.4: Predictions and uncertainty...15

Section 3.4: Pathogen distribution...15

Section 3.4.1: Input data 15

Section 3.4.2: Data processing and analysis 16

Section 3.4.3: Dealing with challenges in pathogen distribution appraisal...17

Section 3.4.4: Age-sex splitting and standardizing measures 18

Section 3.4.5: Modelling framework 18

Section 3.4.6: Exceptions and special handling...19

Section 3.4.7: Model validation 20
Section 3.5: Prevalence of resistance 20
Section 3.5.1: Input data 20
Table 3.5.1.1: Core pathogen–drug combinations 21
Table 3.5.1.2: Supplementary pathogen–drug combinations 21
Section 3.5.2: Data processing 22
Table 3.5.2.1: Pathogens in each pathogen super group 23
Section 3.5.3: Modelling framework 23
Section 3.5.4: Resistance profiles 24
Section 3.5.5: Model validation 25
Section 3.6: Relative risk 25
Section 3.6.1: Input data and data processing 25
Section 3.6.2: Modelling overview 26
Section 3.6.3: Model validation 27
Section 3.7: Counterfactuals and AMR estimation 27
Section 3.7.1: Estimating AMR burden with counterfactual of no infection 27
Section 3.7.2: Estimating AMR burden with counterfactual of infection with susceptible organism 28
Section 3.7.3: Excluded combinations 30
Section 3.8: Special considerations 30
Section 3.8.1: The use of defined daily doses (DDDs) and breakpoint interpretations 30
Section 4: Supplementary Tables and Figures 31
Section 5: GATHER Compliance: Guidelines for Accurate and Transparent Health Estimates Reporting 61
Section 6: PRISMA Compliance: Preferred Reporting Items for Systematic Reviews and Meta-Analyses 64
Prisma 2020 Checklist 64
PRISMA 2020 for Abstracts Checklist 70
PRISMA Diagrams 71
Section 7: References 85
Authors' Affiliations 87
Authors' Contributions 89

Section 1: Abbreviations

Abbreviation	Full phrase
AMASS	AutoMated tool for Antimicrobial resistance Surveillance System
AMR	antimicrobial resistance
ATLAS	Antimicrobial Testing Leadership and Surveillance
AWARE	Assessing Worldwide Antimicrobial Resistance Evaluation
BD	Becton, Dickinson, and Company
BSI	bloodstream infections
CAESAR	Central Asian and European Surveillance of Antimicrobial Resistance
CAI	community-acquired infection
CDC	Centers for Disease Control and Prevention
CFR	case fatality ratio
cIAI	complicated intra-abdominal infection
cUTI	complicated urinary tract infection
DALYs	Disability-adjusted life-years
DDD	Defined Daily Dose
DHS	Demographic Health Surveys
EARS-Net	European Antimicrobial Resistance Surveillance Network
ECDC	European Centre for Disease Prevention and Control
EEA	European Economic Area
EU	European Union
GAM	generalised additive models
GBD	Global Burden of Diseases, Injuries, and Risk Factors Study
GBS	group B *Streptococcus*
GLASS	Global Antimicrobial Resistance Surveillance System
GLM	generalised linear model
GPR	Gaussian process regression
HAI	hospital-acquired infection
HAQ Index	Healthcare Access and Quality Index
ICD	International Classification of Diseases

ICU	intensive care unit
INFORM	International Network for Optimal Resistance Monitoring
INICC	International Nosocomial Infection Control Consortium
iNTS	invasive non-typhoidal *Salmonella*
IORD	Infections in Oxfordshire Research Database
IQVIA	IMS Health and Quintiles
LRI	lower respiratory infection
MCoD	multiple causes of death data
MEPCO	multinomial estimation of partial and composite observations
MICS	Multiple Indicators Cluster Surveys
MR-BRT	meta-regression—Bayesian, regularised, trimmed
MRC	Medical Research Council
OUCRU	Oxford University Clinical Research Unit
PPS HAI	Point Prevalence Survey on Nosocomial Infections and Antibiotic Use
PRISMA	Preferred Reporting Items for Systematic Reviews and Meta-Analyses
SDI	Socio-demographic Index
SEV	summary exposure value
SGUL-GARPEC	St. George's Hospital, University of London - Global Antimicrobial Resistance, Prescribing and Efficacy Among Neonates and Children
SOAR	Survey on Antibiotic Resistance
ST-GPR	spatiotemporal Gaussian process regression
TB	tuberculosis
TESSy	The European Surveillance System
TEST	Tigecycline Evaluation Surveillance Trial
TSAP	Typhoid Fever Surveillance in Africa Program
UI	uncertainty interval
UTI	urinary tract infection
VR	vital registration
WHO	World Health Organization
YLDs	years lived with disability
YLLs	years of life lost

Section 2: Data sources

The data used for this study can be categorised into the following types: multiple causes of death (MCoD), hospital discharge, mortality surveillance, linkage data (mortality only), literature reviews, microbial data with and without outcome, single drug-resistance profiles, pharmaceutical sales, and antibiotic use data;[1] as well as estimates from the Global Burden of Diseases, Injuries, and Risk Factors Study (GBD) 2019.[2] Here we provide principal sources for data stemming from the WHO European Region and information on how we have conducted literature review. More detailed information on data inputs and sources are available in the appendix of Murray et al. (2022)[1] and http://ghdx.healthdata.org/record/ihme-data/global-bacterial-antimicrobial-resistance-burden-estimates-2019

Section 2.1: Data sources for the WHO European Region

- **UK Infections in Oxfordshire Research Database (IORD):** patient microbiology and episodes data from Oxford University Hospitals NHS Foundation Trust.
- **International Nosocomial Infection Control Consortium (INICC) surveillance online system:** data from the INICC data collection software. ICU patient microbiology and hospital data from 50 countries across Latin America, Asia, the Middle East, eastern Europe, and Africa from 2009 to 2020.
- **Bulgaria antimicrobial resistance data**: Medical University of Varna in Varna, Bulgaria. Covers 2014–2020.
- **St. George's Hospital, University of London - Global Antimicrobial Resistance, Prescribing and Efficacy Among Neonates and Children (SGUL-GARPEC) Project bloodstream infection data:** Penta-sponsored global surveillance network focusing on neonatal and paediatric antimicrobial resistance and the organisms causing blood stream infections.
- **SENTRY:** SENTRY Antimicrobial Surveillance Program established by JMI Labs in 1997. Sites are in the USA, Europe, Latin America, parts of Asia, and the Western Pacific
- **Germany National Point Prevalence Survey on Nosocomial Infections and Antibiotic Use (PPS HAI):** Point Prevalence Survey for 2016 data reporting the pathogen distribution for hospital-acquired infections.
- **AMASS:** data collected in an automated tool by Oxford Tropical Network Research Units.
- **The European Surveillance System (TESSy):** managed by the European Centre for Disease Prevention and Control (ECDC), provided data from the following surveillance systems:
    - European Antimicrobial Resistance Surveillance Network (EARS-Net)
    - Food-and Waterborne Diseases and Zoonoses Surveillance Network.
    - Invasive Pneumococcal Disease Surveillance Network, including discharge disposition.
    - Gonococcal Antimicrobial Surveillance Programme.
    - Healthcare Associated Infections Surveillance Network (ICU protocol), including discharge disposition.
    - European Tuberculosis Surveillance Network
    - European Surveillance of Antimicrobial Consumption Network

  For the European Union/European Economic Area (EU/EEA), data were obtained from the Euroepan Surveillance System (TESSy) as provided by Austria, Belgium, Croatia, Cyprus, Czechia, Denmark, Estonia, Finland, France, Germany, Greece, Hungary, Iceland, Ireland, Italy, Latvia, Luxembourg, Malta, Netherlands, Norway, Poland, Portugal, Romania, Slovakia, Slovenia, Spain, Sweden, and the United Kingdom, and released by the European Centre for Disease Prevention and Control (ECDC).

- **Pfizer ATLAS Programme:** the Antimicrobial Testing Leadership and Surveillance (ATLAS) database includes the Tigecycline Evaluation Surveillance Trial (TEST), the Assessing Worldwide Antimicrobial Resistance Evaluation (AWARE) and the International Network for Optimal Resistance Monitoring (INFORM) programs. The study spans in coverage across more than 70 countries between 2004 and 2017.
- **World Health Organization (WHO) Global Tuberculosis Programme**
- **Germany EARS-Net surveillance data 2017–2018**
- **GLASS:** Global Antimicrobial Resistance Surveillance System by WHO

- **CAESAR:** Central Asian and European Surveillance of Antimicrobial Resistance (CAESAR) is a network of national AMR surveillance systems and includes 19 countries in the WHO European Region that are not part of EARS-Net.
- **SOAR:** Survey on Antibiotic Resistance (SOAR) sponsored by GSK.
- **SMART:** Study for Monitoring Antimicrobial Resistance Trends which monitors complicated intra-abdominal infections (cIAIs), complicated urinary tract infections (cUTIs) and respiratory infections worldwide, funded by Merck & Co.

Section 2.2: Literature review details

We conducted literature searches to obtain input data for the following components in the analysis: maternal and neonatal sepsis aetiology, lower respiratory infections (LRIs) aetiology, urinary tract infections (UTIs) aetiology, skin infections aetiology, meningitis aetiology and case fatality, intra-abdominal infection aetiology, bone and joint infections aetiology, prevalence of resistance, relative risk and length of stay. Literature searches were performed on PubMed using the following search strings, and extracted studies covered the time range 1980–2019. The search string for these searches can be found below. Literature was used in the case fatality ratio, pathogen distribution, prevalence of resistance and relative risk component models and data processing, with details on modelling methods provided here and in the appendix of Murray et al. (2022).[1] Literature studies were also used as input into the modelling of the antibiotic usage covariate.[2]

Section 2.2.1: Maternal sepsis, neonatal sepsis, and LRI aetiology

Aetiology terms, combined with OR:

- Infection (Infect*)
- Microbiology (Microbiolog*
- Aetiology (Aetiolog*)
- Etiology (Etiolog*)
- Virology (Virolog*)
- Bacteriology (Bacteriolog*)
- Fungus (fung*)

AND

Syndrome terms, combined with OR:

Maternal Sepsis

- puerperal sepsis (puerper* sepsis)
- maternal sepsis (matern* sepsis)
- puerperal septicaemia (puerper* septicaemia, American spelling too - septicemia)
- maternal septicaemia (matern* septicaemia, American spelling too - septicemia)
- puerperal infection (puerper* infection)
- maternal infection (matern* infection)
- puerperal bacteraemia (puerper* bacteraemia, American spelling too - bacteremia)
- maternal bacteraemia (matern* bacteraemia, American spelling too - bacteremia)

Neonatal Sepsis

- Neonatal sepsis (Neonat* sepsis within 3 or 5 words of each other)
- Neonatal septicaemia (Neonat* septicaemia within 3 or 5 words of each other, American spelling too - septicemia)
- Infant sepsis (Infant* sepsis)
- Infant septicaemia (Infant* septicaemia, American spelling too - septicemia)
- Neonatal bacteraemia (Neonat* bacteraemia, American spelling too - bacteremia)
- Infant bacteraemia (Infant* bacteraemia, American spelling too - bacteremia)

Lower respiratory infections

- LRI
- Lower respiratory infection
- LRTI

- Lower respiratory tract infection
- Pneumonia

Section 2.2.2: Urinary tract infections aetiology

("complicated"[Title/Abstract] OR "uncomplicated"[Title/Abstract]) AND (("Cystitis/etiology"[majr:noexp] OR "Cystitis/microbiology"[majr:noexp]) OR ("Pyelonephritis/etiology"[marj:noexp] OR "Pyelonephritis/microbiology"[majr:noexp]) OR ("Urinary Tract Infections/etiology"[majr:noexp] OR "Urinary Tract Infections/microbiology"[majr:noexp])) OR ("Urinary tract infections"[tiab] AND ("etiology"[tiab] OR "microbiology"[tiab]))

Section 2.2.3: Skin infections aetiology

(("Cellulitis/epidemiology"[majr:noexp] OR "Cellulitis/etiology"[majr:noexp] OR "Cellulitis/microbiology"[majr:noexp]) OR ("Pyoderma/epidemiology"[majr:noexp] OR "Pyoderma/etiology"[marj:noexp] OR "Pyoderma/microbiology"[majr:noexp]) OR "Pressure Ulcer/microbiology"[majr:noexp])

Section 2.2.4: Intra-abdominal infection aetiology

(("Peritonitis/epidemiology"[majr:noexp] OR "Peritonitis /etiology"[majr:noexp] OR "Peritonitis /microbiology"[majr:noexp]) OR ("Intraabdominal infections/epidemiology"[majr:noexp] OR "Intraabdominal infections /etiology"[marj:noexp] OR "Intraabdominal infections /microbiology"[majr:noexp]) OR ("abdominal abscess/epidemiology"[majr:noexp] OR " abdominal abscess /etiology"[majr:noexp] OR "abdominal abscess/microbiology"[majr:noexp]))

Section 2.2.5: Bone and joint infections aetiology

("Osteomyelitis/etiology"[majr:noexp] OR "Osteomyelitis/microbiology"[majr:noexp] NOT 'chronic') OR ("Arthritis, infectious/etiology"[marj:noexp] OR "Arthritis, infectious/microbiology"[majr:noexp] NOT ‘lyme’)

Section 2.2.6: Meningitis infection aetiology

((meningitis[title]) AND (1990/05/01[PDat] : 2018/12/31[PDat]) AND ((etiolog*[title/abstract]) AND Humans[MeSH Terms])

Section 2.2.7: Relative risk studies for specific drug-bug combinations

("Acinetobacter baumannii"[MeSH Terms] AND "carbapenem resistance"[All Fields]) OR ("Acinetobacter baumannii"[MeSH Terms] AND "carbapenem resistant"[All Fields])

('Escherichia coli'[MeSH Terms] AND 'carbapenem resistance'[All Fields]) OR ('Escherichia coli'[MeSH Terms] AND 'carbapenem resistant'[All Fields])

('Escherichia coli'[MeSH Terms] AND 'fluoroquinolone resistance'[All Fields]) OR ('Escherichia coli'[MeSH Terms] AND 'fluoroquinolone resistant'[All Fields])

('Escherichia coli'[MeSH Terms] AND 'third generation cephalosporin'[All Fields]) OR ('Escherichia coli'[MeSH Terms] AND ESBL OR extended-spectrum beta lactamase'[All Fields])

('Klebsiella pneumoniae'[MeSH Terms] AND 'third generation cephalosporin'[All Fields]) OR ('Klebsiella pneumoniae'[MeSH Terms] AND 'ESBL OR extended-spectrum beta lactamase'[All Fields])

('Klebsiella pneumoniae'[MeSH Terms] AND 'carbapenem resistance'[All Fields]) OR ('Klebsiella pneumoniae'[MeSH Terms] AND 'carbapenem resistant'[All Fields])

('Streptococcus pneumoniae'[MeSH Terms] AND 'penicillin resistance'[All Fields]) OR ('Streptococcus pneumoniae'[MeSH Terms] AND 'penicillin resistant'[All Fields])

('Pseudomonas aeruginosa'[MeSH Terms] AND 'carbapenem resistant'[All Fields] AND 'mortality' [MeSH Terms]) OR ('Pseudomonas aeruginosa'[MeSH Terms] AND 'carbapenem resistant' AND 'mortality' [All Fields])

('Enterococcus faec*'[MeSH Terms] AND 'vancomycin-resistant'[All Fields])

("haemophilus influenzae"[MeSH Terms] AND ("penicillin resistance"[MeSH Terms] OR ("penicillin"[All Fields] AND "resistance"[All Fields]) OR "penicillin resistance"[All Fields])) AND ("mortality"[Subheading] OR "mortality"[All Fields] OR "mortality"[MeSH Terms])

("streptococcus agalactiae"[MeSH Terms] AND ("azithromycin resistance"[MeSH Terms] OR ("azithromycin "[All Fields] AND "resistance"[All Fields]) OR " azithromycin resistance"[All Fields] OR "penicillin resistance"[MeSH Terms] OR ("penicillin"[All Fields] AND "resistance"[All Fields]) OR "penicillin resistance"[All Fields] OR "clindamycin resistance"[MeSH Terms] OR ("clindamycin"[All Fields] AND "resistance"[All Fields]) OR "erythromycin resistance"[All Fields] OR "erythromycin resistance"[MeSH Terms] OR ("erythromycin"[All Fields] AND "resistance"[All Fields]) OR "clindamycin resistance"[All Fields]) AND ("mortality"[Subheading] OR "mortality"[All Fields] OR "mortality"[MeSH Terms])

Section 2.2.8: Prevalence of resistance for specific organisms

Medical Subject Heading (MeSH) terms with free text terms in the title and abstract fields for Escherichia coli, Klebsiella pneumoniae, Streptococcus pneumoniae and Staphylococcus aureus with the terms for antimicrobial drug resistance (resistan*, suscept*, surveil*, etc), limited from 1990 up to the search date. The search was undertaken on MEDLINE, Ovid Embase, Global Health, Cochrane Library.

Medical Subject Headings (MeSH) and free text terms for the pathogens of interest (e.g. S. Typhi, S. Paratyphi A, enteric fever) with terms for antimicrobial resistance (e.g. resistan*, suscept*, surveil*). The search was undertaken on MEDLINE, Ovid Embase, Global Health, Cochrane Library, Scopus, Web of Science-Core Collection and LILACS regional WHO database.

Medical Subject Heading (MeSH) terms with free text terms in the title and abstract fields for non-typhoidal Salmonella or Salmonellosis (non-typhi or nontyph or non-typh Salmonel…) with the terms for antimicrobial drug resistance (resistan*, suscept*, surveil*, etc) and invasive (blood stream infection, septicaemia etc), limited from 1990 up to the search date. The search was undertaken on MEDLINE, Ovid Embase, Global Health, Cochrane Library, Scopus, Web of Science-Core Collection and LILACS regional WHO.

Medical Subject Heading (MeSH) terms with free text terms in the title and abstract fields for Shigella or Shigellosis with the terms for antimicrobial drug resistance (resistan*, suscept*, surveil*, etc), limited from 1990 up to the search date. The search was undertaken on MEDLINE, Ovid Embase, Global Health, Cochrane Library, Scopus, Web of Science-Core Collection and LILACS regional WHO database.

Medical Subject Heading (MeSH) terms with free text terms in the title and abstract fields for Neisseria gonorrhoeae, with the terms for antimicrobial drug resistance (resistan*, suscept*, surveil*, etc), MDR, XDR, limited from 1990 up to the search date. The search was undertaken on MEDLINE, Ovid Embase, Global Health, Cochrane Library, Scopus, Web of Science-Core Collection and LILACS regional WHO database.

Section 2.3: Exclusion criteria for literature reviews

Studies were excluded from full text review if:

- The study did not include at least one of the following: *E.coli, K.pneumoniae, S.pneumoniae, S.aureus or S.typhi/paratyphi*
- The entire study was conducted before 1990
- Samples were collected before 1990
- Did not perform resistance testing
- Sample is non-representative (lab strains, only resistant strains)
- Included non-human samples
- Article type was a case study
- Article type was a commentary, editorial or review with no primary data
- Isolates were not from blood culture

- There were duplicated isolates
- Travellers/non-endemic country/ no location information
- Study did not test susceptibility to antimicrobials
- There were fewer than 10 consecutive isolates used for susceptibility testing
- Could not locate the full text
- The study was uninterpretable due to poor data quality
- Studies where data was aggregated with other pathogens
- Studies using non-sterile site/mixed isolates
- Studies with no iNTS AST data

Section 3: Supplementary methods: a summary of the estimation process

Section 3.1: GBD 2019 framework

The study relies on Global Burden of Disease (GBD) 2019 fatal and non-fatal estimates, and a comprehensive description of data sources, data quality, statistical modelling and analyses for GBD 2019 have been reported elsewhere.[2] A brief summary of the fatal and non-fatal estimation processes can be found in the appendix of Murray et al. (2022).[1]

Section 3.2: Deaths where infection plays a role and infectious syndrome estimation

Section 3.2.1: Input data

Multiple causes of death (MCoD) data are individual-based records that provide underlying causes of death and two or more intermediate causes in the chain of death. Additionally, each record includes age, sex, residence, and the date of death.

Hospital record with multiple diagnoses and discharge status of death represents an individual-based hospital record of a patient that provides the main diagnosis and two or more additional diagnoses. Additionally, each record includes age, sex, residence, date of admission, date of discharge, and outcome (dead or alive). Only hospital discharges with discharge status of death were used in this component model, since we aimed to estimate the fraction of deaths that involve infection and the infectious syndrome distribution of those deaths.

Linkage data are generated using probabilistic methods in a defined population that link individual-based hospital data to individual-based MCoD data. Linkage data offer a wider dataset that includes main diagnosis, other diagnoses, underlying cause of death, and intermediate causes of death in the chain.

Section 3.2.2: Data processing and mapping

Within the WHO European region, data for Italy has been extracted at the subnational level by GBD 2019 age groups, sex, year, and causes of death and/or diagnoses, while data for the remaining countries have been analysed at the national level. This allowed us to expand the location-years of data that we had for each Socio-demographic Index (SDI)[3] value.

Prepared data were mapped to GBD causes. The GBD cause list is a mutually exclusive and collectively exhaustive list of diseases and injuries. The GBD cause list is organised hierarchically to accommodate different purposes and needs of various users. The first two levels aggregate causes into general groupings. At Level 1, there are three cause groups: communicable, maternal, neonatal, and nutritional diseases (Group 1 diseases); non-communicable diseases (Group 2); and injuries (Group 3). These Level 1 aggregates are subdivided at Level 2 of the hierarchy into 22 cause groupings (eg, neonatal disorders, neurological disorders, and transport injuries). The disaggregation into Levels 3 and 4 contains the finest level of detail for causes captured in GBD 2019. See section 14, table S1 for the full GBD cause hierarchy by level.

The underlying cause of death or main diagnosis for each record in the data was mapped to a GBD cause. After the mapping of underlying cause, we used the GBD 2019 garbage code redistribution algorithm (see appendix 1, section 2.4 in Vos et al.[2]) to ensure that all deaths had a plausible and specific underlying cause of death. The redistribution

of garbage codes for underlying causes of death followed the same age and sex restrictions as GBD 2019. We did not redistribute garbage codes in the chain causes because the concept of a garbage code applies only to plausible underlying cause of death (see Rudd et al.[4] and appendix 1, section 2.5 in Vos et al.[2]).

Section 3.2.3: Intermediate cause and infectious syndrome mapping hierarchy with modelling pathways

Within our modelling framework, an infectious syndrome is the infection directly responsible for sepsis and serves as the bridge between the underlying cause of death and sepsis. Infectious syndromes can be both underlying causes of death and intermediate causes of death.

For mapping underlying and intermediate causes of death and hospital diagnoses to sepsis and infectious syndromes, we designed a new map, called "*AMR, sepsis, and infectious syndrome map*". This map is a list of mutually exclusive and collectively exhaustive infectious syndromes that we divided into four levels to form the infectious syndrome hierarchy.

Each level of infectious syndrome is mutually exclusive and collectively exhaustive. Furthermore, the infectious syndrome hierarchy is internally consistent across any metric (eg, number, cause fraction)—aggregating across Level 3 syndromes gives us Level 2 syndromes, aggregating the Level 2 syndromes gives us Level 1 syndromes, and the total of Level 1 syndromes is equal to the value of sepsis (figure 4.4.2.1).

Level 0: All International Classification of Diseases 9th (ICD-9) or 10th revision (ICD-10) coded deaths divided into three groups: explicit sepsis (any death with the specific ICD code for sepsis in the MCoD chain or hospital diagnoses), implicit sepsis (any death with an infectious disease code in the underlying cause or cause chain, as well as with a specific organ dysfunction) and non-sepsis (any death that does not meet either of the two aforementioned criteria). More information can be found in the appendix of Murray et al. (2022).[1]

Explicit sepsis (A40, R65.2 in ICD-10 and 039 in ICD-9): Any death has specific ICD code for sepsis in the MCoD chain or hospital diagnoses was considered explicit sepsis.[4]

- Implicit sepsis: Any death that has an infectious disease code in the underlying cause or cause chain and a specific organ dysfunction code was considered implicit sepsis
- Non-sepsis: Any death that does not meet either of the two above criteria (section 14, tables S2, S3)

Of the estimated infection-related deaths with explicit sepsis or implicit sepsis and infectious diseases, 59.4% occur with communicable, maternal, neonatal, and nutritional underlying causes of death. 38.9% infection related deaths occur with non-communicable disease as the underlying cause of death, and 1.7% occur with injuries as the underlying cause of death.

Level 1: All implicit and explicit sepsis deaths were divided into 12 Level 1 infectious syndromes and an "other" category. These are as follows: 1) Bacterial infections of the skin and subcutaneous systems; 2) Bloodstream infections; 3) Gonorrhoea and chlamydia; 4) Diarrhoea; 5) Endocarditis and other cardiac infections; 6) Infections of bones, joints and related organs; 7) Lower respiratory infections and all related infections in the thorax; 8) Meningitis and other bacterial central nervous system infections; 9) Peritoneal and intra-abdominal infections; 10) Tuberculosis; 11) Typhoid, paratyphoid, and invasive non-typhoidal *Salmonella;* 12) Urinary tract infection and pyelonephritis; 13) Other infections

Level 2: Each Level 1 infectious syndrome was divided into Level 2 infectious syndromes based on the pathogen type (eg, bacterial, fungal, viral) causing the infection. Examples include specified bacterial, unspecified bacterial, fungal, viral, and unspecified pathogen.

Level 3: Each specified bacterial infectious syndrome in Level 2 was divided to Level 3 infectious syndromes by the culprit bacterial pathogen. Table S3 (section 14) shows this list and bacterial hierarchy.

Due to our data often having multiple diagnoses associated with each record, a single case of sepsis could potentially map to multiple candidate infectious syndromes. Because multiple infectious syndrome assignments pose a risk of double counting, we employed an informative ranking hierarchy. The informative ranking allowed us to determine the infectious syndrome that provided the most information on the culprit pathogen. The goal of this hierarchy was

to produce the most accurate pathogen burden estimate such that when there were multiple infectious syndromes, we prioritised the syndrome with the most distinctive distribution. For example, bloodstream infections (BSIs) are common infections in sepsis but there is often an earlier source of the infection such as a UTI, cellulitis, or LRI, and each has a unique pathogen distribution that provides more information than the distribution of BSI. In the event that a patient record reflected both BSI and LRI, we would assign the infectious syndrome based on the pathogen distribution that would be the most proximal aetiologic syndrome, LRI (please refer to the appendix of Murray et al. (2022)[1] for more information).

After mapping the underlying and chain causes of death, our database went through two separate modelling pathways. The first model estimated the fraction of deaths that are sepsis-related in each GBD cause; these sepsis-related deaths for non-infectious GBD causes were combined with GBD deaths for infectious causes to create the total envelope of all deaths where infection plays a role. The second pathway estimated each infectious syndrome as a fraction of sepsis-related mortality in each GBD cause. In the last step of infectious syndrome estimation, the fractions of sepsis by Level 1 infectious syndromes were squeezed to sum to one so as to not exceed the sepsis mortality envelope and multiplied by the sepsis estimate in each GBD cause by country and territory, age, and sex in 2019.

Section 3.2.4: First pathway – deaths where infection plays a role

We used a mixed-effects binomial logistic regression to model the logit of the fraction of sepsis-related deaths by GBD cause-age-sex-location, consistent with the modelling approach used by Rudd et al.[4] Sex and Healthcare Access and Quality Index (HAQ Index)[2] were included as covariates and a nested random effect on underlying cause of death was included. A separate model was run for each GBD 2019 age group (0–6, 7–27, 28-364 [days], 1–4, 5–9, 10–14, 15–19, 20–24, 25–29, 30–34, 35–39, 40–44, 45–49, 50–54, 55–59, 60–64, 65–69, 70–74, 75–79, 80–84, 85–89, 90–94, 95+ [years]):

$$sepsis\ related\ deaths \sim B(total\ deaths, sepsis\ fraction) \qquad (3.2.4.1)$$

$$logit(sepsis\ fraction) = \beta_0 + \beta_1 * HAQ\ Index + \beta_2 * sex + \pi_{level\ 1, level\ 2}$$

Where $\pi_{level\ 1, level\ 2}$ is a nested random effect on underlying cause of death. The nested random-effect's structure in the model on underlying cause of death allowed the prediction of sepsis fractions where data were limited by borrowing information from diseases within the same group. There were 22 groups of underlying causes of death, each categorised by physiological relatedness. We produced our predictions and uncertainty intervals (UIs) by generating 1000 draws from the normal distribution of the fixed coefficients, separately for each GBD location, age group, sex, and cause in 2019. The means of our results were used for the point estimates and the 95% UIs were delineated using the 2.5^{th} and 97.5^{th} percentiles of the draws. Uncertainty is attributable to sample size variability between data sources, data availability, and model specifications.

All underlying causes of death that are infectious diseases were included in the model; however, for these causes we used the GBD death estimates rather than the modelled sepsis estimate, since infection inherently plays a role in these deaths even if the pathway doesn't include sepsis. These causes and their associated infectious syndromes are available in the appendix of Murray et al. (2022)**.**[1] For all other causes, we calculated the number of sepsis-related deaths in 2019 by multiplying our predictions of cause-, age group-, sex-, year-, and location-specific sepsis fractions by GBD 2019 death estimates. Finally, we aggregated our results to arrive at regional and global sepsis-related mortality in non-infectious underlying causes of death, which we combined with the GBD infectious disease deaths estimates to create the mortality envelope of all deaths related to infection.

Section 3.2.5: Second pathway – fraction of deaths where infection plays a role by infectious syndrome in each GBD cause

We used a mixed-effects binomial logistic regression to model the logit of the infectious syndrome fraction of sepsis-related mortality by GBD cause. The model covariates varied by infectious syndrome, and all models

included HAQ Index as a covariate and most included a summary exposure value (SEV) scalar calculated for GBD 2019. To more accurately estimate the burden of pathogens responsible for infection, we separated infectious syndromes into hospital-acquired and community-acquired for LRI+ and UTI. More details on the infectious syndrome model covariates and age groups are found in the appendix of Murray et al. (2022).[1]

The infectious syndrome models were specified as mixed-effects binomial logistic regressions, one for each infectious syndrome and age group:

$$syndrome\ related\ deaths \sim B(total\ sepsis\ deaths, syndrome\ fraction) \quad (3.2.5.1)$$

$$logit(syndrome\ fraction) = \beta_0 + \beta * X + \pi_{level\ 1, level\ 2}$$

where β and X are vectors of length $n+1$ for n covariates and $\pi_{level\ 1, level\ 2}$ is a nested random effect on underlying cause of death. The granularity of the age groups estimated for each infectious syndrome was chosen based on the age pattern of the infectious syndrome and the limitations of data sparsity.

As in the first pathway, we derived our predictions and UIs by generating 1000 draws from the normal distribution of the fixed coefficients separately for each GBD location, age group, sex, and cause in 2019. We used the means of our results for the point estimates and the 95% UIs were delineated using the 2.5^{th} and 97.5^{th} percentiles of the draws.

We calculated the number of deaths attributable to each infectious syndrome in 2019 by multiplying our predictions of cause-, age group-, sex-, year-, and location-specific infectious syndrome fractions by our sepsis-mortality estimates from the first pathway. All infectious syndrome fractions were squeezed to sum to one prior to multiplication in order to ensure that we did not exceed the sepsis mortality envelope.

Out of the 12 explicit Level 1 infectious syndromes included in our hierarchy, we excluded (i) tuberculosis (TB), (ii) typhoid, paratyphoid, and invasive non-typhoidal *Salmonella*, and (iii) gonorrhoea and chlamydia from our binomial mixed-effects linear regression model. Instead, we used the published results from GBD 2019[3] for these causes of death, as we believe the GBD 2019 estimates fully represent these infectious syndromes because they are usually not intermediate causes of death.

Section 3.2.6: Model validation

Infectious syndrome modelling aims to predict which cases of infection belong to a specific infectious syndrome, which is a multi-class classification problem. We therefore use the Area Under the Receiver Operating Characteristics (ROC) Curve (AUC) to evaluate model performance. The ROC Curve is determined by the sensitivity (or true positive rate) and the specificity (or false positive rate) of the model, and a higher AUC score indicates that the model is capable of discerning between the different categories. Accuracy is a related measure which considers the proportion of true positives and true negatives predicted by the model with respect to the total number of predictions. More information on this can be found in the appendix of Murray et al. (2022).[1]

Section 3.3: Case fatality ratios

Section 3.3.1: Input data

Case fatality ratios (CFRs) were modelled for the pathogens and infectious syndromes of interest using all available data detailing the organism responsible for infection, the infectious syndrome, and patient outcome, which included hospital and microbial data. Input data for the CFR models were aggregated based on data source, year, GBD location, and age group (as well as hospital/community acquired status, in the case of the lower respiratory and urogenital infectious models). For lower respiratory and blood stream infections, for which CFRs could be vastly different in neonates, we modelled the following age groups: neonatal, post-neonatal–5 years, 5–50 years, 50–70 years, and 70 years and older. For all other infectious syndromes, we modelled the following age groups: neonatal–5 years, 5–50 years, 50–70 years, and 70 years and older. We excluded from the analysis any source-location-year-age with fewer than five cases and zero deaths.

To allow us to implement linear models, CFRs were logit-transformed. We used the delta method to compute the standard error of CFRs in logit space. To incorporate data with zero deaths, or with an equal number of deaths and cases, we applied a 1% offset, such that the CFRs for data with zero deaths was represented as 1% and the CFR for data with an equal number of deaths and cases was represented as 99%.

Pathogen-specific CFRs were modelled separately by infectious syndrome and were calculated as a function of HAQ Index and age. To account for heterogeneity across the sources of input data, we implemented a mixed-effects meta-regression framework, modelling data source as a random effect. We further incorporated a binary fixed-effect denoting whether the data source only included intensive care unit (ICU) patients, for which CFRs were expected to be higher. The pathogens of interest for each infectious syndrome were determined by prevalence in the data and expert opinion, with the goal of modelling approximately 90% of specified-pathogens associated with each infectious syndrome.

Section 3.3.2: Models ran for each infectious syndrome

The interaction of the HAQ Index fixed-effect with the pathogen-specific fixed-effect allowed the relative deadliness of pathogens to vary depending on a location's HAQ Index – this is termed an 'interaction model'. For those pathogens with fewer than ten high quality data points below 0.7 HAQ Index, or those whose results in the interaction models indicated an unrealistically large influence of HAQ Index (eg, 70% CFR in low HAQ Index countries, 1% CFR in high HAQ Index countries), we modelled a pathogen-specific intercept with an HAQ Index fixed-effect shared across the pathogens. As a consequence of the single fixed-effect on HAQ Index, a pathogen that was predicted to be the deadliest in low HAQ Index countries would also be predicted to be the deadliest in high HAQ Index countries in these 'intercept models.' To estimate the CFRs for other known bacteria, which either were not selected as a pathogen of interest or lacked sufficient data for inclusion in the intercept models, we pooled all bacterial data together and estimated a single CFR curve from age, HAQ Index, and the data source heterogeneity covariates. Thus, up to three models were run for each infectious syndrome:

1) an interaction model including data for all data rich pathogens and 'other specified bacteria' (which was included to inform the overall influence of HAQ Index on CFR, predictions were only generated for the data rich pathogens),
2) an intercept model including data for data rich and data sparse pathogens, as well as 'other specified bacteria' (predictions were only generated for the data sparse pathogens), and
3) an 'other bacteria' model that included data for all bacterial pathogens (predictions were generated by HAQ Index and age, without any pathogen specific term).

For some infectious syndromes, the relative deadliness of a pathogen may be strongly determined by either the age of the patient or whether the infection was community- or hospital-acquired. For bloodstream infections, we ran two distinct sets of CFR models, one for neonates (0–27 days) and another for post neonates, to capture the differing dynamics of pathogen deadliness in these two populations. As is done for our other modelling processes, we also separate community-acquired and hospital-acquired cases in our CFR models for lower respiratory and urogenital infections. Because some data sources did not provide enough information to infer whether an infection was community- or hospital-acquired, but still included important information on the relative pathogenesis and the difference in CFRs across varying HAQ indices, infections of unknown origin were included in both the community-acquired and hospital-acquired models for these two syndromes. Any bias in these 'unknown origin' infections was adjusted for using a binary fixed-effect representing an 'unknown origin' infection, and predictions were generated for the community- and hospital-acquired infections only.

Section 3.3.3: Modelling framework

The data were analysed using a meta-analytic mixed effects structure. The main model can be specified as follows:

$$logit(y_i) = X_i\beta + u_i 1 + \epsilon_i, \qquad \epsilon_i \sim N(0, \Sigma_i), \qquad u_i \sim N(0, \gamma) \tag{3.3.3.1}$$

where

- y_i contains CFRs for data source i

- Design matrix X_i contains as columns the following covariates
  - in all models:
    - HAQ Index
    - dummy-coded indicator for age group
    - dummy-coded ICU indicator for data source (1 if data source only compiles information on ICU patients, 0 if a mix between ICU/non-ICU patients)
  - in 'interaction' and 'intercept' models:
    - dummy-coded indicator for pathogen
  - in 'interaction' models only:
    - interaction between pathogen and HAQ Index (product of dummy-coded pathogen columns and HAQ Index)
  - in models evaluating community/hospital acquired infection (LRI+, UTI):
    - dummy-coded variable indicating source of infection (1 if unknown source, 0 if community OR hospital acquired, depending on whether the model is evaluating community or hospital infections)
- β are fixed effect multipliers
- ϵ_i are observation error terms with known variances
- u_i are data source-specific random intercepts with unknown covariance γ

The underlying program used to fit the model (meta-regression, Bayesian, regularized, trimmed [MR-BRT]) is described elsewhere.[5] The program allows specification of priors on γ and β.

Section 3.3.4: Predictions and uncertainty

Predictions for 2019 CFRs were generated for each country, age group, and pathogen as a function of each country's HAQ Index, assuming mixed ICU/non-ICU patients and, in the case of models for UTI and LRI+, that the infection was community- or hospital-acquired (in contrast to infections of unknown origin). For pathogens with insufficient data to estimate a syndrome-specific CFR, we predicted out using the 'other bacteria' CFR associated with the infectious syndrome. Importantly, all of the CFRs we calculate by infectious syndrome are independent of that syndrome's underlying cause.

Uncertainty estimates were generated using asymptotic uncertainty intervals. Specifically, for the model, the posterior uncertainty for the coefficients β is Gaussian, with mean and variance given below:

$$\hat{\beta} = \left(\sum_i X_i^T V_i^{-1} X_i\right)^{-1} \left(\sum_i X_i^T V_i^{-1} y_i\right) \qquad (3.3.4.1)$$

$$Var(\hat{\beta}) = \left(\sum_i X_i^T V_i^{-1} X_i\right)^{-1} \qquad (3.3.4.2)$$

where

$$V_i = 11^T + \hat{\gamma} I \qquad (3.3.4.2)$$

The variance-covariance matrix was used to obtain 1000 draws for the coefficients, which are then used to get intervals for the predictions.

Section 3.4: Pathogen distribution

Section 3.4.1: Input data

With this model, we aimed to estimate the distribution of pathogens causing each infectious syndrome. To get input data for this model, we gathered all available data sources described in section 2 that meet the following criteria:

- Sufficient diagnosis (for patient- or admission-level datasets) or sample specimen type (for isolate- or culture-level datasets) information for us to determine the infectious syndrome

- Information on which pathogen(s) caused the infection or which pathogen(s) were detected in an infectious sample, as determined through culture or genomic-based methods
- Did not have a strongly biased sampling framework across pathogens (for example, did not deliberately sample until 100 cases of every pathogen of interest had been obtained)

The input data source types that met these criteria in this study were:

- Multiple causes of death data
- Hospital discharge
- Linkage data
- Microbial data with and without outcome information
- Literature studies from the aetiology literature reviews

Section 3.4.2: Data processing and analysis

We extracted and standardised the location, year, age, sex, diagnoses, specimen type, pathogens, and hospital- and community-acquired (HAI and CAI) status of each record in every dataset. These datasets report a variety of metrics, including deaths, admissions, cases, cultures, and isolates. While these metrics are not completely comparable (for example, a single patient may often have multiple cultures taken during a single hospital admission), we chose to standardise them into two categories: "deaths," for any unit associated with an outcome of death, and "cases," for any unit regardless of outcome. After standardising the data, we mapped every sample ID or tabulated figure in the data to infectious syndrome based on its diagnoses and specimen type. More details on this process can be found the appendix of Murray et al. (2022).[1]

Some pathogens cause disease so rarely or are so commonly contaminants that we considered them to be contaminants, unlikely to be the true cause of disease. Examples include many *Corynebacterium* species and *Staphylococcus epidermidis*. We dropped all such contaminants from the analysis, as well as any record listed by treating clinicians in the data as a contaminant. We also dropped from the analysis all records where no pathogen was detected, or the patient diagnosis indicated an unspecified bacterium. This assumes that the distribution of pathogens among cases with known aetiology are the same as those with unknown aetiology; in other words that the probability of detection is the same for every pathogen. This assumption may break down if certain pathogens are more difficult to detect than others, or in cases where a pathogen is irregularly tested for within a laboratory.

For data sources where multiple pathogens were listed per sample ID, we classified these cases according to the following criteria. First, if a case contained more than one of "unspecified bacteria," "virus," "fungus," and another pathogen(s), we chose to drop all these pathogens except the one(s) most likely to be responsible for disease, with the following ranking from most to least likely: 1. Another pathogen(s); 2. Unspecified bacteria; 3. Virus; 4. Fungus. This was to drop co-occurrence profiles that we consider to be uninformative, like a viral infection co-occurring with a fungal infection. After applying this drop, we considered any sample ID that contained more than one pathogen to be polymicrobial. Polymicrobial was treated as a distinct pathogen category in all further analysis, and we were unable to include any AMR burden from polymicrobial infections in our final results, which possibly underestimates the burden of AMR by hiding infections caused by resistant pathogens of interest in the polymicrobial category.

Furthermore, in our approach we chose to assume that the relative prevalences of pathogens in datasets that do not report co-occurrence would be comparable to their mono-pathogenic counterparts in datasets that do report co-occurrence. This assumes that the co-occurrence of pathogens is random and is not correlated for certain pathogens. We did not have sufficient data to fully test the validity of this assumption, given that few datasets report the full universe of pathogens which may co-occur. When selecting pathogens for estimation, we took into account that the set of estimated pathogens for each infectious syndrome is mutually exclusive and collectively exhaustive of all possible aetiologies. Polymicrobial infections were either estimated explicitly or included in the "other" category, making all explicitly estimated individual pathogens mono-pathogenic. Additional factors that were considered can be found in the appendix of Murray et al. (2022).[1]

Section 3.4.3: Dealing with challenges in pathogen distribution appraisal

One of the central challenges of estimating pathogen distributions was that not every data source tested for or reported every possible aetiology of a given infectious syndrome. For example, many literature studies on the aetiologies of meningitis only report on bacterial aetiologies, and some surveillance systems only collect data on certain pathogens of interest. Only certain pathogens are referenced explicitly in the International Classification of Diseases (ICD), limiting which pathogens can be identified from ICD-based data types like MCoD and hospital discharge. Finally, some datasets reported only a subset of the pathogens that we are interested in for a given infectious syndrome, reporting the remaining aetiologies in an aggregate "other" category. These practices have led to inconsistencies in the "other" and "polymicrobial" categories across data sources. Datasets can either over or under-report "other," and datasets that report fewer specific pathogens will automatically report fewer polymicrobial infections.

To address this problem, we maintained a list of data sources that we believe have sufficient testing and reporting to give unbiased estimates of other and polymicrobial for all syndromes, dropping any data on polymicrobial or other that did not come from these data sources. These data sources all had a complete sampling framework (eg, they do not limit the scope of aetologies that they test for) and reported their results without any deliberate aggregation. While we believe this list provided an accurate starting place for the estimation of other and polymicrobial, future work to improve this method would involve a more detailed analysis of sampling framework and reporting categories in each dataset, specific to each infectious syndrome.

There were two major exceptions to this method for handling "other specified pathogens." First, determining the pathogenic aetiology of LRI with microbiology represents challenges that have been well described previously.[6,7] In order to account for this limitation, we utilised a vaccine probe design to inform the *Streptococcus pneumoniae* cause fraction of LRI, consistent with the approach used in the GBD aetiology estimation process.[8,9] In brief, we extracted the vaccine efficacy of the pneumococcal vaccine against all pneumonia from 18 vaccine probe studies with randomised-control trial, before-after, and cohort designs among children and adults. We then calculated the PAF of pneumonia due to *S. pneumoniae* in each study ($Strep\ Base\ PAF$) based on these vaccine efficacies ($VE_{all\ pnuemonia}$), the vaccine efficacy of pneumococcal vaccine against vaccine-type pneumococcal pneumonia as pooled from three studies (two in children and one in adults) (VE_{vtpp}), the percentage of the population covered by the pneumococcal vaccine as modelled in GBD (100% for RCTs) (Cov_{PCV3}),[9] and the percent of serotypes covered by the vaccine[10] ($Cov_{serotype}$) (equation 6.2.6.1). We modelled a global age-specific PAF for *S. pneumoniae* based on these data in the MR-BRT environment and finally adjusted this PAF based on the vaccine coverage in children in every GBD location in 2019 and optimal vaccine efficacy in children ($Strep\ Final\ PAF$) (equation 3.4.3.2). In adults (age 5+), we assumed the effects of vaccination on adults would be primarily indirect from vaccination in children, and included an adjustment factor on the vaccine efficacy to account for this, derived from Grijalva et al.[11]

$$Strep\ Base\ PAF = \frac{VE_{all\ pneumonia}}{VE_{vtpp}Cov_{PCV3}Cov_{serotype}} \qquad (3.4.3.1)$$

$$Strep\ Final\ PAF = \frac{Strep\ Base\ PAF\left(1 - Cov_{PCV3}Cov_{serotype}VE_{PCV3\ Optimal}\right)}{1 - (Strep\ Base\ PAF)Cov_{PCV3}Cov_{serotype}VE_{PCV3\ Optimal}} \qquad (3.4.3.2)$$

In this vaccine probe analysis, $(1 - Strep\ Final\ PAF)$ is not consistent with the "other" category in our model, since it includes all non-*S. pneumoniae* aetiologies. We retained all of the data from the vaccine probe analysis as two categories, *S. pneumoniae* and "not *S. pneumoniae*" and addressed the inconsistencies between them and our other data using our modelling framework.

The second major exception involves several literature studies on the proportion of neonatal bacterial meningitis caused by *Streptococcus agalactiae* (Group B *Streptococcus;* GBS). We found that these literature studies were important to our estimation of the pathogen distribution of neonatal meningitis, which is distinct from other age groups because of its high proportion of GBS. However, these studies either only reported or were only extracted

with two categories, GBS and “other bacterial, not GBS.” We retained both these categories and addressed the inconsistencies between them and our other data using our modelling framework.

Section 3.4.4: Age-sex splitting and standardizing measures

We standardised age and sex across all datasets to the following most-detailed groups using the GBD causes of death age-sex splitting algorithm for age:[2] 0–6, 7–27, and 28–364 days, and 1–4, 5–9, 10–14, 15–19, 20–24, 25–29, 30–34, 35–39, 40–44, 45–49, 50–54, 55–59, 60–64, 65–69, 70–74, 75–79, 80–84, 85–89, 90–94, 95+ years; and sex: male and female. This algorithm assumes that age-sex pattern of the death or case rate for a given infectious syndrome or pathogen is inherent to the pathology of the disease and is therefore constant across location and year. Details on how the algorithm was applied can be found in the the appendix of Murray et al. (2022).[1]

The input data sources reported a variety of combinations of measures, including some that reported deaths only, some that reported cases only, and some that reported both cases and deaths. In order to standardise these measures to cases, we estimated infectious syndrome- and pathogen-specific CFRs (see section 5) and used these CFRs to convert all deaths-only datasets to cases. For any infectious syndrome or pathogen combination for which we did not have enough data to estimate plausible CFRs, we used a set of all-bacteria CFRs for that infectious syndrome instead. All modelling was done in case space.

Several of our microbial databases came exclusively from ICUs and were therefore heavily biased towards severe illness. In order to mitigate this bias, we dropped all information on cases in ICU-only datasets and recalculated implied cases based on reported deaths and our CFRs. No similar adjustment was made to attempt to account for biases between hospitalised and un-hospitalised populations, although we did account for HAI versus CAI for two infectious syndromes – LRI and thorax infections and UTI – within our modelling framework. The use of hospital-based data to calculate both pathogen-specific case fatality ratios and pathogen distributions biases our estimate of the distribution of pathogens in incident cases towards more severe disease, particularly for less-severe infectious syndromes like lower respiratory infections; adjusting for this bias would improve the accuracy of our non-fatal estimates

Section 3.4.5: Modelling framework

To model the distribution of pathogens for each infectious syndrome, we developed a method for the multinomial estimation of partial and compositional observations (MEPCO). We assumed that the aetiologies of a given infectious syndrome followed a multinomial distribution. Due to inconsistencies in which pathogens are tested for and reported by different data sources, each data source contained partial observations of the possible outcomes of the underlying multinomial distribution. Certain data sources like the vaccine probe estimates and the GBS neonatal meningitis studies represent compositional observations, where pathogens like “not *S. pneumoniae”* and “other bacterial, not GBS” represent aggregates of more detailed pathogens.

In order to use both partial and compositional data, we constructed a network model with the dependent variable as the log ratio of cases between different pathogens and estimated over a flexible parameterisation of multinomial parameters using a maximum likelihood approach. Consider a given infectious syndrome with a multinomial distribution of n mutually exclusive, collectively exhaustive aetiologies with probabilities $p = (p_1, \ldots, p_n)$, so that each $p_j \in (0,1)$ and $\sum_j p_j = 1$. The likelihood of an observation of $c = (c_1, \ldots, c_n)$, where c_j = number of cases of pathogen j in a total sample of N infections ($\sum_j c_j = N$), is:

$$P(c|p) = N! \prod_{j=1}^{n} \frac{p_j^{c_j}}{c_j!} \qquad (3.4.5.1)$$

We modelled the probabilities using a composition of a link function with a linear predictor:

$$p_{i,j} = \exp\left(x_{i,j}^T \beta_j\right) \qquad (3.4.5.2)$$

for observations i, a vector of covariates $x_{i,j}$, and a vector of coefficientsβ_j for each pathogen j. the appendix of Murray et al. (2022)[1] contains a table with the covariates used for infectious syndrome model, which included a typical specification included an intercept term, HAQ Index, a categorical age group dummy for large age bins, and

any relevant vaccine coverage proportions by country. However, we did not observe these probabilities directly. Rather, we observed ratios between sums of these probabilities, which reduce to ratios between sums of cases within each study. These observations therefore take the form:

$$y_i = \frac{cases\ of\ pathogen\ A}{cases\ of\ pathogen\ B} = \frac{\sum_{j=1}^{n} w_{i,j}^{a} \exp\left(x_{i,j}^{T}\beta_j\right)}{\sum_{j=1}^{n} w_{i,j}^{b} \exp\left(x_{i,j}^{T}\beta_j\right)} \qquad (3.4.5.3)$$

where $w_{i,j}^{a}$ is a weight of 0 or 1 that selects the mutually exclusive, collectively exhaustive most-detailed pathogens that make up observed pathogen A, which may be a composite observation. For example, for the "other bacterial, non-GBS" pathogen, $w_{i,j}$ would be 1 for *Staphyloccocus aureus, S. pneumoniae, Haemophilus influenzae, Neisseria meningitidis, Listeria monocytogenes, K. pneumoniae, E. coli*, and other pathogens and 0 for GBS and virus. We dropped all observations where either the numerator or denominator had 0 observed cases in order to make this calculation and a forthcoming log transform possible. This may bias the model towards overestimating less common pathogens.

It is not possible to infer all coefficients β_j from the observations, since they are all relative. However, if we fix all of the coefficients for one pathogen to 0 as a reference group, then we obtain a well-posed inverse problem, as long as there is enough data to estimate the remaining coefficients. Without loss of generality, we assumed $\beta_1 = 0$ for all elements and obtain estimates of the remaining $\beta_2, \dots, \beta_n$ by minimising the sum of the residuals between log-transformed observations y and corresponding log-transformed predictions from equation 3.3.5.4:

$$\min_{\beta_2,\dots,\beta_n} f(\beta) := \sum_i \frac{1}{\sigma_i^2}\left[\ln(y_i) - \ln\left(\sum_{j=1}^{n} w_{i,j}^{a}\, exp\left(x_{i,j}^{T}\beta_j\right)\right) + \ln\left(\sum_{j=1}^{n} w_{i,j}^{b}\, exp\left(x_{i,j}^{T}\beta_j\right)\right)\right]^2 \qquad (3.4.5.4)$$

where σ_i^2 are variances corresponding to the data points. Equation 3.3.5.4 is a nonlinear likelihood minimisation problem that that we optimised using a standard implementation of the Gauss-Newton method.[12] We then re-normalised the optimal coefficients to obtain final predictions of the probabilities of each pathogen:

$$p_{i,j} = \frac{\exp\left(x_{i,j}^{T}\beta_j\right)}{\sum_{\hat{j}} \exp\left(x_{i,\hat{j}}^{T}\beta_{\hat{j}}\right)} \qquad (3.4.5.5)$$

To quantify the uncertainty of this estimate, we used asymptotic statistics to obtain the posterior distribution of $(\beta_2, \dots, \beta_n)$. Specifically, using the Gauss-Newton Hessian approximation gave us the asymptotic information matrix for all β_j except for the reference pathogen, allowing us to sample draws of $\beta = (\beta_1 = 0, \beta_2, \dots, \beta_n)$. For each β draw and given feature x, we obtained a corresponding draw of p using equation 3.3.3.5.

Finally, to convert $p_{i,j}$ for a given demographic group i from case space to deaths space, we transformed using our CFR estimate for demographic i:

$$p_{i,j}^{deaths} = \frac{p_{i,j} \times CFR_i}{\sum_{\hat{j}} p_{i,\hat{j}} \times CFR_i} \qquad (3.4.5.6)$$

This network regression with covariates framework allowed us to use partial and composite data that reported on one or only a few pathogens, or that reported multiple pathogens aggregated together. Networks, however, can be unstable with sparse data and stable estimates have in some cases required the use of Bayesian priors in these models. In particular, we imposed Gaussian priors with mean 0 and non-zero variance on all coefficients except intercepts, to bias the model away from spurious effects driven by data sparsity. These priors were based on expert opinion and can improved with further empirical validation in the future (appendix of Murray et al.[1]).

Section 3.4.6: Exceptions and special handling

There were several notable exceptions and special handling decisions made for each individual pathogen distribution model, which we hope to address with more sustainable approaches in our future work. For example, for cardiac

infections, we used the pathogen distribution for bloodstream infections rather than estimating specific distributions for these syndromes, due to a lack of complete literature reviews on the aetiologies and case-fatality rates of these syndromes. We consider this to be a serious limitation of our methodology, but do not anticipate that is seriously impactful on our final estimates.

In diarrhoea patients, cultures of specimens taken from the gastrointestinal tract, bowels, rectum, or stool are almost always affected by contaminants or pathogens that are not the cause of diarrhoea. For this reason, we believe that our input data and modelling framework are not able to accurately capture the aetiologies of diarrhoea. We chose to use GBD estimates of the aetiologies of diarrhoea in deaths instead of running our own model.[13] Nonetheless, a major limitation of using such approach is that the GBD diarrhoea aetiology estimates are population attributable fractions (PAFs) for each pathogen. These PAFs may add to greater than 1 and the authors made no attempt to quantify the extent of co-occurrence of pathogens; the latter is inconsistent with the pathogen distribution estimation method used in our study, which quantifies polymicrobial infections and estimates all pathogens as mono-infections. Hence, in order to avoid duplication of cases in our framework, we had to make some assumptions about the co-occurrence of pathogens in diarrhoea (details provided in the appendix of Murray et al.[1]).

Certain skin and subcutaneous samples are easily affect by contaminants, colonization, and other pathogens that are not the cause of infection. For this reason, we considered microbial data and mortality surveillance to be too difficult to extract meaningful aetiology information from, and instead used only ICD-coded databases (multiple cause of death, hospital discharge, and linkage data) and literature studies as inputs into our model of the pathogen distribution of skin infections.

We dropped all data on *S. pneumoniae* for community-acquired LRI and thorax infections in non-neonatal age groups except our estimates from the vaccine probe analysis. Because dedicated anaerobic cultures were not routinely performed for peritoneal samples, we dropped all anaerobes observed in the data for and excluded anaerobes as an etiology of intra-abdominal infections. Moreover, due to the unique pattern of meningitis in neonates, particularly the high prevalence of GBS, we modeled neonatal and adult central nervous syndrome infections separately.

For three infectious syndromes, we did not run a pathogen distribution model – these are “Typhoid, paratyphoid, and invasive non-typhoidal *Salmonella*”, “Tuberculosis” and “Gonorrhoea and chlamydia” infectious syndromes. They are all caused by distinct pathogens whose individual burdens are already estimated in GBD as separate causes of death. Therefore, for these syndromes, we simply used GBD estimates.

Section 3.4.7: Model validation

To assess model validity, we calculated the root mean square error (RMSE) and coefficient of determination (R^2) for each pathogen distribution model in proportion space for both in-sample and out-of-sample predictions. Proportions were predicted for each observation using the specific denominator observed from that study. For example, if a given study reported on only *E. coli* and *S. pneumoniae,* the predictions for model validation for this study were calculated as proportions of the total for *E. coli* and *S. pneumoniae.* In order to calculate out-of-sample fit, we perform non-exhaustive cross-validation, with each round of the validation holding out 1 country of data at a time. This leave-one-country-out approach simulates the prediction task of estimating the pathogen distribution of a country for which we have no data. As evidenced in the appendix of Murray et al. (2022),[1] it was shown that our models have a good fit and good out-of-sample predictive ability.

Section 3.5: Prevalence of resistance

Section 3.5.1: Input data

We identified line level and aggregate data on the prevalence of resistance in bacterial pathogens, which were linked to the country and year in which the infection was acquired, from datasets obtained from pharmaceutical companies, surveillance networks, academic institutions, and individual hospitals (see section 2). We supplemented microbiological data with systematic reviews following the Preferred Reporting Items for Systematic Reviews and Meta-Analyses (PRISMA) guidelines,[14] to collect resistance data published from countries and territories where

surveillance systems do not routinely collect data to ensure extensive coverage of the pathogen–drug combinations thought to contribute the greatest burden of drug resistant infections, which we termed core pathogen–drug combinations (table 3.5.1.1). Data on the prevalence of AMR in these pathogen–drug combinations were extracted from published literature and compiled into comprehensive datasets. The systematic reviews followed similar methodologies; a detailed description can be found either in published literature (*S.* Typhi and *S.* Paratyphi[15]) or in the corresponding PROSPERO records (*E.* coli, *K. pneumoniae, S. aureus* and *S. pneumoniae* PROSPERO registration CRD42019145148; *Shigella* species PROSPERO registration CRD42019127603; iNTS PROSPERO registration CRD42020189935; *N. gonorrhoeae* SPF unique identifier osf.io/4vy5n). The *S.* Typhi and *S.* Paratyphi A systematic review was expanded to include non-blood culture isolates for the current analysis. Forms were created, and screening and data extraction were completed using web-based systematic review software (DistillerSR, Evidence Partners, Ottawa, Canada) for all pathogens except *Salmonella,* for which a smaller number of manuscripts were identified.

For the prevalence of drug resistance in *Mycobacterium tuberculosis* for multi-drug resistance (MDR, characterised by isoniazid and rifampicin co-resistance) excluding extensive drug resistance (XDR, characterised by resistance to isoniazid, rifampicin, and fluoroquinolone, as well as either aminoglycosides or capreomycin) and XDR, we used previously published GBD results.[2] To more comprehensively account for the burden of AMR in bacteria, we also estimated the prevalence of resistance for 71 supplementary pathogen–drug combinations for which we did not conduct a systematic literature review. Data for these supplementary combinations were extracted from the datasets obtained from pharmaceutical companies, academic institutes, and individual hospitals using the same processing procedure as was used for the core pathogen–drug combinations. The list of supplementary combinations is presented in table 3.5.1.2.

Table 3.5.1.1: Core pathogen–drug combinations

Pathogen	Antimicrobial
Escherichia coli	Third-generation cephalosporins<br>Fluoroquinolones
Klebsiella pneumoniae	Third-generation cephalosporins<br>Carbapenems
Staphylococcus aureus	Methicillin
Streptococcus pneumoniae	Penicillin
Salmonella Typhi & Paratyphi A	Multidrug resistance<br>Fluoroquinolones
Invasive non-typhoidal *Salmonella*	Fluoroquinolones
Shigella species	Fluoroquinolones
Neisseria gonorrhoeae	Third-generation cephalosporins
Mycobacterium tuberculosis	Isoniazid mono-resistance, Rifampicin mono-resistance

Table 3.5.1.2: Supplementary pathogen–drug combinations

Pathogen	Antimicrobial
Acinetobacter baumannii	Aminoglycosides, Anti-pseudomonal penicillin/Beta-lactamase inhibitors, Beta-lactam/Beta-lactamase inhibitors, Carbapenems, Third-generation cephalosporins, Fourth-generation cephalosporins, Fluoroquinolones
Citrobacter species	Aminoglycosides, Anti-pseudomonal penicillin/Beta-lactamase inhibitors, Carbapenems, Third-generation cephalosporins, Fourth-generation cephalosporins, Fluoroquinolones
Enterobacter species	Aminoglycosides, Anti-pseudomonal penicillin/Beta-lactamase inhibitors, Carbapenems, Fourth-generation cephalosporins, Fluoroquinolones, Trimethoprim-Sulfamethoxazole
Enterococcus faecalis	Fluoroquinolones, Vancomycin
Enterococcus faecium	Fluoroquinolones, Vancomycin
Enterococcus species	Fluoroquinolones, Vancomycin

Escherichia coli	Aminoglycosides, Aminopenicillin, Beta-lactam/Beta-lactamase inhibitors, Carbapenems, Trimethoprim-Sulfamethoxazole
Group A *Streptococcus*	Macrolide
Group B *Streptococcus*	Fluoroquinolones, Macrolide, Penicillin
Haemophilus influenzae	Aminopenicillin, Third-generation cephalosporins
Klebsiella pneumoniae	Aminoglycosides, Beta-lactam/Beta-lactamase inhibitors, Fluoroquinolones, Trimethoprim-Sulfamethoxazole
Morganella species	Third-generation cephalosporins, Fourth-generation cephalosporins, Fluoroquinolones
Neisseria gonorrhoeae	Fluoroquinolones
Proteus species	Aminoglycosides, Aminopenicillins, Third-generation cephalosporins, Fluoroquinolones, Trimethoprim-Sulfamethoxazole
Pseudomonas aeruginosa	Aminoglycosides, Anti-pseudomonal penicillin/Beta-lactamase inhibitors, Carbapenems, Third-generation cephalosporins, Fourth-generation cephalosporins, Fluoroquinolones
Serratia species	Aminoglycosides, Anti-pseudomonal penicillin/Beta-lactamase inhibitors, Carbapenems, Third-generation cephalosporins, Fourth-generation cephalosporins, Fluoroquinolones
Staphylococcus aureus	Fluoroquinolones, Macrolide, Trimethoprim-Sulfamethoxazole, Vancomycin
Streptococcus pneumoniae	Beta-lactam/Beta-lactamase inhibitors, Carbapenems, Third-generation cephalosporins, Fluoroquinolones, Macrolide, Trimethoprim-Sulfamethoxazole

Group A *Streptococcus = Streptococcus pyogenes.* Group B *Streptococcus = Streptococcus agalactiae*

Section 3.5.2: Data processing

The prevalence of resistance for each pathogen–drug combination was calculated for each data source, by country and year. Whenever possible, we classified resistance using the most recent CLSI guidelines based on the MICs provided in the data. When MICs were unavailable, we deferred to lab interpretation to classify the isolates. All isolates determined to have intermediate resistance were classified as resistant. To determine the prevalence of resistance to a class of antibiotics (eg, fluoroquinolones), resistance to any one of the antibiotics in the class was sufficient to classify an isolate as resistant for line level data (ie, susceptibility data for individual isolates). For aggregate data (ie, the proportion of isolates resistant to various antibiotics), the highest prevalence of resistance to any antibiotic in the class was selected. Multidrug resistance in *Salmonella* species was defined as concurrent resistance to ampicillin/amoxicillin, chloramphenicol, and trimethoprim-sulfamethoxazole; and fluoroquinolone resistance was defined as ciprofloxacin minimum inhibitory concentration of 0.125 µg/ml or higher, or nalidixic acid resistance (CLSI breakpoint for *Salmonella* spp. were updated in 2012 to include 0.125 µg/ml as isolates with 'decreased ciprofloxacin susceptibility', and we have considered these as resistant). Nalidixic acid resistance was also used as a proxy for fluoroquinolone non-susceptibility for *Shigella* species.

To account for biased level of resistance found in tertiary care settings, we reviewed all input data used for the prevalence of resistance estimation and classified each data source as either tertiary, non-tertiary, or unknown/mixed designation, which was a commonly used classification for large resistance surveillance networks which don't report on the hospitals they collect data from. We located datasets that either provided facility information at the line-level or reported samples from exclusively tertiary or non-tertiary facilities. Where possible, we used tertiary/non-tertiary assignments from the data providers. When no assignments were available, we classified sites as primary, secondary and following the definitions provided by Jamison et al.,[16] as described in the appendix of Murray et al. (2022).[1]

Because the degree of bias in resistance between tertiary and non-tertiary data could vary, we ran a separate crosswalk for each super region and pathogen–drug *super group* combination. Certain bacteria and antimicrobials were clustered into super groups to provide the models with more robust input data, though, crucially, while a given model would contain several pathogen–drug combinations in its inputs, every matched pair was made comparing tertiary and non-tertiary values for the same combination. Bacteria were classified as follows (excluding those that would be robust to tertiary care bias, as well as *Morganella* spp. due to no input data for that pathogen from tertiary facilities):

Table 3.5.2.1: Pathogens in each pathogen super group

Pathogen super group	Incorporated pathogens
Gram-positives	*Enterococcus faecalis*, *Enterococcus faecium, Enterococcus* spp., Group A *Streptococcus*, Group B *Streptococcus*, *Staphylococcus aureus, Streptococcus pneumoniae*
Enterobacterales	*Citrobacter* spp., *Enterobacter* spp., *Escherichia coli, Haemophilus influenzae, Klebsiella pneumoniae, Proteus* spp., *Serratia* spp.
Pseudomonadales	*Acinetobacter baumannii, Pseudomonas aeruginosa*

Only one group of antimicrobials was clustered to create an antimicrobial super group, the β-lactam group, which was comprised of: aminopenicillin, anti-pseudomonal penicillin, β-lactamase inhibitors, carbapenems, third and fourth generation cephalosporins, methicillin, and penicillin. All other antibiotic classes (aminoglycosides, fluoroquinolones, macrolides, sulfanoamides, and vancomycin) each individually comprised their own antimicrobial super group.

To allow us to implement linear models, resistance values were logit-transformed. We used the delta method to compute the standard error of the prevalence of resistance in logit space. To incorporate data with zero resistance, or with complete resistance, we applied a 0.1% offset, such that the prevalence of resistance for data with zero resistance was represented as 0.1% and the prevalence of resistance for data with total resistance was represented as 99.9%. We then used the MR-BRT modelling framework to estimate the logit difference of tertiary and non-tertiary data for each super region-pathogen/antimicrobial 'super combination', including a random effect for each pathogen–drug combination within the super combination and employing a positivity prior to enforce the constraint that the tertiary data exceed or be equal to the non-tertiary data.

After modelling the difference between tertiary and non-tertiary data, we implemented the models to adjust all the country-level tertiary input data that was indicated as biased. We then used the adjusted prevalence of resistance estimates from tertiary care facilities and unadjusted prevalence of resistance from non-tertiary/mixed care facilities as data inputs for the prevalence of resistance models. As was done before, resistance values were offset prior to logit-transformation to allow the use of linear models; data with zero resistance or complete resistance was offset by 2%. Exceptions to this offset were made for two combinations, *Staphylococcus aureus*/vancomycin and Group B *Streptococcus*/penicillin, which were anticipated to often have values beneath 2% resistance. For these combinations, we applied a 0.5% offset instead.

Section 3.5.3: Modelling framework

The prevalence of AMR in each pathogen–drug combination was modelled separately. For the core combinations, excluding *N. gonorrhoeae*/3GC, we selected a range of spatially- and temporally-explicit health and socio-demographic-related covariates with biologically plausible associations to the prevalence of AMR in each pathogen from the Global Health Data Exchange (http://ghdx.healthdata.org/), and from published literature.[17] This list was narrowed down by fitting a lasso penalised regression model between the data and the covariates for each dataset (using the 'glmnet' package version 3.0.2 in R version 3.6.1) and selecting the most influential covariates in each of the pathogen–drug models to be taken forward. For the supplementary pathogen–drug combinations and *N. gonorrhoeae*/3GC, we utilised a standard set of covariates for all models: HAQ Index, pigs per capita (as a proxy for antibiotic use in animal husbandry), mean temperature, and antibiotic consumption of the antibiotic class relevant to each pathogen–drug combination. Determining more individualised sets of covariates for each of these supplementary pathogen–drug combinations is an ongoing focus for future extensions of this research. All of the covariates used in our models are available in the appendix of Murray et al. (2022).[1]

Due to the high heterogeneity of the input datasets, we outliered data points found to have the most extreme values for the prevalence of resistance. An initial generalised linear model (GLM) was fit to the data and covariates and input data points that lay outside of two times the median absolute deviation from the modelled estimate for each

location were determined to be outliers and removed. The GLM was fit with nested random effects based on the location to capture spatial effects, and was fit using the ‘lme4’ package version 1.1-21 in R version 3.6.1.

After the removal of extreme values, the datasets were used to fit spatiotemporal statistical models of the prevalence of AMR. Firstly, we used a stacked ensemble model to fit the associations between selected covariates and data. For each of the pathogen–drug combinations, we considered the following child models for inclusion: generalised additive models (GAM), penalised regression models (elastic-net, ridge, lasso), random forest, cubist, and neural-networks. Models were fit in R version 3.6.1, using the packages ‘CARET’ version 6.085, ‘mgcv’ version 1.8.31, and ‘glmnet’ version 3.0.2. We fit the child models using five-fold cross validation for each combination and selected the best performing, non-correlated child models based on the out-of-sample predictive performance (final covariates for each pathogen–drug combination are shown in table S8). We then calculated the R^2-weighted mean of the estimates of the child models, constraining the coefficients to sum to one, and used these ensemble estimates to fit a spatiotemporal Gaussian process regression (ST-GPR) model for each pathogen–drug combination.

ST-GPR is described in detail elsewhere.[1,2] In brief, spatial and temporal weights were applied to the residuals of the stacked ensemble model; these were then added to the modelled estimates to smooth them in time and space. A Gaussian process regression (GPR) was then fit, and the mean prevalence of AMR was calculated from 1000 draws of the GPR for each location and year with endemic disease. The 1000 draws of the model were taken through to the next stage of calculations to propagate uncertainty throughout.

Section 3.5.4: Resistance profiles

To accurately assess the burden associated with resistance to each antibiotic, we needed to first understand the landscape of multidrug-resistant bacteria, for which the burden would be shared across several antibiotics. We therefore estimated, for each bacteria studied, a set of ‘resistance profiles’ characterised as the probabilities for each possible combination of resistance/susceptibility for all of the antibiotics analyzed. For example, for a bacterium for which we assessed three antibiotics, we would estimate eight probabilities: SSS, SSR, SRS, RSS, SRR, RSR, RRS, and RRR (S – susceptible, R – resistant). These probabilities encompass the entire set of possibilities of resistance for the bacterium, and sum to 1.

For a pathogen for which we assessed *n* antibiotics, resistance profiles were estimated by optimising over a $2^n - 1$-dimensional probability simplex with $\frac{n(n+1)}{2}$ linear constraints. Every such set of resistance profiles corresponds to a full specification of a multivariate binomial distribution. The target set of constraints were as follows:

- The inferred marginal probability of resistance for each antibiotic (the prevalence of resistance to an antibiotic irrespective of all others analyzed) exactly matches the estimates from our prevalence of resistance models. Since there are *n* antibiotics, this set comprises *n* constraints.
- The inferred pairwise likelihood of co-resistance for each pair of antibiotics exactly matches the likelihood inferred from the marginal probability of each antibiotic in the pair, and the Pearson correlation of resistance between the two antibiotics observed across all of the laboratory data we compiled. These represent $\frac{n^2-n}{2}$ additional constraints.

The input format for these constraints with an example case can be found in the appendix of Murray et al. (2022).[1] However, there is no *a priori* guarantee that the observables generate a feasible solution. To prevent the constraints from delineating an infeasible probability simplex (for example, an input suggesting the individual resistances to antibiotics A and B are both above 90% but the probability of co-resistance to A and B is below 10%), we solved an optimization problem that identified, for each input matrix, the closest feasible set of input constraints and a corresponding set of resistance profiles that fits these constraints. The 1-simplex in any dimension is specified by

$$\Delta \coloneqq \{p: \quad 0 \le p_i \le 1,\ \textstyle\sum p_i = 1\} \qquad (3.5.5.1)$$

Each marginal observation and each pairwise co-resistance corresponds to a linear constraint, where a sum over a subset of the p in the simplex should be a given value v_i:

$$\mathrm{m}_\mathrm{i}^\mathrm{T} p = v_i \qquad (3.5.5.2)$$

where m_i is a 'mask vector' of zeros and ones, used to pick out the appropriate summands. Overall, there are $\frac{n(n+1)}{2}$ such affine constraints. The optimisation problem we solve is to find the nearest feasible simplex given these constraints:

$$\min_{p \in \Delta} f(p) \coloneqq \sum_{i=1}^{n(n+1)/2} \frac{1}{\sigma_i^2} (m_i^T p - v_i)\hat{}2 \qquad (3.5.5.3)$$

Where $\frac{1}{\sigma_i^2}$ can be used to provide importance weights for the data. This is a least squares problem with linear equality and inequality constraints (corresponding to the simplex), and can be solved very efficiently even for relatively large n (such as 10 co-occurring antibiotic classes). The result is guaranteed to return the probability simplex closest to the specified constraint, even if the original set of constraints is infeasible, and corresponding set of resistance profiles that fits this nearest simplex.

To propagate uncertainty, we repeat this procedure for each of the 1,000 draws we estimate for prevalence of antibiotic resistance. To generate the *i*-th draw of our resistance profiles, we input the *i*-th draw of the marginal probability of resistance for each antibiotic analyzed for a given pathogen into the probability simplex optimization algorithm. Updating the marginal probabilities of resistance in turn influences the probabilities of co-resistance, and each element of the input we feed the algorithm is unique to the *i*-th draw. The optimization is also initialised randomly for every draw. This process is implemented for each country, resulting in 1000 resistance profiles for each country for each pathogen in our analysis. The Pearson correlations of co-resistance that we derive from the input data are assumed to be constant across location, sex, and infectious syndrome.

Section 3.5.5: Model validation

Validation of prevalence of resistance modelling occurs in two instances. For the ensemble estimates, machine-learning candidate models are validated using five random holdout sets, and we select models correlated below a Pearson correlation coefficient threshold of 0.8 which showed the best performance based on the R^2 predictive validity for the out-of-sample predictions. These intermediary results are not reported in this paper because they do not pertain to the final prevalence of resistance estimate.

We then validate the entire ensemble ST-GPR process by calculating in-sample and out-of-sample accuracy metrics. Accuracy is measured as the proportion of correctly classified resistant/susceptible isolates based on the modelled estimate and the raw data's prevalence of resistance. As a written example, if there were 10 isolates with 50% resistance in the raw data and the model predicted 60% resistance for that location, we would have 5 correctly classified resistant samples (true positives), 1 incorrectly classified resistant sample (false positive), and 4 correctly classified susceptible samples (true negatives), for 90% accuracy. For out-of-sample cross-validation, we withheld, at the outset of the ensemble modelling process, a set of countries with data as a holdout group: for the core-combinations we withheld 20% of countries each iteration, for 5 total holdout sets, while for the supplementary-combinations we withheld 10% of countries each iteration, for 10 holdout sets. By holding out all of the data for a set of countries, our out-of-sample accuracy metrics reflect the potential model fit we have for countries that have no input data in the entire prevalence of resistance process. The detailed reports on the accuracy metric for each pathogen–drug combination can be found in the appendix of Murray et al. (2022).[1]

Section 3.6: Relative risk

Section 3.6.1: Input data and data processing

The input data for the relative risk estimation step included literature data that provided relative risk of death for resistant and susceptible organisms and hospital-based microbiology surveillance data linked to outcomes, as well as other clinical parameters (eg, demographics, diagnoses). Published studies were identified from a recent meta-analysis performed by Cassini and colleagues.[18]

The data inputs for the excess duration estimates were literature data that reported on length of stay for resistant and susceptible organisms and hospital-based microbiology surveillance data that were linked to outcomes as well as various other clinical parameters (eg, demographics, diagnoses). The number of days between a positive specimen

date and discharge date was used to obtain the mean duration of infection. We took into account days elapsed between admission and discharge as mean duration of stay if this was the only piece of information provided in the study. We also considered median duration of infection or median duration of stay if the study only provided this piece of information.

Relative risk estimates were extracted from primary literature as were study characteristics that described the adjustments made by the study. When no adjustments were made, or an adjusted odds ratio was presented, we extracted the crude relative risk. For hospital data that contained admission diagnoses, diagnoses were mapped to GBD Level 2 causes. Admission diagnoses were mapped to GBD causes using ICD codes when provided; when admission diagnoses were free-text entries, they were mapped using two expert reviews.

Section 3.6.2: Modelling overview

The measure of excess risk used to estimate the fatal burden of AMR was the relative risk of death from an infection with a pathogen resistant to the antibiotic of interest as compared to an infection of the same site with the same organism that was susceptible to the antibiotic of interest. The relative risk estimate was produced after adjusting for various potential confounders including age, admission diagnosis (mapped to GBD causes), site of culture, and hospital versus community onset. Because of data sparsity, a single measure of relative risk was estimated for each pathogen–drug combination, representing a global estimate for all sites of infection and all underlying causes.

When data availability allowed it, relative risk from hospital-based microbiology surveillance data was estimated after adjusting for age, admission diagnosis, site of culture, and hospital- versus community-acquired infection, otherwise a crude relative risk was used. The adjusted estimates of relative risks were then included with the crude relative risks in a two-stage nested mixed effects meta-regression model using MR-BRT. The stage one model was a meta-regression for each antibiotic class, which was used to produce a prior for the stage two model. We considered study-specific adjustments such as age of patients, admission diagnosis, site of culture and hospital-versus community acquired infection as potential covariates to be included in the second stage. Covariate selection was based on a set of log-linear models with a range of Lasso penalty parameters, and only statistically significant covariates were selected. The stage two model was run for each antibiotic class with a random effect for pathogen and fixed effects for study level characteristics that described whether the relative risk estimate from a study or dataset adjusted for each parameter using the prior from the stage one model for the antibiotic class.

$$Relative\ Risk_{pathogen_n drug_d} = \beta_0 + \beta_d \cdot x + u_{pathogen_n} + \epsilon_d \qquad (3.6.2.1)$$

Where x is a bias covariate, $u_{pathogen_n}$ is a random effect for pathogen n within an antibiotic class, ϵ_K is the measurement error, d is antibiotic class and β and X are vectors of length $i + 1$ for i covariates. From this stage two model, we produced 1000 draws to estimate the relative risk of death and uncertainty attributable to resistance for each pathogen–drug combination.

For non-fatal burden estimation, we estimated the excess duration attributable to resistance – comparing the length of hospital stay for an infection with a pathogen resistant to the antibiotic of interest to an infection of the same site with the same organism that was susceptible to the antibiotic of interest. For community-acquired infections the entire duration of length of stay was attributed to the infection, whereas for hospital-acquired infections we used the time from first positive culture to time of discharge to estimate length of stay. To address the potential confounding effect of longer admissions resulting in higher probability of acquiring resistant infections, we adjusted the relative length of stay obtained from patient level data for the number of hospital days prior to culture positivity. We observed a generally lower relative length of stay when we applied this adjustment, which was expected. We then used the same two-stage nested mixed effects meta-regression modelling framework described for fatal estimation to produce a relative length of stay attributable to resistance for each pathogen–drug combination. One exception to this estimation process was *Neisseria gonorrhoeae*, which had too little data to produce an estimate on the impact of resistance on duration of illness. As a result, we produced a YLD estimate based on the excess duration of illness for a given antibiotic class.

The analysis of relative risk followed the definitions of the prevalence of resistance step (section 3.5) as closely as possible. Both analyses identified resistance to a given antibiotics class if the isolate had an intermediate or resistant interpretation to any one of the antibiotics in that given class. But the analysis of relative risk diverged from the analysis of prevalence of resistance in the following circumstances. First, the relative risk step included molecular resistance testing if this was the only data provided by a study, eg, β-lactamase or *mecA* positive pathogens; this could potentially misclassify some resistant organisms as sensitive if they had an alternate mechanism for resistance, such as a porin alteration leading to carbapenem resistance. Second, the relative risk estimate produced was for sterile sites of infection, as there was limited data from non-sterile sites. Third, it was not possible to assess relative risk of multidrug-resistant pathogens because of limited data availability and because it did not fit in the modelling strategy at the antibiotic class level. Instead, the relative risk of each of the components of multidrug-resistant pathogens was calculated and the antibiotic class with the highest relative risk was used; for *Salmonella* Typhi this was relative risk to Trimethoprim-Suflamethoxazole. Fourth, we had limited availability of data on fatalities attributable to *Salmonella* Paratyphi and *Shigella* species; as a result, we used fatal relative risk estimates from *Salmonella* Typhi as a proxy. Fifth, there were limited data on fatalities attributable to resistant *N. gonorrhoeae,* so we excluded the fatal estimate for this pathogen. Finally, the relative risk of *Mycobacterium tuberculosis* was assessed for multidrug and extensively drug-resistant infections as reported previously in GBD. Estimates of relative risk of death for sterile sources of specimen across 88 pathogen–drug combinations can be viewed in the appendix of Murray et al. (2022).[1]

Section 3.6.3: Model validation

We report three summary metrics to evaluate the relative risk of death models: the root-mean squared error (RMSE), the Mean Average Error (MAE) and the percent coverage of observed data within the full variance of the model. These three metrics were calculated using the real relative risk ratio in the whole sample of data and also by holding out 25% of the sample within antibiotic class in 4 iterations. The details on in-sample and out-of-sample performance metrics for relative risk of death models can be seen in the appendix of Murray et al. (2022).[1]

This approach for relative risk estimation had several limitations, most were attributable to data sparsity. First, it is likely that the impact of resistance on mortality is different across locations. In locations where overall health-care access and quality are lower, the impact of resistance may be smaller because the management of susceptible infections is sub-optimal. Conversely, in locations where broad, second- and third-line antimicrobials are not available, one would expect the impact of resistance to be greater. Second, it is possible that the relative risk of death attributable to resistance is different across anatomical sites of infection because of variable penetrance of antibiotics to different anatomical locations. As we continue efforts to expand data collection and reporting, we hope to be able to address these limitations in future iterations.

Section 3.7: Counterfactuals and AMR estimation

Section 3.7.1: Estimating AMR burden with counterfactual of no infection

We computed two counterfactuals to estimate the drug-resistant burden. First, we estimated the burden of AMR using the counterfactual of no infection. We estimated the fatal burden of individual pathogen–drug combinations by taking the product of the deaths for each underlying cause, fraction of deaths related to infection, infectious syndrome fraction, fatal pathogen fraction, and fatal prevalence of resistance and then summed across all infectious syndromes and underlying causes:

$$Deaths\ with\ Resistance_{Kd} = \sum_J \sum_L D_J \times S_J \times M_{LJ} \times P_{LK} \times R_{Kd} \quad (3.7.1.1)$$

where D = deaths, S = fraction related to infection, M = infectious syndrome fraction, P = fatal pathogen fraction, R = fatal prevalence of resistance, J = cause, L = syndrome, K = pathogen, d = drug. To produce an estimate of deaths with resistance to any antibiotic estimated, we employed the same formula but used the fatal prevalence of resistance to any antibiotic using the resistance profiles, described previously. We calculated the fatal prevalence of resistance R for a given drug d based on the non-fatal prevalence of resistance R' and relative risk of death RR for this drug:

$$R_{Kd} = \frac{R'_{Kd} RR_{Kd}}{(1 - R'_{kd}) + R'_{Kd} RR_{Kd}} \quad (3.7.1.2)$$

We calculated the fatal prevalence of resistance to any antibiotic estimated based on the non-fatal prevalences of each resistance profile, incorporating all resistance profiles δ that are resistant to at least 1 drug with corresponding relative risks RR_{Kd^*}, determined by the method described below (section 3.7.2):

$$R_{K,all\ drugs} = \frac{\sum_\delta R'_{K\delta} RR_{Kd^*}}{\left(1 - \sum_\delta R'_{K\delta}\right) + \sum_\delta R'_{K\delta} RR_{Kd^*}} \quad (3.7.1.3)$$

We then estimated YLLs using standard GBD methods to convert age-sex specific deaths into YLLs.[3]

For the non-fatal estimate, we first estimated the incidence of each infectious syndrome in each underlying cause. For infectious underlying causes, we simply used the incidence estimated in GBD. For non-infectious underlying causes, we divided the infectious syndrome deaths ($D_J \times S_J \times M_{LJ}$) by the syndrome- and pathogen-specific CFRs calculated in section 5, aggregated across pathogen using the nonfatal pathogen distribution P' calculated above.

$$Incidence_{JL} = \frac{D_J S_J M_{LJ}}{\sum_K CFR_{LK} P'_{LK}} \quad (3.7.1.4)$$

We then took the product of the infectious syndrome incidence, the non-fatal pathogen fraction, and the non-fatal prevalence of resistance and summed across all infectious syndromes and underlying causes to get incidence with resistance for every pathogen and drug. As with the fatal estimate, to produce an estimate of incident infections with resistance to any antibiotic, we used the same formula and used the non-fatal prevalence of resistance to any antibiotic estimated from the resistance profiles.

We then calculated YLDs for each pathogen. For some GBD causes, we simply used the GBD YLD estimates and multiplied them by the corresponding nonfatal pathogen distribution (table 8.1.2) For all other causes, we multiplied together the infectious syndrome incidence, the non-fatal pathogen fraction, and a syndrome-specific YLDs per incident case rate, calculated using a proxy cause from GBD.[3] To estimate the YLDs per incident case rate, we extracted GBD incidence and YLD estimates for the proxy causes and divided the YLDs by the incidence for each age, sex, and location. Three infectious syndromes are not estimated in the GBD, and therefore have no standard sequelae or disability weights: bloodstream infections, intra-abdominal infections, and bone and joint infections. For the proxy causes for these three syndromes, we used the closest approximate disease as determined by a group of experts in infectious diseases and epidemiology. This approach is a significant limitation of the study and should be improved in future work.

To get the YLDs associated with resistance for each pathogen, we used the non-fatal prevalences of resistance for each drug and resistance profile and relative length of stay (LOS) for each pathogen–drug combination to calculate the fraction of YLDs associated with resistance for each pathogen, using equations analogous to equations 3.7.1.2 and 3.7.1.3. We multiplied this fraction by the YLDs for each pathogen to get YLDs associated with resistance to each pathogen–drug combination and YLDs associated with resistance any antibiotics estimated. We then added YLLs and YLDs to produce the DALY estimate for burden associated with resistance.

Section 3.7.2: Estimating AMR burden with counterfactual of infection with susceptible organism

For the second counterfactual – comparing resistant to susceptible infections – we calculated mutually exclusive pathogen–drug estimates. To do this, we first estimated the population attributable fraction of deaths ($Mortality\ PAF$) for each resistance profile with resistance to at least 1 drug, δ. The inputs for the PAF were the non-fatal prevalence of the given resistance profile, $R'_{K\delta}$, and the relative risk of death for resistant infection compared to susceptible infection for each drug, RR_{Kd}. Because of data sparsity, we were unable to calculate the relative risk for every possible resistance profile, and so instead used the highest relative risk of all of the drugs in the resistance profile. For example, if for a resistance profile of resistant to penicillin and fluoroquinolones, the relative risk was 1.1 for penicillin and 1.4 for fluoroquinolones, we would use a relative risk of 1.4 for this profile. The mortality PAF is calculated as a multi-category exposure:

$$Mortality\ PAF_{K\delta} = \frac{R'_{K\delta}(RR_{Kd^*} - 1)}{1 + \sum_{\delta} R'_{K\delta}(RR_{Kd^*} - 1)} \qquad (3.7.2.1)$$

where d* is the drug in the resistance profile δ with the highest relative risk.

We then took the product of the deaths for each underlying cause, fraction of deaths related to infection, infectious syndrome fraction, fatal pathogen fraction, and the mortality PAF for each resistance profile to get the deaths attributable to resistance for every resistance profile:

$$Deaths\ due\ to\ Resistance_{K\delta} = \sum_{J}\sum_{L} D_J \times S_J \times M_{LJ} \times P_{LK} \times Mortality\ PAF_{K\delta} \qquad (3.7.2.2)$$

When the resistance profile described resistance to more than one antibiotic, the deaths were then distributed to the component pathogen–drug combinations based on the excess risk of the pathogen–drug combination divided by the sum of the excess risk of all pathogen–drug combinations in the resistance profile. For a resistance profile δ with resistance to drugs $i = 1, \ldots, n$:

$$Redistribution\ Weight_{Kd_i} = \frac{RR_{Kd_i} - 1}{\sum_{\hat{i}}(RR_{Kd_{\hat{i}}} - 1)} \qquad (3.7.2.3)$$

For co-resistance amongst beta-lactam antibiotics (ie, carbapenems, 4GC, 3GC, antipseudomonal, BL/BLI, aminopenicillins, and penicillin), we used a different approach to redistributing burden. Similar to Cassini et al., we applied a hierarchy such that the burden was categorically attributed to the broadest beta-lactam antibiotic, rather than split the burden between multiple beta-lactam antibiotics.[4] When a pathogen was resistant to multiple beta-lactams and a non-beta-lactam antibiotic, we first applied the hierarchy to determine the 'highest' beta-lactam resistance and then generated redistribution weights using only the 'highest' beta-lactam and the non-beta-lactams. We then used these attributable death estimates to estimate YLLs using standard GBD methods to convert age-sex specific deaths to YLLs.

A similar approach was taken to estimate non-fatal burden for the counterfactual of antibiotic-susceptible infection. We first assumed that antibiotic resistance has no effect on the attack rate of pathogens; therefore, there are 0 incident cases attributable to resistance and all non-fatal burden comes from increased length of illness. To quantify the extent of this increased length of illness, we first produced a length of stay (LOS) PAF for each resistance profile using the non-fatal prevalence of resistance and relative LOS for resistant infections as compared to susceptible infections in a method analogous to equation 3.7.2.1. Because of data sparsity, we were unable to calculate the relative LOS for every resistance profile, and so instead used the relative LOS for the drug with the highest relative LOS in the profile. We then took the product of the YLDs for each infectious syndrome, the non-fatal pathogen distribution, and the LOS PAF to produce attributable YLD estimates. This assumes that the attributable LOS PAF is equally applicable to all sequelae, which is an assumption made because of a lack of data on the impact of resistance on the likelihood of different sequelae and the duration of specific sequelae. Specifically for AMR, this assumption fails to account for the fact that patients with resistant infections are more prone to re-infection, treatment failure and long term sequelae as compared to patients with susceptible ones, and we acknowledge this is a significant limitation that should be improved in future work. We then added YLLs and YLDs to produce an estimate of DALYs attributable to resistance.

Because of the optimisation approach used to derive each resistance profile, the prevalence of resistance to for a given pathogen–drug as modelled using ensemble ST-GPR (section 3.5.3), R'_{Kd}, will not necessarily be exactly equal to the sum of all resistance profiles $R'_{K\delta}$ that include resistance to drug d. Due to this inconsistency, in extremely rare cases, an estimate of AMR burden in the susceptible counterfactual may slightly exceed the corresponding estimate of AMR burden in the no infection counterfactual for a specific pathogen–drug. We consider the ensemble ST-GPR estimate to be more accurate than the resistance profiles, since the latter are based on Pearson correlations of multidrug resistance that are calculated from limited microdata and generalised to all locations. For this reason, we cap all individual pathogen–drug estimates of burden for the susceptible counterfactual, which are

based on the resistance profiles, to the burden for the no infection counterfactual, which are based on the ensemble ST-GPR estimates.

Section 3.7.3: Excluded combinations

Although our approach attempted to be exhaustive and include all clinically-relevant pathogen–drug combinations, there are two combinations included in the WHO priority list for which we could not produce an estimate. The first is clarithromycin resistance in *Helicobacter pylori* and the second is fluoroquinolone resistance in *Campylobacter* species. These were excluded due to limited data availability, as highlighted by a recent study in the European Union that found that, as of 2019, no member countries had implemented publicly accessible, mandatory reporting surveillance programmes for these two pathogen–drug combinations.[19] *H. pylori* and *Campylobacter* spp. are commonly diagnosed without culture so resistance profiles are uncommon in passive surveillance systems. The burden of *H. pylori* is not currently estimated in GBD, though some of the consequent diseases are, like peptic ulcer disease and gastric cancer. Producing a burden estimate of *H. pylori was* outside the scope of this work, and without a pathogen burden estimate, we could not produce an estimate of the burden attributable to clarithromycin-resistant *H. pylori*. In contrast, GBD does produce an estimate on the burden of *Campylobacter* spp. There were, however, too few data to produce an estimate on the excess risk of death or duration associated with fluoroquinolone resistance and limited data to inform a global prevalence of resistance estimate. Given these limitations, we did not produce burden estimates for clarithromycin-resistant *H. pylori* or fluoroquinolone-resistant *Campylobacter* spp. Because of the lack of data on risk of death associated with drug-resistant *Neisseria gonorrhoeae,* we were unable to produce an estimate of the fatal burden of resistance so produce only a non-fatal estimate. Many potential pathogen–drug combinations were excluded due to the spectrum of antimicrobial activity (ie, vancomycin and *E. coli*), intrinsic resistance (eg, BL/BLI resistance in *Pseudomonas aeruginosa*) or resistance that is exceedingly common (eg, penicillin resistance in *S. aureus*); these combinations were decided by a group of experts in infectious diseases, microbiology, epidemiology, and population health. A final constraint was the computational burden of estimating more than seven antibiotic classes for a single pathogen. Because of the approach to co-resistance described in section 3.5, each antibiotic class added led to an exponential increase in the computation needs and anything above seven antibiotic classes was not tenable. As additional data are made available, we plan to add clinically relevant combinations and iterate on the computational approach so that we can describe the burden of bacterial AMR more comprehensively.

Section 3.8: Special considerations

Section 3.8.1: The use of defined daily doses (DDDs) and breakpoint interpretations

Although used pervasively, the DDD metric is not ideal and is often misunderstood, which is why novel approaches to quantify drug utilisation have been proposed recently, especially for the paediatric population.[20,21] As DDD aims to capture a dosing regimen intended for a 70-kg adult patient, concentrating on the frequency and the duration of a single-unit dose, this is not always an accurate representation of prescribed doses in certain countries[22]. Bruyndonckx et al.[23] demonstrated how the typical content of an original antibiotic package has significantly increased in European countries over time, with substantial differences between countries and antibiotic groups (apart from fluoroquinolones); this alone has important implications for understanding the link between antibiotic usage and resistance development, and consequently on resultant mortality rates. Likewise, inconsistent associations and predictions of resistance can be observed when DDDs are compared with different metrics, such as "packages per 1000 inhabitants per day (PID)".[24] We should also consider mathematical and theoretical models that indicate how consumption-resistance relationships are usually nonlinear,[25] while patient-related determinants of antibiotic use must also be taken into account.[26] These are some of the reasons why we have decided to pursue separate mortality analyses for different antibiotic groups, prompted also by the recent ECDC/EFSA/EMA report (ie, ECDC in collaboration with European Food Safety Authority and European Medicine Agency),[27] and we expect that our research may influence the development of an optimal metric for future estimations.

In addition, from 2019, EUCAST has changed the long-held definitions of antimicrobial susceptibility testing (AST) categories susceptible (S), intermediate (I), and resistant (R) to susceptible with standard dosing regimen, susceptible with increased exposure, and resistant, respectively.[28] This is in contrast with CLSI clinical breakpoints[29] and methodology, which uses the classic trifecta of AST categories, although they are also changing their approach towards 'susceptible - dose dependent' instead of intermediate category for several pathogen-drug combinations.

Section 4: Supplementary Tables and Figures

Supplementary Table 1. Deaths attributable to and associated with antimicrobial resistance (expressed as counts and age-standardised rates (ASMR) per 100 000 with 95% uncertainty intervals) per country in the WHO European Region and specific pathogen–drug combination (note: first row represents attributable mortality and second row associated mortality for every country).

Country	Pathogen	Antibiotic class	Deaths attributable to / associated with AMR (counts)	Deaths attributable to / associated with AMR (ASMR per 100 000)
**Albania**	*Staphylococcus aureus*	Methicillin	49.8 (21.7–88.5)	1.3 (0.6–2.3)
	Escherichia coli	Aminopenicillin	338 (187–552)	8.4 (4.6–13.7)
**Andorra**	*Escherichia coli*	Fluoroquinolones	0.859 (0.497–1.45)	0.6 (0.3–1)
	Escherichia coli	Aminopenicillin	10.4 (6.58–16.3)	7 (4.4–11.1)
**Armenia**	*Escherichia coli*	Third-generation cephalosporins	41.1 (16.3–81.7)	1.1 (0.4–2.1)
	Escherichia coli	Aminopenicillin	431 (288–601)	11.1 (7.4–15.5)
**Austria**	*Enterococcus faecium*	Fluoroquinolones	68.9 (20.1–133)	0.4 (0.1–0.7)
	Escherichia coli	Aminopenicillin	916 (577–1390)	4.8 (3–7.2)
**Azerbaijan**	*Klebsiella pneumoniae*	Third-generation cephalosporins	100 (24.7–215)	1.4 (0.4–3.1)
	Escherichia coli	Aminopenicillin	908 (598–1320)	12.9 (8.5–19)
**Belarus**	*Escherichia coli*	Third-generation cephalosporins	158 (64.1–298)	1 (0.4–1.9)
	Escherichia coli	Beta lactam/Beta-lactamase inhibitors	1130 (678–1790)	7.4 (4.4–11.7)
**Belgium**	*Staphylococcus aureus*	Methicillin	155 (63.8–278)	0.6 (0.2–1.1)
	Escherichia coli	Aminopenicillin	1980 (1360–2800)	7.5(5.1–10.7)
**Bosnia and Herzegovina**	*Staphylococcus aureus*	Methicillin	38.5 (15.6–72.7)	0.7 (0.3–1.3)
	Escherichia coli	Aminopenicillin	352 (203–548)	6.2 (3.6–9.8)
**Bulgaria**	*Escherichia coli*	Third-generation cephalosporins	165 (60.9–347)	1.2 (0.4–2.5)
	Escherichia coli	Aminopenicillin	1820 (1030–2900)	12.9 (7.3–20.6)
**Croatia**	*Staphylococcus aureus*	Methicillin	57.4 (22.3–108)	0.7 (0.3–1.3)
	Escherichia coli	Trimethoprim-sulfamethoxazole	586 (374–861)	6.5 (4.1–9.6)
**Cyprus**	*Staphylococcus aureus*	Methicillin	21.3 (9.22–37.7)	1.3 (0.6–2.2)
	Escherichia coli	Aminopenicillin	169 (115–242)	1 (6.8–14.3)
**Czech Republic**	*Escherichia coli*	Beta lactam/Beta-lactamase inhibitors	86.1 (52.1–132)	0.4 (0.3–0.6)
	Escherichia coli	Aminopenicillin	1100 (690–1620)	5.2 (3.3–7.7)
**Denmark**	*Enterococcus faecium*	Fluoroquinolones	52.8 (14.8–101)	0.5 (0.13–0.9)

	Escherichia coli	Aminopenicillin	751 (489–1100)	6.2 (4–9.1)
**Estonia**	*Enterococcus faecium*<br>*Escherichia coli*	Fluoroquinolones<br>Aminopenicillin	11.3 (3.27–21.9)<br>109 (68.7–170)	0.5 (0.1–0.9)<br>4.1 (2.5–6.4)
**Finland**	*Enterococcus faecium*<br>*Escherichia coli*	Fluoroquinolones<br>Aminopenicillin	49.8 (13.9–95.8)<br>439 (281–654)	0.4 (0.1–0.8)<br>3.4 (2.2–5.1)
**France**	*Staphylococcus aureus*<br>*Escherichia coli*	Methicillin<br>Aminopenicillin	795 (333–1380)<br>9710 (6350–14,100)	0.5 (0.2–0.88)<br>6.1 (4–8.7)
**Georgia**	*Klebsiella pneumoniae*<br>*Klebsiella pneumoniae*	Third-generation cephalosporins<br>Third-generation cephalosporins	57.3 (20.6–112)<br>462 (283–712)	1 (0.4–1.9)<br>7.8 (4.9–12.2)
**Germany**	*Escherichia coli*<br>*Escherichia coli*	Beta lactam/Beta-lactamase inhibitors<br>Aminopenicillin	1040 (661–1560)<br>15,400<br>(10,200–22,400)	0.5 (0.31–0.8)<br>7.4 (4.8–10.8)
**Greece**	*Staphylococcus aureus*<br>*Escherichia coli*	Methicillin<br>Aminopenicillin	370 (165–620)<br>1730 (1110–2570)	1.3 (0.6–2.2)<br>6.2 (3.9–9.3)
**Hungary**	*Staphylococcus aureus*<br>*Escherichia coli*	Methicillin<br>Aminopenicillin	181 (73.7–336)<br>1840 (1100–2850)	1 (0.4–1.8)<br>9.5 (5.6–14.7)
**Iceland**	*Staphylococcus aureus*<br>*Escherichia coli*	Methicillin<br>Aminopenicillin	1.71 (0.726–3)<br>23.9 (15.4–35.3)	0.3 (0.1–0.5)<br>4 (2.5–5.8)
**Ireland**	*Staphylococcus aureus*<br>*Escherichia coli*	Methicillin<br>Aminopenicillin	37.7 (15.2–66.7)<br>537 (361–776)	0.5 (0.2–0.9)<br>7.1 (4.7–10.2)
**Israel**	*Staphylococcus aureus*<br>*Escherichia coli*	Methicillin<br>Aminopenicillin	165 (73.2–279)<br>1090 (751–1540)	1.3 (0.6–2.3)<br>8.7 (6–12.3)
**Italy**	*Staphylococcus aureus*<br>*Escherichia coli*	Methicillin<br>Aminopenicillin	1310 (544–2320)<br>10,100<br>(6330–15,200)	0.8 (0.3–1.5)<br>6.2 (3.9–9.4)
**Kazakhstan**	*Staphylococcus aureus*<br>*Escherichia coli*	Methicillin<br>Aminopenicillin	217 (90.8–396)<br>1640 (1070–2440)	1.4 (0.6–2.5)<br>10.3 (6.7–15.5)
**Kyrgyzstan**	*Staphylococcus aureus*<br>*Escherichia coli*	Methicillin<br>Aminopenicillin	75.9 (33.3–137)<br>442 (288–643)	1.6 (0.7–3)<br>9.6 (6.2–14.1)
**Latvia**	*Escherichia coli*<br>*Escherichia coli*	Third-generation cephalosporins<br>Aminopenicillin	23.4 (8.79–47.8)<br>323 (199–501)	0.6 (0.2–1.2)<br>7.8 (4.9–12.3)
**Lithuania**	*Escherichia coli*	Trimethoprim-sulfamethoxazole	34.7 (19.8–57)	0.6 (0.3–1)

	Escherichia coli	Aminopenicillin	468 (287–738)	8.1 (4.9–12.7)
**Luxembourg**	*Enterococcus faecium*<br>*Escherichia coli*	Fluoroquinolones<br>Aminopenicillin	4.48 (1.29–8.48)<br>63.1 (39.8–92.6)	0.4 (0.1–0.8)<br>5.9 (3.7–8.7)
**Malta**	*Staphylococcus aureus*<br>*Escherichia coli*	Methicillin<br>Aminopenicillin	8.66 (3.83–14.7)<br>47.4 (32.2–67.2)	0.9 (0.4–1.6)<br>4.9 (3.3–7)
**Moldova**	*Staphylococcus aureus*<br>*Escherichia coli*	Methicillin<br>Aminopenicillin	91.3 (40.7–159)<br>671 (431–1010)	1.7 (0.8–3)<br>12.4 (8–18.6)
**Monaco**	*Staphylococcus aureus*<br>*Escherichia coli*	Methicillin<br>Aminopenicillin	0.631 (0.259–1.13)<br>7.62 (4.61–11.7)	0.6 (0.20–1.1)<br>7.4 (4.4–11.5)
**Montenegro**	*Escherichia coli*<br>*Escherichia coli*	Third-generation cephalosporins<br>Aminopenicillin	8.56 (3.24–17.1)<br>63.5 (36.7–99.9)	0.9 (0.4–1.9)<br>6.9 (3.9–10.8)
**Netherlands**	*Enterococcus faecium*<br>*Escherichia coli*	Fluoroquinolones<br>Aminopenicillin	168 (49.5–317)<br>2330 (1610–3260)	0.5 (0.1–0.9)<br>6.4 (4.4–9)
**North Macedonia**	*Staphylococcus aureus*<br>*Escherichia coli*	Methicillin<br>Trimethoprim-sulfamethoxazole	42.7 (18.6–79.2)<br>289 (160–458)	1.6 (0.7–2.9)<br>10.5 (5.9–16.7)
**Norway**	*Escherichia coli*<br>*Escherichia coli*	Carbapenems<br>Aminopenicillin	84.7 (49.4–132)<br>485 (328–708)	0.8 (0.5–1.2)<br>4.5 (3–6.6)
**Poland**	*Staphylococcus aureus*<br>*Escherichia coli*	Methicillin<br>Aminopenicillin	481 (198–851)<br>5630 (3480–8790)	0.7 (0.3–1.2)<br>8.1 (5–12.7)
**Portugal**	*Staphylococcus aureus*<br>*Escherichia coli*	Methicillin<br>Aminopenicillin	433 (193–713)<br>2170 (1510–3030)	1.6 (0.7–2.6)<br>8 (5.5–11.2)
**Romania**	*Staphylococcus aureus*<br>*Escherichia coli*	Methicillin<br>Aminopenicillin	652 (303–1180)<br>3810 (2300–6070)	1.9 (0.9–3.4)<br>10.5 (6.3–16.5)
**Russia**	*Escherichia coli*<br>*Escherichia coli*	Third-generation cephalosporins<br>Aminopenicillin	3240 (1380–6260)<br>29,200 (18,500–43,600)	1.5 (0.6–2.8)<br>13.1 (8.3–19.6)
**San Marino**	*Staphylococcus aureus*<br>*Escherichia coli*	Methicillin<br>Aminopenicillin	0.531 (0.205–1)<br>4.2 (2.28–6.86)	0.8 (0.3–1.5)<br>5.8 (3.1–9.6)
**Serbia**	*Staphylococcus aureus*<br>*Escherichia coli*	Methicillin<br>Aminopenicillin	160 (62–303)<br>1560 (914–2430)	1.1 (0.4–2.1)<br>10.2 (5.9–16.1)
**Slovakia**	*Staphylococcus aureus*<br>*Escherichia coli*	Methicillin<br>Aminopenicillin	102 (43.7–182)<br>797 (478–1220)	1.2 (0.5–2.1)<br>9 (5.4–13.9)

**Slovenia**	*Staphylococcus aureus*<br>*Escherichia coli*	Methicillin<br>Aminopenicillin	22.9 (9.36–41.5)<br>256 (152–406)	0.5 (0.2–0.9)<br>5.6 (3.7–8.9)
**Spain**	*Staphylococcus aureus*<br>*Escherichia coli*	Methicillin<br>Aminopenicillin	882 (375–1560)<br>8440 (5890–11,800)	0.8 (0.3–1.4)<br>7.4 (5.1–10.5)
**Sweden**	*Enterococcus faecium*<br>*Escherichia coli*	Fluoroquinolones<br>Aminopenicillin	75.8 (21.6–146)<br>758 (494–1140)	0.4 (0.1–0.7)<br>3.2 (2.1–4.8)
**Switzerland**	*Enterococcus faecium*<br>*Escherichia coli*	Fluoroquinolones<br>Aminopenicillin	65.2 (18.9–124)<br>849 (563–1210)	0.4 (0.1–0.7)<br>4.2 (2.8–6.1)
**Tajikistan**	*Streptococcus pneumoniae*<br>*Streptococcus pneumoniae*	Carbapenems<br>Trimethoprim-sulfamethoxazole	107 (47.7–189)<br>780 (567–1090)	1.3 (0.6–2.3)<br>9.6 (7.1–13)
**Turkey**	*Staphylococcus aureus*<br>*Staphylococcus aureus*	Methicillin<br>Methicillin	2050 (942–3470)<br>7020<br>(4750–10,200)	2.6 (1.2–4.3)<br>8.8 (5.6–12.8)
**Turkmenistan**	*Mycobacterium tuberculosis*<br>*Escherichia coli*	Multi-drug resistance (excluding extensive drug resistance)<br>Aminopenicillin	57.8 (6.32–143)<br>482 (314–704)	1.2 (0.5–2.2)<br>11.9 (7.7–17.6)
**Ukraine**	*Mycobacterium tuberculosis*<br>*Escherichia coli*	Multi-drug resistance (excluding extensive drug resistance)<br>Aminopenicillin	814 (112–1600)<br>6070 (3560–9210)	0.8 (0.3–1.6)<br>8.7 (5.1–13.2)
**UK**	*Staphylococcus aureus*<br>*Escherichia coli*	Methicillin<br>Aminopenicillin	942 (401–1620)<br>11,300<br>(7910–15,900)	0.7 (0.3–1.2)<br>8.3 (5.7–11.6)
**Uzbekistan**	*Mycobacterium tuberculosis*<br>*Escherichia coli*	Multi-drug resistance (excluding extensive drug resistance)<br>Aminopenicillin	423 (53–941)<br>2770 (1860–3990)	1.7 (0.4–3.6)<br>14.7 (9.8–22)

Supplementary Table 2. DALYs attributable to and associated with antimicrobial resistance (expressed as counts and age-standardised rates (ASMR) per 100 000 with 95% uncertainty intervals) per country in the WHO European Region and specific pathogen–drug combination (note: first row represents attributable mortality and second row associated mortality for every country).

Country	Pathogen	Antibiotic class	Deaths attributable to / associated with AMR (counts)	Deaths attributable to / associated with AMR (ASMR per 100 000)
**Albania**	*Staphylococcus aureus*	Methicillin	1093 (479.8–1958.4)	34.9 (15.3–62.1)
	Escherichia coli	Aminopenicillin	6845.6 (3841.1–11047.9)	193.8 (110.3–312.4)
**Andorra**	*Escherichia coli*	Fluoroquinolones	15.2 (8.5–26.1)	11.6 (6.6–20.1)
	Escherichia coli	Aminopenicillin	185.1 (112.1–296.5)	142 (86.9–228.1)
**Armenia**	*Staphylococcus aureus*	Methicillin	900 (367.9–1629.4)	27.3 (11.3–48.4)
	Escherichia coli	Aminopenicillin	9103 (6186.5–12530.5)	253.5 (176.5–348)
**Austria**	*Enterococcus faecium*	Fluoroquinolones	1342.9 (392.2–2591)	8.9 (2.6–17.4)
	Escherichia coli	Aminopenicillin	15404.4 (9707.8–23069.2)	98.1 (61.3–147.1)
**Azerbaijan**	*Klebsiella pneumoniae*	Third-generation cephalosporins	3714.7 (940.8–7668.8)	46.5 (11.7–95.8)
	Streptococcus pneumoniae	Trimethoprim-sulfamethoxazole	30403.2 (21621–41785.5)	394.4 (280.1–545.5)
**Belarus**	*Escherichia coli*	Third-generation cephalosporins	3577.5 (1458.2–6763.5)	26 (10.8–49.1)
	Escherichia coli	Beta lactam/Beta-lactamase inhibitors	25651.3 (15422.9–40591.5)	186.7 (112.4–295.5)
**Belgium**	*Staphylococcus aureus*	Methicillin	2230.1 (907.9–4002)	10.6 (4.2–19.3)
	Escherichia coli	Aminopenicillin	29500.4 (19856.9–42137.5)	140.1 (92.1–202.2)
**Bosnia and Herzegovina**	*Staphylococcus aureus*	Methicillin	786.8 (315.8–1486)	15.4 (6.1–29.5)
	Escherichia coli	Aminopenicillin	6813.7 (3914.5–10656.6)	130.8 (75.9–205.1)
**Bulgaria**	*Escherichia coli*	Third-generation cephalosporins	3242.3 (1217.2–6777.7)	27.1 (10.3–56.5)
	Escherichia coli	Aminopenicillin	35897 (20173.3–56876.8)	300.2 (170.7–474.2)
**Croatia**	*Staphylococcus aureus*	Methicillin	1091.6 (421.2–2046.2)	14.7 (5.7–27.9)
	Escherichia coli	Trimethoprim-sulfamethoxazole	10273.2 (6329.6–15391.3)	131.7 (80.2–201.6)
**Cyprus**	*Staphylococcus aureus*	Methicillin	376.3 (159.4–690.2)	21.8 (9.3–40.2)
	Escherichia coli	Aminopenicillin	2758.1 (1812.2–4111.2)	157.1 (103.6–234.4)
**Czech Republic**	*Enterococcus faecium*	Fluoroquinolones	1782.7 (515.3–3509.7)	10 (2.9–19.6)
	Escherichia coli	Aminopenicillin	20538.5 (12813.5–30495.6)	111.1 (69.3–165.9)

**Denmark**	*Enterococcus faecium*<br>*Escherichia coli*	Fluoroquinolones<br>Aminopenicillin	1009.6 (283.2–1932.8)<br>12189.4 (7717.6–18265.9)	10.4 (2.9–20)<br>117.9 (73.8–178.4)
**Estonia**	*Enterococcus faecium*<br>*Escherichia coli*	Fluoroquinolones<br>Aminopenicillin	244.8 (70.5–474.2)<br>2138.7 (1332.2–3322.2)	11.6 (3.3–22.8)<br>98.9 (60.9–154.9)
**Finland**	*Enterococcus faecium*<br>*Escherichia coli*	Fluoroquinolones<br>Aminopenicillin	973.9 (277.6–1879.7)<br>7425 (4706.6–11065.3)	10.2 (2.9–19.8)<br>72.7 (45.5–108.7)
**France**	*Staphylococcus aureus*<br>*Escherichia coli*	Methicillin<br>Aminopenicillin	11754.2 (4952.1–21018.5)<br>147947.4 (96705.2–215729)	10 (4.1–18.3)<br>124 (79.6–182.9)
**Georgia**	*Klebsiella pneumoniae*<br>*Klebsiella pneumoniae*	Third-generation cephalosporins<br>Third-generation cephalosporins	1291.7 (470.1–2508.8)<br>10425.2 (6448.9–15727.3)	27.8 (10.1–53.2)<br>224.4 (140.3–335.2)
**Germany**	*Escherichia coli*<br>*Escherichia coli*	Beta lactam/Beta-lactamase inhibitors<br>Aminopenicillin	16599.3 (10449.9–24948.7)<br>246465.8 (160813.6–360058.8)	9.9 (6.1–15.1)<br>147.1 (94.7–217.9)
**Greece**	*Staphylococcus aureus*<br>*Escherichia coli*	Methicillin<br>Aminopenicillin	5198.3 (2241.4–8945)<br>25599.2 (15917.5–38180.7)	24.8 (10.5–44)<br>122.3 (74.8–185.2)
**Hungary**	*Staphylococcus aureus*<br>*Escherichia coli*	Methicillin<br>Aminopenicillin	3707.5 (1482.5–7015.2)<br>35804.1 (20981.4–55240.1)	23.3 (9.3–44.5)<br>214.7 (124.7–329.6)
**Iceland**	*Staphylococcus aureus*<br>*Escherichia coli*	Methicillin<br>Aminopenicillin	31.3 (8.6–61.8)<br>400.3 (252.3–591.7)	6.4 (1.8–12.8)<br>79.3 (49.4–118.6)
**Ireland**	*Escherichia coli*<br>*Escherichia coli*	Beta lactam/Beta-lactamase inhibitors<br>Aminopenicillin	598.8 (376.5–901.6)<br>9086.6 (5921–13408.3)	8.6 (5.4–13.1)<br>131 (84.3–194.5)
**Israel**	*Staphylococcus aureus*<br>*Escherichia coli*	Methicillin<br>Aminopenicillin	2752.9 (1192–4748.3)<br>17416.7 (11710.3–24898.8)	24.7 (10.5–43.1)<br>153.2 (101.9–220.3)
**Italy**	*Staphylococcus aureus*<br>*Escherichia coli*	Methicillin<br>Aminopenicillin	20332.9 (8312–36561.1)<br>154014.4 (96526.1–230146.5)	17.4 (7–31.9)<br>125.3 (78.2–188.2)
**Kazakhstan**	*Staphylococcus aureus*<br>*Escherichia coli*	Methicillin<br>Aminopenicillin	6573.2 (2724.7–11811.5)<br>48209.1 (31686.6–72282.4)	36.9 (15.3–66.3)<br>270.8 (177.3–404)
**Kyrgyzstan**	*Staphylococcus aureus*<br>*Escherichia coli*	Methicillin<br>Aminopenicillin	2997.1 (1357.6–5261.2)	51.2 (22.9–90.8)<br>282.5 (186.1–403.5)

			15890.2 (10588.1–22504.5)	
**Latvia**	*Escherichia coli*<br>*Escherichia coli*	Third-generation cephalosporins<br>Aminopenicillin	443 (171.5–908.5)<br>6141.6 (3808.8–9414.2)	13.5 (5.2–27.7)<br>186.8 (115.2–289.5)
**Lithuania**	*Escherichia coli*<br>*Escherichia coli*	Trimethoprim-sulfamethoxazole<br>Aminopenicillin	672.9 (380.9–1107.5)<br>9159.1 (5653.8–14406.4)	14.4 (8.2–23.6)<br>196.4 (121–309.4)
**Luxembourg**	*Enterococcus faecium*<br>*Escherichia coli*	Fluoroquinolones<br>Aminopenicillin	88.4 (25.3–169.2)<br>1090.6 (690.2–1609.7)	9.7 (2.8–18.6)<br>117.3 (74–175.2)
**Malta**	*Staphylococcus aureus*<br>*Escherichia coli*	Methicillin<br>Aminopenicillin	138.8 (59.3–243.3)<br>760.8 (506.7–1106.8)	18.3 (7.6–32.6)<br>96.3 (62.4–141.2)
**Moldova**	*Staphylococcus aureus*<br>*Escherichia coli*	Methicillin<br>Aminopenicillin	91.3 (40.7–159)<br>16529.7 (10820–24427.3)	1.7 (0.8–3)<br>352.3 (232.7–509.8)
**Monaco**	*Staphylococcus aureus*<br>*Escherichia coli*	Methicillin<br>Aminopenicillin	9.5 (3.7–17.6)<br>125.2 (74.3–195.1)	12.2 (4.6–23.1)<br>159.4 (93.4–250.4)
**Montenegro**	*Escherichia coli*<br>*Escherichia coli*	Third-generation cephalosporins<br>Aminopenicillin	176.1 (69.6–334.5)<br>1282.5 (746–1992.5)	20.1 (7.9–38.3)<br>143.4 (83.5–222.3)
**Netherlands**	*Enterococcus faecium*<br>*Escherichia coli*	Fluoroquinolones<br>Aminopenicillin	2915.3 (834.8–5506.6)<br>34667.7 (23373.1–49713.1)	9.9 (2.8–18.8)<br>112.5 (75.3–162.9)
**North Macedonia**	*Staphylococcus aureus*<br>*Escherichia coli*	Methicillin<br>Trimethoprim-sulfamethoxazole	951.9 (409.3–1787.2)<br>6065.8 (3406.2–9697.3)	34.9 (15.1–65.6)<br>217.5 (123.3–346.5)
**Norway**	*Escherichia coli*<br>*Escherichia coli*	Carbapenems<br>Aminopenicillin	1243.5 (710.6–1967.4)<br>7206 (4727.4–10701)	13.7 (7.8–22.1)<br>80 (51.8–120.1)
**Poland**	*Staphylococcus aureus*<br>*Escherichia coli*	Methicillin<br>Aminopenicillin	9609.8 (3933.8–17516.3)<br>110588.5 (67352–174610.9)	16.2 (6.6–29.5)<br>182.4 (109.9–287.3)
**Portugal**	*Staphylococcus aureus*<br>*Escherichia coli*	Methicillin<br>Aminopenicillin	6140 (2691.5–10143.6)<br>32397.7 (22099.3–45717.6)	28.6 (12.4–48.6)<br>151.6 (101.6–216.1)
**Romania**	*Staphylococcus aureus*<br>*Escherichia coli*	Methicillin<br>Aminopenicillin	14548.2 (6826.8–26214.3)<br>78679.3 (47809.4–123566.7)	53 (24.8–93.7)<br>257.4 (158.3–403.5)
**Russia**	*Escherichia coli*<br>*Escherichia coli*	Third-generation cephalosporins<br>Aminopenicillin	75228.3 (32343.4–143647.4)	37.3 (16.2–71.4)<br>336.9 (212.2–496.6)

			678298.6 (430234.9–999951.1)	
**San Marino**	*Staphylococcus aureus*<br>*Escherichia coli*	Methicillin<br>Aminopenicillin	8.6 (3.3–16.7)<br>66.6 (34.8–114)	15.9 (5.9–30.8)<br>118.4 (61.7–203)
**Serbia**	*Staphylococcus aureus*<br>*Escherichia coli*	Methicillin<br>Aminopenicillin	3138.8 (1204–6027.8)<br>28939.7 (16864.4–45086.4)	22.7 (8.7–44)<br>201.2 (116–315.7)
**Slovakia**	*Staphylococcus aureus*<br>*Escherichia coli*	Methicillin<br>Aminopenicillin	2105.3 (876.5–3795.4)<br>16766.7 (9880.3–25785.2)	26.8 (11.1–48.8)<br>207.2 (121.5–320.3)
**Slovenia**	*Enterococcus faecium*<br>*Escherichia coli*	Fluoroquinolones<br>Aminopenicillin	419.5 (122.4–832.5)<br>4482.7 (2619.2–7183.3)	11.3 (3.3–22.7)<br>117.9 (69.1–189.6)
**Spain**	*Staphylococcus aureus*<br>*Escherichia coli*	Methicillin<br>Aminopenicillin	13252.4 (5554.8–23829.2)<br>121892.8 (83323.7–173589.2)	15.8 (6.6–28.8)<br>138.7 (93.5–199.9)
**Sweden**	*Enterococcus faecium*<br>*Escherichia coli*	Fluoroquinolones<br>Aminopenicillin	1317.3 (375.9–2536.8)<br>11440.3 (7315–17248.1)	7.6 (2.1–14.6)<br>60.3 (38.1–92)
**Switzerland**	*Enterococcus faecium*<br>*Escherichia coli*	Fluoroquinolones<br>Aminopenicillin	1110.7 (317.7–2088.5)<br>12552.2 (8241.8–18135.1)	7.3 (2.1–13.9)<br>78.5 (50.9–114.3)
**Tajikistan**	*Streptococcus pneumoniae*<br>*Streptococcus pneumoniae*	Carbapenems<br>Trimethoprim-sulfamethoxazole	7742.5 (3466.2–13829.4)<br>56569.8 (40314.5–78262.5)	71.5 (32.1–127)<br>522.7 (376.6–721.8)
**Turkey**	*Staphylococcus aureus*<br>*Staphylococcus aureus*	Methicillin<br>Methicillin	46468 (21500–80443.7)<br>159270.3 (104543.3–239410.7)	61.2 (28.2–105.4)<br>209.8 (138.6–309.5)
**Turkmenistan**	*Staphylococcus aureus*<br>*Escherichia coli*	Methicillin<br>Aminopenicillin	2349.3 (931.1–4210.2)<br>19505.1 (13342.5–27858.1)	47.5 (18.9–85.3)<br>409.3 (276–584.2)
**Ukraine**	*Escherichia coli*<br>*Escherichia coli*	Third-generation cephalosporins<br>Aminopenicillin	14056.2 (5316.3–28229.7)<br>154658.4 (91901.6–232677.1)	23.1 (8.8–46.4)<br>254.6 (153.2–382)
**UK**	*Staphylococcus aureus*<br>*Escherichia coli*	Methicillin<br>Aminopenicillin	13960.4 (5956.5–24626)<br>178441.5 (120170.5–254460.2)	12.5 (5.3–22.3)<br>159.6 (106–230.4)
**Uzbekistan**	*Klebsiella pneumoniae*<br>*Streptococcus pneumoniae*	Third-generation cephalosporins<br>Trimethoprim-sulfamethoxazole	15220.8 (3935.6–31018.9)<br>127989.7 (98532.6–166030)	54.5 (14–112)<br>390.3 (298.7–501.5)

1094

Supplementary Table 3. The overall antimicrobial resistance burden in deaths and DALYs attributable to and associated with AMR by GBD region in 2019 that belong the WHO European Region.

GBD Region	Attributable to AMR				Associated with AMR			
	Deaths		DALYs		Deaths		DALYs	
	Counts	Rate per 100k	Counts	Rate per 100k	Counts	Rate per 100k	Counts	Rate per 100k
Central Asia	12,200 (8,410-17,300)	13.6 (9.3-19.1)	486,000 (353,000-667,000)	538.6 (391.1-740)	47,200 (33,300-66,100)	52.4 (37-73.3)	1,910,000 (1,410,000-2,550,000)	2,116.4 (1,565.6-2,827.2)
Central Europe	19,000 (12,000-28,500)	16.6 (10.5-25)	391,000 (244,000-591,000)	342.7 (214-517.2)	77,600 (49,400-115,000)	68 (43.2-100.9)	1,600,000 (1,010,000-2,380,000)	1,402.6 (881.9-2,082.2)
Eastern Europe	41,800 (27,600-59,900)	19.9 (13.1-28.5)	1,090,000 (732,000-1,520,000)	519 (348.6-723.9)	155,000 (103,000-222,000)	74 (48.8-105.6)	4,030,000 (2,700,000-5,670,000)	1,917.5 (1,287.4-2,698.6)
North Africa and Middle East	8,840 (5,670-13,300)	10.9 (7-16.3)	212,000 (134,000-319,000)	260 (165-391.9)	31,900 (21,100-47,600)	39.2 (25.9-58.4)	771,000 (505,000-1,150,000)	947.4 (620.9-1,407.4)
Western Europe	51,000 (35,100-72,300)	11.7 (8-16.6)	801,000 (535,000-1,160,000)	183.8 (122.7-266.2)	229,000 (161,000-318,000)	52.5 (37-73)	3,600,000 (2,450,000-5,120,000)	826.7 (562.1-1,175.1)

Supplementary Table 4. Relative risk estimates for sterile sources of specimen across 88 pathogen-drug combinations.

Pathogen	Drug	Sample size	Mean relative risk	Lower bound	Upper bound
Acinetobacter baumannii	Anti-pseudomonal penicillin/Beta-lactamase inhibitors	948	1.31	1.12	1.52
Acinetobacter baumannii	Beta-lactam/Beta-lactamase inhibitors	1555	1.27	1.11	1.44
Acinetobacter baumannii	Carbapenem	3232	1.42	1.27	1.58
Acinetobacter baumannii	Fourth-generation cephalosporins	1439	1.31	1.14	1.51
Acinetobacter baumannii	Third-generation cephalosporins	2055	1.35	1.13	1.62
Acinetobacter baumannii	Aminoglycosides	2066	1.1	0.97	1.25
Acinetobacter baumannii	Fluoroquinolones	3020	1.38	1.21	1.56
Citrobacter spp.	Aminoglycosides	4069	1.09	0.94	1.28
Citrobacter spp.	Anti-pseudomonal penicillin/Beta-lactamase inhibitors	3127	1.32	1.14	1.53
Citrobacter spp.	Carbapenem	3097	1.48	1.25	1.76
Citrobacter spp.	Fluoroquinolones	4387	1.36	1.18	1.57
Citrobacter spp.	Fourth-generation cephalosporins	2718	1.31	1.1	1.56
Citrobacter spp.	Third-generation cephalosporins	3984	1.38	1.16	1.64
Enterobacter spp.	Aminoglycosides	15211	1.19	1.06	1.34
Enterobacter spp.	Anti-pseudomonal penicillin/Beta-lactamase inhibitors	11857	1.23	1.13	1.34
Enterobacter spp.	Carbapenem	13299	1.53	1.4	1.67
Enterobacter spp.	Fluoroquinolones	17552	1.28	1.17	1.4
Enterobacter spp.	Fourth-generation cephalosporins	11482	1.31	1.18	1.45
Enterobacter spp.	Trimethoprim-Sulfamethoxazole	14798	1.09	0.98	1.21
Enterococcus faecalis	Fluoroquinolones	1126	1.43	1.24	1.64
Enterococcus faecalis	Vancomycin	36	1.7	1.39	2.07
Enterococcus faecium	Fluoroquinolones	4082	1.37	1.14	1.64
Enterococcus faecium	Vancomycin	9242	1.54	1.39	1.7
Other Enterococci	Fluoroquinolones	107	1.28	1.07	1.55
Other Enterococci	Vancomycin	7730	1.37	1.29	1.46
Escherichia coli	Aminoglycosides	164196	1.2	1.16	1.25
Escherichia coli	Aminopenicillin	157276	1.21	1.17	1.25
Escherichia coli	Beta-lactam/Beta-lactamase inhibitors	143458	1.15	1.11	1.18
Escherichia coli	Carbapenem	131382	1.7	1.5	1.93
Escherichia coli	Trimethoprim-Sulfamethoxazole	164240	1.14	1.11	1.18
Group A *Streptococcus*	Macrolide	130	1.07	0.89	1.29
Group B *Streptococcus*	Fluoroquinolones	44	1.26	1.04	1.53
Group B *Streptococcus*	Macrolide	465	1.18	0.99	1.41
Group B *Streptococcus*	Penicillin	15	1.29	1.06	1.57
Haemophilus influenzae	Aminopenicillin	1438	1.27	1.06	1.51
Haemophilus influenzae	Third-generation cephalosporins	308	1.48	1.23	1.79
Klebsiella pneumoniae	Aminoglycosides	51811	1.24	1.17	1.32
Klebsiella pneumoniae	Beta-lactam/Beta-lactamase inhibitors	46753	1.19	1.13	1.25
Klebsiella pneumoniae	Fluoroquinolones	53414	1.19	1.12	1.26

Klebsiella pneumoniae	Trimethoprim-Sulfamethoxazole	51737	1.12	1.06	1.19
Morganella spp.	Fluoroquinolones	3290	1.26	1.1	1.44
Morganella spp.	Fourth-generation cephalosporins	2352	1.23	1.02	1.49
Morganella spp.	Third-generation cephalosporins	3407	1.33	1.12	1.58
Proteus spp.	Aminoglycosides	21844	1.1	1.01	1.2
Proteus spp.	Aminopenicillin	20638	1.01	0.94	1.09
Proteus spp.	Fluoroquinolones	22141	1.13	1.05	1.21
Proteus spp.	Trimethoprim-Sulfamethoxazole	21838	1.06	0.98	1.14
Proteus spp.	Third-generation cephalosporins	18775	1.27	1.08	1.5
Pseudomonas aeruginosa	Aminoglycosides	39341	1.03	0.98	1.09
Pseudomonas aeruginosa	Anti-pseudomonal penicillin/Beta-lactamase inhibitors	36016	1.3	1.22	1.37
Pseudomonas aeruginosa	Carbapenem	41177	1.27	1.22	1.32
Pseudomonas aeruginosa	Fluoroquinolones	47417	1.19	1.15	1.23
Pseudomonas aeruginosa	Fourth-generation cephalosporins	34020	1.24	1.17	1.31
Pseudomonas aeruginosa	Third-generation cephalosporins	31041	1.35	1.15	1.59
Serratia spp.	Aminoglycosides	5250	1.05	0.93	1.19
Serratia spp.	Anti-pseudomonal penicillin/Beta-lactamase inhibitors	3003	1.17	1.01	1.35
Serratia spp.	Carbapenem	3639	1.39	1.2	1.63
Serratia spp.	Fluoroquinolones	5252	1.09	0.94	1.26
Serratia spp.	Fourth-generation cephalosporins	3928	1.17	0.99	1.38
Serratia spp.	Third-generation cephalosporins	5960	1.29	1.09	1.52
Staphylococcus aureus	Fluoroquinolones	37963	1.07	1.02	1.11
Staphylococcus aureus	Macrolide	53005	1.06	1.02	1.09
Staphylococcus aureus	Trimethoprim-Sulfamethoxazole	59632	1.17	1.09	1.25
Streptococcus pneumoniae	Beta-lactam/Beta-lactamase inhibitors	1419	1.14	0.95	1.37
Streptococcus pneumoniae	Carbapenem	1947	1.37	1.16	1.61
Streptococcus pneumoniae	Fluoroquinolones	6499	1.23	1.05	1.45
Streptococcus pneumoniae	Macrolide	7348	1.05	0.94	1.17
Streptococcus pneumoniae	Trimethoprim-Sulfamethoxazole	5413	1.14	1.01	1.28
Streptococcus pneumoniae	Third-generation cephalosporins	10457	1.33	1.13	1.57
Escherichia coli	Fluoroquinolones	171311	1.31	1.27	1.35
Escherichia coli	Third-generation cephalosporins	163801	1.37	1.17	1.61
Klebsiella pneumoniae	Carbapenem	41943	1.68	1.56	1.82
Klebsiella pneumoniae	Third-generation cephalosporins	52090	1.36	1.16	1.6
Mycobacterium tuberculosis	Extensive drug resistance	428524	2.59	2.46	2.72
Mycobacterium tuberculosis	Isoniazid mono-resistance	14537	1.19	0.84	1.67
Mycobacterium tuberculosis	Multidrug resistance	427342	2.5	1.17	4.74
Mycobacterium tuberculosis	Rifampicin mono-resistance	7161	1.39	1.06	1.77
Non-typhoidal *Salmonella*	Fluoroquinolones	42	1.23	1.01	1.5
Salmonella Paratyphi	Fluoroquinolones	24	1.24	1.02	1.52
Salmonella Paratyphi	Multidrug resistance	25	1.24	1.03	1.5
Salmonella Typhi	Fluoroquinolones	24	1.24	1.02	1.52
Salmonella Typhi	Multidrug resistance	25	1.24	1.03	1.5
Shigella spp.	Fluoroquinolones	24	1.24	1.02	1.52

Staphylococcus aureus	Methicillin	95696	1.43	1.2	1.7
Streptococcus pneumoniae	Penicillin	30849	1.27	1.18	1.36
Staphylococcus aureus	Vancomycin	53623	1.52	1.28	1.81

Sample size are the admission reported with known discharge disposition and antimicrobial susceptibility test.

Supplementary Table 5. Data points (cases or death) included in each primary modelling step by GBD region and the fraction of countries represented in each GBD region.

Region	1. Sepsis and Infectious Syndrome Models	Fraction of countries represented in 1.	2. Case Fatality Rate	Fraction of countries represented in 2.	3. Pathogen Distribution	Fraction of countries represented in 3.	4. Fraction of Resistance	Fraction of countries represented in 4.	5. Relative Risk	Fraction of countries represented in 5.
Central Asia	0	0/9	0	0/9	363	4/9	304,794	9/9	6,970	3/9
Central Europe	0	0/13	5,390	9/13	457,010	11/13	3,152,483	13/13	391,586	10/13
Eastern Europe	0	0/7	1,710	3/7	49,313	6/7	999,227	7/7	107,839	4/7
North Africa and Middle East	0	0/21	28,461	12/21	45,062	20/21	539,417	21/21	90,654	10/21
Western Europe	10,389,042	2/24	11,999,555	17/24	7,606,982	21/24	19,096,988	21/24	1,105,356	21/24

Data points are sourced from a variety of sources including, but not limited to, multiple cause of death data, hospital discharges, literature studies, and microbiology data with and without outcome.
Several data sources inform multiple modeling steps. Therefore, data points should not be summed across a row as that will lead to duplication.
For more information on the data types used and the modeling steps that they inform, see section 2 of the appendix.

Supplementary Table 6. Overall antimicrobial resistance burden by steps in the estimation, 2019.

Country	All-cause deaths		Deaths that involved infection			Deaths associated with AMR			Attributable to AMR		
	Counts	Rate per 100,000	Fraction of all deaths that involve infection	Counts	Rate per 100,000	Fraction of deaths involving infection that are associated with resistance	Counts	Rate per 100,000	Fraction of deaths involving infection that are attributable to resistance	Counts	Rate per 100,000
Albania	22,700	833.4	11.0%	2,490	91.6	52.0%	1,300	47.9	13.5%	339	12.4
Andorra	620	746.1	14.4%	89	107.6	35.5%	32	38.3	7.8%	7	8.4
Armenia	28,000	926.5	13.6%	3,820	126.5	51.7%	1,980	65.5	13.0%	498	16.5
Austria	82,500	925.2	10.6%	8,740	98	35.9%	3,150	35.3	7.3%	640	7.2
Azerbaijan	75,100	730.9	14.4%	10,900	105.7	50.0%	5,440	53	12.6%	1,370	13.3
Belarus	122,000	1281.8	8.8%	10,700	112.5	49.0%	5,250	55.3	12.8%	1,360	14.4
Belgium	114,000	999	17.2%	19,600	171.9	34.3%	6,760	59.2	7.2%	1,410	12.3
Bosnia and Herzegovina	37,400	1134.1	11.2%	4,200	127.4	46.8%	1,970	59.8	12.0%	506	15.3
Bulgaria	124,000	1791.4	10.5%	13,000	187.6	48.9%	6,390	92.1	12.6%	1,650	23.7
Croatia	52,300	1231.5	10.2%	5,330	125.5	47.6%	2,550	59.9	11.5%	614	14.4
Cyprus	8,710	662.9	13.2%	1,150	87.3	47.6%	547	41.6	12.2%	140	10.7
Czech Republic	114,000	1069.2	12.2%	13,900	130.1	37.7%	5,250	49.3	8.2%	1,140	10.7
Denmark	55,400	954.3	15.4%	8,550	147.4	28.3%	2,430	41.9	5.7%	488	8.4
Estonia	15,900	1210.7	9.6%	1,530	116.7	38.5%	592	45.1	8.4%	130	9.9

Finland	56,100	1014	10.0%	5,620	101.5	29.0%	1,640	29.6	6.2%	349	6.3
France	603,000	911.2	14.9%	89,600	135.4	36.3%	32,600	49.3	8.0%	7,160	10.8
Georgia	49,400	1348.5	11.2%	5,520	150.6	50.2%	2,780	75.7	13.7%	758	20.7
Germany	960,000	1130.4	13.1%	126,000	148.2	36.2%	45,700	53.8	7.6%	9,650	11.4
Greece	129,000	1244.7	12.9%	16,700	161.1	46.9%	7,830	75.7	11.9%	1,990	19.3
Hungary	129,000	1332	10.5%	13,500	139.7	48.1%	6,520	67.4	11.5%	1,550	16
Iceland	2,110	612.8	13.2%	280	81.2	30.2%	85	24.6	6.4%	18	5.2
Ireland	32,400	658.9	14.8%	4,790	97.6	36.7%	1,770	36	7.9%	378	7.7
Israel	47,900	514.8	17.3%	8,290	89	45.4%	3,780	40.6	10.9%	903	9.7
Italy	642,000	1065	11.3%	72,300	119.9	49.4%	35,800	59.4	12.1%	8,780	14.6
Kazakhstan	139,000	758.3	14.4%	20,100	109.1	47.3%	9,500	51.7	11.9%	2,380	12.9
Kyrgyzstan	34,700	530.6	15.1%	5,240	80.3	51.0%	2,680	41	14.0%	732	11.2
Latvia	27,400	1432	10.1%	2,780	145.3	43.9%	1,230	64.1	10.2%	286	14.9
Lithuania	38,500	1377.9	10.5%	4,050	144.9	45.9%	1,860	66.7	10.7%	432	15.5
Luxembourg	4,150	670.5	14.0%	580	93.8	35.7%	208	33.6	7.7%	45	7.2
Macedonia	24,000	1117	10.0%	2,410	111.9	52.3%	1,260	58.7	14.8%	358	16.6
Malta	3,780	860.7	15.1%	573	130.4	38.5%	221	50.4	9.3%	53	12.1
Moldova	41,000	1111.6	12.9%	5,290	143.5	53.0%	2,810	76.2	14.2%	752	20.4
Monaco	524	1394.1	13.8%	72	193	38.4%	28	74.4	8.1%	6	15.7
Montenegro	6,790	1095.2	9.2%	625	100.8	50.0%	314	50.6	13.1%	82	13.3
Netherlands	157,000	915.1	15.4%	24,200	141.3	30.3%	7,370	43	5.8%	1,410	8.2
Norway	41,400	773.7	15.4%	6,350	118.8	27.5%	1,760	32.8	6.7%	427	8

Poland	406,000	1057.1	11.9%	48,600	126.4	49.4%	24,100	62.7	11.5%	5,620	14.6
Portugal	116,000	1092.7	18.9%	22,000	206.3	42.9%	9,450	88.7	10.1%	2,230	20.9
Romania	263,000	1366.2	11.9%	31,300	162.7	52.7%	16,500	86	13.7%	4,290	22.3
Russian Federation	1,790,000	1218.9	12.3%	219,000	149.3	51.0%	112,000	76.4	13.9%	30,500	20.8
San Marino	302	911.2	12.8%	38	116.4	39.4%	15	46.1	9.0%	3	10.5
Serbia	118,000	1344.8	10.7%	12,600	144	56.0%	7,080	80.9	14.1%	1,780	20.4
Slovakia	54,500	1003.3	12.5%	6,830	125.6	49.9%	3,420	62.8	11.9%	814	15
Slovenia	20,800	1003.4	12.6%	2,630	126.7	35.9%	948	45.7	8.2%	216	10.4
Spain	429,000	931.3	15.1%	64,600	140.5	42.1%	27,300	59.3	9.6%	6,220	13.5
Sweden	93,800	917.6	12.7%	11,900	116.1	23.1%	2,750	26.9	4.9%	581	5.7
Switzerland	69,800	795.6	13.2%	9,250	105.4	28.2%	2,620	29.8	6.1%	563	6.4
Tajikistan	48,700	513.1	22.1%	10,800	113.3	44.2%	4,770	50.2	12.0%	1,300	13.7
Turkey	455,000	558.9	14.2%	64,700	79.5	49.2%	31,900	39.2	13.6%	8,840	10.9
Turkmenistan	33,600	661.4	17.2%	5,790	113.8	49.5%	2,870	56.5	12.8%	739	14.5
Ukraine	699,000	1586.3	10.2%	71,500	162.4	43.9%	31,500	71.5	11.7%	8,410	19.1
United Kingdom	622,000	925	17.9%	111,000	165.6	31.5%	35,200	52.3	6.8%	7,580	11.3
Uzbekistan	204,000	604.6	17.4%	35,300	105	48.6%	17,200	51.1	12.6%	4,450	13.2

1103

1104 **Supplementary Table 7:** AMR burden by top three pathogens for each country in the WHO European Region

Country	*Escherichia coli*		*Klebsiella pneumoniae*		*Staphylococcus aureus*	
	**Crude mortality rate per 100,000 person/year associated with AMR (rate, (UI))**	**Crude mortality rate per 100,000 person/year attributable to AMR (rate, (UI))**	**Crude mortality rate per 100,000 person/year associated with AMR (rate, (UI))**	**Crude mortality rate per 100,000 person/year attributable to AMR (rate, (UI))**	**Crude mortality rate per 100,000 person/year associated with AMR (rate, (UI))**	**Crude mortality rate per 100,000 person/year attributable to AMR (rate, (UI))**
Albania	14.6 (8.1 - 23.5)	3.8 (2.1 - 6.5)	6.9 (4 - 10.9)	1.9 (1 - 3.3)	8.2 (5.2 - 12.6)	2.3 (1.1 - 3.9)
Andorra	14.7 (9.2 - 22.9)	3.5 (2.1 - 5.7)	3.8 (2.5 - 5.8)	0.8 (0.5 - 1.3)	6 (4 - 9.2)	1 (0.6 - 1.7)
Armenia	16.3 (11 - 22.9)	4.2 (2.7 - 6.3)	10.6 (7 - 15.1)	2.9 (1.6 - 4.7)	7.2 (4.8 - 10.4)	1.8 (0.9 - 2.9)
Austria	12.2 (7.6 - 18.7)	2.6 (1.6 - 3.9)	2.8 (1.8 - 4.2)	0.6 (0.4 - 0.9)	6.4 (4.2 - 9.4)	1 (0.5 - 1.6)
Azerbaijan	9.6 (6.4 - 13.9)	2.4 (1.5 - 3.7)	8.2 (5.4 - 12)	2.2 (1.2 - 3.5)	7.9 (5.4 - 11.4)	1.5 (0.8 - 2.4)
Belarus	14.3 (8.7 - 22.4)	3.7 (2.1 - 6.2)	8 (4.9 - 12.7)	2.3 (1.3 - 3.9)	3.9 (2.3 - 6.2)	0.9 (0.4 - 1.7)
Belgium	19.4 (13.2 - 27.2)	4.1 (2.7 - 6)	5.5 (4 - 7.4)	1.4 (0.9 - 2)	12.5 (9.6 - 16.2)	2.2 (1.3 - 3.5)
Bosnia and Herzegovina	12.8 (7.5 - 19.9)	3.2 (1.8 - 5.2)	8.4 (4.9 - 13.1)	2.5 (1.4 - 4.2)	7 (4 - 10.7)	1.6 (0.8 - 2.8)
Bulgaria	29.3 (16.5 - 46.7)	7.3 (4 - 12.1)	14.7 (8.9 - 22.9)	4.6 (2.6 - 7.5)	8.2 (5.2 - 12.3)	1.8 (0.9 - 3.2)
Croatia	16.3 (10.4 - 23.9)	3.5 (2.2 - 5.2)	6.6 (4.1 - 9.9)	1.8 (1 - 2.9)	7.9 (4.7 - 12.3)	1.9 (0.9 - 3.3)
Cyprus	14.5 (9.9 - 20.7)	3.6 (2.3 - 5.4)	4.6 (3.2 - 6.6)	1.4 (0.9 - 2.2)	8.5 (5.9 - 12.1)	2.2 (1.1 - 3.6)
Czechia	12.2 (7.7 - 17.9)	2.7 (1.7 - 4.1)	6.8 (4.4 - 9.9)	1.7 (1 - 2.7)	6.7 (4.3 - 9.8)	1.2 (0.6 – 2.1)
Denmark	15.6 (10.2 - 22.8)	3.1 (2 - 4.6)	5.5 (3.8 - 7.8)	1 (0.6 - 1.5)	4.1 (3 - 5.4)	0.7 (0.5 - 1.1)
Estonia	10.9 (6.8 - 16.8)	2.2 (1.3 - 3.5)	6.2 (3.9 - 9.7)	1.2 (0.7 - 2.1)	3.8 (2.3 - 6.1)	0.6 (0.3 - 1)
Finland	9.8 (6.3 - 14.5)	2.1 (1.3 - 3.1)	3.2 (2 - 4.7)	0.6 (0.4 - 0.9)	2.1 (1.4 - 3.2)	0.5 (0.3 - 0.9)
France	16 (10.5 - 23.2)	3.3 (2.1 - 5)	5.5 (3.9 - 7.7)	1.4 (0.8 - 2.1)	8.3 (6 - 11.4)	1.7 (1 - 2.7)
Georgia	14.8 (8.9 - 22.5)	4.3 (2.5 - 6.9)	12.7 (7.8 - 19.5)	4.2 (2.4 - 6.7)	8.7 (5.4 - 13.1)	1.9 (1 - 3.4)
Germany	19.6 (12.9 - 28.4)	4.2 (2.7 - 6.3)	5.1 (3.5 - 7.2)	1.1 (0.7 - 1.7)	9.6 (6.7 - 13.1)	1.6 (0.9 - 2.6)
Greece	19 (12.2 - 28.3)	4.4 (2.7 - 6.8)	10.1 (7.1 - 14.1)	3.5 (2.3 - 5.2)	19.6 (14.9 - 25.8)	4.8 (2.7 - 7.6)
Hungary	21.7 (12.8 - 33.2)	5 (2.9 - 8)	8 (4.9 - 12.1)	2 (1.1 - 3.4)	11.3 (7.1 - 17)	2.6 (1.3 - 4.5)
Iceland	7.9 (5.1 - 11.8)	1.7 (1.1 - 2.6)	3 (2 - 4.2)	0.6 (0.4 - 1)	3.9 (2.8 - 5.5)	0.8 (0.4 - 1.2)

Ireland	11.3 (7.6 - 16.3)	2.4 (1.6 - 3.6)	3.9 (2.8 - 5.3)	0.8 (0.5 - 1.2)	7.4 (5.6 - 9.7)	1.3 (0.8 - 2)
Israel	13.2 (9 - 18.4)	3 (2 - 4.5)	4.3 (3 - 6)	1.1 (0.6 - 1.9)	9.5 (7 - 12.5)	2.4 (1.3 - 3.8)
Italy	18.8 (11.7 - 28.3)	4.5 (2.8 - 7)	7 (4.7 - 10.1)	2.1 (1.4 - 3.3)	13.2 (8.9 - 18.7)	3 (1.7 - 5)
Kazakhstan	9.6 (6.3 - 14.3)	2.1 (1.3 - 3.2)	8.2 (5.4 - 12.1)	2.2 (1.2 - 3.7)	6.7 (4.6 - 9.7)	1.6 (0.8 - 2.7)
Kyrgyzstan	7.4 (4.8 - 10.7)	1.9 (1.2 - 2.9)	6 (3.9 - 9)	1.6 (0.9 - 2.7)	5.5 (3.7 - 8)	1.5 (0.8 - 2.6)
Latvia	19.7 (12.1 - 30.4)	4.6 (2.7 - 7.2)	9.1 (5.8 - 13.8)	2.2 (1.2 - 3.5)	6 (3.8 - 9)	1 (0.5 - 1.6)
Lithuania	21 (13.1 - 32.6)	4.5 (2.7 - 7.3)	11.7 (7.5 - 17.8)	2.8 (1.6 - 4.7)	6.5 (4.2 - 9.7)	1.1 (0.6 - 1.8)
Luxembourg	11.3 (7.2 - 16.6)	2.5 (1.5 - 3.7)	3.4 (2.3 - 4.9)	0.9 (0.5 - 1.4)	6.4 (4.4 - 8.9)	1.1 (0.6 - 1.8)
Malta	12.8 (8.7 - 18.1)	3 (2 - 4.3)	5.7 (4.1 - 7.9)	1.8 (1.2 - 2.6)	11.5 (8.3 - 15.7)	2.7 (1.5 - 4.2)
Monaco	22.9 (14.1 - 34.9)	5.2 (3.1 - 8.3)	6.8 (4.5 - 9.9)	1.4 (0.8 - 2.2)	17.7 (12.7 - 24.8)	2.9 (1.7 - 4.6)
Montenegro	12 (7 - 18.8)	3.1 (1.8 - 5.2)	6.9 (4.2 - 10.7)	2 (1.1 - 3.4)	6.2 (3.7 - 9.9)	1.7 (0.8 - 3.1)
Netherlands	16.1 (11.2 - 22.6)	3.3 (2.2 - 4.7)	3.6 (2.5 - 5.1)	0.7 (0.4 - 1.1)	7.4 (5.5 - 10)	0.9 (0.6 - 1.3)
North Macedonia	14.7 (8.2 - 23.4)	4.5 (2.5 - 7.6)	8 (4.6 - 12.5)	2.5 (1.3 - 4.1)	7.3 (4.2 - 11.6)	2.3 (1.1 - 4.2)
Norway	12.4 (8.5 - 17.9)	3.3 (2.2 - 4.9)	3.6 (2.6 - 4.9)	0.7 (0.5 - 1.1)	4.5 (3.3 - 6.2)	1.3 (0.7 - 2.2)
Poland	16.1 (9.9 - 24.9)	3.6 (2.2 - 5.8)	9.2 (6 - 13.9)	2.5 (1.5 - 4)	9.4 (6.4 - 13.3)	1.9 (1 - 3.1)
Portugal	23.7 (16.5 - 33)	5.2 (3.5 - 7.5)	10.8 (8.2 - 14.3)	3.1 (2.1 - 4.4)	24.4 (19.3 - 30.9)	5.7 (3.3 - 8.5)
Republic of Moldova	20.8 (13.3 - 31.8)	5.3 (3.1 - 8.3)	11.4 (7.5 - 17.1)	3.1 (1.7 - 5.1)	9.6 (6.6 - 13.6)	2.9 (1.5 - 4.8)
Romania	21 (12.7 - 33)	4.8 (2.8 - 7.8)	12.1 (7.8 - 18.1)	3.9 (2.4 - 6.1)	16.3 (11.1 - 23.6)	4.2 (2.2 - 7.1)
Russian Federation	21.8 (13.8 - 32.7)	5.8 (3.4 - 9.4)	11.2 (7.4 - 16.2)	4 (2.6 - 5.9)	7.7 (5.1 - 11)	2.1 (1.1 - 3.4)
San Marino	14.7 (8.1 - 24.1)	3.4 (1.8 - 5.6)	4.6 (2.6 - 7.6)	1 (0.5 - 1.6)	9 (5.2 - 14.5)	2.2 (1 - 3.9)
Serbia	20.6 (12 - 31.9)	5 (2.9 - 8.2)	11.3 (6.8 - 17.2)	3.5 (1.9 - 5.9)	13.5 (8.3 - 20.4)	2.7 (1.4 - 4.7)
Slovakia	17.3 (10.4 - 26.3)	4.2 (2.4 - 6.8)	9.4 (6 - 14)	2.4 (1.4 - 3.8)	12 (8.2 - 17.2)	2.7 (1.4 - 4.3)
Slovenia	13.7 (8 - 21.7)	3 (1.7 - 4.8)	5.3 (3.4 - 8.1)	1.2 (0.7 - 2.2)	5.5 (3.7 - 8.1)	1.5 (0.7 - 2.5)
Spain	19.9 (13.9 - 27.7)	4.4 (3 - 6.4)	5.4 (3.9 - 7.4)	1.3 (0.8 - 2)	11.6 (8.3 - 15.8)	2.7 (1.5 - 4.3)
Sweden	10.2 (6.7 - 15)	2.2 (1.4 - 3.2)	2.3 (1.6 - 3.3)	0.5 (0.3 - 0.8)	2.1 (1.5 - 2.9)	0.5 (0.3 - 0.7)
Switzerland	11.2 (7.5 - 16)	2.3 (1.5 - 3.4)	2 (1.4 - 2.8)	0.4 (0.3 - 0.7)	4.2 (3 - 5.9)	1 (0.6 - 1.7)
Tajikistan	7.1 (5.1 - 9.7)	2 (1.3 - 2.8)	6.3 (4.4 - 8.9)	1.7 (1 - 2.8)	5.6 (4 - 7.9)	1.5 (0.8 - 2.4)
Turkiye	7 (4.6 - 10.7)	1.8 (1.1 - 2.8)	5.6 (3.7 - 8.5)	1.8 (1.1 - 2.8)	8.8 (5.9 - 12.7)	2.8 (1.4 - 4.6)
Turkmenistan	10.3 (6.8 - 15.3)	2.7 (1.7 - 4.1)	8.2 (5.4 - 12.1)	2.1 (1.2 - 3.5)	8.4 (5.6 - 12.1)	1.6 (0.9 - 2.7)
Ukraine	16.1 (9.5 - 24.2)	4 (2.2 - 6.3)	9.5 (5.7 - 14.3)	2.5 (1.4 - 4.2)	3.6 (2.1 - 5.5)	0.7 (0.4 - 1.2)
United Kingdom	18.4 (12.8 - 25.7)	3.8 (2.6 - 5.5)	5.5 (4.1 - 7.4)	1.1 (0.7 - 1.6)	9.5 (7.4 - 12.3)	2 (1.2 - 3.1)
Uzbekistan	8.9 (6 - 12.9)	2.3 (1.4 - 3.5)	7.7 (5.3 - 11.1)	2.1 (1.2 - 3.4)	7.6 (5.4 - 10.6)	1.4 (0.8 - 2.3)

1105

Supplementary Table 8: Overall burden by infectious syndrome in 2019.

Infectious Syndrome	Overall infectious burden / Overall bacterial burden (susceptible and resistant)			
	Deaths		DALYs	
	Counts	Rate per 100k	Counts	Rate per 100k
BSI	367,000 / 319,000	2070.9 / 1797.9	8,226,000 / 6,973,000	46,000 / 38,800
Bacterial skin infections	56,000 / 45,000	293.6 / 236.3	994,000 / 810,000	5,150 / 4,200
Bone and joint infections	4,000 / 3,700	22.6 / 19.4	85,000 / 74,000	440 / 385
Cardiac infections	37,000 / 34,400	222.6 / 206.2	661,000 / 611,000	3,770 / 3,490
CNS infections	6,000 / 3,700	36.0 / 20.6	289,000 / 155,000	1,660 / 890
Diarrhoea	21,000 / 1,600	111.5 / 9.4	1,542,000 / 194,000	9,140 / 1,218
Gonorrhoea and chlamydia	-	-	24,000 / 4,300	120 / 25
Intra-abdominal infections	222,000 / 197,000	1,171.0 / 1,041.2	4,957,000 / 4,370,000	26,080 / 22,980
LRI and thorax infections	384,000 / 231,000	2,080.7 / 1,229.8	7,918,000 / 4,870,000	42,970 / 26,125
Tuberculosis	25,000 / 25,000	119.4 / 119.4	996,000 / 996,000	4,690 / 4,690
Typhoid, paratyphoid, and iNTS	384 / 384	1.2 / 1.2	19,000 / 19,000	44 / 44
UTI	79,000 / 75,000	424.6 / 404.0	1,292,000 / 1,233,000	7,060 / 6,735
All infectious syndromes	1,247,000 / 937,000	6731.9 / 5085.3	30,447,000 / 20,307,000	162,550 / 109,620

Both overall infectious burden (which includes susceptible, resistant and non-tested bacterial pathogens, as well as other groups of pathogens) and the overall susceptible and resistant bacterial burden are presented here in accordance with infectious syndromes. Estimates were aggregated across drugs, accounting for the co-occurrence of resistance to multiple drugs. For gonorrhoea and chlamydia, we did not estimate the fatal burden, thus only the DALY burden is presented. BSI=bloodstream infections. CNS=central nervous system. DALYs=disability-adjusted life-years. LRI=lower respiratory infections. iNTS=intestinal nontyphoidal salmonellae. UTI=urinary tract infections.

Supplementary Figure 1. Heatmap representing DALYs attributable to antimicrobial resistance (AMR) by pathogen–drug combination in the WHO European Region in 2019. Abbreviations: 3GC=third-generation cephalosporins. 4GC=fourth-generation cephalosporins. Anti-pseudomonal=anti-pseudomonal penicillin or beta-lactamase inhibitors. BL-BLI=β-lactam or β-lactamase inhibitors. MDR=multidrug resistance. Mono INH=isoniazid mono-resistance. Mono RIF=rifampicin mono-resistance. NA=not applicable. Resistance to 1+=resistance to one or more drug. S Paratyphi=*Salmonella enterica* serotype Paratyphi. S Typhi=*Salmonella enterica* serotype Typhi. TMP-SMX=trimethoprim-sulfamethoxazole. XDR=extensive drug resistance.

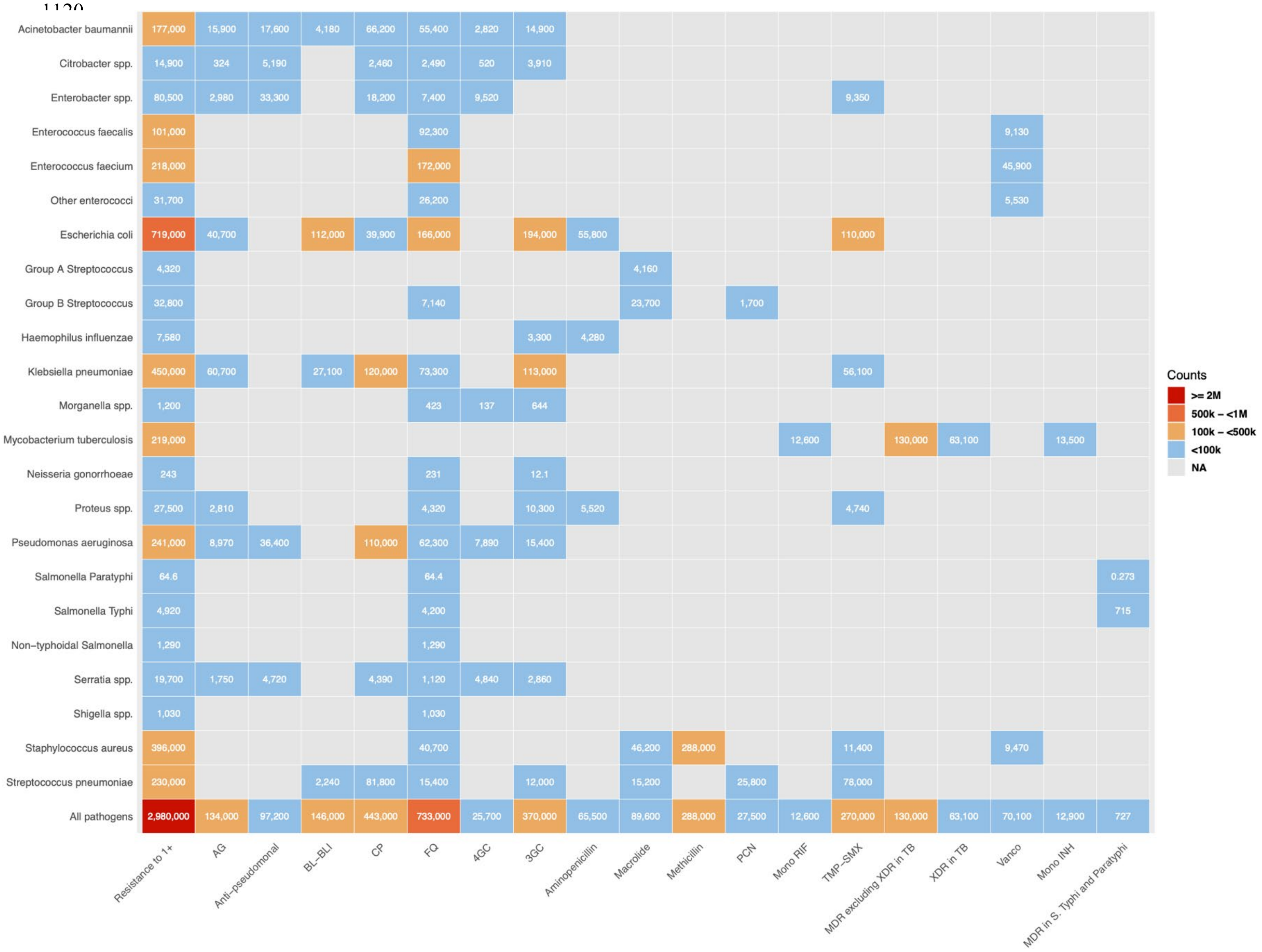
Acinetobacter baumannii
177,000
15,900
17,600
4,180
66,200
55,400
2,820
14,900
Citrobacter spp.
14,900
324
5,190
2,460
2,490
520
3,910
Enterobacter spp.
80,500
2,980
33,300
18,200
7,400
9,520
9,350
Enterococcus faecalis
101,000
92,300
9,130
Enterococcus faecium
218,000
172,000
45,900
Other enterococci
31,700
26,200
5,530
Escherichia coli
719,000
40,700
112,000
39,900
166,000
194,000
55,800
110,000
Group A Streptococcus
4,320
4,160
Group B Streptococcus
32,800
7,140
23,700
1,700
Haemophilus influenzae
7,580
3,300
4,280
Klebsiella pneumoniae
450,000
60,700
27,100
120,000
73,300
113,000
56,100
Morganella spp.
1,200
423
137
644
Mycobacterium tuberculosis
219,000
12,600
130,000
63,100
13,500
Neisseria gonorrhoeae
243
231
12.1
Proteus spp.
27,500
2,810
4,320
10,300
5,520
4,740
Pseudomonas aeruginosa
241,000
8,970
36,400
110,000
62,300
7,890
15,400
Salmonella Paratyphi
64.6
64.4
0.273
Salmonella Typhi
4,920
4,200
715
Non−typhoidal Salmonella
1,290
1,290
Serratia spp.
19,700
1,750
4,720
4,390
1,120
4,840
2,860
Shigella spp.
1,030
1,030
Staphylococcus aureus
396,000
40,700
46,200
288,000
11,400
9,470
Streptococcus pneumoniae
230,000
2,240
81,800
15,400
12,000
15,200
25,800
78,000
All pathogens
2,980,000
134,000
97,200
146,000
443,000
733,000
25,700
370,000
65,500
89,600
288,000
27,500
12,600
270,000
130,000
63,100
70,100
12,900
727
Resistance to 1+
AG
Anti−pseudomonal
BL−BLI
CP
FQ
4GC
3GC
Aminopenicillin
Macrolide
Methicillin
PCN
Mono RIF
TMP−SMX
MDR excluding XDR in TB
XDR in TB
Vanco
Mono INH
MDR in S. Typhi and Paratyphi
Counts
>= 2M
500k − <1M
100k − <500k
<100k
NA

Supplementary Figure 2. Heatmap representing DALYs associated with antimicrobial resistance (AMR) by pathogen–drug combination in the WHO European Region in 2019. Abbreviations: 3GC=third-generation cephalosporins. 4GC=fourth-generation cephalosporins. Anti-pseudomonal=anti-pseudomonal penicillin or beta-lactamase inhibitors. BL-BLI=β-lactam or β-lactamase inhibitors. MDR=multidrug resistance. Mono INH=isoniazid mono-resistance. Mono RIF=rifampicin mono-resistance. NA=not applicable. Resistance to 1+=resistance to one or more drug. S Paratyphi=*Salmonella enterica* serotype Paratyphi. S Typhi=*Salmonella enterica* serotype Typhi. TMP-SMX=trimethoprim-sulfamethoxazole. XDR=extensive drug resistance.

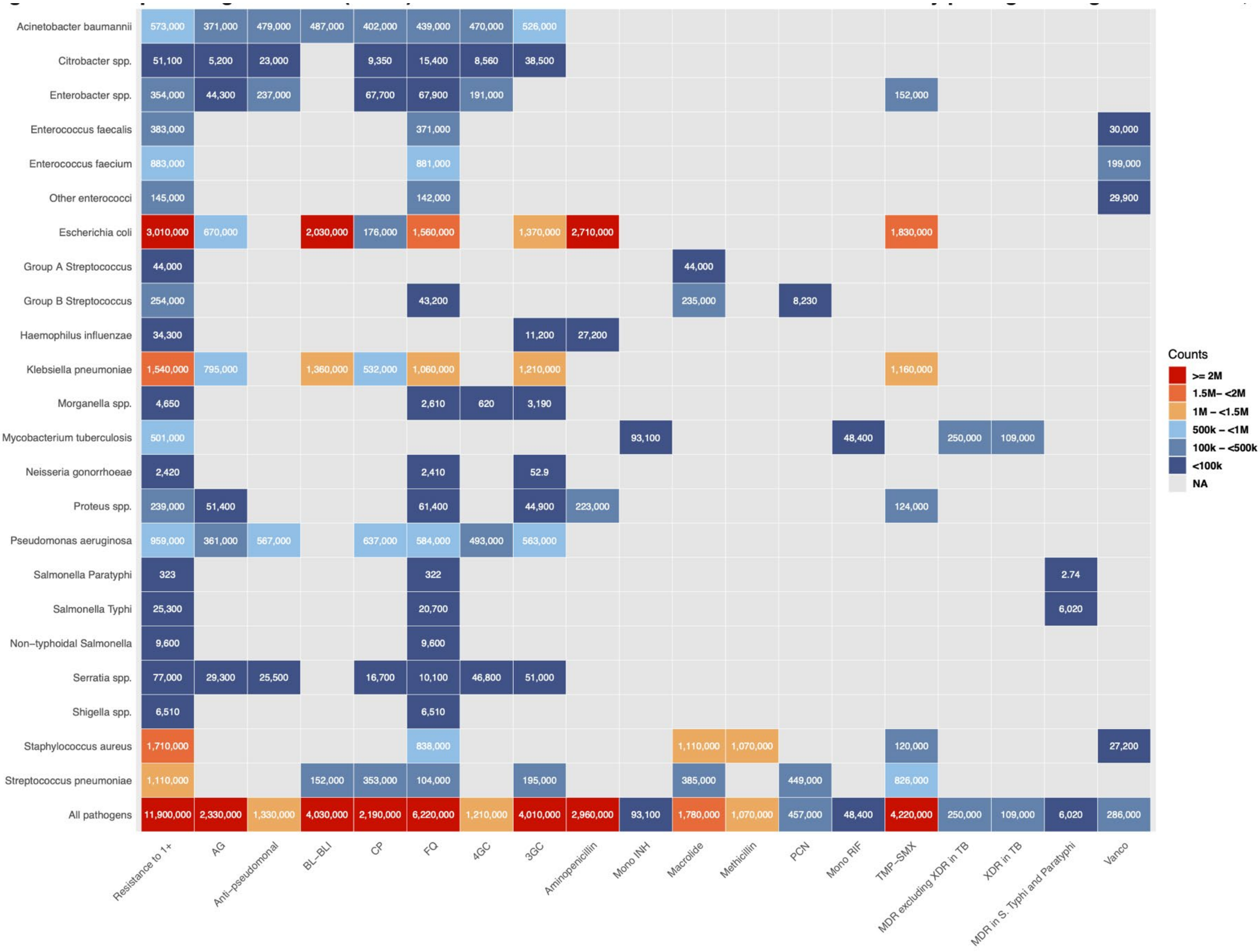

	Resistance to 1+	AG	Anti–pseudomonal	BL–BLI	CP	FQ	4GC	3GC	Aminopenicillin	Mono INH	Macrolide	Methicillin	PCN	Mono RIF	TMP–SMX	MDR excluding XDR in TB	XDR in TB	MDR in S. Typhi and Paratyphi	Vanco
Acinetobacter baumannii	573,000	371,000	479,000	487,000	402,000	439,000	470,000	526,000											
Citrobacter spp.	51,100	5,200	23,000		9,350	15,400	8,560	38,500											
Enterobacter spp.	354,000	44,300	237,000		67,700	67,900	191,000								152,000				
Enterococcus faecalis	383,000					371,000													30,000
Enterococcus faecium	883,000					881,000													199,000
Other enterococci	145,000					142,000													29,900
Escherichia coli	3,010,000	670,000		2,030,000	176,000	1,560,000		1,370,000	2,710,000						1,830,000				
Group A Streptococcus	44,000										44,000								
Group B Streptococcus	254,000					43,200					235,000		8,230						
Haemophilus influenzae	34,300							11,200	27,200										
Klebsiella pneumoniae	1,540,000	795,000		1,360,000	532,000	1,060,000		1,210,000							1,160,000				
Morganella spp.	4,650					2,610	620	3,190											
Mycobacterium tuberculosis	501,000									93,100				48,400		250,000	109,000		
Neisseria gonorrhoeae	2,420					2,410		52.9											
Proteus spp.	239,000	51,400				61,400		44,900	223,000						124,000				
Pseudomonas aeruginosa	959,000	361,000	567,000		637,000	584,000	493,000	563,000											
Salmonella Paratyphi	323					322												2.74	
Salmonella Typhi	25,300					20,700												6,020	
Non–typhoidal Salmonella	9,600					9,600													
Serratia spp.	77,000	29,300	25,500		16,700	10,100	46,800	51,000											
Shigella spp.	6,510					6,510													
Staphylococcus aureus	1,710,000					838,000					1,110,000	1,070,000			120,000				27,200
Streptococcus pneumoniae	1,110,000			152,000	353,000	104,000		195,000			385,000		449,000		826,000				
All pathogens	11,900,000	2,330,000	1,330,000	4,030,000	2,190,000	6,220,000	1,210,000	4,010,000	2,960,000	93,100	1,780,000	1,070,000	457,000	48,400	4,220,000	250,000	109,000	6,020	286,000

Supplementary Figure 3. Heatmap representing age-standardised death rates per 100,000 person years attributable to antimicrobial resistance (AMR) by pathogen and country for the WHO European Region in 2019.

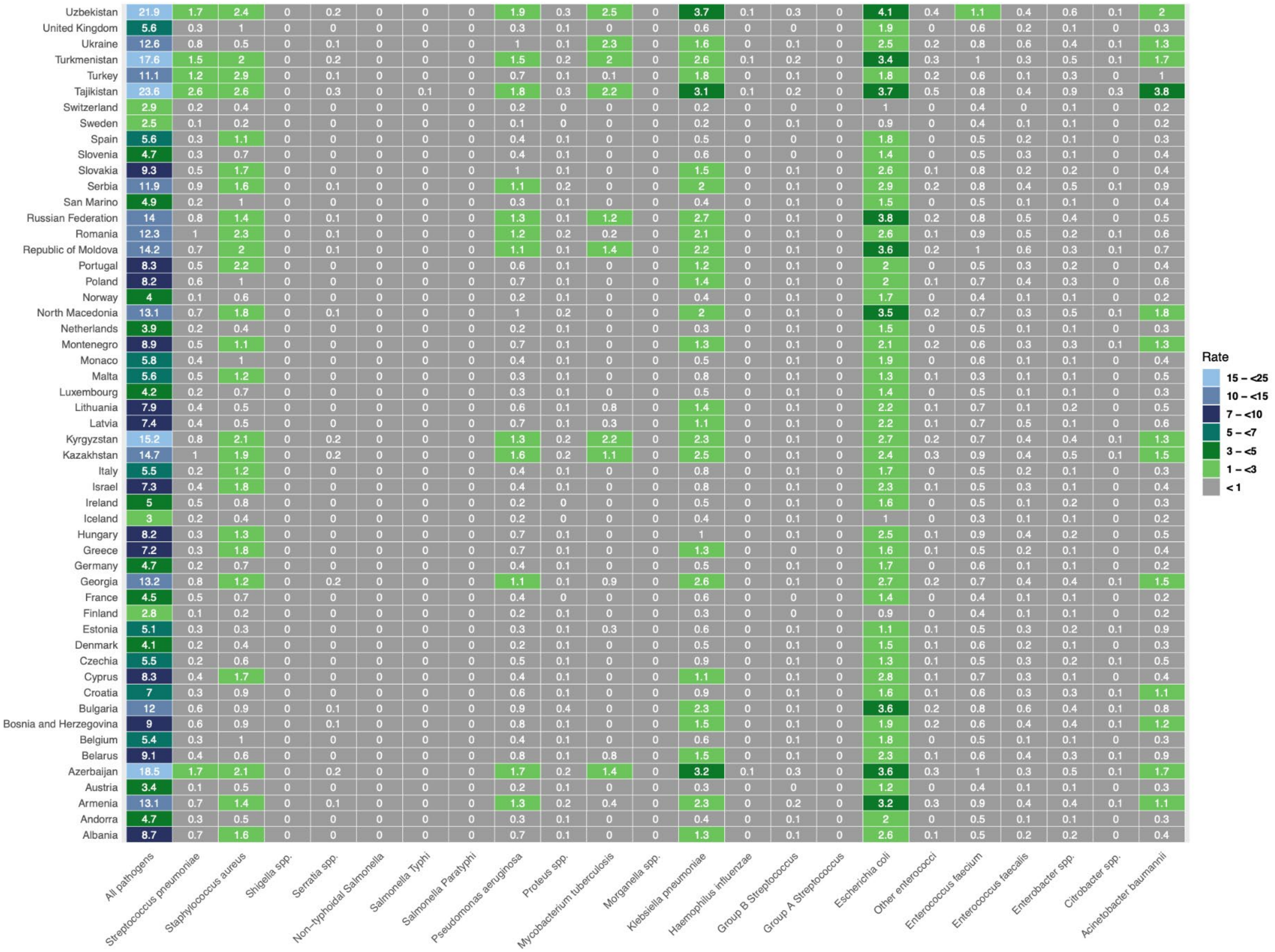

Supplementary Figure 4. Heatmap representing age-standardised death rates per 100,000 person years associated with antimicrobial resistance (AMR) by pathogen and country for the WHO European Region in 2019.

Country	All pathogens	Streptococcus pneumoniae	Staphylococcus aureus	Shigella spp.	Serratia spp.	Non-typhoidal Salmonella	Salmonella Typhi	Salmonella Paratyphi	Pseudomonas aeruginosa	Proteus spp.	Mycobacterium tuberculosis	Morganella spp.	Klebsiella pneumoniae	Haemophilus influenzae	Group B Streptococcus	Group A Streptococcus	Escherichia coli	Other enterococci	Enterococcus faecium	Enterococcus faecalis	Enterobacter spp.	Citrobacter spp.	Acinetobacter baumannii
Uzbekistan	86	8.6	12.8	0	0.7	0	0.2	0	7.5	1.8	5.1	0.1	13.6	0.3	1.7	0.2	16.2	1.8	4.5	1.4	2.5	0.4	6.4
United Kingdom	26	1.7	4.6	0	0.1	0.1	0	0	1.4	0.7	0	0	2.7	0.2	0.2	0.1	9.1	0.2	2.5	0.7	0.6	0.1	1
Ukraine	46.8	4.3	2.3	0	0.4	0	0.1	0	4	1	4.6	0	6.1	0.1	0.8	0.2	10.1	1	3.2	2.1	1.8	0.4	4.1
Turkmenistan	68.6	7	10.2	0	0.6	0	0.2	0	5.8	1.4	4.4	0	10.2	0.3	1.2	0.1	13	1.4	3.7	1.2	2.1	0.4	5.2
Turkey	40.2	4.6	8.9	0	0.3	0	0.1	0	3	0.7	0.2	0	5.8	0.1	1.2	0.1	7.2	0.8	2.4	0.4	1	0.1	3.2
Tajikistan	86.8	11.4	10	0.2	1	0	0.3	0	6.7	2	4.7	0.1	11.2	0.2	1.3	0.2	13.3	2.3	3.3	1.7	4.4	0.9	11.9
Switzerland	13.3	0.7	1.9	0	0	0	0	0	0.7	0.5	0	0	0.9	0.1	0.4	0.1	4.9	0.1	1.7	0.1	0.3	0.1	0.8
Sweden	11.8	0.5	0.9	0	0.1	0	0	0	0.6	0.6	0.1	0	1	0	0.4	0.1	4.4	0.1	1.7	0.2	0.3	0	0.8
Spain	24.6	1.4	4.8	0	0	0	0	0	1.9	0.7	0	0	2.2	0.1	0.3	0.2	8	0.2	2.1	0.9	0.5	0.1	1
Slovenia	20.9	1.5	2.5	0	0.1	0	0	0	1.6	0.6	0	0	2.4	0	0.3	0.2	6.2	0.1	2.3	1.1	0.7	0.1	1.2
Slovakia	39.2	2.5	7.5	0	0.1	0	0	0	3.9	0.8	0	0	5.9	0.1	0.6	0.2	10.6	0.4	2.9	0.9	1	0.1	1.5
Serbia	47.3	3.5	7.9	0	0.5	0	0.1	0	4.1	1.1	0	0	6.6	0.1	0.7	0.4	11.8	0.8	3.1	1.5	1.7	0.3	2.9
San Marino	21.5	1.5	4.2	0	0.1	0	0	0	1.2	0.5	0	0	2.2	0.1	0.5	0.1	6.7	0.1	2	0.4	0.5	0.1	1.3
Russian Federation	51.4	4.3	5.2	0	0.4	0	0.1	0	5.1	1.1	2.4	0	7.5	0	1.1	0.1	14.3	0.8	3.4	2	1.6	0.1	1.7
Romania	47.5	4.1	9.1	0	0.5	0	0.1	0	5	0.9	0.5	0	6.6	0.2	0.8	0.3	11.1	0.7	3.1	2	0.8	0.2	1.8
Republic of Moldova	53.1	3.9	6.6	0	0.4	0	0.2	0	4.4	1.1	2.6	0	8	0.1	0.7	0.1	14.2	0.9	3.8	2.3	1.5	0.2	2
Portugal	35.1	2.2	9.4	0	0	0	0	0	2.5	0.6	0.1	0	4.3	0.1	0.8	0.1	9.3	0.2	2.3	1.1	0.7	0.1	1.2
Poland	35.1	3	5.2	0	0	0	0	0	2.9	0.9	0.1	0	5.2	0	0.7	0	8.9	0.5	2.6	1.5	1.5	0.1	1.9
Norway	16.4	0.7	2.2	0	0	0	0	0	0.7	0.6	0.1	0	1.8	0.1	0.5	0.1	6.1	0.1	1.6	0.4	0.4	0	0.9
North Macedonia	46.1	3	5.7	0	0.4	0	0	0	3.9	1	0.1	0	6.3	0.1	0.7	0.2	11.5	1.1	2.8	1.2	2.1	0.5	5.5
Netherlands	20.5	1	3.6	0	0	0	0	0	0.8	0.6	0	0	1.7	0	0.6	0.1	7.6	0.2	2.3	0.5	0.5	0	1
Montenegro	34.1	2.2	4.2	0	0.2	0	0	0	2.7	0.8	0	0	4.7	0	0.6	0.2	8.1	0.9	2.3	1.1	1.5	0.4	4.2
Monaco	27.3	2	6.3	0	0.1	0	0	0	1.6	0.5	0.1	0	2.5	0.1	0.8	0.1	8.4	0.1	2.4	0.4	0.5	0.1	1.2
Malta	23.3	2.2	5.3	0	0	0	0	0	1.4	0.5	0	0	2.6	0.1	0.4	0.2	5.8	0.2	1.5	0.4	0.5	0.1	1.8
Luxembourg	19.5	0.9	3.7	0	0	0	0	0	1.2	0.5	0	0	2	0.1	0.4	0.1	6.5	0.1	2.1	0.4	0.4	0.1	0.9
Lithuania	33.7	2.2	3.3	0	0.2	0	0.1	0	2.4	0.9	1.6	0	5.8	0	0.7	0.3	10.1	0.5	2.5	0.2	0.9	0.2	1.7
Latvia	31.5	1.8	3	0	0.1	0	0.1	0	2.9	0.9	0.6	0	4.4	0	0.8	0.1	9.3	0.5	2.6	1.8	0.6	0.1	1.8
Kyrgyzstan	56	4.2	7.7	0	0.6	0	0.1	0	5	1.2	5	0	8.4	0.1	1	0.1	10.5	1.1	3.1	1.5	1.9	0.3	4.1
Kazakhstan	59.1	4.8	7.8	0	0.7	0	0.1	0	6.2	1.2	2.4	0	9.5	0.1	1.1	0.2	11.2	1.3	3.7	1.7	2.1	0.3	4.9
Italy	22.6	0.8	5	0	0	0	0	0	1.5	0.5	0	0	2.7	0	0.5	0.1	7	0.2	1.8	0.9	0.4	0.1	0.9
Israel	30.4	1.6	7.1	0	0	0	0	0	1.8	0.9	0	0	3.2	0.1	0.5	0.1	9.8	0.3	1.8	1	0.5	0.1	1.2
Ireland	23.3	2.3	4.8	0	0	0	0	0	1	0.6	0	0	2.5	0.1	0.4	0.1	7.3	0.1	1.9	0.6	0.8	0.1	1
Iceland	14	0.9	2.1	0	0	0	0	0	0.8	0.4	0	0	1.7	0.1	0.5	0.1	4.5	0.1	1.4	0.2	0.4	0	0.8
Hungary	34.6	1.5	5.8	0	0.1	0	0	0	2.7	0.8	0	0	4.1	0	0.7	0.1	10.9	0.5	3.3	1.5	0.8	0.1	1.5
Greece	28.4	1.7	7.2	0	0	0	0	0	2.7	0.6	0.1	0	3.8	0.2	0.2	0.2	7.1	0.3	1.9	0.8	0.6	0.1	1.1
Germany	22.4	0.8	4	0	0.1	0	0	0	1.6	0.6	0	0	2.1	0.1	0.5	0.1	7.9	0.1	2.4	0.6	0.6	0.1	0.8
Georgia	48.2	3.8	5.5	0	0.7	0	0.1	0	4.5	1	1.8	0	8	0.1	0.9	0.2	9.2	1.1	3	1.4	1.9	0.3	4.8
France	20.7	2.1	3.4	0	0.1	0	0	0	1.4	0.4	0.1	0	2.3	0.1	0.2	0.1	6.7	0.1	1.8	0.3	0.6	0.1	1
Finland	13.1	0.7	1	0	0.1	0	0	0	0.8	0.6	0	0	1.4	0	0.4	0.1	4.2	0.1	2	0.4	0.4	0.1	0.9
Estonia	23.1	1.5	2	0	0.1	0	0	0	1.1	0.7	0.5	0	3.1	0	0.6	0.1	5.4	0.3	2.2	1.3	1	0.3	3
Denmark	20.2	0.7	1.9	0	0	0	0	0	0.6	0.7	0	0	2.7	0.1	0.5	0	7.4	0.5	2.3	0.8	0.7	0.1	1.1
Czechia	25.4	1.4	3.4	0	0.1	0	0	0	2.1	0.7	0	0	3.5	0	0.6	0.3	6.2	0.3	2.2	1.2	1	0.2	2.3
Cyprus	32.5	1.5	6.7	0	0.1	0	0	0	1.8	0.8	0.1	0	3.6	0.1	0.6	0.1	11.2	0.5	2.4	1	0.6	0.1	1.3
Croatia	28.9	1.7	3.9	0	0.1	0	0	0	2.4	0.7	0	0	3.2	0	0.5	0.1	7.7	0.4	2.2	1.1	1.4	0.2	3.3
Bulgaria	46.7	2.8	4.3	0	0.5	0	0.1	0	3.6	1.5	0.1	0	7.5	0.1	0.7	0.3	14.4	0.8	3.2	2.2	1.7	0.3	2.6
Bosnia and Herzegovina	35.3	2.6	4.1	0	0.3	0	0	0	3.2	0.9	0	0	5	0.1	0.7	0.2	7.5	0.9	2.4	1.4	1.7	0.5	3.8
Belgium	25.9	1.6	5.4	0	0.1	0.1	0	0	1.7	0.8	0	0	2.4	0.2	0.5	0.1	8.4	0.2	2.3	0.2	0.6	0.1	1.1
Belarus	34.8	2.5	2.4	0	0.2	0	0	0	3.2	0.8	1.5	0	5	0	0.9	0.1	8.8	0.7	2.5	1.7	1.4	0.2	2.8
Azerbaijan	75	8.3	11.5	0	0.7	0	0.2	0	6.6	1.6	3	0.1	12	0.3	2	0.2	14	1.7	3.8	1.1	2.3	0.4	5.4
Austria	16.7	0.6	3.1	0	0	0	0	0	1	0.6	0	0	1.3	0	0.4	0	5.6	0.1	1.9	0.2	0.6	0.1	1.1
Armenia	52.1	3.5	5.8	0	0.6	0	0.1	0	5	1.5	0.9	0.1	8.5	0.1	1.1	0.2	12.7	1.6	3.5	1.4	1.8	0.3	3.5
Andorra	21.5	1.2	3.3	0	0.1	0	0	0	1.1	0.6	0	0	2.2	0.1	0.5	0.1	8.2	0.1	2.2	0.4	0.4	0.1	1
Albania	33.5	3.1	5.9	0	0.1	0	0.1	0	2.7	0.6	0	0	4.8	0.1	0.7	0.2	9.9	0.5	2.1	0.7	0.7	0.1	1.4

Rate
- >=75
- 50 – <75
- 25 – <50
- 15 – <25
- 10 – <15
- 7 – <10
- 5 – <7
- 3 – <5
- 1 – <3
- < 1

Supplementary Figure 5. Heatmap representing age-standardised DALYs per 100,000 person years associated with antimicrobial resistance (AMR) by pathogen and country for the WHO European Region in 2019.

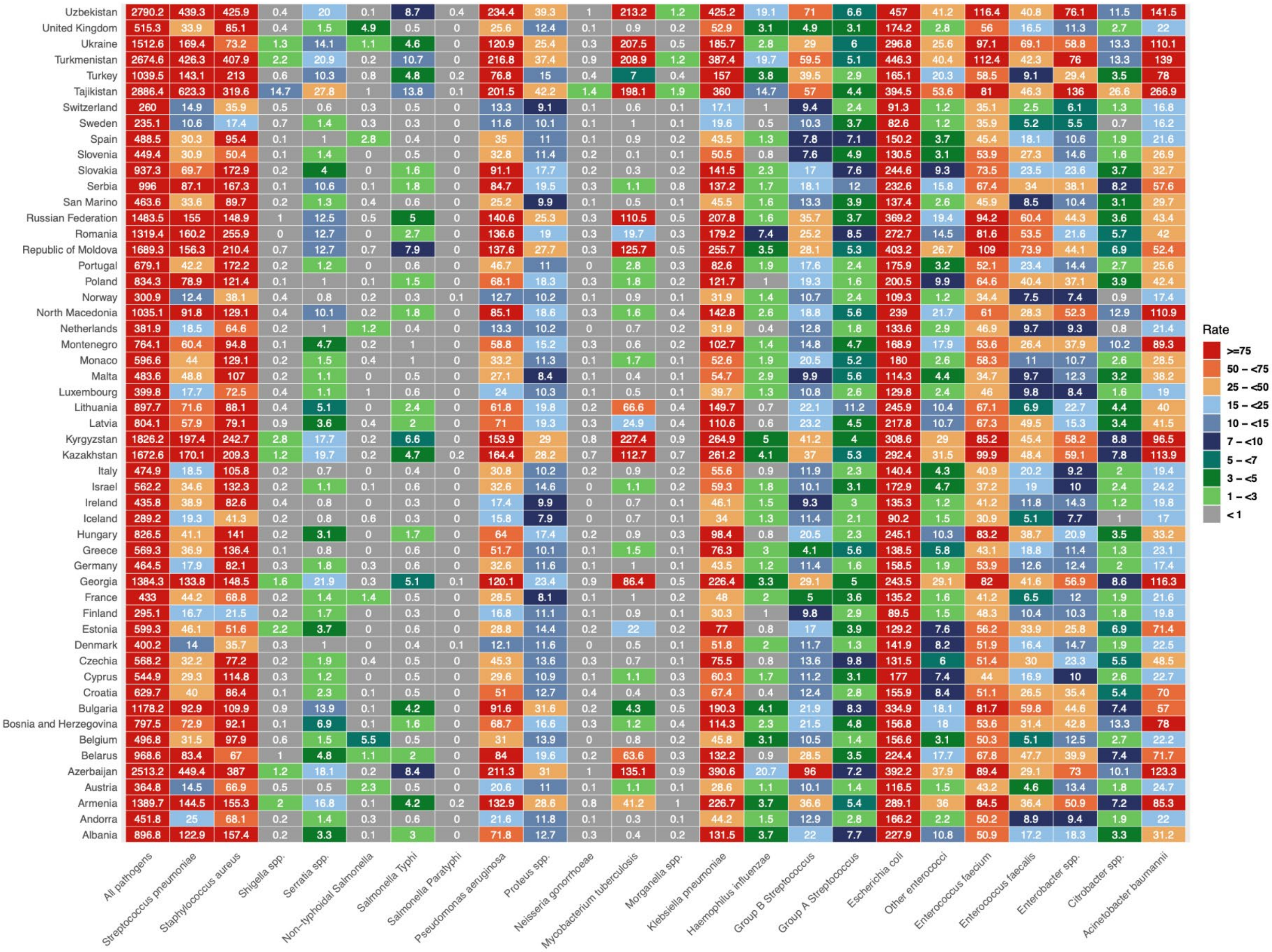

1135 **Supplementary Figure 6.** Heatmap representing age-standardised DALYs per 100,000 person years attributable to
1136 antimicrobial resistance (AMR) by pathogen and country for the WHO European Region in 2019.

	All pathogens	Streptococcus pneumoniae	Staphylococcus aureus	Shigella spp.	Serratia spp.	Non−typhoidal Salmonella	Salmonella Typhi	Salmonella Paratyphi	Pseudomonas aeruginosa	Proteus spp.	Neisseria gonorrhoeae	Mycobacterium tuberculosis	Morganella spp.	Klebsiella pneumoniae	Haemophilus influenzae	Group B Streptococcus	Group A Streptococcus	Escherichia coli	Other enterococci	Enterococcus faecium	Enterococcus faecalis	Enterobacter spp.	Citrobacter spp.	Acinetobacter baumannii
Uzbekistan	702.2	89.2	80.7	0.1	5.1	0	1.7	0.1	60.6	6.2	0.1	96.2	0.3	116.1	4.5	11.2	0.6	115.6	8.4	29.1	11.2	16.7	3.4	45.1
United Kingdom	111	6.3	18.2	0	0.4	0.5	0.1	0	6.1	1.1	0	0.2	0	10.9	0.6	0.8	0.3	36.1	0.7	14.6	4.5	2.8	0.8	6
Ukraine	409.1	32.5	14.7	0.2	3.5	0.2	0.8	0	30.9	2.8	0	93.7	0.1	49.6	0.6	3.6	0.6	73.2	5.6	25.7	18.2	13.1	3.9	35.4
Turkmenistan	675	93.4	78.9	0.4	5.2	0	2	0	56.1	5.8	0.1	86.7	0.3	100.6	4.6	9.7	0.5	115.1	8.2	30.9	11.6	16.5	3.9	44.4
Turkey	284.9	36.8	68.4	0.1	2.7	0.1	1	0	19	1.8	0	1.9	0.1	49.3	0.8	4.8	0.3	41.7	5	14.7	2.7	7.6	1.1	25.1
Tajikistan	773.6	143	84.4	2.9	7.1	0.2	2.7	0	53.8	6.9	0.1	86.7	0.5	99.7	3.6	8	0.4	108.2	10.9	19.9	12.5	28.8	7.9	85.2
Switzerland	55.6	3.1	8.6	0.1	0.2	0	0.1	0	3.2	0.8	0	0.2	0	3.7	0.2	1.1	0.2	18.7	0.3	7.8	0.8	1.2	0.4	4.8
Sweden	49.3	2	3.7	0.1	0.4	0	0.1	0	2.8	0.7	0	0.3	0	4.2	0.1	1.3	0.3	17.7	0.3	7.9	1.4	1.4	0.2	4.4
Spain	110.7	7.4	21.9	0	0.3	0.3	0.1	0	8.4	1	0	0.2	0.1	10.7	0.3	1	0.7	33.1	0.8	10.1	4.7	2.6	0.6	6.5
Slovenia	101.7	5.2	13.3	0	0.4	0	0.1	0	8	1.2	0	0	0	11.8	0.2	1	0.5	28.9	0.7	11.9	7.1	3	0.5	8
Slovakia	222	13.9	38.1	0	1	0	0.3	0	22.5	2.5	0	0.1	0.1	35.9	0.6	2.2	0.7	59.4	2	20	6.1	5.3	1.1	10.1
Serbia	249.3	22.6	34.1	0	2.8	0	0.4	0	21.7	3.5	0	0.4	0.2	42.7	0.4	2.2	1.1	56.7	3.3	17	8.9	10.7	2.3	18.4
San Marino	105.3	5.7	21.6	0	0.4	0.1	0.1	0	6.2	1.3	0	0.1	0	9.4	0.3	2.2	0.4	31.2	0.6	11.2	2.3	2.4	0.9	9
Russian Federation	403.4	28.3	39.9	0.1	3.1	0.1	1	0	36.4	2.4	0	48.1	0.1	73.8	0.4	4.3	0.4	98.3	4.2	21.5	15.9	10	1	13.9
Romania	341.8	39.8	65.5	0	3.2	0	0.5	0	34.2	3.3	0	8.2	0.1	57.9	2	3	0.8	62.4	3	24	14	4.6	1.7	13.5
Republic of Moldova	449.7	28.2	63.5	0.1	3.2	0.1	1.5	0	34.7	3.5	0	57.4	0.1	69	0.8	3.6	0.5	101.9	5.7	27.6	19.5	10	2	16.9
Portugal	159.7	9.4	39.8	0	0.3	0	0.1	0	11.8	1.3	0	0.8	0.1	23.3	0.4	2.1	0.2	38.6	0.7	12.4	6.1	3.5	0.8	7.8
Poland	194.3	16.3	24	0	0.2	0	0.3	0	16.8	2	0	0.5	0.1	32.9	0.2	2.3	0.2	45.1	2.1	17.5	10.6	8.5	1.1	13.5
Norway	72.2	2.5	11.1	0.1	0.2	0	0.1	0	3	0.8	0	0.3	0	6.4	0.3	1.3	0.2	29.1	0.3	7.7	2	1.6	0.3	4.9
North Macedonia	289.6	20.6	40.4	0.1	2.5	0	0.4	0	21.5	2.8	0	0.8	0.1	44.2	0.5	2.6	0.5	73.1	4.7	15.7	7.5	12.2	3.8	35.5
Netherlands	72.8	3.5	7.7	0	0.3	0.1	0.1	0	3	0.9	0	0.2	0	6.4	0.1	1.6	0.2	26.9	0.6	10.2	2.6	2.2	0.2	6
Montenegro	198.4	12.8	25.5	0	1.2	0	0.2	0	14.3	2.6	0	0.2	0.1	29.7	0.3	2.4	0.5	43.9	3.9	14.3	6.9	8.2	3	28.4
Monaco	126.2	7.6	21.3	0	0.4	0	0.2	0	8.2	1.5	0	0.5	0	10.7	0.4	3.7	0.5	40.7	0.6	15	3	2.5	0.7	8.7
Malta	115.8	10	25.1	0	0.3	0	0.1	0	6.8	1.2	0	0.1	0	17.2	0.6	1.3	0.5	26.2	1	7.8	2.7	2.9	0.9	11.1
Luxembourg	85.7	3.7	12.8	0	0.3	0.1	0.1	0	5.6	1.1	0	0.1	0	10.1	0.3	1.6	0.3	28.3	0.5	10.2	2.7	1.9	0.5	5.4
Lithuania	211.2	13.5	14.4	0.1	1.4	0	0.5	0	14.9	2.4	0	29.4	0.1	36.2	0.2	2.6	1.1	52.4	2.2	19	2.2	4.7	1.3	12.8
Latvia	186	11.3	12.6	0.1	0.9	0	0.4	0	17.9	2.4	0	8.8	0.1	26.4	0.2	2.7	0.4	50.2	2.2	18.5	13.5	3	1	13.2
Kyrgyzstan	489	37.8	66.8	0.5	4.6	0	1.3	0	39.2	4.3	0.1	90.8	0.2	71.8	1.2	5.9	0.4	79.8	5.9	20.6	12	12.6	2.6	30.6
Kazakhstan	417	33.8	50.4	0.2	4.9	0	0.9	0	41.7	3.8	0.1	46.5	0.2	69.3	0.9	4.9	0.5	63.5	6.6	24.4	12.7	13.1	2.3	36.2
Italy	115.8	3.6	24.3	0	0.2	0	0.1	0	7.5	1.9	0	0.3	0	16.9	0.2	1.4	0.2	33.7	0.9	10.4	5.3	2.1	0.6	6.2
Israel	134.2	7.3	33.2	0	0.3	0	0.1	0	7.5	1.9	0	0.4	0.1	15.4	0.4	1.4	0.3	39.5	1.1	9.5	5	2.5	0.7	7.6
Ireland	93.6	9.3	14.3	0.1	0.2	0	0.1	0	4.2	0.7	0	0.2	0	9.2	0.3	1.2	0.3	29.2	0.3	11.9	3.1	3.2	0.4	5.4
Iceland	61.3	3.9	8.3	0	0.2	0.1	0.1	0	3.7	0.9	0	0.1	0	7.1	0.3	1.8	0.2	19.3	0.3	7	1.4	1.7	0.3	4.8
Hungary	196	8.7	32.3	0	0.8	0	0.3	0	16	2.4	0	0.2	0.1	24.8	0.2	2.4	0.2	56.6	2.1	23.1	10	4.4	1	10.3
Greece	144.3	7.1	33.5	0	0.2	0	0.1	0	13	1.1	0	0.5	0	26.3	0.7	0.6	0.5	32	1.4	11.6	5.2	2.6	0.4	7.5
Germany	98	3.7	13.7	0	0.5	0	0.1	0	7.8	1	0	0.3	0	9.5	0.2	1.4	0.2	33.9	0.5	13.6	3.3	2.7	0.6	4.9
Georgia	378.9	29.1	32.7	0.2	5.4	0	1	0	30.4	3.2	0.1	37.8	0.1	73.8	0.7	3.7	0.5	71.1	6	19.8	11	12.8	2.5	36.8
France	94.5	10.1	14.4	0	0.4	0.2	0.1	0	7.1	0.8	0	0.3	0	11.9	0.4	0.7	0.3	28	0.4	9.1	1.8	2.6	0.5	5.5
Finland	62.8	3.2	5.2	0	0.5	0	0.1	0	3.9	1	0	0.4	0	5.8	0.2	1.2	0.3	18.7	0.4	10.6	2.9	2.3	0.5	5.6
Estonia	131.1	7.9	7.7	0.3	1	0	0.1	0	6.8	1.2	0	9.4	0	15.4	0.2	2	0.4	26.1	1.6	12.7	8.8	5.7	2.1	21.7
Denmark	80.5	3.3	6.5	0	0.3	0	0.1	0	2.9	0.9	0	0.2	0	8.9	0.4	1.4	0.1	28.1	2	12.3	4.2	2.7	0.6	5.5
Czechia	122.7	5.6	13.8	0	0.5	0.1	0.1	0	10.9	1	0	0.2	0	19.1	0.2	1.8	0.9	28.6	1.3	12.7	7.7	4.6	1.6	11.8
Cyprus	139.7	7.5	29.2	0	0.3	0	0.1	0	7.2	1.4	0	0.2	0.1	18.3	0.4	1.4	0.3	43.3	1.6	13.6	4.5	2.4	0.7	7.2
Croatia	151.6	8.3	20.4	0	0.6	0	0.1	0	12.4	1.6	0	0.1	0.1	18.1	0.1	1.5	0.3	33	1.8	13.7	6.9	8.4	1.6	22.6
Bulgaria	300.2	20.5	24.4	0.1	3.8	0	0.8	0	22.6	8.3	0	1.4	0.1	59.4	1	2.8	0.8	83.1	4	20.7	15.5	10.5	2.2	18.2
Bosnia and Herzegovina	202.5	17.5	21	0	1.7	0	0.3	0	17.4	2.4	0	0.5	0.1	34.5	0.5	2.7	0.5	39.3	4	14	8.2	9.6	3.7	24.5
Belgium	103.2	6.2	17.5	0.1	0.4	0.5	0.1	0	7.6	1.1	0	0.2	0.1	11.2	0.6	1.4	0.1	33.1	0.7	11.3	1.5	2.9	0.8	5.8
Belarus	251.6	14.4	16.1	0.1	1.2	0.2	0.4	0	20.7	2	0	30.3	0.1	38.6	0.2	3.3	0.3	57.4	3.6	16.6	12.5	8.6	2.2	22.7
Azerbaijan	610.6	92.1	71.7	0.2	4.6	0	1.6	0	54.2	5	0.1	59.7	0.2	103.6	4.9	16.3	0.7	99.5	7.7	22.3	8	15.9	2.9	39.4
Austria	73.7	2.6	10	0.1	0.1	0.2	0.1	0	5	1	0	0.4	0	5.8	0.2	1.2	0.1	24.5	0.3	9.8	1.3	3	0.5	7.4
Armenia	345.7	27.9	37.7	0.3	4.2	0	0.8	0	34.3	3.9	0.1	16.1	0.3	61.6	0.8	5.4	0.5	73.6	7.5	20.4	9.7	11.4	2.1	27.2
Andorra	98.8	5.4	11.3	0	0.4	0	0.1	0	5.1	1.3	0	0.1	0	9.1	0.3	2	0.3	39.2	0.5	12.2	2.4	2.1	0.6	6.3
Albania	229.1	26.4	43.5	0	0.8	0	0.6	0	18.6	2.2	0	0.1	0.1	36.5	0.8	3.7	0.7	59.4	2.4	13.6	4.6	4.2	1	9.9

Rate
>=75
50 – <75
25 – <50
15 – <25
10 – <15
7 – <10
5 – <7
3 – <5
1 – <3
< 1

Supplementary Figure 7. Deaths attributable to antimicrobial resistance (AMR) in accordance with the infectious syndrome.

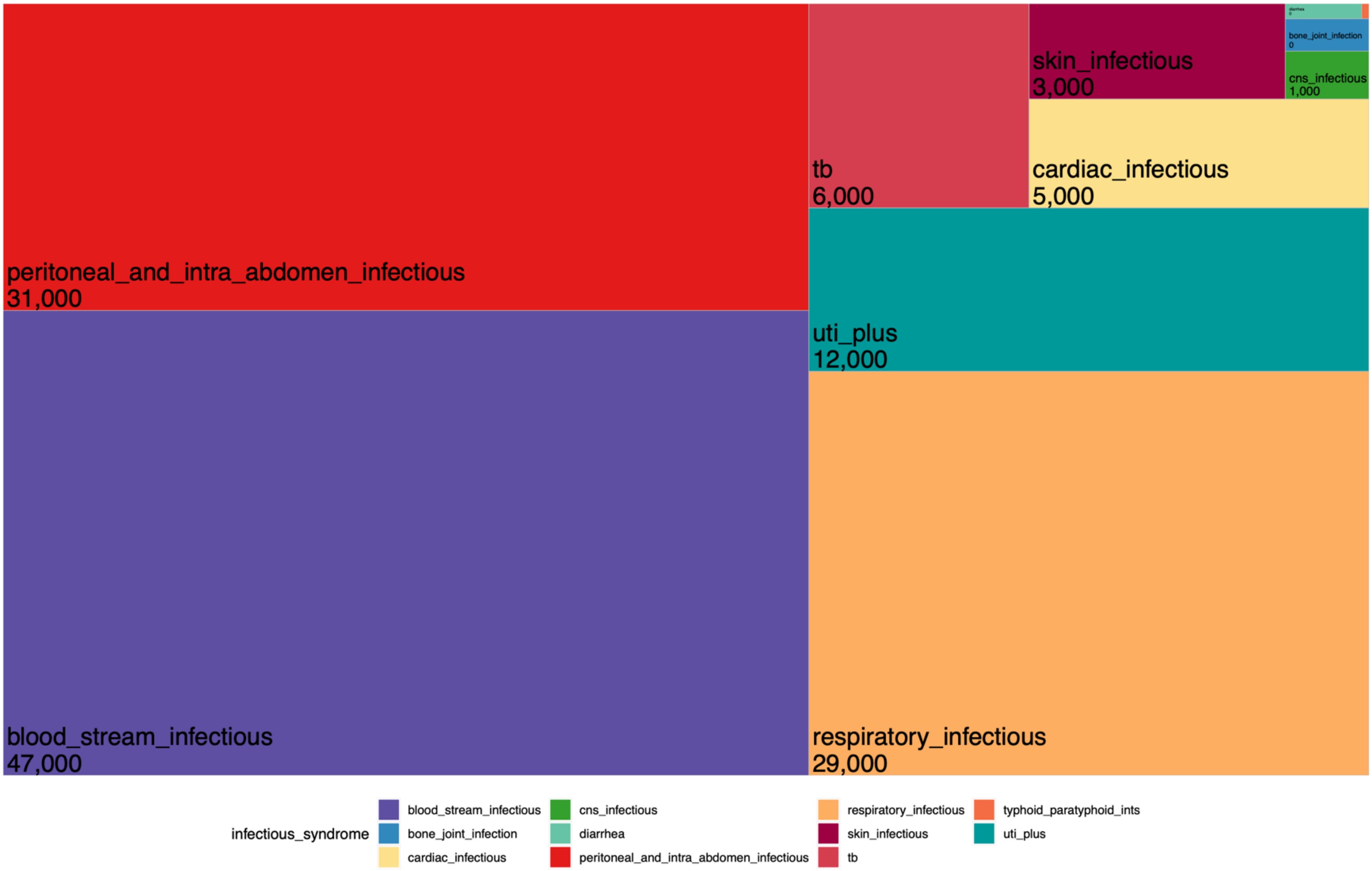

Supplementary Figure 8. Deaths associated with antimicrobial resistance (AMR) in accordance with the infectious syndrome.

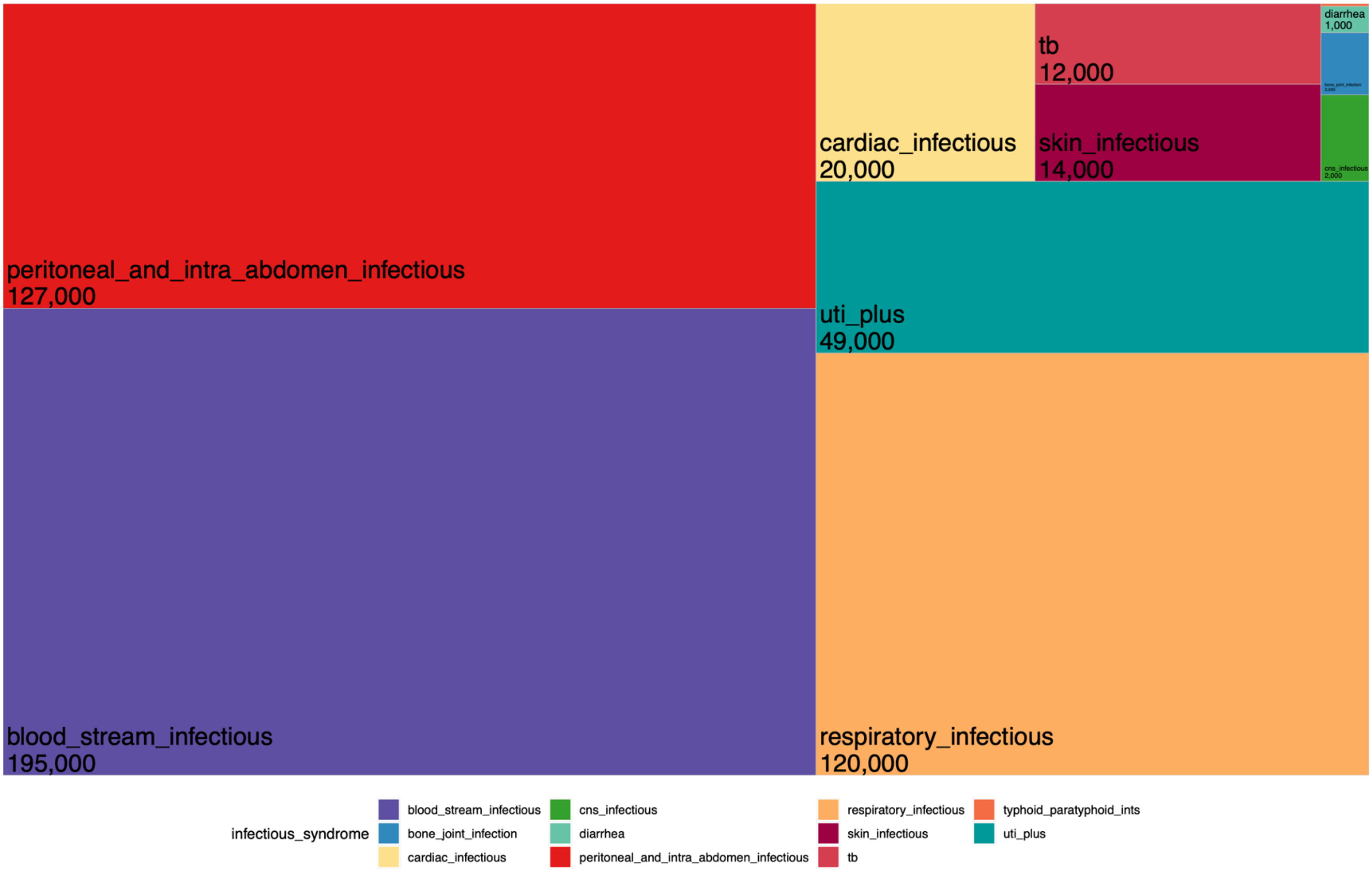

Supplementary Figure 9. Crude mortality rates associated with AMR by Socio-demographic Index (SDI) for countries in the WHO European Region in 2019. Note: highlighted subregions are in accordance with GBD regions. Abbreviations: ALB=Albania. AND=Andorra. ARM=Armenia. AUT=Austria. AZE=Azerbaijan. BLR=Belarus. BEL=Belgium. BIH=Bosnia and Herzegovina. BGR=Bulgaria. HRV=Croatia. CYP=Cyprus. CZE=Czechia (Czech Republic). DNK=Denmark. EST=Estonia. FIN=Finland. FRA=France. GEO=Georgia. DEU=Germany. GRC=Greece. HUN=Hungary. ISL=Iceland. IRL=Ireland. ISR=Israel. ITA=Italy. KAZ=Kazakhstan. KGZ=Kyrgyzstan. LVA=Latvia. LTU=Lithuania. LUX=Luxembourg. MLT=Malta. MCO=Monaco. MNE=Montenegro. NLD=The Netherlands. MKD=North Macedonia. NOR=Norway. POL=Poland. PRT=Portugal. MDA=Republic of Moldova. ROU=Romania. RUS=Russia. SMR=San Marino. SRB=Serbia. SVK=Slovakia. SVN=Slovenia. ESP=Spain. SWE=Sweden. CHE=Switzerland. TJK=Tajikistan. TUR=Turkey. TKM=Turkmenistan. UKR=Ukraine. GBR=United Kingdom. UZB=Uzbekistan.

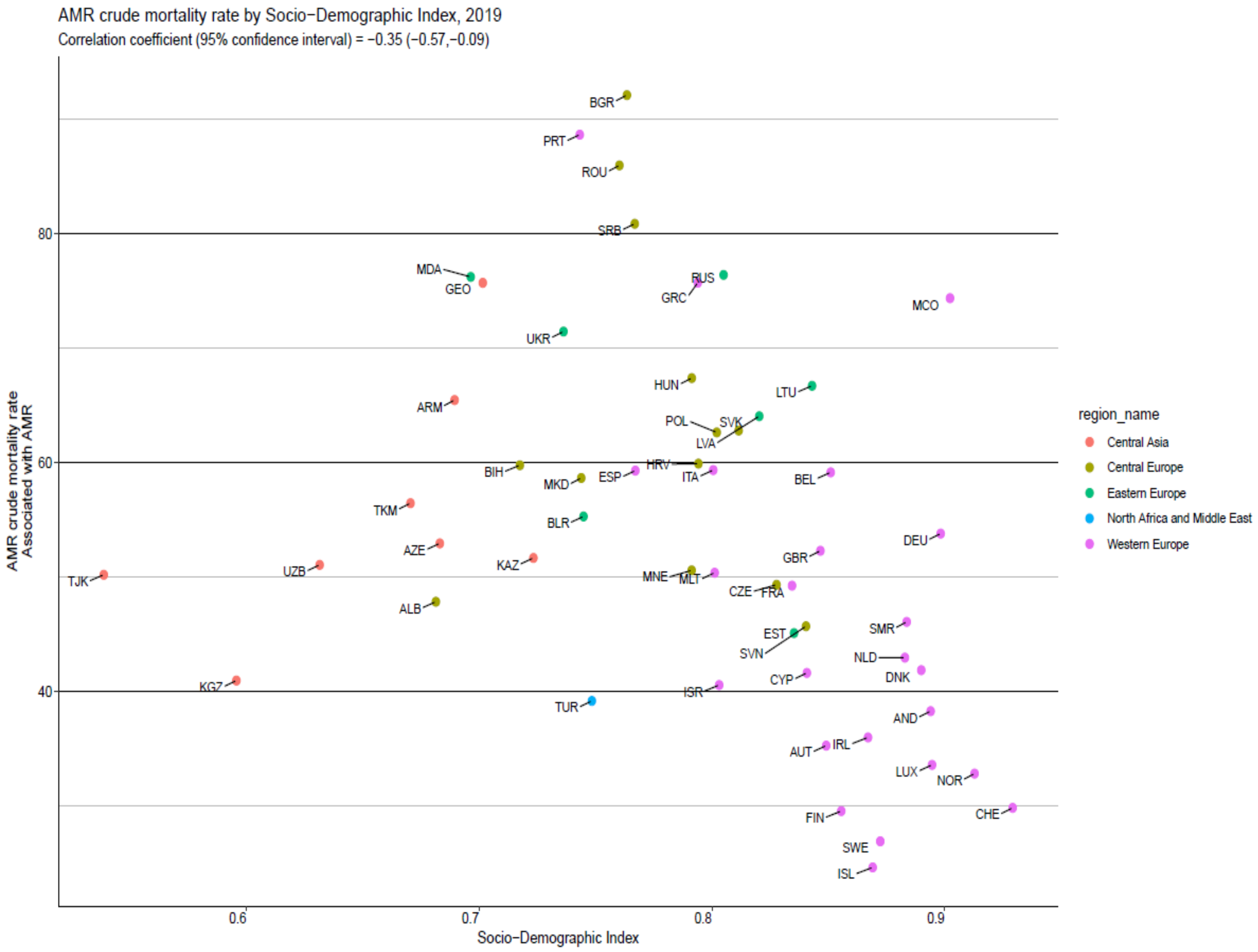

Supplementary Figure 10. Crude mortality rates attributable to AMR by Socio-demographic Index (SDI) for countries in the WHO European Region in 2019. Note: highlighted subregions are in accordance with GBD regions. Abbreviations: ALB=Albania. AND=Andorra. ARM=Armenia. AUT=Austria. AZE=Azerbaijan. BLR=Belarus. BEL=Belgium. BIH=Bosnia and Herzegovina. BGR=Bulgaria. HRV=Croatia. CYP=Cyprus. CZE=Czechia (Czech Republic). DNK=Denmark. EST=Estonia. FIN=Finland. FRA=France. GEO=Georgia. DEU=Germany. GRC=Greece. HUN=Hungary. ISL=Iceland. IRL=Ireland. ISR=Israel. ITA=Italy. KAZ=Kazakhstan. KGZ=Kyrgyzstan. LVA=Latvia. LTU=Lithuania. LUX=Luxembourg. MLT=Malta. MCO=Monaco. MNE=Montenegro. NLD=The Netherlands. MKD=North Macedonia. NOR=Norway. POL=Poland. PRT=Portugal. MDA=Republic of Moldova. ROU=Romania. RUS=Russia. SMR=San Marino. SRB=Serbia. SVK=Slovakia. SVN=Slovenia. ESP=Spain. SWE=Sweden. CHE=Switzerland. TJK=Tajikistan. TUR=Turkey. TKM=Turkmenistan. UKR=Ukraine. GBR=United Kingdom. UZB=Uzbekistan.

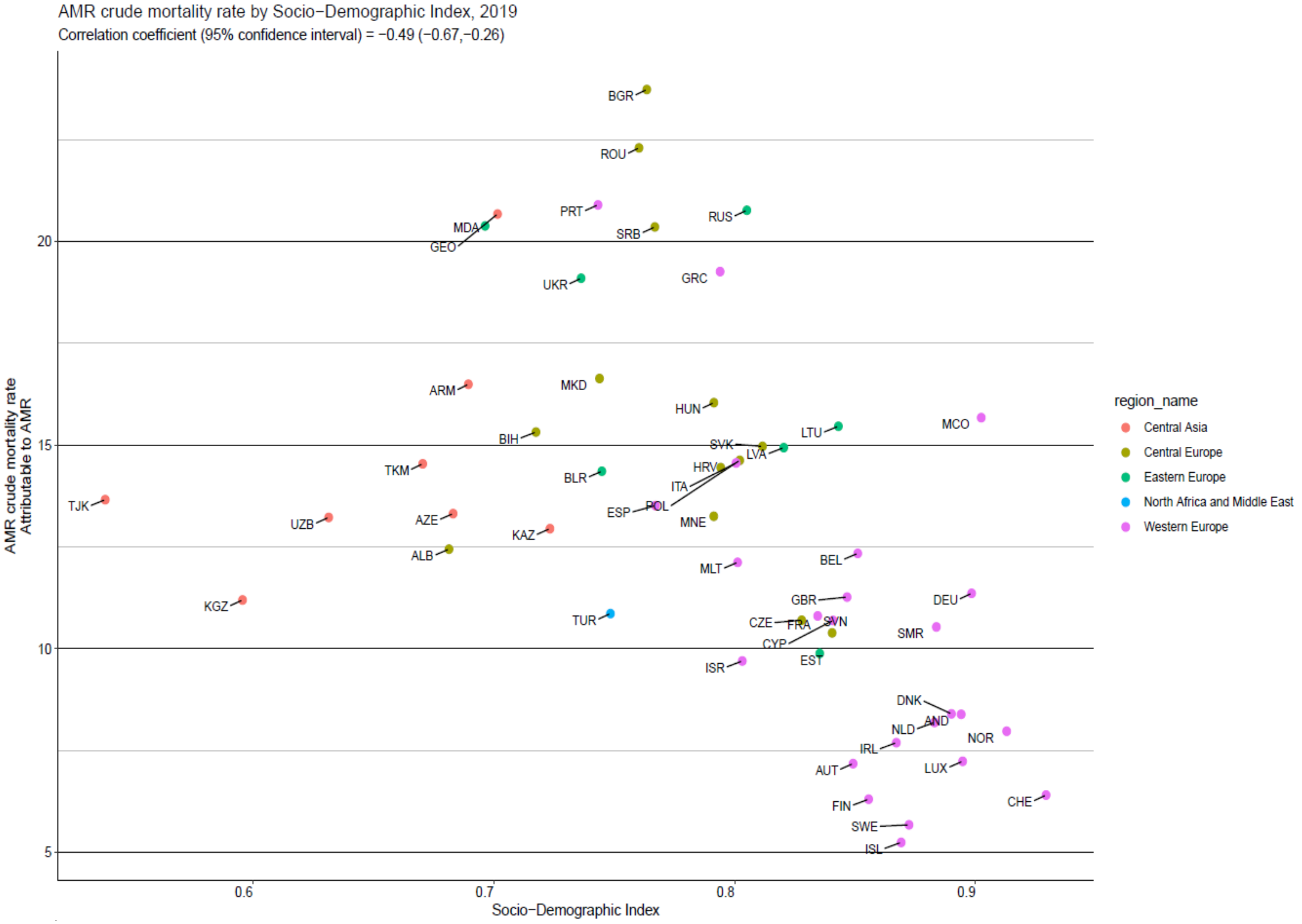

Supplementary Figures 11-36. Crude mortality rates attributable to and associated with AMR for analysed antimicrobial agents / antimicrobial groups by DDDs per 1000 people for countries in the WHO European Region in 2019. Note: highlighted subregions are in accordance with GBD regions. Abbreviations: ALB=Albania. AND=Andorra. ARM=Armenia. AUT=Austria. AZE=Azerbaijan. BLR=Belarus. BEL=Belgium. BIH=Bosnia and Herzegovina. BGR=Bulgaria. HRV=Croatia. CYP=Cyprus. CZE=Czechia (Czech Republic). DNK=Denmark. EST=Estonia. FIN=Finland. FRA=France. GEO=Georgia. DEU=Germany. GRC=Greece. HUN=Hungary. ISL=Iceland. IRL=Ireland. ISR=Israel. ITA=Italy. KAZ=Kazakhstan. KGZ=Kyrgyzstan. LVA=Latvia. LTU=Lithuania. LUX=Luxembourg. MLT=Malta. MCO=Monaco. MNE=Montenegro. NLD=The Netherlands. MKD=North Macedonia. NOR=Norway. POL=Poland. PRT=Portugal. MDA=Republic of Moldova. ROU=Romania. RUS=Russia. SMR=San Marino. SRB=Serbia. SVK=Slovakia. SVN=Slovenia. ESP=Spain. SWE=Sweden. CHE=Switzerland. TJK=Tajikistan. TUR=Turkey. TKM=Turkmenistan. UKR=Ukraine. GBR=United Kingdom. UZB=Uzbekistan. ***(Figures available in separate PDF files)***

Supplementary Figures 37-90. Heatmaps representing death counts attributable to antimicrobial resistance (AMR) by pathogen–drug combination for every country in the WHO European Region in 2019. Abbreviations: 3GC=third-generation cephalosporins. 4GC=fourth-generation cephalosporins. Anti-pseudomonal=anti-pseudomonal penicillin or beta-lactamase inhibitors. BL-BLI=β-lactam or β-lactamase inhibitors. MDR=multidrug resistance. Mono INH=isoniazid mono-resistance. Mono RIF=rifampicin mono-resistance. NA=not applicable. Resistance to 1+=resistance to one or more drug. S Paratyphi=*Salmonella enterica* serotype Paratyphi. S Typhi=*Salmonella enterica* serotype Typhi. TMP-SMX=trimethoprim-sulfamethoxazole. XDR=extensive drug resistance. Countries included: Albania, Andorra, Armenia, Austria, Azerbaijan, Belarus, Belgium, Bosnia and Herzegovina, Bulgaria, Croatia, Cyprus, Czechia (Czech Republic), Denmark, Estonia, Finland, France, Georgia, Germany, Greece, Hungary, Iceland, Ireland, Israel, Italy, Kazakhstan, Kyrgyzstan, Latvia, Lithuania, Luxembourg, Malta, Monaco, Montenegro. The Netherlands, North Macedonia, Norway, Poland, Portugal, Republic of Moldova, Romania, Russian Federation, San Marino, Serbia, Slovakia, Slovenia, Spain, Sweden, Switzerland, Tajikistan, Turkey, Turkmenistan, Ukraine, United Kingdom, Uzbekistan. ***(Figures available in separate PDF files)***

Supplementary Figures 91-144. Heatmaps representing death counts associated with antimicrobial resistance (AMR) by pathogen–drug combination for every country in the WHO European Region in 2019. Abbreviations: 3GC=third-generation cephalosporins. 4GC=fourth-generation cephalosporins. Anti-pseudomonal=anti-pseudomonal penicillin or beta-lactamase inhibitors. BL-BLI=β-lactam or β-lactamase inhibitors. MDR=multidrug resistance. Mono INH=isoniazid mono-resistance. Mono RIF=rifampicin mono-resistance. NA=not applicable. Resistance to 1+=resistance to one or more drug. S Paratyphi=*Salmonella enterica* serotype Paratyphi. S Typhi=*Salmonella enterica* serotype Typhi. TMP-SMX=trimethoprim-sulfamethoxazole. XDR=extensive drug resistance. Countries included: Albania, Andorra, Armenia, Austria, Azerbaijan, Belarus, Belgium, Bosnia and Herzegovina, Bulgaria, Croatia, Cyprus, Czechia (Czech Republic), Denmark, Estonia, Finland, France, Georgia, Germany, Greece, Hungary, Iceland, Ireland, Israel, Italy, Kazakhstan, Kyrgyzstan, Latvia, Lithuania, Luxembourg, Malta, Monaco, Montenegro. The Netherlands, North Macedonia, Norway, Poland, Portugal, Republic of Moldova, Romania, Russian Federation, San Marino, Serbia, Slovakia, Slovenia, Spain, Sweden, Switzerland, Tajikistan, Turkey, Turkmenistan, Ukraine, United Kingdom, Uzbekistan***. (Figures available in separate PDF files)***

Supplementary Figures 145-198. Heatmaps representing DALY counts attributable to antimicrobial resistance (AMR) by pathogen–drug combination for every country in the WHO European Region in 2019. Abbreviations: 3GC=third-generation cephalosporins. 4GC=fourth-generation cephalosporins. Anti-pseudomonal=anti-pseudomonal penicillin or beta-lactamase inhibitors. BL-BLI=β-lactam or β-lactamase inhibitors. MDR=multidrug resistance. Mono INH=isoniazid mono-resistance. Mono RIF=rifampicin mono-resistance. NA=not applicable. Resistance to 1+=resistance to one or more drug. S Paratyphi=*Salmonella enterica* serotype Paratyphi. S Typhi=*Salmonella enterica* serotype Typhi. TMP-SMX=trimethoprim-sulfamethoxazole. XDR=extensive drug

resistance. Countries included: Albania, Andorra, Armenia, Austria, Azerbaijan, Belarus, Belgium, Bosnia and Herzegovina, Bulgaria, Croatia, Cyprus, Czechia (Czech Republic), Denmark, Estonia, Finland, France, Georgia, Germany, Greece, Hungary, Iceland, Ireland, Israel, Italy, Kazakhstan, Kyrgyzstan, Latvia, Lithuania, Luxembourg, Malta, Monaco, Montenegro. The Netherlands, North Macedonia, Norway, Poland, Portugal, Republic of Moldova, Romania, Russian Federation, San Marino, Serbia, Slovakia, Slovenia, Spain, Sweden, Switzerland, Tajikistan, Turkey, Turkmenistan, Ukraine, United Kingdom, Uzbekistan. ***(Figures available in separate PDF files)***

Supplementary Figures 199-252. Heatmaps representing DALY counts associated with antimicrobial resistance (AMR) by pathogen–drug combination for every country in the WHO European Region in 2019. Abbreviations: 3GC=third-generation cephalosporins. 4GC=fourth-generation cephalosporins. Anti-pseudomonal=anti-pseudomonal penicillin or beta-lactamase inhibitors. BL-BLI=β-lactam or β-lactamase inhibitors. MDR=multidrug resistance. Mono INH=isoniazid mono-resistance. Mono RIF=rifampicin mono-resistance. NA=not applicable. Resistance to 1+=resistance to one or more drug. S Paratyphi=*Salmonella enterica* serotype Paratyphi. S Typhi=*Salmonella enterica* serotype Typhi. TMP-SMX=trimethoprim-sulfamethoxazole. XDR=extensive drug resistance. Countries included: Albania, Andorra, Armenia, Austria, Azerbaijan, Belarus, Belgium, Bosnia and Herzegovina, Bulgaria, Croatia, Cyprus, Czechia (Czech Republic), Denmark, Estonia, Finland, France, Georgia, Germany, Greece, Hungary, Iceland, Ireland, Israel, Italy, Kazakhstan, Kyrgyzstan, Latvia, Lithuania, Luxembourg, Malta, Monaco, Montenegro. The Netherlands, North Macedonia, Norway, Poland, Portugal, Republic of Moldova, Romania, Russian Federation, San Marino, Serbia, Slovakia, Slovenia, Spain, Sweden, Switzerland, Tajikistan, Turkey, Turkmenistan, Ukraine, United Kingdom, Uzbekistan. ***(Figures available in separate PDF files)***

Section 5: GATHER Compliance: Guidelines for Accurate and Transparent Health Estimates Reporting

This study complies with GATHER recommendations. We have documented the steps in our analytical procedures and detailed the data sources used. The GATHER recommendations can be found on the GATHER website.

GATHER
Guidelines for Accurate and Transparent Health Estimates Reporting

Checklist of information that should be included in new reports of global health estimates

Item #	Checklist item	Reported on page #
**Objectives and funding**		
**1**	Define the indicator(s), populations (including age, sex, and geographic entities), and time period(s) for which estimates were made.	Main text methods section (overview and input data)
**2**	List the funding sources for the work.	Main text abstract section (funding statement) and acknowledgements section
**Data Inputs**		
For all data inputs from multiple sources that are synthesized as part of the study:		
**3**	Describe how the data were identified and how the data were accessed.	Main text methods section and supplementary appendix (sections 2, 3.2.1, 3.3.1, 3.4.1, 3.5.1, and 3.6.1)
**4**	Specify the inclusion and exclusion criteria. Identify all ad-hoc exclusions.	Supplementary appendix (section 2)
**5**	Provide information on all included data sources and their main characteristics. For each data source used, report reference information or contact name/institution, population represented, data collection method, year(s) of data collection, sex and age range, diagnostic criteria or measurement method, and sample size, as relevant.	Supplementary appendix (section 2) and https://ghdx.healthdata.org/gbd-2019/data-input-sources

**6**	Identify and describe any categories of input data that have potentially important biases (e.g., based on characteristics listed in item 5).	Main text limitations section and supplementary appendix (biases for input data in each modelling step identified in each section)
For data inputs that contribute to the analysis but were not synthesized as part of the study:		
**7**	Describe and give sources for any other data inputs.	GBD 2019 estimates (https://ghdx.healthdata.org/gbd-results-tool)
For all data inputs:		
**8**	Provide all data inputs in a file format from which data can be efficiently extracted (e.g., a spreadsheet rather than a PDF), including all relevant meta-data listed in item 5. For any data inputs that cannot be shared because of ethical or legal reasons, such as third-party ownership, provide a contact name or the name of the institution that retains the right to the data.	Data inputs and/or contact information available at (https://ghdx.healthdata.org/gbd-2019/data-input-sources)
**Data analysis**		
**9**	Provide a conceptual overview of the data analysis method. A diagram may be helpful.	Main text methods section
**10**	Provide a detailed description of all steps of the analysis, including mathematical formulae. This description should cover, as relevant, data cleaning, data pre-processing, data adjustments and weighting of data sources, and mathematical or statistical model(s).	Supplementary appendix (section 3)
**11**	Describe how candidate models were evaluated and how the final model(s) were selected.	Supplementary appendix (section 3)
**12**	Provide the results of an evaluation of model performance, if done, as well as the results of any relevant sensitivity analysis.	Supplementary appendix (section 3.5.3)
**13**	Describe methods for calculating uncertainty of the estimates. State which sources of uncertainty were, and were not, accounted for in the uncertainty analysis.	Main text methods section (modelling tools and framework), main text limitations section, and supplementary

		appendix (section 3)
**14**	State how analytic or statistical source code used to generate estimates can be accessed.	Main text methods section (link to GitHub code will be available at the time of publication)
**Results and Discussion**		
**15**	Provide published estimates in a file format from which data can be efficiently extracted.	Main text results section. CSV files are available upon request to the corresponding author
**16**	Report a quantitative measure of the uncertainty of the estimates (e.g. uncertainty intervals).	Uncertainty intervals are provided for all estimates throughout the main text (summary, results, and discussion sections)
**17**	Interpret results in light of existing evidence. If updating a previous set of estimates, describe the reasons for changes in estimates.	Main text (research in context, introduction, and discussion sections)
**18**	Discuss limitations of the estimates. Include a discussion of any modelling assumptions or data limitations that affect interpretation of the estimates.	Main text limitations section and supplementary appendix (section 3)

This checklist should be used in conjunction with the GATHER statement and Explanation and Elaboration document, found on gather-statement.org

Section 6: PRISMA Compliance: Preferred Reporting Items for Systematic Reviews and Meta-Analyses

Prisma 2020 Checklist

Section and Topic	Item #	Checklist item	Location where item is reported
**TITLE**			
Title	1	Identify the report as a systematic review.	This report is not a systematic review, but utilises the input data from 24 systematic reviews.
**ABSTRACT**			
Abstract	2	See the PRISMA 2020 for Abstracts checklist.	See PRISMA 2020 for Abstracts Checklist below (appendix p 70)
**INTRODUCTION**			
Rationale	3	Describe the rationale for the review in the context of existing knowledge.	The evidence before this study is found in the Research in Context section of the manuscript.
Objectives	4	Provide an explicit statement of the objective(s) or question(s) the review addresses.	The objective of this study can be found in the Introduction section of the main text.
**METHODS**			
Eligibility criteria	5	Specify the inclusion and exclusion criteria for the review and how studies were grouped for the syntheses.	This is in section 2.2 of the appendix.
Information sources	6	Specify all databases, registers, websites, organisations, reference lists and other sources searched or consulted to identify studies. Specify the date when each source was last searched or consulted.	This can be found in section 2.2 of the appendix and the PRISMA diagrams for each review.
Search strategy	7	Present the full search strategies for all databases, registers and websites, including any filters and limits used.	All search strings and strategies are in the literature review section of the appendix.
Selection process	8	Specify the methods used to decide whether a study met the inclusion criteria of the review, including how many reviewers screened each record and each report retrieved, whether they worked independently, and if applicable, details of automation tools used in the process.	The exclusion criteria for the systematic reviews are documented in section 2.2 of the appendix and were screened by a project team member. No automation was used.
Data collection process	9	Specify the methods used to collect data from reports, including how many reviewers collected data from each report, whether they worked independently, any processes for obtaining or confirming data from study investigators, and if	The extraction template for the systematic reviews

Section and Topic	Item #	Checklist item	Location where item is reported
		applicable, details of automation tools used in the process.	will be published along with the GHDx upon publication. Articles were screened by a project team member. No automation was used.
Data items	10a	List and define all outcomes for which data were sought. Specify whether all results that were compatible with each outcome domain in each study were sought (e.g. for all measures, time points, analyses), and if not, the methods used to decide which results to collect.	The outcomes are described in the Estimation Steps section of the manuscript.
	10b	List and define all other variables for which data were sought (e.g. participant and intervention characteristics, funding sources). Describe any assumptions made about any missing or unclear information.	The outcomes are described in the Estimation Steps section of the manuscript and the extraction templates will be available in the GHDx upon publication. The assumptions and their associated limitations are detailed in the Limitations section of the manuscript.
Study risk of bias assessment	11	Specify the methods used to assess risk of bias in the included studies, including details of the tool(s) used, how many reviewers assessed each study and whether they worked independently, and if applicable, details of automation tools used in the process.	The potential bias of the input data, modelling, and the associated limitations can be found in the "Limitations" section of the main text.
Effect measures	12	Specify for each outcome the effect measure(s) (e.g. risk ratio, mean difference) used in the synthesis or presentation of results.	NA
Synthesis methods	13a	Describe the processes used to decide which studies were eligible for each synthesis (e.g. tabulating the study intervention characteristics and comparing against the planned groups for each synthesis (item #5)).	This information is available in the "Data Inputs" section of the main text and appendix section 2.1.
	13b	Describe any methods required to prepare the data for presentation or synthesis, such as handling of missing summary statistics, or data conversions.	Detailed methods on the estimation process have been published previously[1] and can be found in the Results and Limitations section of this manuscript.
	13c	Describe any methods used to tabulate or visually display results of individual studies and syntheses.	NA

Section and Topic	Item #	Checklist item	Location where item is reported
	13d	Describe any methods used to synthesize results and provide a rationale for the choice(s). If meta-analysis was performed, describe the model(s), method(s) to identify the presence and extent of statistical heterogeneity, and software package(s) used.	Details on the methods can be found in the "Methods" section of the main text, Section 3 of the appendix and have been published previously.[1]
	13e	Describe any methods used to explore possible causes of heterogeneity among study results (e.g. subgroup analysis, meta-regression).	Details on heterogeneity in the prevalence of resistance models can be found in Section 3.5 of the appendix.
	13f	Describe any sensitivity analyses conducted to assess robustness of the synthesized results.	This can be found in the "Uncertainty analysis" section of the main text, Sections 3.2.6, 3.4.7, 3.5.6 and 3.6.3 of the appendix and these methods have been published previously.[2]
Reporting bias assessment	14	Describe any methods used to assess risk of bias due to missing results in a synthesis (arising from reporting biases).	Detailed methods on the estimation process have been published previously[1] and can be found in the Results and Limitations section of this manuscript.
Certainty assessment	15	Describe any methods used to assess certainty (or confidence) in the body of evidence for an outcome.	Please see section 13f.
**RESULTS**			
Study selection	16a	Describe the results of the search and selection process, from the number of records identified in the search to the number of studies included in the review, ideally using a flow diagram.	All prisma diagrams for the literature searches conducted for the prevalence of resistance and relative risk modelling steps can be found in Section 6 of the appendix. The pathogen distribution diagrams are under review.[30]
	16b	Cite studies that might appear to meet the inclusion criteria, but which were excluded, and explain why they were excluded.	We did not encounter studies that meet this definition. Any studies outliered will be included in the

Section and Topic	Item #	Checklist item	Location where item is reported
			citation list on the GHDx and will be available upon publication.
Study characteristics	17	Cite each included study and present its characteristics.	All study citations will be included in the GHDx record for the manuscript and will be available upon publication.
Risk of bias in studies	18	Present assessments of risk of bias for each included study.	The assessment of bias in the input data is available in the limitations section and previously published.[1]
Results of individual studies	19	For all outcomes, present, for each study: (a) summary statistics for each group (where appropriate) and (b) an effect estimate and its precision (e.g. confidence/credible interval), ideally using structured tables or plots.	Because this report is not a systematic review, this was not included.
Results of syntheses	20a	For each synthesis, briefly summarise the characteristics and risk of bias among contributing studies.	The bias of input data and the overall bias in our study can be found in the "Limitations" section of the main text and throughout the appendix.
	20b	Present results of all statistical syntheses conducted. If meta-analysis was done, present for each the summary estimate and its precision (e.g. confidence/credible interval) and measures of statistical heterogeneity. If comparing groups, describe the direction of the effect.	The results can be found in the "Results" section of the main text, throughout the text in the manuscript and in the "Uncertainty analysis" section of the manuscript.
	20c	Present results of all investigations of possible causes of heterogeneity among study results.	Because this report is not a systematic review, this was not included.
	20d	Present results of all sensitivity analyses conducted to assess the robustness of the synthesized results.	This can be found in the "Uncertainty analysis" section of the main text, Sections 3.2.6, 3.4.7, 3.5.6 and 3.6.3 of the appendix and these methods have been published previously.[2]
Reporting biases	21	Present assessments of risk of bias due to missing results (arising from reporting biases) for each synthesis assessed.	Assessments of risk for each modelling component can be

Section and Topic	Item #	Checklist item	Location where item is reported
			found in Sections 3.2-3.6 of the appendix.
Certainty of evidence	22	Present assessments of certainty (or confidence) in the body of evidence for each outcome assessed.	This can be found in the "Uncertainty analysis" section of the main text, Sections 3.2.6, 3.4.7, 3.5.6 and 3.6.3 of the appendix and these methods have been published previously.[2]
**DISCUSSION**			
Discussion	23a	Provide a general interpretation of the results in the context of other evidence.	This can be found in the "Research in Context" section of the main text.
	23b	Discuss any limitations of the evidence included in the review.	This can be found in the "Limitations" paragraph in the "Discussion" section of the main text.
	23c	Discuss any limitations of the review processes used.	The exclusion criteria can be found in Section 2.2 of the appendix.
	23d	Discuss implications of the results for practice, policy, and future research.	This can be found in the "Discussion" section of the main text.
**OTHER INFORMATION**			
Registration and protocol	24a	Provide registration information for the review, including register name and registration number, or state that the review was not registered.	The entirety of the Global Burden of Disease, Injuries, and Risk Factors Study has been registered and approved through the UW IRB. The systematic reviews contained in this manuscript were not registered on its own.
	24b	Indicate where the review protocol can be accessed, or state that a protocol was not prepared.	We did not prepare a review protocol.
	24c	Describe and explain any amendments to information provided at registration or in the protocol.	N/A
Support	25	Describe sources of financial or non-financial support for the review, and the role of the funders or sponsors in the review.	Financial support can be found in the "Acknowledgments" section of the main text.

Section and Topic	Item #	Checklist item	Location where item is reported
Competing interests	26	Declare any competing interests of review authors.	These can be found in the “Declaration of interests” section of the main text and will be finalised following resubmission.
Availability of data, code and other materials	27	Report which of the following are publicly available and where they can be found: template data collection forms; data extracted from included studies; data used for all analyses; analytic code; any other materials used in the review.	The data collection forms, citations for all data used, analytic code and the results will be available on the GHDx upon publication.

PRISMA 2020 for Abstracts Checklist

Section and Topic	Item #	Checklist item	Reported (Yes/No)
**TITLE**			
Title	1	Identify the report as a systematic review.	No, this study is not a systematic review
**BACKGROUND**			
Objectives	2	Provide an explicit statement of the main objective(s) or question(s) the review addresses.	Yes
**METHODS**			
Eligibility criteria	3	Specify the inclusion and exclusion criteria for the review.	No
Information sources	4	Specify the information sources (e.g. databases, registers) used to identify studies and the date when each was last searched.	No
Risk of bias	5	Specify the methods used to assess risk of bias in the included studies.	No
Synthesis of results	6	Specify the methods used to present and synthesise results.	Yes
**RESULTS**			
Included studies	7	Give the total number of included studies and participants and summarise relevant characteristics of studies.	No
Synthesis of results	8	Present results for main outcomes, preferably indicating the number of included studies and participants for each. If meta-analysis was done, report the summary estimate and confidence/credible interval. If comparing groups, indicate the direction of the effect (i.e. which group is favoured).	Yes
**DISCUSSION**			
Limitations of evidence	9	Provide a brief summary of the limitations of the evidence included in the review (e.g. study risk of bias, inconsistency and imprecision).	No
Interpretation	10	Provide a general interpretation of the results and important implications.	Yes
**OTHER**			
Funding	11	Specify the primary source of funding for the review.	Yes
Registration	12	Provide the register name and registration number.	N/A

From: Page MJ, McKenzie JE, Bossuyt PM, Boutron I, Hoffmann TC, Mulrow CD, et al. The PRISMA 2020 statement: an updated guideline for reporting systematic reviews. BMJ 2021;372:n71. doi: 10.1136/bmj.n71

PRISMA Diagrams

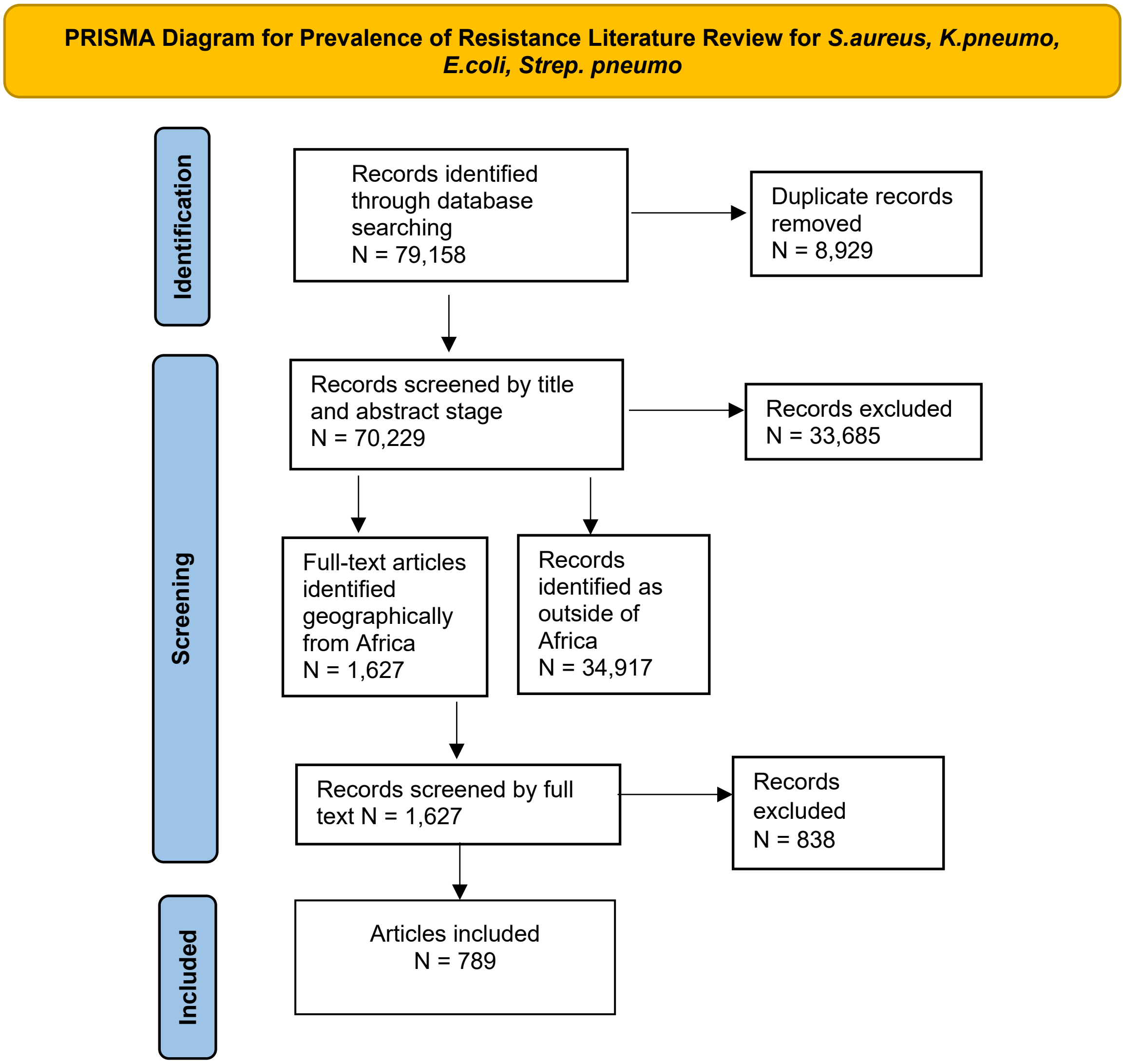

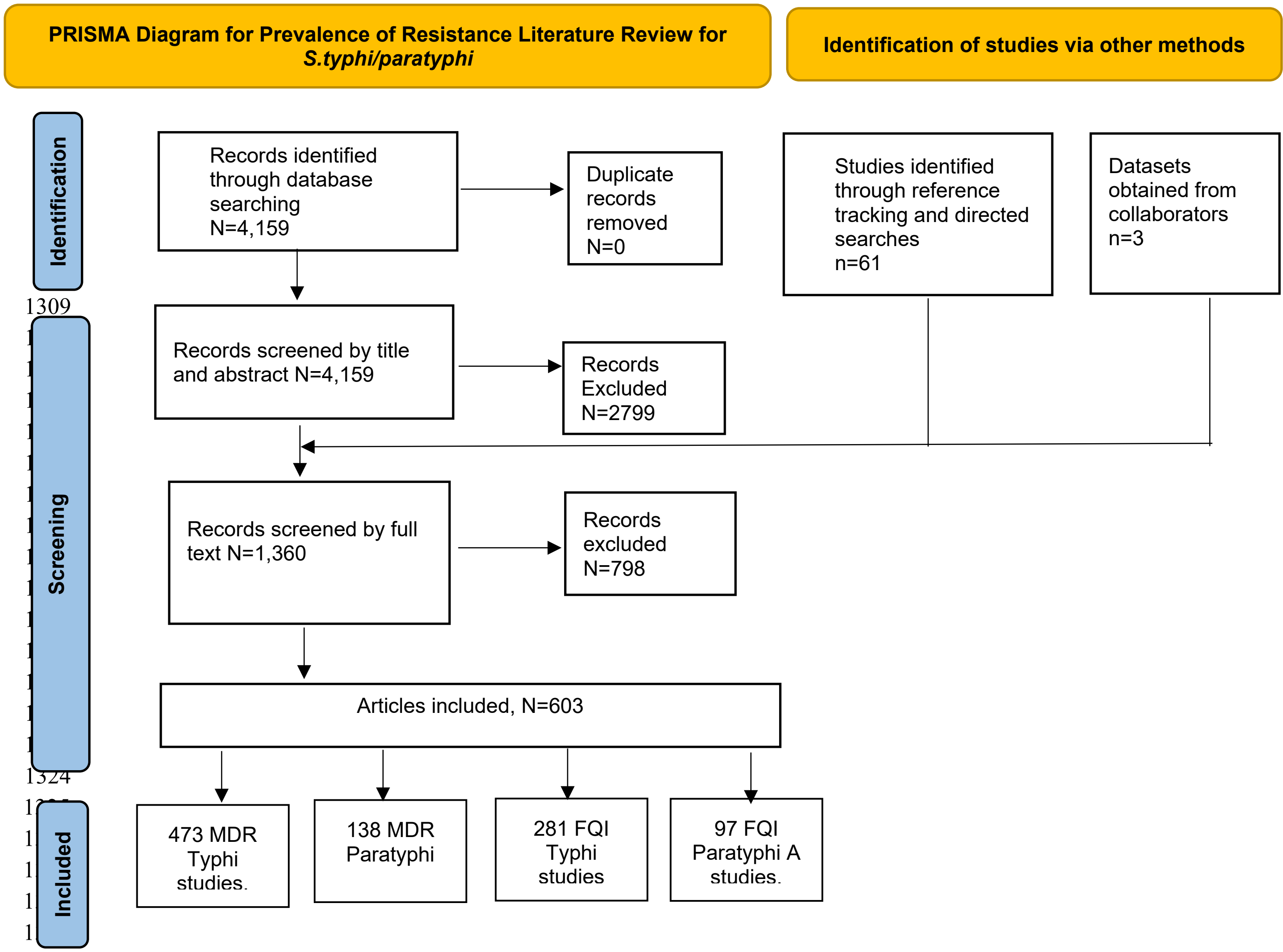

PRISMA Diagram for Prevalence of Resistance Literature Review for S.typhi/paratyphi
Identification of studies via other methods
Identification
Screening
Included
Records identified through database searching N=4,159
Duplicate records removed N=0
Studies identified through reference tracking and directed searches n=61
Datasets obtained from collaborators n=3
Records screened by title and abstract N=4,159
Records Excluded N=2799
Records screened by full text N=1,360
Records excluded N=798
Articles included, N=603
473 MDR Typhi studies.
138 MDR Paratyphi
281 FQI Typhi studies
97 FQI Paratyphi A studies.

PRISMA Diagram for Prevalence of Resistance Literature Review for *Shigella*

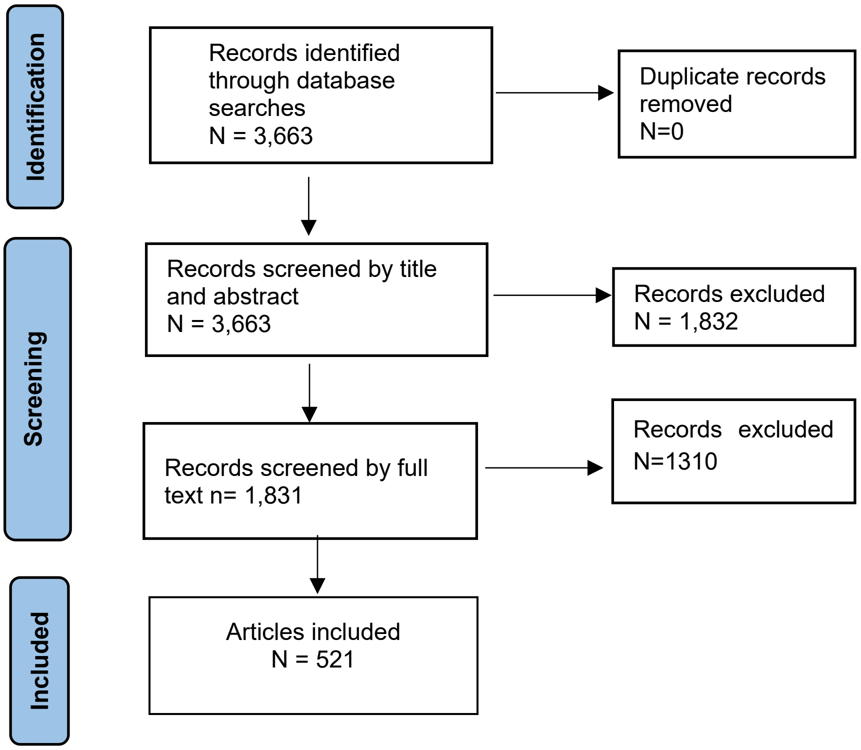

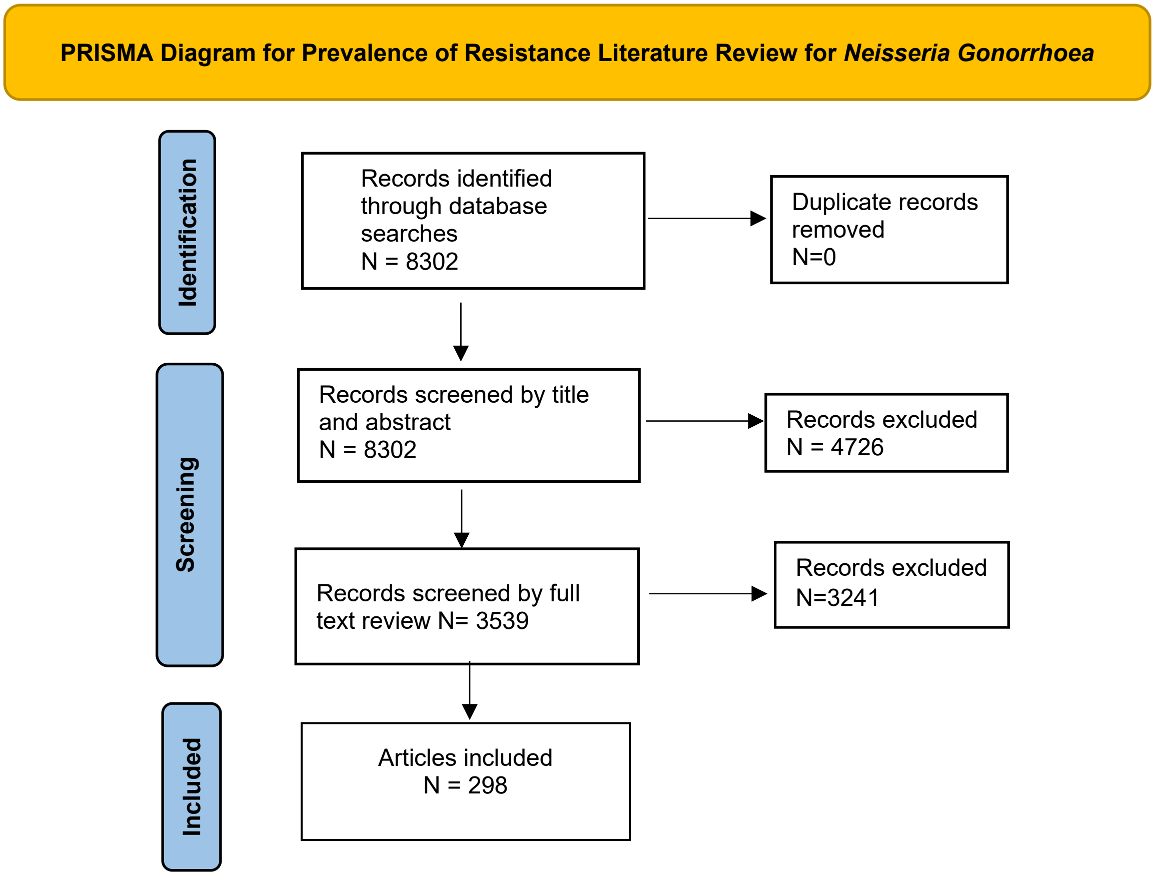
PRISMA Diagram for Prevalence of Resistance Literature Review for *Neisseria Gonorrhoea*
Identification
Screening
Included
Records identified through database searches N = 8302
Duplicate records removed N=0
Records screened by title and abstract N = 8302
Records excluded N = 4726
Records screened by full text review N= 3539
Records excluded N=3241
Articles included N = 298

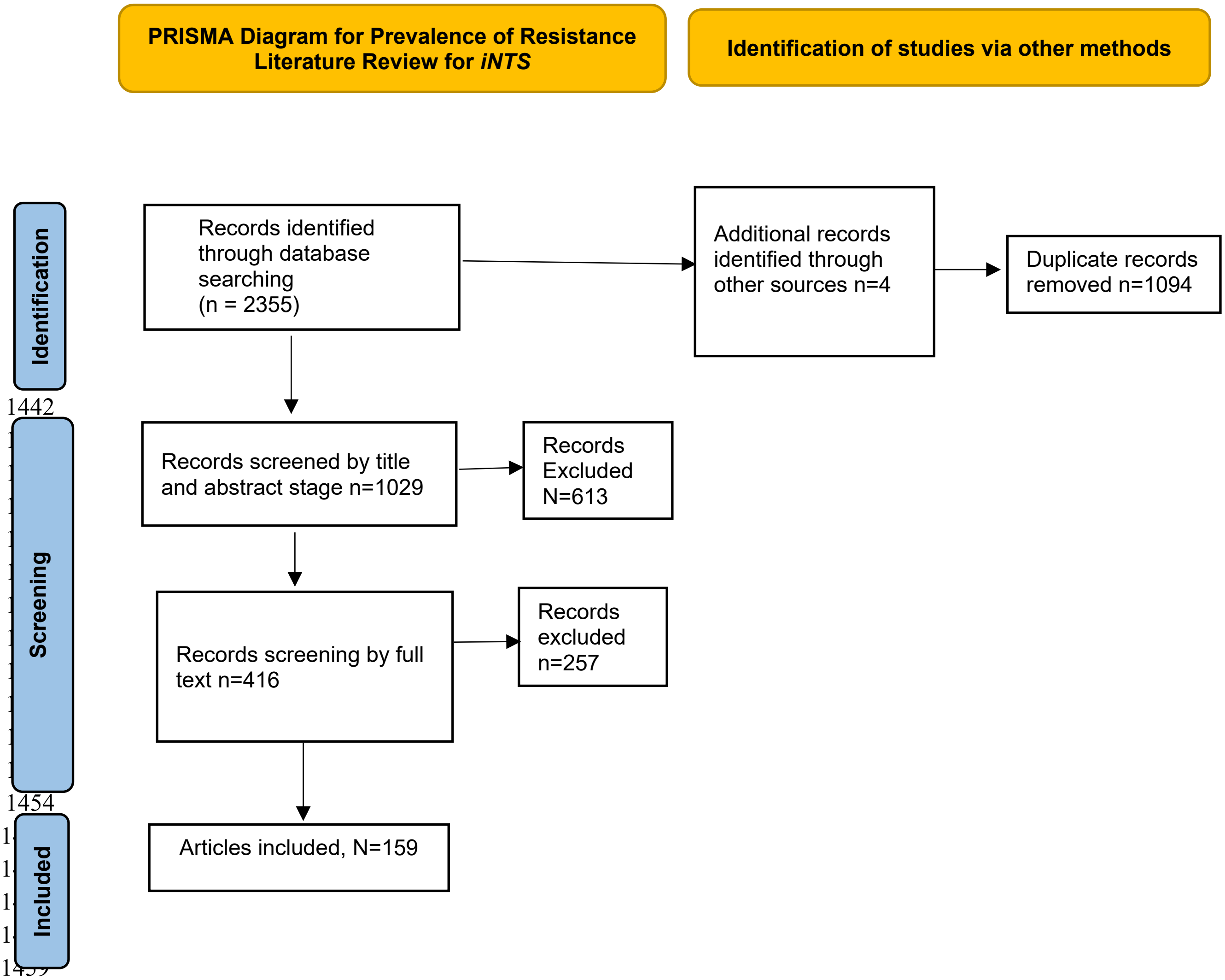
PRISMA Diagram for Prevalence of Resistance Literature Review for *iNTS*
Identification of studies via other methods
Identification
Records identified through database searching (n = 2355)
Additional records identified through other sources n=4
Duplicate records removed n=1094
Screening
Records screened by title and abstract stage n=1029
Records Excluded N=613
Records screening by full text n=416
Records excluded n=257
Included
Articles included, N=159

Prisma flow chart of Carbapenem resistant *Acinetobacter baumannii* review

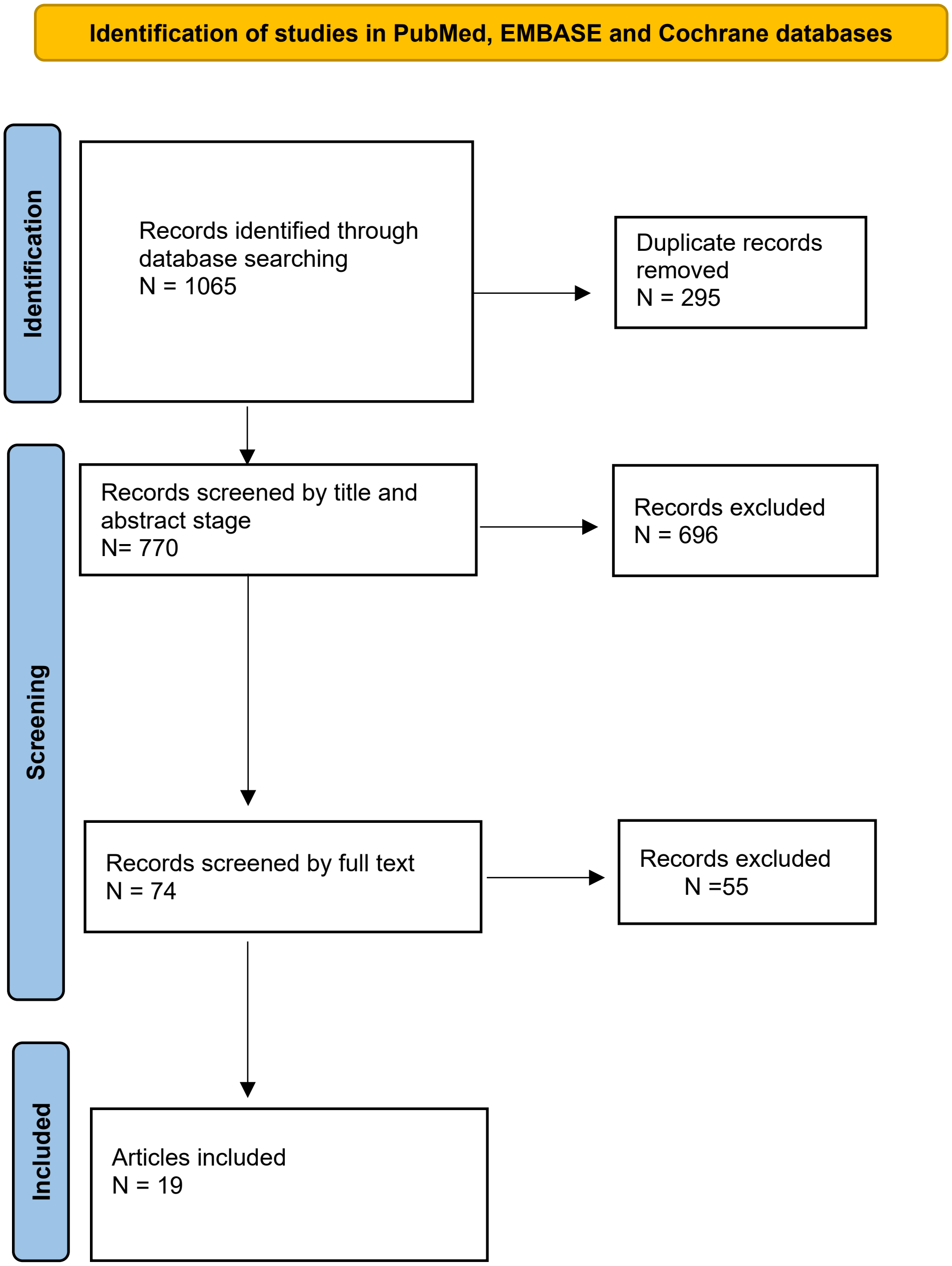

Prisma flow chart of Carbapenem resistant *Escherichia coli* review

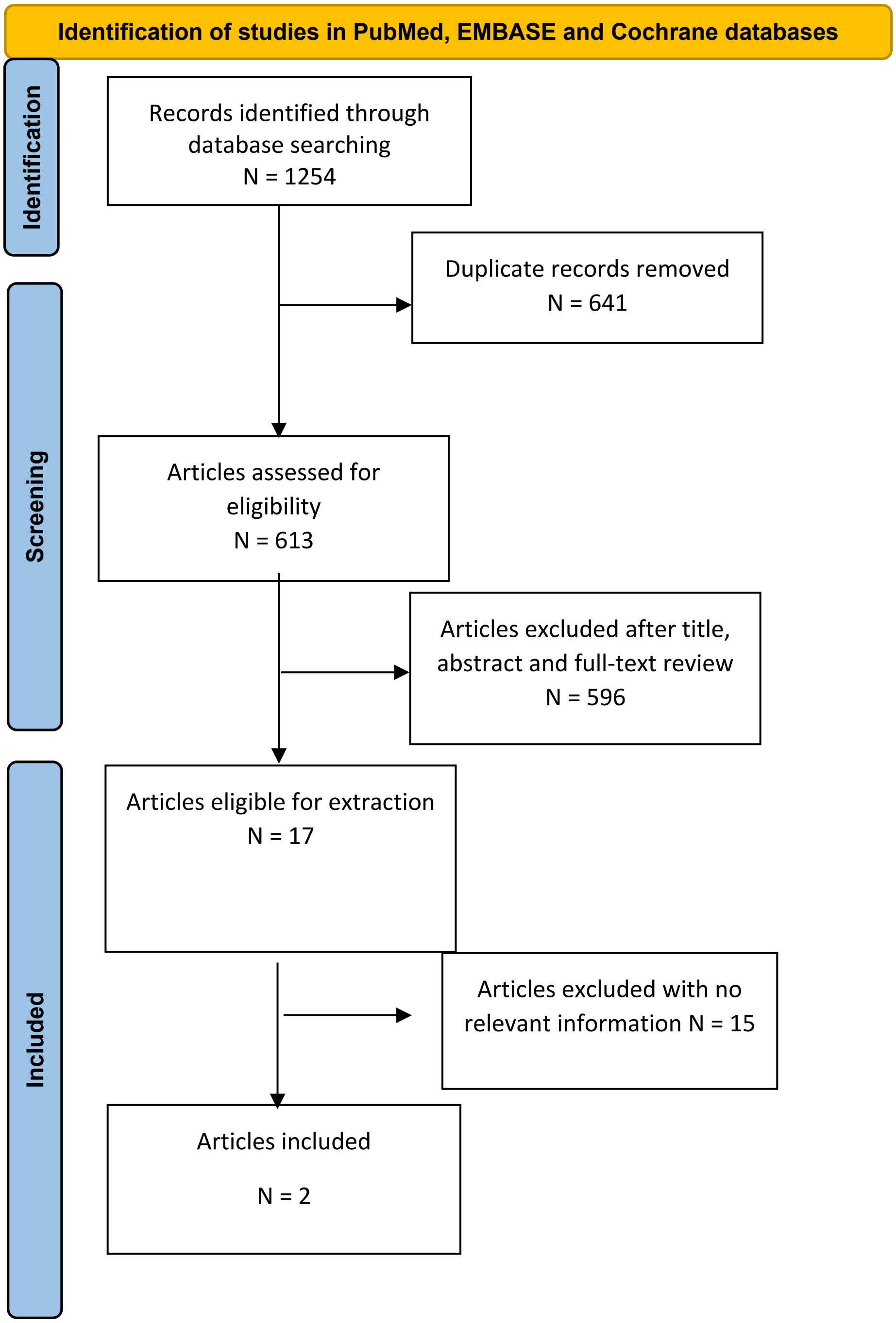

Prisma flow chart of 3rd generation cephalosporin resistant *Escherichia coli* review

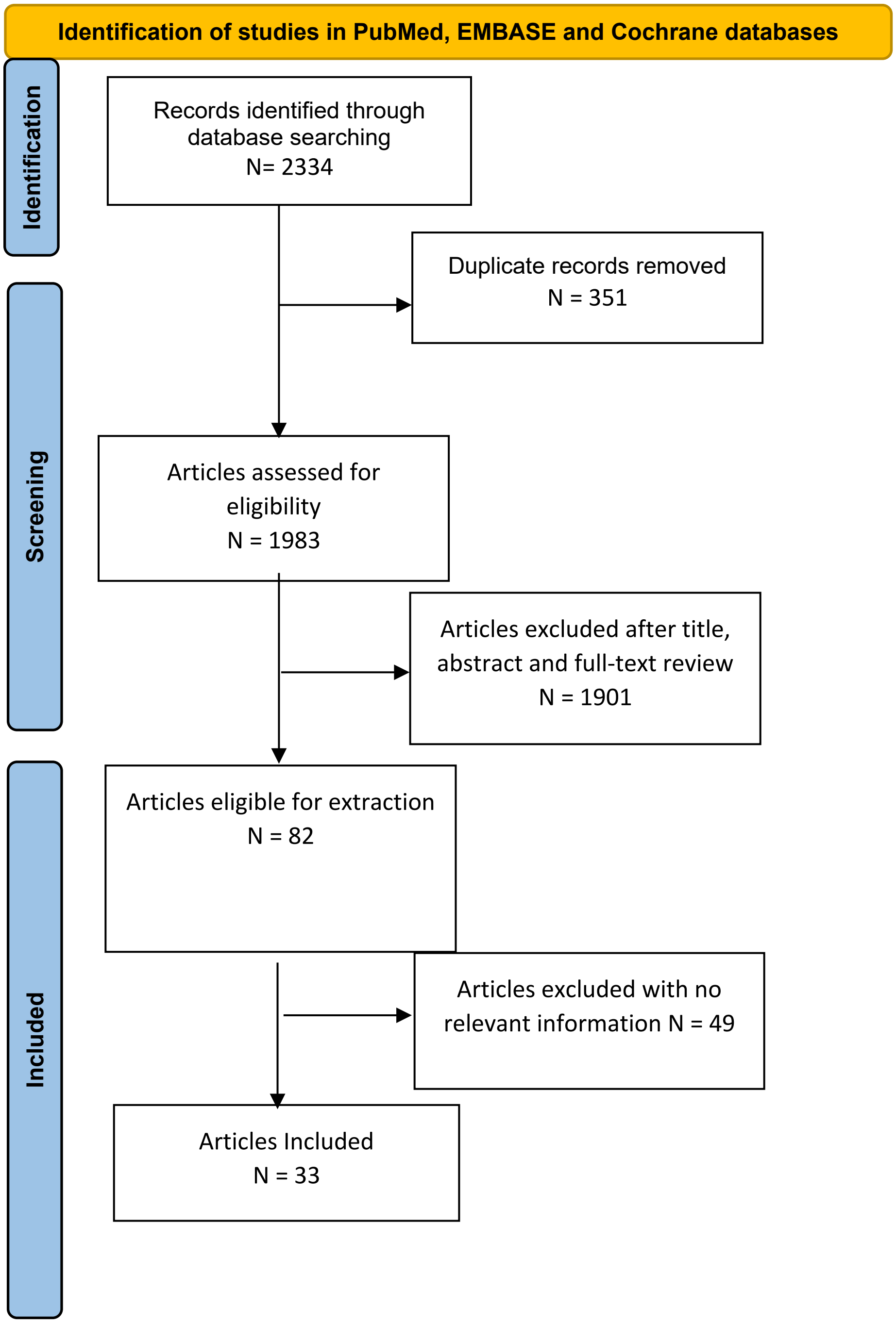

Prisma flow chart of Carbapenem resistant *Klebsiella pneumoniae* review

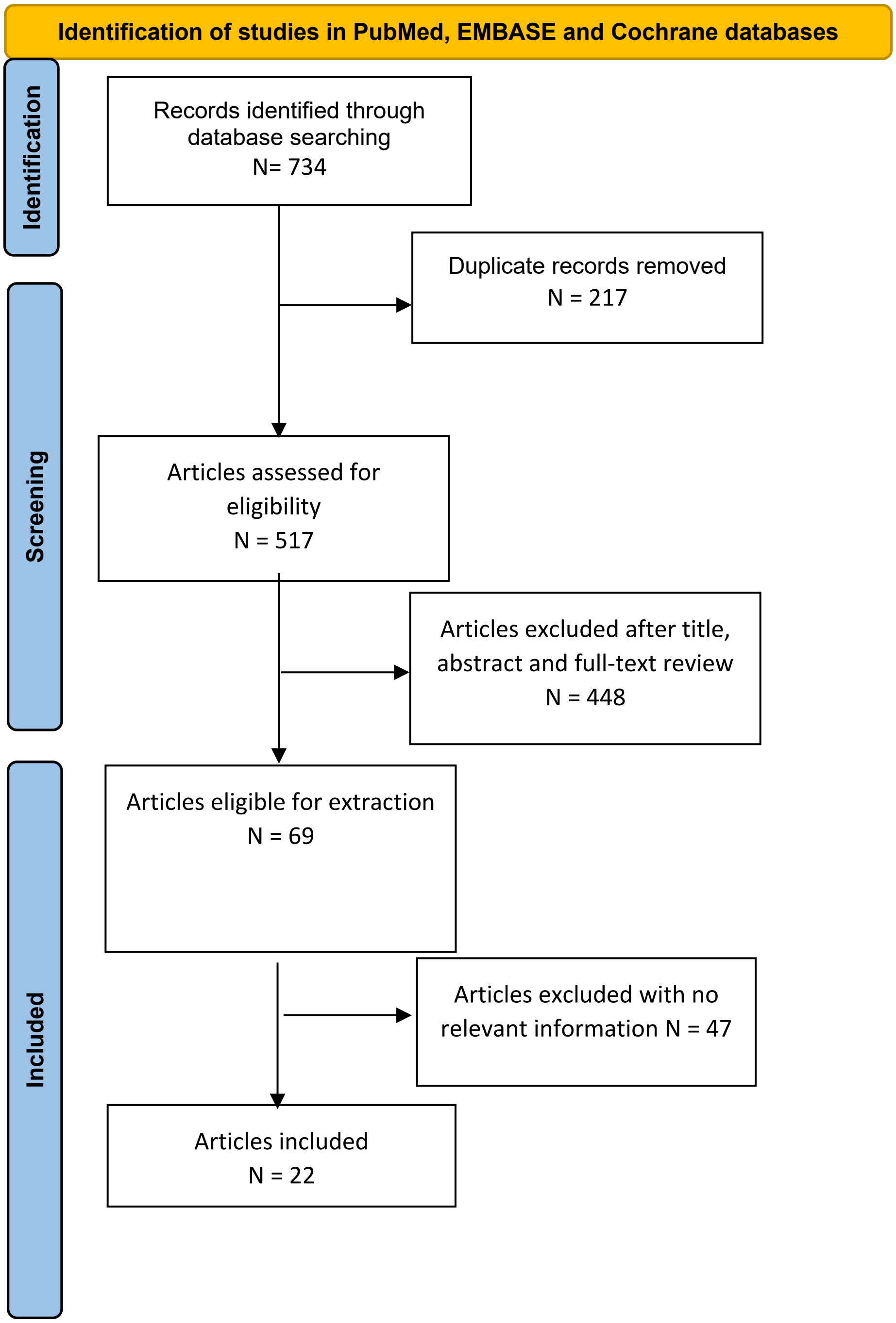

Prisma flow chart of 3rd generation cephalosporin resistant *Klebsiella pneumoniae* review

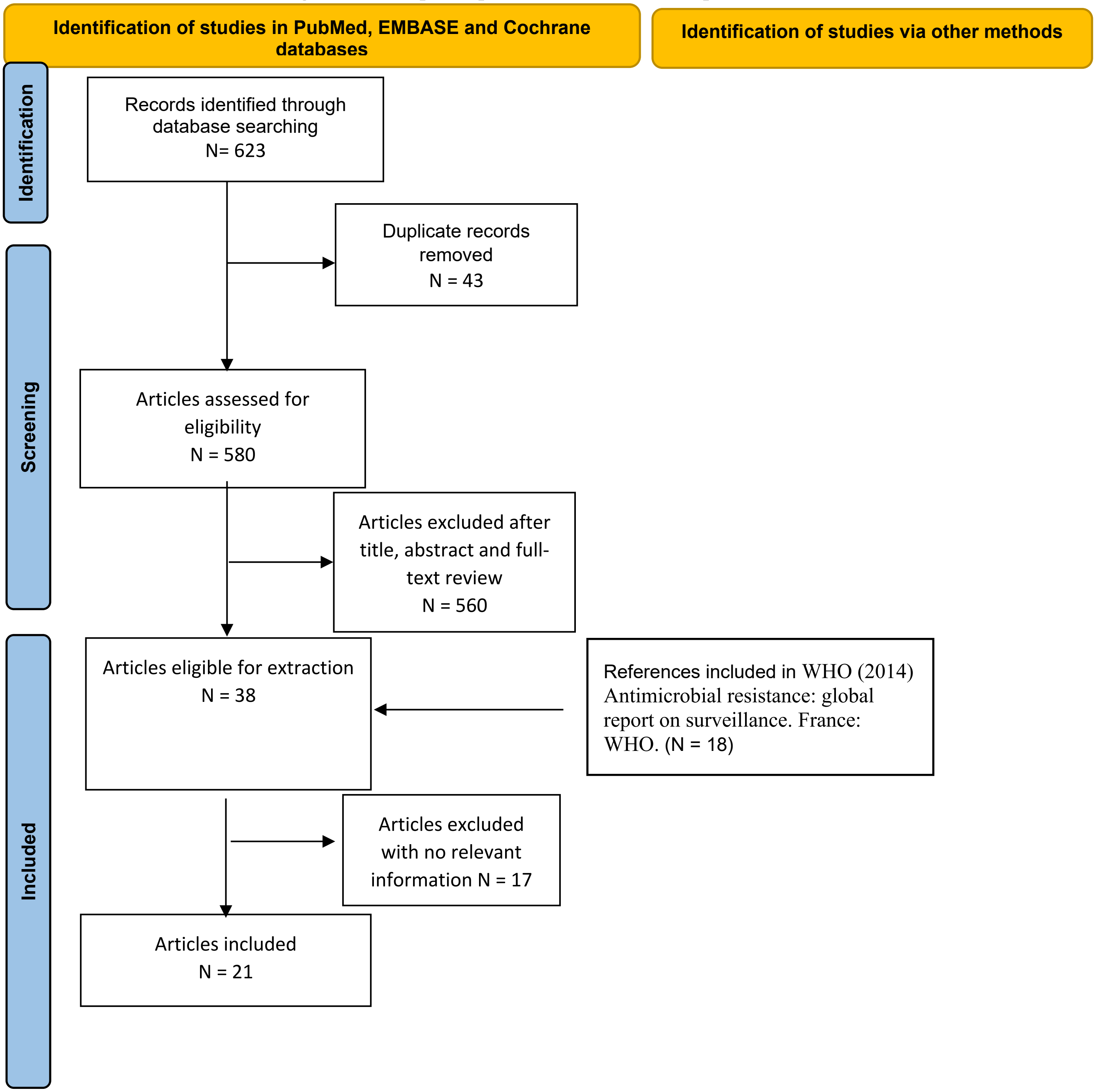

Prisma flow chart of Methicillin resistant *Staphylococcus aureus* review

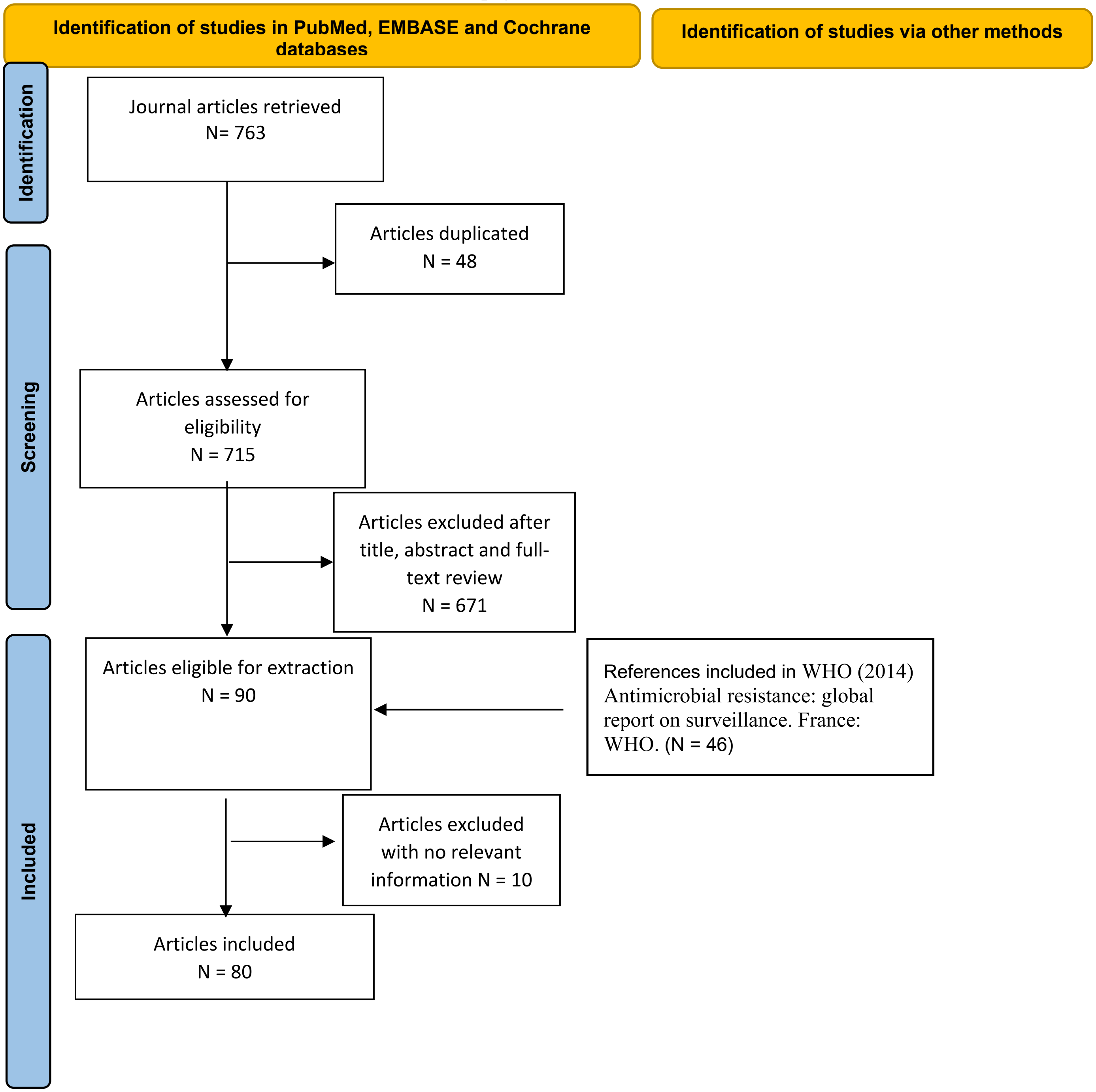

Prisma flow chart of Carbapenem resistant *Pseudomonas aeruginosa* review

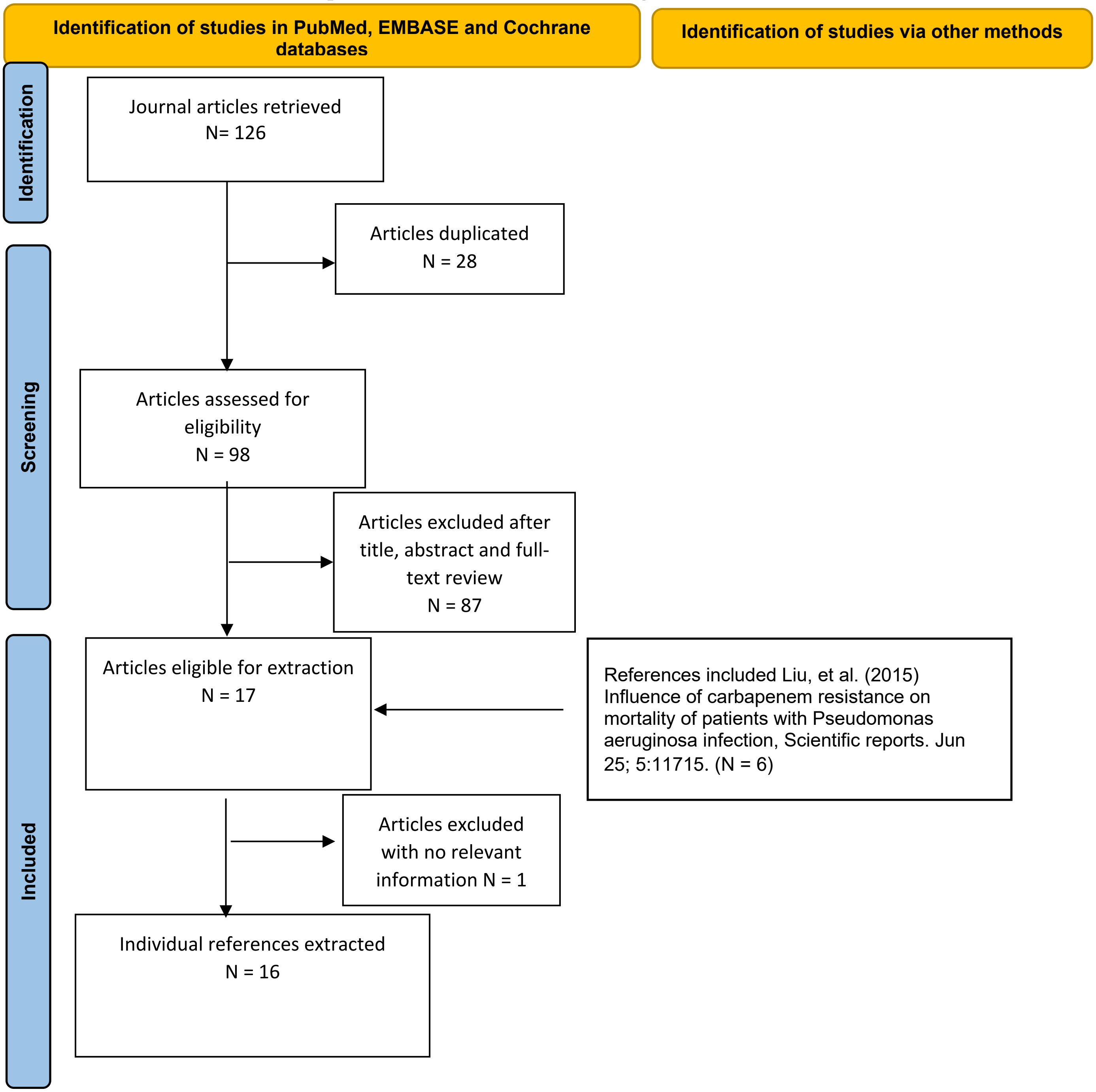

Prisma flow chart of Penicillin resistant *Streptococcus pneumoniae* review

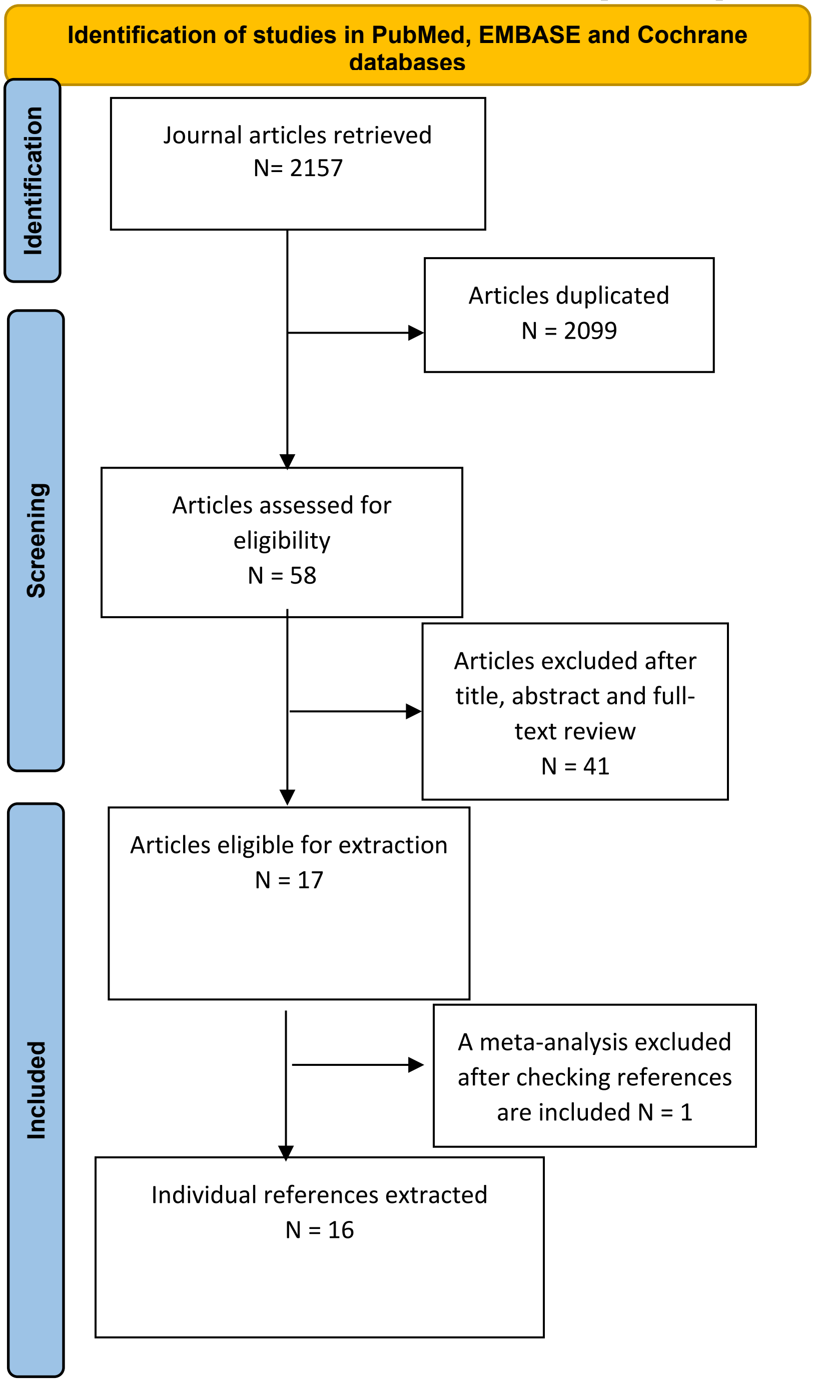

Prisma flow chart of Vancomycin resistant *Enterococcus species* review

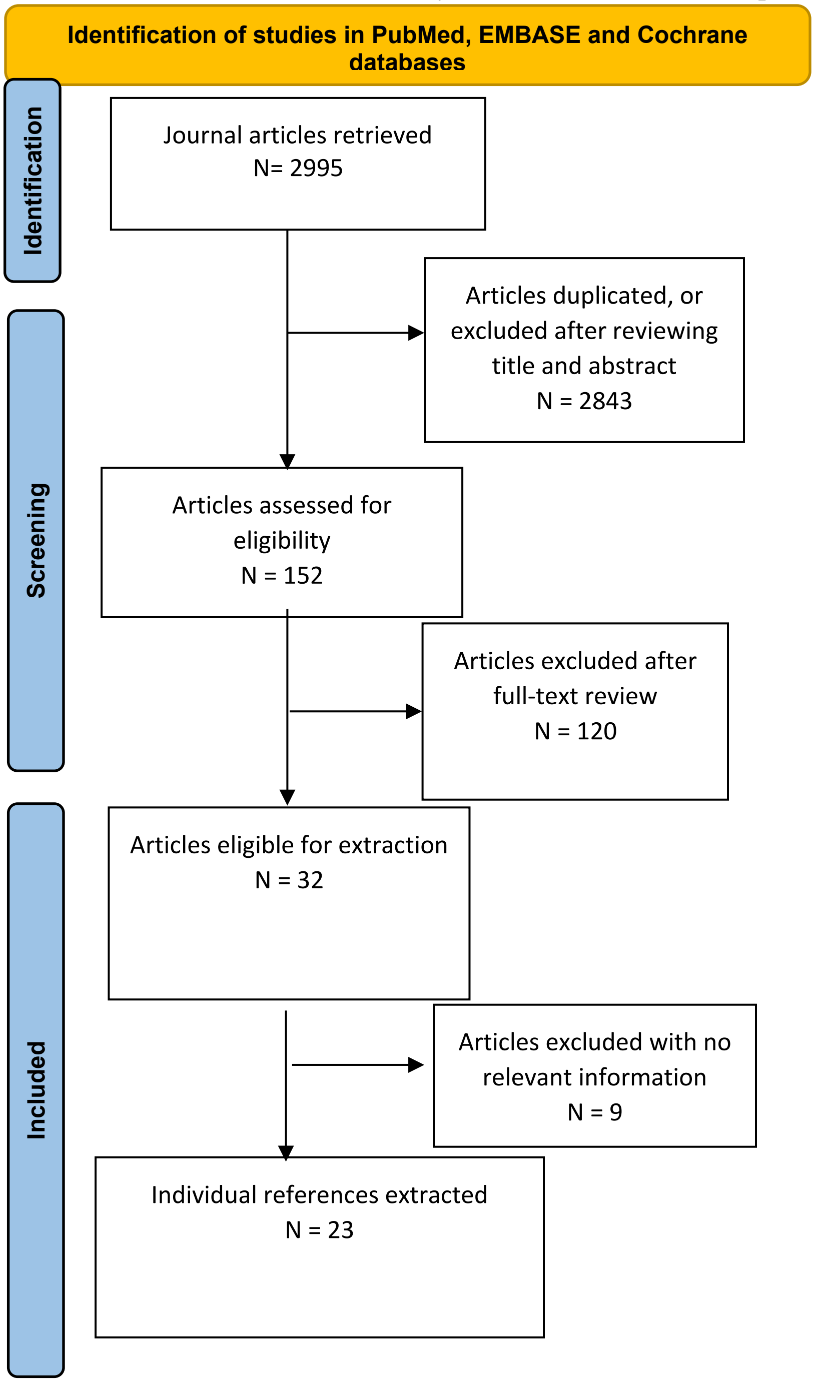

Section 7: References

1 Antimicrobial Resistance Collaborators. Global burden of bacterial antimicrobial resistance in 2019: a systematic analysis. *The Lancet* 2022; **399**: 629–55.

2 GBD 2019 Diseases and Injuries Collaborators. Global burden of 369 diseases and injuries in 204 countries and territories, 1990–2019: a systematic analysis for the Global Burden of Disease Study 2019. *The Lancet* 2020; **396**: 1204–22.

3 GBD 2019 Demographics Collaborators. Global age-sex-specific fertility, mortality, healthy life expectancy (HALE), and population estimates in 204 countries and territories, 1950–2019: a comprehensive demographic analysis for the Global Burden of Disease Study 2019. *The Lancet* 2020; **396**: 1160–203.

4 Rudd KE, Johnson SC, Agesa KM, *et al.* Global, regional, and national sepsis incidence and mortality, 1990–2017: analysis for the Global Burden of Disease Study. *The Lancet* 2020; **395**: 200–11.

5 Zheng P, Barber R, Sorensen RJD, Murray CJL, Aravkin AY. Trimmed constrained mixed effects models: formulations and algorithms. *J Comput Graph Stat* 2021; **30**: 544–56.

6 Ewig S, Schlochtermeier M, Göke N, Niederman MS. Applying sputum as a diagnostic tool in pneumonia: limited yield, minimal impact on treatment decisions. *Chest* 2002; **121**: 1486–92.

7 Ogawa H, Kitsios GD, Iwata M, Terasawa T. Sputum gram stain for bacterial pathogen diagnosis in community-acquired pneumonia: a systematic review and bayesian meta-analysis of diagnostic accuracy and yield. *Clin Infect Dis Off Publ Infect Dis Soc Am* 2020; **71**: 499–513.

8 Feikin DR, Scott JAG, Gessner BD. Use of vaccines as probes to define disease burden. *Lancet Lond Engl* 2014; **383**: 1762–70.

9 GBD 2016 Lower Respiratory Infections Collaborators. Estimates of the global, regional, and national morbidity, mortality, and aetiologies of lower respiratory infections in 195 countries, 1990-2016: a systematic analysis for the Global Burden of Disease Study 2016. *Lancet Infect Dis* 2018; **18**: 1191–210.

10 Johnson HL, Deloria-Knoll M, Levine OS, *et al.* Systematic evaluation of serotypes causing invasive pneumococcal disease among children under five: The Pneumococcal Global Serotype Project. *PLOS Med* 2010; **7**: e1000348.

11 Grijalva CG, Nuorti JP, Arbogast PG, Martin SW, Edwards KM, Griffin MR. Decline in pneumonia admissions after routine childhood immunisation with pneumococcal conjugate vaccine in the USA: a time-series analysis. *Lancet Lond Engl* 2007; **369**: 1179–86.

12 Nocedal J, Wright S. Numerical Optimization, 2nd edn. New York: Springer-Verlag, 2006 https://link.springer.com/book/10.1007/978-0-387-40065-5 (accessed Aug 8, 2022).

13 GBD Diarrhoeal Diseases Collaborators. Estimates of global, regional, and national morbidity, mortality, and aetiologies of diarrhoeal diseases: a systematic analysis for the Global Burden of Disease Study 2015. *Lancet Infect Dis* 2017; **17**: 909–48.

14 Moher D, Liberati A, Tetzlaff J, Altman DG, PRISMA Group. Preferred reporting items for systematic reviews and meta-analyses: the PRISMA statement. *PLoS Med* 2009; **6**: e1000097.

15 Browne AJ, Kashef Hamadani BH, Kumaran EAP, et al. Drug-resistant enteric fever worldwide, 1990 to 2018: a systematic review and meta-analysis. *BMC Med* 2020; **18**.

16 Jamison DT, Breman JG, Measham AR, *et al.*, editors. Disease control priorities in developing countries, 2nd edn. Washington (DC): World Bank, 2006 http://www.ncbi.nlm.nih.gov/books/NBK11728/ (accessed Aug 8, 2022).

17 Browne A, Chipeta M, Haines-Woodhouse G, et al. Global antibiotic consumption and usage in humans, 2000–18: a spatial modelling study. *Lancet Planet Health* 2021; **5**: E893–904.

18 Cassini A, Högberg LD, Plachouras D, *et al.* Attributable deaths and disability-adjusted life-years caused by infections with antibiotic-resistant bacteria in the EU and the European Economic Area in 2015: a population-level modelling analysis. *Lancet Infect Dis* 2019; **19**: 56–66.

19 Rajendran NB, Mutters NT, Marasca G, et al. Mandatory surveillance and outbreaks reporting of the WHO priority pathogens for research & discovery of new antibiotics in European countries. *Clin Microbiol Infect Off Publ Eur Soc Clin Microbiol Infect Dis* 2020; **26**: 943.e1-943.e6.

20 Versporten A, Gyssens IC, Pulcini C, *et al.* Metrics to assess the quantity of antibiotic use in the outpatient setting: a systematic review followed by an international multidisciplinary consensus procedure. *J Antimicrob Chemother* 2018; **73**: vi59–66.

21 Ahmed S, Jhaj R, Patidar R, *et al.* A novel metric system to quantify antibiotic consumption in paediatric population: a hospital based, biphasic pilot study. *Discoveries* 2020; **8**: e119.

22 Pulcini C, ESGAP AMOXDOSE working group. Amoxicillin dosing recommendations are very different in European countries: a cross-sectional survey. *Clin Microbiol Infect* 2017; **23**: 414–5.

23 Bruyndonckx R, Hens N, Aerts M, Goossens H, Molenberghs G, Coenen S. Measuring trends of outpatient antibiotic use in Europe: jointly modelling longitudinal data in defined daily doses and packages. *J Antimicrob Chemother* 2014; **69**: 1981–6.

24 Bruyndonckx R, Hens N, Aerts M, Goossens H, Cortiñas Abrahantes J, Coenen S. Exploring the association between resistance and outpatient antibiotic use expressed as DDDs or packages. *J Antimicrob Chemother* 2015; **70**: 1241–4.

25 López-Lozano J-M, Lawes T, Nebot C, *et al.* A nonlinear time-series analysis approach to identify thresholds in associations between population antibiotic use and rates of resistance. *Nat Microbiol* 2019; **4**: 1160–72.

26 Zanichelli V, Tebano G, Gyssens IC, *et al.* Patient-related determinants of antibiotic use: a systematic review. *Clin Microbiol Infect Off Publ Eur Soc Clin Microbiol Infect Dis* 2019; **25**: 48–53.

27 European Centre for Disease Prevention and Control (ECDC), Authority (EFSA) EFS, Agency (EMA) EM. Third joint inter-agency report on integrated analysis of consumption of antimicrobial agents and occurrence of antimicrobial resistance in bacteria from humans and food-producing animals in the EU/EEA. *EFSA J* 2021; **19**: e06712.

28 The European Committee on Antimicrobial Susceptibility Testing. Breakpoint tables for interpretation of MICs and zone diameters, Version 12.0, 2022. .

29 Clinical and Laboratory Standards Institute. Performance standards for antimicrobial susceptibility testing. 30th ed. CLSI supplement M100. Wayne, PA. 2020.

30 Global Burden of Disease & Antimicrobial Resistance Collaborators. Global mortality associated with 33 bacterial pathogens in 2019: a systematic analysis for the Global Burden of Disease Study 2019. *The Lancet*; **in press.**

Authors' Affiliations

Institute for Health Metrics and Evaluation (T Mestrovic PhD, L R Swetschinski MSc, K S Ikuta MD, A P Gray BSc, N Davis Weaver MPH, C Han BA, E E Wool MPH, A Gershberg Hayoon MSc, Prof S I Hay FMedSci, Prof C J L Murray DPhil, H H Kyu PhD, Prof M Naghavi PhD), Department of Health Metrics Sciences, School of Medicine (Prof S I Hay FMedSci, Prof C J L Murray DPhil, H H Kyu PhD, Prof A Stergachis PhD, Prof M Naghavi PhD), Department of Pharmacy (Prof A Stergachis PhD), University of Washington, Seattle, WA, USA; University Centre Varazdin (T Mestrovic PhD), University North, Varazdin, Croatia; Nuffield Department of Medicine (G Robles Aguilar DPhil, B Sartorius PhD), Oxford Centre for Global Health Research (C Dolecek PhD), Centre for Tropical Medicine and Global Health (B Sartorius PhD), University of Oxford, Oxford, UK; Division of Infectious Diseases (K S Ikuta MD), Veterans Affairs Greater Los Angeles, Los Angeles, CA, USA; Mahidol Oxford Tropical Medicine Research Unit (C Dolecek PhD), Mahidol University, Bangkok, Thailand; Centre for Social Research in Health (I Y Addo PhD), University of New South Wales, Sydney, NSW, Australia; Quality and Systems Performance Unit (I Y Addo PhD), Cancer Institute NSW, Sydney, NSW, Australia; School of Public Health (B O Ahinkorah MPhil), University of Technology Sydney, Sydney, NSW, Australia; Institute of Endemic Diseases (A Ahmed MSc), University of Khartoum, Khartoum, Sudan; Swiss Tropical and Public Health Institute (A Ahmed MSc), University of Basel, Basel, Switzerland; Department of Pharmacy (M A Aldeyab PhD), University of Huddersfield, Huddersfield, UK; Department of Disease Control (K Allel MSc), London School of Hygiene & Tropical Medicine, London, UK; Institute for Global Health (K Allel MSc), University College London, London, UK; Faculty of Pharmacy (Prof R Ancuceanu PhD), Department of General Surgery (I Negoi PhD, B Socea PhD), Department of Anatomy and Embryology (R I Negoi PhD), Carol Davila University of Medicine and Pharmacy, Bucharest, Romania; School of Dentistry and Medical Sciences (A E Anyasodor PhD), Charles Sturt University, Orange, NSW, Australia; Department of Statistics and Econometrics (Prof M Ausloos PhD, Prof C Herteliu PhD, I Petcu PhD), Bucharest University of Economic Studies, Bucharest, Romania; School of Business (Prof M Ausloos PhD), University of Leicester, Leicester, UK; Unit of Obstetrics and Gynecology (F Barra MD), Academic Unit of Obstetrics and Gynecology (Prof S Ferrero PhD), IRCCS Ospedale Policlinico San Martino, Genoa, Italy; Department of Health, Human Performance and Recreation (A S Bhagavathula PhD), University of Arkansas, Fayetteville, AR, USA; School of Public Health (D Bhandari PhD), University of Adelaide, Adelaide, SA, Australia; Public Health Research Laboratory (D Bhandari PhD), Tribhuvan University, Kathmandu, Nepal; Global Health Neurology Lab (S Bhaskar PhD), NSW Brain Clot Bank, Sydney, NSW, Australia; Department of Neurology and Neurophysiology (S Bhaskar PhD), South West Sydney Local Heath District and Liverpool Hospital, Sydney, NSW, Australia; Therapeutic and Diagnostic Technologies (Prof N Cruz-Martins PhD), Cooperativa de Ensino Superior Politecnico e UniversitÃ¡rio, Gandra, Portugal; Institute for Research and Innovation in Health (Prof N Cruz-Martins PhD), University of Porto, Porto, Portugal; 2nd University Ophthalmology Department (A Dastiridou MD), Aristotle University of Thessaloniki, Thessaloniki, Greece; Ophthalmology Dpt (A Dastiridou MD), Medical School (F Mulita PhD), University of Thessaly, Larissa, Greece; Department of Social Medicine and Health Care Organisation (K Dokova PhD), Department of Microbiology and Virology (Prof T Z Stoeva PhD), Medical University of Varna, Varna, Bulgaria; Institute of Microbiology and Immunology (E Dubljanin PhD), Faculty of Medicine (I M Ilic PhD), University of Belgrade, Belgrade, Serbia; Department of Infection and Tropical Medicine (O C Durojaiye MPH), University of Sheffield, Sheffield, UK; Department of Epidemiology and Medical Statistics (A F Fagbamigbe PhD), University of Ibadan, Ibadan, Nigeria; Population and Behavioural Sciences Division (A F Fagbamigbe PhD), St Andrews University, St Andrews, UK; Health Services Management Training Centre (P A Gaal PhD, T Joo PhD, J Lám PhD, T Palicz MD), Semmelweis University, Budapest, Hungary; Department of Applied Social Sciences (P A Gaal PhD), Sapientia Hungarian University of Transylvania, Târgu-Mureş, Romania; School of Medicine (V Gupta PhD), Deakin University, Geelong, VIC, Australia; Biorefining and Advanced Materials Research Center (V Gupta PhD), Scotland's

Rural College (SRUC), Edinburgh, UK; Faculty of Medicine Health and Human Sciences (Prof V K Gupta PhD), School of Engineering (N Rabiee PhD), Macquarie University, Sydney, NSW, Australia; School of Business (Prof C Herteliu PhD), London South Bank University, London, UK; Czech National Centre for Evidence-Based Healthcare and Knowledge Translation (S Hussain PhD), Institute of Biostatistics and Analyses (S Hussain PhD), Masaryk University, Brno, Czech Republic; Department of Epidemiology (Prof M D Ilic PhD), University of Kragujevac, Kragujevac, Serbia; Functional Neurosurgery Research Center (E Jamshidi PharmD), Shahid Beheshti University of Medical Sciences, Tehran, Iran; Division of Pulmonary Medicine (E Jamshidi PharmD), Lausanne University Hospital (CHUV), Lausanne, Switzerland; Hungarian Health Management Association (T Palicz MD), Hungarian Health Management Association, Budapest, Hungary (T Joo PhD); Institute for Epidemiology and Social Medicine (A Karch MD), University of Münster, Münster, Germany; School of Health Sciences (Prof A Kisa PhD), Kristiania University College, Oslo, Norway; Department of International Health and Sustainable Development (Prof A Kisa PhD), Tulane University, New Orleans, LA, USA; Department of Nursing and Health Promotion (S Kisa PhD), Oslo Metropolitan University, Oslo, Norway; Microbiology Department (T Kostyanev PhD), Laboratoire National de Santé, Dudelange, Luxembourg; Laboratory of Medical Microbiology (T Kostyanev PhD), University of Antwerp, Antwerp, Belgium; NEVES Society for Patient Safety, Budapest, Hungary (J Lám PhD); Interdisciplinary Centre of Marine and Environmental Research (G Lopes PhD), University of Porto, Matosinhos, Portugal; Division of Infection, Immunity and Respiratory Medicine (A G Mathioudakis PhD), University of Manchester, Manchester, UK; North West Lung Centre (A G Mathioudakis PhD), Manchester University NHS Foundation Trust, Manchester, UK; International Dx (A A Mentis MD), BGI Genomics, Shenzhen, China; Polish National Cancer Registry (I Michalek PhD), Department of Pathology (I Michalek PhD), Maria Sklodowska-Curie National Research Institute of Oncology, Warsaw, Poland; School of Health & Rehabilitation Sciences (M Moni PhD), The University of Queensland, Brisbane, QLD, Australia; Centre for Neonatal and Paediatric Infection (C E Moore PhD), St. George's University of London, London, UK; Department of Surgery (F Mulita PhD), General University Hospital of Patras, Patras, Greece; Department of General Surgery (I Negoi PhD), Emergency Hospital of Bucharest, Bucharest, Romania; Department of Cardiology (R I Negoi PhD), Cardio-Aid, Bucharest, Romania; Department of Public Health (A Pana PhD), Babes Bolyai University, Cluj Napoca, Romania; Department of Health Metrics (A Pana PhD), Center for Health Outcomes & Evaluation, Bucharest, Romania; Research Institute for Medicines (Prof J Perdigão PhD), University of Lisbon, Lisbon, Portugal; Pohang University of Science and Technology, Pohang, South Korea (N Rabiee PhD); WHO Collaborating Centre for Public Health Education and Training (D L Rawaf MRCS), Department of Primary Care and Public Health (Prof S Rawaf MD), Imperial College London, London, UK; Inovus Medical, St Helens, UK (D L Rawaf MRCS); Academic Public Health England (Prof S Rawaf MD), Public Health England, London, UK; Department of Infectious Diseases and Epidemiology (Prof M Z Shakhmardanov PhD, A A Skryabina MD), Pirogov Russian National Research Medical University, Moscow, Russia; Centre for Medical Informatics (Prof A Sheikh MD), University of Edinburgh, Edinburgh, UK; Division of General Internal Medicine (Prof A Sheikh MD), Harvard University, Boston, MA, USA; Center of Potential and Innovation of Natural Resources (Prof L M R Silva PhD), Polytechnic Institute of Guarda, Guarda, Portugal; Health Sciences Research Centre (Prof L M R Silva PhD), University of Beira Interior, Covilhã, Portugal; Clinical Branch (V Y Skryabin MD), Moscow Research and Practical Centre on Addictions, Moscow, Russia; Addiction Psychiatry Department (V Y Skryabin MD), Russian Medical Academy of Continuous Professional Education, Moscow, Russia; Surgery Department (B Socea PhD), "Sf. Pantelimon" Emergency Clinical Hospital Bucharest, Bucharest, Romania; Microbiology Laboratory (Prof T Z Stoeva PhD), University Hospital, Varna, Bulgaria; The University of Queensland Centre for Clinical Research (C Sumi PhD), The University of Queensland, Woollongabba, QLD, Australia; Department of Clinical Epidemiology (A Thiyagarajan MPH), Leibniz Institute for Prevention Research and Epidemiology, Bremen, Germany; Saveetha Dental College and Hospitals (M R Tovani-Palone PhD), Saveetha Institute of Medical and

Technical Sciences (SIMATS), Chennai, India; Modestum LTD, Eastbourne, UK (M R Tovani-Palone PhD); Department of Pharmacology, Physiology & Neuroscience (M Yesiltepe PhD), Rutgers University, Newark, NJ, USA; Clinical Investigation Unit (M Yesiltepe PhD), Ankara City Hospital, Ankara, Turkey; Department of Medicine (S Zaman MSc), Monash University, Melbourne, VIC, Australia; Maternal and Child Health Division (S Zaman MSc), International Centre for Diarrhoeal Disease Research, Bangladesh, Dhaka, Bangladesh

Authors' Contributions

Managing the overall research enterprise

Christiane Dolecek, Simon I Hay, Kevin S Ikuta, Christopher J L Murray, Mohsen Naghavi, Benn Sartorius, and Eve E Wool.

Writing the first draft of the manuscript

Nicole Davis Weaver, Kevin S Ikuta, Tomislav Mestrovic, Mohsen Naghavi, Gisela Robles Aguilar, and Lucien R Swetschinski.

Primary responsibility for applying analytical methods to produce estimates

Authia P Gray, Kevin S Ikuta, Gisela Robles Aguilar, Benn Sartorius, and Lucien R Swetschinski.

Primary responsibility for seeking, cataloguing, extracting, or cleaning data; designing or coding figures and tables

Anna Gershberg Hayoon, Authia P Gray, and Gisela Robles Aguilar.

Providing data or critical feedback on data sources

Bright Opoku Ahinkorah, Akshaya Srikanth Bhagavathula, Dinesh Bhandari, Sonu Bhaskar, Natália Cruz-Martins, Klara Dokova, Christiane Dolecek, Adeniyi Francis Fagbamigbe, Peter Andras Gaal, Anna Gershberg Hayoon, Vijai Kumar Gupta, Vivek Kumar Gupta, Chieh Han, Claudiu Herteliu, Salman Hussain, Kevin S Ikuta, Tamas Joo, André Karch, Adnan Kisa, Sezer Kisa, Tomislav Kostyanev, Hmwe Hmwe Kyu, Judit Lám, Tomislav Mestrovic, Catrin E Moore, Francesk Mulita, Christopher J L Murray, Mohsen Naghavi, Ionut Negoi, Ruxandra Irina Negoi, Tamás Palicz, Adrian Pana, Navid Rabiee, Salman Rawaf, Gisela Robles Aguilar, Benn Sartorius, Murad Ziyaudinovich Shakhmardanov, Luís Manuel Lopes Rodrigues Silva, Valentin Yurievich Skryabin, Anna Aleksandrovna Skryabina, Bogdan Socea, Andy Stergachis, Temenuga Zhekova Stoeva, Lucien R Swetschinski, and Marcos Roberto Tovani-Palone.

Developing methods or computational machinery

Christiane Dolecek, Authia P Gray, Chieh Han, Simon I Hay, Kevin S Ikuta, Adnan Kisa, Catrin E Moore, Francesk Mulita, Christopher J L Murray, Mohsen Naghavi, Navid Rabiee, Benn Sartorius, Andy Stergachis, and Lucien R Swetschinski.

Providing critical feedback on methods or results

Isaac Yeboah Addo, Bright Opoku Ahinkorah, Ayman Ahmed, Mamoon A Aldeyab, Kasim Allel, Robert Ancuceanu, Anayochukwu Edward Anyasodor, Marcel Ausloos, Akshaya Srikanth Bhagavathula, Dinesh Bhandari, Sonu Bhaskar, Natália Cruz-Martins, Klara Dokova, Christiane Dolecek, Oyewole Christopher Durojaiye, Adeniyi Francis Fagbamigbe, Peter Andras Gaal, Authia P Gray, Vijai Kumar Gupta, Vivek Kumar Gupta, Simon I Hay, Claudiu Herteliu, Salman Hussain, Kevin S Ikuta, Irena M Ilic, Milena D Ilic, Elham Jamshidi, Tamas Joo, André Karch, Adnan Kisa, Sezer Kisa, Tomislav Kostyanev, Hmwe Hmwe Kyu, Judit Lám, Graciliana Lopes, Alexander G Mathioudakis, Alexios-Fotios A Mentis, Tomislav Mestrovic, Irmina Maria Michalek, Mohammad Ali Moni, Catrin E Moore, Francesk Mulita, Christopher J L Murray, Mohsen Naghavi, Ionut Negoi, Ruxandra Irina Negoi, Tamás Palicz, Adrian Pana, Ionela-Roxana Petcu, Navid Rabiee, David Laith Rawaf, Salman Rawaf, Gisela Robles Aguilar, Benn Sartorius, Murad Ziyaudinovich Shakhmardanov, Aziz Sheikh, Luís Manuel Lopes Rodrigues Silva, Valentin Yurievich Skryabin,

Anna Aleksandrovna Skryabina, Bogdan Socea, Andy Stergachis, Temenuga Zhekova Stoeva, Lucien R Swetschinski, Arulmani Thiyagarajan, Marcos Roberto Tovani-Palone, Metin Yesiltepe, and Sojib Bin Zaman.

Drafting the work or revising is critically for important intellectual content

Isaac Yeboah Addo, Bright Opoku Ahinkorah, Ayman Ahmed, Kasim Allel, Robert Ancuceanu, Anayochukwu Edward Anyasodor, Fabio Barra, Akshaya Srikanth Bhagavathula, Dinesh Bhandari, Sonu Bhaskar, Natália Cruz-Martins, Anna Dastiridou, Nicole Davis Weaver, Christiane Dolecek, Eleonora Dubljanin, Oyewole Christopher Durojaiye, Adeniyi Francis Fagbamigbe, Simone Ferrero, Peter Andras Gaal, Anna Gershberg Hayoon, Veer Bala Gupta, Vivek Kumar Gupta, Simon I Hay, Claudiu Herteliu, Salman Hussain, Kevin S Ikuta, Irena M Ilic, Milena D Ilic, Tamas Joo, André Karch, Adnan Kisa, Sezer Kisa, Judit Lám, Graciliana Lopes, Alexander G Mathioudakis, Alexios-Fotios A Mentis, Tomislav Mestrovic, Irmina Maria Michalek, Mohammad Ali Moni, Catrin E Moore, Christopher J L Murray, Mohsen Naghavi, Ionut Negoi, Ruxandra Irina Negoi, Tamás Palicz, João Perdigão, Ionela-Roxana Petcu, Navid Rabiee, David Laith Rawaf, Salman Rawaf, Benn Sartorius, Murad Ziyaudinovich Shakhmardanov, Luís Manuel Lopes Rodrigues Silva, Valentin Yurievich Skryabin, Anna Aleksandrovna Skryabina, Bogdan Socea, Chandra Datta Sumi, Lucien R Swetschinski, Arulmani Thiyagarajan, Marcos Roberto Tovani-Palone, Eve E Wool, Metin Yesiltepe, and Sojib Bin Zaman.

Managing the estimation or publications process

Nicole Davis Weaver, Christiane Dolecek, Simon I Hay, Kevin S Ikuta, Christopher J L Murray, Mohsen Naghavi, Benn Sartorius, Lucien R Swetschinski, and Eve E Wool.
