# Supplementary material for: The burden of bacterial antimicrobial resistance in the WHO European region in 2019: a cross-country systematic analysis
Source: Lancet Public Health. 2022 Oct 14;7(11):e897–913. doi: 10.1016/S2468-2667(22)00225-0 (PMC9630253; doi:10.1016/S2468-2667(22)00225-0)
Supplement: Supplementary appendix 2 [file mmc2.pdf]

THE LANCET
Infectious Diseases

Supplementary appendix 2

This appendix formed part of the original submission and has been peer reviewed. We post it as supplied by the authors.

Supplement to: European Antimicrobial Resistance Collaborators. The burden of bacterial antimicrobial resistance in the WHO European region in 2019: a cross-country systematic analysis. *Lancet Infect Dis* 2022; published online Oct 13. https://doi.org/10.1016/S2468-2667(22)00225-0.

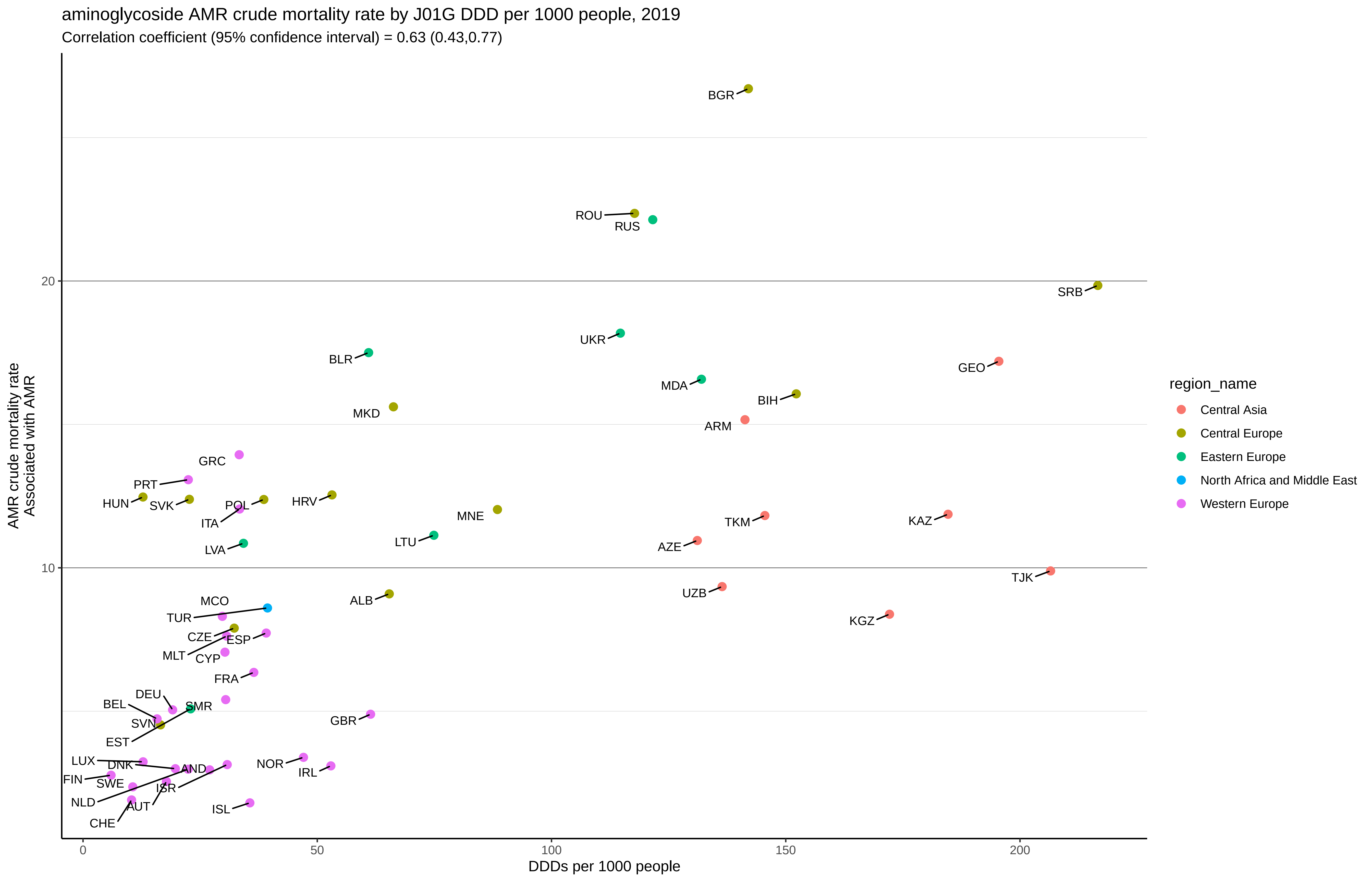
aminoglycoside AMR crude mortality rate by J01G DDD per 1000 people, 2019
Correlation coefficient (95% confidence interval) = 0.63 (0.43,0.77)
AMR crude mortality rate
Associated with AMR
DDDs per 1000 people
20
10
0
50
100
150
200
region_name
Central Asia
Central Europe
Eastern Europe
North Africa and Middle East
Western Europe
BGR
ROU
RUS
SRB
UKR
BLR
GEO
MDA
BIH
MKD
ARM
GRC
PRT
HUN
SVK
POL
HRV
ITA
MNE
TKM
KAZ
LVA
LTU
AZE
TJK
MCO
UZB
ALB
TUR
KGZ
CZE
ESP
MLT
CYP
FRA
DEU
BEL
SMR
SVN
GBR
EST
LUX
DNK
AND
NOR
IRL
FIN
SWE
ISR
NLD
AUT
ISL
CHE

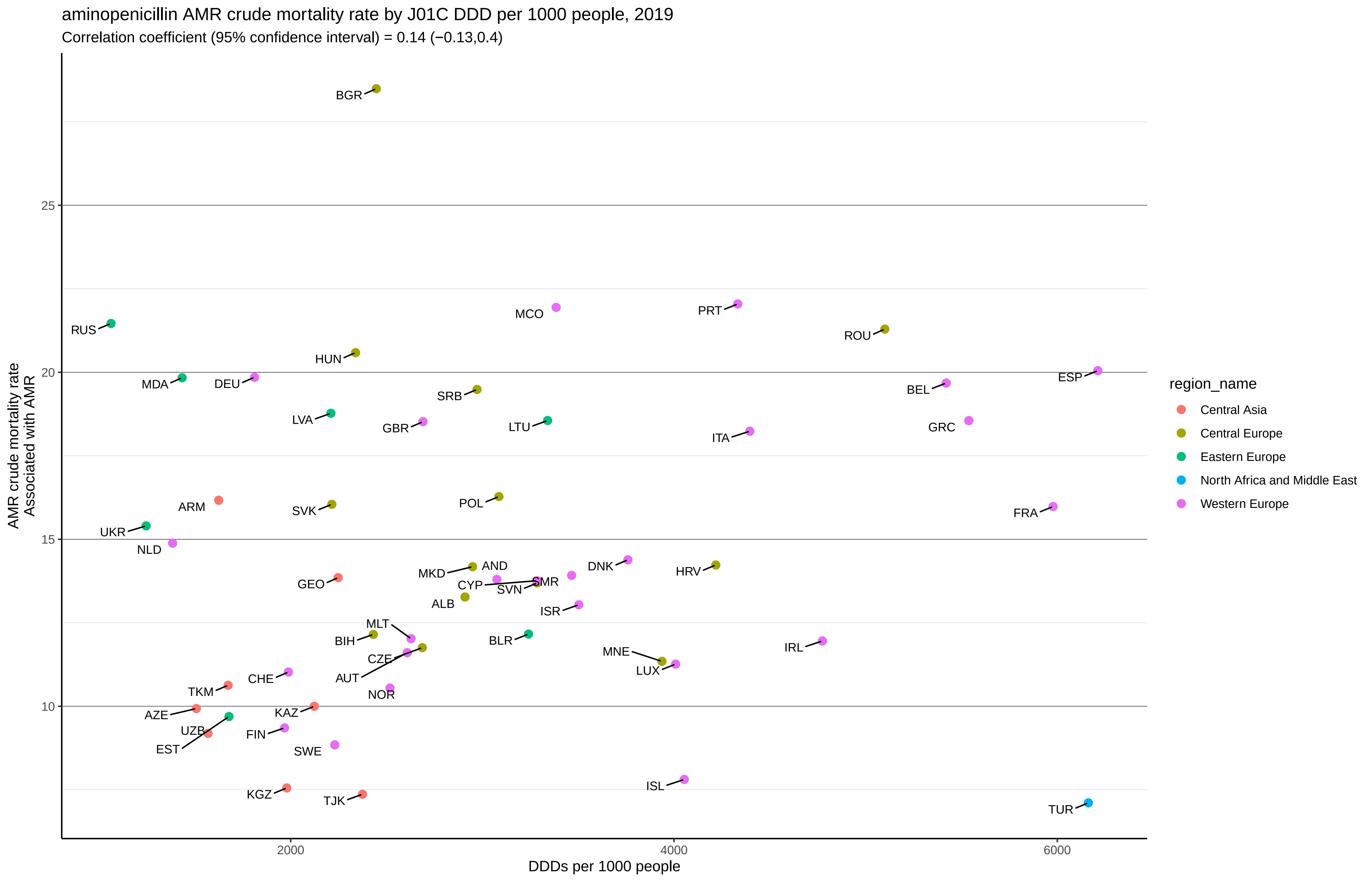

aminopenicillin AMR crude mortality rate by J01C DDD per 1000 people, 2019
Correlation coefficient (95% confidence interval) = 0.14 (−0.13,0.4)
AMR crude mortality rate
Associated with AMR
DDDs per 1000 people
25
20
15
10
2000
4000
6000
region_name
Central Asia
Central Europe
Eastern Europe
North Africa and Middle East
Western Europe
BGR
MCO
PRT
RUS
ROU
HUN
ESP
MDA
DEU
BEL
SRB
LVA
GBR
LTU
GRC
ITA
ARM
SVK
POL
FRA
UKR
NLD
AND
DNK
MKD
HRV
GEO
CYP
SMR
SVN
ALB
ISR
MLT
BIH
BLR
MNE
IRL
CZE
LUX
AUT
CHE
TKM
NOR
KAZ
AZE
UZB
FIN
EST
SWE
ISL
KGZ
TJK
TUR

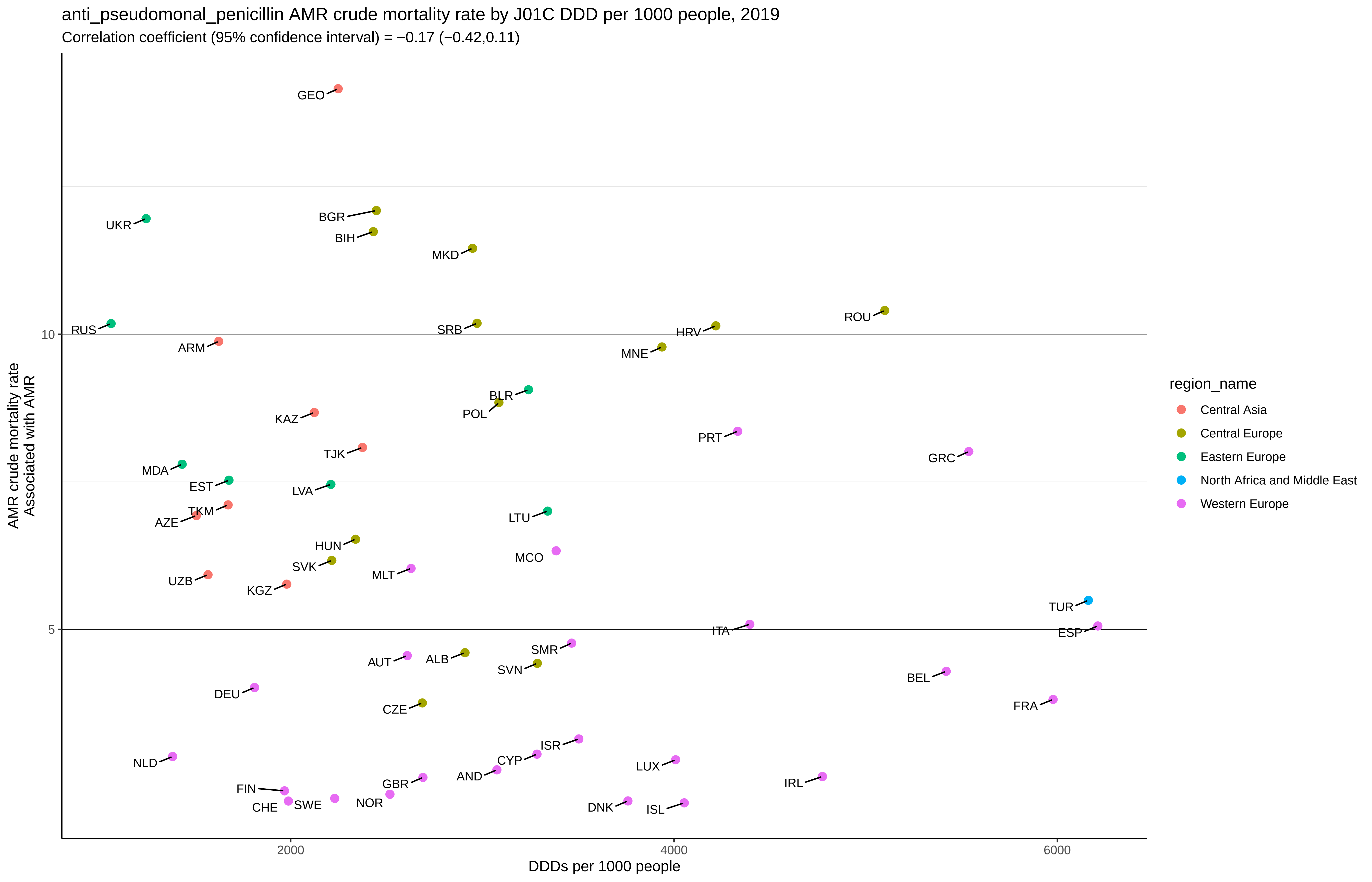
anti_pseudomonal_penicillin AMR crude mortality rate by J01C DDD per 1000 people, 2019
Correlation coefficient (95% confidence interval) = −0.17 (−0.42,0.11)
AMR crude mortality rate
Associated with AMR
DDDs per 1000 people
10
5
2000
4000
6000
region_name
Central Asia
Central Europe
Eastern Europe
North Africa and Middle East
Western Europe
GEO
UKR
BGR
BIH
MKD
RUS
SRB
ARM
HRV
MNE
ROU
BLR
POL
KAZ
PRT
TJK
GRC
MDA
EST
LVA
TKM
AZE
LTU
HUN
MCO
SVK
MLT
UZB
KGZ
TUR
ITA
ESP
SMR
AUT
ALB
SVN
BEL
DEU
FRA
CZE
ISR
NLD
CYP
LUX
AND
IRL
GBR
FIN
CHE
SWE
NOR
DNK
ISL

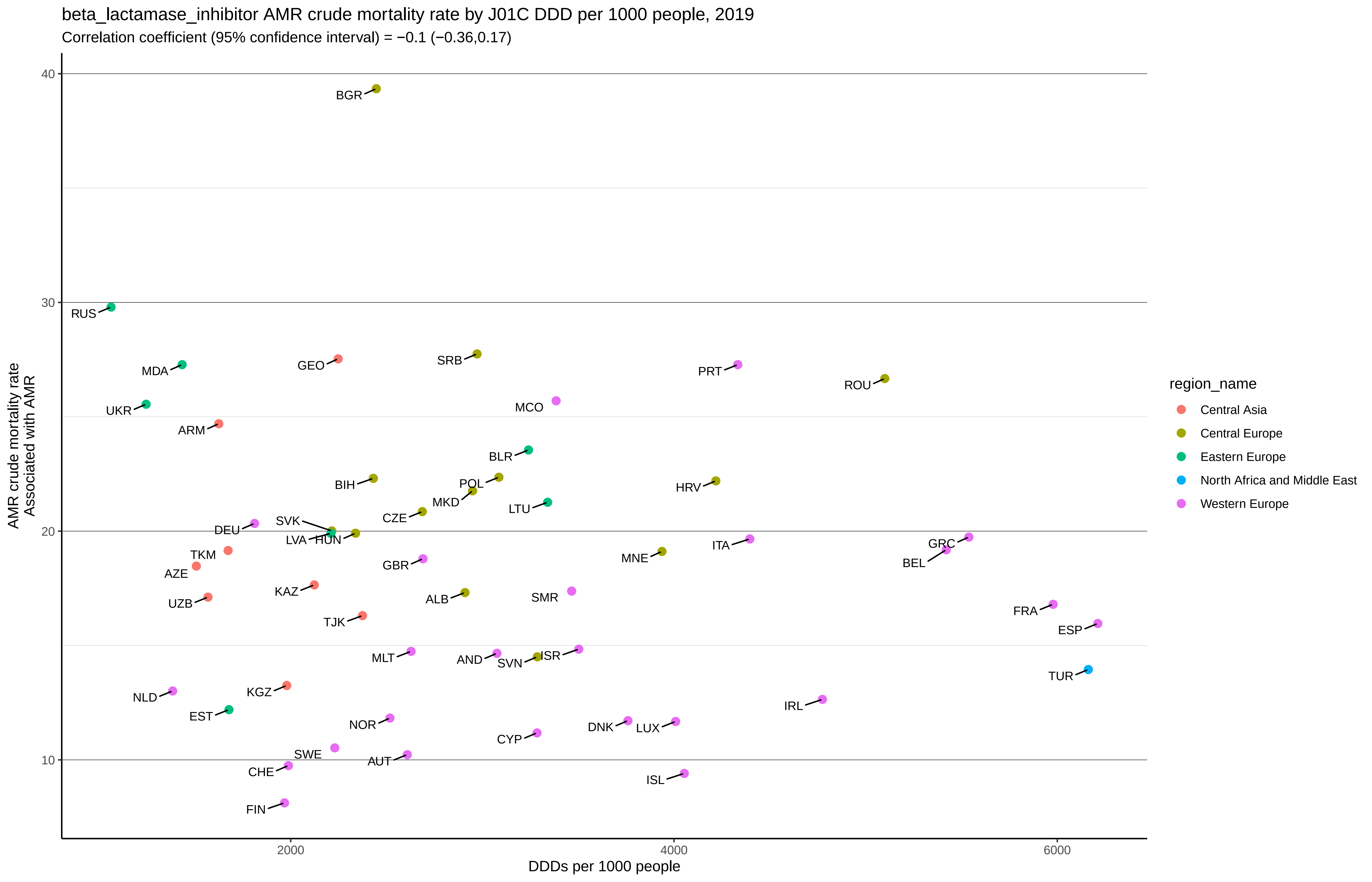
beta_lactamase_inhibitor AMR crude mortality rate by J01C DDD per 1000 people, 2019
Correlation coefficient (95% confidence interval) = −0.1 (−0.36,0.17)
AMR crude mortality rate
Associated with AMR
DDDs per 1000 people
40
30
20
10
2000
4000
6000
region_name
Central Asia
Central Europe
Eastern Europe
North Africa and Middle East
Western Europe
BGR
RUS
MDA
GEO
SRB
PRT
ROU
UKR
MCO
ARM
BLR
BIH
POL
HRV
MKD
LTU
CZE
SVK
DEU
LVA
HUN
ITA
GRC
TKM
MNE
BEL
GBR
AZE
KAZ
SMR
ALB
UZB
FRA
TJK
ESP
MLT
AND
SVN
ISR
TUR
NLD
KGZ
IRL
EST
NOR
DNK
LUX
CYP
SWE
AUT
CHE
ISL
FIN

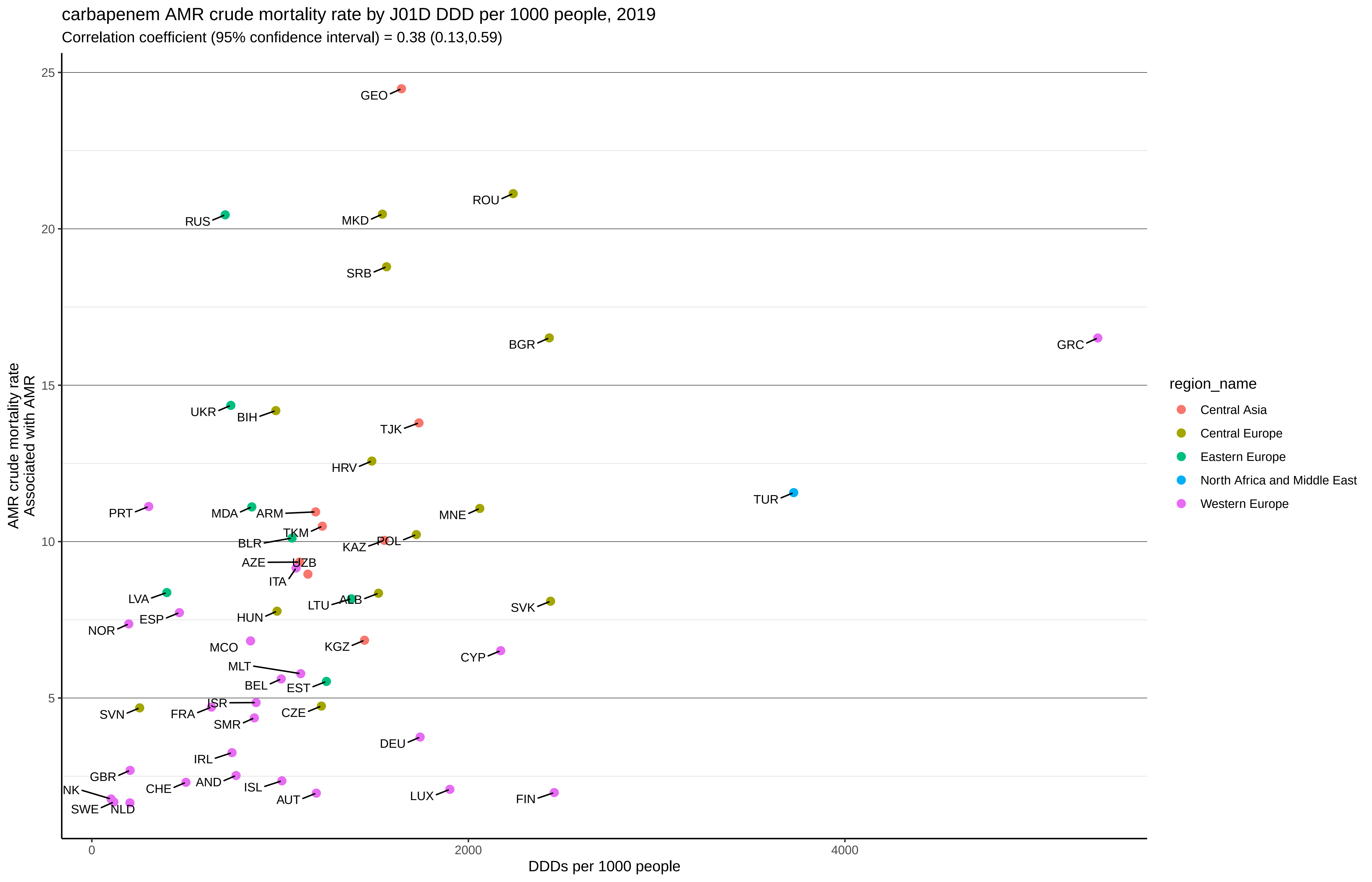

carbapenem AMR crude mortality rate by J01D DDD per 1000 people, 2019
Correlation coefficient (95% confidence interval) = 0.38 (0.13,0.59)
AMR crude mortality rate
Associated with AMR
DDDs per 1000 people
0
2000
4000
5
10
15
20
25
region_name
Central Asia
Central Europe
Eastern Europe
North Africa and Middle East
Western Europe
GEO
ROU
RUS
MKD
SRB
BGR
GRC
UKR
BIH
TJK
HRV
TUR
PRT
MDA
ARM
MNE
TKM
BLR
KAZ
POL
AZE
UZB
ITA
LVA
LTU
ALB
SVK
ESP
HUN
NOR
MCO
KGZ
CYP
MLT
BEL
EST
ISR
SVN
FRA
CZE
SMR
DEU
IRL
GBR
AND
CHE
ISL
NK
LUX
FIN
AUT
SWE
NLD

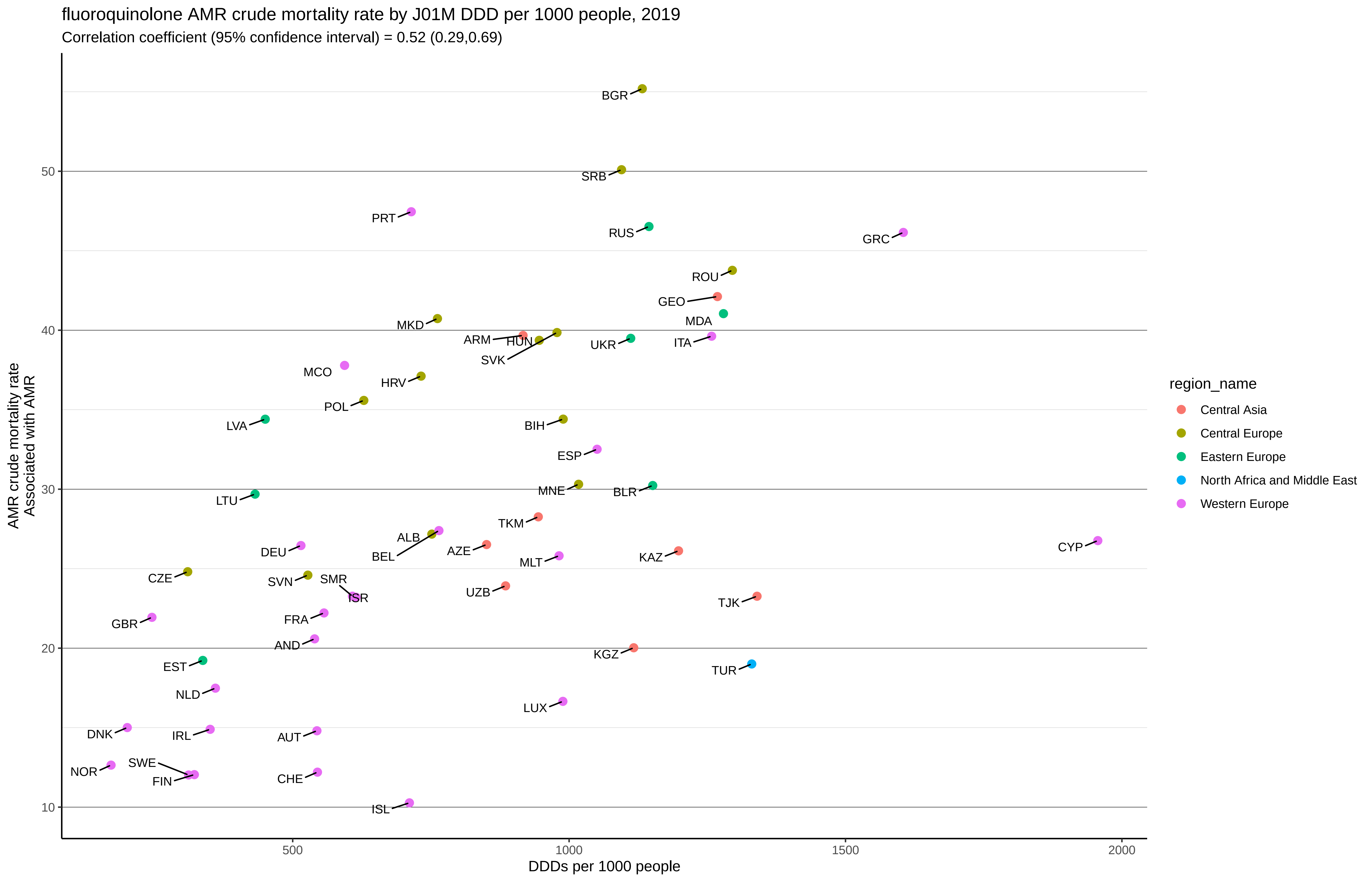

fluoroquinolone AMR crude mortality rate by J01M DDD per 1000 people, 2019
Correlation coefficient (95% confidence interval) = 0.52 (0.29,0.69)
AMR crude mortality rate
Associated with AMR
DDDs per 1000 people
region_name
Central Asia
Central Europe
Eastern Europe
North Africa and Middle East
Western Europe
BGR
SRB
PRT
RUS
GRC
ROU
GEO
MDA
MKD
ARM
HUN
SVK
UKR
ITA
MCO
HRV
POL
LVA
BIH
ESP
LTU
MNE
BLR
TKM
ALB
BEL
DEU
AZE
MLT
KAZ
CYP
CZE
SVN
SMR
ISR
UZB
TJK
GBR
FRA
AND
KGZ
TUR
EST
NLD
LUX
DNK
IRL
AUT
NOR
SWE
FIN
CHE
ISL

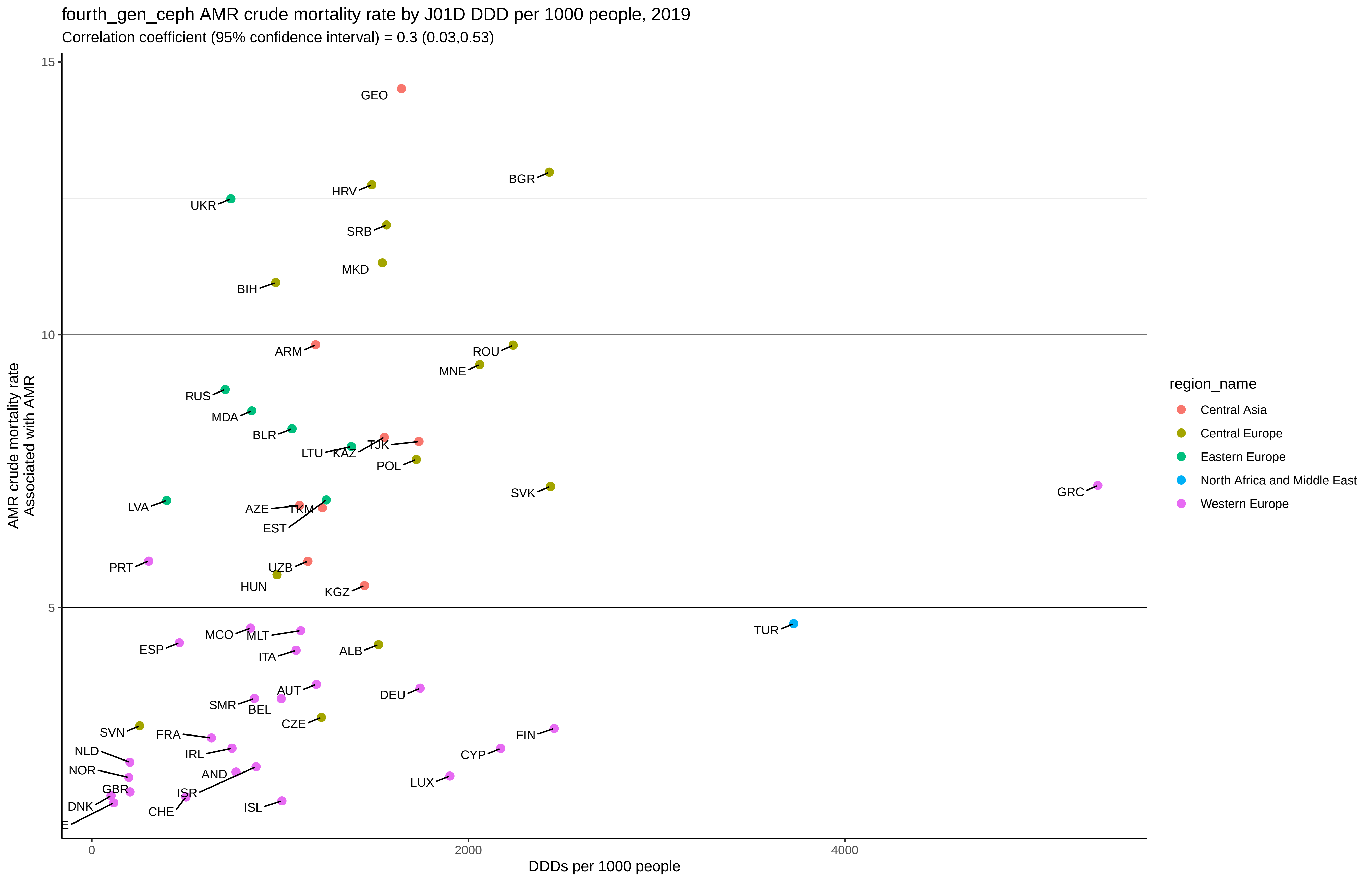
fourth_gen_ceph AMR crude mortality rate by J01D DDD per 1000 people, 2019
Correlation coefficient (95% confidence interval) = 0.3 (0.03,0.53)
AMR crude mortality rate
Associated with AMR
DDDs per 1000 people
15
10
5
0
2000
4000
region_name
Central Asia
Central Europe
Eastern Europe
North Africa and Middle East
Western Europe
GEO
BGR
HRV
UKR
SRB
MKD
BIH
ARM
ROU
MNE
RUS
MDA
BLR
TJK
LTU
KAZ
POL
SVK
GRC
LVA
AZE
TKM
EST
PRT
UZB
HUN
KGZ
TUR
MCO
MLT
ESP
ITA
ALB
AUT
DEU
SMR
BEL
CZE
SVN
FRA
FIN
NLD
IRL
CYP
NOR
AND
LUX
GBR
ISR
DNK
CHE
ISL
E

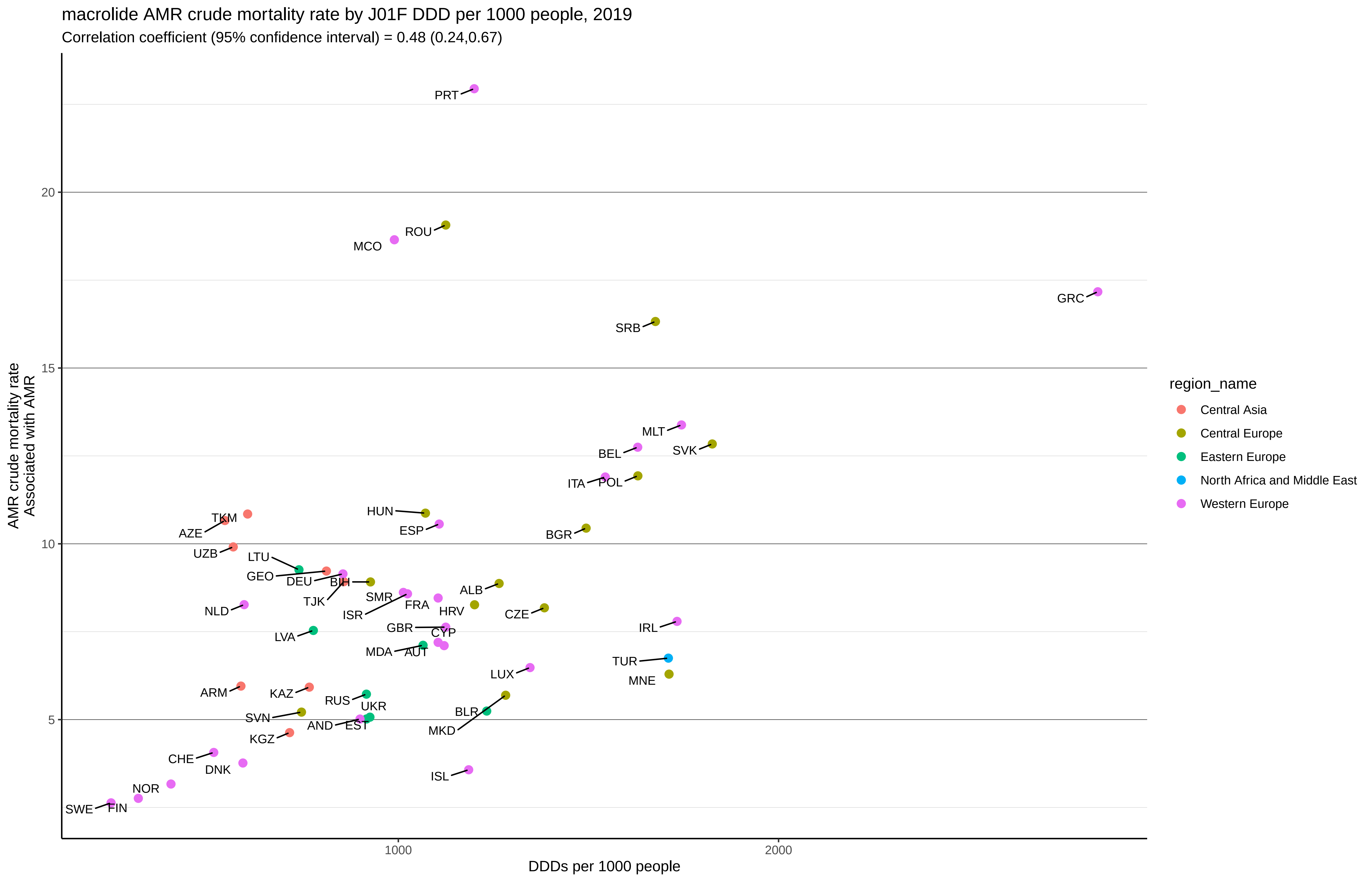
macrolide AMR crude mortality rate by J01F DDD per 1000 people, 2019
Correlation coefficient (95% confidence interval) = 0.48 (0.24,0.67)
AMR crude mortality rate
Associated with AMR
DDDs per 1000 people
20
15
10
5
1000
2000
region_name
Central Asia
Central Europe
Eastern Europe
North Africa and Middle East
Western Europe
PRT
ROU
MCO
GRC
SRB
MLT
BEL
SVK
ITA
POL
TKM
AZE
HUN
ESP
BGR
UZB
LTU
GEO
DEU
BIH
TJK
SMR
FRA
ALB
ISR
HRV
CZE
NLD
GBR
CYP
IRL
LVA
MDA
AUT
TUR
LUX
MNE
ARM
KAZ
RUS
UKR
BLR
SVN
AND
EST
MKD
KGZ
CHE
DNK
ISL
NOR
SWE
FIN

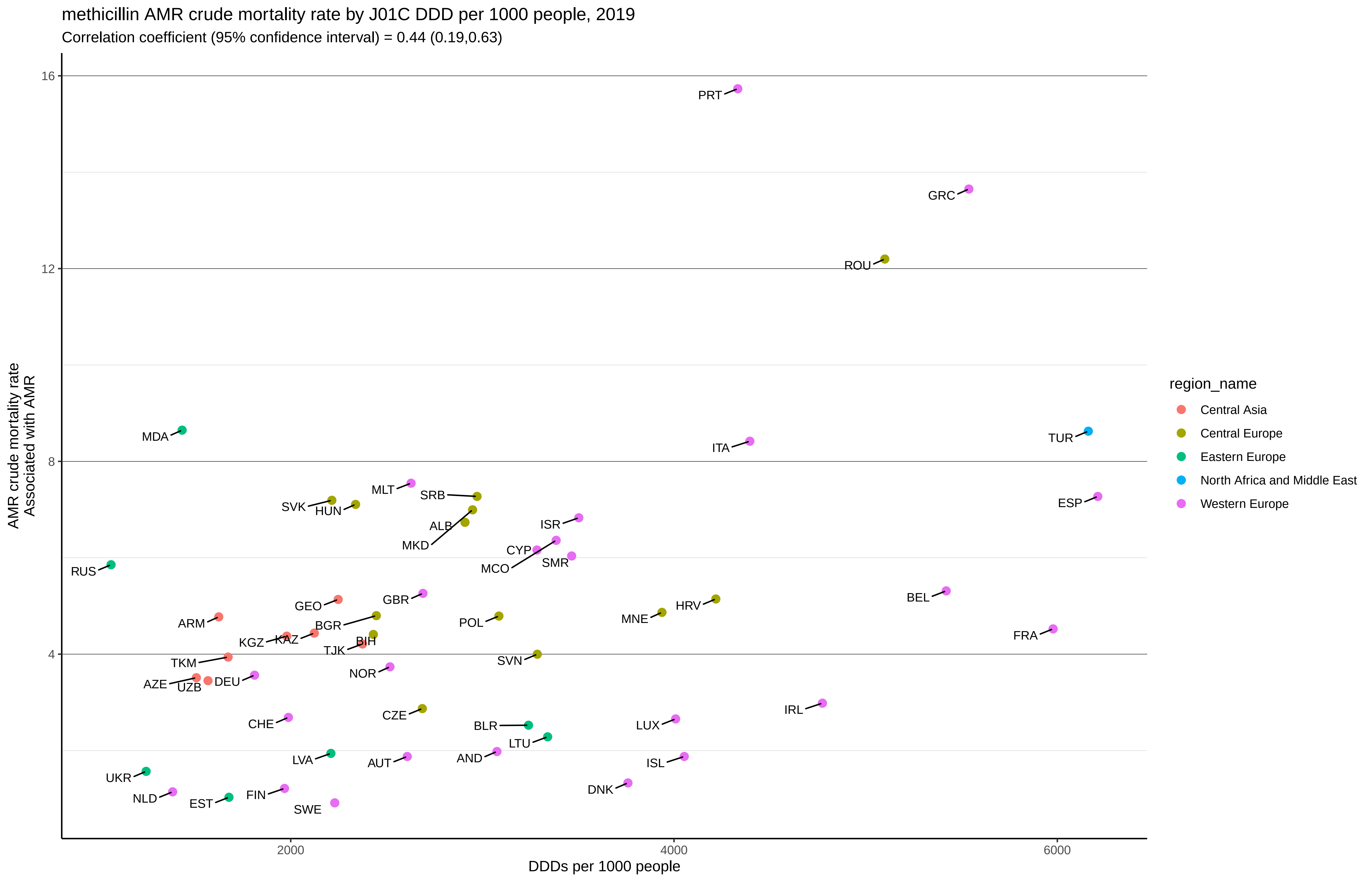
methicillin AMR crude mortality rate by J01C DDD per 1000 people, 2019
Correlation coefficient (95% confidence interval) = 0.44 (0.19,0.63)
AMR crude mortality rate
Associated with AMR
DDDs per 1000 people
16
12
8
4
2000
4000
6000
region_name
Central Asia
Central Europe
Eastern Europe
North Africa and Middle East
Western Europe
PRT
GRC
ROU
MDA
ITA
TUR
MLT
SRB
SVK
HUN
ALB
MKD
ISR
CYP
MCO
SMR
ESP
RUS
GEO
GBR
ARM
BGR
KGZ
KAZ
BIH
TJK
POL
MNE
HRV
BEL
FRA
TKM
SVN
AZE
UZB
DEU
NOR
CHE
CZE
BLR
LTU
LUX
IRL
LVA
AUT
AND
ISL
UKR
NLD
EST
FIN
SWE
DNK

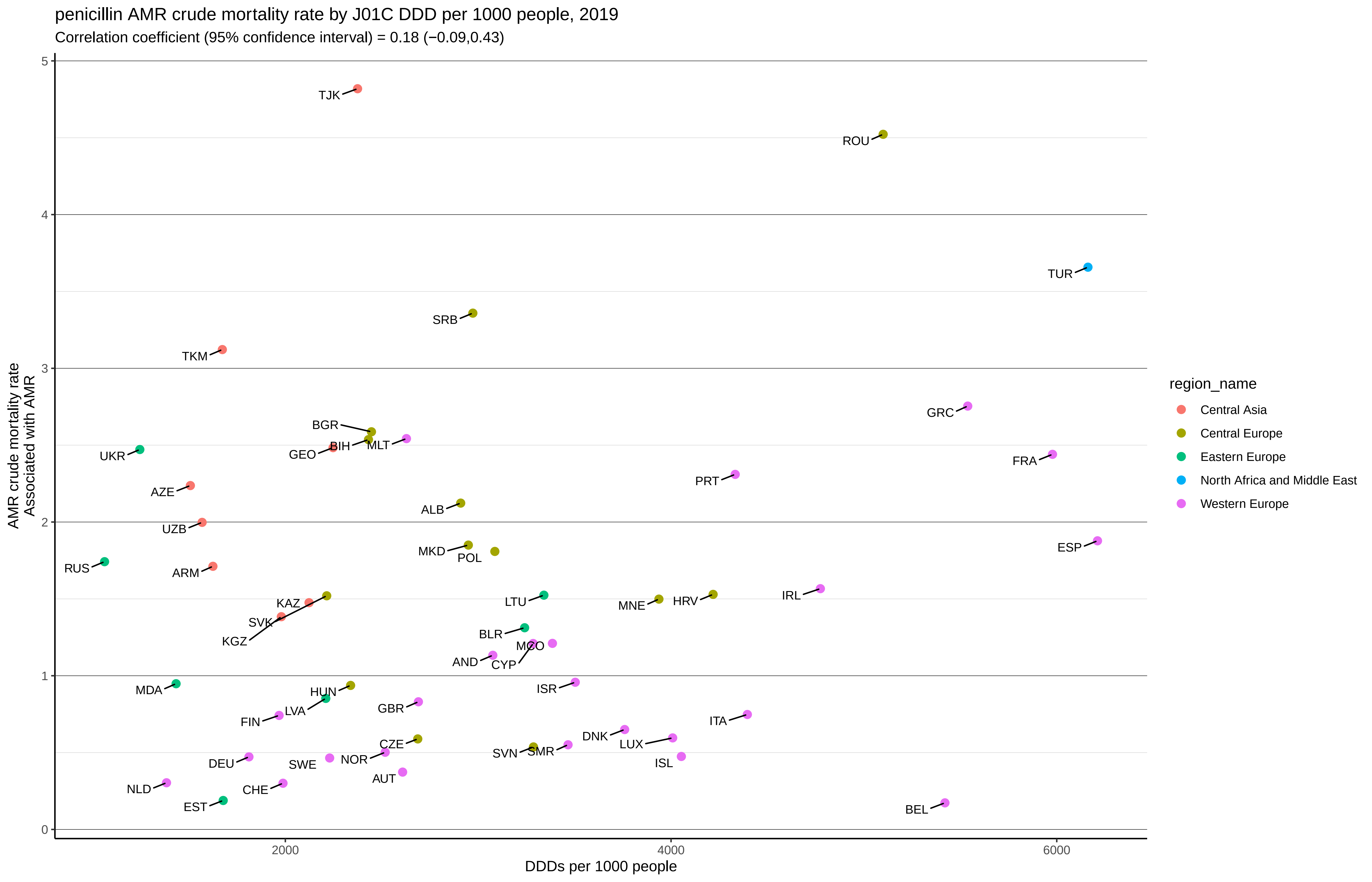
penicillin AMR crude mortality rate by J01C DDD per 1000 people, 2019
Correlation coefficient (95% confidence interval) = 0.18 (−0.09,0.43)
AMR crude mortality rate
Associated with AMR
DDDs per 1000 people
0
1
2
3
4
5
2000
4000
6000
region_name
Central Asia
Central Europe
Eastern Europe
North Africa and Middle East
Western Europe
TJK
ROU
TUR
SRB
TKM
GRC
BGR
BIH
MLT
UKR
GEO
FRA
PRT
AZE
ALB
UZB
ESP
MKD
POL
RUS
ARM
KAZ
LTU
MNE
HRV
IRL
SVK
KGZ
BLR
MCO
AND
CYP
MDA
ISR
HUN
GBR
LVA
FIN
ITA
DNK
LUX
CZE
SVN
SMR
ISL
DEU
SWE
NOR
AUT
NLD
CHE
EST
BEL

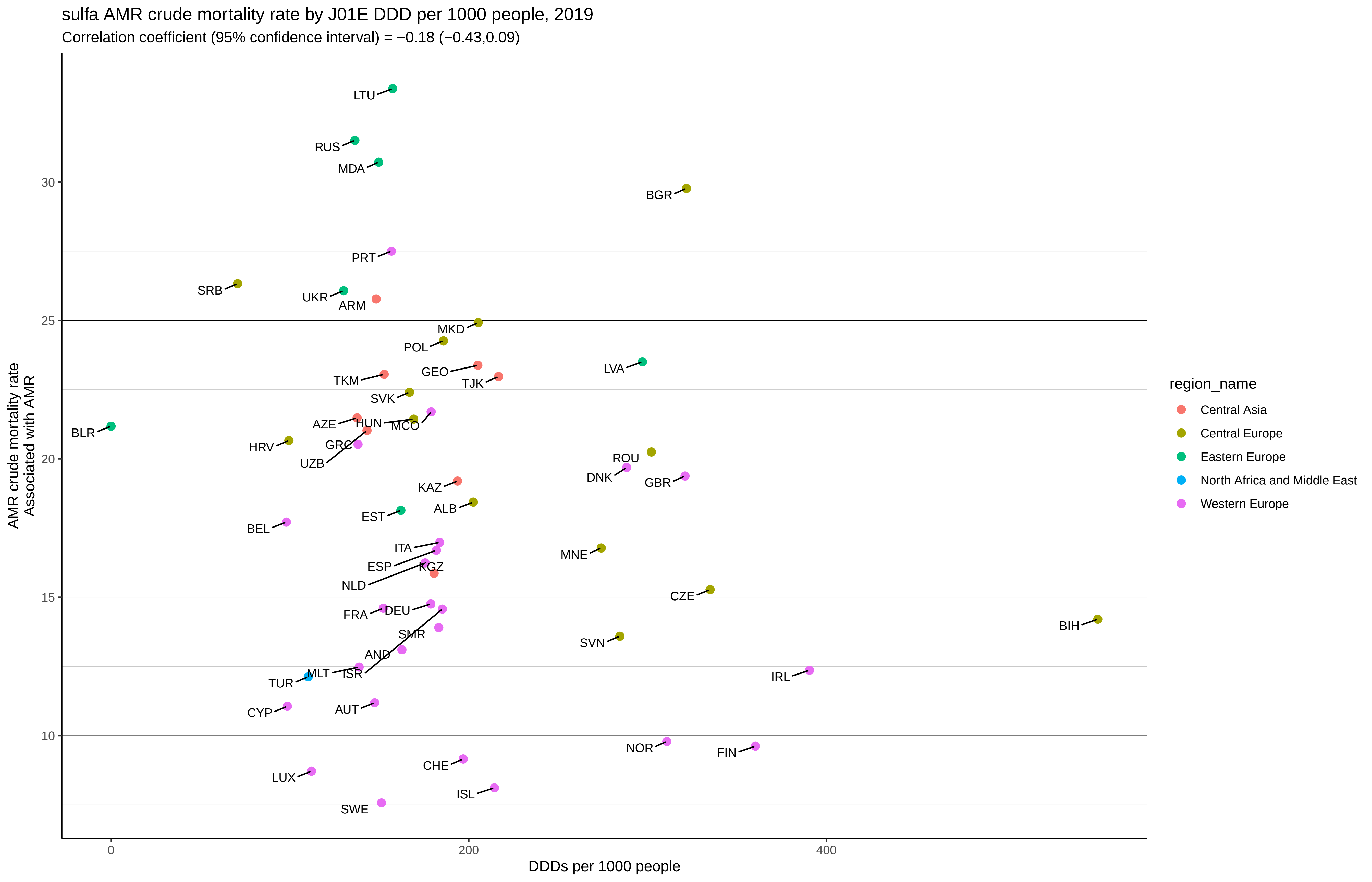
sulfa AMR crude mortality rate by J01E DDD per 1000 people, 2019
Correlation coefficient (95% confidence interval) = −0.18 (−0.43,0.09)
AMR crude mortality rate
Associated with AMR
DDDs per 1000 people
0
200
400
10
15
20
25
30
region_name
Central Asia
Central Europe
Eastern Europe
North Africa and Middle East
Western Europe
LTU
RUS
MDA
BGR
PRT
SRB
UKR
ARM
MKD
POL
GEO
TJK
LVA
TKM
SVK
AZE
HUN
MCO
BLR
HRV
GRC
UZB
ROU
DNK
GBR
KAZ
ALB
EST
BEL
ITA
ESP
KGZ
NLD
MNE
CZE
FRA
DEU
BIH
SMR
SVN
AND
MLT
ISR
TUR
IRL
CYP
AUT
NOR
FIN
CHE
LUX
ISL
SWE

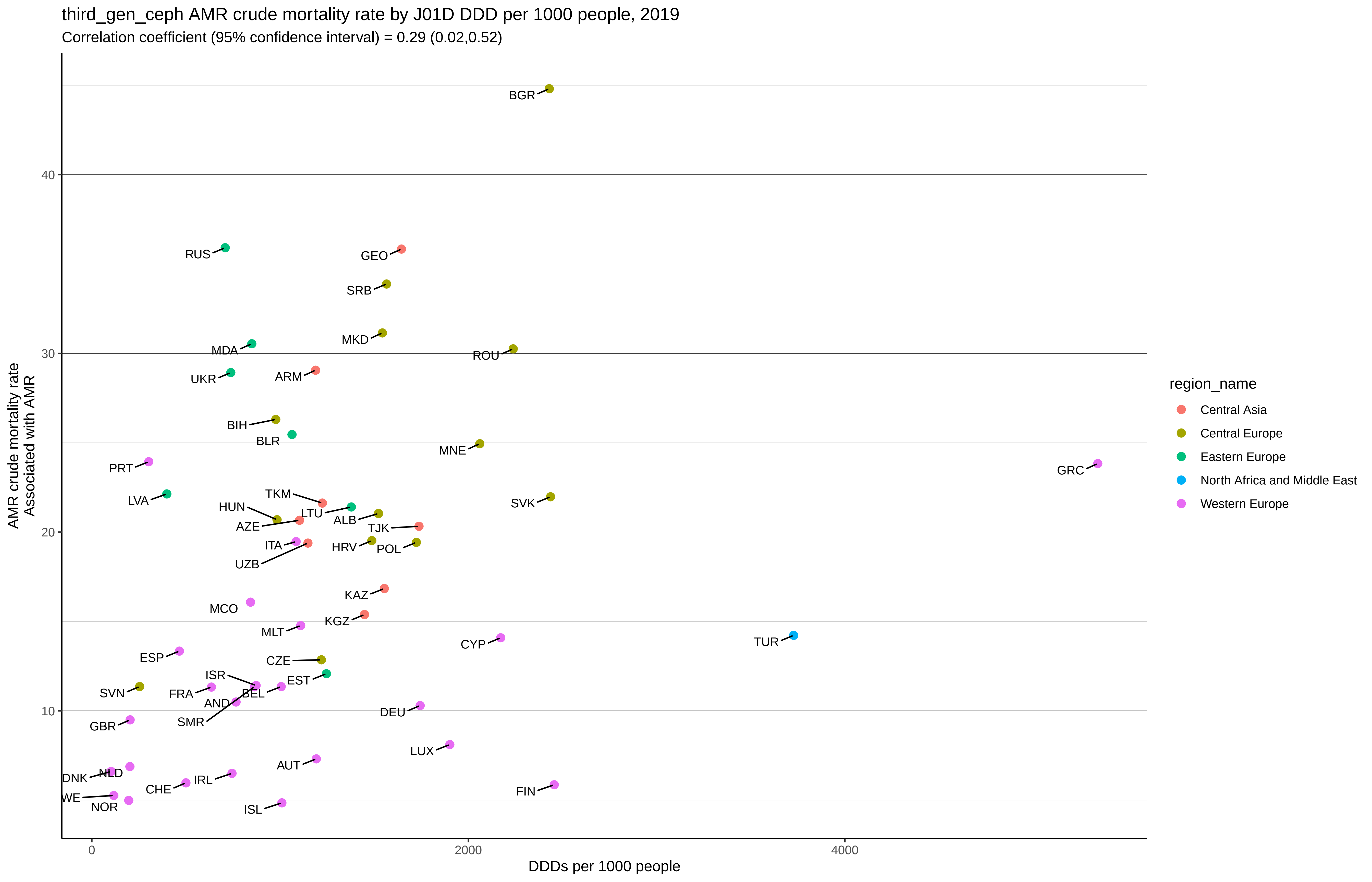

third_gen_ceph AMR crude mortality rate by J01D DDD per 1000 people, 2019
Correlation coefficient (95% confidence interval) = 0.29 (0.02,0.52)
AMR crude mortality rate
Associated with AMR
DDDs per 1000 people
0
2000
4000
10
20
30
40
region_name
Central Asia
Central Europe
Eastern Europe
North Africa and Middle East
Western Europe
BGR
RUS
GEO
SRB
MKD
MDA
ROU
UKR
ARM
BIH
BLR
MNE
PRT
GRC
TKM
LVA
SVK
HUN
LTU
ALB
AZE
TJK
ITA
HRV
POL
UZB
KAZ
MCO
KGZ
MLT
TUR
CYP
ESP
CZE
ISR
EST
SVN
FRA
BEL
AND
DEU
SMR
GBR
LUX
AUT
DNK
NLD
IRL
CHE
FIN
SWE
NOR
ISL

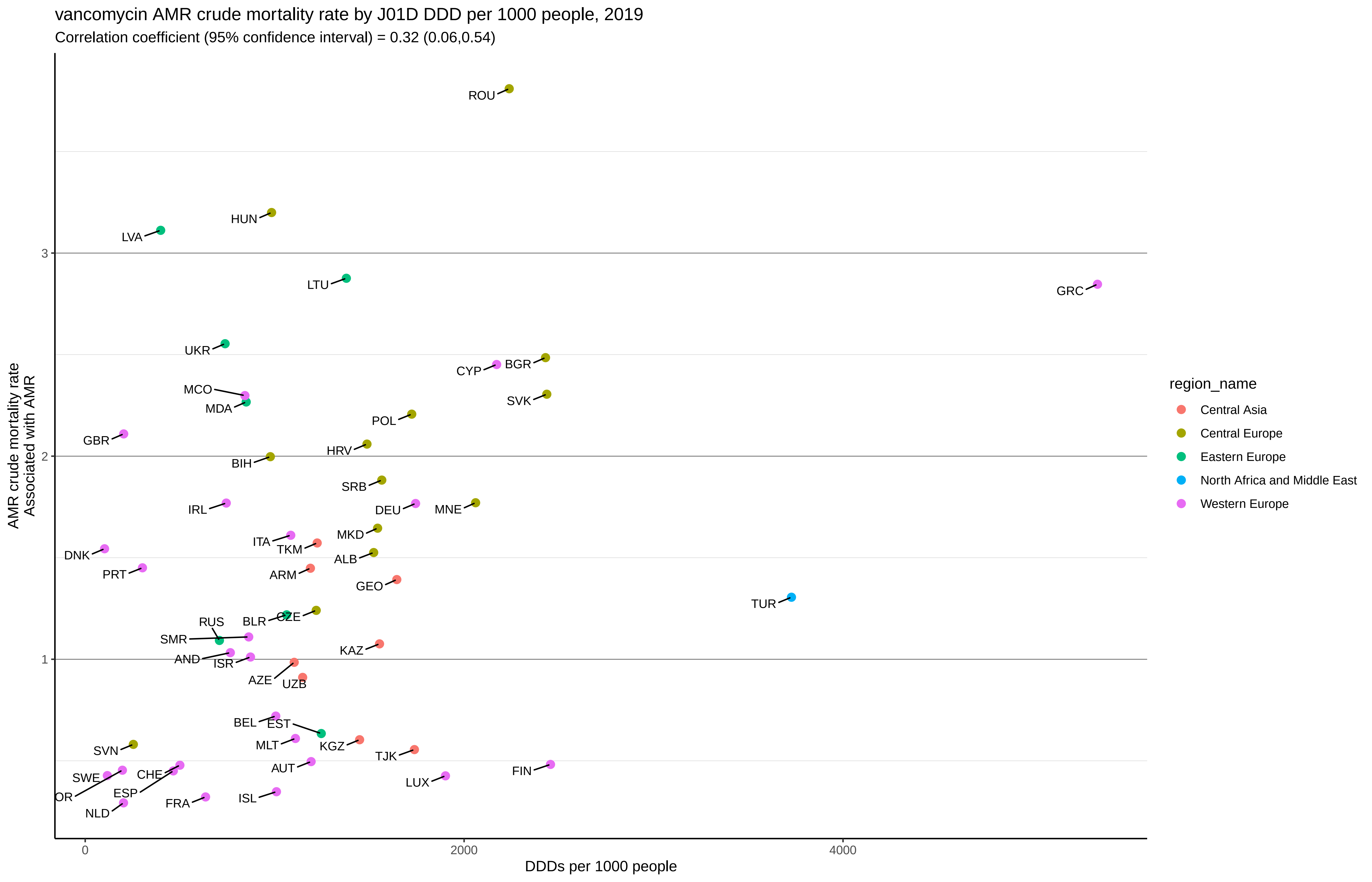

vancomycin AMR crude mortality rate by J01D DDD per 1000 people, 2019
Correlation coefficient (95% confidence interval) = 0.32 (0.06,0.54)
AMR crude mortality rate
Associated with AMR
DDDs per 1000 people
0
2000
4000
1
2
3
region_name
Central Asia
Central Europe
Eastern Europe
North Africa and Middle East
Western Europe
ROU
HUN
LVA
LTU
GRC
UKR
CYP
BGR
MCO
MDA
SVK
POL
GBR
HRV
BIH
SRB
IRL
DEU
MNE
MKD
ITA
TKM
DNK
ALB
PRT
ARM
GEO
TUR
RUS
BLR
CZE
SMR
KAZ
AND
ISR
AZE
UZB
BEL
EST
MLT
KGZ
SVN
TJK
AUT
SWE
CHE
FIN
LUX
OR
ESP
FRA
ISL
NLD

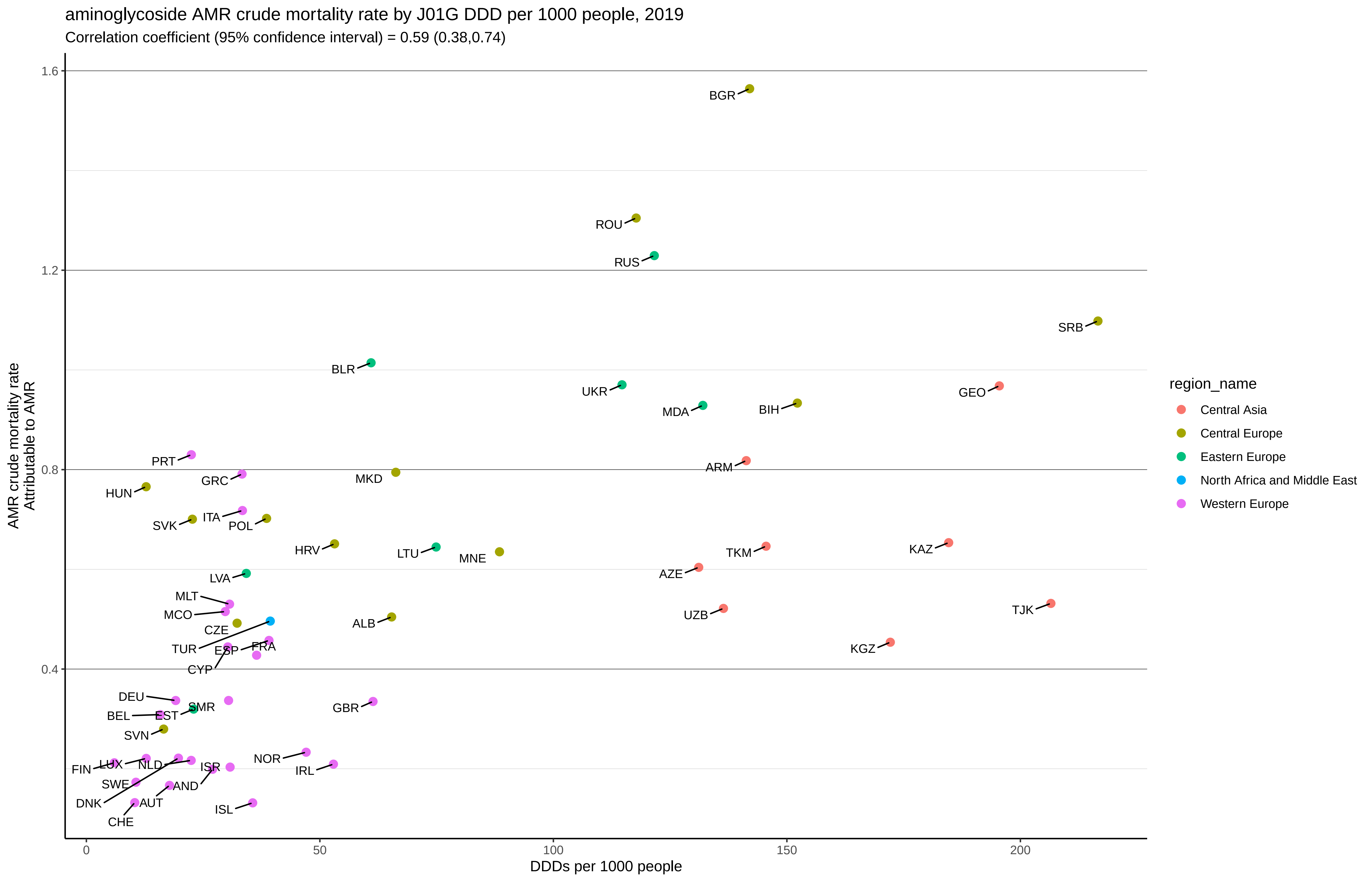

aminoglycoside AMR crude mortality rate by J01G DDD per 1000 people, 2019
Correlation coefficient (95% confidence interval) = 0.59 (0.38,0.74)
AMR crude mortality rate
Attributable to AMR
DDDs per 1000 people
1.6
1.2
0.8
0.4
0
50
100
150
200
region_name
Central Asia
Central Europe
Eastern Europe
North Africa and Middle East
Western Europe
BGR
ROU
RUS
SRB
BLR
UKR
MDA
BIH
GEO
PRT
GRC
MKD
ARM
HUN
SVK
ITA
POL
HRV
LTU
MNE
TKM
KAZ
AZE
LVA
MLT
MCO
CZE
ALB
UZB
TJK
TUR
ESP
FRA
KGZ
CYP
DEU
SMR
GBR
BEL
EST
SVN
NOR
FIN
LUX
NLD
ISR
IRL
SWE
AND
DNK
AUT
ISL
CHE

aminopenicillin AMR crude mortality rate by J01C DDD per 1000 people, 2019

Correlation coefficient (95% confidence interval) = 0.46 (0.22,0.65)

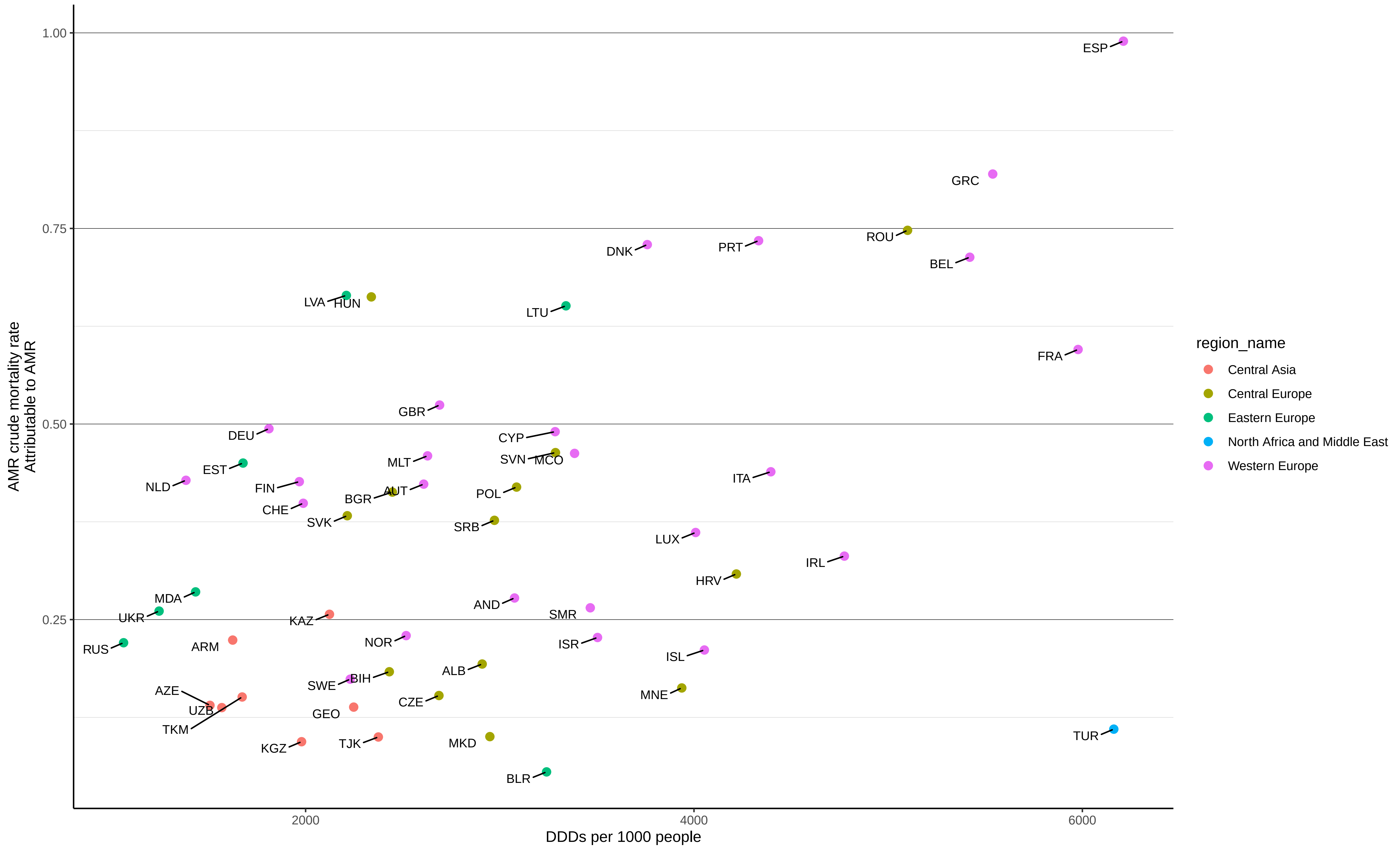

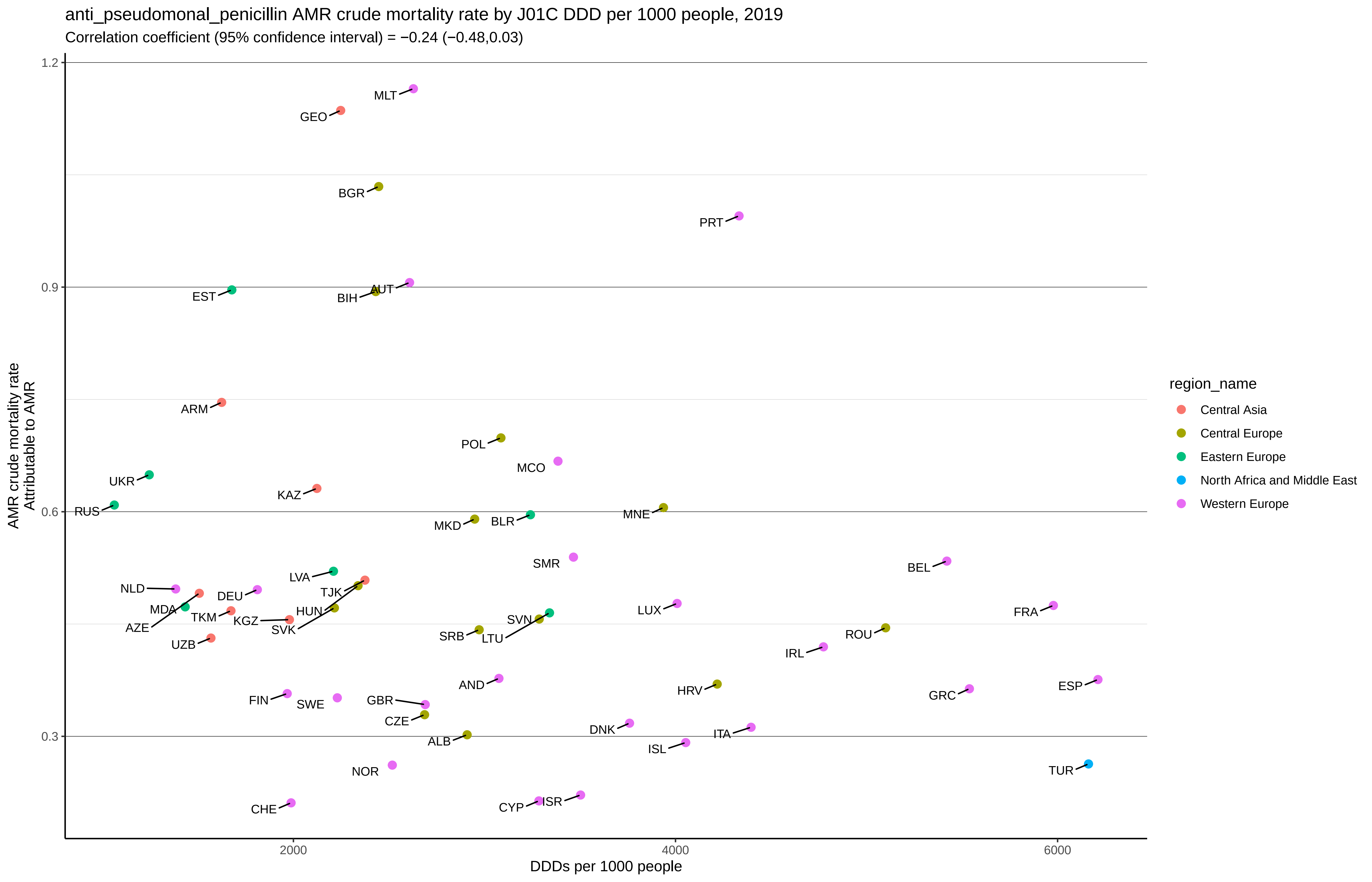

anti_pseudomonal_penicillin AMR crude mortality rate by J01C DDD per 1000 people, 2019
Correlation coefficient (95% confidence interval) = −0.24 (−0.48,0.03)
AMR crude mortality rate
Attributable to AMR
1.2
0.9
0.6
0.3
2000
4000
6000
DDDs per 1000 people
MLT
GEO
BGR
PRT
EST
BIH
AUT
ARM
POL
MCO
UKR
RUS
KAZ
MKD
BLR
MNE
SMR
BEL
LVA
NLD
DEU
TJK
MDA
TKM
HUN
KGZ
AZE
SVK
LUX
FRA
SVN
SRB
LTU
ROU
UZB
IRL
AND
HRV
ESP
FIN
SWE
GBR
GRC
CZE
DNK
ITA
ALB
ISL
NOR
TUR
CHE
CYP
ISR
region_name
Central Asia
Central Europe
Eastern Europe
North Africa and Middle East
Western Europe

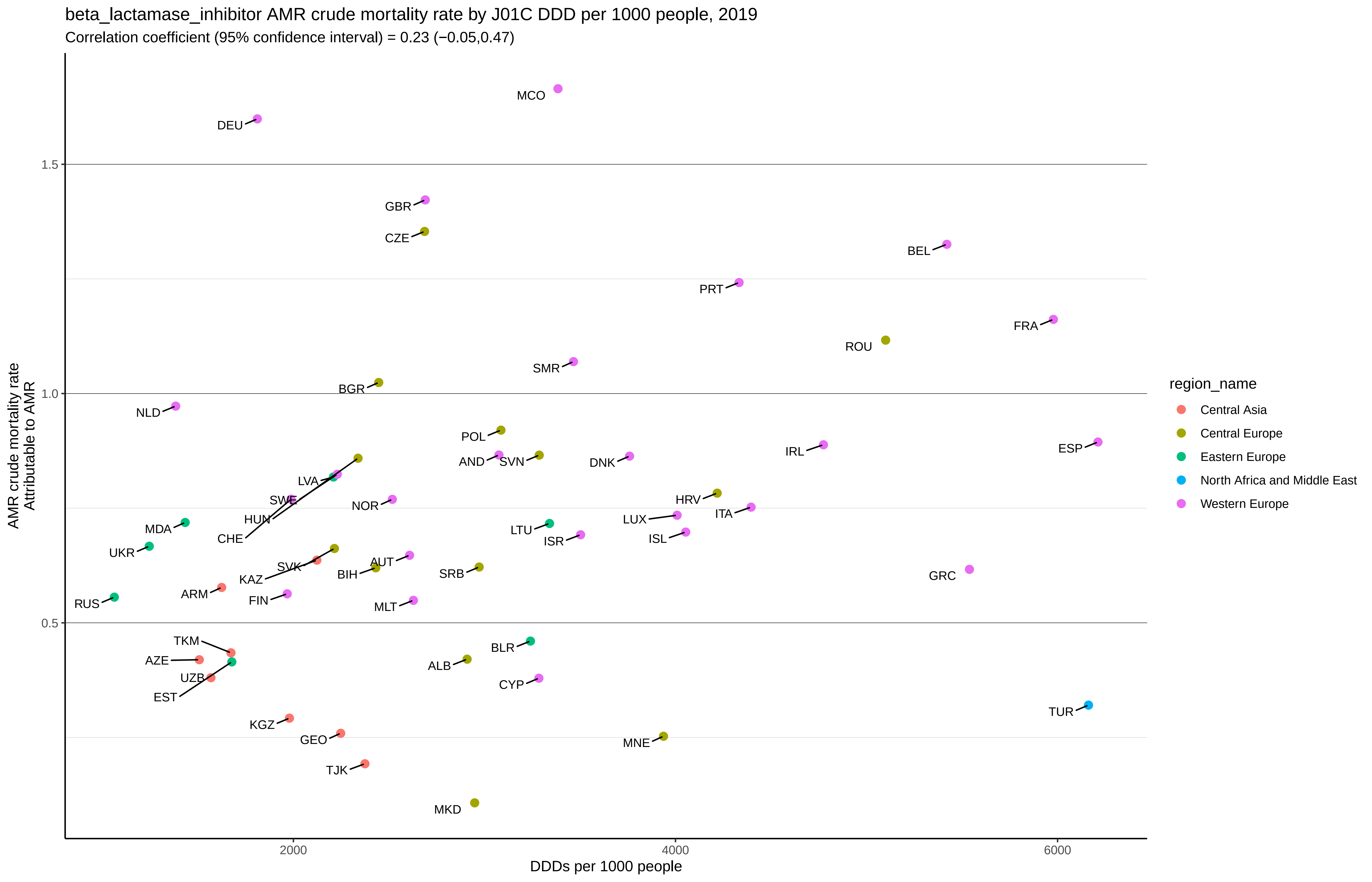

beta_lactamase_inhibitor AMR crude mortality rate by J01C DDD per 1000 people, 2019
Correlation coefficient (95% confidence interval) = 0.23 (−0.05,0.47)
AMR crude mortality rate
Attributable to AMR
DDDs per 1000 people
1.5
1.0
0.5
2000
4000
6000
region_name
Central Asia
Central Europe
Eastern Europe
North Africa and Middle East
Western Europe
MCO
DEU
GBR
CZE
BEL
PRT
FRA
ROU
SMR
BGR
NLD
POL
AND
SVN
DNK
IRL
ESP
LVA
SWE
NOR
HRV
HUN
LUX
ITA
MDA
CHE
LTU
ISR
ISL
UKR
AUT
SVK
BIH
SRB
GRC
KAZ
ARM
FIN
MLT
RUS
TKM
BLR
AZE
ALB
UZB
CYP
EST
TUR
KGZ
GEO
MNE
TJK
MKD

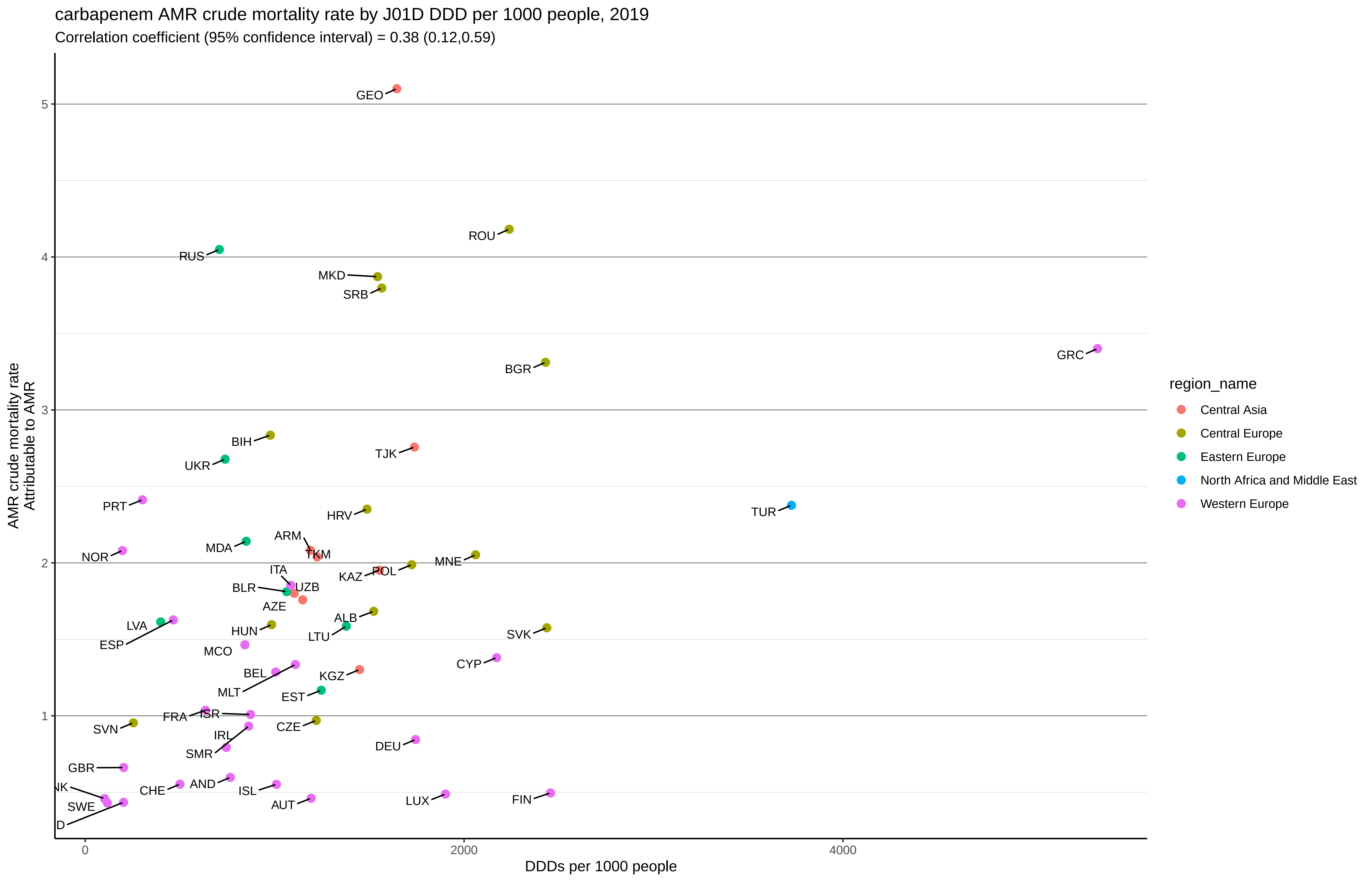
carbapenem AMR crude mortality rate by J01D DDD per 1000 people, 2019
Correlation coefficient (95% confidence interval) = 0.38 (0.12,0.59)
AMR crude mortality rate
Attributable to AMR
DDDs per 1000 people
0
2000
4000
1
2
3
4
5
region_name
Central Asia
Central Europe
Eastern Europe
North Africa and Middle East
Western Europe
GEO
ROU
RUS
MKD
SRB
GRC
BGR
BIH
TJK
UKR
PRT
HRV
TUR
ARM
MDA
TKM
NOR
MNE
ITA
KAZ
POL
BLR
UZB
AZE
ALB
LVA
HUN
LTU
SVK
ESP
MCO
CYP
BEL
KGZ
MLT
EST
FRA
ISR
SVN
CZE
IRL
SMR
DEU
GBR
AND
CHE
ISL
SWE
AUT
LUX
FIN

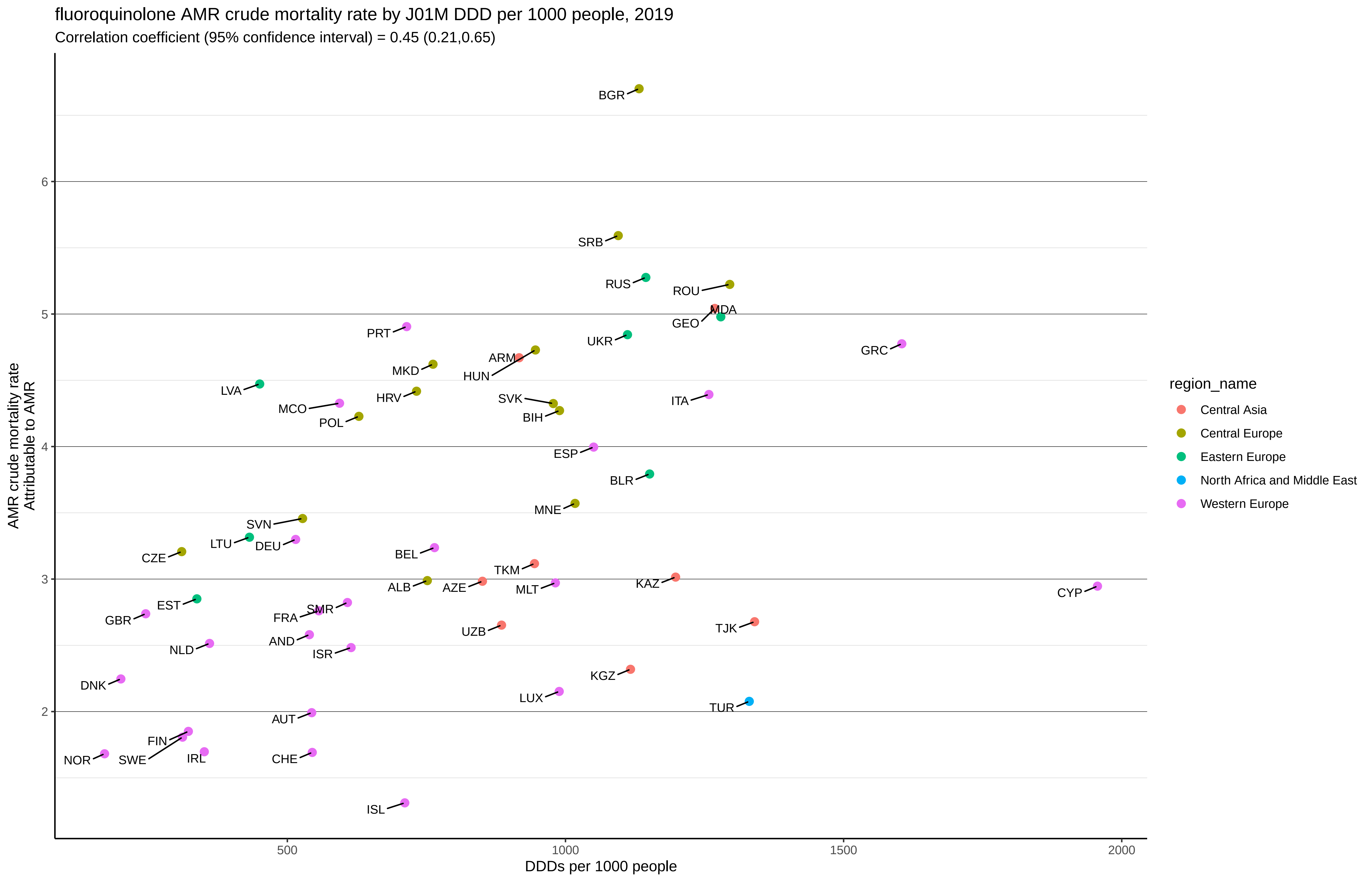

fluoroquinolone AMR crude mortality rate by J01M DDD per 1000 people, 2019
Correlation coefficient (95% confidence interval) = 0.45 (0.21,0.65)
AMR crude mortality rate
Attributable to AMR
DDDs per 1000 people
6
5
4
3
2
500
1000
1500
2000
BGR
SRB
RUS
ROU
MDA
GEO
UKR
GRC
PRT
ARM
MKD
HUN
LVA
HRV
SVK
ITA
MCO
POL
BIH
ESP
BLR
MNE
SVN
LTU
DEU
CZE
BEL
TKM
ALB
AZE
MLT
KAZ
CYP
EST
SMR
GBR
FRA
UZB
TJK
AND
NLD
ISR
DNK
KGZ
LUX
TUR
AUT
FIN
NOR
SWE
IRL
CHE
ISL
region_name
Central Asia
Central Europe
Eastern Europe
North Africa and Middle East
Western Europe

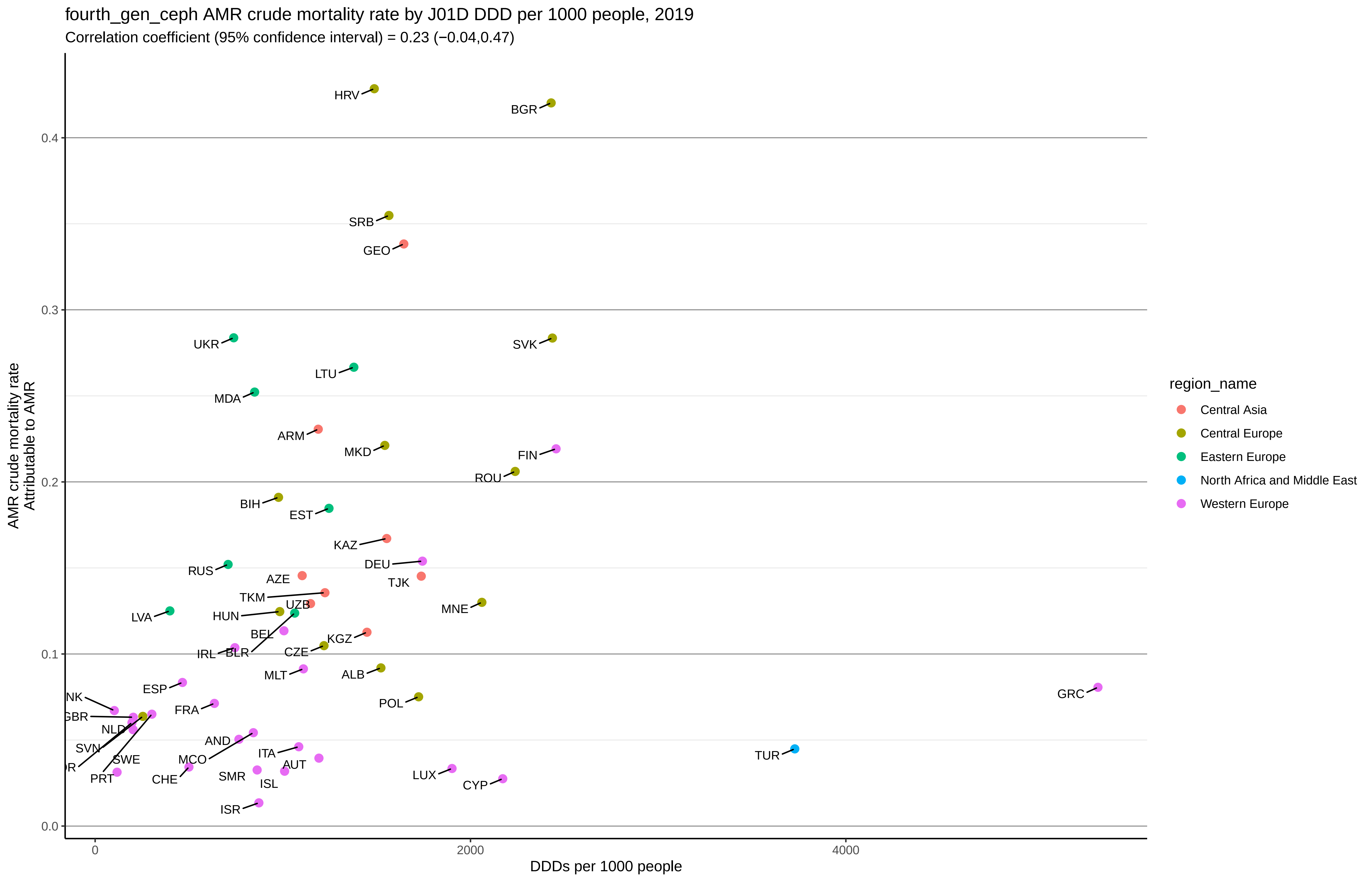
fourth_gen_ceph AMR crude mortality rate by J01D DDD per 1000 people, 2019
Correlation coefficient (95% confidence interval) = 0.23 (−0.04,0.47)
AMR crude mortality rate
Attributable to AMR
DDDs per 1000 people
0.0
0.1
0.2
0.3
0.4
0
2000
4000
region_name
Central Asia
Central Europe
Eastern Europe
North Africa and Middle East
Western Europe
HRV
BGR
SRB
GEO
UKR
SVK
LTU
MDA
ARM
MKD
FIN
ROU
BIH
EST
KAZ
DEU
RUS
AZE
TJK
TKM
UZB
MNE
LVA
HUN
BEL
KGZ
IRL
BLR
CZE
MLT
ALB
ESP
GRC
FRA
POL
GBR
NLD
SVN
AND
ITA
TUR
SWE
MCO
AUT
PRT
CHE
SMR
ISL
LUX
CYP
ISR

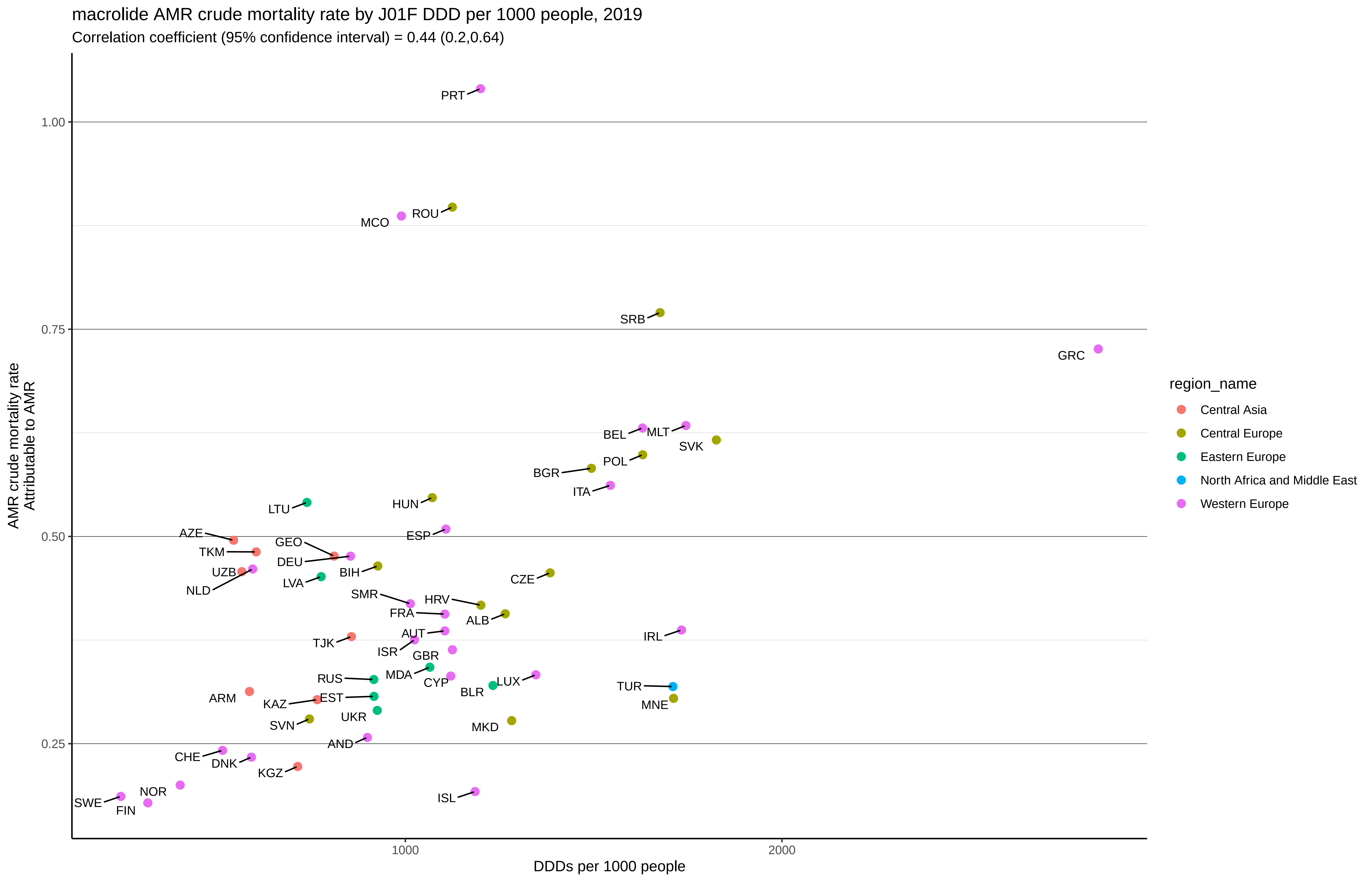

macrolide AMR crude mortality rate by J01F DDD per 1000 people, 2019
Correlation coefficient (95% confidence interval) = 0.44 (0.2,0.64)
AMR crude mortality rate
Attributable to AMR
DDDs per 1000 people
1.00
0.75
0.50
0.25
1000
2000
region_name
Central Asia
Central Europe
Eastern Europe
North Africa and Middle East
Western Europe
PRT
MCO
ROU
SRB
GRC
BEL
MLT
SVK
POL
BGR
ITA
LTU
HUN
ESP
AZE
GEO
TKM
DEU
BIH
UZB
LVA
NLD
CZE
SMR
HRV
FRA
ALB
AUT
TJK
ISR
GBR
IRL
RUS
MDA
CYP
LUX
BLR
TUR
ARM
KAZ
EST
MNE
UKR
SVN
MKD
AND
CHE
DNK
KGZ
NOR
SWE
FIN
ISL

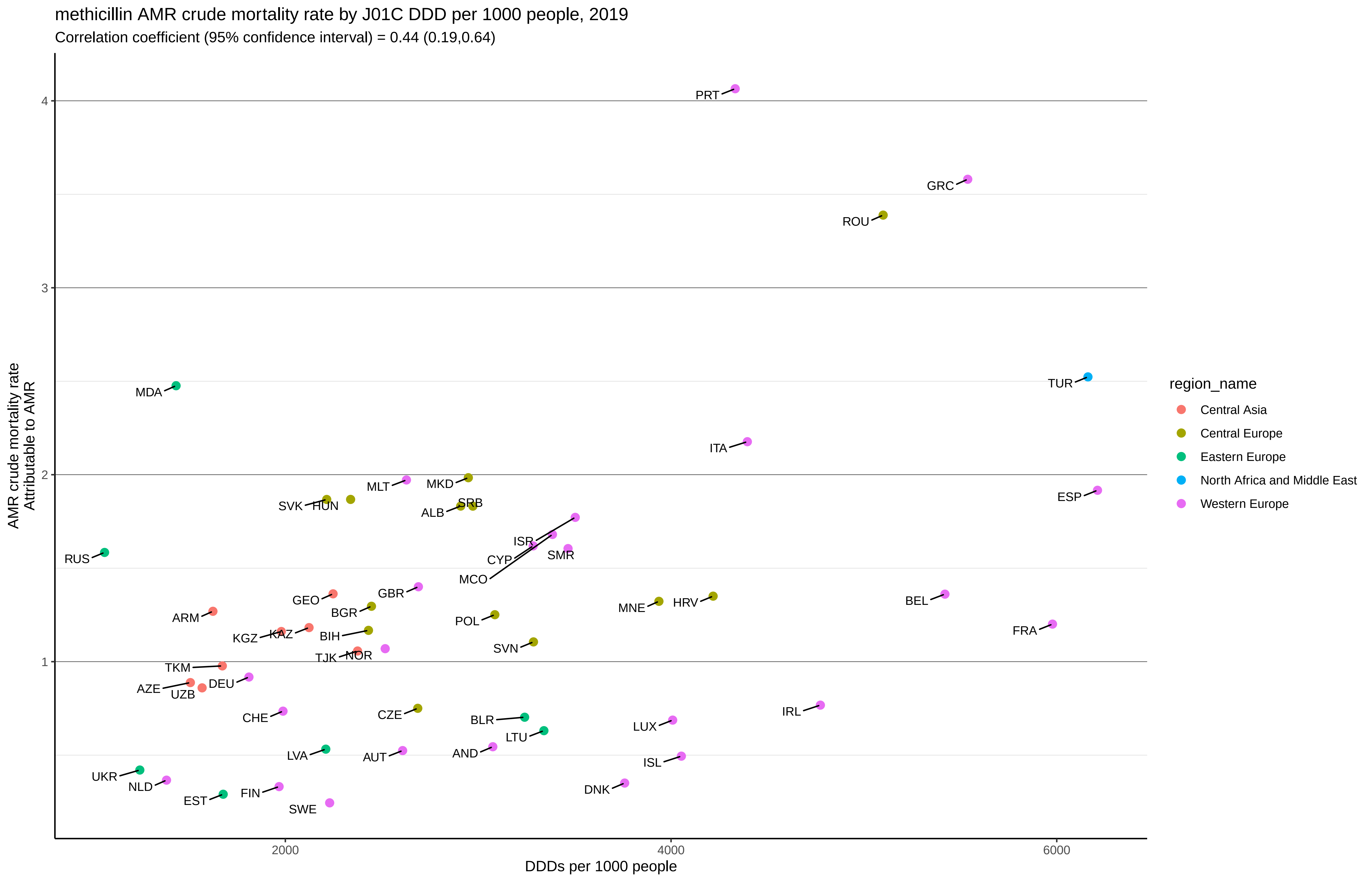
methicillin AMR crude mortality rate by J01C DDD per 1000 people, 2019
Correlation coefficient (95% confidence interval) = 0.44 (0.19,0.64)
AMR crude mortality rate
Attributable to AMR
DDDs per 1000 people
4
3
2
1
2000
4000
6000
region_name
Central Asia
Central Europe
Eastern Europe
North Africa and Middle East
Western Europe
PRT
GRC
ROU
TUR
MDA
ITA
ESP
MLT
MKD
SVK
HUN
SRB
ALB
ISR
CYP
SMR
MCO
RUS
GEO
GBR
BGR
ARM
KGZ
KAZ
BIH
POL
MNE
HRV
BEL
FRA
SVN
TJK
NOR
TKM
DEU
AZE
UZB
CHE
CZE
BLR
LTU
LUX
IRL
LVA
AUT
AND
ISL
UKR
NLD
EST
FIN
SWE
DNK

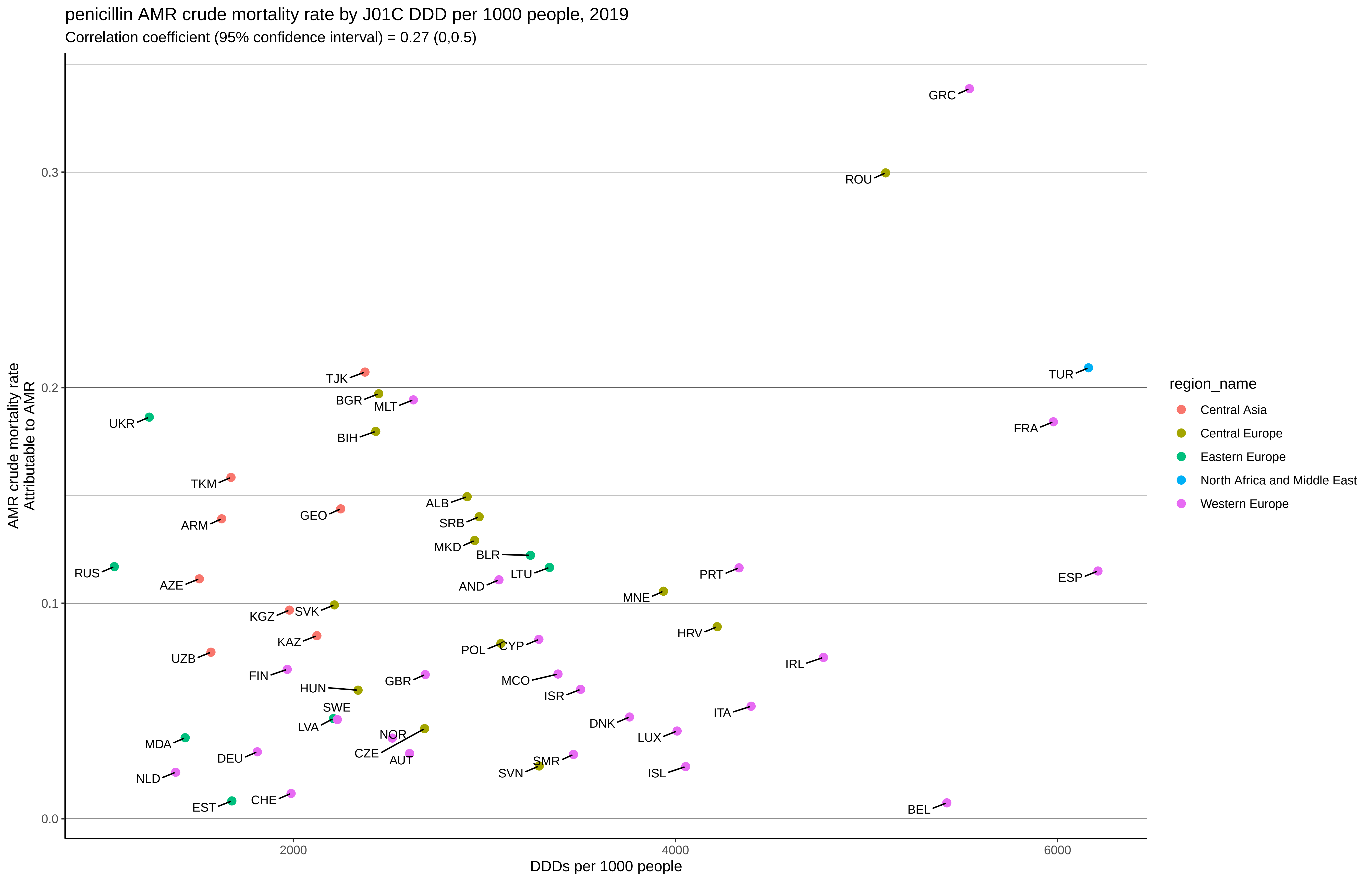

penicillin AMR crude mortality rate by J01C DDD per 1000 people, 2019
Correlation coefficient (95% confidence interval) = 0.27 (0,0.5)
AMR crude mortality rate
Attributable to AMR
DDDs per 1000 people
0.0
0.1
0.2
0.3
2000
4000
6000
region_name
Central Asia
Central Europe
Eastern Europe
North Africa and Middle East
Western Europe
GRC
ROU
TUR
TJK
BGR
MLT
UKR
BIH
FRA
TKM
ALB
GEO
SRB
ARM
MKD
BLR
RUS
LTU
PRT
ESP
AZE
AND
MNE
KGZ
SVK
HRV
KAZ
POL
CYP
UZB
IRL
FIN
MCO
GBR
HUN
ISR
SWE
ITA
DNK
LVA
NOR
LUX
MDA
CZE
DEU
AUT
SMR
SVN
ISL
NLD
CHE
EST
BEL

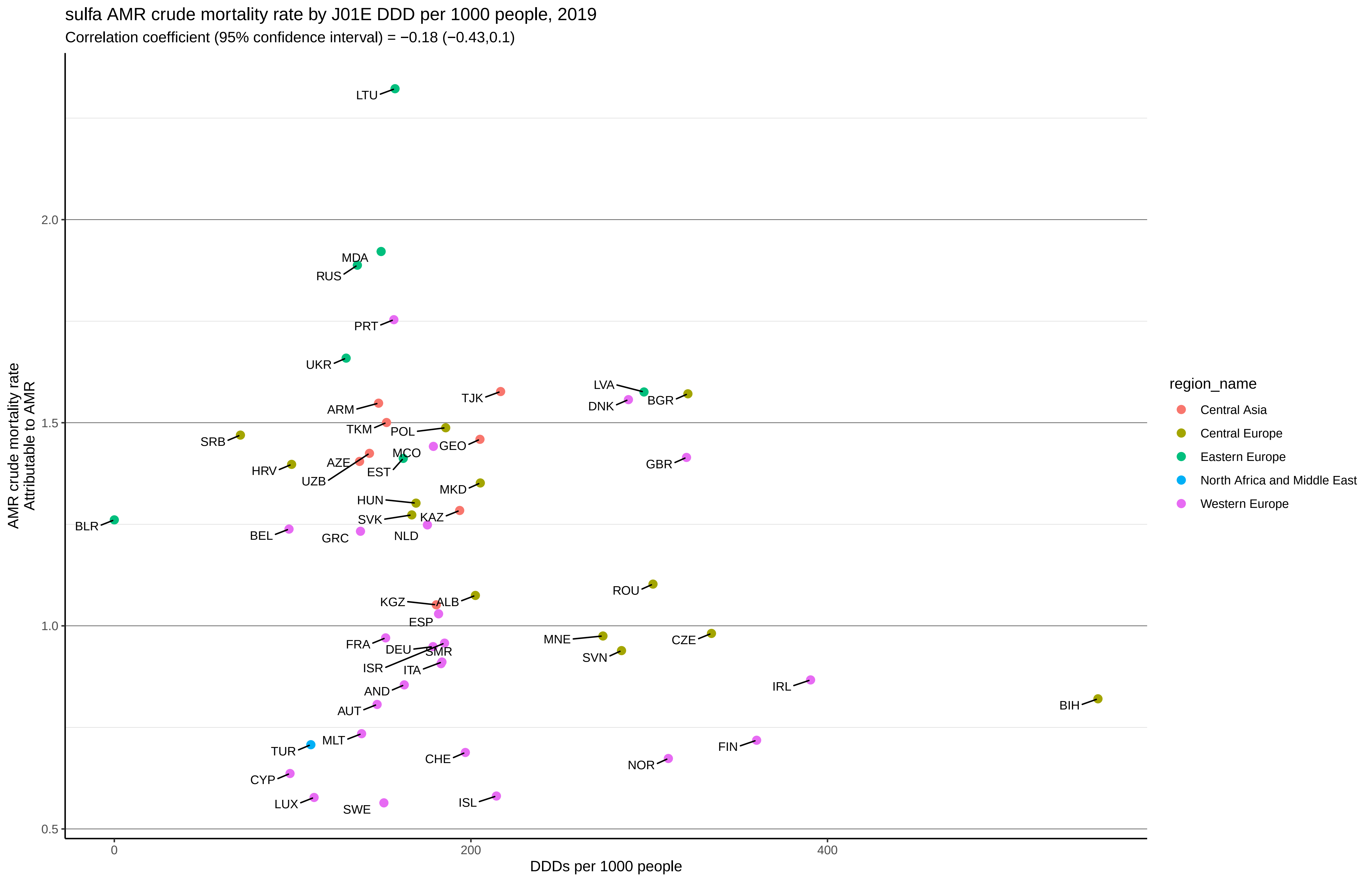

sulfa AMR crude mortality rate by J01E DDD per 1000 people, 2019
Correlation coefficient (95% confidence interval) = −0.18 (−0.43,0.1)
AMR crude mortality rate
Attributable to AMR
DDDs per 1000 people
0
200
400
0.5
1.0
1.5
2.0
region_name
Central Asia
Central Europe
Eastern Europe
North Africa and Middle East
Western Europe
LTU
MDA
RUS
PRT
UKR
LVA
TJK
DNK
BGR
ARM
TKM
POL
SRB
GEO
AZE
MCO
HRV
EST
GBR
UZB
MKD
HUN
SVK
KAZ
BLR
BEL
GRC
NLD
ROU
KGZ
ALB
ESP
MNE
CZE
FRA
DEU
SMR
SVN
ISR
ITA
IRL
AND
BIH
AUT
MLT
FIN
TUR
CHE
NOR
CYP
LUX
SWE
ISL

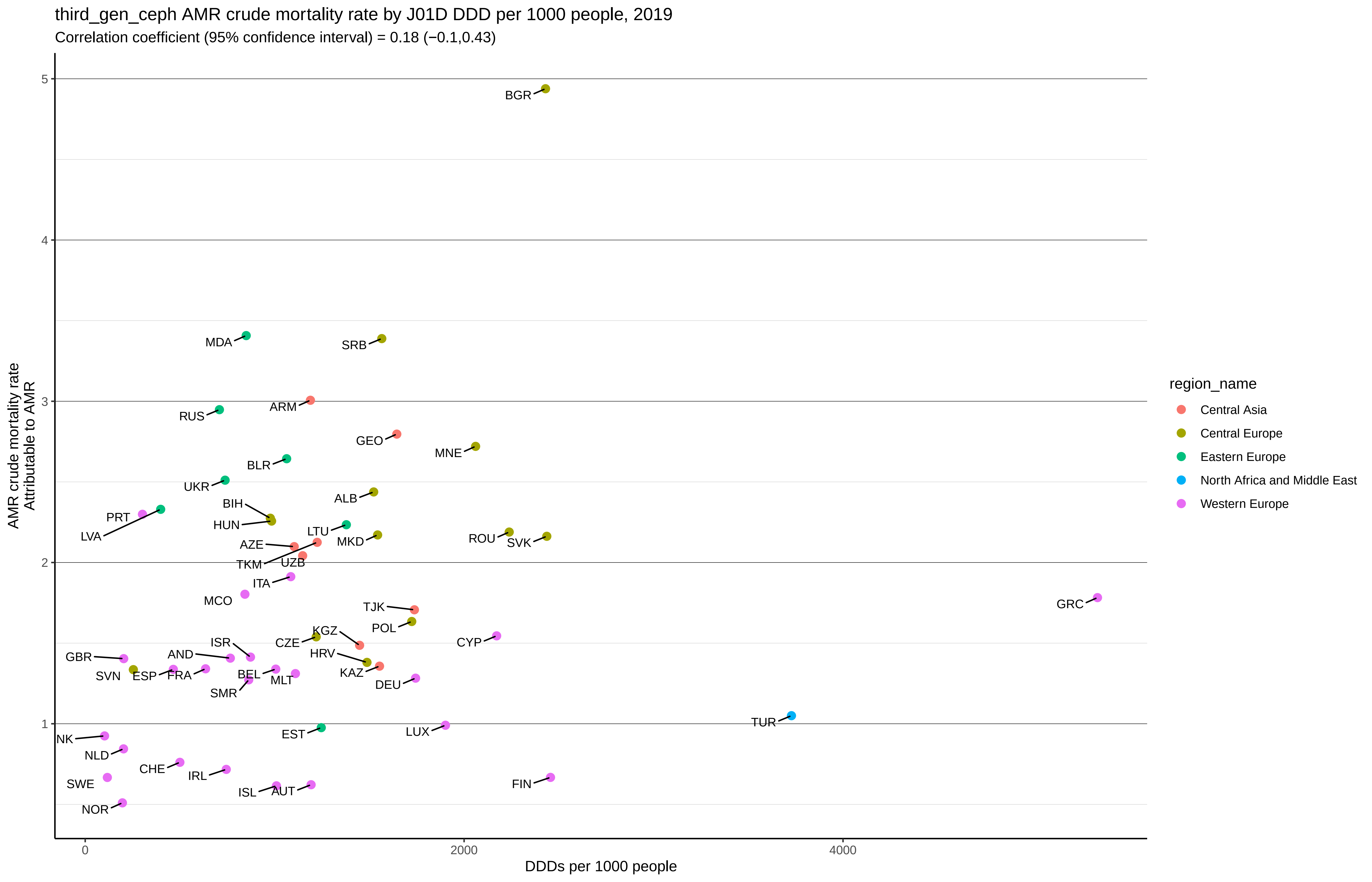

third_gen_ceph AMR crude mortality rate by J01D DDD per 1000 people, 2019
Correlation coefficient (95% confidence interval) = 0.18 (−0.1,0.43)
AMR crude mortality rate Attributable to AMR
DDDs per 1000 people
0
2000
4000
1
2
3
4
5
region_name
Central Asia
Central Europe
Eastern Europe
North Africa and Middle East
Western Europe
BGR
MDA
SRB
ARM
RUS
GEO
MNE
BLR
UKR
ALB
BIH
PRT
HUN
LVA
LTU
AZE
MKD
ROU
SVK
TKM
UZB
ITA
MCO
TJK
GRC
POL
KGZ
CYP
ISR
CZE
AND
HRV
GBR
KAZ
SVN
ESP
FRA
BEL
MLT
DEU
SMR
TUR
LUX
EST
NK
NLD
CHE
IRL
SWE
ISL
AUT
FIN
NOR

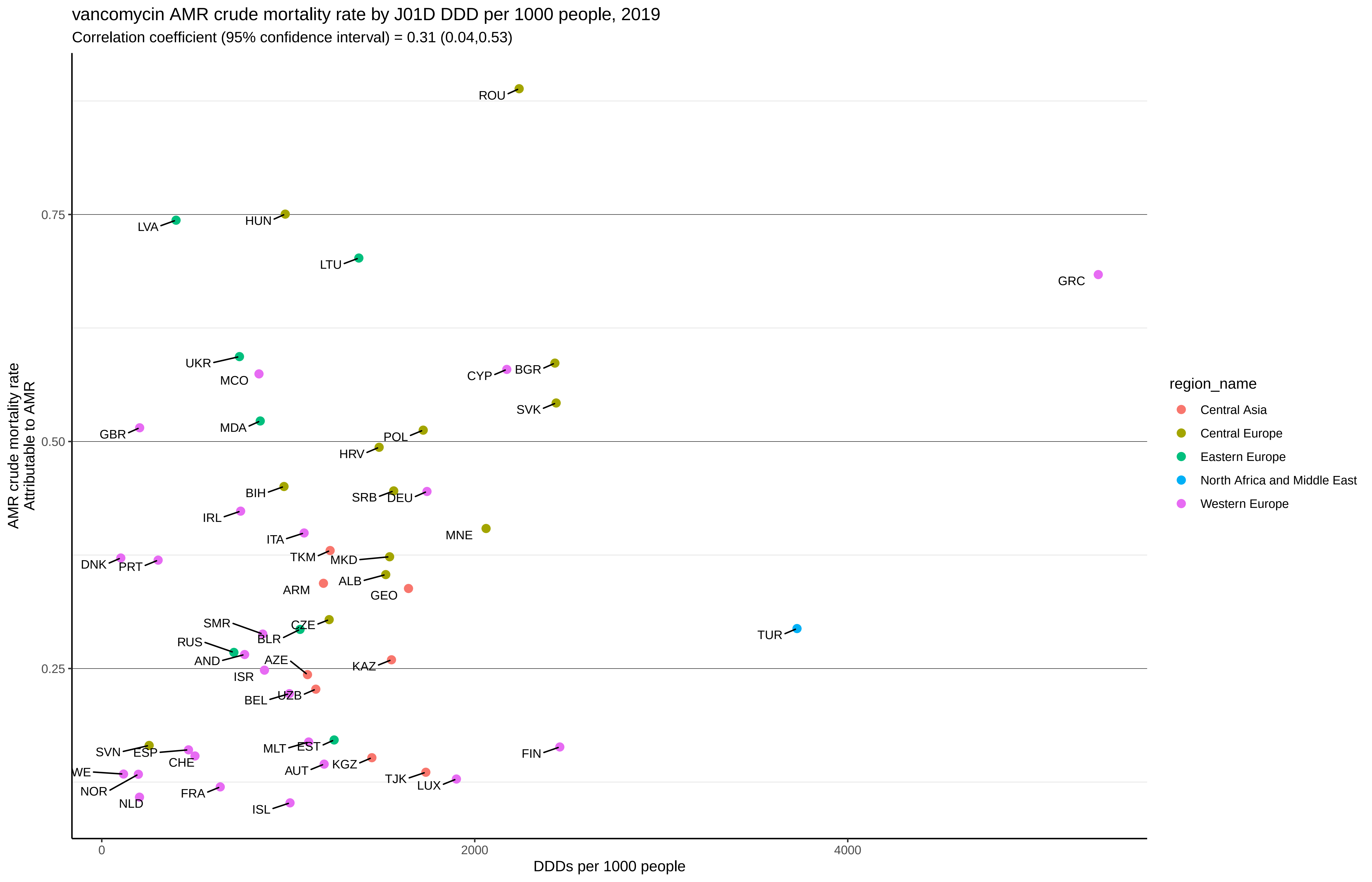

vancomycin AMR crude mortality rate by J01D DDD per 1000 people, 2019
Correlation coefficient (95% confidence interval) = 0.31 (0.04,0.53)
AMR crude mortality rate Attributable to AMR
DDDs per 1000 people
0.25
0.50
0.75
0
2000
4000
region_name
Central Asia
Central Europe
Eastern Europe
North Africa and Middle East
Western Europe
ROU
LVA
HUN
LTU
GRC
UKR
MCO
CYP
BGR
SVK
GBR
MDA
POL
HRV
BIH
SRB
DEU
IRL
MNE
ITA
TKM
MKD
DNK
PRT
ALB
ARM
GEO
SMR
CZE
BLR
TUR
RUS
AND
AZE
KAZ
ISR
BEL
UZB
SVN
ESP
CHE
MLT
EST
FIN
AUT
KGZ
WE
NOR
TJK
LUX
FRA
NLD
ISL

Albania Deaths (count) attributable to bacterial antimicrobial resistance by pathogen−drug combinations, 2019

	Resistance to 1+	Anti−pseudomonal	BL−BLI	CP	FQ	4GC	3GC	AG	Vanco	Aminopenicillin	TMP−SMX	PCN	Macrolide	Methicillin	Mono RIF	MDR excluding XDR in TB	XDR in TB	MDR in S. Typhi and Paratyphi	Mono INH
Acinetobacter baumannii	18.2	1.58	0.00691	7.74	6.32	0.00812	0.608	1.94											
Citrobacter spp.	1.32	0.417		0.171	0.227	0.0303	0.456	0.021											
Enterobacter spp.	6.06	1.8		1.41	0.91	0.807		0.244			0.857								
Enterococcus faecalis	7.06				6.29				0.769										
Enterococcus faecium	22.2				15.2				7.06										
Other enterococci	4.37				3.46				0.91										
Escherichia coli	104		9.31	11.4	25.2		34	5.02		4.61	15								
Group A Streptococcus	0.737												0.709						
Group B Streptococcus	4.03				2.49							0.123	1.46						
Haemophilus influenzae	0.494						0.138			0.357									
Klebsiella pneumoniae	51.8		2.06	5.08	8.23		23.9	5.47			7.06								
Morganella spp.	0.123				0.0502	0.0213	0.0513												
Mycobacterium tuberculosis	0.201														0.00745	0.0982	0.046		0.0485
Proteus spp.	4.46				0.74		2.66	0.0824		0.285	0.678								
Pseudomonas aeruginosa	27.1	4.21		9.62	6.82	1.27	4.23	0.882											
Salmonella Paratyphi	0.000496				0.000494													2.11e−06	
Salmonella Typhi	0.425				0.392													0.034	
Non−typhoidal Salmonella	0.00695				0.00695														
Serratia spp.	1.16	0.199		0.228	0.0994	0.361	0.182	0.0876											
Shigella spp.	0.00221				0.00221														
Staphylococcus aureus	62.2				4.52				0.876		1.1		5.82	49.8					
Streptococcus pneumoniae	22.3		0.068	10.2	0.395		0.154				4.46	3.94	3.17						
All pathogens	339	8.22	11.4	45.8	81.3	2.5	66.3	13.7	9.62	5.26	29.2	4.07	11.1	49.8	0.00745	0.0982	0.046	0.0307	0.0504

Counts
< 5k
NA

Andorra Deaths (count) attributable to bacterial antimicrobial resistance by pathogen−drug combinations, 2019

	Resistance to 1+	Anti−pseudomonal	BL−BLI	CP	FQ	4GC	3GC	AG	Vanco	Aminopenicillin	TMP−SMX	PCN	Macrolide	Methicillin	Mono RIF	MDR excluding XDR in TB	XDR in TB	MDR in S. Typhi and Paratyphi	Mono INH
Acinetobacter baumannii	0.407	0.148	0.0178	0.0577	0.0431	0.00921	0.122	0.00976											
Citrobacter spp.	0.0351	0.0136		0.00274	0.00499	0.000835	0.0123	0.000559											
Enterobacter spp.	0.15	0.0703		0.0455	0.00779	0.00856		0.00382			0.0142								
Enterococcus faecalis	0.154				0.133				0.0216										
Enterococcus faecium	0.762				0.614				0.149										
Other enterococci	0.0383				0.0283				0.00993										
Escherichia coli	2.89		0.498	0.0523	0.859		0.801	0.0745		0.188	0.423								
Group A Streptococcus	0.0107												0.0102						
Group B Streptococcus	0.112				0.0548							0.005	0.0516						
Haemophilus influenzae	0.024						0.00361			0.0204									
Klebsiella pneumoniae	0.659		0.198	0.05	0.0728		0.149	0.0649			0.124								
Morganella spp.	0.00401				0.00161	0.000455	0.00194												
Mycobacterium tuberculosis	0.0036														3.19e−05	0.000824	0.00022		0.00278
Proteus spp.	0.108				0.0137		0.0441	0.00349		0.0231	0.0231								
Pseudomonas aeruginosa	0.383	0.0765		0.159	0.102	0.018	0.0203	0.00754											
Salmonella Paratyphi	7.94e−06				7.91e−06													2.45e−08	
Salmonella Typhi	0.00367				0.00347													0.000207	
Non−typhoidal Salmonella	0.000968				0.000968														
Serratia spp.	0.0211	0.00509		0.00406	0.00159	0.0048	0.00509	0.000498											
Shigella spp.	0.000546				0.000546														
Staphylococcus aureus	0.823				0.164				0.0404		0.0305		0.135	0.453					
Streptococcus pneumoniae	0.376		0.00553	0.125	0.0392		0.00883				0.0953	0.0871	0.0157						
All pathogens	6.97	0.313	0.719	0.496	2.14	0.0419	1.17	0.165	0.22	0.231	0.71	0.0921	0.214	0.453	3.19e−05	0.000824	0.00022	0.000194	0.00273

Counts
< 5k
NA

Armenia Deaths (count) attributable to bacterial antimicrobial resistance by pathogen−drug combinations, 2019

	Resistance to 1+	Anti−pseudomonal	BL−BLI	CP	FQ	4GC	3GC	AG	Vanco	Aminopenicillin	TMP−SMX	PCN	Macrolide	Methicillin	Mono RIF	MDR excluding XDR in TB	XDR in TB	MDR in S. Typhi and Paratyphi	Mono INH
Acinetobacter baumannii	42.6	5.09	0.0104	17.3	14.6	0.132	0.629	4.78											
Citrobacter spp.	3.11	0.446		0.687	0.881	0.065	0.996	0.0388											
Enterobacter spp.	15.6	6.53		2.28	2	2.4		0.676			1.7								
Enterococcus faecalis	15				13.6				1.33										
Enterococcus faecium	34				27.8				6.22										
Other enterococci	13				11.8				1.21										
Escherichia coli	126		13	6.93	34.2		41.1	6.34		5.29	19								
Group A Streptococcus	0.592												0.559						
Group B Streptococcus	5.63				2.02							0.445	3.1						
Haemophilus influenzae	0.527						0.171			0.356									
Klebsiella pneumoniae	87.1		4.23	7.96	13.6		38.2	10			13.1								
Morganella spp.	0.583				0.236	0.0685	0.278												
Mycobacterium tuberculosis	15.9														0.787	9.23	4.48		1.35
Proteus spp.	7.87				1.09		3.73	0.784		1.07	1.12								
Pseudomonas aeruginosa	48.8	9.01		20.4	10.8	2.45	4.48	1.75											
Salmonella Paratyphi	0.086				0.0857													0.000266	
Salmonella Typhi	0.573				0.538													0.0333	
Non−typhoidal Salmonella	0.012				0.012														
Serratia spp.	5.57	1.45		0.932	0.356	1.85	0.604	0.381											
Shigella spp.	0.0441				0.0441														
Staphylococcus aureus	52.9				6.64				1.62		1.35		4.93	38.3					
Streptococcus pneumoniae	22.8		0.185	6.41	0.654		0.614				10.5	3.76	0.756						
All pathogens	498	22.5	17.4	62.9	141	6.96	90.8	24.7	10.4	6.76	46.7	4.2	9.45	38.3	0.787	9.23	4.48	0.035	1.34

Counts
< 5k
NA

Austria Deaths (count) attributable to bacterial antimicrobial resistance by pathogen−drug combinations, 2019

	Resistance to 1+	Anti−pseudomonal	BL−BLI	CP	FQ	4GC	3GC	AG	Vanco	Aminopenicillin	TMP−SMX	PCN	Macrolide	Methicillin	Mono RIF	MDR excluding XDR in TB	XDR in TB	MDR in S. Typhi and Paratyphi	Mono INH
Acinetobacter baumannii	65.1	53.6	0.00334	4.74	3.92	0.62	0.915	1.32											
Citrobacter spp.	3.81	1.09		0.839	0.415	0.117	1.28	0.0693											
Enterobacter spp.	23.9	15.3		3.81	0.936	1.98		0.433			1.43								
Enterococcus faecalis	9.68				8.07				1.6										
Enterococcus faecium	75.4				68.9				6.42										
Other enterococci	3.36				2.49				0.872										
Escherichia coli	230		46.7	2.11	58.2		34	8.57		33.2	47.3								
Group A Streptococcus	0.764												0.762						
Group B Streptococcus	8.63				1.34							0.374	6.79						
Haemophilus influenzae	1.77						0.37			1.4									
Klebsiella pneumoniae	50.8		10.5	4.47	9.92		10.7	3.4			11.8								
Morganella spp.	0.315				0.106	0.0314	0.177												
Mycobacterium tuberculosis	2.83														0.00816	1.87	0.507		0.462
Proteus spp.	10.6				1.6		4	0.208		3.27	1.59								
Pseudomonas aeruginosa	44.2	10.6		19.9	9.58	0.7	2.74	0.791											
Salmonella Paratyphi	0.0243				0.0242													7.53e−05	
Salmonella Typhi	0.357				0.304													0.0587	
Non−typhoidal Salmonella	0.925				0.925														
Serratia spp.	0.871	0.196		0.317	0.121	0.0754	0.104	0.0585											
Shigella spp.	0.16				0.16														
Staphylococcus aureus	86				7.47				4.01		3.52		24.2	46.8					
Streptococcus pneumoniae	20.8		0.463	4.93	3.1		1.23				6.29	2.32	2.5						
All pathogens	640	80.8	57.7	41.1	178	3.52	55.5	14.9	12.9	37.7	71.9	2.7	34.4	46.8	0.00816	1.87	0.507	0.0515	0.456

Counts: **< 5k** | **NA**

Azerbaijan Deaths (count) attributable to bacterial antimicrobial resistance by pathogen−drug combinations, 2019

	Resistance to 1+	Anti−pseudomonal	BL−BLI	CP	FQ	4GC	3GC	AG	Vanco	Aminopenicillin	TMP−SMX	PCN	Macrolide	Methicillin	Mono RIF	MDR excluding XDR in TB	XDR in TB	MDR in S. Typhi and Paratyphi	Mono INH
Acinetobacter baumannii	117	12.9	0.0449	49.6	40.6	0.158	1.2	12.1											
Citrobacter spp.	8.52	1.17		2.1	2.44	0.165	2.34	0.297											
Enterobacter spp.	37.8	13.5		7.3	3.4	5.53		2.18			5.91								
Enterococcus faecalis	24.2				20.3				3.92										
Enterococcus faecium	74.6				57.6				16.9										
Other enterococci	22.6				21.4				1.19										
Escherichia coli	251		28.9	15.1	62		78.8	16.7		10.6	38.5								
Group A Streptococcus	1.73												1.67						
Group B Streptococcus	24.4				13							2.19	9.19						
Haemophilus influenzae	5.49						3.18			2.31									
Klebsiella pneumoniae	224		11.9	18.2	30.5		100	25.1			37.7								
Morganella spp.	0.835				0.477	0.13	0.227												
Mycobacterium tuberculosis	153														9.96	90.9	44.4		7.75
Proteus spp.	17				2.55		9.4	0.969		1.61	2.47								
Pseudomonas aeruginosa	117	19.2		40.5	29.8	6.96	17.2	3.49											
Salmonella Paratyphi	0.00592				0.0059													2.01e−05	
Salmonella Typhi	2.82				2.4													0.403	
Non−typhoidal Salmonella	0.058				0.058														
Serratia spp.	12.8	3.62		2.58	1.23	2.02	2.32	1.05											
Shigella spp.	0.156				0.156														
Staphylococcus aureus	151				16.1				2.96		11.1		29.8	91.3					
Streptococcus pneumoniae	123		2.27	49.6	2.66		0.623				48.7	9.26	10.5						
All pathogens	1,370	50.5	43.1	185	307	15	216	62.1	25	14.4	144	11.4	50.9	91.3	9.96	90.9	44.4	0.422	7.61

Counts
< 5k
NA

Belarus Deaths (count) attributable to bacterial antimicrobial resistance by pathogen−drug combinations, 2019

	Resistance to 1+	Anti−pseudomonal	BL−BLI	CP	FQ	4GC	3GC	AG	Vanco	Aminopenicillin	TMP−SMX	PCN	Macrolide	Methicillin	Mono RIF	MDR excluding XDR in TB	XDR in TB	MDR in S. Typhi and Paratyphi	Mono INH
Acinetobacter baumannii	133	17.9	0.0121	43.9	52.9	3.34	0.172	15.1											
Citrobacter spp.	10.7	4.25		2.21	0.958	0.101	3.02	0.178											
Enterobacter spp.	45.3	22.5		7.4	3.64	4.34		1.39			5.97								
Enterococcus faecalis	65				60.8				4.17										
Enterococcus faecium	94.7				75.2				19.5										
Other enterococci	22.9				21.7				1.18										
Escherichia coli	350		37.2	12	61.6		158	34.1		1.17	45.4								
Group A Streptococcus	1.42												1.33						
Group B Streptococcus	15.2				1.33							0.333	13.8						
Haemophilus influenzae	0.76						0.444			0.316									
Klebsiella pneumoniae	222		5.66	35.4	31		76.4	40.5			33.3								
Morganella spp.	0.638				0.126	0.0463	0.465												
Mycobacterium tuberculosis	110														0.418	72.4	36		1.56
Proteus spp.	13.9				3.3		4.43	0.523		3.43	2.28								
Pseudomonas aeruginosa	121	10.9		60.2	38.3	3.07	3.83	4.04											
Salmonella Paratyphi	0.00261				0.00261													8.58e−06	
Salmonella Typhi	1.11				0.745													0.363	
Non−typhoidal Salmonella	0.766				0.766														
Serratia spp.	6.5	1.14		1.49	0.283	0.858	2.23	0.486											
Shigella spp.	0.0539				0.0539														
Staphylococcus aureus	88.1				4.31				2.97		2.91		11.1	66.8					
Streptococcus pneumoniae	61.3		0.859	9.51	3.14		2.06				30	11.3	4.36						
All pathogens	1,360	56.6	43.7	172	360	11.8	251	96.4	27.8	5.25	120	11.6	30.4	66.8	0.418	72.4	36	0.356	1.7

Counts
- **< 5k**
- **NA**

Belgium Deaths (count) attributable to bacterial antimicrobial resistance by pathogen−drug combinations, 2019

	Resistance to 1+	Anti−pseudomonal	BL−BLI	CP	FQ	4GC	3GC	AG	Vanco	Aminopenicillin	TMP−SMX	PCN	Macrolide	Methicillin	Mono RIF	MDR excluding XDR in TB	XDR in TB	MDR in S. Typhi and Paratyphi	Mono INH
Acinetobacter baumannii	72	14.6	12.7	7.36	7.96	7.09	20.7	1.67											
Citrobacter spp.	8.77	4.5		1.09	1.23	0.049	1.8	0.108											
Enterobacter spp.	38.4	19.2		6.6	9.89	0.79		0.828			1.01								
Enterococcus faecalis	17.6				12.4				5.25										
Enterococcus faecium	126				118				8.71										
Other enterococci	10.9				8.14				2.8										
Escherichia coli	470		113	14	110		61.1	16.4		66.3	89.1								
Group A Streptococcus	1.22												1.22						
Group B Streptococcus	15.2				3.72							0.568	11.1						
Haemophilus influenzae	9.82						1.3			8.51									
Klebsiella pneumoniae	154		24.8	14.1	31		55.5	12.7			16.2								
Morganella spp.	1.3				0.362	0.0884	0.853												
Mycobacterium tuberculosis	2.89														0.0308	1.77	0.479		0.592
Proteus spp.	17.7				2.94		4.19	0.429		6.35	3.56								
Pseudomonas aeruginosa	111	21.8		54.2	22.4	4.66	4.65	2.92											
Salmonella Paratyphi	0.00712				0.0071													2.44e−05	
Salmonella Typhi	0.511				0.443													0.0679	
Non−typhoidal Salmonella	3.23				3.23														
Serratia spp.	4.16	0.913		0.588	0.36	0.291	1.92	0.0829											
Shigella spp.	0.916				0.916														
Staphylococcus aureus	256				35.4				8.63		8.04		48.7	155					
Streptococcus pneumoniae	87.2		0.496	48.9	1.94		0.954				23.5	0.275	11						
All pathogens	1,410	61	151	147	370	13	153	35.2	25.4	81.4	141	0.843	72	155	0.0308	1.77	0.479	0.0722	0.628

Counts
< 5k
NA

Bosnia and Herzegovina Deaths (count) attributable to bacterial antimicrobial resistance by pathogen−drug combinations, 2019

	Resistance to 1+	Anti−pseudomonal	BL−BLI	CP	FQ	4GC	3GC	AG	Vanco	Aminopenicillin	TMP−SMX	PCN	Macrolide	Methicillin	Mono RIF	MDR excluding XDR in TB	XDR in TB	MDR in S. Typhi and Paratyphi	Mono INH
Acinetobacter baumannii	67.3	12.2	0.0337	21.3	26.3	0.152	1.18	6.26											
Citrobacter spp.	8.37	1.91		1.21	3.95	0.225	0.979	0.107											
Enterobacter spp.	21.7	7.3		2.5	4.64	3.5		1.06			2.67								
Enterococcus faecalis	20.1				18.9				1.18										
Enterococcus faecium	36.8				26				10.7										
Other enterococci	11				8.99				1.97										
Escherichia coli	106		16.2	16.8	19.3		27.2	8.08		4.71	14.1								
Group A Streptococcus	0.914												0.933						
Group B Streptococcus	4.86				0.97							0.2	3.85						
Haemophilus influenzae	0.599						0.166			0.433									
Klebsiella pneumoniae	83.5		4.16	19.6	12.2		29.8	12.7			5.02								
Morganella spp.	0.327				0.124	0.0522	0.151												
Mycobacterium tuberculosis	1.17														0.00538	0.658	0.311		0.198
Proteus spp.	7.46				1.84		3.39	0.472		0.901	0.833								
Pseudomonas aeruginosa	44.9	7.19		19.9	11.3	1.42	3.22	1.85											
Salmonella Paratyphi	0.000389				0.000387													1.36e−06	
Salmonella Typhi	0.445				0.416													0.0299	
Non−typhoidal Salmonella	0.0347				0.0347														
Serratia spp.	4.02	0.922		0.767	0.269	0.955	0.726	0.376											
Shigella spp.	0.00277				0.00277														
Staphylococcus aureus	52.7				4.59				0.986		1.04		7.55	38.5					
Streptococcus pneumoniae	32.9		0.0336	11.4	1.01		8.17				3.34	5.73	3.17						
All pathogens	506	29.5	20.5	93.5	141	6.3	75.1	30.8	14.9	6.05	27.1	5.93	15.3	38.5	0.00538	0.658	0.311	0.0273	0.194

Counts
< 5k
NA

Bulgaria Deaths (count) attributable to bacterial antimicrobial resistance by pathogen−drug combinations, 2019

	Resistance to 1+	Anti−pseudomonal	BL−BLI	CP	FQ	4GC	3GC	AG	Vanco	Aminopenicillin	TMP−SMX	PCN	Macrolide	Methicillin	Mono RIF	MDR excluding XDR in TB	XDR in TB	MDR in S. Typhi and Paratyphi	Mono INH
Acinetobacter baumannii	117	16.8	0.105	49	37.9	0.665	0.272	12.2											
Citrobacter spp.	10.8	2.79		2.18	2.17	0.191	3.32	0.123											
Enterobacter spp.	54.7	15.9		3.55	13.9	12.1		4.19			5.02								
Enterococcus faecalis	78				74.1				3.86										
Enterococcus faecium	109				82.6				26.5										
Other enterococci	26.6				21.4				5.24										
Escherichia coli	506		62.6	16.1	126		165	40.4		27.2	68.8								
Group A Streptococcus	3.16												3.34						
Group B Streptococcus	11.9				2.69							0.59	8.57						
Haemophilus influenzae	3.01						1.6			1.41									
Klebsiella pneumoniae	318		5.99	78.2	52.1		115	42.5			24.7								
Morganella spp.	0.995				0.449	0.148	0.398												
Mycobacterium tuberculosis	6														0.283	3.2	1.51		1.05
Proteus spp.	56.1				3.18		48.6	1.47		0.00392	2.84								
Pseudomonas aeruginosa	122	33.1		43.4	29	5.77	4.78	6.47											
Salmonella Paratyphi	0.00288				0.00287													1.04e−05	
Salmonella Typhi	2.22				1.92													0.293	
Non−typhoidal Salmonella	0.0501				0.0501														
Serratia spp.	18.6	3.15		1.61	0.447	10.2	1.87	1.27											
Shigella spp.	0.0519				0.0519														
Staphylococcus aureus	127				8.98				5.04		4.56		18.5	89.9					
Streptococcus pneumoniae	73.8		2.31	35.6	7.75		2.04				3.13	13.1	9.82						
All pathogens	1,650	71.7	71	230	465	29.1	342	108	40.7	28.6	109	13.7	40.4	89.9	0.283	3.2	1.51	0.309	0.945

Counts
< 5k
NA

Croatia Deaths (count) attributable to bacterial antimicrobial resistance by pathogen−drug combinations, 2019

	Resistance to 1+	Anti−pseudomonal	BL−BLI	CP	FQ	4GC	3GC	AG	Vanco	Aminopenicillin	TMP−SMX	PCN	Macrolide	Methicillin	Mono RIF	MDR excluding XDR in TB	XDR in TB	MDR in S. Typhi and Paratyphi	Mono INH
Acinetobacter baumannii	93.2	1.72	0.0133	45.5	34.5	0.45	0.541	10.6											
Citrobacter spp.	5.23	1.77		0.64	0.737	0.122	1.87	0.0907											
Enterobacter spp.	29.3	6.74		3.69	6.04	10.2		0.374			2.33								
Enterococcus faecalis	26.1				24.3				1.71										
Enterococcus faecium	51.6				34.9				16.7										
Other enterococci	8.27				7.54				0.734										
Escherichia coli	148		24.8	3.14	35.1		23.9	6		11.5	43.1								
Group A Streptococcus	0.726												0.794						
Group B Streptococcus	5.26				0.808							0.135	4.16						
Haemophilus influenzae	0.329						0.213			0.116									
Klebsiella pneumoniae	75.5		6.58	10.2	17.2		25.9	9.06			6.52								
Morganella spp.	0.605				0.0372	0.0459	0.522												
Mycobacterium tuberculosis	0.466														0.00163	0.237	0.11		0.116
Proteus spp.	7.78				1.74		3.29	0.244		1.44	1.06								
Pseudomonas aeruginosa	51	5.08		22.8	13.3	6.87	1.61	1.37											
Salmonella Paratyphi	0.00133				0.00132													4.88e−06	
Salmonella Typhi	0.18				0.16													0.0193	
Non−typhoidal Salmonella	0.0284				0.0284														
Serratia spp.	2.08	0.398		0.314	0.183	0.524	0.591	0.0714											
Shigella spp.	0.00526				0.00526														
Staphylococcus aureus	79.4				10.2				1.85		1.57		8.4	57.4					
Streptococcus pneumoniae	29.1		1.83	13.6	0.673		0.214				4.77	3.65	4.41						
All pathogens	614	15.7	33.3	99.9	188	18.2	58.6	27.7	21	13.1	59.4	3.79	17.7	57.4	0.00163	0.237	0.11	0.0183	0.12

Counts
< 5k
NA

Cyprus Deaths (count) attributable to bacterial antimicrobial resistance by pathogen−drug combinations, 2019

	Resistance to 1+	Anti−pseudomonal	BL−BLI	CP	FQ	4GC	3GC	AG	Vanco	Aminopenicillin	TMP−SMX	PCN	Macrolide	Methicillin	Mono RIF	MDR excluding XDR in TB	XDR in TB	MDR in S. Typhi and Paratyphi	Mono INH
Acinetobacter baumannii	6.89	0.00777	0.0675	3.17	2.5	0.0512	0.439	0.658											
Citrobacter spp.	0.684	0.173		0.0671	0.105	0.0456	0.285	0.00798											
Enterobacter spp.	2.42	0.89		0.764	0.52	0.0645		0.0474			0.136								
Enterococcus faecalis	4.44				4.06				0.38										
Enterococcus faecium	12.8				6.37				6.41										
Other enterococci	1.84				1.49				0.35										
Escherichia coli	46.7		4.14	1.24	13.4		14.1	2.5		6.01	5.23								
Group A Streptococcus	0.223												0.223						
Group B Streptococcus	1.14				0.152							0.0596	0.995						
Haemophilus influenzae	0.335						0.158			0.177									
Klebsiella pneumoniae	18.5		0.764	6	4.22		4.09	2.49			0.976								
Morganella spp.	0.0838				0.0502	0.00882	0.0248												
Mycobacterium tuberculosis	0.245														7.53e−05	0.109	0.0297		0.108
Proteus spp.	1.64				0.542		0.578	0.0498		0.247	0.211								
Pseudomonas aeruginosa	7.37	1.69		3.08	1.94	0.15	0.428	0.0762											
Salmonella Paratyphi	0.000128				0.000127													4.27e−07	
Salmonella Typhi	0.0493				0.044													0.00514	
Non−typhoidal Salmonella	8.22e−06				8.22e−06														
Serratia spp.	0.298	0.0472		0.081	0.019	0.0411	0.101	0.008											
Shigella spp.	0.0138				0.0138														
Staphylococcus aureus	28.5				3.05				0.469		0.8		2.88	21.3					
Streptococcus pneumoniae	6.38		0.0127	3.74	0.192		0.0721				1.01	1.03	0.31						
All pathogens	140	2.81	4.98	18.1	38.7	0.361	20.3	5.84	7.61	6.44	8.36	1.09	4.35	21.3	7.53e−05	0.109	0.0297	0.00542	0.109

Counts
< 5k
NA

Czechia Deaths (count) attributable to bacterial antimicrobial resistance by pathogen−drug combinations, 2019

	Resistance to 1+	Anti−pseudomonal	BL−BLI	CP	FQ	4GC	3GC	AG	Vanco	Aminopenicillin	TMP−SMX	PCN	Macrolide	Methicillin	Mono RIF	MDR excluding XDR in TB	XDR in TB	MDR in S. Typhi and Paratyphi	Mono INH
Acinetobacter baumannii	115	3.82	37.8	23.7	23.4	1.51	19.8	4.69											
Citrobacter spp.	12.8	3.62		2.66	1.06	0.187	5.09	0.173											
Enterobacter spp.	37.6	11.2		5.8	3.5	7.21		0.952			8.99								
Enterococcus faecalis	64.8				62.5				2.29										
Enterococcus faecium	108				84.8				23.6										
Other enterococci	13.9				11.6				2.22										
Escherichia coli	285		86.1	6.15	67.5		55.5	14.3		10.9	44.3								
Group A Streptococcus	6.49												6.35						
Group B Streptococcus	14.7				4.06							0.469	10.5						
Haemophilus influenzae	1.45						0.701			0.753									
Klebsiella pneumoniae	182		18.5	6.5	33		71.1	29			23.8								
Morganella spp.	0.217				0.0642	0.0547	0.0981												
Mycobacterium tuberculosis	2.09														0.0184	1.24	0.583		0.243
Proteus spp.	11.3				1.08		2.31	0.359		4.75	2.95								
Pseudomonas aeruginosa	103	15.8		47.4	29.7	1.68	6.14	2.75											
Salmonella Paratyphi	0.0506				0.0504													0.000211	
Salmonella Typhi	0.745				0.63													0.108	
Non−typhoidal Salmonella	0.338				0.338														
Serratia spp.	3.94	0.579		1.27	0.691	0.519	0.77	0.117											
Shigella spp.	0.0513				0.0513														
Staphylococcus aureus	127				11.3				4.19		3.96		28	79.9					
Streptococcus pneumoniae	48		1.6	9.65	6		2.27				20.5	3.98	4.01						
All pathogens	1,140	35	144	103	341	11.2	164	52.4	32.3	16.3	104	4.45	48.5	79.9	0.0184	1.24	0.583	0.109	0.249

Counts
< 5k
NA

Denmark Deaths (count) attributable to bacterial antimicrobial resistance by pathogen−drug combinations, 2019

	Resistance to 1+	Anti−pseudomonal	BL−BLI	CP	FQ	4GC	3GC	AG	Vanco	Aminopenicillin	TMP−SMX	PCN	Macrolide	Methicillin	Mono RIF	MDR excluding XDR in TB	XDR in TB	MDR in S. Typhi and Paratyphi	Mono INH
Acinetobacter baumannii	31.2	7.69	8.16	2.67	1.83	1.63	8.66	0.508											
Citrobacter spp.	2.83	0.948		0.264	0.482	0.203	0.887	0.0491											
Enterobacter spp.	15.7	6.9		2.04	0.916	0.826		0.52			4.48								
Enterococcus faecalis	22.7				21.8				0.898										
Enterococcus faecium	62.9				52.8				10.1										
Other enterococci	14				7.77				6.27										
Escherichia coli	180		32.7	5	27.5		22.3	7.79		38.1	46.9								
Group A Streptococcus	0.457												0.439						
Group B Streptococcus	7.15				1.23							0.183	5.98						
Haemophilus influenzae	2.85						0.571			2.27									
Klebsiella pneumoniae	55.5		8.9	1.68	5.55		12.2	3.36			23.7								
Morganella spp.	0.227				0.0844	0.0422	0.101												
Mycobacterium tuberculosis	0.85														0.000341	0.543	0.147		0.151
Proteus spp.	6.61				0.345		1.05	0.227		1.87	3.13								
Pseudomonas aeruginosa	18.9	2.65		9.12	3.89	1.14	1.77	0.316											
Salmonella Paratyphi	0.141				0.14													0.000464	
Salmonella Typhi	0.212				0.189													0.0239	
Non−typhoidal Salmonella	0.000643				0.000643														
Serratia spp.	1.33	0.242		0.237	0.102	0.0531	0.654	0.0387											
Shigella spp.	0.23				0.23														
Staphylococcus aureus	42.8				3.43				4.33		8.19		6.53	20.3					
Streptococcus pneumoniae	20.9		0.358	5.6	2.09		5.42				4	2.56	0.929						
All pathogens	488	18.4	50.1	26.6	130	3.89	53.7	12.8	21.6	42.3	90.3	2.74	13.6	20.3	0.000341	0.543	0.147	0.0224	0.153

Counts
< 5k
NA

Estonia Deaths (count) attributable to bacterial antimicrobial resistance by pathogen−drug combinations, 2019

	Resistance to 1+	Anti−pseudomonal	BL−BLI	CP	FQ	4GC	3GC	AG	Vanco	Aminopenicillin	TMP−SMX	PCN	Macrolide	Methicillin	Mono RIF	MDR excluding XDR in TB	XDR in TB	MDR in S. Typhi and Paratyphi	Mono INH
Acinetobacter baumannii	24.2	7.74	0.298	6.37	5.19	1.57	1.91	1.08											
Citrobacter spp.	1.85	0.51		0.4	0.0755	0.035	0.806	0.0254											
Enterobacter spp.	5.25	2.21		1.31	0.329	0.588		0.104			0.737								
Enterococcus faecalis	8.21				7.68				0.534										
Enterococcus faecium	12.3				11.3				1.01										
Other enterococci	1.94				1.77				0.179										
Escherichia coli	29		3.79	0.398	5.85		4.37	1.17		5.31	8.11								
Group A Streptococcus	0.267												0.254						
Group B Streptococcus	1.78				0.227							0.0632	1.49						
Haemophilus influenzae	0.119						0.0497			0.0696									
Klebsiella pneumoniae	16.3		1.31	0.722	2.62		4.58	1.55			5.48								
Morganella spp.	0.0751				0.00957	0.00537	0.0601												
Mycobacterium tuberculosis	5.74														0.0437	3.64	1.77		0.293
Proteus spp.	1.52				0.171		0.426	0.0689		0.504	0.325								
Pseudomonas aeruginosa	7	1.14		4.12	1.34	0.104	0.179	0.121											
Salmonella Paratyphi	0.00428				0.00426													1.92e−05	
Salmonella Typhi	0.0595				0.0525													0.00679	
Non−typhoidal Salmonella	6.32e−58				6.32e−58														
Serratia spp.	0.921	0.17		0.219	0.048	0.123	0.306	0.0542											
Shigella spp.	0.0135				0.0135														
Staphylococcus aureus	7.42				0.507				0.53		0.503		2.06	3.82					
Streptococcus pneumoniae	5.89		0.0458	1.77	0.234		0.119				3.4	0.0454	0.284						
All pathogens	130	11.8	5.44	15.3	37.4	2.42	12.8	4.2	2.25	5.91	18.5	0.109	4.03	3.82	0.0437	3.64	1.77	0.00705	0.298

Counts
- < 5k
- NA

Finland Deaths (count) attributable to bacterial antimicrobial resistance by pathogen−drug combinations, 2019

	Resistance to 1+	Anti−pseudomonal	BL−BLI	CP	FQ	4GC	3GC	AG	Vanco	Aminopenicillin	TMP−SMX	PCN	Macrolide	Methicillin	Mono RIF	MDR excluding XDR in TB	XDR in TB	MDR in S. Typhi and Paratyphi	Mono INH
Acinetobacter baumannii	31.2	10.1	1.53	2.61	1.24	8.42	6.54	0.758											
Citrobacter spp.	2.53	0.608		0.633	0.227	0.0811	0.942	0.0437											
Enterobacter spp.	12.3	5.5		3.86	0.736	0.705		0.263			1.3								
Enterococcus faecalis	13.9				11.3				2.59										
Enterococcus faecium	51.6				49.8				1.86										
Other enterococci	2.42				1.19				1.23										
Escherichia coli	114		20.7	2.78	21.8		18.6	7.84		20.7	21.1								
Group A Streptococcus	0.891												0.906						
Group B Streptococcus	5.73				1.02							0.386	4.28						
Haemophilus influenzae	1.28						0.491			0.789									
Klebsiella pneumoniae	33.3		8.79	2.88	4.71		5.48	2.32			9.17								
Morganella spp.	0.197				0.0752	0.0202	0.101												
Mycobacterium tuberculosis	3.04														0.00177	2.14	0.582		0.304
Proteus spp.	6.83				0.493		2.79	0.245		2.11	1.18								
Pseudomonas aeruginosa	21.9	3.21		9	6.5	2.33	0.62	0.223											
Salmonella Paratyphi	0.0134				0.0134													4.66e−05	
Salmonella Typhi	0.153				0.134													0.0183	
Non−typhoidal Salmonella	0.00342				0.00342														
Serratia spp.	2.1	0.353		0.557	0.127	0.579	0.437	0.044											
Shigella spp.	0.0356				0.0356														
Staphylococcus aureus	28.9				2.07				3.38		2.43		2.69	18.4					
Streptococcus pneumoniae	17.1		0.128	5.1	0.955		0.936				4.56	3.45	1.85						
All pathogens	349	19.8	31.2	27.4	102	12.1	36.9	11.7	9.05	23.6	39.8	3.84	9.89	18.4	0.00177	2.14	0.582	0.0191	0.314

Counts

< 5k

NA

France Deaths (count) attributable to bacterial antimicrobial resistance by pathogen−drug combinations, 2019

	Resistance to 1+	Anti−pseudomonal	BL−BLI	CP	FQ	4GC	3GC	AG	Vanco	Aminopenicillin	TMP−SMX	PCN	Macrolide	Methicillin	Mono RIF	MDR excluding XDR in TB	XDR in TB	MDR in S. Typhi and Paratyphi	Mono INH
Acinetobacter baumannii	377	59.1	85.1	38.3	36.8	8.63	139	10.1											
Citrobacter spp.	32.8	13.9		3.42	2.89	2.45	9.63	0.538											
Enterobacter spp.	189	82.7		32.4	23.5	20.9		2.72			26.6								
Enterococcus faecalis	116				96.5				19										
Enterococcus faecium	577				554				23										
Other enterococci	30				21.1				8.87										
Escherichia coli	2,200		583	83.6	355		323	84.8		342	432								
Group A Streptococcus	18.1												16.9						
Group B Streptococcus	40.4				10.9							2.64	27.5						
Haemophilus influenzae	36.1						4.24			31.8									
Klebsiella pneumoniae	900		100	45.8	145		338	162			109								
Morganella spp.	5.64				1.31	0.39	3.94												
Mycobacterium tuberculosis	38.1														0.36	24.1	6.56		7.01
Proteus spp.	66.8				7.44		25.9	4.12		20.3	9.22								
Pseudomonas aeruginosa	573	153		251	112	14	25.8	18.4											
Salmonella Paratyphi	0.0229				0.0228													8.73e−05	
Salmonella Typhi	2.68				2.25													0.436	
Non−typhoidal Salmonella	6.29				6.29														
Serratia spp.	21.8	6.16		3.02	1.55	0.913	9.65	0.484											
Shigella spp.	1.08				1.08														
Staphylococcus aureus	1,150				118				28.3		35		174	795					
Streptococcus pneumoniae	771		1.29	228	333		8.41				30.3	119	52.9						
All pathogens	7,160	314	769	686	1,830	47.2	888	283	79.2	394	642	122	269	795	0.36	24.1	6.56	0.414	6.67

Counts
- 5k − <10k
- < 5k
- NA

Georgia Deaths (count) attributable to bacterial antimicrobial resistance by pathogen−drug combinations, 2019

	Resistance to 1+	Anti−pseudomonal	BL−BLI	CP	FQ	4GC	3GC	AG	Vanco	Aminopenicillin	TMP−SMX	PCN	Macrolide	Methicillin	Mono RIF	MDR excluding XDR in TB	XDR in TB	MDR in S. Typhi and Paratyphi	Mono INH
Acinetobacter baumannii	90.9	15.9	0.0269	31.7	34.4	0.691	1.01	7.1											
Citrobacter spp.	4.47	0.73		0.987	0.743	0.244	1.64	0.127											
Enterobacter spp.	24.7	9.54		3.67	2.9	4.38		1.35			2.85								
Enterococcus faecalis	20.6				19				1.61										
Enterococcus faecium	41.9				34.3				7.65										
Other enterococci	14.2				13				1.2										
Escherichia coli	159		9.25	52.2	31.3		32.9	8.13		3.19	22.4								
Group A Streptococcus	0.845												0.855						
Group B Streptococcus	6.04				1.11							0.313	4.48						
Haemophilus influenzae	1.03						0.366			0.668									
Klebsiella pneumoniae	152		0.147	49.8	20.3		57.3	15.1			9.45								
Morganella spp.	0.424				0.225	0.0526	0.146												
Mycobacterium tuberculosis	40.8														0.836	25.3	12.2		2.37
Proteus spp.	8.32				1.69		3.54	0.759		1.22	1.13								
Pseudomonas aeruginosa	66.4	13.1		26.7	16.8	3.23	4.47	2.05											
Salmonella Paratyphi	0.109				0.108													0.000464	
Salmonella Typhi	0.961				0.902													0.0558	
Non−typhoidal Salmonella	0.0415				0.0415														
Serratia spp.	9.93	2.29		1.5	0.588	3.8	0.953	0.792											
Shigella spp.	0.0533				0.0533														
Staphylococcus aureus	70.4				6.63				1.94		1.68		10.3	49.9					
Streptococcus pneumoniae	44.1		0.0733	20.3	0.822		0.208				16	4.96	1.77						
All pathogens	758	41.6	9.49	187	185	12.4	102	35.5	12.4	5.06	53.5	5.27	17.5	49.9	0.836	25.3	12.2	0.0591	2.73

Counts
- **< 5k**
- **NA**

Germany Deaths (count) attributable to bacterial antimicrobial resistance by pathogen−drug combinations, 2019

	Resistance to 1+	Anti−pseudomonal	BL−BLI	CP	FQ	4GC	3GC	AG	Vanco	Aminopenicillin	TMP−SMX	PCN	Macrolide	Methicillin	Mono RIF	MDR excluding XDR in TB	XDR in TB	MDR in S. Typhi and Paratyphi	Mono INH
Acinetobacter baumannii	463	99.8	31.3	46.8	45.9	69.3	160	9.83											
Citrobacter spp.	47.4	19.2		5.69	6.53	1.49	13.6	0.952											
Enterobacter spp.	257	120		53	22.5	30.8		5.12			25.5								
Enterococcus faecalis	287				267				20										
Enterococcus faecium	1,180				885				291										
Other enterococci	51.5				32.4				19.2										
Escherichia coli	3,580		1,040	69.4	818		566	155		362	573								
Group A Streptococcus	9.79												9.44						
Group B Streptococcus	112				19.7							3.83	91.9						
Haemophilus influenzae	24.4						5.75			18.7									
Klebsiella pneumoniae	952		282	50.3	152		264	95.6			108								
Morganella spp.	2.58				0.679	0.499	1.4												
Mycobacterium tuberculosis	30.7														0.344	20.6	5.61		4.09
Proteus spp.	114				10.9		35.3	5.06		38.4	23.5								
Pseudomonas aeruginosa	789	177		384	174	26.2	13.4	14											
Salmonella Paratyphi	0.0289				0.0288													9.34e−05	
Salmonella Typhi	4.25				3.4													0.848	
Non−typhoidal Salmonella	1.58				1.58														
Serratia spp.	39.1	5.38		6.16	3.65	2.49	20.7	0.728											
Shigella spp.	2.59				2.59														
Staphylococcus aureus	1,360				209				47.4		46		276	779					
Streptococcus pneumoniae	341		3.66	102	146		8.39				29.3	22.5	28.1						
All pathogens	9,650	421	1,360	717	2,800	131	1,090	286	378	419	806	26.4	404	779	0.344	20.6	5.61	0.844	4.32

Counts
- 5k − <10k
- < 5k
- NA

Greece Deaths (count) attributable to bacterial antimicrobial resistance by pathogen−drug combinations, 2019

	Resistance to 1+	Anti−pseudomonal	BL−BLI	CP	FQ	4GC	3GC	AG	Vanco	Aminopenicillin	TMP−SMX	PCN	Macrolide	Methicillin	Mono RIF	MDR excluding XDR in TB	XDR in TB	MDR in S. Typhi and Paratyphi	Mono INH
Acinetobacter baumannii	94.4	1.5	0.0553	46.3	35.1	0.383	0.439	10.5											
Citrobacter spp.	4.1	1.21		1.26	1.25	0.0565	0.241	0.0828											
Enterobacter spp.	34.9	11.2		7.67	9.13	1.84		0.602			4.42								
Enterococcus faecalis	60.9				50.1				10.8										
Enterococcus faecium	132				86.9				44.6										
Other enterococci	19.7				13				6.65										
Escherichia coli	457		55.9	18.5	122		106	16.6		76.5	61.8								
Group A Streptococcus	3.79												3.53						
Group B Streptococcus	5.76				1.68							0.517	3.47						
Haemophilus influenzae	10.6						6.48			4.13									
Klebsiella pneumoniae	359		6.73	164	63.1		51.2	45.5			27.8								
Morganella spp.	0.429				0.317	0.0479	0.0647												
Mycobacterium tuberculosis	7.04														0.00907	4.61	1.25		1.17
Proteus spp.	17.2				4.93		5.65	0.605		4.26	1.91								
Pseudomonas aeruginosa	191	23.2		96.8	48.1	5.91	9.69	7.89											
Salmonella Paratyphi	0.00339				0.00337													1.85e−05	
Salmonella Typhi	0.6				0.544													0.0575	
Non−typhoidal Salmonella	0.000106				0.000106														
Serratia spp.	2.15	0.467		1.19	0.205	0.0943	0.108	0.0865											
Shigella spp.	0.0246				0.0246														
Staphylococcus aureus	499				53.4				8.6		7.74		58.6	370					
Streptococcus pneumoniae	92.9		1.04	15.4	4.14		4.84				23.7	34.5	9.22						
All pathogens	1,990	37.6	63.7	352	494	8.34	184	81.8	70.7	84.7	127	35	75.1	370	0.00907	4.61	1.25	0.055	1.24

Counts
< 5k
NA

Hungary Deaths (count) attributable to bacterial antimicrobial resistance by pathogen−drug combinations, 2019

	Resistance to 1+	Anti−pseudomonal	BL−BLI	CP	FQ	4GC	3GC	AG	Vanco	Aminopenicillin	TMP−SMX	PCN	Macrolide	Methicillin	Mono RIF	MDR excluding XDR in TB	XDR in TB	MDR in S. Typhi and Paratyphi	Mono INH
Acinetobacter baumannii	87.6	14.2	0.377	31.9	26.9	5.76	2.61	6.06											
Citrobacter spp.	7.15	2.89		1.04	0.735	0.158	2.22	0.109											
Enterobacter spp.	32.9	12.2		6.63	4.17	3.42		0.605			5.77								
Enterococcus faecalis	71.9				68.5				3.37										
Enterococcus faecium	169				107				62.5										
Other enterococci	19.6				18.4				1.15										
Escherichia coli	486		68.2	12.7	125		113	33.1		60.2	74.1								
Group A Streptococcus	1.18												1.15						
Group B Streptococcus	16				1.96							0.49	13.7						
Haemophilus influenzae	1.09						0.525			0.569									
Klebsiella pneumoniae	195		14.3	6.65	37.8		80.7	30.6			25.5								
Morganella spp.	0.74				0.348	0.0471	0.345												
Mycobacterium tuberculosis	1.93														0.0112	0.953	0.439		0.515
Proteus spp.	21.8				5.44		9.98	0.386		3.15	2.76								
Pseudomonas aeruginosa	126	18.4		71.5	23	2.2	7.85	2.96											
Salmonella Paratyphi	0.00365				0.00364													1.4e−05	
Salmonella Typhi	1.3				1.1													0.197	
Non−typhoidal Salmonella	0.000146				0.000146														
Serratia spp.	5.43	0.867		2.43	0.819	0.464	0.444	0.413											
Shigella spp.	0.0437				0.0437														
Staphylococcus aureus	250				25.9				5.59		4.37		33.8	181					
Streptococcus pneumoniae	55.9		0.238	21.5	10.2		0.924				13.4	5.28	4.13						
All pathogens	1,550	48.5	83.1	154	457	12.1	218	74.1	72.6	64.1	126	5.77	52.9	181	0.0112	0.953	0.439	0.192	0.553

Counts
< 5k
NA

Iceland Deaths (count) attributable to bacterial antimicrobial resistance by pathogen−drug combinations, 2019

	Resistance to 1+	Anti−pseudomonal	BL−BLI	CP	FQ	4GC	3GC	AG	Vanco	Aminopenicillin	TMP−SMX	PCN	Macrolide	Methicillin	Mono RIF	MDR excluding XDR in TB	XDR in TB	MDR in S. Typhi and Paratyphi	Mono INH
Acinetobacter baumannii	1.26	0.456	0.0845	0.186	0.103	0.0216	0.372	0.0363											
Citrobacter spp.	0.0698	0.029		0.0102	0.00895	0.00209	0.0181	0.00142											
Enterobacter spp.	0.493	0.271		0.11	0.0237	0.0202		0.012			0.0557								
Enterococcus faecalis	0.358				0.304				0.0546										
Enterococcus faecium	1.76				1.61				0.15										
Other enterococci	0.103				0.0768				0.0267										
Escherichia coli	5.88		1.59	0.301	1.2		0.971	0.166		0.59	1.07								
Group A Streptococcus	0.0302												0.0303						
Group B Streptococcus	0.41				0.21							0.0119	0.183						
Haemophilus influenzae	0.088						0.0149			0.0731									
Klebsiella pneumoniae	2.16		0.729	0.117	0.193		0.569	0.205			0.345								
Morganella spp.	0.01				0.00409	0.000837	0.00507												
Mycobacterium tuberculosis	0.0375														5.74e−05	0.0143	0.0039		0.0194
Proteus spp.	0.284				0.0336		0.121	0.0134		0.065	0.0533								
Pseudomonas aeruginosa	1.2	0.239		0.582	0.282	0.0579	0.0158	0.0181											
Salmonella Paratyphi	0.00101				0.001													4.19e−06	
Salmonella Typhi	0.00635				0.00585													0.000497	
Non−typhoidal Salmonella	0.00466				0.00466														
Serratia spp.	0.0524	0.0106		0.0159	0.00425	0.00729	0.0126	0.00174											
Shigella spp.	0.00303				0.00303														
Staphylococcus aureus	2.68				0.402				0.121		0.107		0.341	1.71					
Streptococcus pneumoniae	1.22		0.0078	0.581	0.0574		0.0221				0.379	0.0716	0.104						
All pathogens	18.1	1.01	2.41	1.9	4.52	0.11	2.12	0.454	0.352	0.728	2	0.0835	0.663	1.71	5.74e−05	0.0143	0.0039	0.000545	0.02

Counts

< 5k

NA

Ireland Deaths (count) attributable to bacterial antimicrobial resistance by pathogen−drug combinations, 2019

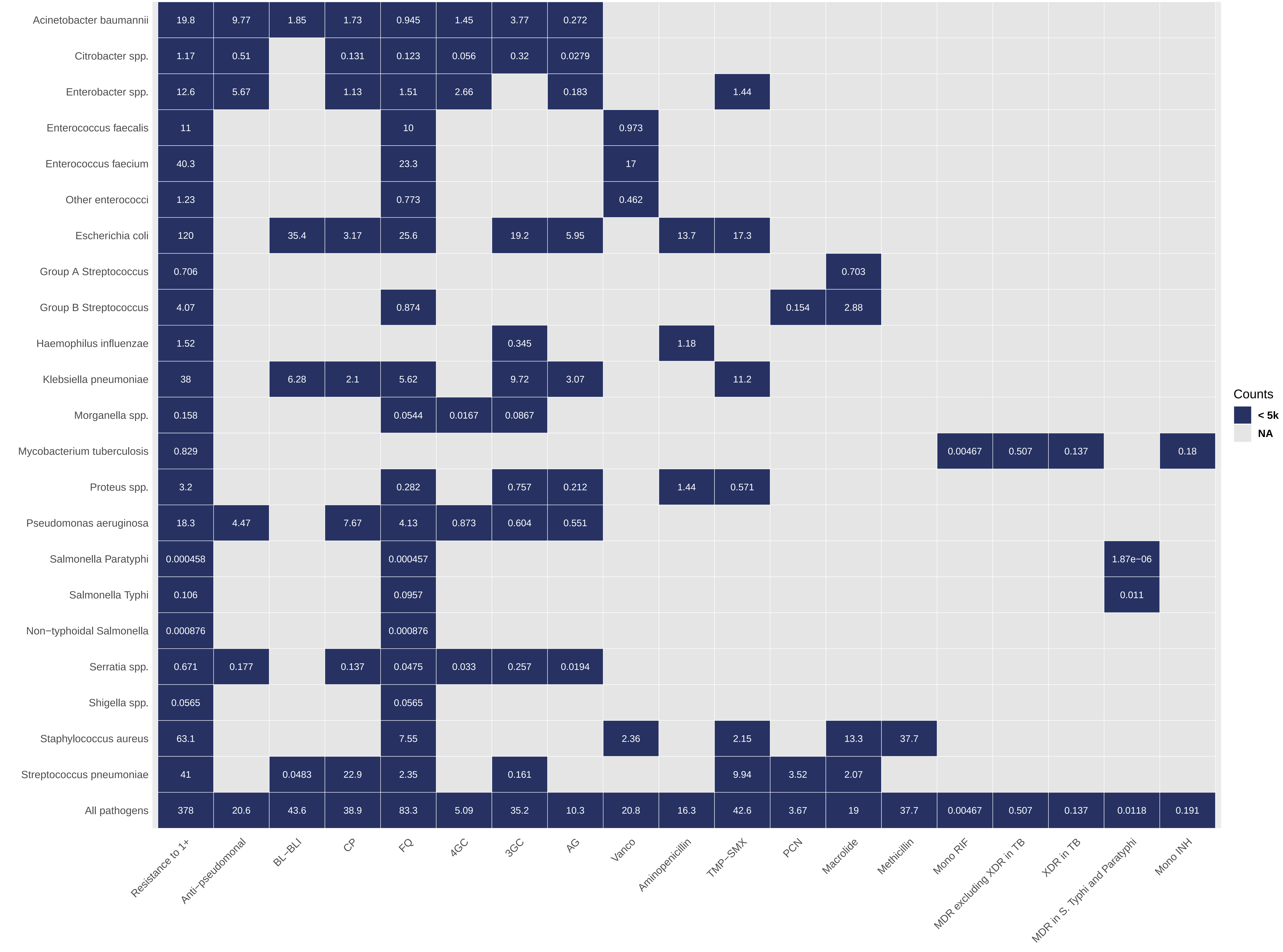

	Resistance to 1+	Anti−pseudomonal	BL−BLI	CP	FQ	4GC	3GC	AG	Vanco	Aminopenicillin	TMP−SMX	PCN	Macrolide	Methicillin	Mono RIF	MDR excluding XDR in TB	XDR in TB	MDR in S. Typhi and Paratyphi	Mono INH
Acinetobacter baumannii	19.8	9.77	1.85	1.73	0.945	1.45	3.77	0.272											
Citrobacter spp.	1.17	0.51		0.131	0.123	0.056	0.32	0.0279											
Enterobacter spp.	12.6	5.67		1.13	1.51	2.66		0.183			1.44								
Enterococcus faecalis	11				10				0.973										
Enterococcus faecium	40.3				23.3				17										
Other enterococci	1.23				0.773				0.462										
Escherichia coli	120		35.4	3.17	25.6		19.2	5.95		13.7	17.3								
Group A Streptococcus	0.706												0.703						
Group B Streptococcus	4.07				0.874							0.154	2.88						
Haemophilus influenzae	1.52						0.345			1.18									
Klebsiella pneumoniae	38		6.28	2.1	5.62		9.72	3.07			11.2								
Morganella spp.	0.158				0.0544	0.0167	0.0867												
Mycobacterium tuberculosis	0.829														0.00467	0.507	0.137		0.18
Proteus spp.	3.2				0.282		0.757	0.212		1.44	0.571								
Pseudomonas aeruginosa	18.3	4.47		7.67	4.13	0.873	0.604	0.551											
Salmonella Paratyphi	0.000458				0.000457													1.87e−06	
Salmonella Typhi	0.106				0.0957													0.011	
Non−typhoidal Salmonella	0.000876				0.000876														
Serratia spp.	0.671	0.177		0.137	0.0475	0.033	0.257	0.0194											
Shigella spp.	0.0565				0.0565														
Staphylococcus aureus	63.1				7.55				2.36		2.15		13.3	37.7					
Streptococcus pneumoniae	41		0.0483	22.9	2.35		0.161				9.94	3.52	2.07						
All pathogens	378	20.6	43.6	38.9	83.3	5.09	35.2	10.3	20.8	16.3	42.6	3.67	19	37.7	0.00467	0.507	0.137	0.0118	0.191

Israel Deaths (count) attributable to bacterial antimicrobial resistance by pathogen−drug combinations, 2019

	Resistance to 1+	Anti−pseudomonal	BL−BLI	CP	FQ	4GC	3GC	AG	Vanco	Aminopenicillin	TMP−SMX	PCN	Macrolide	Methicillin	Mono RIF	MDR excluding XDR in TB	XDR in TB	MDR in S. Typhi and Paratyphi	Mono INH
Acinetobacter baumannii	47.5	7.51	0.255	16.8	16.7	0.054	2.06	4.09											
Citrobacter spp.	4.18	0.906		0.432	0.563	0.0906	2.14	0.0484											
Enterobacter spp.	16.5	4.28		7.71	1.64	0.835		0.312			1.69								
Enterococcus faecalis	32.6				29.8				2.77										
Enterococcus faecium	57				41.9				15.1										
Other enterococci	8.71				6.46				2.25										
Escherichia coli	283		53.7	11.4	68.8		67.9	5.14		16.8	58.9								
Group A Streptococcus	1.63												1.71						
Group B Streptococcus	8.14				2.45							0.292	5.32						
Haemophilus influenzae	2.28						0.427			1.86									
Klebsiella pneumoniae	104		9.53	8.88	13.9		49.5	8.6			13.4								
Morganella spp.	0.598				0.349	0.0515	0.198												
Mycobacterium tuberculosis	2.43														0.017	1.67	0.455		0.27
Proteus spp.	15.5				3.54		6.84	0.235		2.48	2.51								
Pseudomonas aeruginosa	52.8	7.67		29.4	14	0.146	1.23	0.494											
Salmonella Paratyphi	0.00115				0.00114													5.19e−06	
Salmonella Typhi	0.347				0.301													0.0476	
Non−typhoidal Salmonella	0.0261				0.0261														
Serratia spp.	1.6	0.258		0.293	0.116	0.0781	0.812	0.0445											
Shigella spp.	0.177				0.177														
Staphylococcus aureus	222				28.4				3.01		2.52		23	165					
Streptococcus pneumoniae	42.9		0.958	19	2.13		0.572				10.1	5.3	4.82						
All pathogens	903	20.6	64.4	93.9	231	1.25	132	18.9	23.1	21.1	89.1	5.59	34.9	165	0.017	1.67	0.455	0.0467	0.294

Counts
< 5k
NA

Italy Deaths (count) attributable to bacterial antimicrobial resistance by pathogen−drug combinations, 2019

	Resistance to 1+	Anti−pseudomonal	BL−BLI	CP	FQ	4GC	3GC	AG	Vanco	Aminopenicillin	TMP−SMX	PCN	Macrolide	Methicillin	Mono RIF	MDR excluding XDR in TB	XDR in TB	MDR in S. Typhi and Paratyphi	Mono INH
Acinetobacter baumannii	439	20.9	4.38	204	142	4.19	15.9	48.3											
Citrobacter spp.	35.8	13.2		3.62	4.48	1.93	12.1	0.46											
Enterobacter spp.	159	55.6		56.5	19	4.57		4.5			18.5								
Enterococcus faecalis	362				336				25.5										
Enterococcus faecium	700				523				177										
Other enterococci	72.4				65.5				6.83										
Escherichia coli	2,740		379	52.4	824		721	179		243	337								
Group A Streptococcus	10.1												9.99						
Group B Streptococcus	80.8				11.1							2.97	67.4						
Haemophilus influenzae	15						2.94			12									
Klebsiella pneumoniae	1,290		69.4	402	238		273	179			124								
Morganella spp.	4.37				2.95	0.159	1.27												
Mycobacterium tuberculosis	21.7														0.675	13.3	3.62		3.88
Proteus spp.	168				38.5		89.1	7.07		9.56	23.5								
Pseudomonas aeruginosa	603	96.2		288	158	16	30.4	15.1											
Salmonella Paratyphi	0.0189				0.0188													6.5e−05	
Salmonella Typhi	2.34				2.12													0.209	
Non−typhoidal Salmonella	0.00874				0.00874														
Serratia spp.	10.5	2.34		2.89	1.42	0.997	2.15	0.719											
Shigella spp.	0.938				0.938														
Staphylococcus aureus	1,840				254				31.5		23		216	1,310					
Streptococcus pneumoniae	239		1.33	108	28.1		4.84				23.8	28.5	45.3						
All pathogens	8,780	188	454	1,120	2,650	27.8	1,150	433	241	265	549	31.5	339	1,310	0.675	13.3	3.62	0.237	4.18

Counts
- **5k − <10k**
- **< 5k**
- **NA**

Kazakhstan Deaths (count) attributable to bacterial antimicrobial resistance by pathogen−drug combinations, 2019

	Resistance to 1+	Anti−pseudomonal	BL−BLI	CP	FQ	4GC	3GC	AG	Vanco	Aminopenicillin	TMP−SMX	PCN	Macrolide	Methicillin	Mono RIF	MDR excluding XDR in TB	XDR in TB	MDR in S. Typhi and Paratyphi	Mono INH
Acinetobacter baumannii	235	28.1	0.0781	94.1	84	0.202	3.36	24.9											
Citrobacter spp.	13.2	1.96		3.57	2.07	0.67	4.68	0.276											
Enterobacter spp.	75.9	31.4		10.5	9.19	11.8		3.65			9.17								
Enterococcus faecalis	74.2				68.5				5.7										
Enterococcus faecium	154				123				30.3										
Other enterococci	41.1				36.8				4.28										
Escherichia coli	387		93.5	57.1	40.9		37.9	31.9		40.9	84.3								
Group A Streptococcus	2.48												2.56						
Group B Streptococcus	23.9				5.8							1.4	16.4						
Haemophilus influenzae	3.85						1.34			2.51									
Klebsiella pneumoniae	398		22.8	28.4	70.5		169	46.8			60.3								
Morganella spp.	1.33				0.625	0.18	0.529												
Mycobacterium tuberculosis	214														6.76	128	62.4		17
Proteus spp.	25.5				4.64		11.1	2.19		3.96	3.85								
Pseudomonas aeruginosa	246	48.1		102	64.5	8.58	15.2	7.92											
Salmonella Paratyphi	0.251				0.25													0.00112	
Salmonella Typhi	3.47				2.94													0.531	
Non−typhoidal Salmonella	0.101				0.101														
Serratia spp.	28.3	6.52		4.49	1.72	9.33	3.97	2.31											
Shigella spp.	0.232				0.232														
Staphylococcus aureus	297				34.4				7.49		7.07		30.5	217					
Streptococcus pneumoniae	158		0.721	59.2	3.99		2.37				71.3	14.2	6.08						
All pathogens	2,380	116	117	359	554	30.7	250	120	47.8	47.2	236	15.6	55.7	217	6.76	128	62.4	0.477	16.8

Counts
< 5k
NA

Kyrgyzstan Deaths (count) attributable to bacterial antimicrobial resistance by pathogen−drug combinations, 2019

	Resistance to 1+	Anti−pseudomonal	BL−BLI	CP	FQ	4GC	3GC	AG	Vanco	Aminopenicillin	TMP−SMX	PCN	Macrolide	Methicillin	Mono RIF	MDR excluding XDR in TB	XDR in TB	MDR in S. Typhi and Paratyphi	Mono INH
Acinetobacter baumannii	54.4	7.72	0.0159	20.6	19.9	0.027	0.477	5.54											
Citrobacter spp.	4.12	0.409		0.945	1.15	0.356	1.11	0.143											
Enterobacter spp.	19.3	8.05		3.16	1.43	2.67		1			2.98								
Enterococcus faecalis	19.1				17.3				1.75										
Enterococcus faecium	35				28.7				6.34										
Other enterococci	9.82				9.42				0.398										
Escherichia coli	125		14.4	13.6	28.8		38	6.1		4.74	19.5								
Group A Streptococcus	0.559												0.548						
Group B Streptococcus	7.32				2.44							0.498	4.28						
Haemophilus influenzae	1.27						0.782			0.492									
Klebsiella pneumoniae	107		4.24	11.6	13.6		45.3	13.3			18.5								
Morganella spp.	0.408				0.214	0.087	0.107												
Mycobacterium tuberculosis	130														9.47	74.2	36.5		10.3
Proteus spp.	7.56				1.01		3.89	0.585		0.905	1.2								
Pseudomonas aeruginosa	59.1	11.7		20.7	16.3	2.4	5.65	2.33											
Salmonella Paratyphi	0.00865				0.00861													4.07e−05	
Salmonella Typhi	1.58				1.45													0.128	
Non−typhoidal Salmonella	0.0354				0.0354														
Serratia spp.	7.2	1.88		1.5	0.534	1.81	0.982	0.477											
Shigella spp.	0.364				0.364														
Staphylococcus aureus	99.1				7.95				1.44		5.56		8.24	75.9					
Streptococcus pneumoniae	43.6		0.447	13.1	1.02		0.823				21.1	5.83	1.36						
All pathogens	732	29.8	19.1	85.1	152	7.36	97.1	29.7	9.92	6.14	68.7	6.33	14.5	75.9	9.47	74.2	36.5	0.13	10.7

Counts
< 5k
NA

Latvia Deaths (count) attributable to bacterial antimicrobial resistance by pathogen−drug combinations, 2019

	Resistance to 1+	Anti−pseudomonal	BL−BLI	CP	FQ	4GC	3GC	AG	Vanco	Aminopenicillin	TMP−SMX	PCN	Macrolide	Methicillin	Mono RIF	MDR excluding XDR in TB	XDR in TB	MDR in S. Typhi and Paratyphi	Mono INH
Acinetobacter baumannii	22.9	2.47	0.0455	9.86	7.97	0.00646	0.568	1.86											
Citrobacter spp.	1.45	0.494		0.478	0.166	0.0275	0.268	0.0195											
Enterobacter spp.	4.63	1.01		0.912	0.973	0.417		0.138			1.16								
Enterococcus faecalis	19.1				16.2				2.88										
Enterococcus faecium	27.5				17.6				9.92										
Other enterococci	4.21				3.9				0.305										
Escherichia coli	87.3		11.4	3.64	19.3		23.4	3.12		11.8	14.5								
Group A Streptococcus	0.477												0.459						
Group B Streptococcus	3.32				0.352							0.0934	2.85						
Haemophilus influenzae	0.166						0.11			0.0567									
Klebsiella pneumoniae	41.4		4.07	1.95	7.61		15.3	4.85			7.57								
Morganella spp.	0.237				0.0271	0.0232	0.186												
Mycobacterium tuberculosis	8.66														0.00611	5.04	2.4		1.15
Proteus spp.	4.57				1.06		1.89	0.186		0.838	0.578								
Pseudomonas aeruginosa	28.1	5.79		9.31	7.78	1.81	2.45	0.946											
Salmonella Paratyphi	0.000486				0.000484													1.98e−06	
Salmonella Typhi	0.29				0.25													0.0393	
Non−typhoidal Salmonella	0.0413				0.0413														
Serratia spp.	1.29	0.211		0.485	0.0531	0.116	0.323	0.108											
Shigella spp.	0.00962				0.00962														
Staphylococcus aureus	18.3				1.08				1.15		1.17		4.75	10.2					
Streptococcus pneumoniae	12.3		0.109	4.27	1.26		0.137				5.15	0.797	0.544						
All pathogens	286	9.97	15.7	30.9	85.7	2.39	44.6	11.3	14.2	12.7	30.2	0.89	8.65	10.2	0.00611	5.04	2.4	0.0399	1.21

Counts: **< 5k** · **NA**

Lithuania Deaths (count) attributable to bacterial antimicrobial resistance by pathogen−drug combinations, 2019

	Resistance to 1+	Anti−pseudomonal	BL−BLI	CP	FQ	4GC	3GC	AG	Vanco	Aminopenicillin	TMP−SMX	PCN	Macrolide	Methicillin	Mono RIF	MDR excluding XDR in TB	XDR in TB	MDR in S. Typhi and Paratyphi	Mono INH
Acinetobacter baumannii	31.7	0.865	0.128	14.9	11.4	0.24	0.719	3.3											
Citrobacter spp.	2.52	0.869		0.241	0.339	0.0298	1.01	0.0277											
Enterobacter spp.	9.85	4.85		1.56	0.626	1		0.223			1.55								
Enterococcus faecalis	4.19				2.05				2.14										
Enterococcus faecium	39				23.5				15.6										
Other enterococci	5.66				5.23				0.433										
Escherichia coli	125		16.5	5.51	22.5		24.7	4.59		17	34.7								
Group A Streptococcus	1.53												1.58						
Group B Streptococcus	4.63				0.498							0.108	3.93						
Haemophilus influenzae	0.274						0.233			0.0413									
Klebsiella pneumoniae	79.1		3.13	3.3	14.9		30.9	8.75			18.1								
Morganella spp.	0.339				0.0123	0.0339	0.293												
Mycobacterium tuberculosis	35.4														0.671	22	10.7		2.11
Proteus spp.	6.42				1.7		2.51	0.204		1.23	0.807								
Pseudomonas aeruginosa	32.3	6.14		12.9	6.81	5.22	0.504	0.739											
Salmonella Paratyphi	0.00233				0.00232													1.05e−05	
Salmonella Typhi	0.473				0.395													0.078	
Non−typhoidal Salmonella	5.35e−05				5.35e−05														
Serratia spp.	2.64	0.259		0.325	0.0877	0.93	0.988	0.0458											
Shigella spp.	0.0109				0.0109														
Staphylococcus aureus	29.6				1.23				1.47		1.65		7.66	17.6					
Streptococcus pneumoniae	21.2		0.269	5.63	1.42		0.628				8.05	3.15	2.06						
All pathogens	432	13	20	44.3	92.7	7.45	62.4	18	19.6	18.2	64.9	3.26	15.1	17.6	0.671	22	10.7	0.0811	2.09

Counts
< 5k
NA

Luxembourg Deaths (count) attributable to bacterial antimicrobial resistance by pathogen−drug combinations, 2019

	Resistance to 1+	Anti−pseudomonal	BL−BLI	CP	FQ	4GC	3GC	AG	Vanco	Aminopenicillin	TMP−SMX	PCN	Macrolide	Methicillin	Mono RIF	MDR excluding XDR in TB	XDR in TB	MDR in S. Typhi and Paratyphi	Mono INH
Acinetobacter baumannii	2.7	1.55	0.133	0.12	0.231	0.0893	0.55	0.031											
Citrobacter spp.	0.207	0.0906		0.023	0.0221	0.00635	0.0617	0.00352											
Enterobacter spp.	0.985	0.507		0.244	0.102	0.0367		0.0199			0.0737								
Enterococcus faecalis	1.24				1.02				0.225										
Enterococcus faecium	4.73				4.48				0.247										
Other enterococci	0.292				0.241				0.0513										
Escherichia coli	15.3		3.62	0.501	3.71		2.56	0.588		1.96	2.38								
Group A Streptococcus	0.0836												0.0869						
Group B Streptococcus	0.665				0.278							0.0245	0.37						
Haemophilus influenzae	0.156						0.0337			0.123									
Klebsiella pneumoniae	5.41		0.727	0.319	1.04		2.35	0.632			0.335								
Morganella spp.	0.0137				0.00683	0.00119	0.0057												
Mycobacterium tuberculosis	0.0827														5.3e−05	0.0543	0.0147		0.0143
Proteus spp.	0.651				0.119		0.256	0.0315		0.149	0.0929								
Pseudomonas aeruginosa	3.11	0.785		0.958	1.04	0.0648	0.21	0.056											
Salmonella Paratyphi	0.00179				0.00179													6.04e−06	
Salmonella Typhi	0.0312				0.0292													0.00192	
Non−typhoidal Salmonella	0.0238				0.0238														
Serratia spp.	0.136	0.025		0.0316	0.0107	0.00854	0.0564	0.00331											
Shigella spp.	0.0117				0.0117														
Staphylococcus aureus	6.98				0.817				0.27		0.199		1.45	4.25					
Streptococcus pneumoniae	1.94		0.0637	0.823	0.124		0.0441				0.489	0.227	0.172						
All pathogens	44.8	2.95	4.54	3.02	13.3	0.207	6.13	1.37	0.794	2.24	3.57	0.252	2.06	4.25	5.3e−05	0.0543	0.0147	0.00208	0.0144

Counts
- < 5k
- NA

Malta Deaths (count) attributable to bacterial antimicrobial resistance by pathogen−drug combinations, 2019

	Resistance to 1+	Anti−pseudomonal	BL−BLI	CP	FQ	4GC	3GC	AG	Vanco	Aminopenicillin	TMP−SMX	PCN	Macrolide	Methicillin	Mono RIF	MDR excluding XDR in TB	XDR in TB	MDR in S. Typhi and Paratyphi	Mono INH
Acinetobacter baumannii	4.8	2.85	0.113	0.345	0.288	0.118	0.967	0.12											
Citrobacter spp.	0.307	0.111		0.0163	0.0472	0.0134	0.114	0.00465											
Enterobacter spp.	1.16	0.479		0.361	0.136	0.0575		0.0269			0.0974								
Enterococcus faecalis	1.1				0.916				0.182										
Enterococcus faecium	3.12				2.92				0.206										
Other enterococci	0.531				0.371				0.16										
Escherichia coli	13		1.88	0.141	4.85		2.1	0.828		1.72	1.46								
Group A Streptococcus	0.206												0.192						
Group B Streptococcus	0.537				0.141							0.041	0.363						
Haemophilus influenzae	0.256						0.0514			0.205									
Klebsiella pneumoniae	7.89		0.251	2.46	1.35		1.96	1.26			0.605								
Morganella spp.	0.0249				0.013	0.00346	0.00839												
Mycobacterium tuberculosis	0.027														1.4e−05	0.0117	0.00311		0.0119
Proteus spp.	0.647				0.106		0.333	0.025		0.0852	0.0895								
Pseudomonas aeruginosa	3.36	1.66		0.737	0.542	0.205	0.166	0.0531											
Salmonella Paratyphi	0.000271				0.00027													8.83e−07	
Salmonella Typhi	0.0147				0.0125													0.00226	
Non−typhoidal Salmonella	5.5e−06				5.5e−06														
Serratia spp.	0.1	0.0223		0.0263	0.0143	0.00398	0.0294	0.0039											
Shigella spp.	0.00209				0.00209														
Staphylococcus aureus	11.9				1.19				0.194		0.232		1.59	8.66					
Streptococcus pneumoniae	4.32		0.168	1.78	0.15		0.0341				0.743	0.813	0.643						
All pathogens	53.2	5.12	2.41	5.87	13.1	0.401	5.76	2.33	0.743	2.02	3.23	0.854	2.78	8.66	1.4e−05	0.0117	0.00311	0.00239	0.0124

Counts
< 5k
NA

Monaco Deaths (count) attributable to bacterial antimicrobial resistance by pathogen−drug combinations, 2019

	Resistance to 1+	Anti−pseudomonal	BL−BLI	CP	FQ	4GC	3GC	AG	Vanco	Aminopenicillin	TMP−SMX	PCN	Macrolide	Methicillin	Mono RIF	MDR excluding XDR in TB	XDR in TB	MDR in S. Typhi and Paratyphi	Mono INH
Acinetobacter baumannii	0.366	0.0904	0.00589	0.123	0.0672	0.00471	0.0316	0.0423											
Citrobacter spp.	0.0271	0.00971		0.0035	0.00394	0.00137	0.00821	0.000367											
Enterobacter spp.	0.116	0.0509		0.0391	0.00881	0.00442		0.00268			0.0104								
Enterococcus faecalis	0.115				0.099				0.0161										
Enterococcus faecium	0.582				0.424				0.158										
Other enterococci	0.0264				0.0192				0.00721										
Escherichia coli	1.96		0.438	0.0377	0.507		0.45	0.067		0.144	0.313								
Group A Streptococcus	0.0115												0.0112						
Group B Streptococcus	0.128				0.0887							0.00359	0.036						
Haemophilus influenzae	0.0244						0.00513			0.0193									
Klebsiella pneumoniae	0.518		0.176	0.0316	0.0559		0.101	0.0686			0.085								
Morganella spp.	0.00142				0.000642	0.000167	0.000608												
Mycobacterium tuberculosis	0.0243														0.000136	0.0137	0.00365		0.0066
Proteus spp.	0.0745				0.0103		0.0344	0.00283		0.0106	0.0161								
Pseudomonas aeruginosa	0.423	0.0968		0.184	0.0894	0.0085	0.0353	0.00912											
Salmonella Paratyphi	3.09e−06				3.08e−06													1.01e−08	
Salmonella Typhi	0.00328				0.00288													0.000445	
Non−typhoidal Salmonella	0.000795				0.000795														
Serratia spp.	0.0141	0.00308		0.003	0.00128	0.00121	0.00502	0.000502											
Shigella spp.	0.000223				0.000223														
Staphylococcus aureus	1.1				0.192				0.0348		0.0239		0.217	0.631					
Streptococcus pneumoniae	0.378		0.00617	0.128	0.0538		0.00567				0.0934	0.0216	0.0684						
All pathogens	5.89	0.251	0.626	0.55	1.63	0.0204	0.678	0.194	0.216	0.174	0.542	0.0252	0.333	0.631	0.000136	0.0137	0.00365	0.000406	0.00687

Counts
< 5k
NA

Montenegro Deaths (count) attributable to bacterial antimicrobial resistance by pathogen−drug combinations, 2019

	Resistance to 1+	Anti−pseudomonal	BL−BLI	CP	FQ	4GC	3GC	AG	Vanco	Aminopenicillin	TMP−SMX	PCN	Macrolide	Methicillin	Mono RIF	MDR excluding XDR in TB	XDR in TB	MDR in S. Typhi and Paratyphi	Mono INH
Acinetobacter baumannii	12.4	1.7	0.00277	4.93	4.42	0.0414	0.127	1.16											
Citrobacter spp.	1.07	0.29		0.15	0.244	0.0112	0.364	0.0134											
Enterobacter spp.	3.07	0.98		0.555	0.488	0.408		0.113			0.528								
Enterococcus faecalis	2.73				2.56				0.169										
Enterococcus faecium	5.89				4.01				1.87										
Other enterococci	1.72				1.45				0.278										
Escherichia coli	19.5		1.46	1.32	3.42		8.56	1.08		0.855	2.84								
Group A Streptococcus	0.134												0.137						
Group B Streptococcus	0.841				0.477							0.021	0.345						
Haemophilus influenzae	0.0819						0.0275			0.0544									
Klebsiella pneumoniae	12.4		0.0665	0.984	2.01		6.68	1.32			1.37								
Morganella spp.	0.029				0.0139	0.00286	0.0123												
Mycobacterium tuberculosis	0.0734														0.000467	0.0471	0.0221		0.00357
Proteus spp.	1.23				0.153		0.755	0.0363		0.101	0.187								
Pseudomonas aeruginosa	6	0.718		2.91	1.76	0.226	0.201	0.173											
Salmonella Paratyphi	8.53e−05				8.49e−05													3.54e−07	
Salmonella Typhi	0.0486				0.0451													0.00373	
Non−typhoidal Salmonella	0.00744				0.00744														
Serratia spp.	0.463	0.071		0.111	0.0435	0.117	0.0903	0.0295											
Shigella spp.	0.000253				0.000253														
Staphylococcus aureus	10.4				0.908				0.186		0.167		0.945	8.21					
Streptococcus pneumoniae	4.06		0.0405	1.77	0.139		0.054				0.959	0.634	0.456						
All pathogens	82.2	3.76	1.57	12.7	22.2	0.807	16.9	3.94	2.51	1.01	6.05	0.655	1.89	8.21	0.000467	0.0471	0.0221	0.00331	0.0039

Counts
< 5k
NA

Netherlands Deaths (count) attributable to bacterial antimicrobial resistance by pathogen−drug combinations, 2019

	Resistance to 1+	Anti−pseudomonal	BL−BLI	CP	FQ	4GC	3GC	AG	Vanco	Aminopenicillin	TMP−SMX	PCN	Macrolide	Methicillin	Mono RIF	MDR excluding XDR in TB	XDR in TB	MDR in S. Typhi and Paratyphi	Mono INH
Acinetobacter baumannii	102	51.1	6.33	7.58	6.7	2.88	25.7	1.93											
Citrobacter spp.	3.61	0.904		0.87	1.15	0.239	0.291	0.149											
Enterobacter spp.	41.8	15.6		15.3	1.72	4.02		1.19			3.91								
Enterococcus faecalis	47.1				43.3				3.82										
Enterococcus faecium	172				168				4.66										
Other enterococci	14.1				11.4				2.68										
Escherichia coli	561		157	14.6	105		66.6	20.8		66.5	130								
Group A Streptococcus	2.14												2.27						
Group B Streptococcus	23.7				3.64							1.31	18.6						
Haemophilus influenzae	2.4						1.71			0.693									
Klebsiella pneumoniae	124		2.45	5.18	23.8		39.4	10.9			42.4								
Morganella spp.	1.36				0.418	0.166	0.773												
Mycobacterium tuberculosis	4.14														0.0257	2.38	0.64		1.15
Proteus spp.	20.3				0.997		5.23	0.717		6.31	7.18								
Pseudomonas aeruginosa	62.3	17.1		18.8	21.2	2.18	1.83	1.18											
Salmonella Paratyphi	0.0116				0.0116													3.56e−05	
Salmonella Typhi	0.508				0.448													0.0625	
Non−typhoidal Salmonella	1.18				1.18														
Serratia spp.	3.96	0.503		1.27	0.399	0.116	1.4	0.27											
Shigella spp.	0.302				0.302														
Staphylococcus aureus	152				15.4				7.43		11.6		54.9	62.8					
Streptococcus pneumoniae	64.8		1.48	10.9	26.3		1.92				18.9	2.4	2.99						
All pathogens	1,410	85.2	167	74.5	431	9.61	145	37.2	18.6	73.4	214	3.7	79	62.8	0.0257	2.38	0.64	0.0609	1.14

Counts: < 5k; NA

North Macedonia Deaths (count) attributable to bacterial antimicrobial resistance by pathogen−drug combinations, 2019

	Resistance to 1+	Anti−pseudomonal	BL−BLI	CP	FQ	4GC	3GC	AG	Vanco	Aminopenicillin	TMP−SMX	PCN	Macrolide	Methicillin	Mono RIF	MDR excluding XDR in TB	XDR in TB	MDR in S. Typhi and Paratyphi	Mono INH
Acinetobacter baumannii	48.6	3.34	0.0106	21.7	17.3	0.0653	1.12	5.22											
Citrobacter spp.	4.1	1.23		0.737	0.725	0.109	1.25	0.0451											
Enterobacter spp.	13.6	3.72		1.56	4.5	1.9		0.421			1.52								
Enterococcus faecalis	9.22				8.48				0.745										
Enterococcus faecium	20.8				15.1				5.72										
Other enterococci	6.37				5.36				1.01										
Escherichia coli	97.6		2	28.3	25.2		21	4.14		1.48	15.5								
Group A Streptococcus	0.566												0.536						
Group B Streptococcus	2.39				0.75							0.111	1.51						
Haemophilus influenzae	0.325						0.101			0.224									
Klebsiella pneumoniae	53.2		0.231	11.3	10.4		18.7	6.03			6.62								
Morganella spp.	0.168				0.0617	0.0283	0.0782												
Mycobacterium tuberculosis	0.909														0.0046	0.592	0.278		0.038
Proteus spp.	4.19				0.916		2.08	0.133		0.458	0.608								
Pseudomonas aeruginosa	26.4	3.93		11.5	6.8	1.2	1.9	1.07											
Salmonella Paratyphi	0.000247				0.000246													8.92e−07	
Salmonella Typhi	0.25				0.233													0.0161	
Non−typhoidal Salmonella	0.024				0.024														
Serratia spp.	2.99	0.489		0.483	0.167	1.46	0.226	0.159											
Shigella spp.	0.00494				0.00494														
Staphylococcus aureus	49.4				3.02				0.552		0.472		2.63	42.7					
Streptococcus pneumoniae	17		0.0648	7.73	0.488		0.35				4.38	2.67	1.29						
All pathogens	358	12.7	2.31	83.3	99.5	4.76	46.7	17.1	8.03	2.16	29.1	2.78	5.98	42.7	0.0046	0.592	0.278	0.0197	0.0349

Counts
< 5k
NA

Norway Deaths (count) attributable to bacterial antimicrobial resistance by pathogen−drug combinations, 2019

	Resistance to 1+	Anti−pseudomonal	BL−BLI	CP	FQ	4GC	3GC	AG	Vanco	Aminopenicillin	TMP−SMX	PCN	Macrolide	Methicillin	Mono RIF	MDR excluding XDR in TB	XDR in TB	MDR in S. Typhi and Paratyphi	Mono INH
Acinetobacter baumannii	24.9	4.87	1.95	7.1	1.04	1.34	7.11	1.52											
Citrobacter spp.	1.29	0.46		0.188	0.17	0.0464	0.394	0.033											
Enterobacter spp.	9.35	4.6		2.32	0.446	0.519		0.291			1.14								
Enterococcus faecalis	10.7				9.5				1.24										
Enterococcus faecium	36.8				34.5				2.3										
Other enterococci	1.98				1.19				0.793										
Escherichia coli	179		28.9	84.7	21.5		9.09	6.46		8.48	19.6								
Group A Streptococcus	0.839												0.824						
Group B Streptococcus	6.35				0.92							0.366	4.95						
Haemophilus influenzae	2.05						0.286			1.76									
Klebsiella pneumoniae	38.5		10.1	1.47	10.3		7.18	3.38			6.1								
Morganella spp.	0.324				0.091	0.0458	0.187												
Mycobacterium tuberculosis	2.01														0.00209	1.22	0.334		0.465
Proteus spp.	5.63				0.409		1.54	0.325		1.98	1.34								
Pseudomonas aeruginosa	19.4	3.85		9.67	3.74	1.23	0.468	0.403											
Salmonella Paratyphi	0.0886				0.0883													0.000281	
Salmonella Typhi	0.13				0.119													0.011	
Non−typhoidal Salmonella	0.0609				0.0609														
Serratia spp.	0.958	0.212		0.374	0.0615	0.029	0.24	0.044											
Shigella spp.	0.289				0.289														
Staphylococcus aureus	70.5				4.06				2.81		2.6		3.85	57.2					
Streptococcus pneumoniae	15.9		0.253	5.48	1.54		0.755				5.19	1.64	1.02						
All pathogens	427	14	41.1	111	89.9	3.22	27.3	12.5	7.14	12.3	36	2	10.7	57.2	0.00209	1.22	0.334	0.011	0.446

Counts
< 5k
NA

Poland Deaths (count) attributable to bacterial antimicrobial resistance by pathogen−drug combinations, 2019

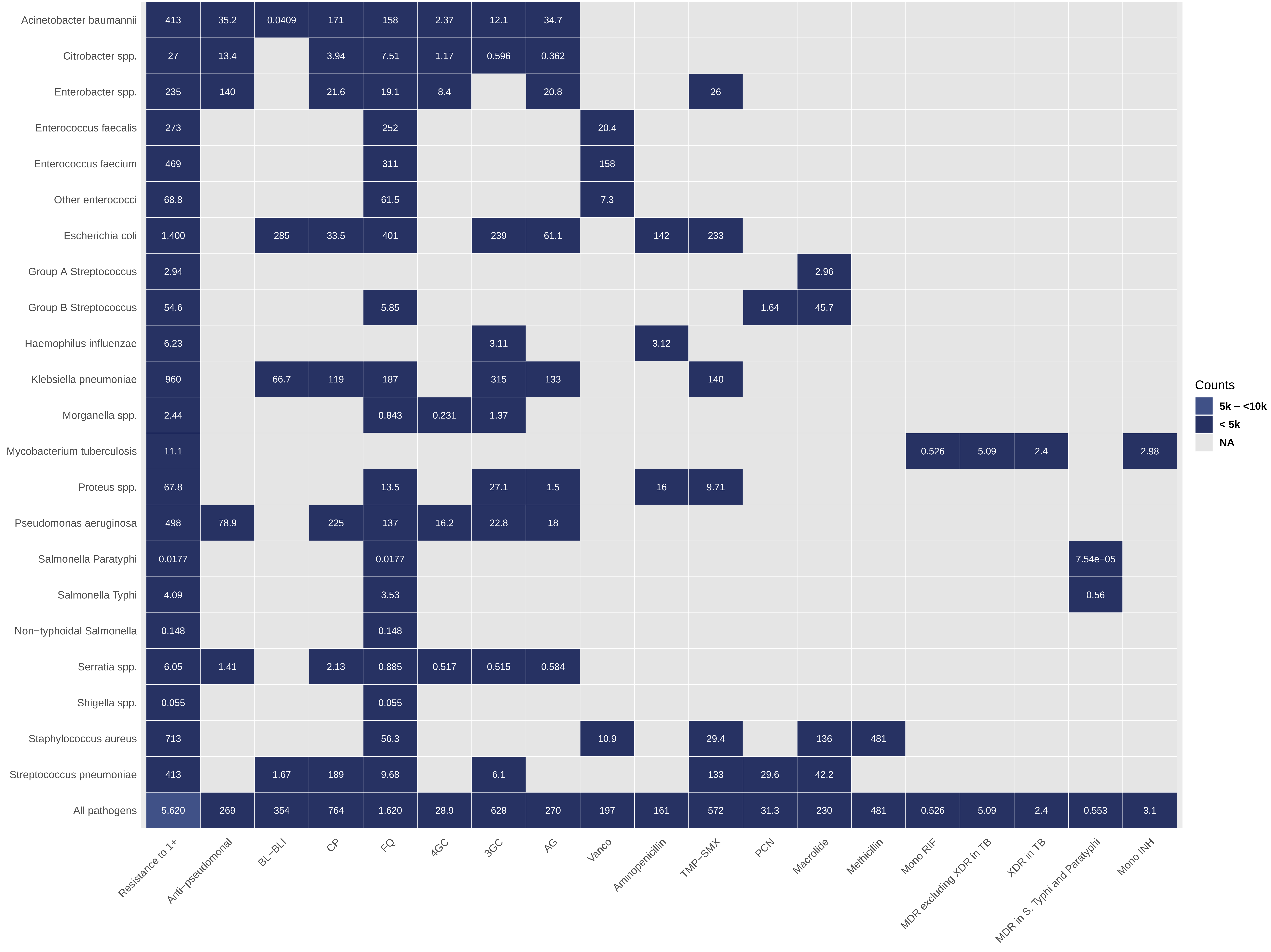

Portugal Deaths (count) attributable to bacterial antimicrobial resistance by pathogen−drug combinations, 2019

	Resistance to 1+	Anti−pseudomonal	BL−BLI	CP	FQ	4GC	3GC	AG	Vanco	Aminopenicillin	TMP−SMX	PCN	Macrolide	Methicillin	Mono RIF	MDR excluding XDR in TB	XDR in TB	MDR in S. Typhi and Paratyphi	Mono INH
Acinetobacter baumannii	97.1	29.7	1.68	31.8	20.4	1.27	7.33	4.85											
Citrobacter spp.	8.89	4.03		0.811	0.638	0.166	3.12	0.123											
Enterobacter spp.	47.4	20.7		19.1	2.8	1.31		0.766			2.67								
Enterococcus faecalis	71.4				66.2				5.2										
Enterococcus faecium	135				114				20.8										
Other enterococci	11.7				8.3				3.43										
Escherichia coli	555		96.4	15.8	128		103	33.8		70.1	108								
Group A Streptococcus	1.8												1.89						
Group B Streptococcus	22.7				2.37							0.68	19.2						
Haemophilus influenzae	6.37						1.6			4.77									
Klebsiella pneumoniae	325		20.4	64	60.4		99.6	43.6			37.3								
Morganella spp.	1.75				0.646	0.449	0.655												
Mycobacterium tuberculosis	7.45														0.0841	4.02	1.09		2.31
Proteus spp.	21.2				1.59		10.1	0.735		3.34	5.79								
Pseudomonas aeruginosa	174	50.7		60.7	39.2	3.51	15.2	4.33											
Salmonella Paratyphi	0.0189				0.0188													6.16e−05	
Salmonella Typhi	0.597				0.529													0.0684	
Non−typhoidal Salmonella	0.000122				0.000122														
Serratia spp.	3.2	0.758		0.624	0.376	0.219	1.13	0.0949											
Shigella spp.	0.149				0.149														
Staphylococcus aureus	602				72.7				9.94		8.17		78.4	433					
Streptococcus pneumoniae	133		13.4	64	3.84		3.42				24.9	11.7	11.1						
All pathogens	2,230	106	132	257	522	6.92	245	88.4	39.3	78.2	187	12.4	111	433	0.0841	4.02	1.09	0.0725	2.31

Counts
< 5k
NA

Republic of Moldova Deaths (count) attributable to bacterial antimicrobial resistance by pathogen−drug combinations, 2019

	Resistance to 1+	Anti−pseudomonal	BL−BLI	CP	FQ	4GC	3GC	AG	Vanco	Aminopenicillin	TMP−SMX	PCN	Macrolide	Methicillin	Mono RIF	MDR excluding XDR in TB	XDR in TB	MDR in S. Typhi and Paratyphi	Mono INH
Acinetobacter baumannii	36.2	2.04	0.00531	16.2	13.5	0.134	0.346	3.78											
Citrobacter spp.	3.47	0.717		0.544	0.612	0.136	1.37	0.0859											
Enterobacter spp.	17.9	6.17		3.04	2.17	3.67		0.649			2.13								
Enterococcus faecalis	32.8				30.3				2.55										
Enterococcus faecium	54.1				40.8				13.4										
Other enterococci	10.6				9.19				1.39										
Escherichia coli	195		20.8	14.6	44.1		64.2	9.53		8.78	32.7								
Group A Streptococcus	0.633												0.589						
Group B Streptococcus	4.12				0.628							0.315	2.97						
Haemophilus influenzae	0.732						0.299			0.432									
Klebsiella pneumoniae	113		5.39	7.2	18		50.3	16.6			15.9								
Morganella spp.	0.346				0.0773	0.0303	0.238												
Mycobacterium tuberculosis	69.8														1.01	44.6	22.3		1.79
Proteus spp.	7.67				1.86		2.59	0.897		1.29	0.989								
Pseudomonas aeruginosa	58.7	7.19		26.8	15.2	3.56	3.8	2.16											
Salmonella Paratyphi	0.00769				0.00767													2.36e−05	
Salmonella Typhi	1.19				1													0.19	
Non−typhoidal Salmonella	0.122				0.122														
Serratia spp.	5.42	1.33		0.857	0.218	1.77	0.779	0.475											
Shigella spp.	0.0202				0.0202														
Staphylococcus aureus	106				5.03				1.96		1.31		6.89	91.3					
Streptococcus pneumoniae	33.3		0.337	9.64	0.802		1.7				17.8	1.07	2.06						
All pathogens	752	17.4	26.5	79	184	9.3	126	34.3	19.3	10.5	70.9	1.39	12.6	91.3	1.01	44.6	22.3	0.175	1.92

Counts
- < 5k
- NA

Romania Deaths (count) attributable to bacterial antimicrobial resistance by pathogen−drug combinations, 2019

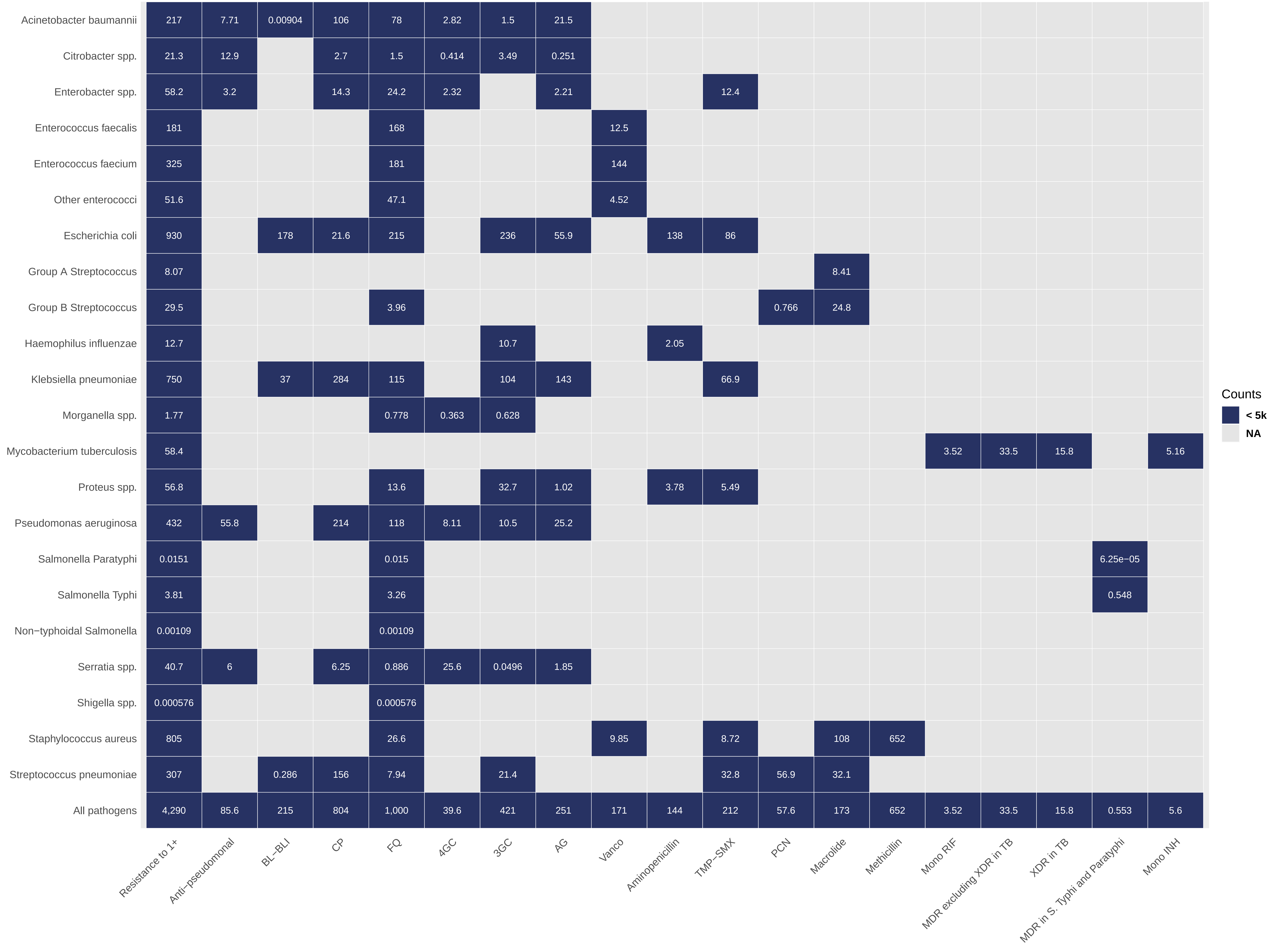

Russian Federation Deaths (count) attributable to bacterial antimicrobial resistance by pathogen−drug combinations, 2019

	Resistance to 1+	Anti−pseudomonal	BL−BLI	CP	FQ	4GC	3GC	AG	Vanco	Aminopenicillin	TMP−SMX	PCN	Macrolide	Methicillin	Mono RIF	MDR excluding XDR in TB	XDR in TB	MDR in S. Typhi and Paratyphi	Mono INH
Acinetobacter baumannii	1,230	70.1	0.551	538	458	1.81	18.6	138											
Citrobacter spp.	78.7	39.7		8.16	13.2	1.94	13.9	1.78											
Enterobacter spp.	809	407		154	26.3	112		23			85.8								
Enterococcus faecalis	1,170				1,080				87.3										
Enterococcus faecium	1,740				1,560				177										
Other enterococci	391				334				56.9										
Escherichia coli	8,550		711	716	1,950		3,240	528		257	1,150								
Group A Streptococcus	22.7												21.4						
Group B Streptococcus	284				37.1							13.5	227						
Haemophilus influenzae	22						14.3			7.68									
Klebsiella pneumoniae	5,860		96.7	2,780	948		578	847			613								
Morganella spp.	16.3				2.76	1.42	12.1												
Mycobacterium tuberculosis	2,260														108	1,350	664		129
Proteus spp.	237				34.4		29.2	77		57.9	37.9								
Pseudomonas aeruginosa	2,930	308		1,440	839	56.2	143	152											
Salmonella Paratyphi	0.437				0.435													0.00165	
Salmonella Typhi	40.1				32.9													7.33	
Non−typhoidal Salmonella	3.5				3.5														
Serratia spp.	235	68.3		66.4	3.29	49.6	13.3	34.4											
Shigella spp.	1.01				1.01														
Staphylococcus aureus	3,010				375				71.5		53.7		190	2,320					
Streptococcus pneumoniae	1,580		7.12	239	44.9		262				831	158	41						
All pathogens	30,500	893	816	5,940	7,740	223	4,330	1,800	393	323	2,770	172	480	2,320	108	1,350	664	7.09	132

Counts

25k − <50k

5k − <10k

< 5k

NA

San Marino Deaths (count) attributable to bacterial antimicrobial resistance by pathogen−drug combinations, 2019

	Resistance to 1+	Anti−pseudomonal	BL−BLI	CP	FQ	4GC	3GC	AG	Vanco	Aminopenicillin	TMP−SMX	PCN	Macrolide	Methicillin	Mono RIF	MDR excluding XDR in TB	XDR in TB	MDR in S. Typhi and Paratyphi	Mono INH
Acinetobacter baumannii	0.281	0.0819	0.00542	0.079	0.0553	0.00173	0.0286	0.0287											
Citrobacter spp.	0.0233	0.00701		0.00563	0.00359	0.000477	0.00624	0.000295											
Enterobacter spp.	0.0748	0.0323		0.024	0.00724	0.00341		0.00165			0.00647								
Enterococcus faecalis	0.0681				0.0575				0.0105										
Enterococcus faecium	0.333				0.269				0.0638										
Other enterococci	0.02				0.0157				0.00436										
Escherichia coli	1.12		0.238	0.022	0.299		0.268	0.0371		0.0717	0.18								
Group A Streptococcus	0.00676												0.00581						
Group B Streptococcus	0.0548				0.0309							0.00203	0.0214						
Haemophilus influenzae	0.0115						0.00332			0.00819									
Klebsiella pneumoniae	0.318		0.107	0.021	0.0256		0.0739	0.0374			0.0534								
Morganella spp.	0.00133				0.000541	0.00015	0.000642												
Mycobacterium tuberculosis	0.0056														2.49e−05	0.00301	0.00082		0.00175
Proteus spp.	0.049				0.00657		0.0225	0.00187		0.00754	0.00965								
Pseudomonas aeruginosa	0.216	0.055		0.095	0.0449	0.00357	0.0129	0.00403											
Salmonella Paratyphi	2.67e−06				2.66e−06													8.75e−09	
Salmonella Typhi	0.00149				0.00132													0.000181	
Non−typhoidal Salmonella	0.000706				0.000706														
Serratia spp.	0.0096	0.00238		0.00219	0.000882	0.00145	0.00244	0.000273											
Shigella spp.	0.000145				0.000145														
Staphylococcus aureus	0.721				0.0868				0.0166		0.011		0.075	0.531					
Streptococcus pneumoniae	0.177		0.00372	0.0598	0.0287		0.00274				0.0391	0.00784	0.0355						
All pathogens	3.49	0.179	0.354	0.309	0.934	0.0108	0.421	0.112	0.0953	0.0877	0.3	0.00987	0.139	0.531	2.49e−05	0.00301	0.00082	0.00017	0.00173

Counts
< 5k
NA

Serbia Deaths (count) attributable to bacterial antimicrobial resistance by pathogen−drug combinations, 2019

	Resistance to 1+	Anti−pseudomonal	BL−BLI	CP	FQ	4GC	3GC	AG	Vanco	Aminopenicillin	TMP−SMX	PCN	Macrolide	Methicillin	Mono RIF	MDR excluding XDR in TB	XDR in TB	MDR in S. Typhi and Paratyphi	Mono INH
Acinetobacter baumannii	140	8.99	0.0402	61.2	50.7	1.92	1.95	15.2											
Citrobacter spp.	14.3	2.58		1.36	2.63	1.1	5.5	1.15											
Enterobacter spp.	73.1	14.2		33.8	11.9	3.05		5.96			4.23								
Enterococcus faecalis	60.9				57.2				3.69										
Enterococcus faecium	120				90.6				29.3										
Other enterococci	25.5				23.4				2.06										
Escherichia coli	441		51.1	12.6	118		133	27.9		29.8	68.9								
Group A Streptococcus	6.03												5.93						
Group B Streptococcus	12.3				1.66							0.502	10.5						
Haemophilus influenzae	1.83						0.597			1.23									
Klebsiella pneumoniae	307		2.95	71.9	44.9		120	36.1			31.6								
Morganella spp.	1.99				0.869	0.112	1.01												
Mycobacterium tuberculosis	2.3														0.0187	1.34	0.632		0.319
Proteus spp.	28.8				6.3		15	3.52		1.9	1.96								
Pseudomonas aeruginosa	158	12		66.9	42.6	13.4	17.5	5.25											
Salmonella Paratyphi	0.00101				0.001													3.51e−06	
Salmonella Typhi	1.36				1.22													0.134	
Non−typhoidal Salmonella	0.0632				0.0632														
Serratia spp.	18.1	0.879		1.11	0.994	11.5	2.44	1.18											
Shigella spp.	0.00789				0.00789														
Staphylococcus aureus	241				32.8				3.89		3.71		39.9	160					
Streptococcus pneumoniae	128		0.272	83.2	3.45		0.21				18.2	11.8	11						
All pathogens	1,780	38.7	54.4	332	489	31	296	96	39	33	129	12.3	67.4	160	0.0187	1.34	0.632	0.151	0.3

Counts
- **< 5k**
- **NA**

Slovakia Deaths (count) attributable to bacterial antimicrobial resistance by pathogen−drug combinations, 2019

	Resistance to 1+	Anti−pseudomonal	BL−BLI	CP	FQ	4GC	3GC	AG	Vanco	Aminopenicillin	TMP−SMX	PCN	Macrolide	Methicillin	Mono RIF	MDR excluding XDR in TB	XDR in TB	MDR in S. Typhi and Paratyphi	Mono INH
Acinetobacter baumannii	39.7	8.24	0.0154	12.1	10.3	6.59	0.857	1.57											
Citrobacter spp.	3.56	0.982		0.86	0.419	0.0597	1.19	0.0463											
Enterobacter spp.	18.9	5.6		4.2	3.73	2.1		0.411			2.91								
Enterococcus faecalis	20.1				19.1				1.01										
Enterococcus faecium	69.3				45.4				23.9										
Other enterococci	8.42				6.97				1.44										
Escherichia coli	229		27.6	3.86	72.8		64.1	12.9		18.6	29.6								
Group A Streptococcus	1.67												1.65						
Group B Streptococcus	6.38				1.26							0.303	4.85						
Haemophilus influenzae	1.64						0.922			0.715									
Klebsiella pneumoniae	130		8.28	11.2	26.1		41	19.8			23.9								
Morganella spp.	0.294				0.139	0.0252	0.129												
Mycobacterium tuberculosis	0.425														0.0187	0.262	0.122		0.0222
Proteus spp.	10.5				2.61		5.02	0.204		1.53	1.18								
Pseudomonas aeruginosa	83.3	10.4		34.6	26.8	5.31	3.09	2.92											
Salmonella Paratyphi	0.00049				0.000487													2.13e−06	
Salmonella Typhi	0.565				0.507													0.0573	
Non−typhoidal Salmonella	6.29e−05				6.29e−05														
Serratia spp.	3.29	0.396		0.744	0.215	1.33	0.486	0.124											
Shigella spp.	0.0051				0.0051														
Staphylococcus aureus	144				16.6				3.1		2.38		20.6	102					
Streptococcus pneumoniae	41.8		0.126	18.1	2.12		0.834				9.31	5.09	6.35						
All pathogens	814	25.6	36	85.7	235	15.4	118	38.1	29.5	20.8	69.2	5.39	33.5	102	0.0187	0.262	0.122	0.0585	0.0226

Counts
< 5k
NA

Slovenia Deaths (count) attributable to bacterial antimicrobial resistance by pathogen−drug combinations, 2019

	Resistance to 1+	Anti−pseudomonal	BL−BLI	CP	FQ	4GC	3GC	AG	Vanco	Aminopenicillin	TMP−SMX	PCN	Macrolide	Methicillin	Mono RIF	MDR excluding XDR in TB	XDR in TB	MDR in S. Typhi and Paratyphi	Mono INH
Acinetobacter baumannii	16.7	3.39	0.439	3.97	3.68	0.0297	4.33	0.827											
Citrobacter spp.	0.809	0.237		0.15	0.135	0.0208	0.246	0.019											
Enterobacter spp.	6.08	2.37		0.976	0.496	1.02		0.126			1.09								
Enterococcus faecalis	12.8				12				0.791										
Enterococcus faecium	22.2				21.1				1.1										
Other enterococci	1.48				1.2				0.28										
Escherichia coli	63		15.4	3.22	17		9.05	2.56		8.57	7.29								
Group A Streptococcus	0.57												0.62						
Group B Streptococcus	1.83				0.534							0.0827	1.25						
Haemophilus influenzae	0.44						0.11			0.33									
Klebsiella pneumoniae	25.7		1.56	0.692	6.3		10.7	1.82			4.61								
Morganella spp.	0.0783				0.0216	0.0125	0.0442												
Mycobacterium tuberculosis	0.0335														0.000127	0.017	0.00794		0.00805
Proteus spp.	2.7				0.797		0.697	0.0548		0.716	0.43								
Pseudomonas aeruginosa	17.9	3.4		7.45	4.45	0.22	1.97	0.391											
Salmonella Paratyphi	0.00164				0.00164													6.22e−06	
Salmonella Typhi	0.0881				0.0801													0.00785	
Non−typhoidal Salmonella	6.3e−05				6.3e−05														
Serratia spp.	0.673	0.0756		0.247	0.0486	0.0157	0.269	0.0175											
Shigella spp.	0.00157				0.00157														
Staphylococcus aureus	30.2				2.39				1.26		1.12		2.49	22.9					
Streptococcus pneumoniae	12.2		0.601	3.08	1.48		0.255				4.92	0.425	1.49						
All pathogens	216	9.47	18	19.8	71.7	1.32	27.7	5.8	3.43	9.61	19.5	0.508	5.8	22.9	0.000127	0.017	0.00794	0.00785	0.00819

Counts
< 5k
NA

Spain Deaths (count) attributable to bacterial antimicrobial resistance by pathogen−drug combinations, 2019

	Resistance to 1+	Anti−pseudomonal	BL−BLI	CP	FQ	4GC	3GC	AG	Vanco	Aminopenicillin	TMP−SMX	PCN	Macrolide	Methicillin	Mono RIF	MDR excluding XDR in TB	XDR in TB	MDR in S. Typhi and Paratyphi	Mono INH
Acinetobacter baumannii	327	28.6	12.2	121	92.6	4.05	49.2	18.5											
Citrobacter spp.	26.5	10.4		3.89	2.97	0.565	8.17	0.471											
Enterobacter spp.	145	56.6		40	23.1	18.2		2.95			4.02								
Enterococcus faecalis	248				235				12.7										
Enterococcus faecium	489				460				29										
Other enterococci	52.6				46.6				5.98										
Escherichia coli	2,030		302	45.9	552		303	111		424	289								
Group A Streptococcus	23.6												22.5						
Group B Streptococcus	43.5				11.5							2.09	30.7						
Haemophilus influenzae	16.6						4.07			12.6									
Klebsiella pneumoniae	611		96.3	66.1	99.5		211	62.8			74.7								
Morganella spp.	5.48				2.66	0.441	2.38												
Mycobacterium tuberculosis	10.8														0.132	5.65	1.54		4.15
Proteus spp.	71.3				9.4		17.7	1.93		18.1	23.4								
Pseudomonas aeruginosa	499	75		252	134	14.4	11.2	13											
Salmonella Paratyphi	0.0263				0.0262													9.84e−05	
Salmonella Typhi	1.74				1.56													0.168	
Non−typhoidal Salmonella	6.29				6.29														
Serratia spp.	11.2	2.4		2.34	1.23	0.756	4.17	0.315											
Shigella spp.	0.329				0.329														
Staphylococcus aureus	1,220				145				26.2		22.7		149	882					
Streptococcus pneumoniae	381		1.19	217	15.7		3.89				60.1	50.8	31.7						
All pathogens	6,220	173	412	749	1,840	38.4	615	211	73.8	455	474	52.9	234	882	0.132	5.65	1.54	0.171	4.03

Counts
5k − <10k
< 5k
NA

Sweden Deaths (count) attributable to bacterial antimicrobial resistance by pathogen−drug combinations, 2019

	Resistance to 1+	Anti−pseudomonal	BL−BLI	CP	FQ	4GC	3GC	AG	Vanco	Aminopenicillin	TMP−SMX	PCN	Macrolide	Methicillin	Mono RIF	MDR excluding XDR in TB	XDR in TB	MDR in S. Typhi and Paratyphi	Mono INH
Acinetobacter baumannii	48.3	22	5.22	3.76	3.11	2.09	11.1	0.972											
Citrobacter spp.	2.09	0.304		0.36	0.375	0.16	0.824	0.0668											
Enterobacter spp.	16	7.4		6.86	0.737	0.15		0.463			0.33								
Enterococcus faecalis	15.4				12.8				2.52										
Enterococcus faecium	79.1				75.8				3.21										
Other enterococci	3.87				2.36				1.52										
Escherichia coli	224		60.4	5.83	62		35	11.2		12.3	37.5								
Group A Streptococcus	2.93												2.74						
Group B Streptococcus	11.6				2.25							0.47	9.83						
Haemophilus influenzae	1.56						0.616			0.946									
Klebsiella pneumoniae	50.7		17.8	2.8	9.54		13.2	3.84			3.43								
Morganella spp.	0.503				0.162	0.0721	0.269												
Mycobacterium tuberculosis	4.68														0.0238	2.8	0.761		1.08
Proteus spp.	9.57				0.834		1.57	0.7		4.56	2.05								
Pseudomonas aeruginosa	35	4.92		18.6	7.71	0.658	2.75	0.398											
Salmonella Paratyphi	0.00998				0.00994													3.23e−05	
Salmonella Typhi	0.239				0.215													0.0232	
Non−typhoidal Salmonella	0.142				0.142														
Serratia spp.	3.45	1.29		0.414	0.108	0.069	1.51	0.0503											
Shigella spp.	1.24				1.24														
Staphylococcus aureus	46.3				3.88				6.43		5.82		5.14	25					
Streptococcus pneumoniae	23.3		0.795	5.38	1.36		1.35				8.59	4.24	1.54						
All pathogens	581	35.9	84.2	44	185	3.2	68.2	17.7	13.7	17.8	57.7	4.71	19.1	25	0.0238	2.8	0.761	0.0241	1.13

Counts
< 5k
NA

Switzerland Deaths (count) attributable to bacterial antimicrobial resistance by pathogen−drug combinations, 2019

	Resistance to 1+	Anti−pseudomonal	BL−BLI	CP	FQ	4GC	3GC	AG	Vanco	Aminopenicillin	TMP−SMX	PCN	Macrolide	Methicillin	Mono RIF	MDR excluding XDR in TB	XDR in TB	MDR in S. Typhi and Paratyphi	Mono INH
Acinetobacter baumannii	45.5	2.29	2.81	18	3.67	0.392	14.6	3.67											
Citrobacter spp.	3.17	0.449		0.319	0.232	0.123	1.99	0.0581											
Enterobacter spp.	11.9	6.49		2.04	0.56	0.634		0.406			1.73								
Enterococcus faecalis	7.37				3.68				3.7										
Enterococcus faecium	69.5				65.2				4.31										
Other enterococci	3.12				1.97				1.15										
Escherichia coli	204		55.4	3.92	43.6		27.7	4.16		30	39.1								
Group A Streptococcus	1.36												1.41						
Group B Streptococcus	8.61				0.882							0.343	7.52						
Haemophilus influenzae	2.3						0.399			1.9									
Klebsiella pneumoniae	38.2		9.18	4.03	6.88		9.91	2.18			6.03								
Morganella spp.	0.387				0.134	0.0444	0.208												
Mycobacterium tuberculosis	2.46														0.00315	1.76	0.477		0.229
Proteus spp.	9.39				1.4		2.48	0.453		3.04	1.98								
Pseudomonas aeruginosa	33.9	8.92		10.2	9.57	1.74	2.94	0.551											
Salmonella Paratyphi	0.00286				0.00285													8.85e−06	
Salmonella Typhi	0.563				0.51													0.056	
Non−typhoidal Salmonella	0.0955				0.0955														
Serratia spp.	1.33	0.381		0.32	0.0939	0.081	0.403	0.046											
Shigella spp.	0.312				0.312														
Staphylococcus aureus	89.3				7.03				4.33		3.6		9.81	64.5					
Streptococcus pneumoniae	29.8		0.185	9.68	2.73		6.11				7.96	0.688	2.38						
All pathogens	563	18.5	67.5	48.5	148	3.01	66.8	11.6	13.5	35	60.4	1.03	21.2	64.5	0.00315	1.76	0.477	0.0518	0.221

Counts
< 5k
NA

Tajikistan Deaths (count) attributable to bacterial antimicrobial resistance by pathogen−drug combinations, 2019

	Resistance to 1+	Anti−pseudomonal	BL−BLI	CP	FQ	4GC	3GC	AG	Vanco	Aminopenicillin	TMP−SMX	PCN	Macrolide	Methicillin	Mono RIF	MDR excluding XDR in TB	XDR in TB	MDR in S. Typhi and Paratyphi	Mono INH
Acinetobacter baumannii	148	17.1	0.0244	58.5	57.5	0.265	0.768	13.4											
Citrobacter spp.	13.6	1.64		4.29	2.63	0.639	3.81	0.56											
Enterobacter spp.	48.9	14.6		10.5	4.09	5.84		3.68			10.4								
Enterococcus faecalis	22.7				20				2.66										
Enterococcus faecium	37.7				30				7.69										
Other enterococci	18.9				18				0.899										
Escherichia coli	185		11.4	23.1	53		55.5	10.2		6.51	25.3								
Group A Streptococcus	0.711												0.689						
Group B Streptococcus	12.4				4.06							0.699	7.73						
Haemophilus influenzae	5.29						3.49			1.8									
Klebsiella pneumoniae	165		5.32	20.6	22.9		70.6	18.4			27.1								
Morganella spp.	0.843				0.511	0.113	0.219												
Mycobacterium tuberculosis	159														12.8	92.3	44.6		10.5
Proteus spp.	12.7				2.1		6.56	1.28		1.11	1.53								
Pseudomonas aeruginosa	89.6	11.7		35.5	17.7	3.87	18.9	1.95											
Salmonella Paratyphi	0.0167				0.0165													0.000224	
Salmonella Typhi	4.22				3.68													0.503	
Non−typhoidal Salmonella	0.301				0.301														
Serratia spp.	12.5	3.21		2.48	1.16	3.06	1.77	0.798											
Shigella spp.	3.88				3.88														
Staphylococcus aureus	142				9.17				1.64		16.5		14	100					
Streptococcus pneumoniae	214		1.53	107	3.6		0.444				69	19	13.3						
All pathogens	1,300	48.3	18.3	262	254	13.8	162	50.5	12.9	9.49	150	19.7	36	100	12.8	92.3	44.6	0.537	9.88

Counts
< 5k
NA

Turkey Deaths (count) attributable to bacterial antimicrobial resistance by pathogen−drug combinations, 2019

	Resistance to 1+	Anti−pseudomonal	BL−BLI	CP	FQ	4GC	3GC	AG	Vanco	Aminopenicillin	TMP−SMX	PCN	Macrolide	Methicillin	Mono RIF	MDR excluding XDR in TB	XDR in TB	MDR in S. Typhi and Paratyphi	Mono INH
Acinetobacter baumannii	824	24.4	1.57	386	307	1.82	12	92.1											
Citrobacter spp.	27.2	7.81		8.14	1.9	0.195	8.8	0.353											
Enterobacter spp.	209	62.9		118	6.86	2.39		4.48			14.3								
Enterococcus faecalis	84.4				56.1				28.3										
Enterococcus faecium	487				370				117										
Other enterococci	156				93.8				62.7										
Escherichia coli	1,450		169	73.8	330		514	99.6		63	205								
Group A Streptococcus	6.76												6.42						
Group B Streptococcus	108				16.7							1.72	90						
Haemophilus influenzae	18						4.01			14									
Klebsiella pneumoniae	1,440		89.9	537	243		247	189			134								
Morganella spp.	4.7				3.09	0.806	0.803												
Mycobacterium tuberculosis	59.8														2.57	41.1	3.14		12.4
Proteus spp.	71.5				8.29		32.5	1.59		13.1	16.5								
Pseudomonas aeruginosa	593	103		302	129	16	28.4	14.8											
Salmonella Paratyphi	0.424				0.422													0.00185	
Salmonella Typhi	10.7				10.2													0.516	
Non−typhoidal Salmonella	1.24				1.24														
Serratia spp.	68.9	15.7		20.5	8.52	15.3	5.93	2.91											
Shigella spp.	0.753				0.753														
Staphylococcus aureus	2,290				90.3				31.6		19.3		99.3	2,050					
Streptococcus pneumoniae	919		0.418	488	12.8		0.908				186	168	62.6						
All pathogens	8,840	214	261	1,930	1,690	36.5	854	404	239	89.5	576	170	259	2,050	2.57	41.1	3.14	0.525	12.8

Counts
- 5k − <10k
- < 5k
- NA

Turkmenistan Deaths (count) attributable to bacterial antimicrobial resistance by pathogen−drug combinations, 2019

	Resistance to 1+	Anti−pseudomonal	BL−BLI	CP	FQ	4GC	3GC	AG	Vanco	Aminopenicillin	TMP−SMX	PCN	Macrolide	Methicillin	Mono RIF	MDR excluding XDR in TB	XDR in TB	MDR in S. Typhi and Paratyphi	Mono INH
Acinetobacter baumannii	60	5.94	0.0209	25.4	21.8	0.0372	0.728	6.29											
Citrobacter spp.	4.85	0.483		1.53	1.39	0.0718	1.22	0.148											
Enterobacter spp.	19.2	5.89		3.98	2.6	2.2		1.16			3.36								
Enterococcus faecalis	14.4				12.1				2.36										
Enterococcus faecium	41.7				26.9				14.9										
Other enterococci	10.5				10				0.521										
Escherichia coli	136		13.6	8.09	35.7		42.8	10.4		5.36	19.9								
Group A Streptococcus	0.517												0.575						
Group B Streptococcus	8.82				4.22							0.889	3.54						
Haemophilus influenzae	3.33						1.83			1.5									
Klebsiella pneumoniae	108		7.74	9.33	14.7		45.6	12.2			18.8								
Morganella spp.	0.417				0.253	0.0697	0.0943												
Mycobacterium tuberculosis	97.6														1.32	57.8	27.9		11.2
Proteus spp.	8.09				1.37		4.25	0.479		0.806	1.19								
Pseudomonas aeruginosa	61.1	9.88		21.2	14.8	2.89	10.4	1.96											
Salmonella Paratyphi	0.00062				0.000618													2.61e−06	
Salmonella Typhi	1.85				1.5													0.356	
Non−typhoidal Salmonella	0.0303				0.0303														
Serratia spp.	6.4	1.59		1.28	0.548	1.62	0.849	0.508											
Shigella spp.	0.231				0.231														
Staphylococcus aureus	82.5				8.99				1.58		7.6		14.6	49.7					
Streptococcus pneumoniae	73.3		0.766	32.8	1.35		0.259				25.4	7.16	5.77						
All pathogens	739	23.8	22.1	104	158	6.89	108	32.9	19.3	7.68	76.3	8.05	24.5	49.7	1.32	57.8	27.9	0.351	11.1

Counts
< 5k
NA

Ukraine Deaths (count) attributable to bacterial antimicrobial resistance by pathogen−drug combinations, 2019

	Resistance to 1+	Anti−pseudomonal	BL−BLI	CP	FQ	4GC	3GC	AG	Vanco	Aminopenicillin	TMP−SMX	PCN	Macrolide	Methicillin	Mono RIF	MDR excluding XDR in TB	XDR in TB	MDR in S. Typhi and Paratyphi	Mono INH
Acinetobacter baumannii	937	62.2	0.146	415	342	5.79	8.44	106											
Citrobacter spp.	77.1	25.4		10.4	15.9	4.8	19.1	1.5											
Enterobacter spp.	278	106		42.4	32.6	55		8.52			33.1								
Enterococcus faecalis	367				339				28.2										
Enterococcus faecium	584				404				181										
Other enterococci	150				126				23.6										
Escherichia coli	1,750		211	113	398		552	85.4		92.2	299								
Group A Streptococcus	10.4												10.8						
Group B Streptococcus	67.1				9.61							4.02	49.8						
Haemophilus influenzae	8.13						2.72			5.41									
Klebsiella pneumoniae	1,120		79.5	116	200		386	187			155								
Morganella spp.	4.19				0.75	0.361	3.08												
Mycobacterium tuberculosis	1,360														82.1	814	401		56.4
Proteus spp.	76.7				14.9		23.6	9.16		17.5	12								
Pseudomonas aeruginosa	709	75.1		345	168	35.4	59.2	25.5											
Salmonella Paratyphi	0.00105				0.00104													3.11e−06	
Salmonella Typhi	10.4				7.17													3.39	
Non−typhoidal Salmonella	3.69				3.69														
Serratia spp.	76.8	17.6		12.9	2.68	23.7	13	6.71											
Shigella spp.	0.491				0.491														
Staphylococcus aureus	317				56.1				29		15.9		30.8	185					
Streptococcus pneumoniae	507		2.87	125	13		37.7				216	78	35.6						
All pathogens	8,410	286	294	1,180	2,130	125	1,110	427	261	115	731	82.1	128	185	82.1	814	401	3.2	59.8

Counts
- 5k − <10k
- < 5k
- NA

United Kingdom Deaths (count) attributable to bacterial antimicrobial resistance by pathogen−drug combinations, 2019

	Resistance to 1+	Anti−pseudomonal	BL−BLI	CP	FQ	4GC	3GC	AG	Vanco	Aminopenicillin	TMP−SMX	PCN	Macrolide	Methicillin	Mono RIF	MDR excluding XDR in TB	XDR in TB	MDR in S. Typhi and Paratyphi	Mono INH
Acinetobacter baumannii	369	6.76	29	19.9	17	5.82	285	5.95											
Citrobacter spp.	44.5	24.7		3.53	2.39	0.382	12.9	0.642											
Enterobacter spp.	185	72.8		65.3	6.75	25.5		4.71			9.83								
Enterococcus faecalis	262				223				38.4										
Enterococcus faecium	821				579				242										
Other enterococci	51.9				34.6				17.3										
Escherichia coli	2,580		750	50.9	461		374	144		287	509								
Group A Streptococcus	11.8												11.3						
Group B Streptococcus	41.6				18.4							3.65	19.7						
Haemophilus influenzae	48.6						7.44			41.2									
Klebsiella pneumoniae	757		163	56.8	94.3		199	56.7			187								
Morganella spp.	5.72				1.76	0.858	3.1												
Mycobacterium tuberculosis	12.4														0.411	5.93	1.61		4.54
Proteus spp.	87.8				7.31		19.9	4.7		24.5	31.7								
Pseudomonas aeruginosa	442	121		179	111	8.93	14.3	7.42											
Salmonella Paratyphi	0.0238				0.0237													8e−05	
Salmonella Typhi	2.32				2.07													0.23	
Non−typhoidal Salmonella	14.4				14.4														
Serratia spp.	21.3	4.41		3.16	1.83	1.01	10.4	0.472											
Shigella spp.	1.63				1.63														
Staphylococcus aureus	1,370				139				48.5		46.7		196	942					
Streptococcus pneumoniae	448		13.9	66.3	125		17.9				166	41.3	16.6						
All pathogens	7,580	230	956	445	1,840	42.5	944	225	346	352	951	45	244	942	0.411	5.93	1.61	0.24	4.13

Counts
- 5k − <10k
- < 5k
- NA

Uzbekistan Deaths (count) attributable to bacterial antimicrobial resistance by pathogen−drug combinations, 2019

	Resistance to 1+	Anti−pseudomonal	BL−BLI	CP	FQ	4GC	3GC	AG	Vanco	Aminopenicillin	TMP−SMX	PCN	Macrolide	Methicillin	Mono RIF	MDR excluding XDR in TB	XDR in TB	MDR in S. Typhi and Paratyphi	Mono INH
Acinetobacter baumannii	305	35.2	0.105	130	107	0.244	3.04	29.8											
Citrobacter spp.	24.6	3.2		6.62	7.12	0.424	6.36	0.826											
Enterobacter spp.	110	37.9		21.6	12.1	15.3		6.18			16.9								
Enterococcus faecalis	81.4				68.8				12.6										
Enterococcus faecium	223				172				51										
Other enterococci	55.9				53.2				2.69										
Escherichia coli	760		89.7	47.5	181		240	48.6		33.3	120								
Group A Streptococcus	4.04												4.43						
Group B Streptococcus	60.1				25.3							6.22	28.3						
Haemophilus influenzae	20.8						12.2			8.65									
Klebsiella pneumoniae	706		27.5	69.4	89		331	73.8			115								
Morganella spp.	2.07				1.15	0.333	0.588												
Mycobacterium tuberculosis	715														55.9	423	207		29.7
Proteus spp.	45.2				6.76		24.8	2.55		4.31	6.59								
Pseudomonas aeruginosa	368	58.9		127	89	21.4	60	10.8											
Salmonella Paratyphi	0.432				0.43													0.00148	
Salmonella Typhi	10.1				9.26													0.897	
Non−typhoidal Salmonella	0.139				0.139														
Serratia spp.	36	9.95		7.28	3.44	5.87	6.51	3.16											
Shigella spp.	0.199				0.199														
Staphylococcus aureus	482				58.1				10.3		39.4		85.2	290					
Streptococcus pneumoniae	444		10.8	183	9.76		2.88				182	19.8	36.1						
All pathogens	4,450	145	128	592	893	43.5	688	176	76.5	46.3	480	26	154	290	55.9	423	207	0.905	28.1

Counts
< 5k
NA
