# Supplementary material for: The burden of bacterial antimicrobial resistance in the WHO European region in 2019: a cross-country systematic analysis
Source: Lancet Public Health. 2022 Oct 14;7(11):e897–913. doi: 10.1016/S2468-2667(22)00225-0 (PMC9630253; doi:10.1016/S2468-2667(22)00225-0)

THE LANCET
Infectious Diseases

Supplementary appendix 3

This appendix formed part of the original submission and has been peer reviewed. We post it as supplied by the authors.

Supplement to: European Antimicrobial Resistance Collaborators. The burden of bacterial antimicrobial resistance in the WHO European region in 2019: a cross-country systematic analysis. *Lancet Infect Dis* 2022; published online Oct 13. https://doi.org/10.1016/S2468-2667(22)00225-0.

Albania Deaths (count) associated with bacterial antimicrobial resistance by pathogen−drug combinations, 2019

	Resistance to 1+	AG	Anti−pseudomonal	BL−BLI	CP	FQ	4GC	3GC	TMP−SMX	Vanco	Aminopenicillin	Macrolide	PCN	MDR in S. Typhi and Paratyphi	Methicillin	Mono INH	Mono RIF	MDR excluding XDR in TB	XDR in TB
Acinetobacter baumannii	57	45	54.8	43.2	48	51.1	47.2	54.9											
Citrobacter spp.	4.51	0.347	1.85		0.659	1.38	0.572	3.77											
Enterobacter spp.	26.7	3.98	14.9		5.77	8.5	14.8		14.9										
Enterococcus faecalis	26.3					25.1				2.36									
Enterococcus faecium	83.4					83.1				31.2									
Other enterococci	19.5					19				5.22									
Escherichia coli	398	86.6		253	51.3	238		244	258		338								
Group A Streptococcus	7.65											7.65							
Group B Streptococcus	23.8					13.9						15.4	0.553						
Haemophilus influenzae	2.38							0.465			2.15								
Klebsiella pneumoniae	187	72.5		162	21	120		164	144										
Morganella spp.	0.48					0.3	0.0986	0.29											
Mycobacterium tuberculosis	0.579															0.312	0.0264	0.165	0.075
Proteus spp.	25.5	1.33				10.5		12.7	17.4		20.4								
Pseudomonas aeruginosa	105	35.9	52.8		56.1	62.9	51.9	75.9											
Salmonella Paratyphi	0.00244					0.00243								1.97e−05					
Salmonella Typhi	2.13					1.91								0.274					
Non−typhoidal Salmonella	0.0332					0.0332													
Serratia spp.	4.75	1.56	0.968		0.832	0.941	2.95	2.66											
Shigella spp.	0.0107					0.0107													
Staphylococcus aureus	224					99.9			13.2	2.77		147			183				
Streptococcus pneumoniae	103			12.6	43.5	3.32		13.7	53.9			70.9	57.2						
All pathogens	1,300	247	125	471	227	739	117	572	502	41.5	361	241	57.8	0.274	183	0.312	0.0264	0.165	0.075

Counts
< 5k
NA

Andorra Deaths (count) associated with bacterial antimicrobial resistance by pathogen−drug combinations, 2019

	Resistance to 1+	AG	Anti−pseudomonal	BL−BLI	CP	FQ	4GC	3GC	TMP−SMX	Vanco	Aminopenicillin	Macrolide	PCN	MDR in S. Typhi and Paratyphi	Methicillin	Mono INH	Mono RIF	MDR excluding XDR in TB	XDR in TB
Acinetobacter baumannii	1.41	0.15	0.89	1.07	0.25	0.283	0.701	1.28											
Citrobacter spp.	0.121	0.00848	0.055		0.00991	0.0303	0.0136	0.103											
Enterobacter spp.	0.659	0.049	0.479		0.159	0.0698	0.242		0.21										
Enterococcus faecalis	0.565					0.527				0.0617									
Enterococcus faecium	3.13					3.12				0.644									
Other enterococci	0.168					0.149				0.0453									
Escherichia coli	12.2	1.15		8.21	0.165	7.17		5.28	6.81		10.4								
Group A Streptococcus	0.11											0.11							
Group B Streptococcus	0.718					0.312						0.545	0.0257						
Haemophilus influenzae	0.122							0.012			0.118								
Klebsiella pneumoniae	3.2	0.756		2.66	0.137	0.988		0.972	2										
Morganella spp.	0.0156					0.00949	0.00199	0.00989											
Mycobacterium tuberculosis	0.0182															0.0164	0.000114	0.00136	0.000359
Proteus spp.	0.958	0.055				0.178		0.18	0.58		0.926								
Pseudomonas aeruginosa	1.61	0.273	0.731		0.858	0.87	0.652	0.65											
Salmonella Paratyphi	3.92e−05					3.92e−05								2.54e−07					
Salmonella Typhi	0.0182					0.0169								0.00165					
Non−typhoidal Salmonella	0.00461					0.00461													
Serratia spp.	0.0793	0.00807	0.0221		0.0141	0.0141	0.0394	0.0565											
Shigella spp.	0.00265					0.00265													
Staphylococcus aureus	4.97					3.1			0.297	0.106		3.14			1.64				
Streptococcus pneumoniae	1.73			0.238	0.503	0.251		0.176	0.981			0.375	0.916						
All pathogens	31.8	2.45	2.18	12.2	2.09	17.1	1.65	8.72	10.9	0.858	11.5	4.17	0.941	0.00165	1.64	0.0164	0.000114	0.00136	0.000359

Counts
< 5k
NA

Armenia Deaths (count) associated with bacterial antimicrobial resistance by pathogen−drug combinations, 2019

	Resistance to 1+	AG	Anti−pseudomonal	BL−BLI	CP	FQ	4GC	3GC	TMP−SMX	Vanco	Aminopenicillin	Macrolide	PCN	MDR in S. Typhi and Paratyphi	Methicillin	Mono INH	Mono RIF	MDR excluding XDR in TB	XDR in TB
Acinetobacter baumannii	134	111	131	111	108	118	127	128											
Citrobacter spp.	10.7	0.667	2.41		2.72	5.07	1.37	7.37											
Enterobacter spp.	69.6	11.2	45.9		9.08	19.1	41.2		29.9										
Enterococcus faecalis	56					54.2				4.22									
Enterococcus faecium	141					140				26.4									
Other enterococci	62.3					62.2				7.29									
Escherichia coli	492	112		339	32.5	319		293	332		431								
Group A Streptococcus	6.1											6.1							
Group B Streptococcus	38.3					11.9						32.5	2.46						
Haemophilus influenzae	2.49							0.573			2.16								
Klebsiella pneumoniae	320	134		285	32.8	203		272	265										
Morganella spp.	2.26					1.43	0.317	1.47											
Mycobacterium tuberculosis	35.1															9.01	2.81	16	7.3
Proteus spp.	57.1	16.1				16.4		17	32.1		54.6								
Pseudomonas aeruginosa	189	66.3	112		114	103	111	128											
Salmonella Paratyphi	0.424					0.423								0.00224					
Salmonella Typhi	2.87					2.64								0.288					
Non−typhoidal Salmonella	0.0565					0.0565													
Serratia spp.	22.1	6.61	7.23		3.42	3.41	16	13.5											
Shigella spp.	0.215					0.215													
Staphylococcus aureus	218					132			14.6	5.78		121			144				
Streptococcus pneumoniae	118			10.9	28.7	5.36		15.7	105			20.6	49.2						
All pathogens	1,980	458	298	746	331	1,200	296	877	778	43.7	488	180	51.7	0.29	144	9.01	2.81	16	7.3

Counts: **< 5k**, **NA**

Austria Deaths (count) associated with bacterial antimicrobial resistance by pathogen−drug combinations, 2019

	Resistance to 1+	AG	Anti−pseudomonal	BL−BLI	CP	FQ	4GC	3GC	TMP−SMX	Vanco	Aminopenicillin	Macrolide	PCN	MDR in S. Typhi and Paratyphi	Methicillin	Mono INH	Mono RIF	MDR excluding XDR in TB	XDR in TB
Acinetobacter baumannii	216	19.6	211	151	20.5	26	211	211											
Citrobacter spp.	12.8	1.02	4.75		2.97	2.57	1.9	10.2											
Enterobacter spp.	107	5.13	91.6		13.2	8.24	50.8		24										
Enterococcus faecalis	35.1					32.1				4.05									
Enterococcus faecium	333					332				24.5									
Other enterococci	14.8					13				3.83									
Escherichia coli	1,090	135		581	2.63	487		242	667		916								
Group A Streptococcus	7.74											7.74							
Group B Streptococcus	70.7					8.21						67.5	1.44						
Haemophilus influenzae	8.8							1.27			8.33								
Klebsiella pneumoniae	250	33.6		167	10.6	119		80.8	171										
Morganella spp.	1.2					0.638	0.128	0.844											
Mycobacterium tuberculosis	6.87															2.87	0.029	3.14	0.827
Proteus spp.	112	2.95				20.4		15.2	38.2		111								
Pseudomonas aeruginosa	183	28.9	97.5		104	83.8	55.4	77											
Salmonella Paratyphi	0.12					0.12								0.000729					
Salmonella Typhi	1.87					1.5								0.435					
Non−typhoidal Salmonella	4.46					4.46													
Serratia spp.	3.52	0.924	1.08		1.11	1	0.854	1.52											
Shigella spp.	0.788					0.788													
Staphylococcus aureus	573					160			34.1	11.8		515			167				
Streptococcus pneumoniae	114			13.2	19.1	18.8		12.1	63.3			51.3	31.8						
All pathogens	3,150	227	406	912	175	1,320	320	652	997	44.2	1,030	642	33.3	0.436	167	2.87	0.029	3.14	0.827

Counts
< 5k
NA

Azerbaijan Deaths (count) associated with bacterial antimicrobial resistance by pathogen−drug combinations, 2019

	Resistance to 1+	AG	Anti−pseudomonal	BL−BLI	CP	FQ	4GC	3GC	TMP−SMX	Vanco	Aminopenicillin	Macrolide	PCN	MDR in S. Typhi and Paratyphi	Methicillin	Mono INH	Mono RIF	MDR excluding XDR in TB	XDR in TB
Acinetobacter baumannii	365	287	360	324	306	326	345	352											
Citrobacter spp.	29.2	5.45	7.32		8.74	15	4.22	19.7											
Enterobacter spp.	174	34	101		29.2	34.2	94.4		95.4										
Enterococcus faecalis	87.7					81.4				11.7									
Enterococcus faecium	299					297				72.4									
Other enterococci	111					111				7									
Escherichia coli	985	287		730	71.7	607		578	681		908								
Group A Streptococcus	17.4											17.4							
Group B Streptococcus	144					74.5						102	12.6						
Haemophilus influenzae	23.1							10.9			16.2								
Klebsiella pneumoniae	843	330		720	70.5	453		688	720										
Morganella spp.	3.43					2.73	0.675	1.5											
Mycobacterium tuberculosis	315															50	35.7	157	72.4
Proteus spp.	106	20.6				38.5		44.9	66.6		96.2								
Pseudomonas aeruginosa	459	144	225		236	273	239	319											
Salmonella Paratyphi	0.0292					0.0291								0.000183					
Salmonella Typhi	14.7					12								3.39					
Non−typhoidal Salmonella	0.273					0.273													
Serratia spp.	50.6	17.6	18.1		9.78	11.7	22.3	34.3											
Shigella spp.	0.741					0.741													
Staphylococcus aureus	815					364			118	10.1		709			361				
Streptococcus pneumoniae	601			124	230	23.7		76	527			268	217						
All pathogens	5,440	1,130	712	1,900	961	2,730	706	2,120	2,210	101	1,020	1,100	230	3.39	361	50	35.7	157	72.4

Counts
- **5k − <10k**
- **< 5k**
- **NA**

Belarus Deaths (count) associated with bacterial antimicrobial resistance by pathogen−drug combinations, 2019

	Resistance to 1+	AG	Anti−pseudomonal	BL−BLI	CP	FQ	4GC	3GC	TMP−SMX	Vanco	Aminopenicillin	Macrolide	PCN	MDR in S. Typhi and Paratyphi	Methicillin	Mono INH	Mono RIF	MDR excluding XDR in TB	XDR in TB
Acinetobacter baumannii	421	352	402	354	282	408	419	393											
Citrobacter spp.	36.2	2.83	18.6		7.91	6.36	2.79	29.3											
Enterobacter spp.	211	20.3	151		28.4	35.4	96.7		95.8										
Enterococcus faecalis	247					244				15.4									
Enterococcus faecium	386					385				85.3									
Other enterococci	111					110				5.69									
Escherichia coli	1,360	534		1,130	51.2	653		984	799		1,020								
Group A Streptococcus	13.8											13.8							
Group B Streptococcus	132					8.38						130	1.05						
Haemophilus influenzae	3.18							1.49			2.06								
Klebsiella pneumoniae	760	553		728	177	496		692	734										
Morganella spp.	2.34					0.831	0.159	2.01											
Mycobacterium tuberculosis	198															10.3	1.49	128	58.7
Proteus spp.	132	9.65				44.5		17.9	57.3		130								
Pseudomonas aeruginosa	489	184	283		370	363	260	254											
Salmonella Paratyphi	0.0128					0.0128								8.18e−05					
Salmonella Typhi	6.04					3.73								3.02					
Non−typhoidal Salmonella	3.65					3.65													
Serratia spp.	25	7.71	5.06		5.34	2.57	8.07	18.9											
Shigella spp.	0.262					0.262													
Staphylococcus aureus	366					84.5			27.8	9.44		243			240				
Streptococcus pneumoniae	352			24.3	38.7	23		25.4	298			112	124						
All pathogens	5,250	1,660	861	2,240	961	2,870	786	2,420	2,010	116	1,160	498	125	3.02	240	10.3	1.49	128	58.7

Counts
5k − <10k
< 5k
NA

Belgium Deaths (count) associated with bacterial antimicrobial resistance by pathogen−drug combinations, 2019

	Resistance to 1+	AG	Anti−pseudomonal	BL−BLI	CP	FQ	4GC	3GC	TMP−SMX	Vanco	Aminopenicillin	Macrolide	PCN	MDR in S. Typhi and Paratyphi	Methicillin	Mono INH	Mono RIF	MDR excluding XDR in TB	XDR in TB
Acinetobacter baumannii	275	27.5	102	266	33	54.2	117	189											
Citrobacter spp.	30.1	1.7	19.6		4.15	8.05	2.17	23.9											
Enterobacter spp.	166	11.5	132		25.8	71.9	48.6		21.9										
Enterococcus faecalis	60.1					49.5				13.7									
Enterococcus faecium	564					563				33.2									
Other enterococci	48.1					43.2				13.1									
Escherichia coli	2,210	247		1,350	51.2	947		446	1,290		1,980								
Group A Streptococcus	12.6											12.6							
Group B Streptococcus	117					22						107	2.43						
Haemophilus influenzae	50.4							4.36			49.4								
Klebsiella pneumoniae	630	139		520	41.6	372		389	296										
Morganella spp.	4.87					2.36	0.344	3.89											
Mycobacterium tuberculosis	7.68															3.83	0.11	2.95	0.782
Proteus spp.	220	6.28				37.7		15	83.1		219								
Pseudomonas aeruginosa	451	106	232		284	207	209	202											
Salmonella Paratyphi	0.0352					0.0353								0.00029					
Salmonella Typhi	2.6					2.15								0.545					
Non−typhoidal Salmonella	15.5					15.5													
Serratia spp.	15.1	1.36	3.69		2.07	3.16	2.71	13.5											
Shigella spp.	4.47					4.47													
Staphylococcus aureus	1,430					711			83.1	22.2		1,100			607				
Streptococcus pneumoniae	438			57.1	198	14		10.2	244			235	17.3						
All pathogens	6,760	540	490	2,190	641	3,130	380	1,300	2,020	82.2	2,250	1,460	19.8	0.545	607	3.83	0.11	2.95	0.782

Counts
5k − <10k
< 5k
NA

Bosnia and Herzegovina Deaths (count) associated with bacterial antimicrobial resistance by pathogen−drug combinations, 2

	Resistance to 1+	AG	Anti−pseudomonal	BL−BLI	CP	FQ	4GC	3GC	TMP−SMX	Vanco	Aminopenicillin	Macrolide	PCN	MDR in S. Typhi and Paratyphi	Methicillin	Mono INH	Mono RIF	MDR excluding XDR in TB	XDR in TB
Acinetobacter baumannii	214	143	209	172	134	200	196	198											
Citrobacter spp.	29.8	1.82	11.7		5.5	21.5	5.3	16											
Enterobacter spp.	96.5	18.9	56.3		10.6	42.1	59.4		50.5										
Enterococcus faecalis	76.5					75.3				4.02									
Enterococcus faecium	141					140				47.3									
Other enterococci	49.5					48.7				11.4									
Escherichia coli	422	120		294	66.3	186		196	228		352								
Group A Streptococcus	9.69											9.69							
Group B Streptococcus	37.5					5.98						35.3	0.931						
Haemophilus influenzae	2.89							0.558			2.61								
Klebsiella pneumoniae	277	157		256	88.4	171		250	111										
Morganella spp.	1.26					0.762	0.243	0.838											
Mycobacterium tuberculosis	2.84															1.21	0.0193	1.11	0.508
Proteus spp.	50.6	10.2				25.3		16.1	25.1		46.1								
Pseudomonas aeruginosa	178	72.5	106		115	108	91.5	110											
Salmonella Paratyphi	0.00191					0.00191								1.11e−05					
Salmonella Typhi	2.19					2								0.229					
Non−typhoidal Salmonella	0.166					0.166													
Serratia spp.	16.3	6.43	4.57		2.89	2.6	8.84	10.5											
Shigella spp.	0.0133					0.0133													
Staphylococcus aureus	230					98.8			11.8	3.14		178			145				
Streptococcus pneumoniae	136			14.4	45.3	7.59		70.5	42.2			71.6	82.8						
All pathogens	1,970	530	387	736	468	1,140	362	868	469	65.9	401	294	83.7	0.229	145	1.21	0.0193	1.11	0.508

Counts
< 5k
NA

Bulgaria Deaths (count) associated with bacterial antimicrobial resistance by pathogen−drug combinations, 2019

	Resistance to 1+	AG	Anti−pseudomonal	BL−BLI	CP	FQ	4GC	3GC	TMP−SMX	Vanco	Aminopenicillin	Macrolide	PCN	MDR in S. Typhi and Paratyphi	Methicillin	Mono INH	Mono RIF	MDR excluding XDR in TB	XDR in TB
Acinetobacter baumannii	367	282	362	354	298	307	363	353											
Citrobacter spp.	36.5	2.24	14.3		8.59	13.4	4.79	29.3											
Enterobacter spp.	233	78	131		14.3	133	188		112										
Enterococcus faecalis	299					296				14.1									
Enterococcus faecium	431					428				113									
Other enterococci	120					118				30.8									
Escherichia coli	2,030	661		1,400	69.9	1,210		1,140	1,230		1,820								
Group A Streptococcus	33.1											33.1							
Group B Streptococcus	92.8					16.3						85.9	2.76						
Haemophilus influenzae	12.9							5.45			9.43								
Klebsiella pneumoniae	1,020	542		892	363	734		975	555										
Morganella spp.	3.92					2.67	0.711	2.29											
Mycobacterium tuberculosis	15.3															6.41	1.01	5.4	2.46
Proteus spp.	212	33.6				54.5		212	86.1		146								
Pseudomonas aeruginosa	497	234	317		251	273	283	267											
Salmonella Paratyphi	0.0143					0.0142								1e−04					
Salmonella Typhi	11.3					9.31								2.5					
Non−typhoidal Salmonella	0.239					0.239													
Serratia spp.	68.8	20.1	13.9		5.52	3.74	60.6	59.2											
Shigella spp.	0.251					0.251													
Staphylococcus aureus	572					183			44.4	14.9		406			333				
Streptococcus pneumoniae	334			81.9	135	49.1		64.2	40			199	177						
All pathogens	6,390	1,850	839	2,730	1,150	3,830	900	3,110	2,060	172	1,980	724	179	2.51	333	6.41	1.01	5.4	2.46

Counts
- 5k − <10k
- < 5k
- NA

Croatia Deaths (count) associated with bacterial antimicrobial resistance by pathogen−drug combinations, 2019

	Resistance to 1+	AG	Anti−pseudomonal	BL−BLI	CP	FQ	4GC	3GC	TMP−SMX	Vanco	Aminopenicillin	Macrolide	PCN	MDR in S. Typhi and Paratyphi	Methicillin	Mono INH	Mono RIF	MDR excluding XDR in TB	XDR in TB
Acinetobacter baumannii	288	253	283	252	277	279	284	283											
Citrobacter spp.	17.9	1.36	7.34		2.36	4.43	2.02	15											
Enterobacter spp.	123	4.47	51.5		13.5	52.9	104		44.6										
Enterococcus faecalis	98.8					96.8				5.53									
Enterococcus faecium	193					191				72.8									
Other enterococci	39					38.6				3.74									
Escherichia coli	694	97.8		407	11.1	324		185	586		543								
Group A Streptococcus	7.66											7.66							
Group B Streptococcus	42.1					5.07						40.5	0.52						
Haemophilus influenzae	1.34							0.71			0.785								
Klebsiella pneumoniae	281	114		237	41.1	217		212	143										
Morganella spp.	2.15					0.3	0.149	2.13											
Mycobacterium tuberculosis	1.32															0.741	0.00575	0.395	0.18
Proteus spp.	63.2	4.32				23.8		13.9	28.7		60.3								
Pseudomonas aeruginosa	210	57.2	87.6		131	125	148	101											
Salmonella Paratyphi	0.00652					0.0065								4.6e−05					
Salmonella Typhi	0.9					0.767								0.16					
Non−typhoidal Salmonella	0.134					0.134													
Serratia spp.	7.95	1.23	1.63		1.05	1.64	3.84	5.69											
Shigella spp.	0.0255					0.0255													
Staphylococcus aureus	335					210			17.4	5.44		209			219				
Streptococcus pneumoniae	139			47	56.3	5.26		10.5	58			94.4	64.4						
All pathogens	2,550	533	431	943	534	1,580	541	829	878	87.5	604	351	65	0.16	219	0.741	0.00575	0.395	0.18

Counts
< 5k
NA

Cyprus Deaths (count) associated with bacterial antimicrobial resistance by pathogen−drug combinations, 2019

	Resistance to 1+	AG	Anti−pseudomonal	BL−BLI	CP	FQ	4GC	3GC	TMP−SMX	Vanco	Aminopenicillin	Macrolide	PCN	MDR in S. Typhi and Paratyphi	Methicillin	Mono INH	Mono RIF	MDR excluding XDR in TB	XDR in TB
Acinetobacter baumannii	21.8	15.9	13.5	17.7	19.5	20.2	17.2	19.2											
Citrobacter spp.	2.35	0.138	0.721		0.258	0.647	0.483	2.02											
Enterobacter spp.	9.91	0.662	6.94		2.86	3.95	3.11		2.41										
Enterococcus faecalis	16.7					16.1				1.18									
Enterococcus faecium	41.5					41				27.6									
Other enterococci	8.3					7.98				1.87									
Escherichia coli	190	42.3		82	5.14	118		95.9	94.8		169								
Group A Streptococcus	2.28											2.28							
Group B Streptococcus	9.37					0.956						9.02	0.275						
Haemophilus influenzae	1.48							0.548			1.21								
Klebsiella pneumoniae	60.9	29.9		43.7	26.3	50.3		44.5	21										
Morganella spp.	0.349					0.28	0.044	0.146											
Mycobacterium tuberculosis	0.938															0.705	0.000268	0.184	0.0485
Proteus spp.	12.8	0.95				6.77		2.57	5.99		10.9								
Pseudomonas aeruginosa	30.6	2.7	16.5		16.8	16.7	10.6	13.4											
Salmonella Paratyphi	0.00063					0.00063								4.93e−06					
Salmonella Typhi	0.252					0.217								0.0434					
Non−typhoidal Salmonella	3.9e−05					3.9e−05													
Serratia spp.	1.08	0.137	0.204		0.283	0.17	0.37	0.839											
Shigella spp.	0.0661					0.0661													
Staphylococcus aureus	111					66.3			9.55	1.53		74.3			80.9				
Streptococcus pneumoniae	24.9			3.45	14.4	1.46		5.87	11.5			7.74	15.6						
All pathogens	547	92.7	37.9	147	85.5	352	31.8	185	145	32.2	181	93.3	15.9	0.0435	80.9	0.705	0.000268	0.184	0.0485

Counts
< 5k
NA

Czechia Deaths (count) associated with bacterial antimicrobial resistance by pathogen−drug combinations, 2019

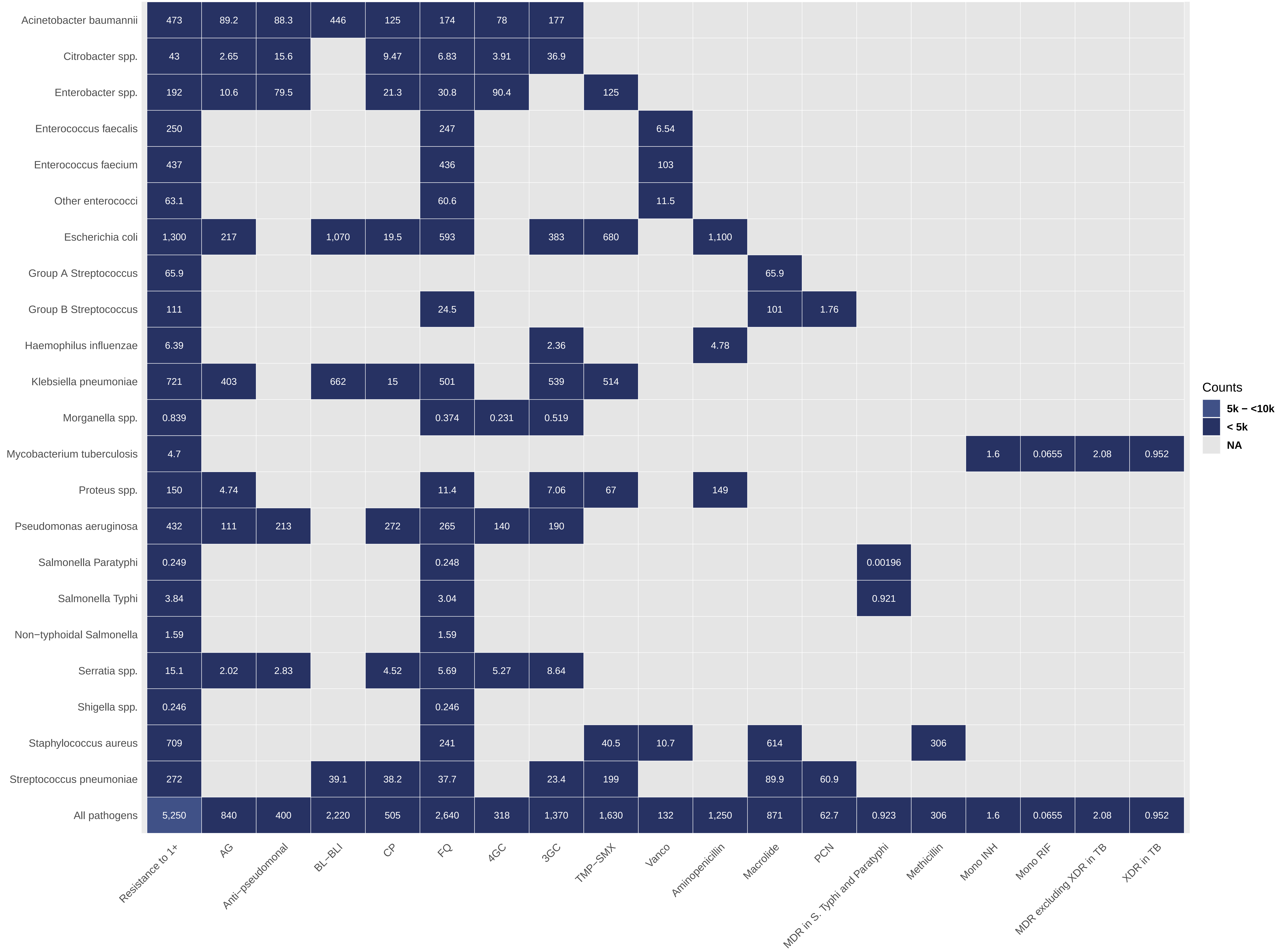

Denmark Deaths (count) associated with bacterial antimicrobial resistance by pathogen−drug combinations, 2019

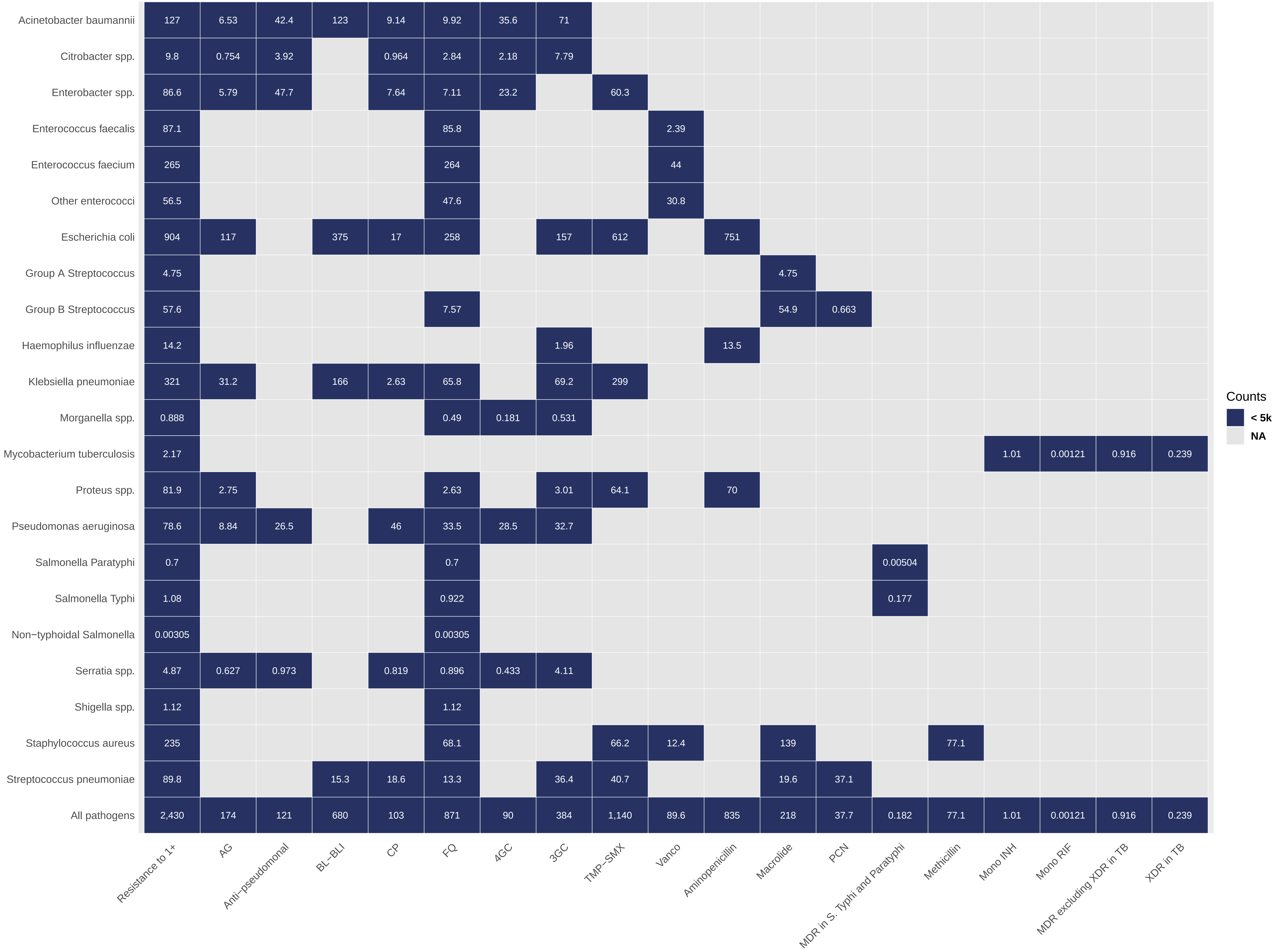

Estonia Deaths (count) associated with bacterial antimicrobial resistance by pathogen−drug combinations, 2019

	Resistance to 1+	AG	Anti−pseudomonal	BL−BLI	CP	FQ	4GC	3GC	TMP−SMX	Vanco	Aminopenicillin	Macrolide	PCN	MDR in S. Typhi and Paratyphi	Methicillin	Mono INH	Mono RIF	MDR excluding XDR in TB	XDR in TB
Acinetobacter baumannii	79.4	21.6	66.5	65.3	34.1	38.4	70.1	74											
Citrobacter spp.	6.2	0.372	2.15		1.4	0.524	0.664	5.55											
Enterobacter spp.	24	1.18	15.4		4.75	3.02	11		10.6										
Enterococcus faecalis	31.2					30.6				1.88									
Enterococcus faecium	54.6					54.5				4.01									
Other enterococci	9.16					9.04				0.905									
Escherichia coli	142	18.4		50.2	0.812	52.3		30.8	106		109								
Group A Streptococcus	2.71											2.71							
Group B Streptococcus	14.2					1.47						13.8	0.273						
Haemophilus influenzae	0.541							0.165			0.431								
Klebsiella pneumoniae	81.3	18.7		42.4	1.72	35.3		31.9	78.7										
Morganella spp.	0.271					0.0664	0.0174	0.25											
Mycobacterium tuberculosis	11.2															1.84	0.155	6.29	2.89
Proteus spp.	18	1.11				2.06		1.46	7.75		17.7								
Pseudomonas aeruginosa	29.6	4.45	14		21.3	12.2	8.61	9.19											
Salmonella Paratyphi	0.0212					0.0211								0.000167					
Salmonella Typhi	0.302					0.253								0.0553					
Non−typhoidal Salmonella	3e−57					3e−57													
Serratia spp.	3.46	0.912	0.765		0.784	0.443	1.18	2.69											
Shigella spp.	0.0653					0.0653													
Staphylococcus aureus	49.6					10.3			4.4	1.53		42.6			13.5				
Streptococcus pneumoniae	34			2.17	7.73	1.72		2.52	31			6.78	2.2						
All pathogens	592	66.7	98.8	160	72.6	252	91.5	158	238	8.32	127	65.9	2.47	0.0555	13.5	1.84	0.155	6.29	2.89

Counts
< 5k
NA

Finland Deaths (count) associated with bacterial antimicrobial resistance by pathogen−drug combinations, 2019

	Resistance to 1+	AG	Anti−pseudomonal	BL−BLI	CP	FQ	4GC	3GC	TMP−SMX	Vanco	Aminopenicillin	Macrolide	PCN	MDR in S. Typhi and Paratyphi	Methicillin	Mono INH	Mono RIF	MDR excluding XDR in TB	XDR in TB
Acinetobacter baumannii	110	8.35	48.7	83.3	7.16	5.12	80.2	95.1											
Citrobacter spp.	8.51	0.641	2.66		2.22	1.45	1.28	6.84											
Enterobacter spp.	54.4	3.22	38.5		13.6	6.75	20.1		19.2										
Enterococcus faecalis	49.9					45.3				7.42									
Enterococcus faecium	236					235				6.33									
Other enterococci	9.74					6.6				5.06									
Escherichia coli	541	107		240	8	187		123	288		439								
Group A Streptococcus	9.76											9.76							
Group B Streptococcus	44.5					6.28						42	2.05						
Haemophilus influenzae	5.89							1.69			5.03								
Klebsiella pneumoniae	175	23.6		120	6.76	63.7		36.9	129										
Morganella spp.	0.762					0.442	0.0839	0.495											
Mycobacterium tuberculosis	6.57															2	0.0063	3.62	0.949
Proteus spp.	74.3	3.91				5.68		10.4	27.9		73.4								
Pseudomonas aeruginosa	95.4	6.1	33.9		49.1	54.6	47.6	30.7											
Salmonella Paratyphi	0.0662					0.0662								0.000518					
Salmonella Typhi	0.767					0.636								0.147					
Non−typhoidal Salmonella	0.0162					0.0162													
Serratia spp.	7.78	0.658	1.62		1.95	1.13	4.68	5.17											
Shigella spp.	0.175					0.175													
Staphylococcus aureus	119					39.4			20.9	7.87		59.1			67.2				
Streptococcus pneumoniae	87.5			6.54	20.6	6.43		13.9	47.1			41.9	39						
All pathogens	1,640	153	125	449	109	667	154	324	532	26.7	518	153	41.1	0.148	67.2	2	0.0063	3.62	0.949

Counts
< 5k
NA

France Deaths (count) associated with bacterial antimicrobial resistance by pathogen−drug combinations, 2019

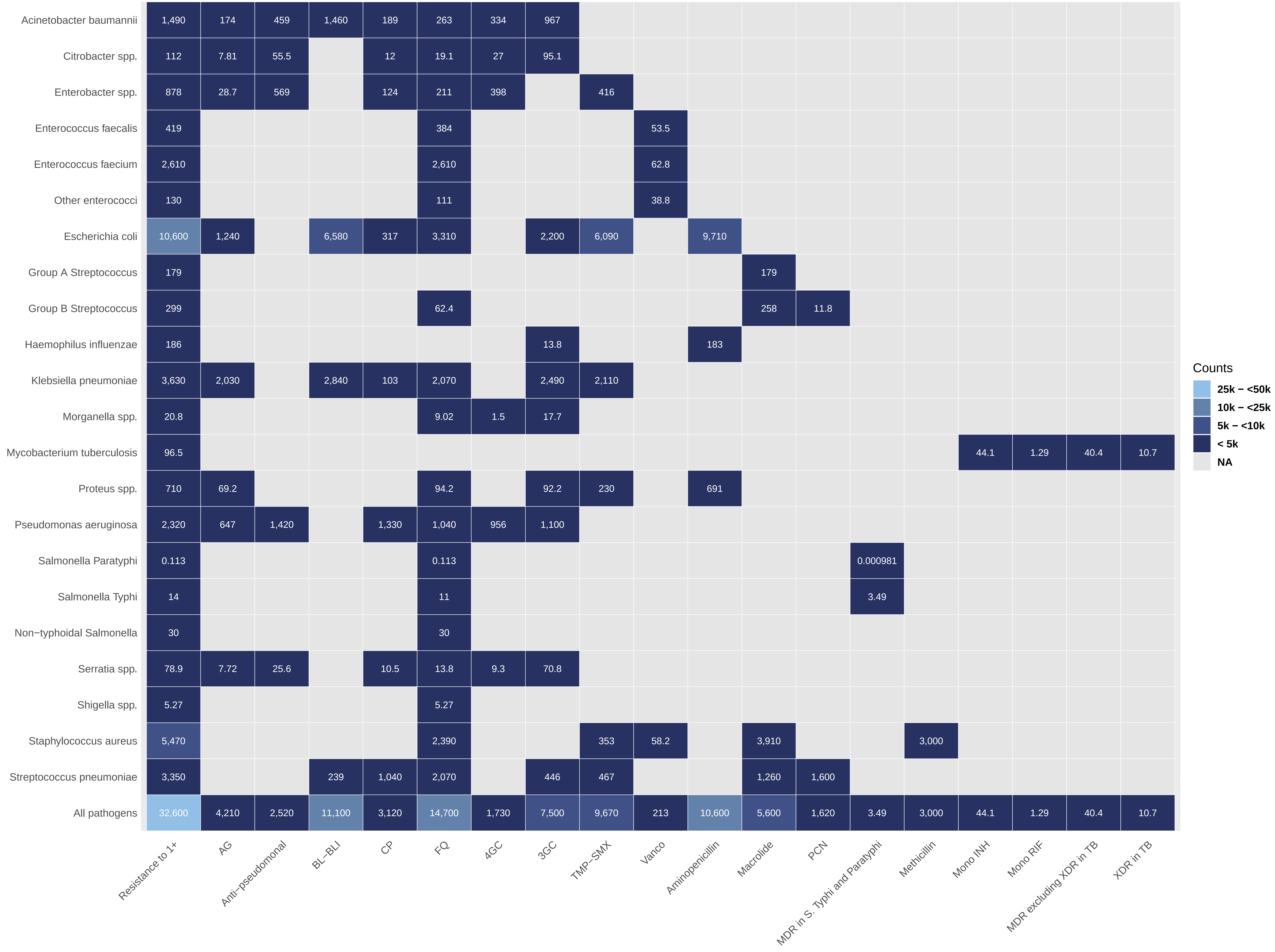

Georgia Deaths (count) associated with bacterial antimicrobial resistance by pathogen−drug combinations, 2019

	Resistance to 1+	AG	Anti−pseudomonal	BL−BLI	CP	FQ	4GC	3GC	TMP−SMX	Vanco	Aminopenicillin	Macrolide	PCN	MDR in S. Typhi and Paratyphi	Methicillin	Mono INH	Mono RIF	MDR excluding XDR in TB	XDR in TB
Acinetobacter baumannii	287	168	281	248	198	261	279	276											
Citrobacter spp.	15.2	2.25	3.72		3.86	4.9	3.47	12.2											
Enterobacter spp.	110	22.2	68.7		14.6	28.9	70		51										
Enterococcus faecalis	77.6					75.8				5.39									
Enterococcus faecium	174					173				32.1									
Other enterococci	68					67.9				7.18									
Escherichia coli	542	140		369	222	313		335	386		446								
Group A Streptococcus	8.51											8.51							
Group B Streptococcus	47.3					6.82						44.7	1.52						
Haemophilus influenzae	4.82							1.24			4.12								
Klebsiella pneumoniae	466	186		371	213	274		462	201										
Morganella spp.	1.71					1.31	0.266	0.869											
Mycobacterium tuberculosis	82.5															16	2.99	43.6	19.9
Proteus spp.	60.8	16.2				25		16.4	32.7		57.7								
Pseudomonas aeruginosa	263	81.6	154		153	156	148	158											
Salmonella Paratyphi	0.535					0.533								0.00436					
Salmonella Typhi	4.82					4.42								0.47					
Non−typhoidal Salmonella	0.196					0.196													
Serratia spp.	40.1	14	11.5		5.4	5.72	31	23.9											
Shigella spp.	0.258					0.258													
Staphylococcus aureus	319					138			18	6.33		236			188				
Streptococcus pneumoniae	202			20.2	88.1	7.09		28	168			49.1	89.5						
All pathogens	2,780	630	519	1,010	897	1,540	532	1,310	857	51	508	338	91	0.475	188	16	2.99	43.6	19.9

Counts
- < 5k
- NA

Germany Deaths (count) associated with bacterial antimicrobial resistance by pathogen−drug combinations, 2019

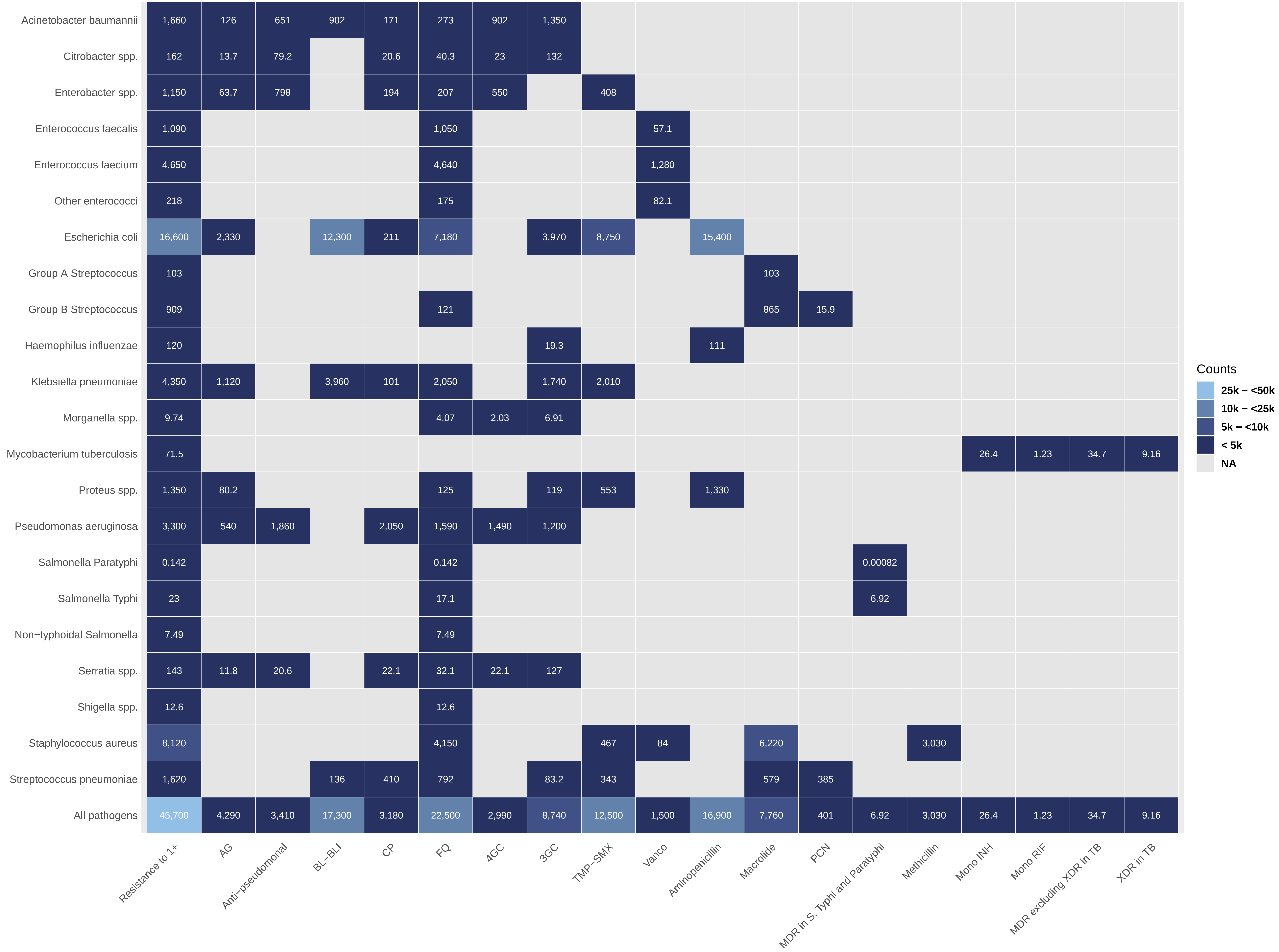

Greece Deaths (count) associated with bacterial antimicrobial resistance by pathogen−drug combinations, 2019

	Resistance to 1+	AG	Anti−pseudomonal	BL−BLI	CP	FQ	4GC	3GC	TMP−SMX	Vanco	Aminopenicillin	Macrolide	PCN	MDR in S. Typhi and Paratyphi	Methicillin	Mono INH	Mono RIF	MDR excluding XDR in TB	XDR in TB
Acinetobacter baumannii	292	256	286	286	282	284	286	287											
Citrobacter spp.	14.3	1.27	6.32		4.64	6.82	1.51	6.02											
Enterobacter spp.	153	7.47	91.6		31.4	71.4	55.7		73.8										
Enterococcus faecalis	219					205				35.7									
Enterococcus faecium	487					486				199									
Other enterococci	83.2					74				33.1									
Escherichia coli	1,960	268		833	75.3	1,040		720	1,010		1,730								
Group A Streptococcus	36.8											36.8							
Group B Streptococcus	41.2					9.32						34.3	1.62						
Haemophilus influenzae	44							22.5			31.5								
Klebsiella pneumoniae	1,040	585		890	702	868		925	641										
Morganella spp.	1.89					1.67	0.248	0.438											
Mycobacterium tuberculosis	17.3															7.45	0.0323	7.78	2.04
Proteus spp.	157	11.3				64.4		23.1	51.1		154								
Pseudomonas aeruginosa	762	311	442		553	465	403	435											
Salmonella Paratyphi	0.0166					0.0166								0.000219					
Salmonella Typhi	3.04					2.66								0.458					
Non−typhoidal Salmonella	0.000504					0.000504													
Serratia spp.	8.12	1.41	2.82		4.11	1.77	1.75	2.82											
Shigella spp.	0.121					0.121													
Staphylococcus aureus	2,030					1,170			91.9	26.8		1,490			1,410				
Streptococcus pneumoniae	479			30.6	54	28.8		42.5	257			215	283						
All pathogens	7,830	1,440	828	2,040	1,710	4,770	748	2,460	2,120	294	1,920	1,770	285	0.458	1,410	7.45	0.0323	7.78	2.04

Counts
- 5k − <10k
- < 5k
- NA

Hungary Deaths (count) associated with bacterial antimicrobial resistance by pathogen−drug combinations, 2019

	Resistance to 1+	AG	Anti−pseudomonal	BL−BLI	CP	FQ	4GC	3GC	TMP−SMX	Vanco	Aminopenicillin	Macrolide	PCN	MDR in S. Typhi and Paratyphi	Methicillin	Mono INH	Mono RIF	MDR excluding XDR in TB	XDR in TB
Acinetobacter baumannii	281	134	248	223	188	209	268	250											
Citrobacter spp.	24.2	1.66	12.2		3.79	4.77	3.15	20.7											
Enterobacter spp.	156	7.1	89.9		25.8	37.9	66.9		88.6										
Enterococcus faecalis	275					272				10.4									
Enterococcus faecium	611					609				277									
Other enterococci	94					93.6				5.61									
Escherichia coli	2,100	524		1,100	49.2	1,120		799	1,210		1,840								
Group A Streptococcus	12.7											12.7							
Group B Streptococcus	135					12.7						132	1.75						
Haemophilus influenzae	4.82							1.77			3.62								
Klebsiella pneumoniae	776	414		582	14	528		583	506										
Morganella spp.	2.93					2.04	0.207	1.72											
Mycobacterium tuberculosis	5.66															3.32	0.0395	1.58	0.716
Proteus spp.	161	5.92				71.9		44	73.9		151								
Pseudomonas aeruginosa	503	113	276		373	222	197	259											
Salmonella Paratyphi	0.018					0.0179								0.000122					
Salmonella Typhi	6.8					5.45								1.59					
Non−typhoidal Salmonella	0.000693					0.000693													
Serratia spp.	21.8	6.82	5.24		8.8	7.2	6.94	9.09											
Shigella spp.	0.211					0.211													
Staphylococcus aureus	1,090					548			48.5	16.8		811			688				
Streptococcus pneumoniae	262			22.3	90.1	63.7		34.4	144			96.3	88.9						
All pathogens	6,520	1,210	632	1,930	752	3,810	542	2,000	2,070	310	1,990	1,050	90.7	1.59	688	3.32	0.0395	1.58	0.716

Counts
- 5k − <10k
- < 5k
- NA

Iceland Deaths (count) associated with bacterial antimicrobial resistance by pathogen−drug combinations, 2019

	Resistance to 1+	AG	Anti−pseudomonal	BL−BLI	CP	FQ	4GC	3GC	TMP−SMX	Vanco	Aminopenicillin	Macrolide	PCN	MDR in S. Typhi and Paratyphi	Methicillin	Mono INH	Mono RIF	MDR excluding XDR in TB	XDR in TB
Acinetobacter baumannii	4.45	0.542	2.69	3.91	0.773	0.641	1.93	3.86											
Citrobacter spp.	0.239	0.0208	0.121		0.0369	0.0553	0.0346	0.186											
Enterobacter spp.	2.29	0.15	1.77		0.393	0.211	0.735		0.819										
Enterococcus faecalis	1.31					1.21				0.155									
Enterococcus faecium	7.77					7.76				0.601									
Other enterococci	0.454					0.404				0.122									
Escherichia coli	27.2	2.32		18.7	1.13	10.4		6.6	15.2		23.9								
Group A Streptococcus	0.317											0.317							
Group B Streptococcus	2.61					1.21						1.98	0.0459						
Haemophilus influenzae	0.443							0.0477			0.423								
Klebsiella pneumoniae	10.3	2.28		9.3	0.28	2.47		3.4	5.92										
Morganella spp.	0.039					0.0242	0.00352	0.0248											
Mycobacterium tuberculosis	0.161															0.13	0.000204	0.0242	0.00636
Proteus spp.	2.61	0.244				0.447		0.496	1.27		2.57								
Pseudomonas aeruginosa	5.15	0.634	2.48		3.1	2.48	2.25	1.44											
Salmonella Paratyphi	0.00499					0.00499								4.56e−05					
Salmonella Typhi	0.0318					0.0283								0.004					
Non−typhoidal Salmonella	0.0222					0.0222													
Serratia spp.	0.192	0.0296	0.05		0.056	0.0381	0.0736	0.135											
Shigella spp.	0.0148					0.0148													
Staphylococcus aureus	13.3					7.58			1.03	0.321		7.76			6.47				
Streptococcus pneumoniae	5.94			0.534	2.34	0.399		0.55	3.77			2.26	1.59						
All pathogens	84.9	6.22	7.11	32.4	8.11	35.4	5.02	16.7	28	1.2	26.9	12.3	1.64	0.00404	6.47	0.13	0.000204	0.0242	0.00636

Counts

< 5k

NA

Ireland Deaths (count) associated with bacterial antimicrobial resistance by pathogen−drug combinations, 2019

	Resistance to 1+	AG	Anti−pseudomonal	BL−BLI	CP	FQ	4GC	3GC	TMP−SMX	Vanco	Aminopenicillin	Macrolide	PCN	MDR in S. Typhi and Paratyphi	Methicillin	Mono INH	Mono RIF	MDR excluding XDR in TB	XDR in TB
Acinetobacter baumannii	71.9	2.51	44.2	66.9	4.73	4.08	44.8	55.2											
Citrobacter spp.	4	0.382	2.04		0.447	0.784	0.716	3.2											
Enterobacter spp.	56.7	2.19	36.4		3.73	14.3	38.4		24.6										
Enterococcus faecalis	41.3					39.8				2.96									
Enterococcus faecium	140					139				74.5									
Other enterococci	5.21					4.12				1.95									
Escherichia coli	554	89		417	11.5	233		135	276		537								
Group A Streptococcus	7.43											7.43							
Group B Streptococcus	30.4					5.22						28.1	0.65						
Haemophilus influenzae	7.5							1.17			7.02								
Klebsiella pneumoniae	189	34.7		126	4.72	77.2		69.6	162										
Morganella spp.	0.605					0.324	0.0683	0.417											
Mycobacterium tuberculosis	2.24															1.16	0.0167	0.846	0.223
Proteus spp.	43.9	3.45				3.25		2.39	13.2		43.4								
Pseudomonas aeruginosa	76.3	19.3	39.7		40.6	36.8	34.6	29.9											
Salmonella Paratyphi	0.00224					0.00224								2.06e−05					
Salmonella Typhi	0.537					0.465								0.0825					
Non−typhoidal Salmonella	0.00416					0.00416													
Serratia spp.	2.45	0.323	0.757		0.477	0.42	0.326	2.02											
Shigella spp.	0.279					0.279													
Staphylococcus aureus	362					155			21.8	7.45		295			146				
Streptococcus pneumoniae	171			11.1	93.5	17.5		20.7	110			52.1	76.3						
All pathogens	1,770	152	123	621	160	731	119	319	607	86.9	587	383	76.9	0.0825	146	1.16	0.0167	0.846	0.223

Counts
< 5k
NA

Israel Deaths (count) associated with bacterial antimicrobial resistance by pathogen−drug combinations, 2019

	Resistance to 1+	AG	Anti−pseudomonal	BL−BLI	CP	FQ	4GC	3GC	TMP−SMX	Vanco	Aminopenicillin	Macrolide	PCN	MDR in S. Typhi and Paratyphi	Methicillin	Mono INH	Mono RIF	MDR excluding XDR in TB	XDR in TB
Acinetobacter baumannii	152	96.3	143	123	106	130	124	138											
Citrobacter spp.	14.3	0.83	3.59		1.65	3.5	1.3	12.8											
Enterobacter spp.	66.6	4.05	36.7		26.9	14.6	25.1		26.5										
Enterococcus faecalis	123					119				9.06									
Enterococcus faecium	222					221				64.5									
Other enterococci	38.2					34.6				10.8									
Escherichia coli	1,230	71.6		890	46.6	623		475	879		1,090								
Group A Streptococcus	17.5											17.5							
Group B Streptococcus	60.3					14.2						52.9	0.995						
Haemophilus influenzae	11.4							1.47			11								
Klebsiella pneumoniae	398	98.9		325	29.8	187		306	243										
Morganella spp.	2.47					1.95	0.249	1.1											
Mycobacterium tuberculosis	5.46															1.84	0.061	2.82	0.743
Proteus spp.	117	3.38				49.1		30.6	64.6		115								
Pseudomonas aeruginosa	229	16.3	109		161	122	42.2	64.4											
Salmonella Paratyphi	0.00564					0.00565								6.09e−05					
Salmonella Typhi	1.77					1.45								0.361					
Non−typhoidal Salmonella	0.123					0.123													
Serratia spp.	5.84	0.732	1.02		1.03	1.03	0.671	5.03											
Shigella spp.	0.857					0.857													
Staphylococcus aureus	881					621			30.6	9.79		619			636				
Streptococcus pneumoniae	202			43	79.2	15.4		28.9	113			109	88.2						
All pathogens	3,780	292	293	1,380	452	2,160	194	1,060	1,360	94.1	1,210	799	89.2	0.361	636	1.84	0.061	2.82	0.743

Counts
< 5k
NA

Italy Deaths (count) associated with bacterial antimicrobial resistance by pathogen−drug combinations, 2019

	Resistance to 1+	AG	Anti−pseudomonal	BL−BLI	CP	FQ	4GC	3GC	TMP−SMX	Vanco	Aminopenicillin	Macrolide	PCN	MDR in S. Typhi and Paratyphi	Methicillin	Mono INH	Mono RIF	MDR excluding XDR in TB	XDR in TB
Acinetobacter baumannii	1,380	1,110	1,280	1,280	1,220	1,170	1,280	1,300											
Citrobacter spp.	122	7.13	55.1		13.5	28.2	24.4	106											
Enterobacter spp.	682	66.9	445		207	170	214		291										
Enterococcus faecalis	1,370					1,340				89.5									
Enterococcus faecium	2,760					2,740				766									
Other enterococci	341					337				35.6									
Escherichia coli	11,300	2,980		6,870	194	7,220		5,160	6,040		10,100								
Group A Streptococcus	106											106							
Group B Streptococcus	675					69.7						653	10.3						
Haemophilus influenzae	74.7							10.1			71.1								
Klebsiella pneumoniae	4,240	2,350		3,610	1,840	3,240		3,360	2,680										
Morganella spp.	18.5					16.6	0.888	7.5											
Mycobacterium tuberculosis	56.9															26.2	2.41	22.5	5.91
Proteus spp.	912	157				570		473	693		820								
Pseudomonas aeruginosa	2,480	578	1,270		1,610	1,430	1,020	1,160											
Salmonella Paratyphi	0.0926					0.0927								0.000691					
Salmonella Typhi	11.8					10.3								1.75					
Non−typhoidal Salmonella	0.0417					0.0417													
Serratia spp.	42.5	12.2	11.2		10.3	12.1	10.5	24.6											
Shigella spp.	4.56					4.56													
Staphylococcus aureus	7,970					5,350			246	80.1		5,530			5,080				
Streptococcus pneumoniae	1,240			98.5	428	171		147	291			884	441						
All pathogens	35,800	7,270	3,070	11,900	5,520	23,900	2,540	11,700	10,200	971	11,000	7,180	451	1.75	5,080	26.2	2.41	22.5	5.91

Counts
- **25k − <50k**
- **10k − <25k**
- **5k − <10k**
- **< 5k**
- **NA**

Kazakhstan Deaths (count) associated with bacterial antimicrobial resistance by pathogen−drug combinations, 2019

	Resistance to 1+	AG	Anti−pseudomonal	BL−BLI	CP	FQ	4GC	3GC	TMP−SMX	Vanco	Aminopenicillin	Macrolide	PCN	MDR in S. Typhi and Paratyphi	Methicillin	Mono INH	Mono RIF	MDR excluding XDR in TB	XDR in TB
Acinetobacter baumannii	738	597	724	624	591	672	676	705											
Citrobacter spp.	44.6	4.79	10.1		13.5	13.4	9.93	34.2											
Enterobacter spp.	342	60.4	222		41.9	90.2	203		161										
Enterococcus faecalis	280					274				18.9									
Enterococcus faecium	629					626				129									
Other enterococci	195					195				25.9									
Escherichia coli	1,770	479		1,250	225	415		310	1,250		1,640								
Group A Streptococcus	26.6											26.6							
Group B Streptococcus	180					35						165	7.21						
Haemophilus influenzae	18							4.47			15.4								
Klebsiella pneumoniae	1,500	638		1,300	107	1,040		1,210	1,230										
Morganella spp.	5.28					3.71	0.882	3.04											
Mycobacterium tuberculosis	456															107	24.2	223	102
Proteus spp.	189	46.3				69.9		50.7	108		180								
Pseudomonas aeruginosa	969	317	607		590	602	526	594											
Salmonella Paratyphi	1.25					1.24								0.0111					
Salmonella Typhi	18.1					14.6								4.3					
Non−typhoidal Salmonella	0.48					0.48													
Serratia spp.	113	39.2	32.3		16.7	16.6	78.1	72.6											
Shigella spp.	1.13					1.13													
Staphylococcus aureus	1,230					696			76.7	23.6		738			816				
Streptococcus pneumoniae	792			74.8	262	32.8		115	708			160	264						
All pathogens	9,500	2,180	1,600	3,250	1,850	4,800	1,490	3,100	3,530	198	1,840	1,090	271	4.31	816	107	24.2	223	102

Counts
- 5k − <10k
- < 5k
- NA

Kyrgyzstan Deaths (count) associated with bacterial antimicrobial resistance by pathogen−drug combinations, 2019

	Resistance to 1+	AG	Anti−pseudomonal	BL−BLI	CP	FQ	4GC	3GC	TMP−SMX	Vanco	Aminopenicillin	Macrolide	PCN	MDR in S. Typhi and Paratyphi	Methicillin	Mono INH	Mono RIF	MDR excluding XDR in TB	XDR in TB
Acinetobacter baumannii	171	134	169	150	130	157	156	164											
Citrobacter spp.	14.2	2.72	2.48		3.96	7.25	4.5	9.5											
Enterobacter spp.	89.2	15.7	57.9		12.6	14.7	50		48.8										
Enterococcus faecalis	71.6					69.4				5.77									
Enterococcus faecium	145					144				26.5									
Other enterococci	48.3					48.3				2.28									
Escherichia coli	482	104		363	62.2	280		282	333		442								
Group A Streptococcus	5.86											5.86							
Group B Streptococcus	51					14.5						44.4	2.76						
Haemophilus influenzae	5.27							2.65			3.35								
Klebsiella pneumoniae	393	176		328	49	205		328	351										
Morganella spp.	1.66					1.25	0.449	0.766											
Mycobacterium tuberculosis	291															67.9	34	130	59.5
Proteus spp.	51.4	12.2				15.2		18	31.7		48.7								
Pseudomonas aeruginosa	232	94.3	138		125	150	124	155											
Salmonella Paratyphi	0.0422					0.042								0.000407					
Salmonella Typhi	7.93					7.08								1.06					
Non−typhoidal Salmonella	0.169					0.169													
Serratia spp.	27.8	8.51	9.71		5.71	5.17	17.8	18.6											
Shigella spp.	1.75					1.75													
Staphylococcus aureus	359					179			62.4	4.92		216			286				
Streptococcus pneumoniae	227			24.5	59.2	8.64		28.2	210			36.3	87.7						
All pathogens	2,680	548	377	866	447	1,310	353	1,010	1,040	39.5	494	303	90.5	1.06	286	67.9	34	130	59.5

Counts
< 5k
NA

Latvia Deaths (count) associated with bacterial antimicrobial resistance by pathogen−drug combinations, 2019

	Resistance to 1+	AG	Anti−pseudomonal	BL−BLI	CP	FQ	4GC	3GC	TMP−SMX	Vanco	Aminopenicillin	Macrolide	PCN	MDR in S. Typhi and Paratyphi	Methicillin	Mono INH	Mono RIF	MDR excluding XDR in TB	XDR in TB
Acinetobacter baumannii	71.8	46.7	69.6	62.9	60	63.3	58.6	69.1											
Citrobacter spp.	4.84	0.297	2.42		1.69	1.07	0.743	3.38											
Enterobacter spp.	23.4	1.71	8.32		3.61	7.9	7.65		16.6										
Enterococcus faecalis	69.4					67.1				10.8									
Enterococcus faecium	99.9					99.5				43.6									
Other enterococci	20.1					19.9				1.55									
Escherichia coli	377	49.7		178	14.5	173		151	221		323								
Group A Streptococcus	4.87											4.87							
Group B Streptococcus	28.5					2.27						28	0.322						
Haemophilus influenzae	0.674							0.367			0.384								
Klebsiella pneumoniae	173	65.5		134	4.83	108		114	136										
Morganella spp.	0.847					0.196	0.0832	0.801											
Mycobacterium tuberculosis	20.3															7.78	0.0218	8.59	3.92
Proteus spp.	37	3.66				14.6		8.21	16		36								
Pseudomonas aeruginosa	112	38.6	61.4		55.4	70.9	64.8	70.1											
Salmonella Paratyphi	0.00239					0.00238								1.72e−05					
Salmonella Typhi	1.48					1.2								0.31					
Non−typhoidal Salmonella	0.197					0.197													
Serratia spp.	4.97	1.68	1.09		1.72	0.494	1.45	3.25											
Shigella spp.	0.0469					0.0469													
Staphylococcus aureus	115					21.3			10.3	3.68		98			37.2				
Streptococcus pneumoniae	62.3			6.48	18.6	8.42		4.35	50.4			13.4	16						
All pathogens	1,230	208	143	381	160	659	133	424	450	59.6	360	144	16.3	0.31	37.2	7.78	0.0218	8.59	3.92

Counts: **< 5k** **NA**

Lithuania Deaths (count) associated with bacterial antimicrobial resistance by pathogen−drug combinations, 2019

	Resistance to 1+	AG	Anti−pseudomonal	BL−BLI	CP	FQ	4GC	3GC	TMP−SMX	Vanco	Aminopenicillin	Macrolide	PCN	MDR in S. Typhi and Paratyphi	Methicillin	Mono INH	Mono RIF	MDR excluding XDR in TB	XDR in TB
Acinetobacter baumannii	98.9	83	93.6	90.9	91.4	92.8	93.9	94.4											
Citrobacter spp.	8.6	0.466	3.63		0.918	2.1	0.658	7.69											
Enterobacter spp.	47.4	2.61	32		5.74	5.76	20.3		23.4										
Enterococcus faecalis	13					8.36				5.54									
Enterococcus faecium	138					135				67.9									
Other enterococci	27					26.8				2.23									
Escherichia coli	586	72.6		259	22.3	217		174	466		468								
Group A Streptococcus	16											16							
Group B Streptococcus	38.3					3.17						37.6	0.272						
Haemophilus influenzae	1.01							0.785			0.331								
Klebsiella pneumoniae	327	119		232	10.4	217		229	322										
Morganella spp.	1.21					0.0873	0.111	1.2											
Mycobacterium tuberculosis	71.1															13.3	2.39	38	17.4
Proteus spp.	52	3.85				22.9		10.8	22.3		50.2								
Pseudomonas aeruginosa	134	29	65.5		70.9	64.1	102	57.9											
Salmonella Paratyphi	0.0115					0.0114								9.34e−05					
Salmonella Typhi	2.46					1.93								0.612					
Non−typhoidal Salmonella	0.000254					0.000254													
Serratia spp.	9.82	0.647	0.875		1.09	0.697	5.56	7.98											
Shigella spp.	0.053					0.053													
Staphylococcus aureus	181					21.6			14.7	4.65		156			63.9				
Streptococcus pneumoniae	112			12.8	25.4	10.3		14.4	83.9			48.9	42.3						
All pathogens	1,860	311	196	594	228	830	222	598	933	80.4	518	259	42.6	0.612	63.9	13.3	2.39	38	17.4

Counts
< 5k
NA

Luxembourg Deaths (count) associated with bacterial antimicrobial resistance by pathogen−drug combinations, 2019

	Resistance to 1+	AG	Anti−pseudomonal	BL−BLI	CP	FQ	4GC	3GC	TMP−SMX	Vanco	Aminopenicillin	Macrolide	PCN	MDR in S. Typhi and Paratyphi	Methicillin	Mono INH	Mono RIF	MDR excluding XDR in TB	XDR in TB
Acinetobacter baumannii	9.45	0.296	6.71	8.34	0.308	1.24	6.04	8.36											
Citrobacter spp.	0.707	0.0513	0.369		0.0823	0.142	0.103	0.593											
Enterobacter spp.	4.35	0.253	3.4		0.88	0.88	1.42		1.21										
Enterococcus faecalis	4.47					4.07				0.648									
Enterococcus faecium	21.3					21.3				0.867									
Other enterococci	1.32					1.25				0.254									
Escherichia coli	69.8	8.89		44.2	1.86	31.5		18	36.1		63.1								
Group A Streptococcus	0.847											0.847							
Group B Streptococcus	4.52					1.61						3.68	0.0941						
Haemophilus influenzae	0.776							0.115			0.734								
Klebsiella pneumoniae	21.2	7.64		17.7	0.778	13		15.2	7.04										
Morganella spp.	0.0552					0.0389	0.00528	0.0294											
Mycobacterium tuberculosis	0.201															0.0864	0.00019	0.091	0.024
Proteus spp.	5.93	0.598				1.63		1.05	2.44		5.82								
Pseudomonas aeruginosa	13.3	2.24	6.68		5.54	8.52	4.16	5.58											
Salmonella Paratyphi	0.00883					0.00883								6.93e−05					
Salmonella Typhi	0.155					0.143								0.0177					
Non−typhoidal Salmonella	0.113					0.113													
Serratia spp.	0.49	0.0547	0.107		0.111	0.094	0.0896	0.406											
Shigella spp.	0.0568					0.0568													
Staphylococcus aureus	39.5					16.6			2.02	0.866		31.8			16.4				
Streptococcus pneumoniae	9.19			2.01	3.29	0.826		0.864	5.12			3.74	3.59						
All pathogens	208	20	17.3	72.2	12.9	103	11.8	50.2	53.9	2.63	69.7	40.1	3.68	0.0178	16.4	0.0864	0.00019	0.091	0.024

Counts
< 5k
NA

Malta Deaths (count) associated with bacterial antimicrobial resistance by pathogen−drug combinations, 2019

	Resistance to 1+	AG	Anti−pseudomonal	BL−BLI	CP	FQ	4GC	3GC	TMP−SMX	Vanco	Aminopenicillin	Macrolide	PCN	MDR in S. Typhi and Paratyphi	Methicillin	Mono INH	Mono RIF	MDR excluding XDR in TB	XDR in TB
Acinetobacter baumannii	16.5	1.42	12.4	12.8	1.01	1.45	10.8	15.4											
Citrobacter spp.	1.06	0.0704	0.438		0.0521	0.285	0.161	0.911											
Enterobacter spp.	4.91	0.377	3.47		1.3	1.16	1.81		1.52										
Enterococcus faecalis	3.98					3.67				0.531									
Enterococcus faecium	14					13.9				0.751									
Other enterococci	2.3					2.01				0.747									
Escherichia coli	56.2	13.4		27.8	0.315	37.9		16.5	25.9		47.4								
Group A Streptococcus	2.1											2.1							
Group B Streptococcus	3.93					0.824						3.49	0.218						
Haemophilus influenzae	1.28							0.179			1.22								
Klebsiella pneumoniae	25.1	15.7		19	11.3	18.4		21.7	13.3										
Morganella spp.	0.101					0.0739	0.0166	0.0488											
Mycobacterium tuberculosis	0.0999															0.0754	4.98e−05	0.0194	0.00508
Proteus spp.	4.66	0.471				1.51		1.48	2.42		4.23								
Pseudomonas aeruginosa	13.3	1.92	10		3.88	4.85	7.3	6.99											
Salmonella Paratyphi	0.00134					0.00134								9.82e−06					
Salmonella Typhi	0.0758					0.0607								0.0181					
Non−typhoidal Salmonella	2.61e−05					2.61e−05													
Serratia spp.	0.387	0.0662	0.105		0.094	0.12	0.0499	0.257											
Shigella spp.	0.0102					0.0102													
Staphylococcus aureus	50.4					26			2.79	0.65		39.3			33.1				
Streptococcus pneumoniae	21			5.05	7.45	1.11		1.38	8.92			13.9	11						
All pathogens	221	33.5	26.5	64.8	25.4	113	20.1	64.9	54.8	2.68	52.8	58.8	11.2	0.0181	33.1	0.0754	4.98e−05	0.0194	0.00508

Counts
< 5k
NA

Monaco Deaths (count) associated with bacterial antimicrobial resistance by pathogen−drug combinations, 2019

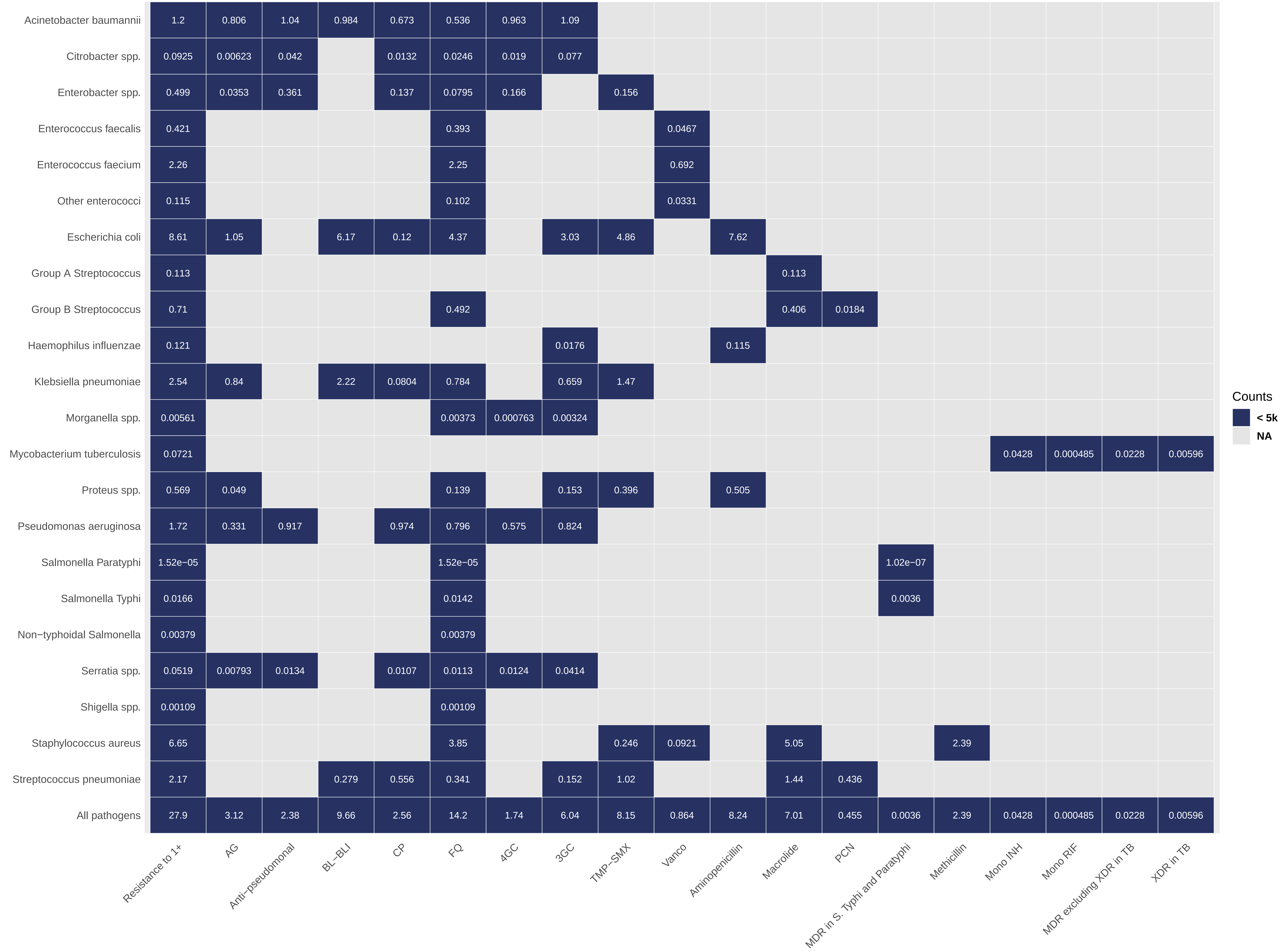

Montenegro Deaths (count) associated with bacterial antimicrobial resistance by pathogen−drug combinations, 2019

	Resistance to 1+	AG	Anti−pseudomonal	BL−BLI	CP	FQ	4GC	3GC	TMP−SMX	Vanco	Aminopenicillin	Macrolide	PCN	MDR in S. Typhi and Paratyphi	Methicillin	Mono INH	Mono RIF	MDR excluding XDR in TB	XDR in TB
Acinetobacter baumannii	39	27.6	38.3	33	30.5	34.9	37.4	37.1											
Citrobacter spp.	3.68	0.233	1.37		0.595	1.44	0.296	2.92											
Enterobacter spp.	14.1	1.77	7.8		2.33	4.51	7.39		8.58										
Enterococcus faecalis	10.4					10.2				0.556									
Enterococcus faecium	22.1					22				8.27									
Other enterococci	7.87					7.78				1.62									
Escherichia coli	74.7	17.9		45.1	5.92	35.2		53.3	47.9		63.5								
Group A Streptococcus	1.42											1.42							
Group B Streptococcus	5.19					2.65						3.62	0.0848						
Haemophilus influenzae	0.384							0.0931			0.333								
Klebsiella pneumoniae	43	18.3		37.4	4.28	29.6		42.4	30.3										
Morganella spp.	0.116					0.0801	0.0132	0.0648											
Mycobacterium tuberculosis	0.139															0.0236	0.00166	0.0782	0.036
Proteus spp.	7.28	0.656				2.24		3.54	4.68		6.54								
Pseudomonas aeruginosa	24.8	7.66	12.9		17.1	16.4	12.5	11.4											
Salmonella Paratyphi	0.00042					0.000419								3.5e−06					
Salmonella Typhi	0.243					0.22								0.028					
Non−typhoidal Salmonella	0.0357					0.0357													
Serratia spp.	1.81	0.514	0.346		0.406	0.409	1.02	1.12											
Shigella spp.	0.00123					0.00123													
Staphylococcus aureus	38.6					19.4			1.9	0.542		23.5			30.2				
Streptococcus pneumoniae	19.1			3.04	7.43	1.05		2.75	10.7			10.5	9.21						
All pathogens	314	74.6	60.7	119	68.6	188	58.6	155	104	11	70.4	39	9.3	0.028	30.2	0.0236	0.00166	0.0782	0.036

Counts
< 5k
NA

Netherlands Deaths (count) associated with bacterial antimicrobial resistance by pathogen−drug combinations, 2019

	Resistance to 1+	AG	Anti−pseudomonal	BL−BLI	CP	FQ	4GC	3GC	TMP−SMX	Vanco	Aminopenicillin	Macrolide	PCN	MDR in S. Typhi and Paratyphi	Methicillin	Mono INH	Mono RIF	MDR excluding XDR in TB	XDR in TB
Acinetobacter baumannii	364	21.3	236	280	20.9	32.9	197	305											
Citrobacter spp.	12.9	2.2	4.45		3.15	6.18	2.98	5.26											
Enterobacter spp.	177	15.5	112		52.6	15.1	80.5		58										
Enterococcus faecalis	177					171				10.6									
Enterococcus faecium	792					792				16.9									
Other enterococci	63.6					59.6				12.9									
Escherichia coli	2,760	303		1,750	44.6	933		468	1,730		2,330								
Group A Streptococcus	22.8											22.8							
Group B Streptococcus	193					22.5						184	6.13						
Haemophilus influenzae	9.48							5.76			4.86								
Klebsiella pneumoniae	618	118		168	8.37	293		273	554										
Morganella spp.	5.13					2.55	0.693	3.76											
Mycobacterium tuberculosis	12															6.91	0.0916	3.99	1.04
Proteus spp.	241	8.97				7.64		16.7	153		221								
Pseudomonas aeruginosa	274	37.1	134		105	169	88.6	82.7											
Salmonella Paratyphi	0.0574					0.0575								0.00034					
Salmonella Typhi	2.57					2.16								0.475					
Non−typhoidal Salmonella	5.59					5.59													
Serratia spp.	15.7	4.44	2.34		4.51	3.51	1.48	9.99											
Shigella spp.	1.47					1.47													
Staphylococcus aureus	1,280					338			103	9.79		1,140			196				
Streptococcus pneumoniae	341			34	43.9	146		10.9	186			67	46						
All pathogens	7,370	511	488	2,230	283	3,000	371	1,180	2,790	50.3	2,550	1,420	52.1	0.475	196	6.91	0.0916	3.99	1.04

Counts
- 5k − <10k
- < 5k
- NA

North Macedonia Deaths (count) associated with bacterial antimicrobial resistance by pathogen−drug combinations, 2019

	Resistance to 1+	AG	Anti−pseudomonal	BL−BLI	CP	FQ	4GC	3GC	TMP−SMX	Vanco	Aminopenicillin	Macrolide	PCN	MDR in S. Typhi and Paratyphi	Methicillin	Mono INH	Mono RIF	MDR excluding XDR in TB	XDR in TB
Acinetobacter baumannii	152	122	147	118	134	139	138	146											
Citrobacter spp.	13.9	0.793	5.79		2.8	4.42	2.12	11.2											
Enterobacter spp.	58.5	7.16	32		7.14	36.5	34.8		29.6										
Enterococcus faecalis	34.7					33.8				2.39									
Enterococcus faecium	80.7					80.4				25.3									
Other enterococci	29.1					28.7				5.82									
Escherichia coli	317	76.1		191	142	251		249	289		279								
Group A Streptococcus	5.51											5.51							
Group B Streptococcus	17.3					4.39						15.1	0.457						
Haemophilus influenzae	1.54							0.341			1.36								
Klebsiella pneumoniae	172	80.9		150	54.8	149		167	148										
Morganella spp.	0.647					0.378	0.129	0.43											
Mycobacterium tuberculosis	1.68															0.22	0.0165	0.989	0.453
Proteus spp.	27.7	2.55				12.7		9.8	16.3		24.7								
Pseudomonas aeruginosa	105	44	59.1		66.7	64.5	58.5	64.4											
Salmonella Paratyphi	0.00122					0.00122								7.63e−06					
Salmonella Typhi	1.24					1.12								0.141					
Non−typhoidal Salmonella	0.115					0.115													
Serratia spp.	12.1	2.93	2.41		1.68	1.59	10.5	6.46											
Shigella spp.	0.0237					0.0237													
Staphylococcus aureus	157					64.5			5.77	1.95		71			151				
Streptococcus pneumoniae	75.3			9.95	32.1	3.85		15.4	48			30.9	39.4						
All pathogens	1,260	336	247	469	441	877	244	670	536	35.4	305	123	39.8	0.141	151	0.22	0.0165	0.989	0.453

Counts
< 5k
NA

Norway Deaths (count) associated with bacterial antimicrobial resistance by pathogen−drug combinations, 2019

	Resistance to 1+	AG	Anti−pseudomonal	BL−BLI	CP	FQ	4GC	3GC	TMP−SMX	Vanco	Aminopenicillin	Macrolide	PCN	MDR in S. Typhi and Paratyphi	Methicillin	Mono INH	Mono RIF	MDR excluding XDR in TB	XDR in TB
Acinetobacter baumannii	87.8	23.1	46.8	71.2	29.6	5.19	46.8	72.8											
Citrobacter spp.	4.42	0.473	1.88		0.68	1.03	0.628	3.48											
Enterobacter spp.	43.3	3.77	30.8		8.22	3.9	14.7		17										
Enterococcus faecalis	39.8					37.7				3.61									
Enterococcus faecium	165					165				8.69									
Other enterococci	8.27					6.37				3.33									
Escherichia coli	663	93.7		404	280	198		98.1	292		485								
Group A Streptococcus	9.05											9.05							
Group B Streptococcus	50.1					5.79						48.1	1.89						
Haemophilus influenzae	10.5							0.92			10.1								
Klebsiella pneumoniae	192	40.1		149	2.66	124		46.7	109										
Morganella spp.	1.22					0.565	0.191	0.919											
Mycobacterium tuberculosis	5.52															2.89	0.00743	2.07	0.546
Proteus spp.	69.5	5.56				4.45		5.55	30.4		69								
Pseudomonas aeruginosa	81.7	13.9	37.6		49.7	33.7	38	27.1											
Salmonella Paratyphi	0.439					0.439								0.00316					
Salmonella Typhi	0.656					0.576								0.0923					
Non−typhoidal Salmonella	0.288					0.288													
Serratia spp.	3.54	0.704	1.06		1.3	0.557	0.475	2.31											
Shigella spp.	1.4					1.4													
Staphylococcus aureus	241					77.7			23.8	8.63		89			200				
Streptococcus pneumoniae	78.6			8.55	21.8	9.86		9.04	50.4			23.3	25						
All pathogens	1,760	181	118	633	394	676	101	267	524	24.3	564	169	26.9	0.0955	200	2.89	0.00743	2.07	0.546

Counts
< 5k
NA

Poland Deaths (count) associated with bacterial antimicrobial resistance by pathogen−drug combinations, 2019

	Resistance to 1+	AG	Anti−pseudomonal	BL−BLI	CP	FQ	4GC	3GC	TMP−SMX	Vanco	Aminopenicillin	Macrolide	PCN	MDR in S. Typhi and Paratyphi	Methicillin	Mono INH	Mono RIF	MDR excluding XDR in TB	XDR in TB
Acinetobacter baumannii	1,300	828	1,240	910	1,050	1,220	1,210	1,250											
Citrobacter spp.	97.4	5.73	64.3		15.3	42.6	23.4	44.6											
Enterobacter spp.	1,030	341	945		86.1	225	724		527										
Enterococcus faecalis	1,030					1,010				75.9									
Enterococcus faecium	1,740					1,730				709									
Other enterococci	322					319				39.6									
Escherichia coli	6,180	997		4,080	120	3,470		1,820	3,740		5,630								
Group A Streptococcus	31.1											31.1							
Group B Streptococcus	466					38.1						458	6.36						
Haemophilus influenzae	27.2							10.5			20								
Klebsiella pneumoniae	3,550	1,830		3,330	526	2,820		2,790	3,060										
Morganella spp.	9.35					5.14	0.959	6.61											
Mycobacterium tuberculosis	33.5															19.2	1.88	8.55	3.91
Proteus spp.	618	24.5				179		113	254		610								
Pseudomonas aeruginosa	2,020	728	1,140		1,310	1,280	998	1,050											
Salmonella Paratyphi	0.0871					0.0867								0.000654					
Salmonella Typhi	20.8					17.1								4.44					
Non−typhoidal Salmonella	0.708					0.708													
Serratia spp.	26.3	9.25	8		7.54	7.45	6.23	9.34											
Shigella spp.	0.263					0.263													
Staphylococcus aureus	3,600					1,230			305	23.9		3,050			1,840				
Streptococcus pneumoniae	1,990			266	814	80.1		369	1,440			1,040	689						
All pathogens	24,100	4,760	3,400	8,590	3,930	13,700	2,960	7,470	9,330	848	6,260	4,590	695	4.44	1,840	19.2	1.88	8.55	3.91

Counts
- **10k − <25k**
- **5k − <10k**
- **< 5k**
- **NA**

Portugal Deaths (count) associated with bacterial antimicrobial resistance by pathogen−drug combinations, 2019

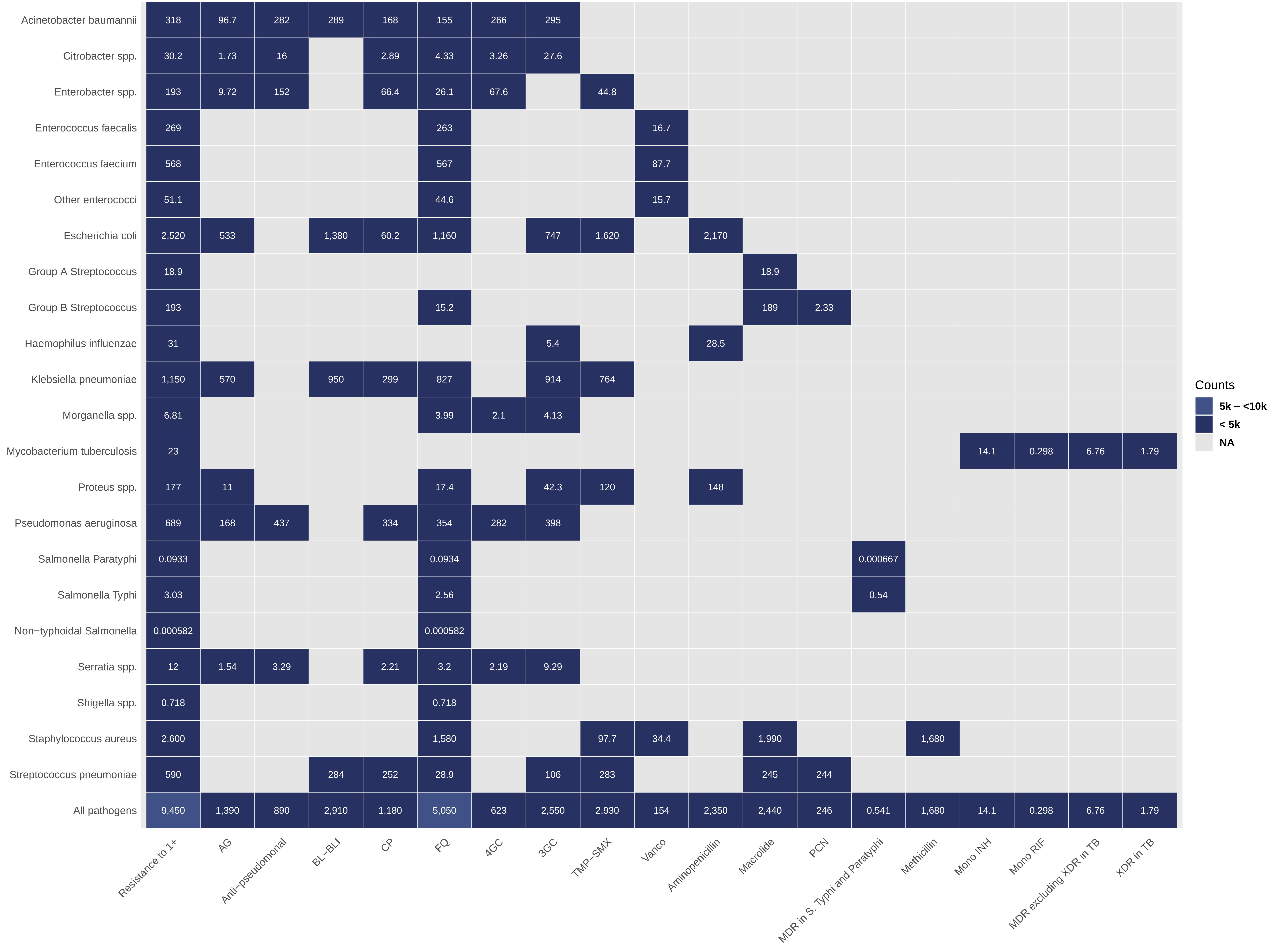

Republic of Moldova Deaths (count) associated with bacterial antimicrobial resistance by pathogen−drug combinations, 20

	Resistance to 1+	AG	Anti−pseudomonal	BL−BLI	CP	FQ	4GC	3GC	TMP−SMX	Vanco	Aminopenicillin	Macrolide	PCN	MDR in S. Typhi and Paratyphi	Methicillin	Mono INH	Mono RIF	MDR excluding XDR in TB	XDR in TB
Acinetobacter baumannii	112	93.5	110	96.7	100	108	109	110											
Citrobacter spp.	11.9	1.39	3.18		2.07	3.69	1.88	9.52											
Enterobacter spp.	79.2	10.5	45		11.9	20.7	51.1		36.5										
Enterococcus faecalis	124					121				9.14									
Enterococcus faecium	214					213				58.6									
Other enterococci	49.4					49.3				8.36									
Escherichia coli	768	164		521	66.5	429		449	542		671								
Group A Streptococcus	6.43											6.43							
Group B Streptococcus	31.8					3.88						30.1	1.67						
Haemophilus influenzae	3.33							1.01			2.69								
Klebsiella pneumoniae	421	229		369	27.6	278		358	338										
Morganella spp.	1.27					0.511	0.118	1.08											
Mycobacterium tuberculosis	131															11.8	3.6	79.2	36.3
Proteus spp.	61.3	19.1				26.9		11.7	29.9		57.9								
Pseudomonas aeruginosa	233	86	123		155	144	140	139											
Salmonella Paratyphi	0.0372					0.0371								0.000202					
Salmonella Typhi	6.11					4.9								1.49					
Non−typhoidal Salmonella	0.576					0.576													
Serratia spp.	21.6	7.97	6.48		3.15	2	14.8	14.3											
Shigella spp.	0.097					0.097													
Staphylococcus aureus	352					101			14.9	7.52		174			319				
Streptococcus pneumoniae	184			20.3	43.1	6.49		31.7	172			51.9	33.3						
All pathogens	2,810	611	288	1,010	410	1,510	317	1,130	1,130	83.6	732	262	35	1.49	319	11.8	3.6	79.2	36.3

Counts
< 5k
NA

Romania Deaths (count) associated with bacterial antimicrobial resistance by pathogen−drug combinations, 2019

	Resistance to 1+	AG	Anti−pseudomonal	BL−BLI	CP	FQ	4GC	3GC	TMP−SMX	Vanco	Aminopenicillin	Macrolide	PCN	MDR in S. Typhi and Paratyphi	Methicillin	Mono INH	Mono RIF	MDR excluding XDR in TB	XDR in TB
Acinetobacter baumannii	677	513	650	423	632	628	662	603											
Citrobacter spp.	73.2	3.39	53.8		9.74	10.4	12	56.9											
Enterobacter spp.	272	29.1	41.9		58.4	162	55		176										
Enterococcus faecalis	685					675				46									
Enterococcus faecium	1,110					1,090				626									
Other enterococci	245					244				24.4									
Escherichia coli	4,040	853		2,400	76.3	1,920		1,540	1,460		3,810								
Group A Streptococcus	86.7											86.7							
Group B Streptococcus	251					24.9						243	1.55						
Haemophilus influenzae	47							36.4			18.5								
Klebsiella pneumoniae	2,320	1,810		2,170	1,340	1,730		1,860	1,570										
Morganella spp.	7.01					4.65	1.74	3.9											
Mycobacterium tuberculosis	130															34.9	12.6	56.5	25.8
Proteus spp.	321	18				177		157	158		263								
Pseudomonas aeruginosa	1,730	1,040	1,230		1,300	1,160	995	1,000											
Salmonella Paratyphi	0.0747					0.0744								0.000583					
Salmonella Typhi	19.8					16.1								4.57					
Non−typhoidal Salmonella	0.00515					0.00515													
Serratia spp.	163	31.7	29.6		22.2	7.4	161	93.3											
Shigella spp.	0.00279					0.00279													
Staphylococcus aureus	3,140					508			106	36.7		2,610			2,350				
Streptococcus pneumoniae	1,230			129	621	65.4		465	430			724	868						
All pathogens	16,500	4,300	2,000	5,130	4,060	8,420	1,890	5,820	3,900	733	4,100	3,670	870	4.57	2,350	34.9	12.6	56.5	25.8

Counts
- 10k − <25k
- 5k − <10k
- < 5k
- NA

Russian Federation Deaths (count) associated with bacterial antimicrobial resistance by pathogen−drug combinations, 2019

	Resistance to 1+	AG	Anti−pseudomonal	BL−BLI	CP	FQ	4GC	3GC	TMP−SMX	Vanco	Aminopenicillin	Macrolide	PCN	MDR in S. Typhi and Paratyphi	Methicillin	Mono INH	Mono RIF	MDR excluding XDR in TB	XDR in TB
Acinetobacter baumannii	3,820	3,390	3,740	3,440	3,370	3,680	3,620	3,740											
Citrobacter spp.	275	26.8	165		29.5	79.9	37.6	191											
Enterobacter spp.	3,570	310	2,680		574	233	2,220		1,410										
Enterococcus faecalis	4,410					4,330				315									
Enterococcus faecium	7,610					7,590				737									
Other enterococci	1,800					1,790				329									
Escherichia coli	32,000	9,160		23,600	3,450	19,500		22,700	21,000		29,200								
Group A Streptococcus	219											219							
Group B Streptococcus	2,290					236						2,220	64						
Haemophilus influenzae	89.3							47.9			52								
Klebsiella pneumoniae	16,500	11,200		16,000	12,700	14,100		15,200	14,100										
Morganella spp.	59.2					19.1	5.27	53.2											
Mycobacterium tuberculosis	4,670															834	386	2,360	1,080
Proteus spp.	2,440	1,370				552		103	1,140		2,260								
Pseudomonas aeruginosa	11,300	6,420	7,920		8,720	8,130	6,660	7,830											
Salmonella Paratyphi	2.17					2.16								0.0145					
Salmonella Typhi	206					161								59.5					
Non−typhoidal Salmonella	16.5					16.5													
Serratia spp.	937	571	425		263	24.5	659	592											
Shigella spp.	4.89					4.89													
Staphylococcus aureus	11,200					7,490			586	222		4,930			8,590				
Streptococcus pneumoniae	8,650			651	936	360		2,150	8,020			1,030	2,490						
All pathogens	112,000	32,500	14,900	43,700	30,000	68,300	13,200	52,700	46,200	1,600	31,500	8,400	2,560	59.5	8,590	834	386	2,360	1,080

Counts
- >=100k
- 50k − <75k
- 25k − <50k
- 10k − <25k
- 5k − <10k
- < 5k
- NA

San Marino Deaths (count) associated with bacterial antimicrobial resistance by pathogen−drug combinations, 2019

	Resistance to 1+	AG	Anti−pseudomonal	BL−BLI	CP	FQ	4GC	3GC	TMP−SMX	Vanco	Aminopenicillin	Macrolide	PCN	MDR in S. Typhi and Paratyphi	Methicillin	Mono INH	Mono RIF	MDR excluding XDR in TB	XDR in TB
Acinetobacter baumannii	0.93	0.554	0.8	0.769	0.446	0.44	0.676	0.84											
Citrobacter spp.	0.0782	0.00508	0.0341		0.0208	0.0223	0.0112	0.0605											
Enterobacter spp.	0.321	0.0219	0.23		0.0852	0.0634	0.113		0.0999										
Enterococcus faecalis	0.247					0.229				0.0301									
Enterococcus faecium	1.36					1.36				0.275									
Other enterococci	0.0891					0.0819				0.0207									
Escherichia coli	4.87	0.587		3.47	0.0712	2.56		1.79	2.79		4.2								
Group A Streptococcus	0.0679											0.0679							
Group B Streptococcus	0.334					0.173						0.232	0.0103						
Haemophilus influenzae	0.0552							0.0115			0.0505								
Klebsiella pneumoniae	1.54	0.432		1.36	0.055	0.33		0.463	0.91										
Morganella spp.	0.00518					0.0032	0.000668	0.00329											
Mycobacterium tuberculosis	0.0176															0.0111	8.91e−05	0.00508	0.00134
Proteus spp.	0.386	0.0323				0.0894		0.0968	0.248		0.355								
Pseudomonas aeruginosa	0.883	0.152	0.503		0.5	0.399	0.288	0.388											
Salmonella Paratyphi	1.32e−05					1.32e−05								9.13e−08					
Salmonella Typhi	0.00752					0.00642								0.00143					
Non−typhoidal Salmonella	0.00336					0.00336													
Serratia spp.	0.0355	0.00443	0.0106		0.00771	0.00775	0.0136	0.026											
Shigella spp.	0.000703					0.000703													
Staphylococcus aureus	2.99					1.76			0.114	0.0413		1.83			2				
Streptococcus pneumoniae	1.03			0.15	0.258	0.18		0.0737	0.439			0.72	0.172						
All pathogens	15.3	1.79	1.58	5.75	1.44	7.7	1.1	3.75	4.6	0.367	4.61	2.85	0.182	0.00143	2	0.0111	8.91e−05	0.00508	0.00134

Counts
< 5k
NA

Serbia Deaths (count) associated with bacterial antimicrobial resistance by pathogen−drug combinations, 2019

	Resistance to 1+	AG	Anti−pseudomonal	BL−BLI	CP	FQ	4GC	3GC	TMP−SMX	Vanco	Aminopenicillin	Macrolide	PCN	MDR in S. Typhi and Paratyphi	Methicillin	Mono INH	Mono RIF	MDR excluding XDR in TB	XDR in TB
Acinetobacter baumannii	436	354	419	360	378	407	424	413											
Citrobacter spp.	51.3	18.2	12.2		5.96	18	12.1	40.6											
Enterobacter spp.	261	101	196		144	123	173		98.6										
Enterococcus faecalis	232					228				12.4									
Enterococcus faecium	474					473				129									
Other enterococci	121					120				10.9									
Escherichia coli	1,800	472		1,100	52.8	1,090		928	1,170		1,560								
Group A Streptococcus	62.6											62.6							
Group B Streptococcus	106					10.3						102	1.75						
Haemophilus influenzae	8.62							2.02			7.52								
Klebsiella pneumoniae	986	477		858	328	652		957	688										
Morganella spp.	7.46					6.28	0.663	5.95											
Mycobacterium tuberculosis	5.27															1.93	0.0667	2.24	1.03
Proteus spp.	162	72.7				89.9		78.2	66.1		140								
Pseudomonas aeruginosa	617	219	261		395	400	382	423											
Salmonella Paratyphi	0.00496					0.00495								3.18e−05					
Salmonella Typhi	6.89					5.95								1.17					
Non−typhoidal Salmonella	0.3					0.3													
Serratia spp.	69.4	21.2	3.06		3.51	9.37	59.2	55											
Shigella spp.	0.038					0.038													
Staphylococcus aureus	1,180					720			45.3	12.6		997			636				
Streptococcus pneumoniae	493			113	336	29.9		60.8	235			266	292						
All pathogens	7,080	1,740	891	2,430	1,640	4,380	1,050	2,960	2,300	165	1,700	1,430	294	1.17	636	1.93	0.0667	2.24	1.03

Counts
- 5k − <10k
- < 5k
- NA

Slovakia Deaths (count) associated with bacterial antimicrobial resistance by pathogen−drug combinations, 2019

	Resistance to 1+	AG	Anti−pseudomonal	BL−BLI	CP	FQ	4GC	3GC	TMP−SMX	Vanco	Aminopenicillin	Macrolide	PCN	MDR in S. Typhi and Paratyphi	Methicillin	Mono INH	Mono RIF	MDR excluding XDR in TB	XDR in TB
Acinetobacter baumannii	128	36.4	102	97	69	78.1	125	121											
Citrobacter spp.	12	0.745	4.55		3.11	2.63	1.36	9.65											
Enterobacter spp.	83.7	5.75	45.8		17.1	32	40.9		46										
Enterococcus faecalis	76.7					75.2				2.77									
Enterococcus faecium	255					254				106									
Other enterococci	38.2					37.3				8.11									
Escherichia coli	941	218		534	12.9	622		448	519		797								
Group A Streptococcus	17.9											17.9							
Group B Streptococcus	51.5					7.77						48.7	1.46						
Haemophilus influenzae	6.94							3.13			4.82								
Klebsiella pneumoniae	514	279		441	45.1	402		364	491										
Morganella spp.	1.17					0.803	0.112	0.659											
Mycobacterium tuberculosis	0.84															0.139	0.0671	0.435	0.199
Proteus spp.	74.1	3.5				34.8		22.6	32.2		70.7								
Pseudomonas aeruginosa	337	128	181		215	249	215	185											
Salmonella Paratyphi	0.00242					0.00241								1.94e−05					
Salmonella Typhi	2.87					2.48								0.471					
Non−typhoidal Salmonella	0.000299					0.000299													
Serratia spp.	12.9	2.1	1.85		2.65	1.99	9.68	7.2											
Shigella spp.	0.0244					0.0244													
Staphylococcus aureus	653					352			26.4	8.95		498			391				
Streptococcus pneumoniae	209			15.8	75.3	15		33.3	104			134	81.2						
All pathogens	3,420	674	335	1,090	440	2,170	392	1,190	1,220	125	872	698	82.6	0.471	391	0.139	0.0671	0.435	0.199

Counts: < 5k; NA

Slovenia Deaths (count) associated with bacterial antimicrobial resistance by pathogen−drug combinations, 2019

	Resistance to 1+	AG	Anti−pseudomonal	BL−BLI	CP	FQ	4GC	3GC	TMP−SMX	Vanco	Aminopenicillin	Macrolide	PCN	MDR in S. Typhi and Paratyphi	Methicillin	Mono INH	Mono RIF	MDR excluding XDR in TB	XDR in TB
Acinetobacter baumannii	55.7	18	39	45.1	24	29.4	24.1	52.6											
Citrobacter spp.	2.77	0.288	1.01		0.543	0.778	0.322	2.05											
Enterobacter spp.	29.3	1.4	16.1		3.59	4.55	14.6		15.9										
Enterococcus faecalis	48.7					47.9				2.73									
Enterococcus faecium	100					100				3.91									
Other enterococci	6.68					6.3				1.39									
Escherichia coli	284	38.4		178	12.5	136		66.3	116		256								
Group A Streptococcus	6.29											6.29							
Group B Streptococcus	13.5					3.08						11.8	0.346						
Haemophilus influenzae	2.14							0.363			1.96								
Klebsiella pneumoniae	110	19.8		63.9	1.09	75.1		69.7	77.2										
Morganella spp.	0.294					0.135	0.0524	0.221											
Mycobacterium tuberculosis	0.094															0.0521	0.000452	0.0285	0.0129
Proteus spp.	26.6	0.837				10.6		2.71	11.7		26								
Pseudomonas aeruginosa	73.8	14.8	35.3		40.8	38.7	19.4	35.4											
Salmonella Paratyphi	0.0081					0.00807								5.76e−05					
Salmonella Typhi	0.438					0.384								0.0637					
Non−typhoidal Salmonella	0.000299					0.000299													
Serratia spp.	2.41	0.301	0.342		0.857	0.436	0.198	1.76											
Shigella spp.	0.00753					0.00753													
Staphylococcus aureus	114					47.2			10.7	4.02		57.3			82.9				
Streptococcus pneumoniae	71.4			14.2	13.7	9.58		4.55	50			32.7	10.8						
All pathogens	948	93.8	91.8	301	97.2	510	58.7	236	282	12	284	108	11.1	0.0638	82.9	0.0521	0.000452	0.0285	0.0129

Counts
< 5k
NA

Spain Deaths (count) associated with bacterial antimicrobial resistance by pathogen−drug combinations, 2019

	Resistance to 1+	AG	Anti−pseudomonal	BL−BLI	CP	FQ	4GC	3GC	TMP−SMX	Vanco	Aminopenicillin	Macrolide	PCN	MDR in S. Typhi and Paratyphi	Methicillin	Mono INH	Mono RIF	MDR excluding XDR in TB	XDR in TB
Acinetobacter baumannii	1,080	441	797	1,020	719	743	743	947											
Citrobacter spp.	89.8	6.8	43.8		14.1	18.8	10.9	75.4											
Enterobacter spp.	586	40.5	407		148	189	328		83.2										
Enterococcus faecalis	945					928				34.9									
Enterococcus faecium	2,200					2,190				97.7									
Other enterococci	245					239				29.2									
Escherichia coli	9,140	1,780		4,000	176	4,770		2,310	4,790		8,440								
Group A Streptococcus	242											242							
Group B Streptococcus	337					66.7						301	9.38						
Haemophilus influenzae	81.4							14			75.8								
Klebsiella pneumoniae	2,480	749		2,080	227	1,310		1,550	1,330										
Morganella spp.	21.8					15.6	2.12	12.6											
Mycobacterium tuberculosis	37.9															25.6	0.465	9.37	2.5
Proteus spp.	770	25.3				111		62.7	529		708								
Pseudomonas aeruginosa	2,100	509	1,070		1,410	1,220	912	802											
Salmonella Paratyphi	0.129					0.129								0.00113					
Salmonella Typhi	8.74					7.57								1.38					
Non−typhoidal Salmonella	30.1					30.1													
Serratia spp.	41.4	5.06	10.3		8.27	10.5	7.38	32.6											
Shigella spp.	1.61					1.61													
Staphylococcus aureus	5,320					3,000			244	45.4		3,600			3,350				
Streptococcus pneumoniae	1,550			247	852	114		334	702			720	855						
All pathogens	27,300	3,560	2,330	7,350	3,560	15,000	2,000	6,140	7,680	207	9,230	4,860	864	1.38	3,350	25.6	0.465	9.37	2.5

Counts
25k − <50k
10k − <25k
5k − <10k
< 5k
NA

Sweden Deaths (count) associated with bacterial antimicrobial resistance by pathogen−drug combinations, 2019

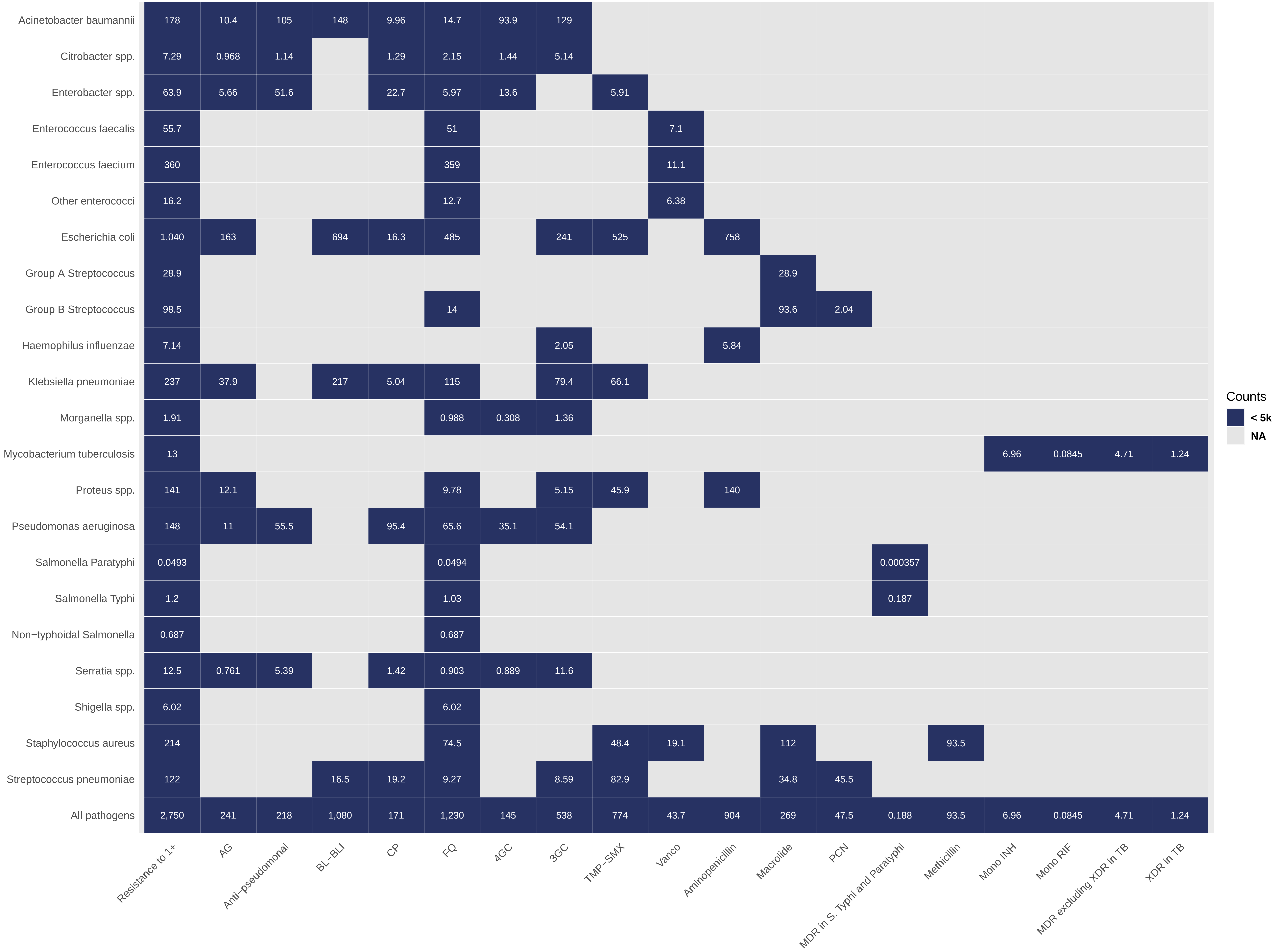

Switzerland Deaths (count) associated with bacterial antimicrobial resistance by pathogen−drug combinations, 2019

	Resistance to 1+	AG	Anti−pseudomonal	BL−BLI	CP	FQ	4GC	3GC	TMP−SMX	Vanco	Aminopenicillin	Macrolide	PCN	MDR in S. Typhi and Paratyphi	Methicillin	Mono INH	Mono RIF	MDR excluding XDR in TB	XDR in TB
Acinetobacter baumannii	157	67.5	72.2	120	84.4	26.3	57.6	134											
Citrobacter spp.	10.9	0.85	1.43		1.15	1.53	1.09	10											
Enterobacter spp.	58.7	4.99	40.8		7.03	4.49	17.3		24.8										
Enterococcus faecalis	23					14.9				9.53									
Enterococcus faecium	312					311				14.8									
Other enterococci	13.2					10.5				4.87									
Escherichia coli	987	48.8		588	6.74	357		182	527		849								
Group A Streptococcus	14.2											14.2							
Group B Streptococcus	74.4					5.56						72.6	1.35						
Haemophilus influenzae	11.5							1.36			11.1								
Klebsiella pneumoniae	176	18.2		133	8.56	80.2		65.1	91.3										
Morganella spp.	1.48					0.806	0.189	1.03											
Mycobacterium tuberculosis	5.16															1.41	0.0113	2.96	0.779
Proteus spp.	109	7.87				18.3		8.91	47.3		107								
Pseudomonas aeruginosa	141	18.3	67.5		55.1	78.5	57	66.2											
Salmonella Paratyphi	0.0141					0.0141								8.32e−05					
Salmonella Typhi	2.86					2.49								0.435					
Non−typhoidal Salmonella	0.449					0.449													
Serratia spp.	4.9	0.769	1.7		1.12	0.816	0.828	3.69											
Shigella spp.	1.52					1.52													
Staphylococcus aureus	371					139			33.6	12.8		218			236				
Streptococcus pneumoniae	144			15.4	38.1	17.4		52.4	79.4			52.2	25						
All pathogens	2,620	167	184	856	202	1,070	134	524	803	42	967	357	26.4	0.435	236	1.41	0.0113	2.96	0.779

Counts

< 5k

NA

Tajikistan Deaths (count) associated with bacterial antimicrobial resistance by pathogen−drug combinations, 2019

	Resistance to 1+	AG	Anti−pseudomonal	BL−BLI	CP	FQ	4GC	3GC	TMP−SMX	Vanco	Aminopenicillin	Macrolide	PCN	MDR in S. Typhi and Paratyphi	Methicillin	Mono INH	Mono RIF	MDR excluding XDR in TB	XDR in TB
Acinetobacter baumannii	463	324	458	408	364	442	449	446											
Citrobacter spp.	45.4	10.5	11.1		17.5	18.5	12.6	34.5											
Enterobacter spp.	231	56.2	123		43.6	41.2	116		162										
Enterococcus faecalis	83.8					80.4				8.66									
Enterococcus faecium	153					153				33									
Other enterococci	92.7					92.6				5.14									
Escherichia coli	673	188		447	117	506		462	483		612								
Group A Streptococcus	7.42											7.42							
Group B Streptococcus	88.2					23.9						76.7	3.64						
Haemophilus influenzae	21.4							11.9			12.8								
Klebsiella pneumoniae	595	242		492	88.4	340		513	520										
Morganella spp.	3.5					2.92	0.62	1.49											
Mycobacterium tuberculosis	339															61.6	46	159	72.8
Proteus spp.	77.2	28				32.9		32.5	46.5		74								
Pseudomonas aeruginosa	336	76.7	159		192	165	156	277											
Salmonella Paratyphi	0.0828					0.0823								0.00251					
Salmonella Typhi	21.5					18.5								5.18					
Non−typhoidal Salmonella	1.44					1.44													
Serratia spp.	48.8	13.7	16.2		9.28	11	29.2	31.1											
Shigella spp.	18.7					18.7													
Staphylococcus aureus	535					227			188	5.92		390			400				
Streptococcus pneumoniae	930			201	478	33.7		119	780			373	454						
All pathogens	4,770	939	767	1,550	1,310	2,210	763	1,930	2,180	52.7	699	847	457	5.18	400	61.6	46	159	72.8

Counts
< 5k
NA

Turkey Deaths (count) associated with bacterial antimicrobial resistance by pathogen−drug combinations, 2019

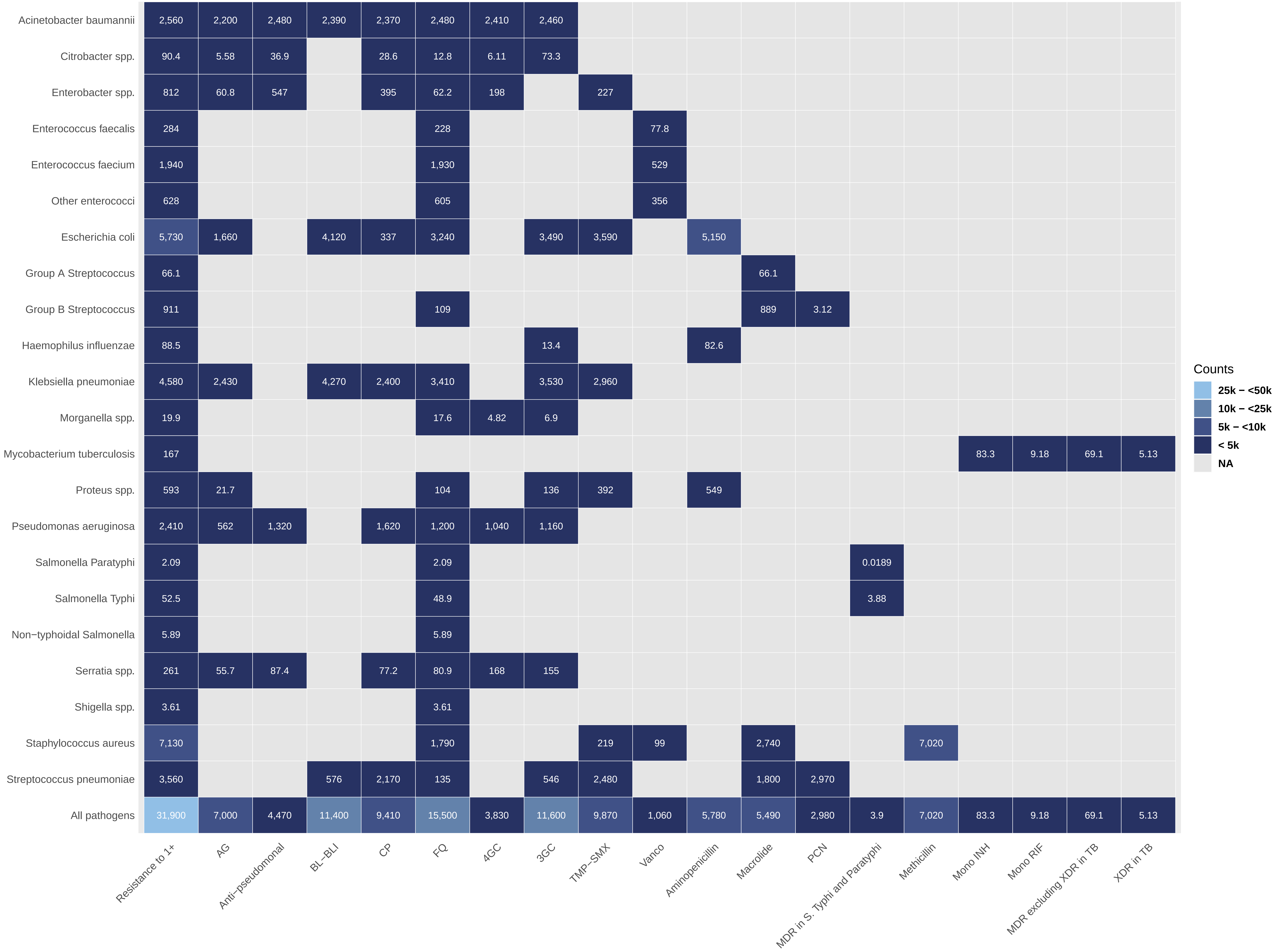

Turkmenistan Deaths (count) associated with bacterial antimicrobial resistance by pathogen−drug combinations, 2019

	Resistance to 1+	AG	Anti−pseudomonal	BL−BLI	CP	FQ	4GC	3GC	TMP−SMX	Vanco	Aminopenicillin	Macrolide	PCN	MDR in S. Typhi and Paratyphi	Methicillin	Mono INH	Mono RIF	MDR excluding XDR in TB	XDR in TB
Acinetobacter baumannii	188	145	185	164	158	174	171	181											
Citrobacter spp.	16.5	2.85	3.45		6.34	8.67	2.36	10.5											
Enterobacter spp.	88	18.6	48.2		16.6	25.2	43.6		54.5										
Enterococcus faecalis	52.2					48.4				7.08									
Enterococcus faecium	152					150				64.5									
Other enterococci	51.6					51.5				3.05									
Escherichia coli	526	180		382	39.6	349		323	366		482								
Group A Streptococcus	5.72											5.72							
Group B Streptococcus	53.9					24						39.5	4.96						
Haemophilus influenzae	14.2							6.28			10.4								
Klebsiella pneumoniae	417	159		361	36.5	218		321	356										
Morganella spp.	1.74					1.43	0.371	0.673											
Mycobacterium tuberculosis	219															68.9	4.72	99.5	45.5
Proteus spp.	51.8	10.3				20.5		20.3	32.2		47.5								
Pseudomonas aeruginosa	236	76.9	117		122	136	114	173											
Salmonella Paratyphi	0.00306					0.00305								2.39e−05					
Salmonella Typhi	9.8					7.5								2.96					
Non−typhoidal Salmonella	0.144					0.144													
Serratia spp.	25.7	8.71	8.02		4.81	5.28	15.4	15.4											
Shigella spp.	1.11					1.11													
Staphylococcus aureus	426					204			80.6	5.32		356			200				
Streptococcus pneumoniae	334			67.1	149	12.4		47.9	283			151	154						
All pathogens	2,870	601	361	974	533	1,440	347	1,100	1,170	79.9	540	551	159	2.96	200	68.9	4.72	99.5	45.5

Counts
< 5k
NA

Ukraine Deaths (count) associated with bacterial antimicrobial resistance by pathogen−drug combinations, 2019

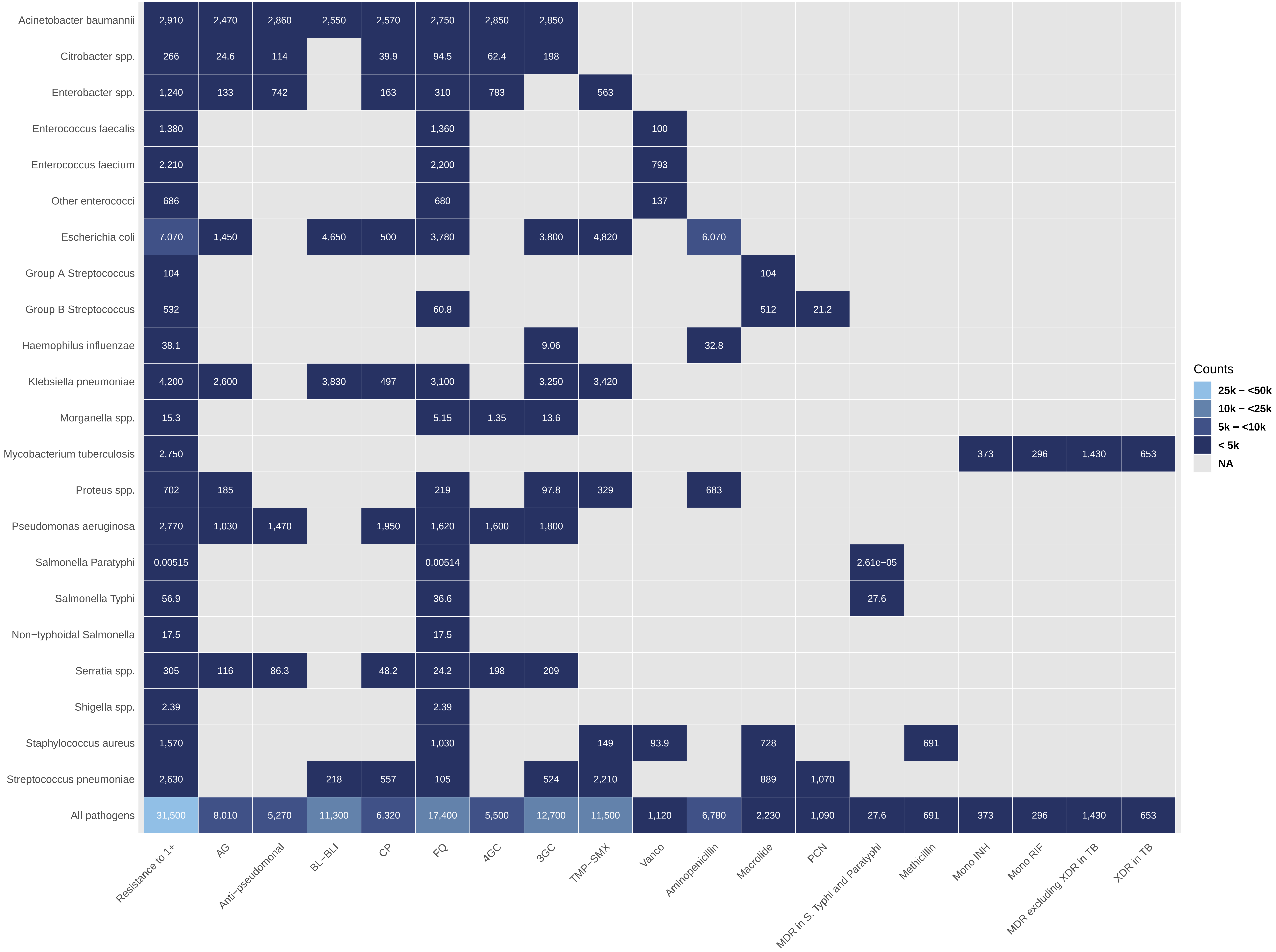

United Kingdom Deaths (count) associated with bacterial antimicrobial resistance by pathogen−drug combinations, 2019

	Resistance to 1+	AG	Anti−pseudomonal	BL−BLI	CP	FQ	4GC	3GC	TMP−SMX	Vanco	Aminopenicillin	Macrolide	PCN	MDR in S. Typhi and Paratyphi	Methicillin	Mono INH	Mono RIF	MDR excluding XDR in TB	XDR in TB
Acinetobacter baumannii	1,350	82	44.2	872	67.6	102	57.3	1,200											
Citrobacter spp.	153	9.39	97.5		12.4	16.4	12.4	133											
Enterobacter spp.	756	55.7	494		220	54	407		157										
Enterococcus faecalis	957					894				115									
Enterococcus faecium	3,140					3,130				1,070									
Other enterococci	222					186				76.8									
Escherichia coli	12,400	2,190		8,800	163	4,350		2,630	7,390		11,300								
Group A Streptococcus	120											120							
Group B Streptococcus	270					98.7						192	16.8						
Haemophilus influenzae	247							25.4			240								
Klebsiella pneumoniae	3,670	591		2,650	124	1,280		1,410	2,690										
Morganella spp.	21.7					10.8	3.65	15.6											
Mycobacterium tuberculosis	42.1															28	1.46	10	2.63
Proteus spp.	997	73.1				74.1		66.7	701		880								
Pseudomonas aeruginosa	1,870	276	1,020		956	939	600	662											
Salmonella Paratyphi	0.117					0.117								0.000902					
Salmonella Typhi	11.8					10.1								1.92					
Non−typhoidal Salmonella	69.2					69.2													
Serratia spp.	77.9	8.01	17.8		11.1	16	9.29	68.6											
Shigella spp.	8					8													
Staphylococcus aureus	6,400					2,780			462	152		4,390			3,540				
Streptococcus pneumoniae	2,390			314	252	730		170	1,630			426	541						
All pathogens	35,200	3,290	1,680	12,600	1,810	14,700	1,090	6,380	13,000	1,420	12,400	5,130	558	1.92	3,540	28	1.46	10	2.63

Counts
- 25k − <50k
- 10k − <25k
- 5k − <10k
- < 5k
- NA

Uzbekistan Deaths (count) associated with bacterial antimicrobial resistance by pathogen−drug combinations, 2019

	Resistance to 1+	AG	Anti−pseudomonal	BL−BLI	CP	FQ	4GC	3GC	TMP−SMX	Vanco	Aminopenicillin	Macrolide	PCN	MDR in S. Typhi and Paratyphi	Methicillin	Mono INH	Mono RIF	MDR excluding XDR in TB	XDR in TB
Acinetobacter baumannii	956	711	942	846	797	853	890	919											
Citrobacter spp.	83.9	15.4	20.9		27.5	43.8	12	55.3											
Enterobacter spp.	501	98.4	292		88.1	120	271		278										
Enterococcus faecalis	297					276				38									
Enterococcus faecium	892					888				219									
Other enterococci	274					274				15.7									
Escherichia coli	3,000	832		2,220	223	1,780		1,740	2,090		2,770								
Group A Streptococcus	42.1											42.1							
Group B Streptococcus	380					147						300	35.1						
Haemophilus influenzae	87.5							41.7			60.6								
Klebsiella pneumoniae	2,590	957		2,180	283	1,310		2,220	2,190										
Morganella spp.	8.48					6.63	1.71	3.85											
Mycobacterium tuberculosis	1,460															189	200	736	338
Proteus spp.	285	53.7				102		118	179		261								
Pseudomonas aeruginosa	1,420	428	691		731	816	730	1,030											
Salmonella Paratyphi	2.15					2.14								0.0143					
Salmonella Typhi	51.4					45.5								7.32					
Non−typhoidal Salmonella	0.667					0.667													
Serratia spp.	142	50.6	50		27.6	32.9	64	96.5											
Shigella spp.	0.955					0.955													
Staphylococcus aureus	2,540					1,270			410	34.4		2,050			1,160				
Streptococcus pneumoniae	2,180			521	840	85.8		304	1,940			950	638						
All pathogens	17,200	3,150	2,000	5,760	3,020	8,050	1,970	6,530	7,080	307	3,090	3,340	673	7.34	1,160	189	200	736	338

Counts
- **10k − <25k**
- **5k − <10k**
- **< 5k**
- **NA**

Albania DALYs (count) attributable to bacterial antimicrobial resistance by pathogen−drug combinations, 2019

	Resistance to 1+	AG	Anti−pseudomonal	BL−BLI	CP	FQ	4GC	3GC	Vanco	Aminopenicillin	TMP−SMX	PCN	Macrolide	Methicillin	Mono RIF	MDR excluding XDR in TB	XDR in TB	MDR in S. Typhi and Paratyphi	Mono INH
Acinetobacter baumannii	359	37.7	31.3	0.137	153	125	0.161	12											
Citrobacter spp.	31.3	0.498	9.87		4.04	5.36	0.718	10.8											
Enterobacter spp.	135	5.48	40.2		31.3	20.2	18				19.5								
Enterococcus faecalis	157					140			17.2										
Enterococcus faecium	493					337			157										
Other enterococci	86.1					67.9			18.2										
Escherichia coli	2,100	101		188	228	506		685		93.1	301								
Group A Streptococcus	22.9												21.5						
Group B Streptococcus	105					65						3.19	39.2						
Haemophilus influenzae	16.6							4.63		12									
Klebsiella pneumoniae	1,150	122		45.9	113	185		531			157								
Morganella spp.	2.04					0.826	0.356	0.855											
Mycobacterium tuberculosis	4.42														0.181	2.09	0.98		1.18
Neisseria gonorrhoeae	0.916					0.881		0.0354											
Proteus spp.	84.2	1.56				13.9		50.3		5.28	13.1								
Pseudomonas aeruginosa	587	19.9	91		208	149	27.5	91.4											
Salmonella Paratyphi	0.0254					0.0253												0.000106	
Salmonella Typhi	15.3					14												1.25	
Non−typhoidal Salmonella	0.26					0.26													
Serratia spp.	27.3	2.19	4.68		5.35	2.36	8.49	4.28											
Shigella spp.	0.459					0.454													
Staphylococcus aureus	1,360					99.1			19.2		24.1		128	1,090					
Streptococcus pneumoniae	653			1.99	299	11.5		4.54			131	115	86.8						
All pathogens	7,400	290	177	236	1,040	1,740	55.3	1,390	211	110	645	119	276	1,090	0.181	2.09	0.98	1.19	1.18

Counts
<100k
NA

Andorra DALYs (count) attributable to bacterial antimicrobial resistance by pathogen−drug combinations, 2019

	Resistance to 1+	AG	Anti−pseudomonal	BL−BLI	CP	FQ	4GC	3GC	Vanco	Aminopenicillin	TMP−SMX	PCN	Macrolide	Methicillin	Mono RIF	MDR excluding XDR in TB	XDR in TB	MDR in S. Typhi and Paratyphi	Mono INH
Acinetobacter baumannii	8.05	0.199	2.92	0.35	1.14	0.85	0.182	2.4											
Citrobacter spp.	0.705	0.011	0.274		0.0549	0.0999	0.0168	0.248											
Enterobacter spp.	2.78	0.0715	1.3		0.844	0.144	0.159				0.263								
Enterococcus faecalis	3.06					2.63			0.432										
Enterococcus faecium	16					12.8			3.11										
Other enterococci	0.638					0.469			0.168										
Escherichia coli	51.3	1.32		8.83	0.924	15.2		14.2		3.33	7.44								
Group A Streptococcus	0.284												0.274						
Group B Streptococcus	2.38					1.15						0.105	1.14						
Haemophilus influenzae	0.355							0.0535		0.302									
Klebsiella pneumoniae	11.7	1.16		3.53	0.885	1.31		2.65			2.19								
Morganella spp.	0.0513					0.0204	0.00586	0.025											
Mycobacterium tuberculosis	0.0694														0.000632	0.0144	0.00385		0.0497
Neisseria gonorrhoeae	0.00608					0.00587		0.000208											
Proteus spp.	1.77	0.0577				0.224		0.728		0.374	0.392								
Pseudomonas aeruginosa	6.73	0.133	1.34		2.79	1.8	0.315	0.355											
Salmonella Paratyphi	0.00041					0.000408												1.31e−06	
Salmonella Typhi	0.114					0.108												0.00614	
Non−typhoidal Salmonella	0.0319					0.0319													
Serratia spp.	0.46	0.0113	0.111		0.0882	0.0349	0.104	0.11											
Shigella spp.	0.0209					0.0209													
Staphylococcus aureus	14.6					2.91			0.719		0.541		2.41	8.05					
Streptococcus pneumoniae	6.56			0.0961	2.18	0.682		0.155			1.66	1.52	0.273						
All pathogens	128	2.96	5.95	12.8	8.91	40.5	0.783	20.9	4.43	4	12.5	1.62	4.08	8.05	0.000632	0.0144	0.00385	0.00629	0.0504

Counts
<100k
NA

Armenia DALYs (count) attributable to bacterial antimicrobial resistance by pathogen−drug combinations, 2019

	Resistance to 1+	AG	Anti−pseudomonal	BL−BLI	CP	FQ	4GC	3GC	Vanco	Aminopenicillin	TMP−SMX	PCN	Macrolide	Methicillin	Mono RIF	MDR excluding XDR in TB	XDR in TB	MDR in S. Typhi and Paratyphi	Mono INH
Acinetobacter baumannii	942	106	113	0.229	383	324	2.93	13.9											
Citrobacter spp.	72.5	0.88	10.4		16	20.5	1.52	23.2											
Enterobacter spp.	377	16.4	158		55.1	48.2	58.1				40.2								
Enterococcus faecalis	347					316			30.9										
Enterococcus faecium	766					626			140										
Other enterococci	262					237			24.7										
Escherichia coli	2,650	133		274	145	719		864		111	398								
Group A Streptococcus	15.4												15.9						
Group B Streptococcus	160					57.1						12.6	90.3						
Haemophilus influenzae	19							6.17		12.8									
Klebsiella pneumoniae	2,020	232		98.1	184	317		884			302								
Morganella spp.	10.3					4.18	1.22	4.94											
Mycobacterium tuberculosis	562														30.3	323	157		53.9
Neisseria gonorrhoeae	2.17					2.13		0.0425											
Proteus spp.	150	15				20.6		71.1		21.1	22.1								
Pseudomonas aeruginosa	1,130	39.7	208		470	251	56.5	103											
Salmonella Paratyphi	1.57					1.56												0.00452	
Salmonella Typhi	22.2					20.8												1.36	
Non−typhoidal Salmonella	0.508					0.508													
Serratia spp.	142	9.63	37		23.8	9.13	47.2	15.4											
Shigella spp.	6.97					6.97													
Staphylococcus aureus	1,240					156			38.2		31.6		116	900					
Streptococcus pneumoniae	741			5.99	209	21.2		20			339	122	24.9						
All pathogens	11,600	553	526	379	1,490	3,160	168	2,010	234	145	1,130	134	247	900	30.3	323	157	1.34	56

Counts
<100k
NA

Austria DALYs (count) attributable to bacterial antimicrobial resistance by pathogen−drug combinations, 2019

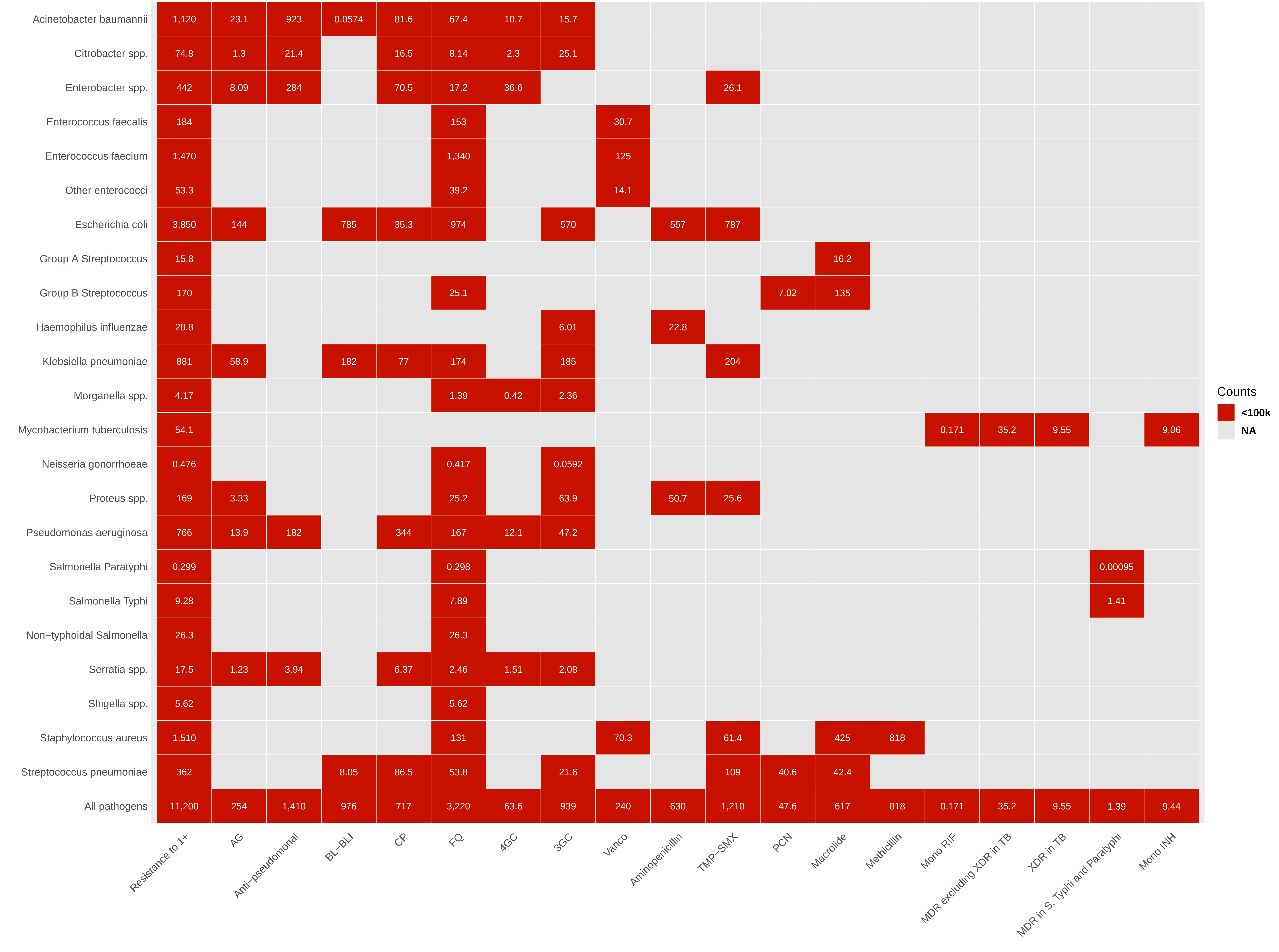

Azerbaijan DALYs (count) attributable to bacterial antimicrobial resistance by pathogen−drug combinations, 2019

	Resistance to 1+	AG	Anti−pseudomonal	BL−BLI	CP	FQ	4GC	3GC	Vanco	Aminopenicillin	TMP−SMX	PCN	Macrolide	Methicillin	Mono RIF	MDR excluding XDR in TB	XDR in TB	MDR in S. Typhi and Paratyphi	Mono INH
Acinetobacter baumannii	3,280	342	364	1.26	1,400	1,140	4.44	33.6											
Citrobacter spp.	262	9.49	36		64.6	75	5.06	71.9											
Enterobacter spp.	1,330	76.9	475		256	119	194				201								
Enterococcus faecalis	733					614			119										
Enterococcus faecium	2,090					1,610			475										
Other enterococci	631					597			33.7										
Escherichia coli	8,220	547		950	495	2,030		2,590		347	1,260								
Group A Streptococcus	57.2												58.4						
Group B Streptococcus	1,250					665						112	473						
Haemophilus influenzae	362							210		152									
Klebsiella pneumoniae	8,290	931		442	674	1,130		3,710			1,390								
Morganella spp.	19.4					11	3.04	5.3											
Mycobacterium tuberculosis	6,630														460	3,910	1,910		341
Neisseria gonorrhoeae	10.8					10.4		0.327											
Proteus spp.	424	24.3				63.3		235		42.8	61.5								
Pseudomonas aeruginosa	4,330	136	707		1,490	1,100	257	634											
Salmonella Paratyphi	0.395					0.394												0.00144	
Salmonella Typhi	137					117												20.4	
Non−typhoidal Salmonella	2.81					2.81													
Serratia spp.	409	34.7	115		82.2	39.3	64.5	73.9											
Shigella spp.	16.7					16.7													
Staphylococcus aureus	5,800					616			113		424		1,140	3,500					
Streptococcus pneumoniae	7,100			131	2,860	153		35.9			2,800	532	588						
All pathogens	51,400	2,100	1,700	1,520	7,320	10,100	528	7,600	741	538	6,150	644	2,260	3,500	460	3,910	1,910	20.1	365

Counts: **<100k** | **NA**

Belarus DALYs (count) attributable to bacterial antimicrobial resistance by pathogen−drug combinations, 2019

	Resistance to 1+	AG	Anti−pseudomonal	BL−BLI	CP	FQ	4GC	3GC	Vanco	Aminopenicillin	TMP−SMX	PCN	Macrolide	Methicillin	Mono RIF	MDR excluding XDR in TB	XDR in TB	MDR in S. Typhi and Paratyphi	Mono INH
Acinetobacter baumannii	3,010	340	404	0.272	993	1,200	75.5	3.87											
Citrobacter spp.	277	4.66	110		57.1	24.7	2.6	77.9											
Enterobacter spp.	1,120	34.7	557		183	89.9	108				148								
Enterococcus faecalis	1,650					1,540			106										
Enterococcus faecium	2,340					1,860			483										
Other enterococci	492					466			25.7										
Escherichia coli	7,900	772		843	271	1,390		3,580		26.5	1,020								
Group A Streptococcus	37.6												37.1						
Group B Streptococcus	396					34.5						8.61	367						
Haemophilus influenzae	22.2							13		9.23									
Klebsiella pneumoniae	5,170	944		132	820	727		1,780			773								
Morganella spp.	12.3					2.42	0.896	8.99											
Mycobacterium tuberculosis	3,790														16	2,480	1,230		56.9
Neisseria gonorrhoeae	2.02					1.98		0.0342											
Proteus spp.	292	11.1				69.1		93.5		76	46								
Pseudomonas aeruginosa	2,810	97.9	253		1,400	897	71.5	89.1											
Salmonella Paratyphi	0.0501					0.0499												0.000174	
Salmonella Typhi	38.4					25.9												12.1	
Non−typhoidal Salmonella	22.4					22.4													
Serratia spp.	165	12.7	29		38	7.23	21.8	56.7											
Shigella spp.	10.6					10.2													
Staphylococcus aureus	2,160					105			72.7		71		272	1,630					
Streptococcus pneumoniae	1,700			23.7	265	86.7		57.4			830	313	120						
All pathogens	33,400	2,220	1,350	999	4,030	8,560	280	5,750	688	106	2,890	321	787	1,630	16	2,480	1,230	12.7	60.9

Counts
<100k
NA

Belgium DALYs (count) attributable to bacterial antimicrobial resistance by pathogen−drug combinations, 2019

	Resistance to 1+	AG	Anti−pseudomonal	BL−BLI	CP	FQ	4GC	3GC	TMP−SMX	Vanco	Aminopenicillin	PCN	Macrolide	Methicillin	Mono RIF	MDR excluding XDR in TB	XDR in TB	MDR in S. Typhi and Paratyphi	Mono INH
Acinetobacter baumannii	1,150	27.8	234	203	118	127	113	331											
Citrobacter spp.	150	1.82	77		18.6	21.1	0.84	30.7											
Enterobacter spp.	592	12.9	297		102	152	12.2		15.9										
Enterococcus faecalis	289					202				86.6									
Enterococcus faecium	2,210					2,060				153									
Other enterococci	150					111				38.9									
Escherichia coli	6,970	244		1,690	207	1,630		908	1,310		986								
Group A Streptococcus	22.5												23.1						
Group B Streptococcus	257					60.5						9.22	178						
Haemophilus influenzae	128							17			111								
Klebsiella pneumoniae	2,300	190		371	209	466		825	240										
Morganella spp.	14.9					4.11	1.01	9.77											
Mycobacterium tuberculosis	47.6														0.552	28.7	7.76		10.3
Neisseria gonorrhoeae	0.586					0.48		0.107											
Proteus spp.	247	6.01				40.7		58.5	49.2		89.6								
Pseudomonas aeruginosa	1,610	43.2	316		787	327	67.5	67.3											
Salmonella Paratyphi	0.14					0.14												0.000493	
Salmonella Typhi	13.3					11.6												1.73	
Non−typhoidal Salmonella	80.6					80.6													
Serratia spp.	75.7	1.55	16.6		10.7	6.64	5.29	34.9											
Shigella spp.	14.8					14.8													
Staphylococcus aureus	3,680					508			115	124			701	2,230					
Streptococcus pneumoniae	1,240			7.02	696	27.5		13.6	333			3.9	154						
All pathogens	21,200	527	940	2,270	2,150	5,850	200	2,300	2,070	402	1,190	13.1	1,060	2,230	0.552	28.7	7.76	1.79	10.3

Counts
- <100k
- NA

Bosnia and Herzegovina DALYs (count) attributable to bacterial antimicrobial resistance by pathogen−drug combinations, 2019

	Resistance to 1+	AG	Anti−pseudomonal	BL−BLI	CP	FQ	4GC	3GC	Vanco	Aminopenicillin	TMP−SMX	PCN	Macrolide	Methicillin	Mono RIF	MDR excluding XDR in TB	XDR in TB	MDR in S. Typhi and Paratyphi	Mono INH
Acinetobacter baumannii	1,290	118	233	0.645	407	503	2.91	22.6											
Citrobacter spp.	183	2.36	41.6		26.3	86.1	4.91	21.4											
Enterobacter spp.	464	22.8	156		53.4	98.8	74.8				62								
Enterococcus faecalis	419					394			24.7										
Enterococcus faecium	773					548			225										
Other enterococci	206					169			37.5										
Escherichia coli	2,050	156		314	323	372		526		91.1	270								
Group A Streptococcus	20.6												20.6						
Group B Streptococcus	105					21.6						4.45	80.2						
Haemophilus influenzae	15.3							4.24		11.1									
Klebsiella pneumoniae	1,680	257		83.9	394	247		601			101								
Morganella spp.	5.5					2.07	0.882	2.55											
Mycobacterium tuberculosis	25														0.12	14	6.62		4.37
Neisseria gonorrhoeae	0.788					0.751		0.0367											
Proteus spp.	136	8.66				33.5		62.1		17	15.6								
Pseudomonas aeruginosa	881	36	141		391	223	27.8	62.9											
Salmonella Paratyphi	0.0172					0.0171												5.83e−05	
Salmonella Typhi	12.1					11.3												0.821	
Non−typhoidal Salmonella	0.84					0.84													
Serratia spp.	87.2	8.01	20		16.6	5.87	20.7	15.7											
Shigella spp.	0.448					0.496													
Staphylococcus aureus	1,080					93.9			20.2		21.2		155	787					
Streptococcus pneumoniae	725			0.734	253	22.1		181			73.2	126	69.3						
All pathogens	10,200	609	591	399	1,860	2,830	132	1,500	308	119	540	130	327	787	0.12	14	6.62	0.794	4.33

Counts
<100k
NA

Bulgaria DALYs (count) attributable to bacterial antimicrobial resistance by pathogen−drug combinations, 2019

	Resistance to 1+	AG	Anti−pseudomonal	BL−BLI	CP	FQ	4GC	3GC	Vanco	Aminopenicillin	TMP−SMX	PCN	Macrolide	Methicillin	Mono RIF	MDR excluding XDR in TB	XDR in TB	MDR in S. Typhi and Paratyphi	Mono INH
Acinetobacter baumannii	2,230	235	320	2	934	721	12.7	5.17											
Citrobacter spp.	241	2.7	62.4		48.8	48.4	4.28	74.1											
Enterobacter spp.	1,180	90.7	343		76.4	299	261				108								
Enterococcus faecalis	1,740					1,660			86.5										
Enterococcus faecium	2,430					1,840			590										
Other enterococci	492					394			97.6										
Escherichia coli	9,950	795		1,230	316	2,480		3,240		536	1,350								
Group A Streptococcus	71.8												77.5						
Group B Streptococcus	275					60						13.1	213						
Haemophilus influenzae	72.1							38.3		33.8									
Klebsiella pneumoniae	6,560	876		124	1,610	1,080		2,370			508								
Morganella spp.	16.8					7.55	2.5	6.73											
Mycobacterium tuberculosis	157														7.91	82.5	39		26.8
Neisseria gonorrhoeae	1.4					1.33		0.0736											
Proteus spp.	1,060	27.9				59.9		921		0.075	53								
Pseudomonas aeruginosa	2,510	126	677		890	597	118	97.8											
Salmonella Paratyphi	0.0663					0.066												0.000258	
Salmonella Typhi	65					56.2												8.63	
Non−typhoidal Salmonella	1.31					1.31													
Serratia spp.	421	29	71.5		36.6	10.2	232	42.4											
Shigella spp.	6.17					6.17													
Staphylococcus aureus	2,660					188			106		95.2		388	1,880					
Streptococcus pneumoniae	1,750			54.6	845	183		48.4			74.2	310	233						
All pathogens	33,900	2,190	1,470	1,410	4,760	9,690	631	6,840	880	570	2,190	323	892	1,880	7.91	82.5	39	8.32	27.9

Counts
<100k
NA

Croatia DALYs (count) attributable to bacterial antimicrobial resistance by pathogen−drug combinations, 2019

	Resistance to 1+	AG	Anti−pseudomonal	BL−BLI	CP	FQ	4GC	3GC	Vanco	Aminopenicillin	TMP−SMX	PCN	Macrolide	Methicillin	Mono RIF	MDR excluding XDR in TB	XDR in TB	MDR in S. Typhi and Paratyphi	Mono INH
Acinetobacter baumannii	1,680	189	30.9	0.239	820	621	8.1	9.74											
Citrobacter spp.	107	1.8	36.1		13	15	2.48	38.1											
Enterobacter spp.	588	7.54	135		73.9	121	204				48.3								
Enterococcus faecalis	506					473			33.4										
Enterococcus faecium	1,040					701			335										
Other enterococci	137					125			12.3										
Escherichia coli	2,580	105		435	54.7	614		418		202	749								
Group A Streptococcus	15.9												16						
Group B Streptococcus	105					15.8						2.63	84.8						
Haemophilus influenzae	6.12							3.96		2.16									
Klebsiella pneumoniae	1,370	164		119	184	314		469			118								
Morganella spp.	8.54					0.522	0.649	7.37											
Mycobacterium tuberculosis	9.68														0.0359	4.84	2.26		2.6
Neisseria gonorrhoeae	1.36					1.35		0.0118											
Proteus spp.	128	4.01				28.4		54		22.4	17.5								
Pseudomonas aeruginosa	936	25.7	92.9		417	245	126	29.5											
Salmonella Paratyphi	0.0219					0.0219												8.53e−05	
Salmonella Typhi	4.76					4.23												0.528	
Non−typhoidal Salmonella	0.676					0.676													
Serratia spp.	42.8	1.49	8.15		6.43	3.79	10.8	12.1											
Shigella spp.	0.421					0.437													
Staphylococcus aureus	1,510					195			35.3		29.9		160	1,090					
Streptococcus pneumoniae	553			34.6	260	12.7		4.08			90.5	69.4	82.9						
All pathogens	11,300	498	303	590	1,830	3,490	352	1,050	416	228	1,050	72	342	1,090	0.0359	4.84	2.26	0.506	2.54

Counts
<100k
NA

Cyprus DALYs (count) attributable to bacterial antimicrobial resistance by pathogen−drug combinations, 2019

	Resistance to 1+	AG	Anti−pseudomonal	BL−BLI	CP	FQ	4GC	3GC	Vanco	Aminopenicillin	TMP−SMX	PCN	Macrolide	Methicillin	Mono RIF	MDR excluding XDR in TB	XDR in TB	MDR in S. Typhi and Paratyphi	Mono INH
Acinetobacter baumannii	125	12	0.141	1.22	57.5	45.4	0.931	7.98											
Citrobacter spp.	12.4	0.15	3.15		1.22	1.91	0.83	5.18											
Enterobacter spp.	42	0.831	15.5		13.3	8.98	1.12				2.27								
Enterococcus faecalis	77.3					70.7			6.69										
Enterococcus faecium	239					119			120										
Other enterococci	27.9					22.5			5.39										
Escherichia coli	760	40.9		67.6	20.1	219		230		98.1	84.6								
Group A Streptococcus	4.48												4.58						
Group B Streptococcus	21.3					2.94						1.15	19.3						
Haemophilus influenzae	5.75							2.71		3.04									
Klebsiella pneumoniae	314	42.2		13	101	72.1		69.2			16.5								
Morganella spp.	1.06					0.632	0.112	0.314											
Mycobacterium tuberculosis	4.34														0.00143	1.84	0.499		1.94
Neisseria gonorrhoeae	0.112					0.106		0.00542											
Proteus spp.	24.7	0.755				8.12		8.75		3.85	3.28								
Pseudomonas aeruginosa	124	1.31	28.3		51.7	32.9	2.52	7.16											
Salmonella Paratyphi	0.00813					0.0081												2.87e−05	
Salmonella Typhi	1.49					1.33												0.158	
Non−typhoidal Salmonella	0.00029					0.00029													
Serratia spp.	5.83	0.169	0.924		1.58	0.374	0.805	1.98											
Shigella spp.	0.524					0.524													
Staphylococcus aureus	504					54			8.32		14.1		51.1	376					
Streptococcus pneumoniae	120			0.239	70.6	3.61		1.36			18.9	19.4	5.92						
All pathogens	2,420	98.3	48	82	317	664	6.32	335	140	105	140	20.6	80.1	376	0.00143	1.84	0.499	0.153	1.99

Counts
<100k
NA

Czechia DALYs (count) attributable to bacterial antimicrobial resistance by pathogen−drug combinations, 2019

	Resistance to 1+	AG	Anti−pseudomonal	BL−BLI	CP	FQ	4GC	3GC	Vanco	Aminopenicillin	TMP−SMX	PCN	Macrolide	Methicillin	Mono RIF	MDR excluding XDR in TB	XDR in TB	MDR in S. Typhi and Paratyphi	Mono INH
Acinetobacter baumannii	2,140	89.1	71.1	703	442	435	28.1	367											
Citrobacter spp.	272	3.68	77.1		56.7	22.5	3.98	108											
Enterobacter spp.	778	19.8	231		120	72.2	149				174								
Enterococcus faecalis	1,350					1,300			48										
Enterococcus faecium	2,280					1,780			497										
Other enterococci	243					204			39.5										
Escherichia coli	5,300	266		1,610	114	1,260		1,040		204	819								
Group A Streptococcus	143												144						
Group B Streptococcus	297					81.3						9.39	203						
Haemophilus influenzae	27.2							13.1		14.1									
Klebsiella pneumoniae	3,440	548		350	122	628		1,340			448								
Morganella spp.	3.29					0.969	0.833	1.49											
Mycobacterium tuberculosis	43.6														0.429	25.4	12		5.59
Neisseria gonorrhoeae	2.3					2.15		0.151											
Proteus spp.	198	6.29				18.7		40.5		79.7	51.5								
Pseudomonas aeruginosa	1,960	52.6	299		897	564	31.7	116											
Salmonella Paratyphi	0.783					0.78												0.00319	
Salmonella Typhi	16					13.5												2.32	
Non−typhoidal Salmonella	8.37					8.37													
Serratia spp.	84.3	2.57	12.4		27.1	14.9	11.1	16.4											
Shigella spp.	2.18					2.18													
Staphylococcus aureus	2,470					220			81.3		76.6		544	1,550					
Streptococcus pneumoniae	921			30.7	186	115		43.7			392	76.5	76.8						
All pathogens	22,000	988	690	2,690	1,960	6,740	225	3,080	666	299	1,970	85.8	968	1,550	0.429	25.4	12	2.37	5.74

Counts
- **<100k**
- **NA**

Denmark DALYs (count) attributable to bacterial antimicrobial resistance by pathogen−drug combinations, 2019

	Resistance to 1+	AG	Anti−pseudomonal	BL−BLI	CP	FQ	4GC	3GC	Vanco	Aminopenicillin	TMP−SMX	PCN	Macrolide	Methicillin	Mono RIF	MDR excluding XDR in TB	XDR in TB	MDR in S. Typhi and Paratyphi	Mono INH
Acinetobacter baumannii	545	9.4	135	143	46.8	31.9	28.5	152											
Citrobacter spp.	52.4	0.905	17.5		4.89	8.9	3.76	16.4											
Enterobacter spp.	264	8.83	116		34.3	15.4	13.9				76.4								
Enterococcus faecalis	406					390			16.2										
Enterococcus faecium	1,200					1,010			192										
Other enterococci	212					116			95.4										
Escherichia coli	2,910	126		530	80.5	444		361		617	753								
Group A Streptococcus	10.4												10.3						
Group B Streptococcus	133					21.9						3.25	102						
Haemophilus influenzae	38.9							7.81		31.1									
Klebsiella pneumoniae	899	54.6		145	27.1	90.9		198			384								
Morganella spp.	2.73					1.01	0.509	1.21											
Mycobacterium tuberculosis	15.7														0.00695	9.89	2.67		3.18
Neisseria gonorrhoeae	0.161					0.148		0.0131											
Proteus spp.	101	3.47				5.22		16		30.5	45.5								
Pseudomonas aeruginosa	299	5.2	41.8		144	61.9	18	27.8											
Salmonella Paratyphi	2.15					2.14												0.00698	
Salmonella Typhi	5.73					5.11												0.596	
Non−typhoidal Salmonella	0.0161					0.0161													
Serratia spp.	26.2	0.742	4.78		4.67	2.04	1.05	12.9											
Shigella spp.	3.77					3.77													
Staphylococcus aureus	663					53.1			67.2		127		101	315					
Streptococcus pneumoniae	315			5.37	84.4	31.3		81.8			60	38.4	12.8						
All pathogens	8,100	209	315	823	427	2,290	65.8	875	371	678	1,450	41.7	227	315	0.00695	9.89	2.67	0.622	3.28

Counts: **<100k** | **NA**

Estonia DALYs (count) attributable to bacterial antimicrobial resistance by pathogen−drug combinations, 2019

	Resistance to 1+	AG	Anti−pseudomonal	BL−BLI	CP	FQ	4GC	3GC	Vanco	Aminopenicillin	TMP−SMX	PCN	Macrolide	Methicillin	Mono RIF	MDR excluding XDR in TB	XDR in TB	MDR in S. Typhi and Paratyphi	Mono INH
Acinetobacter baumannii	464	21.2	149	5.71	122	99.4	30.1	36.6											
Citrobacter spp.	40.6	0.547	11.2		8.77	1.65	0.768	17.7											
Enterobacter spp.	114	2.27	47.8		28.4	7.12	12.8				15.7								
Enterococcus faecalis	180					168			11.8										
Enterococcus faecium	267					245			21.8										
Other enterococci	35.7					32.4			3.34										
Escherichia coli	566	22.8		74.3	7.75	114		85.6		104	157								
Group A Streptococcus	6.51												6.66						
Group B Streptococcus	39.3					5.04						1.4	32.2						
Haemophilus influenzae	2.84							1.18		1.66									
Klebsiella pneumoniae	325	31		26.3	14.4	52.9		91.4			109								
Morganella spp.	1.21					0.153	0.0871	0.973											
Mycobacterium tuberculosis	168														1.43	106	51.7		9.26
Neisseria gonorrhoeae	0.225					0.223		0.00235											
Proteus spp.	27.6	1.26				3.09		7.77		9.62	5.95								
Pseudomonas aeruginosa	142	2.55	23		83.5	27.3	2.11	3.61											
Salmonella Paratyphi	0.0497					0.0495												0.000233	
Salmonella Typhi	1.75					1.55												0.208	
Non−typhoidal Salmonella	1.63e−56					1.63e−56													
Serratia spp.	20.3	1.26	3.74		4.81	1.06	2.7	6.73											
Shigella spp.	2.87					2.8													
Staphylococcus aureus	157					10.7			11.2		10.6		43.7	80.6					
Streptococcus pneumoniae	139			1.07	42	5.49		2.83			79.7	1.07	6.43						
All pathogens	2,700	82.9	234	107	312	778	48.5	254	48.1	115	378	2.47	88.8	80.6	1.43	106	51.7	0.198	9.74

Counts
<100k
NA

Finland DALYs (count) attributable to bacterial antimicrobial resistance by pathogen−drug combinations, 2019

	Resistance to 1+	AG	Anti−pseudomonal	BL−BLI	CP	FQ	4GC	3GC	Vanco	Aminopenicillin	TMP−SMX	PCN	Macrolide	Methicillin	Mono RIF	MDR excluding XDR in TB	XDR in TB	MDR in S. Typhi and Paratyphi	Mono INH
Acinetobacter baumannii	548	13.6	177	26.8	45.8	21.7	148	115											
Citrobacter spp.	49.1	0.807	11.8		12.3	4.4	1.57	18.3											
Enterobacter spp.	226	4.87	101		70.6	13.4	12.9				23.5								
Enterococcus faecalis	265					215			49.6										
Enterococcus faecium	1,010					974			36.4										
Other enterococci	38.1					18.5			19.6										
Escherichia coli	1,910	132		350	46.8	367		314		349	353								
Group A Streptococcus	21.1												21						
Group B Streptococcus	109					19.2						7.28	78.8						
Haemophilus influenzae	20.3							7.78		12.5									
Klebsiella pneumoniae	577	40.1		152	49.6	82.3		94.6			158								
Morganella spp.	2.43					0.921	0.252	1.26											
Mycobacterium tuberculosis	43.2														0.027	30.2	8.21		4.91
Neisseria gonorrhoeae	0.469					0.46		0.00968											
Proteus spp.	109	3.9				7.77		44.4		36	19.4								
Pseudomonas aeruginosa	383	3.91	55.9		157	114	40.6	10.8											
Salmonella Paratyphi	0.116					0.116												0.000395	
Salmonella Typhi	4.09					3.59												0.513	
Non−typhoidal Salmonella	0.0865					0.0865													
Serratia spp.	42.3	0.871	7.12		11.2	2.58	11.7	8.79											
Shigella spp.	1.59					1.57													
Staphylococcus aureus	509					36.6			59.6		42.6		47.5	323					
Streptococcus pneumoniae	296			2.21	88.8	16.5		16.3			78.6	59.7	34						
All pathogens	6,170	200	353	531	482	1,900	215	631	165	395	675	67	184	323	0.027	30.2	8.21	0.495	5.07

Counts
<100k
NA

France DALYs (count) attributable to bacterial antimicrobial resistance by pathogen−drug combinations, 2019

	Resistance to 1+	AG	Anti−pseudomonal	BL−BLI	CP	FQ	4GC	3GC	Vanco	Aminopenicillin	TMP−SMX	PCN	Macrolide	Methicillin	Mono RIF	MDR excluding XDR in TB	XDR in TB	MDR in S. Typhi and Paratyphi	Mono INH
Acinetobacter baumannii	6,130	168	961	1,380	623	598	141	2,260											
Citrobacter spp.	580	9.89	245		60.5	51.1	43.4	170											
Enterobacter spp.	2,960	43.1	1,300		508	366	327				416								
Enterococcus faecalis	1,940					1,620			322										
Enterococcus faecium	10,200					9,750			405										
Other enterococci	431					302			130										
Escherichia coli	33,400	1,290		8,870	1,260	5,380		4,920		5,210	6,520								
Group A Streptococcus	318												331						
Group B Streptococcus	685					184						44.4	453						
Haemophilus influenzae	458							54		404									
Klebsiella pneumoniae	13,800	2,480		1,530	697	2,240		5,150			1,660								
Morganella spp.	66					15.2	4.59	46.2											
Mycobacterium tuberculosis	468														4.69	292	79.7		95.8
Neisseria gonorrhoeae	3.1					2.98		0.128											
Proteus spp.	957	59.1				105		371		282	135								
Pseudomonas aeruginosa	8,440	270	2,240		3,690	1,650	205	379											
Salmonella Paratyphi	0.38					0.378												0.00142	
Salmonella Typhi	70					58.8												11.6	
Non−typhoidal Salmonella	151					151													
Serratia spp.	404	8.71	114		55.9	29	16.9	179											
Shigella spp.	26					26													
Staphylococcus aureus	17,000					1,750			420		517		2,580	11,800					
Streptococcus pneumoniae	11,200			18.7	3,330	4,820		123			439	1,730	744						
All pathogens	110,000	4,330	4,860	11,800	10,200	29,100	738	13,600	1,280	5,900	9,680	1,780	4,090	11,800	4.69	292	79.7	11.6	89.5

Counts
- **100k – <500k**
- **<100k**
- **NA**

Georgia DALYs (count) attributable to bacterial antimicrobial resistance by pathogen−drug combinations, 2019

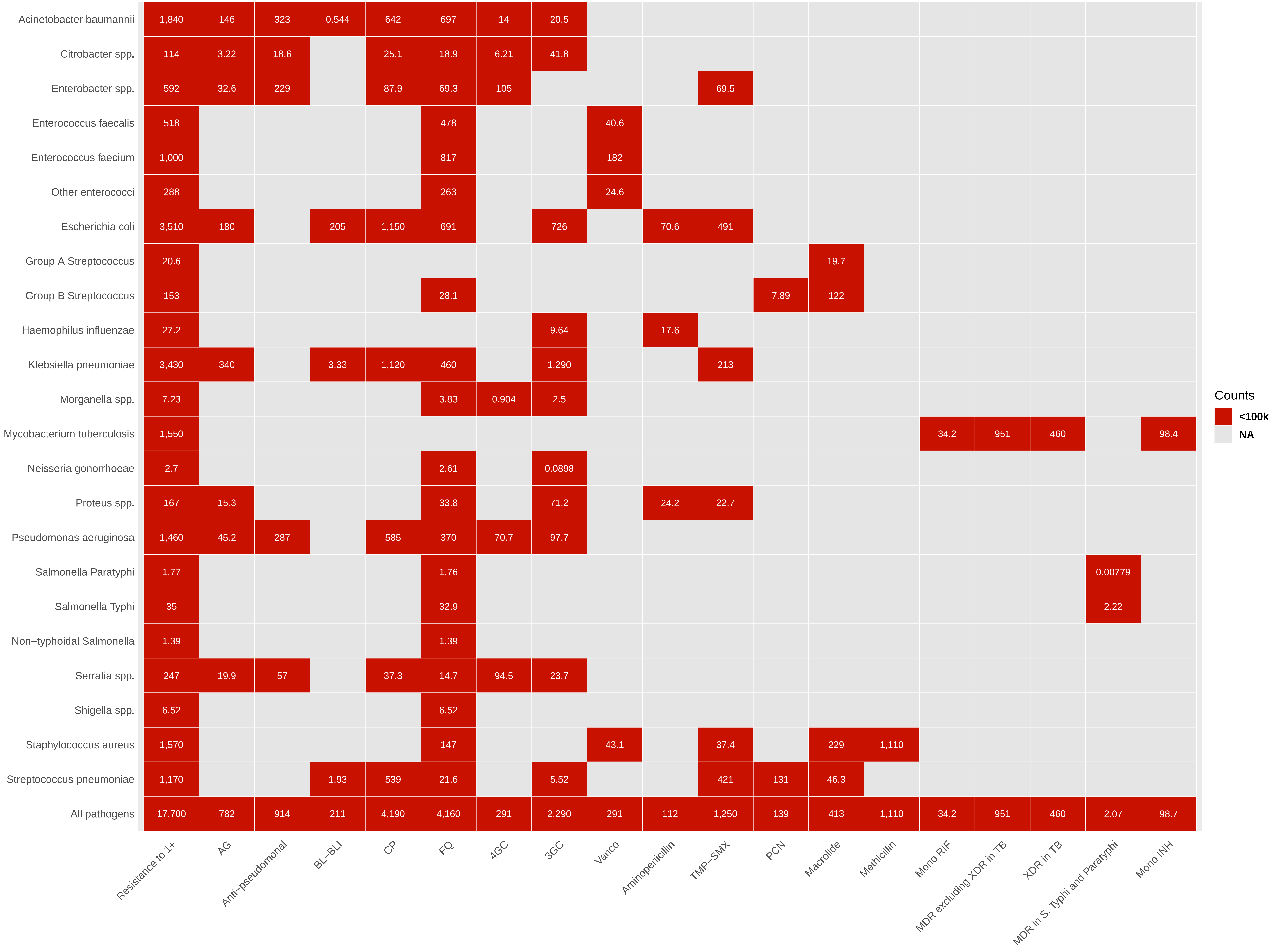

Germany DALYs (count) attributable to bacterial antimicrobial resistance by pathogen−drug combinations, 2019

	Resistance to 1+	AG	Anti−pseudomonal	BL−BLI	CP	FQ	4GC	3GC	Vanco	Aminopenicillin	TMP−SMX	PCN	Macrolide	Methicillin	Mono RIF	MDR excluding XDR in TB	XDR in TB	MDR in S. Typhi and Paratyphi	Mono INH
Acinetobacter baumannii	7,710	160	1,660	521	780	763	1,150	2,670											
Citrobacter spp.	862	17.7	349		103	119	27	247											
Enterobacter spp.	4,390	88.2	2,040		904	383	526				437								
Enterococcus faecalis	5,060					4,710			354										
Enterococcus faecium	21,500					16,200			5,330										
Other enterococci	772					482			290										
Escherichia coli	57,000	2,470		16,600	1,100	13,000		9,030		5,760	9,050								
Group A Streptococcus	180												193						
Group B Streptococcus	2,030					346						67.1	1,550						
Haemophilus influenzae	369							86.9		282									
Klebsiella pneumoniae	15,500	1,550		4,590	813	2,490		4,280			1,750								
Morganella spp.	33.2					8.69	6.46	18.1											
Mycobacterium tuberculosis	522														6.5	345	94.3		77.9
Neisseria gonorrhoeae	4.25					3.94		0.306											
Proteus spp.	1,720	76.7				163		533		612	356								
Pseudomonas aeruginosa	12,800	233	2,860		6,220	2,850	425	217											
Salmonella Paratyphi	0.437					0.436												0.0013	
Salmonella Typhi	106					85.1												20.7	
Non−typhoidal Salmonella	37.4					37.4													
Serratia spp.	740	13.9	102		117	69.8	47.1	391											
Shigella spp.	53.7					53.7													
Staphylococcus aureus	22,100					3,400			773		747		4,510	12,700					
Streptococcus pneumoniae	5,550			59.5	1,670	2,370		137			477	368	470						
All pathogens	159,000	4,610	7,020	21,800	11,700	47,500	2,190	17,600	6,740	6,640	12,800	435	6,750	12,700	6.5	345	94.3	22	75.9

Counts
- **100k – <500k**
- **<100k**
- **NA**

Greece DALYs (count) attributable to bacterial antimicrobial resistance by pathogen−drug combinations, 2019

	Resistance to 1+	AG	Anti−pseudomonal	BL−BLI	CP	FQ	4GC	3GC	Vanco	Aminopenicillin	TMP−SMX	PCN	Macrolide	Methicillin	Mono RIF	MDR excluding XDR in TB	XDR in TB	MDR in S. Typhi and Paratyphi	Mono INH
Acinetobacter baumannii	1,470	166	23.5	0.863	724	548	5.97	6.86											
Citrobacter spp.	69.1	1.39	20.3		21.3	21.1	0.952	4.05											
Enterobacter spp.	524	9.12	168		115	137	27.7				67.5								
Enterococcus faecalis	988					811			177										
Enterococcus faecium	2,240					1,480			760										
Other enterococci	283					186			97										
Escherichia coli	6,720	245		825	272	1,790		1,560		1,130	905								
Group A Streptococcus	74.8												72.3						
Group B Streptococcus	96.8					28						8.63	59.3						
Haemophilus influenzae	139							85		54									
Klebsiella pneumoniae	5,270	669		99.1	2,410	933		752			407								
Morganella spp.	5.05					3.71	0.569	0.768											
Mycobacterium tuberculosis	109														0.147	70.6	19.2		19
Neisseria gonorrhoeae	0.568					0.46		0.108											
Proteus spp.	242	8.57				69		79.9		56.9	27								
Pseudomonas aeruginosa	2,720	111	328		1,370	685	83.7	137											
Salmonella Paratyphi	0.0644					0.064												0.000349	
Salmonella Typhi	15.2					13.7												1.42	
Non−typhoidal Salmonella	0.00319					0.00319													
Serratia spp.	38.1	1.48	8.27		21	3.67	1.67	1.92											
Shigella spp.	1.61					1.59													
Staphylococcus aureus	7,000					751			121		109		826	5,200					
Streptococcus pneumoniae	1,330			14.8	221	59		69.4			338	493	135						
All pathogens	29,300	1,210	548	940	5,150	7,520	121	2,690	1,150	1,240	1,850	501	1,090	5,200	0.147	70.6	19.2	1.32	19

Counts
<100k
NA

Hungary DALYs (count) attributable to bacterial antimicrobial resistance by pathogen−drug combinations, 2019

	Resistance to 1+	AG	Anti−pseudomonal	BL−BLI	CP	FQ	4GC	3GC	Vanco	Aminopenicillin	TMP−SMX	PCN	Macrolide	Methicillin	Mono RIF	MDR excluding XDR in TB	XDR in TB	MDR in S. Typhi and Paratyphi	Mono INH
Acinetobacter baumannii	1,710	116	276	7.33	620	523	112	50.7											
Citrobacter spp.	160	2.45	64.7		23.3	16.5	3.54	49.8											
Enterobacter spp.	708	13.1	262		143	89.5	73.7				127								
Enterococcus faecalis	1,580					1,510			74.5										
Enterococcus faecium	3,750					2,370			1,390										
Other enterococci	355					334			21.2										
Escherichia coli	9,450	644		1,330	247	2,430		2,190		1,170	1,430								
Group A Streptococcus	27.7												27.9						
Group B Streptococcus	347					42.3						10.6	281						
Haemophilus influenzae	23.1							11.1		12									
Klebsiella pneumoniae	3,950	619		288	134	768		1,630			514								
Morganella spp.	12.1					5.65	0.772	5.64											
Mycobacterium tuberculosis	37.6														0.242	18	8.31		10.8
Neisseria gonorrhoeae	2.18					1.75		0.439											
Proteus spp.	408	7.23				101		187		59.5	50.5								
Pseudomonas aeruginosa	2,570	61.4	374		1,460	472	45	160											
Salmonella Paratyphi	0.0781					0.0778												0.000308	
Salmonella Typhi	37					31.3												5.68	
Non−typhoidal Salmonella	0.00437					0.00437													
Serratia spp.	123	9.38	19.6		55	18.7	10.5	10											
Shigella spp.	2.33					2.33													
Staphylococcus aureus	5,140					531			115		89.5		695	3,710					
Streptococcus pneumoniae	1,210			5.15	470	222		20.2			291	115	96.1						
All pathogens	31,600	1,470	997	1,630	3,150	9,470	246	4,320	1,600	1,250	2,500	125	1,110	3,710	0.242	18	8.31	5.83	11.3

Counts
<100k
NA

Iceland DALYs (count) attributable to bacterial antimicrobial resistance by pathogen−drug combinations, 2019

	Resistance to 1+	AG	Anti−pseudomonal	BL−BLI	CP	FQ	4GC	3GC	Vanco	Aminopenicillin	TMP−SMX	PCN	Macrolide	Methicillin	Mono RIF	MDR excluding XDR in TB	XDR in TB	MDR in S. Typhi and Paratyphi	Mono INH
Acinetobacter baumannii	23.3	0.688	8.43	1.56	3.44	1.9	0.397	6.87											
Citrobacter spp.	1.35	0.0277	0.561		0.197	0.173	0.0406	0.351											
Enterobacter spp.	8.3	0.205	4.57		1.86	0.397	0.34				0.912								
Enterococcus faecalis	6.63					5.61			1.02										
Enterococcus faecium	34.2					31.3			2.92										
Other enterococci	1.69					1.24			0.446										
Escherichia coli	97.7	2.76		26.5	4.98	19.9		16.2		9.83	17.5								
Group A Streptococcus	0.799												0.799						
Group B Streptococcus	8.01					4.07						0.23	3.74						
Haemophilus influenzae	1.25							0.212		1.03									
Klebsiella pneumoniae	35.4	3.36		12	1.9	3.21		9.31			5.63								
Morganella spp.	0.125					0.0506	0.0106	0.0641											
Mycobacterium tuberculosis	0.627														0.00104	0.229	0.0626		0.34
Neisseria gonorrhoeae	0.0123					0.0121		0.000222											
Proteus spp.	4.48	0.212				0.521		1.91		0.979	0.807								
Pseudomonas aeruginosa	18.8	0.286	3.74		9.13	4.48	0.908	0.247											
Salmonella Paratyphi	0.0144					0.0143												5.74e−05	
Salmonella Typhi	0.198					0.182												0.0146	
Non−typhoidal Salmonella	0.188					0.188													
Serratia spp.	1.08	0.0361	0.218		0.326	0.0884	0.15	0.257											
Shigella spp.	0.109					0.109													
Staphylococcus aureus	42					6.31			1.9		1.68		5.36	26.7					
Streptococcus pneumoniae	19			0.121	9.09	0.892		0.347			5.89	1.12	1.54						
All pathogens	305	7.58	17.5	40.1	30.9	80.7	1.85	35.7	6.29	11.9	32.5	1.35	11.5	26.7	0.00104	0.229	0.0626	0.0165	0.358

Counts
- <100k
- NA

Ireland DALYs (count) attributable to bacterial antimicrobial resistance by pathogen−drug combinations, 2019

	Resistance to 1+	AG	Anti−pseudomonal	BL−BLI	CP	FQ	4GC	3GC	Vanco	Aminopenicillin	TMP−SMX	PCN	Macrolide	Methicillin	Mono RIF	MDR excluding XDR in TB	XDR in TB	MDR in S. Typhi and Paratyphi	Mono INH
Acinetobacter baumannii	365	5.07	180	34.2	31.9	17.4	26.7	69.6											
Citrobacter spp.	22.9	0.537	10		2.57	2.42	1.1	6.28											
Enterobacter spp.	216	3.17	97.2		19.3	25.7	45.7				24.5								
Enterococcus faecalis	208					189			18.6										
Enterococcus faecium	805					466			339										
Other enterococci	20					12.4			7.58										
Escherichia coli	2,030	100		599	53.2	431		324		231	290								
Group A Streptococcus	16.4												16.7						
Group B Streptococcus	74.2					16.7						2.94	57.3						
Haemophilus influenzae	20.5							4.64		15.9									
Klebsiella pneumoniae	630	50.9		104	34.7	94.4		161			185								
Morganella spp.	2					0.684	0.214	1.11											
Mycobacterium tuberculosis	14.8														0.0903	8.93	2.41		3.41
Neisseria gonorrhoeae	0.235					0.229		0.00638											
Proteus spp.	51.2	3.39				4.44		12.1		22.1	9.23								
Pseudomonas aeruginosa	290	8.84	70.7		121	65.9	13.8	9.53											
Salmonella Paratyphi	0.0144					0.0144												5.59e−05	
Salmonella Typhi	3.31					2.98												0.335	
Non−typhoidal Salmonella	0.0285					0.0285													
Serratia spp.	14	0.406	3.69		2.85	1	0.689	5.36											
Shigella spp.	2.52					2.6													
Staphylococcus aureus	993					119			37.2		33.7		210	593					
Streptococcus pneumoniae	620			0.731	347	35.4		2.45			150	53.2	32.5						
All pathogens	6,400	173	362	738	613	1,490	88.2	596	403	269	692	56.2	314	593	0.0903	8.93	2.41	0.331	3.39

Counts
<100k
NA

Israel DALYs (count) attributable to bacterial antimicrobial resistance by pathogen−drug combinations, 2019

	Resistance to 1+	AG	Anti−pseudomonal	BL−BLI	CP	FQ	4GC	3GC	Vanco	Aminopenicillin	TMP−SMX	PCN	Macrolide	Methicillin	Mono RIF	MDR excluding XDR in TB	XDR in TB	MDR in S. Typhi and Paratyphi	Mono INH
Acinetobacter baumannii	837	72.7	132	4.51	297	293	0.956	36.3											
Citrobacter spp.	75.8	0.89	16.5		7.84	10.2	1.64	38.8											
Enterobacter spp.	275	5.28	71.6		129	27.3	14				28.3								
Enterococcus faecalis	555					508			47.7										
Enterococcus faecium	1,050					771			277										
Other enterococci	124					91.3			32.6										
Escherichia coli	4,490	81.9		858	180	1,090		1,080		268	926								
Group A Streptococcus	30.9												33.3						
Group B Streptococcus	148					44.1						5.25	98.6						
Haemophilus influenzae	38.9							7.3		31.6									
Klebsiella pneumoniae	1,730	143		159	147	235		824			223								
Morganella spp.	7.03					4.08	0.61	2.34											
Mycobacterium tuberculosis	43.9														0.347	29.7	8.11		5.85
Neisseria gonorrhoeae	0.437					0.42		0.0166											
Proteus spp.	223	3.39				50.3		98.5		35.6	34.9								
Pseudomonas aeruginosa	851	7.95	123		472	226	2.33	19.8											
Salmonella Paratyphi	0.0501					0.0499												0.00023	
Salmonella Typhi	11.6					10												1.61	
Non−typhoidal Salmonella	0.784					0.784													
Serratia spp.	31.5	0.872	5.07		5.75	2.31	1.54	15.9											
Shigella spp.	3.91					3.91													
Staphylococcus aureus	3,710					474			50.4		42		386	2,750					
Streptococcus pneumoniae	796			17.7	354	39.3		10.7			188	98.2	89.6						
All pathogens	15,000	316	348	1,040	1,590	3,880	21.1	2,140	408	336	1,440	103	605	2,750	0.347	29.7	8.11	1.6	5.58

Counts
- <100k
- NA

Italy DALYs (count) attributable to bacterial antimicrobial resistance by pathogen−drug combinations, 2019

	Resistance to 1+	AG	Anti−pseudomonal	BL−BLI	CP	FQ	4GC	3GC	Vanco	Aminopenicillin	TMP−SMX	PCN	Macrolide	Methicillin	Mono RIF	MDR excluding XDR in TB	XDR in TB	MDR in S. Typhi and Paratyphi	Mono INH
Acinetobacter baumannii	7,050	764	335	70.2	3,270	2,280	67.1	255											
Citrobacter spp.	615	7.67	227		62.3	76.8	33.2	207											
Enterobacter spp.	2,500	71.5	877		891	298	72.2				292								
Enterococcus faecalis	5,990					5,560			426										
Enterococcus faecium	12,000					8,960			3,030										
Other enterococci	1,070					968			103										
Escherichia coli	41,600	2,720		5,760	795	12,500		11,000		3,700	5,090								
Group A Streptococcus	190												184						
Group B Streptococcus	1,410					191						51.3	1,220						
Haemophilus influenzae	204							40.1		164									
Klebsiella pneumoniae	19,800	2,770		1,070	6,170	3,700		4,210			1,900								
Morganella spp.	54.2					36.4	1.99	15.8											
Mycobacterium tuberculosis	344														11.4	208	56.6		66
Neisseria gonorrhoeae	9.3					7.93		1.37											
Proteus spp.	2,450	103				556		1,300		141	350								
Pseudomonas aeruginosa	9,100	222	1,450		4,340	2,390	240	457											
Salmonella Paratyphi	0.321					0.32												0.00101	
Salmonella Typhi	59.7					54.2												5.52	
Non−typhoidal Salmonella	0.211					0.211													
Serratia spp.	189	13.6	41.9		51.9	25.8	17.9	38.5											
Shigella spp.	19.8					19.8													
Staphylococcus aureus	28,500					3,940			488		355		3,360	20,300					
Streptococcus pneumoniae	3,720			20.7	1,680	435		75.5			370	444	666						
All pathogens	137,000	6,670	2,930	6,920	17,300	42,000	433	17,600	4,050	4,000	8,360	495	5,420	20,300	11.4	208	56.6	5.21	69.3

Counts
100k – <500k
<100k
NA

Kazakhstan DALYs (count) attributable to bacterial antimicrobial resistance by pathogen−drug combinations, 2019

	Resistance to 1+	AG	Anti−pseudomonal	BL−BLI	CP	FQ	4GC	3GC	Vanco	Aminopenicillin	TMP−SMX	PCN	Macrolide	Methicillin	Mono RIF	MDR excluding XDR in TB	XDR in TB	MDR in S. Typhi and Paratyphi	Mono INH
Acinetobacter baumannii	6,270	674	749	2.08	2,510	2,240	5.38	89.6											
Citrobacter spp.	421	8.59	62.2		114	65.9	21.3	149											
Enterobacter spp.	2,360	114	978		327	285	366				288								
Enterococcus faecalis	2,350					2,170			182										
Enterococcus faecium	4,490					3,610			885										
Other enterococci	1,130					1,010			120										
Escherichia coli	11,300	935		2,740	1,660	1,190		1,110		1,200	2,450								
Group A Streptococcus	89.1												90.3						
Group B Streptococcus	876					212						51	614						
Haemophilus influenzae	157							54.7		102									
Klebsiella pneumoniae	12,300	1,440		702	872	2,180		5,210			1,850								
Morganella spp.	31					14.5	4.21	12.3											
Mycobacterium tuberculosis	9,170														314	5,460	2,650		736
Neisseria gonorrhoeae	13.2					12.7		0.467											
Proteus spp.	662	57.1				120		288		91.3	99.6								
Pseudomonas aeruginosa	7,380	237	1,440		3,050	1,940	257	457											
Salmonella Paratyphi	6.83					6.8												0.0315	
Salmonella Typhi	163					138												26	
Non−typhoidal Salmonella	4.7					4.7													
Serratia spp.	898	72.1	206		142	54.7	296	126											
Shigella spp.	31.7					31.7													
Staphylococcus aureus	8,980					1,040			227		213		924	6,570					
Streptococcus pneumoniae	6,100			27.8	2,300	154		91.9			2,750	550	226						
All pathogens	75,200	3,540	3,440	3,470	11,000	16,500	951	7,580	1,410	1,400	7,660	601	1,860	6,570	314	5,460	2,650	23.8	759

Counts
<100k
NA

Kyrgyzstan DALYs (count) attributable to bacterial antimicrobial resistance by pathogen−drug combinations, 2019

	Resistance to 1+	AG	Anti−pseudomonal	BL−BLI	CP	FQ	4GC	3GC	Vanco	Aminopenicillin	TMP−SMX	PCN	Macrolide	Methicillin	Mono RIF	MDR excluding XDR in TB	XDR in TB	MDR in S. Typhi and Paratyphi	Mono INH
Acinetobacter baumannii	1,590	165	225	0.464	600	580	0.789	13.9											
Citrobacter spp.	145	5.02	14.4		33.2	40.6	12.5	39											
Enterobacter spp.	724	37.8	302		118	53.4	100				111								
Enterococcus faecalis	686					623			63.4										
Enterococcus faecium	1,130					925			204										
Other enterococci	313					301			12.9										
Escherichia coli	4,480	219		518	485	1,030		1,370		170	696								
Group A Streptococcus	22.8												22						
Group B Streptococcus	369					122						24.9	223						
Haemophilus influenzae	81.9							50.3		31.6									
Klebsiella pneumoniae	4,160	521		166	453	536		1,770			720								
Morganella spp.	10.7					5.61	2.31	2.83											
Mycobacterium tuberculosis	5,720														446	3,220	1,580		478
Neisseria gonorrhoeae	5.92					5.18		0.739											
Proteus spp.	220	17.1				29.2		113		26.6	34								
Pseudomonas aeruginosa	2,260	89.9	447		788	623	91.6	215											
Salmonella Paratyphi	0.148					0.147												0.000691	
Salmonella Typhi	84.2					77.3												6.81	
Non−typhoidal Salmonella	2.06					2.06													
Serratia spp.	262	16.8	68.3		54.4	19.5	65.9	35.5											
Shigella spp.	35.9					35.9													
Staphylococcus aureus	3,910					314			56.8		219		326	3,000					
Streptococcus pneumoniae	2,490			25.5	749	57.8		47.1			1,200	332	78.5						
All pathogens	28,700	1,070	1,060	710	3,280	5,380	273	3,650	337	228	2,980	357	647	3,000	446	3,220	1,580	6.95	485

Counts
<100k
NA

Latvia DALYs (count) attributable to bacterial antimicrobial resistance by pathogen−drug combinations, 2019

	Resistance to 1+	AG	Anti−pseudomonal	BL−BLI	CP	FQ	4GC	3GC	Vanco	Aminopenicillin	TMP−SMX	PCN	Macrolide	Methicillin	Mono RIF	MDR excluding XDR in TB	XDR in TB	MDR in S. Typhi and Paratyphi	Mono INH
Acinetobacter baumannii	434	37	46.8	0.864	187	151	0.122	10.8											
Citrobacter spp.	30.9	0.41	10.5		10.2	3.53	0.585	5.7											
Enterobacter spp.	95.4	2.87	20.8		18.8	20	8.61				24.3								
Enterococcus faecalis	410					347			62										
Enterococcus faecium	586					374			211										
Other enterococci	75					69.5			5.52										
Escherichia coli	1,650	59.2		217	68.9	366		443		224	274								
Group A Streptococcus	11.9												11.3						
Group B Streptococcus	75.1					7.85						2.08	64.6						
Haemophilus influenzae	3.95							2.6		1.34									
Klebsiella pneumoniae	825	96.7		81.1	38.7	153		305			150								
Morganella spp.	3.85					0.438	0.379	3.03											
Mycobacterium tuberculosis	248														0.191	143	68.3		37.4
Neisseria gonorrhoeae	0.425					0.406		0.0191											
Proteus spp.	82.6	3.38				19		34.2		15.8	10.6								
Pseudomonas aeruginosa	562	19.7	115		186	156	36	48.8											
Salmonella Paratyphi	0.0185					0.0184												7.34e−05	
Salmonella Typhi	8.76					7.55												1.11	
Non−typhoidal Salmonella	1.18					1.18													
Serratia spp.	28.4	2.35	4.63		10.7	1.18	2.54	7.1											
Shigella spp.	1.72					1.72													
Staphylococcus aureus	382					22.4			23.9		24.2		99.1	212					
Streptococcus pneumoniae	295			2.6	103	30.2		3.3			123	19.1	13.3						
All pathogens	5,810	222	198	302	623	1,730	48.2	864	303	241	607	21.2	188	212	0.191	143	68.3	1.22	36.5

Counts: <100k, NA

Lithuania DALYs (count) attributable to bacterial antimicrobial resistance by pathogen−drug combinations, 2019

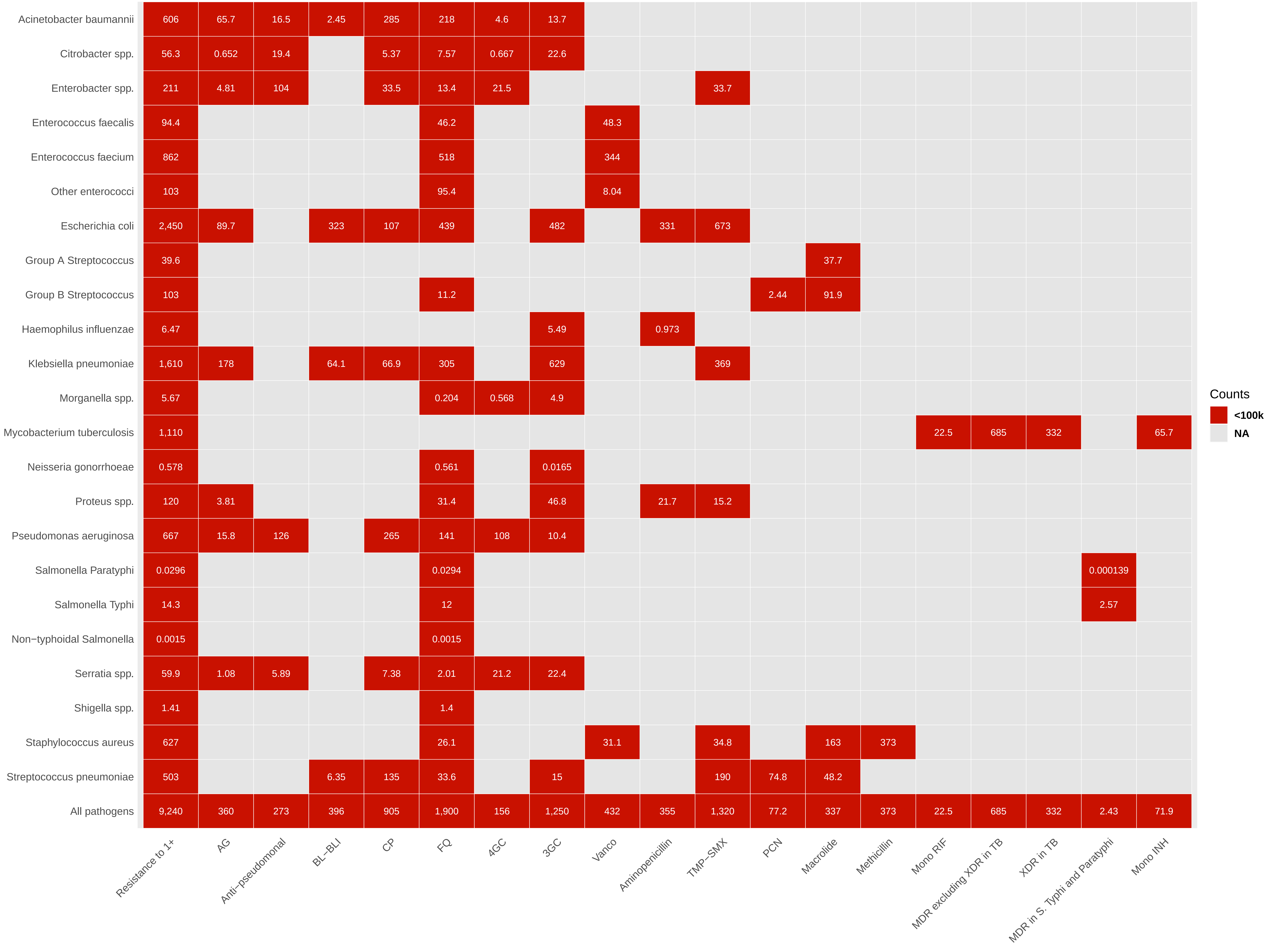

Luxembourg DALYs (count) attributable to bacterial antimicrobial resistance by pathogen−drug combinations, 2019

	Resistance to 1+	AG	Anti−pseudomonal	BL−BLI	CP	FQ	4GC	3GC	Vanco	Aminopenicillin	TMP−SMX	PCN	Macrolide	Methicillin	Mono RIF	MDR excluding XDR in TB	XDR in TB	MDR in S. Typhi and Paratyphi	Mono INH
Acinetobacter baumannii	48.9	0.545	28	2.4	2.17	4.18	1.62	9.97											
Citrobacter spp.	4.13	0.0773	1.81		0.458	0.439	0.127	1.23											
Enterobacter spp.	17.6	0.361	9.08		4.37	1.82	0.659				1.34								
Enterococcus faecalis	24.1					19.7			4.41										
Enterococcus faecium	93.2					88.4			4.88										
Other enterococci	4.88					4			0.882										
Escherichia coli	263	10.1		62.4	8.56	63.7		44.1		33.8	40.5								
Group A Streptococcus	1.95												1.93						
Group B Streptococcus	13.3					5.51						0.485	7.18						
Haemophilus influenzae	2.27							0.489		1.78									
Klebsiella pneumoniae	93	10.9		12.5	5.45	18.1		40.3			5.74								
Morganella spp.	0.188					0.0924	0.0166	0.0788											
Mycobacterium tuberculosis	1.4														0.00106	0.897	0.243		0.247
Neisseria gonorrhoeae	0.0564					0.0481		0.00824											
Proteus spp.	10.7	0.521				1.93		4.21		2.33	1.54								
Pseudomonas aeruginosa	52.6	0.996	13.2		16.1	17.7	1.09	3.53											
Salmonella Paratyphi	0.0297					0.0296												9.92e−05	
Salmonella Typhi	0.933					0.873												0.0587	
Non−typhoidal Salmonella	0.719					0.719													
Serratia spp.	2.8	0.0677	0.515		0.651	0.223	0.176	1.16											
Shigella spp.	0.318					0.318													
Staphylococcus aureus	118					13.8			4.57		3.35		24.5	71.8					
Streptococcus pneumoniae	32.6			1.07	13.9	2.08		0.743			8.19	3.82	2.85						
All pathogens	787	23.5	52.6	78.4	51.7	244	3.69	106	14.7	38	60.7	4.3	36.6	71.8	0.00106	0.897	0.243	0.0635	0.262

Counts
- <100k
- NA

Malta DALYs (count) attributable to bacterial antimicrobial resistance by pathogen−drug combinations, 2019

	Resistance to 1+	AG	Anti−pseudomonal	BL−BLI	CP	FQ	4GC	3GC	Vanco	Aminopenicillin	TMP−SMX	PCN	Macrolide	Methicillin	Mono RIF	MDR excluding XDR in TB	XDR in TB	MDR in S. Typhi and Paratyphi	Mono INH
Acinetobacter baumannii	82.5	2.1	49	1.95	5.94	4.95	2.03	16.6											
Citrobacter spp.	6.02	0.09	2.18		0.319	0.926	0.263	2.24											
Enterobacter spp.	20.3	0.478	8.44		6.35	2.38	1.01				1.65								
Enterococcus faecalis	19.4					16.2			3.24										
Enterococcus faecium	58.1					54.3			3.84										
Other enterococci	8.03					5.57			2.46										
Escherichia coli	208	13.3		30.1	2.25	77.6		33.7		27.6	23.2								
Group A Streptococcus	3.57												3.45						
Group B Streptococcus	9.1					2.38						0.688	6.14						
Haemophilus influenzae	3.82							0.768		3.05									
Klebsiella pneumoniae	128	20.5		4.08	39.8	22.2		31.7			9.8								
Morganella spp.	0.321					0.168	0.0449	0.109											
Mycobacterium tuberculosis	0.474														0.000292	0.195	0.052		0.245
Neisseria gonorrhoeae	0.0246					0.0243		0.000288											
Proteus spp.	9.8	0.381				1.59		5.06		1.39	1.4								
Pseudomonas aeruginosa	52.7	0.871	25.9		11.5	8.56	3.21	2.59											
Salmonella Paratyphi	0.00439					0.00437												1.52e−05	
Salmonella Typhi	0.423					0.359												0.0628	
Non−typhoidal Salmonella	0.000139					0.000139													
Serratia spp.	1.97	0.0762	0.437		0.517	0.284	0.0782	0.577											
Shigella spp.	0.0983					0.103													
Staphylococcus aureus	190					19.1			3.13		3.7		25.6	139					
Streptococcus pneumoniae	67.7			2.61	28	2.34		0.536			11.6	12.7	10.1						
All pathogens	871	37.8	85.9	38.8	94.6	219	6.63	93.9	12.7	32	51.4	13.4	45.1	139	0.000292	0.195	0.052	0.0611	0.223

Counts
<100k
NA

Monaco DALYs (count) attributable to bacterial antimicrobial resistance by pathogen−drug combinations, 2019

	Resistance to 1+	AG	Anti−pseudomonal	BL−BLI	CP	FQ	4GC	3GC	Vanco	Aminopenicillin	TMP−SMX	PCN	Macrolide	Methicillin	Mono RIF	MDR excluding XDR in TB	XDR in TB	MDR in S. Typhi and Paratyphi	Mono INH
Acinetobacter baumannii	6.33	0.742	1.56	0.102	2.13	1.16	0.0813	0.546											
Citrobacter spp.	0.515	0.00726	0.184		0.0665	0.0749	0.0261	0.156											
Enterobacter spp.	1.89	0.044	0.83		0.637	0.143	0.0722				0.158								
Enterococcus faecalis	2.09					1.8			0.295										
Enterococcus faecium	10.9					7.93			2.95										
Other enterococci	0.443					0.32			0.123										
Escherichia coli	32	1.1		7.18	0.615	8.3		7.38		2.36	5.1								
Group A Streptococcus	0.268												0.247						
Group B Streptococcus	2.33					1.61						0.0653	0.623						
Haemophilus influenzae	0.311							0.0653		0.245									
Klebsiella pneumoniae	8.27	1.09		2.81	0.502	0.9		1.61			1.35								
Morganella spp.	0.0198					0.00887	0.00235	0.00854											
Mycobacterium tuberculosis	0.393														0.00231	0.218	0.058		0.118
Neisseria gonorrhoeae	0.00175					0.00169		6e−05											
Proteus spp.	1.2	0.0456				0.164		0.554		0.17	0.26								
Pseudomonas aeruginosa	6.46	0.14	1.47		2.81	1.37	0.129	0.537											
Salmonella Paratyphi	0.000165					0.000164												5.4e−07	
Salmonella Typhi	0.0877					0.0769												0.011	
Non−typhoidal Salmonella	0.0221					0.0221													
Serratia spp.	0.275	0.00977	0.0601		0.0584	0.0252	0.0236	0.0978											
Shigella spp.	0.0107					0.0101													
Staphylococcus aureus	16.6					2.9			0.525		0.36		3.28	9.51					
Streptococcus pneumoniae	5.51			0.0896	1.87	0.783		0.0829			1.36	0.316	0.982						
All pathogens	95.9	3.18	4.11	10.2	8.69	27.6	0.335	11	3.9	2.77	8.6	0.382	5.19	9.51	0.00231	0.218	0.058	0.0108	0.11

Counts
<100k
NA

Montenegro DALYs (count) attributable to bacterial antimicrobial resistance by pathogen−drug combinations, 2019

	Resistance to 1+	AG	Anti−pseudomonal	BL−BLI	CP	FQ	4GC	3GC	Vanco	Aminopenicillin	TMP−SMX	PCN	Macrolide	Methicillin	Mono RIF	MDR excluding XDR in TB	XDR in TB	MDR in S. Typhi and Paratyphi	Mono INH
Acinetobacter baumannii	250	23.7	34.3	0.0558	99.6	89.3	0.836	2.57											
Citrobacter spp.	24.9	0.308	6.73		3.48	5.66	0.26	8.44											
Enterobacter spp.	69.8	2.58	22.3		12.6	11.1	9.29				12.1								
Enterococcus faecalis	60.3					56.5			3.76										
Enterococcus faecium	131					89.1			41.6										
Other enterococci	34.4					28.8			5.62										
Escherichia coli	393	21.7		29.4	26.5	68.7		172		17.2	56.9								
Group A Streptococcus	3.67												3.53						
Group B Streptococcus	19.3					10.9						0.479	8.44						
Haemophilus influenzae	1.98							0.666		1.32									
Klebsiella pneumoniae	259	27.5		1.38	20.4	42.1		139			28.4								
Morganella spp.	0.508					0.241	0.0504	0.216											
Mycobacterium tuberculosis	2.07														0.0144	1.32	0.619		0.106
Neisseria gonorrhoeae	0.172					0.164		0.00766											
Proteus spp.	23.6	0.699				2.92		14.5		1.97	3.47								
Pseudomonas aeruginosa	125	3.83	14.8		60.3	36.8	4.67	4.15											
Salmonella Paratyphi	0.00411					0.00409												1.76e−05	
Salmonella Typhi	1.46					1.35												0.102	
Non−typhoidal Salmonella	0.195					0.195													
Serratia spp.	10.6	0.671	1.62		2.53	1	2.68	2.06											
Shigella spp.	0.053					0.051													
Staphylococcus aureus	223					19.5			4.01		3.58		20.3	176					
Streptococcus pneumoniae	95.9			0.952	42.1	3.26		1.28			22.6	14.9	11						
All pathogens	1,730	81	79.8	31.8	267	468	17.8	345	55	20.5	127	15.4	42.6	176	0.0144	1.32	0.619	0.0999	0.112

Counts
<100k
NA

Netherlands DALYs (count) attributable to bacterial antimicrobial resistance by pathogen−drug combinations, 2019

	Resistance to 1+	AG	Anti−pseudomonal	BL−BLI	CP	FQ	4GC	3GC	Vanco	Aminopenicillin	TMP−SMX	PCN	Macrolide	Methicillin	Mono RIF	MDR excluding XDR in TB	XDR in TB	MDR in S. Typhi and Paratyphi	Mono INH
Acinetobacter baumannii	1,720	33.6	860	107	128	113	48.6	433											
Citrobacter spp.	60.8	2.56	15.2		14.7	19.4	4.02	4.91											
Enterobacter spp.	651	18.7	244		239	26.7	62.8				60.6								
Enterococcus faecalis	743					682			60.8										
Enterococcus faecium	3,000					2,920			81.1										
Other enterococci	195					158			37.6										
Escherichia coli	8,310	309		2,330	215	1,560		990		988	1,920								
Group A Streptococcus	41.7												45.8						
Group B Streptococcus	422					62.1						22.3	313						
Haemophilus influenzae	32.8							23.3		9.46									
Klebsiella pneumoniae	1,890	165		37.3	78.4	365		598			642								
Morganella spp.	15.2					4.64	1.86	8.65											
Mycobacterium tuberculosis	60.9														0.41	34.3	9.23		17.3
Neisseria gonorrhoeae	0.546					0.525		0.0215											
Proteus spp.	279	9.88				13.6		71.9		89.2	96.2								
Pseudomonas aeruginosa	925	17.8	253		279	316	32.3	27											
Salmonella Paratyphi	0.168					0.167												0.000527	
Salmonella Typhi	13.9					12.2												1.72	
Non−typhoidal Salmonella	29.7					29.7													
Serratia spp.	72.2	5.15	9.16		23.2	7.36	2.12	25.4											
Shigella spp.	6.17					6.17													
Staphylococcus aureus	2,310					234			113		176		836	953					
Streptococcus pneumoniae	974			22.2	164	395		29			284	36.1	43.3						
All pathogens	21,700	562	1,380	2,500	1,140	6,920	152	2,210	292	1,080	3,180	58.4	1,250	953	0.41	34.3	9.23	1.73	16.5

Counts
<100k
NA

North Macedonia DALYs (count) attributable to bacterial antimicrobial resistance by pathogen−drug combinations, 2019

	Resistance to 1+	AG	Anti−pseudomonal	BL−BLI	CP	FQ	4GC	3GC	Vanco	Aminopenicillin	TMP−SMX	PCN	Macrolide	Methicillin	Mono RIF	MDR excluding XDR in TB	XDR in TB	MDR in S. Typhi and Paratyphi	Mono INH
Acinetobacter baumannii	1,000	105	68.8	0.217	447	355	1.34	23											
Citrobacter spp.	98.7	1.19	29.7		17.8	17.5	2.63	30											
Enterobacter spp.	320	9.97	87.5		36.8	106	44.7				35.3								
Enterococcus faecalis	209					192			17										
Enterococcus faecium	470					341			129										
Other enterococci	130					109			20.9										
Escherichia coli	2,040	86.8		42.2	591	528		440		31.1	322								
Group A Streptococcus	12.7												14.3						
Group B Streptococcus	59.4					18.5						2.74	38						
Haemophilus influenzae	9.66							3		6.66									
Klebsiella pneumoniae	1,170	132		5.08	247	229		408			145								
Morganella spp.	2.99					1.09	0.506	1.39											
Mycobacterium tuberculosis	23.5														0.126	15.3	7.16		0.96
Neisseria gonorrhoeae	0.587					0.56		0.027											
Proteus spp.	82.3	2.62				17.9		40.8		8.89	12								
Pseudomonas aeruginosa	570	24	84.7		247	147	25.9	40.9											
Salmonella Paratyphi	0.013					0.013												4.91e−05	
Salmonella Typhi	7.82					7.27												0.548	
Non−typhoidal Salmonella	0.657					0.657													
Serratia spp.	70.2	4.07	11.5		11.3	3.94	34.3	5.29											
Shigella spp.	0.871					0.837													
Staphylococcus aureus	1,100					67.2			12.3		10.5		58.8	952					
Streptococcus pneumoniae	445			1.69	203	12.7		9.2			114	69.8	33.4						
All pathogens	7,820	365	282	49.2	1,800	2,160	109	1,000	179	46.7	640	72.5	144	952	0.126	15.3	7.16	0.548	0.932

Counts
<100k
NA

Norway DALYs (count) attributable to bacterial antimicrobial resistance by pathogen−drug combinations, 2019

	Resistance to 1+	AG	Anti−pseudomonal	BL−BLI	CP	FQ	4GC	3GC	Vanco	Aminopenicillin	TMP−SMX	PCN	Macrolide	Methicillin	Mono RIF	MDR excluding XDR in TB	XDR in TB	MDR in S. Typhi and Paratyphi	Mono INH
Acinetobacter baumannii	417	25.7	81.5	32.5	119	17.4	22.4	119											
Citrobacter spp.	21.6	0.555	7.7		3.15	2.84	0.778	6.6											
Enterobacter spp.	141	4.42	69.2		34.9	6.66	7.83				17.6								
Enterococcus faecalis	170					150			19.9										
Enterococcus faecium	649					608			40.7										
Other enterococci	27.1					16.1			11										
Escherichia coli	2,630	95.6		428	1,240	317		135		126	287								
Group A Streptococcus	16.1												17						
Group B Streptococcus	105					15.3						6.1	79.5						
Haemophilus influenzae	25							3.5		21.5									
Klebsiella pneumoniae	568	49.8		149	21.5	154		105			89.2								
Morganella spp.	3.55					0.986	0.504	2.05											
Mycobacterium tuberculosis	27.5														0.0301	16.5	4.52		6.43
Neisseria gonorrhoeae	0.779					0.734		0.0443											
Proteus spp.	77.5	4.48				5.53		21.2		28.3	18.2								
Pseudomonas aeruginosa	274	5.97	54.2		136	53.4	17.4	6.57											
Salmonella Paratyphi	1					0.996												0.00325	
Salmonella Typhi	3.56					3.26												0.317	
Non−typhoidal Salmonella	1.41					1.41													
Serratia spp.	17.6	0.783	3.89		6.85	1.15	0.533	4.39											
Shigella spp.	4.12					4.12													
Staphylococcus aureus	1,000					57.7			40		36.8		54.8	811					
Streptococcus pneumoniae	217			3.44	75.2	20.9		10.4			70.6	22.4	14.1						
All pathogens	6,400	187	217	613	1,640	1,440	49.5	413	112	175	519	28.4	169	811	0.0301	16.5	4.52	0.333	6.07

Counts
- <100k
- NA

Poland DALYs (count) attributable to bacterial antimicrobial resistance by pathogen−drug combinations, 2019

	Resistance to 1+	AG	Anti−pseudomonal	BL−BLI	CP	FQ	4GC	3GC	Vanco	Aminopenicillin	TMP−SMX	PCN	Macrolide	Methicillin	Mono RIF	MDR excluding XDR in TB	XDR in TB	MDR in S. Typhi and Paratyphi	Mono INH
Acinetobacter baumannii	8,190	696	697	0.809	3,390	3,120	46.9	240											
Citrobacter spp.	609	7.88	303		88.8	169	26.4	13.4											
Enterobacter spp.	4,990	443	2,960		458	404	178				552								
Enterococcus faecalis	6,110					5,650			459										
Enterococcus faecium	10,500					6,950			3,540										
Other enterococci	1,280					1,140			137										
Escherichia coli	27,400	1,200		5,600	655	7,870		4,700		2,790	4,540								
Group A Streptococcus	73.3												71.9						
Group B Streptococcus	1,130					128						36	1,070						
Haemophilus influenzae	125							62.4		62.7									
Klebsiella pneumoniae	19,300	2,670		1,340	2,370	3,790		6,330			2,810								
Morganella spp.	38.6					13.3	3.66	21.7											
Mycobacterium tuberculosis	298														14.8	135	63.6		84.1
Neisseria gonorrhoeae	10.2					9.82		0.406											
Proteus spp.	1,260	28				250		505		280	187								
Pseudomonas aeruginosa	9,960	365	1,570		4,490	2,750	323	455											
Salmonella Paratyphi	0.245					0.244												0.00098	
Salmonella Typhi	126					109												17.2	
Non−typhoidal Salmonella	4.01					4.01													
Serratia spp.	140	14.3	32.4		49	20.6	11.9	11.8											
Shigella spp.	3.29					3.47													
Staphylococcus aureus	14,300					1,130			219		587		2,720	9,610					
Streptococcus pneumoniae	8,740			35.3	4,000	204		129			2,810	627	926						
All pathogens	115,000	5,420	5,570	6,980	15,500	33,700	590	12,500	4,350	3,150	11,500	663	4,770	9,610	14.8	135	63.6	16.1	81.7

Counts
- 100k – <500k
- <100k
- NA

Portugal DALYs (count) attributable to bacterial antimicrobial resistance by pathogen−drug combinations, 2019

	Resistance to 1+	AG	Anti−pseudomonal	BL−BLI	CP	FQ	4GC	3GC	Vanco	Aminopenicillin	TMP−SMX	PCN	Macrolide	Methicillin	Mono RIF	MDR excluding XDR in TB	XDR in TB	MDR in S. Typhi and Paratyphi	Mono INH
Acinetobacter baumannii	1,560	78.1	478	27	511	328	20.4	118											
Citrobacter spp.	150	2.04	68.1		13.7	10.8	2.8	52.7											
Enterobacter spp.	729	11.9	319		294	42.9	20.2				41.7								
Enterococcus faecalis	1,190					1,100			87.3										
Enterococcus faecium	2,410					2,040			371										
Other enterococci	161					113			47.4										
Escherichia coli	8,270	503		1,440	235	1,910		1,530		1,050	1,600								
Group A Streptococcus	35.4												37.1						
Group B Streptococcus	379					38.7						11.1	336						
Haemophilus influenzae	82							20.6		61.4									
Klebsiella pneumoniae	4,830	648		304	947	903		1,480			553								
Morganella spp.	20.2					7.44	5.2	7.58											
Mycobacterium tuberculosis	145														1.73	77	20.9		44.2
Neisseria gonorrhoeae	0.319					0.313		0.00681											
Proteus spp.	298	10.3				22.1		141		46.2	77.2								
Pseudomonas aeruginosa	2,510	64.9	732		877	571	50.7	219											
Salmonella Paratyphi	0.293					0.292												0.000921	
Salmonella Typhi	15.7					13.9												1.74	
Non−typhoidal Salmonella	0.00319					0.00319													
Serratia spp.	59.1	1.79	14		11.5	7.01	4.04	20.8											
Shigella spp.	3.41					3.41													
Staphylococcus aureus	8,540					1,030			141		116		1,110	6,140					
Streptococcus pneumoniae	1,870			198	907	54.1		48.5			351	166	159						
All pathogens	33,300	1,320	1,610	1,960	3,800	8,200	103	3,640	647	1,150	2,740	177	1,630	6,140	1.73	77	20.9	1.76	45.3

Counts
<100k
NA

Republic of Moldova DALYs (count) attributable to bacterial antimicrobial resistance by pathogen−drug combinations, 2019

	Resistance to 1+	AG	Anti−pseudomonal	BL−BLI	CP	FQ	4GC	3GC	Vanco	Aminopenicillin	TMP−SMX	PCN	Macrolide	Methicillin	Mono RIF	MDR excluding XDR in TB	XDR in TB	MDR in S. Typhi and Paratyphi	Mono INH
Acinetobacter baumannii	812	87.3	45.7	0.119	364	303	3	7.77											
Citrobacter spp.	93.4	2.31	19.3		14.7	16.5	3.67	37											
Enterobacter spp.	474	17.3	164		80.6	57.5	97.5				58.6								
Enterococcus faecalis	905					835			70.7										
Enterococcus faecium	1,430					1,070			352										
Other enterococci	242					210			32.3										
Escherichia coli	4,790	235		512	359	1,080		1,580		216	801								
Group A Streptococcus	18.5												17.5						
Group B Streptococcus	124					19.1						9.56	91.1						
Haemophilus influenzae	24.9							10.2		14.7									
Klebsiella pneumoniae	2,970	435		141	188	475		1,320			415								
Morganella spp.	7.02					1.56	0.616	4.84											
Mycobacterium tuberculosis	2,640														42.5	1,680	842		76.8
Neisseria gonorrhoeae	1.03					1.01		0.027											
Proteus spp.	176	20.7				42.6		59.7		30.1	23								
Pseudomonas aeruginosa	1,540	56.7	188		704	400	93.4	99.6											
Salmonella Paratyphi	0.107					0.106												0.000332	
Salmonella Typhi	46					39												6.79	
Non−typhoidal Salmonella	4.07					4.07													
Serratia spp.	148	13.2	36.3		23.4	6	48.4	21.3											
Shigella spp.	2.7					2.58													
Staphylococcus aureus	2,870					136			52.8		35.1		186	2,460					
Streptococcus pneumoniae	1,060			10.7	309	25.5		54.5			566	34.2	63.1						
All pathogens	20,400	867	453	664	2,040	4,730	247	3,190	508	261	1,900	43.7	364	2,460	42.5	1,680	842	7.13	77.1

Counts
<100k
NA

Romania DALYs (count) attributable to bacterial antimicrobial resistance by pathogen−drug combinations, 2019

	Resistance to 1+	AG	Anti−pseudomonal	BL−BLI	CP	FQ	4GC	3GC	Vanco	Aminopenicillin	TMP−SMX	PCN	Macrolide	Methicillin	Mono RIF	MDR excluding XDR in TB	XDR in TB	MDR in S. Typhi and Paratyphi	Mono INH
Acinetobacter baumannii	4,220	415	150	0.175	2,050	1,510	54.8	29.1											
Citrobacter spp.	492	5.89	299		62.4	34.7	9.58	80.6											
Enterobacter spp.	1,320	50.5	72.9		326	549	52.8				262								
Enterococcus faecalis	4,190					3,900			291										
Enterococcus faecium	7,480					4,160			3,320										
Other enterococci	959					873			85.3										
Escherichia coli	19,100	1,150		3,650	443	4,410		4,860		2,840	1,760								
Group A Streptococcus	205												187						
Group B Streptococcus	736					93.8						18.1	599						
Haemophilus influenzae	358							301		57.7									
Klebsiella pneumoniae	16,200	3,110		803	6,130	2,500		2,250			1,450								
Morganella spp.	29.3					12.8	6.04	10.4											
Mycobacterium tuberculosis	1,990														125	1,130	535		211
Neisseria gonorrhoeae	4.86					4.23		0.632											
Proteus spp.	1,100	19.8				263		634		71.7	109								
Pseudomonas aeruginosa	9,490	562	1,220		4,700	2,610	178	231											
Salmonella Paratyphi	0.354					0.353												0.00144	
Salmonella Typhi	113					96.8												16.6	
Non−typhoidal Salmonella	0.0303					0.0303													
Serratia spp.	953	44.3	140		146	20.8	600	1.16											
Shigella spp.	0.0654					0.0645													
Staphylococcus aureus	18,000					593			220		194		2,410	14,500					
Streptococcus pneumoniae	8,190			7.61	4,180	211		572			876	1,520	874						
All pathogens	95,100	5,350	1,880	4,460	18,000	21,900	901	8,970	3,910	2,970	4,660	1,540	4,050	14,500	125	1,130	535	17.3	197

Counts
<100k
NA

Russian Federation DALYs (count) attributable to bacterial antimicrobial resistance by pathogen−drug combinations, 2019

	Resistance to 1+	AG	Anti−pseudomonal	BL−BLI	CP	FQ	4GC	3GC	Vanco	Aminopenicillin	TMP−SMX	PCN	Macrolide	Methicillin	Mono RIF	MDR excluding XDR in TB	XDR in TB	MDR in S. Typhi and Paratyphi	Mono INH
Acinetobacter baumannii	28,200	3,250	1,610	12.6	12,400	10,500	41.6	428											
Citrobacter spp.	2,020	43.5	1,020		209	340	49.9	357											
Enterobacter spp.	20,100	573	10,100		3,830	651	2,790				2,160								
Enterococcus faecalis	30,900					28,600			2,330										
Enterococcus faecium	44,300					39,800			4,510										
Other enterococci	8,370					7,140			1,230										
Escherichia coli	198,000	12,300		16,500	16,600	45,200		75,200		5,970	26,500								
Group A Streptococcus	620												598						
Group B Streptococcus	7,510					1,020						373	6,580						
Haemophilus influenzae	668							434		233									
Klebsiella pneumoniae	143,000	20,700		2,370	67,800	23,300		14,100			14,900								
Morganella spp.	304					51.2	26.6	226											
Mycobacterium tuberculosis	86,000														4,370	51,300	25,100		5,240
Neisseria gonorrhoeae	36.5					35.2		1.31											
Proteus spp.	5,140	1,670				741		632		1,270	823								
Pseudomonas aeruginosa	71,800	3,740	7,520		35,100	20,600	1,370	3,480											
Salmonella Paratyphi	5.52					5.5												0.0197	
Salmonella Typhi	1,500					1,240												253	
Non−typhoidal Salmonella	118					118													
Serratia spp.	6,210	908	1,810		1,750	87.4	1,310	351											
Shigella spp.	173					161													
Staphylococcus aureus	77,700					9,660			1,850		1,380		4,910	59,900					
Streptococcus pneumoniae	48,600			219	7,370	1,380		8,060			25,500	4,860	1,200						
All pathogens	782,000	43,200	22,100	19,100	145,000	191,000	5,590	103,000	9,920	7,470	71,300	5,230	13,100	59,900	4,370	51,300	25,100	268	4,990

Counts
- **500k − <1M**
- **100k − <500k**
- **<100k**
- **NA**

San Marino DALYs (count) attributable to bacterial antimicrobial resistance by pathogen−drug combinations, 2019

	Resistance to 1+	AG	Anti−pseudomonal	BL−BLI	CP	FQ	4GC	3GC	Vanco	Aminopenicillin	TMP−SMX	PCN	Macrolide	Methicillin	Mono RIF	MDR excluding XDR in TB	XDR in TB	MDR in S. Typhi and Paratyphi	Mono INH
Acinetobacter baumannii	4.75	0.49	1.38	0.0913	1.33	0.933	0.0291	0.484											
Citrobacter spp.	0.439	0.00569	0.133		0.106	0.0678	0.00902	0.118											
Enterobacter spp.	1.28	0.0284	0.551		0.409	0.123	0.0582				0.113								
Enterococcus faecalis	1.2					1.01			0.188										
Enterococcus faecium	6					4.85			1.15										
Other enterococci	0.315					0.245			0.0699										
Escherichia coli	17.6	0.587		3.77	0.347	4.72		4.23		1.13	2.82								
Group A Streptococcus	0.149												0.151						
Group B Streptococcus	1.02					0.571						0.0375	0.391						
Haemophilus influenzae	0.166							0.048		0.118									
Klebsiella pneumoniae	5.11	0.602		1.72	0.335	0.416		1.19			0.854								
Morganella spp.	0.0169					0.00678	0.00192	0.0082											
Mycobacterium tuberculosis	0.0832														0.000404	0.0434	0.0118		0.0268
Neisseria gonorrhoeae	0.00195					0.00188		6.37e−05											
Proteus spp.	0.737	0.0283				0.0978		0.34		0.118	0.153								
Pseudomonas aeruginosa	3.42	0.0666	0.869		1.5	0.718	0.0565	0.204											
Salmonella Paratyphi	0.00015					0.000149												5e−07	
Salmonella Typhi	0.0415					0.0368												0.00483	
Non−typhoidal Salmonella	0.0194					0.0194													
Serratia spp.	0.182	0.00505	0.0451		0.0415	0.017	0.0275	0.0462											
Shigella spp.	0.00839					0.00779													
Staphylococcus aureus	11.7					1.41			0.27		0.178		1.22	8.62					
Streptococcus pneumoniae	2.84			0.0594	0.964	0.459		0.0443			0.625	0.126	0.546						
All pathogens	57.1	1.81	2.98	5.64	5.04	15.7	0.182	6.71	1.68	1.37	4.74	0.163	2.34	8.62	0.000404	0.0434	0.0118	0.00479	0.0283

Counts
- <100k
- NA

Serbia DALYs (count) attributable to bacterial antimicrobial resistance by pathogen−drug combinations, 2019

	Resistance to 1+	AG	Anti−pseudomonal	BL−BLI	CP	FQ	4GC	3GC	Vanco	Aminopenicillin	TMP−SMX	PCN	Macrolide	Methicillin	Mono RIF	MDR excluding XDR in TB	XDR in TB	MDR in S. Typhi and Paratyphi	Mono INH
Acinetobacter baumannii	2,630	280	169	0.754	1,150	953	36.2	36.7											
Citrobacter spp.	304	24.8	54.7		28.8	55.8	23.4	117											
Enterobacter spp.	1,470	120	286		680	239	61.4				83.4								
Enterococcus faecalis	1,230					1,160			74.8										
Enterococcus faecium	2,450					1,850			600										
Other enterococci	469					430			38.4										
Escherichia coli	8,170	518		950	233	2,180		2,460		553	1,270								
Group A Streptococcus	130												130						
Group B Streptococcus	265					34.9						10.5	213						
Haemophilus influenzae	38.6							12.6		26									
Klebsiella pneumoniae	5,900	694		56.7	1,380	867		2,300			605								
Morganella spp.	33.2					14.4	1.88	16.9											
Mycobacterium tuberculosis	52.7														0.46	30.5	14.3		7.32
Neisseria gonorrhoeae	2.1					2		0.105											
Proteus spp.	516	63.3				112		269		34.2	36.3								
Pseudomonas aeruginosa	3,010	102	229		1,270	815	255	333											
Salmonella Paratyphi	0.0451					0.0449												0.000158	
Salmonella Typhi	37					33.2												3.56	
Non−typhoidal Salmonella	1.58					1.58													
Serratia spp.	384	26.9	18.6		23.6	21.1	243	51.5											
Shigella spp.	0.893					0.809													
Staphylococcus aureus	4,710					643			76.3		72.5		783	3,140					
Streptococcus pneumoniae	2,710			5.73	1,770	72.8		4.46			384	249	223						
All pathogens	34,500	1,830	757	1,010	6,540	9,490	621	5,600	790	614	2,450	259	1,370	3,140	0.46	30.5	14.3	3.59	7.5

Counts
<100k
NA

Slovakia DALYs (count) attributable to bacterial antimicrobial resistance by pathogen−drug combinations, 2019

	Resistance to 1+	AG	Anti−pseudomonal	BL−BLI	CP	FQ	4GC	3GC	Vanco	Aminopenicillin	TMP−SMX	PCN	Macrolide	Methicillin	Mono RIF	MDR excluding XDR in TB	XDR in TB	MDR in S. Typhi and Paratyphi	Mono INH
Acinetobacter baumannii	822	34.6	171	0.318	250	212	136	17.7											
Citrobacter spp.	85.3	1.08	23.5		20.6	10	1.43	28.6											
Enterobacter spp.	422	9.25	125		93.8	83.1	47.1				63.8								
Enterococcus faecalis	482					458			24.4										
Enterococcus faecium	1,640					1,080			567										
Other enterococci	164					136			28.7										
Escherichia coli	4,810	270		579	80.7	1,530		1,350		391	618								
Group A Streptococcus	45.8												49.5						
Group B Streptococcus	149					29.1						7.01	120						
Haemophilus influenzae	35.3							19.9		15.4									
Klebsiella pneumoniae	2,790	424		177	238	563		875			510								
Morganella spp.	5.03					2.37	0.434	2.22											
Mycobacterium tuberculosis	10														0.488	6.14	2.85		0.547
Neisseria gonorrhoeae	1.29					1.14		0.144											
Proteus spp.	210	4.09				51.7		100		28.7	23.4								
Pseudomonas aeruginosa	1,770	63.5	221		734	572	113	65.4											
Salmonella Paratyphi	0.0222					0.0221												0.000104	
Salmonella Typhi	18.3					16.5												1.95	
Non−typhoidal Salmonella	0.00223					0.00223													
Serratia spp.	80.3	2.97	9.66		18.1	5.29	32.4	11.9											
Shigella spp.	0.991					0.994													
Staphylococcus aureus	2,990					345			64.4		49.3		428	2,110					
Streptococcus pneumoniae	937			2.83	407	47.4		18.8			208	114	138						
All pathogens	17,500	810	550	760	1,840	5,140	330	2,490	685	436	1,470	121	730	2,110	0.488	6.14	2.85	1.81	0.56

Counts
<100k
NA

Slovenia DALYs (count) attributable to bacterial antimicrobial resistance by pathogen−drug combinations, 2019

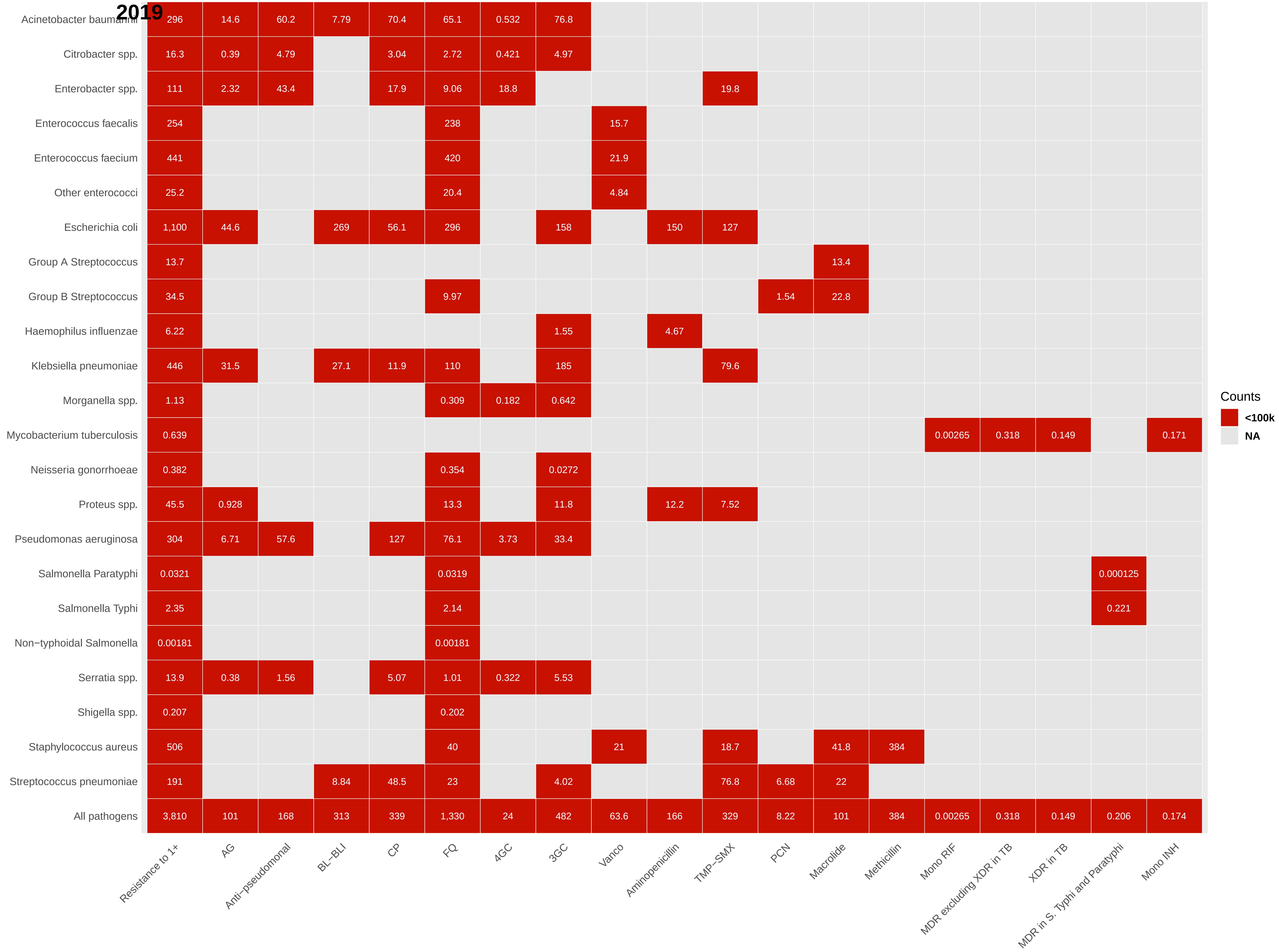

Spain DALYs (count) attributable to bacterial antimicrobial resistance by pathogen−drug combinations, 2019

	Resistance to 1+	AG	Anti−pseudomonal	BL−BLI	CP	FQ	4GC	3GC	TMP−SMX	Vanco	Aminopenicillin	PCN	Macrolide	Methicillin	Mono RIF	MDR excluding XDR in TB	XDR in TB	MDR in S. Typhi and Paratyphi	Mono INH
Acinetobacter baumannii	5,240	306	458	195	1,950	1,480	65	789											
Citrobacter spp.	433	7.18	171		63.6	48.5	9.24	134											
Enterobacter spp.	2,200	45.2	861		607	349	277		62.3										
Enterococcus faecalis	3,860					3,660				199									
Enterococcus faecium	8,260					7,770				490									
Other enterococci	700					619				81									
Escherichia coli	29,100	1,590		4,350	658	7,930		4,370	4,130		6,100								
Group A Streptococcus	446												451						
Group B Streptococcus	750					191						34.8	535						
Haemophilus influenzae	220							53.9			166								
Klebsiella pneumoniae	8,990	924		1,420	968	1,480		3,110	1,100										
Morganella spp.	59.3					28.6	4.8	25.9											
Mycobacterium tuberculosis	188														2.4	95.4	25.9		70.9
Neisseria gonorrhoeae	2.76					2.27		0.485											
Proteus spp.	952	25.9				124		236	313		266								
Pseudomonas aeruginosa	7,240	186	1,080		3,650	1,950	209	161											
Salmonella Paratyphi	0.461					0.459												0.00185	
Salmonella Typhi	45					40.4												4.42	
Non−typhoidal Salmonella	165					165													
Serratia spp.	198	5.65	42.3		41.3	22	13.4	73.7											
Shigella spp.	7.46					7.46													
Staphylococcus aureus	18,400					2,190			341	394			2,240	13,300					
Streptococcus pneumoniae	5,700			17.8	3,260	235		58.5	898			760	476						
All pathogens	93,200	3,090	2,620	5,990	11,200	28,300	578	9,010	6,840	1,160	6,520	795	3,660	13,300	2.4	95.4	25.9	4.71	71

Counts: **<100k** | **NA**

Sweden DALYs (count) attributable to bacterial antimicrobial resistance by pathogen−drug combinations, 2019

	Resistance to 1+	AG	Anti−pseudomonal	BL−BLI	CP	FQ	4GC	3GC	Vanco	Aminopenicillin	TMP−SMX	PCN	Macrolide	Methicillin	Mono RIF	MDR excluding XDR in TB	XDR in TB	MDR in S. Typhi and Paratyphi	Mono INH
Acinetobacter baumannii	778	16	355	84.1	60.7	50.1	33.7	179											
Citrobacter spp.	35.8	1.18	5.2		6.16	6.41	2.75	14.1											
Enterobacter spp.	249	7.28	115		107	11.4	2.35				5.23								
Enterococcus faecalis	250					208			41.4										
Enterococcus faecium	1,370					1,320			55.8										
Other enterococci	54.7					33			21.7										
Escherichia coli	3,360	168		909	87.1	930		526		186	558								
Group A Streptococcus	49.1												52						
Group B Streptococcus	199					37.3						7.79	164						
Haemophilus influenzae	20.8							8.21		12.6									
Klebsiella pneumoniae	770	58.3		271	42.3	147		200			51.9								
Morganella spp.	5.89					1.88	0.849	3.16											
Mycobacterium tuberculosis	59.2														0.326	34.8	9.46		14.9
Neisseria gonorrhoeae	0.747					0.68		0.0666											
Proteus spp.	136	9.96				11.7		22.2		61.6	28.4								
Pseudomonas aeruginosa	517	6.02	72.2		274	115	9.68	40.3											
Salmonella Paratyphi	0.191					0.19												0.000625	
Salmonella Typhi	6.31					5.67												0.636	
Non−typhoidal Salmonella	3.34					3.34													
Serratia spp.	62.4	0.916	23.4		7.48	1.98	1.25	27.4											
Shigella spp.	17.8					17.8													
Staphylococcus aureus	688					57.7			95.7		86.3		76.7	372					
Streptococcus pneumoniae	344			11.7	80	20		20			127	62.6	22.4						
All pathogens	8,990	268	571	1,280	665	2,980	50.6	1,040	215	262	856	70.4	311	372	0.326	34.8	9.46	0.604	14.6

Counts
<100k
NA

Switzerland DALYs (count) attributable to bacterial antimicrobial resistance by pathogen−drug combinations, 2019

	Resistance to 1+	AG	Anti−pseudomonal	BL−BLI	CP	FQ	4GC	3GC	Vanco	Aminopenicillin	TMP−SMX	PCN	Macrolide	Methicillin	Mono RIF	MDR excluding XDR in TB	XDR in TB	MDR in S. Typhi and Paratyphi	Mono INH
Acinetobacter baumannii	728	60.4	36.6	44.8	288	58.5	6.26	233											
Citrobacter spp.	54.7	0.966	7.75		5.51	3.99	2.13	34.3											
Enterobacter spp.	187	6.46	102		32.1	8.79	10				28.8								
Enterococcus faecalis	119					59.2			60.2										
Enterococcus faecium	1,180					1,110			73.4										
Other enterococci	44.2					27.7			16.5										
Escherichia coli	3,000	61.3		816	57.5	640		409		442	570								
Group A Streptococcus	27.4												27.1						
Group B Streptococcus	152					14.9						5.79	134						
Haemophilus influenzae	30.5							5.32		25.2									
Klebsiella pneumoniae	574	32.7		138	60.2	104		149			90.2								
Morganella spp.	4.49					1.54	0.519	2.42											
Mycobacterium tuberculosis	35.7														0.0522	25.2	6.85		3.39
Neisseria gonorrhoeae	0.69					0.571		0.119											
Proteus spp.	131	6.34				19.3		34.6		42.7	27.2								
Pseudomonas aeruginosa	503	8.15	132		151	143	25.7	43.4											
Salmonella Paratyphi	0.0425					0.0424												0.000132	
Salmonella Typhi	11.1					10												1.01	
Non−typhoidal Salmonella	2.76					2.76													
Serratia spp.	23.6	0.874	6.78		5.7	1.69	1.44	7.17											
Shigella spp.	6.94					6.94													
Staphylococcus aureus	1,340					105			64.9		53.8		147	965					
Streptococcus pneumoniae	442			2.74	144	40.4		90.8			118	10.2	35.3						
All pathogens	8,590	177	285	1,000	744	2,360	46.1	1,010	215	511	887	16	341	965	0.0522	25.2	6.85	1.14	3.83

Counts
<100k
NA

Tajikistan DALYs (count) attributable to bacterial antimicrobial resistance by pathogen−drug combinations, 2019

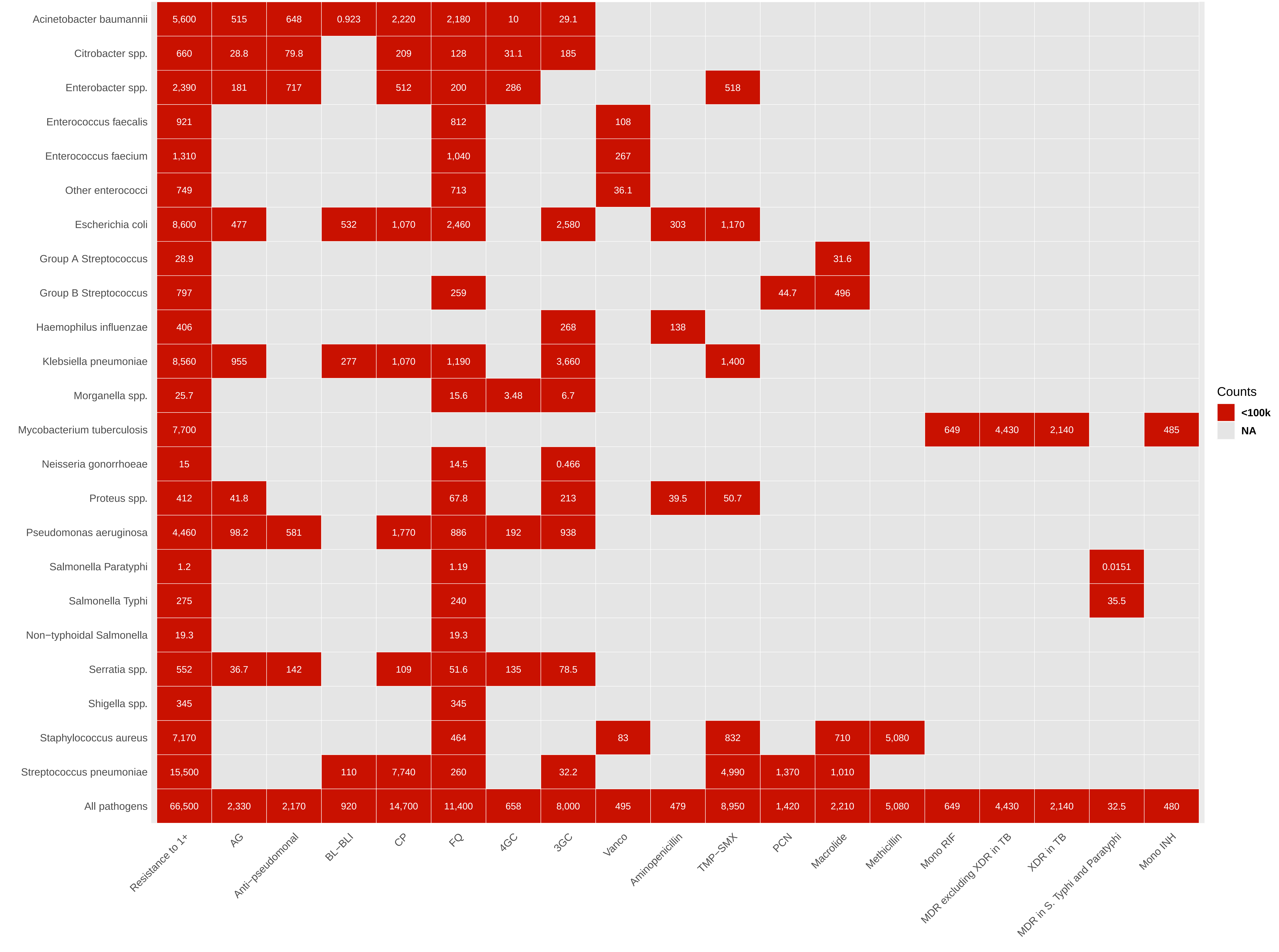

Turkey DALYs (count) attributable to bacterial antimicrobial resistance by pathogen−drug combinations, 2019

	Resistance to 1+	AG	Anti−pseudomonal	BL−BLI	CP	FQ	4GC	3GC	Vanco	Aminopenicillin	TMP−SMX	PCN	Macrolide	Methicillin	Mono RIF	MDR excluding XDR in TB	XDR in TB	MDR in S. Typhi and Paratyphi	Mono INH
Acinetobacter baumannii	19,200	2,130	569	36.6	9,010	7,170	42.5	281											
Citrobacter spp.	747	10.2	215		223	52.2	5.37	242											
Enterobacter spp.	5,430	117	1,640		3,060	178	62.3				378								
Enterococcus faecalis	2,070					1,370			700										
Enterococcus faecium	11,700					8,890			2,800										
Other enterococci	3,640					2,170			1,480										
Escherichia coli	31,900	2,190		3,720	1,610	7,260		11,300		1,390	4,450								
Group A Streptococcus	186												182						
Group B Streptococcus	3,290					497						51	2,680						
Haemophilus influenzae	507							113		393									
Klebsiella pneumoniae	35,300	4,640		2,210	13,100	5,990		6,050			3,280								
Morganella spp.	76.9					50.3	13.4	13.3											
Mycobacterium tuberculosis	1,660														82.1	1,100	84		400
Neisseria gonorrhoeae	39.6					37.9		1.66											
Proteus spp.	1,430	31.9				164		651		248	327								
Pseudomonas aeruginosa	14,000	351	2,430		7,130	3,050	377	668											
Salmonella Paratyphi	28.6					28.5												0.121	
Salmonella Typhi	639					608												30.4	
Non−typhoidal Salmonella	67.6					67.6													
Serratia spp.	1,950	91.1	443		578	244	433	167											
Shigella spp.	59.5					59.5													
Staphylococcus aureus	51,900					2,050			717		437		2,260	46,500					
Streptococcus pneumoniae	25,600			11.6	13,700	356		25.5			5,170	4,690	1,790						
All pathogens	212,000	9,560	5,290	5,980	48,400	40,300	933	19,500	5,690	2,030	14,100	4,740	6,920	46,500	82.1	1,100	84	32.3	384

Counts
100k − <500k
<100k
NA

Turkmenistan DALYs (count) attributable to bacterial antimicrobial resistance by pathogen−drug combinations, 2019

	Resistance to 1+	AG	Anti−pseudomonal	BL−BLI	CP	FQ	4GC	3GC	Vanco	Aminopenicillin	TMP−SMX	PCN	Macrolide	Methicillin	Mono RIF	MDR excluding XDR in TB	XDR in TB	MDR in S. Typhi and Paratyphi	Mono INH
Acinetobacter baumannii	1,960	197	194	0.682	830	712	1.21	23.7											
Citrobacter spp.	186	5.72	18.6		58.9	53.5	2.76	46.9											
Enterobacter spp.	800	48.7	246		166	108	92.1				141								
Enterococcus faecalis	563					470			92.4										
Enterococcus faecium	1,450					932			515										
Other enterococci	368					349			18.4										
Escherichia coli	5,480	419		548	326	1,440		1,730		217	802								
Group A Streptococcus	22.1												22.3						
Group B Streptococcus	494					236						49.5	200						
Haemophilus influenzae	245							135		111									
Klebsiella pneumoniae	4,900	551		350	422	665		2,060			851								
Morganella spp.	12.2					7.4	2.06	2.78											
Mycobacterium tuberculosis	4,530														64	2,660	1,290		520
Neisseria gonorrhoeae	4.9					4.76		0.145											
Proteus spp.	259	15.4				43.5		136		25.8	38.5								
Pseudomonas aeruginosa	2,720	90.6	439		943	661	128	462											
Salmonella Paratyphi	0.0209					0.0208												8.77e−05	
Salmonella Typhi	104					84.5												19.4	
Non−typhoidal Salmonella	1.67					1.67													
Serratia spp.	248	19.3	61.4		49.6	21.3	62.8	32.9											
Shigella spp.	21.9					21.9													
Staphylococcus aureus	3,900					425			74.9		359		690	2,350					
Streptococcus pneumoniae	4,920			51.3	2,210	90.5		17.4			1,700	480	360						
All pathogens	33,200	1,350	959	950	5,000	6,330	289	4,650	701	353	3,890	530	1,300	2,350	64	2,660	1,290	20.4	522

Counts
<100k
NA

Ukraine DALYs (count) attributable to bacterial antimicrobial resistance by pathogen−drug combinations, 2019

	Resistance to 1+	AG	Anti−pseudomonal	BL−BLI	CP	FQ	4GC	3GC	Vanco	Aminopenicillin	TMP−SMX	PCN	Macrolide	Methicillin	Mono RIF	MDR excluding XDR in TB	XDR in TB	MDR in S. Typhi and Paratyphi	Mono INH
Acinetobacter baumannii	21,500	2,380	1,420	3.35	9,500	7,840	133	194											
Citrobacter spp.	2,100	41.1	692		284	434	131	522											
Enterobacter spp.	7,550	232	2,870		1,150	883	1,490				918								
Enterococcus faecalis	10,600					9,800			819										
Enterococcus faecium	16,000					11,100			4,960										
Other enterococci	3,350					2,820			533										
Escherichia coli	44,500	2,170		5,380	2,880	10,100		14,100		2,350	7,560								
Group A Streptococcus	292												280						
Group B Streptococcus	1,840					270						113	1,500						
Haemophilus influenzae	269							90.1		179									
Klebsiella pneumoniae	28,900	4,820		2,050	2,970	5,180		9,950			3,980								
Morganella spp.	85.9					15.3	7.42	63.2											
Mycobacterium tuberculosis	51,300														3,280	30,700	15,100		2,070
Neisseria gonorrhoeae	12					11.9		0.111											
Proteus spp.	1,780	213				344		548		392	270								
Pseudomonas aeruginosa	18,400	672	1,940		8,940	4,370	916	1,530											
Salmonella Paratyphi	0.0612					0.061												0.000189	
Salmonella Typhi	380					261												118	
Non−typhoidal Salmonella	109					109													
Serratia spp.	2,090	187	480		352	73.3	644	352											
Shigella spp.	61.3					65.7													
Staphylococcus aureus	8,660					1,530			794		433		843	5,060					
Streptococcus pneumoniae	16,300			92.1	4,030	416		1,220			6,940	2,510	1,090						
All pathogens	236,000	10,700	7,410	7,530	30,100	55,600	3,320	28,500	7,100	2,930	20,100	2,620	3,720	5,060	3,280	30,700	15,100	113	2,140

Counts
100k − <500k
<100k
NA

United Kingdom DALYs (count) attributable to bacterial antimicrobial resistance by pathogen−drug combinations, 2019

	Resistance to 1+	AG	Anti−pseudomonal	BL−BLI	CP	FQ	4GC	3GC	Vanco	Aminopenicillin	TMP−SMX	PCN	Macrolide	Methicillin	Mono RIF	MDR excluding XDR in TB	XDR in TB	MDR in S. Typhi and Paratyphi	Mono INH
Acinetobacter baumannii	6,380	103	117	502	345	294	101	4,920											
Citrobacter spp.	803	11.8	446		63.6	43	6.9	232											
Enterobacter spp.	3,010	77	1,180		1,060	109	415				162								
Enterococcus faecalis	4,640					3,960			685										
Enterococcus faecium	15,400					10,900			4,540										
Other enterococci	746					495			252										
Escherichia coli	40,500	2,270		11,800	797	7,240		5,890		4,520	7,960								
Group A Streptococcus	256												246						
Group B Streptococcus	728					320						63.5	341						
Haemophilus influenzae	662							101		561									
Klebsiella pneumoniae	11,900	889		2,550	887	1,490		3,110			2,920								
Morganella spp.	66.7					20.4	10	36.2											
Mycobacterium tuberculosis	241														8.44	113	30.7		91.4
Neisseria gonorrhoeae	7.74					7.12		0.616											
Proteus spp.	1,290	68.9				106		292		354	464								
Pseudomonas aeruginosa	6,750	119	1,850		2,730	1,710	136	217											
Salmonella Paratyphi	0.873					0.87												0.0029	
Salmonella Typhi	69.3					61.9												7	
Non−typhoidal Salmonella	408					408													
Serratia spp.	417	9.88	86.3		61.9	36.1	19.8	203											
Shigella spp.	38.8					38.8													
Staphylococcus aureus	20,300					2,060			721		692		2,910	14,000					
Streptococcus pneumoniae	6,600			205	981	1,840		266			2,440	610	248						
All pathogens	121,000	3,550	3,680	15,100	6,920	31,100	689	15,300	6,200	5,430	14,600	674	3,760	14,000	8.44	113	30.7	8.07	88.9

Counts
100k − <500k
<100k
NA

Uzbekistan DALYs (count) attributable to bacterial antimicrobial resistance by pathogen−drug combinations, 2019

	Resistance to 1+	AG	Anti−pseudomonal	BL−BLI	CP	FQ	4GC	3GC	Vanco	Aminopenicillin	TMP−SMX	PCN	Macrolide	Methicillin	Mono RIF	MDR excluding XDR in TB	XDR in TB	MDR in S. Typhi and Paratyphi	Mono INH
Acinetobacter baumannii	10,400	1,020	1,210	3.6	4,440	3,650	8.36	104											
Citrobacter spp.	897	30.7	117		242	260	15.5	232											
Enterobacter spp.	4,650	262	1,600		913	512	645				717								
Enterococcus faecalis	3,090					2,610			480										
Enterococcus faecium	7,570					5,840			1,730										
Other enterococci	1,970					1,870			96										
Escherichia coli	31,000	1,990		3,680	1,940	7,400		9,810		1,360	4,870								
Group A Streptococcus	162												160						
Group B Streptococcus	3,430					1,440						354	1,670						
Haemophilus influenzae	1,510							883		627									
Klebsiella pneumoniae	32,400	3,390		1,260	3,180	4,110		15,200			5,280								
Morganella spp.	62.8					34.8	10.1	17.9											
Mycobacterium tuberculosis	31,200														2,570	18,300	8,970		1,340
Neisseria gonorrhoeae	34					32.9		1.1											
Proteus spp.	1,450	81.7				215		795		145	217								
Pseudomonas aeruginosa	16,900	494	2,710		5,860	4,100	983	2,760											
Salmonella Paratyphi	9.51					9.48												0.0349	
Salmonella Typhi	545					497												50.3	
Non−typhoidal Salmonella	7.33					7.33													
Serratia spp.	1,360	112	374		274	130	221	244											
Shigella spp.	23.9					23.9													
Staphylococcus aureus	23,300					2,800			496		1,900		4,120	14,000					
Streptococcus pneumoniae	29,300			710	12,000	642		190			12,000	1,310	2,450						
All pathogens	201,000	7,390	6,010	5,650	28,900	36,200	1,880	30,300	2,800	2,130	25,000	1,660	8,300	14,000	2,570	18,300	8,970	49.1	1,360

Counts: 100k – <500k; <100k; NA

Albania DALYs (count) associated with bacterial antimicrobial resistance by pathogen−drug combinations, 2019

	Resistance to 1+	AG	Anti−pseudomonal	BL−BLI	CP	FQ	4GC	3GC	TMP−SMX	Vanco	Aminopenicillin	Macrolide	PCN	MDR in S. Typhi and Paratyphi	Methicillin	Mono INH	Mono RIF	MDR excluding XDR in TB	XDR in TB
Acinetobacter baumannii	1,130	892	1,080	855	951	1,010	935	1,090											
Citrobacter spp.	107	8.21	43.7		15.6	32.6	13.5	89.1											
Enterobacter spp.	595	88.8	333		129	189	330		332										
Enterococcus faecalis	589					563				52.8									
Enterococcus faecium	1,850					1,840				691									
Other enterococci	386					376				103									
Escherichia coli	8,050	1,750		5,120	1,030	4,800		4,930	5,210		6,850								
Group A Streptococcus	218											218							
Group B Streptococcus	626					366						406	14.5						
Haemophilus influenzae	80.3							15.7			72.5								
Klebsiella pneumoniae	4,150	1,610		3,610	466	2,670		3,650	3,210										
Morganella spp.	8.01					5	1.65	4.84											
Mycobacterium tuberculosis	15.7															8.86	0.736	4.31	1.8
Neisseria gonorrhoeae	9.13					9.1		0.145											
Proteus spp.	482	25.1				199		240	329		385								
Pseudomonas aeruginosa	2,270	777	1,140		1,210	1,360	1,120	1,640											
Salmonella Paratyphi	0.125					0.124								0.00101					
Salmonella Typhi	76.5					68.5								9.84					
Non−typhoidal Salmonella	2					2													
Serratia spp.	112	36.8	22.8		19.6	22.2	69.5	62.5											
Shigella spp.	3.49					3.49													
Staphylococcus aureus	4,930					2,190			289	60.8		3,240			4,030				
Streptococcus pneumoniae	3,040			371	1,280	97.4		402	1,590			2,080	1,680						
All pathogens	28,700	5,190	2,630	9,950	5,110	15,800	2,470	12,100	11,000	908	7,300	5,950	1,700	9.84	4,030	8.86	0.736	4.31	1.8

Counts
<100k
NA

Andorra DALYs (count) associated with bacterial antimicrobial resistance by pathogen−drug combinations, 2019

	Resistance to 1+	AG	Anti−pseudomonal	BL−BLI	CP	FQ	4GC	3GC	TMP−SMX	Vanco	Aminopenicillin	Macrolide	PCN	MDR in S. Typhi and Paratyphi	Methicillin	Mono INH	Mono RIF	MDR excluding XDR in TB	XDR in TB
Acinetobacter baumannii	27.9	2.97	17.6	21.1	4.94	5.59	13.8	25.3											
Citrobacter spp.	2.42	0.17	1.1		0.199	0.608	0.274	2.07											
Enterobacter spp.	12.2	0.91	8.89		2.95	1.3	4.49		3.9										
Enterococcus faecalis	11.3					10.5				1.24									
Enterococcus faecium	65.6					65.4				13.5									
Other enterococci	2.81					2.5				0.761									
Escherichia coli	217	20.5		146	2.92	127		93.7	121		185								
Group A Streptococcus	2.94											2.94							
Group B Streptococcus	15.3					6.63						11.6	0.546						
Haemophilus influenzae	1.82							0.179			1.76								
Klebsiella pneumoniae	56.9	13.5		47.3	2.42	17.6		17.3	35.6										
Morganella spp.	0.201					0.122	0.0257	0.127											
Mycobacterium tuberculosis	0.415															0.377	0.00258	0.0285	0.00696
Neisseria gonorrhoeae	0.0611					0.0609		0.000816											
Proteus spp.	15.8	0.909				2.93		2.97	9.59		15.3								
Pseudomonas aeruginosa	28.3	4.78	12.8		15	15.3	11.4	11.4											
Salmonella Paratyphi	0.00204					0.00204								1.32e−05					
Salmonella Typhi	0.566					0.525								0.0513					
Non−typhoidal Salmonella	0.25					0.25													
Serratia spp.	1.72	0.176	0.48		0.305	0.307	0.857	1.23											
Shigella spp.	0.152					0.152													
Staphylococcus aureus	88.5					55.2			5.3	1.9		55.9			29.3				
Streptococcus pneumoniae	30.3			4.17	8.81	4.39		3.08	17.2			6.56	16						
All pathogens	582	43.9	40.9	219	37.6	317	30.9	157	193	17.4	202	77	16.6	0.0513	29.3	0.377	0.00258	0.0285	0.00696

Counts
<100k
NA

Armenia DALYs (count) associated with bacterial antimicrobial resistance by pathogen−drug combinations, 2019

	Resistance to 1+	AG	Anti−pseudomonal	BL−BLI	CP	FQ	4GC	3GC	TMP−SMX	Vanco	Aminopenicillin	Macrolide	PCN	MDR in S. Typhi and Paratyphi	Methicillin	Mono INH	Mono RIF	MDR excluding XDR in TB	XDR in TB
Acinetobacter baumannii	2,960	2,450	2,890	2,460	2,380	2,610	2,810	2,830											
Citrobacter spp.	250	15.5	56.2		63.4	118	32.1	172											
Enterobacter spp.	1,680	272	1,110		220	462	997		724										
Enterococcus faecalis	1,300					1,260				98.3									
Enterococcus faecium	3,170					3,160				596									
Other enterococci	1,260					1,250				147									
Escherichia coli	10,400	2,360		7,150	683	6,720		6,190	7,000		9,100								
Group A Streptococcus	169											169							
Group B Streptococcus	1,090					340						928	70						
Haemophilus influenzae	89.9							20.7			78.1								
Klebsiella pneumoniae	7,420	3,110		6,600	759	4,710		6,300	6,140										
Morganella spp.	40.1					25.4	5.63	26.1											
Mycobacterium tuberculosis	1,430															391	120	642	277
Neisseria gonorrhoeae	23.2					23.2		0.0986											
Proteus spp.	1,090	308				312		324	612		1,040								
Pseudomonas aeruginosa	4,370	1,530	2,590		2,630	2,380	2,550	2,960											
Salmonella Paratyphi	7.79					7.77								0.0412					
Salmonella Typhi	111					102								11.1					
Non−typhoidal Salmonella	3.85					3.85													
Serratia spp.	565	169	185		87.4	87.1	409	345											
Shigella spp.	49.7					49.7													
Staphylococcus aureus	5,120					3,110			342	136		2,840			3,390				
Streptococcus pneumoniae	3,850			355	933	174		512	3,410			672	1,600						
All pathogens	46,400	10,200	6,830	16,600	7,760	26,900	6,800	19,700	18,200	977	10,200	4,600	1,670	11.2	3,390	391	120	642	277

Counts
- <100k
- NA

Austria DALYs (count) associated with bacterial antimicrobial resistance by pathogen−drug combinations, 2019

	Resistance to 1+	AG	Anti−pseudomonal	BL−BLI	CP	FQ	4GC	3GC	TMP−SMX	Vanco	Aminopenicillin	Macrolide	PCN	MDR in S. Typhi and Paratyphi	Methicillin	Mono INH	Mono RIF	MDR excluding XDR in TB	XDR in TB
Acinetobacter baumannii	3,730	337	3,640	2,600	354	447	3,640	3,640											
Citrobacter spp.	252	20.1	93.4		58.4	50.6	37.4	201											
Enterobacter spp.	1,990	95	1,700		245	152	940		444										
Enterococcus faecalis	672					614				77.7									
Enterococcus faecium	6,480					6,470				478									
Other enterococci	236					207				61.3									
Escherichia coli	18,300	2,270		9,770	44.1	8,180		4,060	11,200		15,400								
Group A Streptococcus	169											169							
Group B Streptococcus	1,350					156						1,290	27.4						
Haemophilus influenzae	144							20.7			136								
Klebsiella pneumoniae	4,330	583		2,890	184	2,070		1,400	2,970										
Morganella spp.	16.1					8.51	1.71	11.3											
Mycobacterium tuberculosis	154															67.7	0.674	68.7	17
Neisseria gonorrhoeae	4.42					4.38		0.322											
Proteus spp.	1,800	47.3				326		242	611		1,770								
Pseudomonas aeruginosa	3,160	499	1,680		1,800	1,450	957	1,330											
Salmonella Paratyphi	1.48					1.49								0.00901					
Salmonella Typhi	48.5					39								11.3					
Non−typhoidal Salmonella	217					217													
Serratia spp.	70.8	18.6	21.7		22.3	20.2	17.2	30.5											
Shigella spp.	41.3					41.3													
Staphylococcus aureus	10,100					2,820			599	208		9,040			2,940				
Streptococcus pneumoniae	2,000			231	334	329		212	1,110			901	558						
All pathogens	55,200	3,870	7,130	15,500	3,050	23,600	5,590	11,200	16,900	824	17,300	11,400	585	11.3	2,940	67.7	0.674	68.7	17

Counts
- <100k
- NA

Azerbaijan DALYs (count) associated with bacterial antimicrobial resistance by pathogen−drug combinations, 2019

	Resistance to 1+	AG	Anti−pseudomonal	BL−BLI	CP	FQ	4GC	3GC	TMP−SMX	Vanco	Aminopenicillin	Macrolide	PCN	MDR in S. Typhi and Paratyphi	Methicillin	Mono INH	Mono RIF	MDR excluding XDR in TB	XDR in TB
Acinetobacter baumannii	10,300	8,080	10,100	9,100	8,590	9,170	9,710	9,890											
Citrobacter spp.	899	168	225		269	459	130	605											
Enterobacter spp.	6,100	1,190	3,570		1,020	1,200	3,320		3,350										
Enterococcus faecalis	2,670					2,480				358									
Enterococcus faecium	8,370					8,330				2,030									
Other enterococci	3,100					3,100				196									
Escherichia coli	32,400	9,430		24,000	2,360	20,000		19,000	22,400		29,900								
Group A Streptococcus	615											615							
Group B Streptococcus	7,380					3,820						5,230	644						
Haemophilus influenzae	1,530							722			1,070								
Klebsiella pneumoniae	31,200	12,200		26,700	2,620	16,800		25,500	26,700										
Morganella spp.	80.1					63.6	15.7	35.1											
Mycobacterium tuberculosis	15,100															2,520	1,780	7,470	3,300
Neisseria gonorrhoeae	108					108		1.24											
Proteus spp.	2,640	515				965		1,130	1,670		2,410								
Pseudomonas aeruginosa	16,900	5,320	8,300		8,680	10,100	8,810	11,700											
Salmonella Paratyphi	1.97					1.96								0.0123					
Salmonella Typhi	716					585								165					
Non−typhoidal Salmonella	17.1					17.1													
Serratia spp.	1,610	561	577		312	374	711	1,090											
Shigella spp.	95.2					95.2													
Staphylococcus aureus	31,300					14,000			4,520	387		27,200			13,900				
Streptococcus pneumoniae	34,600			7,160	13,200	1,360		4,380	30,400			15,400	12,500						
All pathogens	208,000	37,500	22,800	67,000	37,100	92,900	22,700	74,100	89,000	2,970	33,400	48,500	13,200	165	13,900	2,520	1,780	7,470	3,300

Counts
- 100k – <500k
- <100k
- NA

Belarus DALYs (count) associated with bacterial antimicrobial resistance by pathogen−drug combinations, 2019

	Resistance to 1+	AG	Anti−pseudomonal	BL−BLI	CP	FQ	4GC	3GC	TMP−SMX	Vanco	Aminopenicillin	Macrolide	PCN	MDR in S. Typhi and Paratyphi	Methicillin	Mono INH	Mono RIF	MDR excluding XDR in TB	XDR in TB
Acinetobacter baumannii	9,530	7,960	9,110	8,010	6,390	9,240	9,480	8,910											
Citrobacter spp.	935	73.1	480		204	164	72.1	757											
Enterobacter spp.	5,230	503	3,750		702	877	2,400		2,370										
Enterococcus faecalis	6,310					6,220				392									
Enterococcus faecium	9,530					9,510				2,110									
Other enterococci	2,390					2,380				123									
Escherichia coli	30,800	12,100		25,700	1,160	14,800		22,300	18,100		23,200								
Group A Streptococcus	401											401							
Group B Streptococcus	3,450					219						3,390	27.5						
Haemophilus influenzae	93.9							43.9			60.9								
Klebsiella pneumoniae	17,700	12,900		17,000	4,120	11,600		16,100	17,100										
Morganella spp.	45.2					16.1	3.08	38.8											
Mycobacterium tuberculosis	7,850															454	64.3	5,120	2,200
Neisseria gonorrhoeae	20.6					20.5		0.066											
Proteus spp.	2,790	204				939		377	1,210		2,740								
Pseudomonas aeruginosa	11,400	4,280	6,600		8,610	8,450	6,050	5,920											
Salmonella Paratyphi	0.246					0.245								0.00157					
Salmonella Typhi	210					130								105					
Non−typhoidal Salmonella	123					123													
Serratia spp.	637	196	129		136	65.4	205	480											
Shigella spp.	85.4					85.4													
Staphylococcus aureus	8,980					2,070			682	231		5,960			5,880				
Streptococcus pneumoniae	9,790			676	1,080	640		706	8,310			3,110	3,440						
All pathogens	128,000	38,200	20,100	51,300	22,400	67,500	18,200	55,700	47,800	2,850	26,000	12,900	3,470	105	5,880	454	64.3	5,120	2,200

Counts
100k − <500k
<100k
NA

Belgium DALYs (count) associated with bacterial antimicrobial resistance by pathogen−drug combinations, 2019

	Resistance to 1+	AG	Anti−pseudomonal	BL−BLI	CP	FQ	4GC	3GC	TMP−SMX	Vanco	Aminopenicillin	Macrolide	PCN	MDR in S. Typhi and Paratyphi	Methicillin	Mono INH	Mono RIF	MDR excluding XDR in TB	XDR in TB
Acinetobacter baumannii	4,410	441	1,640	4,270	530	870	1,880	3,040											
Citrobacter spp.	515	29.1	335		71	138	37.2	409											
Enterobacter spp.	2,560	177	2,040		399	1,110	751		338										
Enterococcus faecalis	992					815				227									
Enterococcus faecium	9,890					9,870				581									
Other enterococci	661					594				181									
Escherichia coli	33,000	3,690		20,100	760	14,100		6,640	19,300		29,500								
Group A Streptococcus	234											234							
Group B Streptococcus	1,930					362						1,760	39.9						
Haemophilus influenzae	660							57			647								
Klebsiella pneumoniae	9,400	2,070		7,760	620	5,550		5,800	4,410										
Morganella spp.	55.8					27	3.95	44.6											
Mycobacterium tuberculosis	148															76.7	2.17	55	13.7
Neisseria gonorrhoeae	5.22					5.09		0.572											
Proteus spp.	3,080	87.8				527		209	1,160		3,060								
Pseudomonas aeruginosa	6,550	1,540	3,370		4,130	3,010	3,040	2,930											
Salmonella Paratyphi	0.695					0.695								0.00572					
Salmonella Typhi	68.2					56.2								14.2					
Non−typhoidal Salmonella	721					721													
Serratia spp.	276	24.8	67.2		37.7	57.6	49.3	246											
Shigella spp.	93.9					93.9													
Staphylococcus aureus	20,600					10,200			1,200	319		15,900			8,730				
Streptococcus pneumoniae	6,250			813	2,830	199		145	3,480			3,340	247						
All pathogens	102,000	8,060	7,450	32,900	9,370	48,300	5,760	19,500	29,900	1,310	33,200	21,200	287	14.3	8,730	76.7	2.17	55	13.7

Counts
100k – <500k
<100k
NA

Bosnia and Herzegovina DALYs (count) associated with bacterial antimicrobial resistance by pathogen−drug combinations, 2

	Resistance to 1+	AG	Anti−pseudomonal	BL−BLI	CP	FQ	4GC	3GC	TMP−SMX	Vanco	Aminopenicillin	Macrolide	PCN	MDR in S. Typhi and Paratyphi	Methicillin	Mono INH	Mono RIF	MDR excluding XDR in TB	XDR in TB
Acinetobacter baumannii	4,100	2,740	3,990	3,290	2,560	3,820	3,750	3,780											
Citrobacter spp.	651	39.7	255		120	470	116	350											
Enterobacter spp.	2,060	404	1,200		226	898	1,270		1,080										
Enterococcus faecalis	1,600					1,580				84.2									
Enterococcus faecium	2,950					2,940				995									
Other enterococci	933					918				216									
Escherichia coli	8,170	2,320		5,690	1,280	3,590		3,790	4,410		6,810								
Group A Streptococcus	215											215							
Group B Streptococcus	848					135						800	21						
Haemophilus influenzae	75							14.4			67.6								
Klebsiella pneumoniae	5,580	3,170		5,160	1,780	3,450		5,040	2,240										
Morganella spp.	21.2					12.9	4.1	14.2											
Mycobacterium tuberculosis	66.1															28.7	0.456	25.6	11.3
Neisseria gonorrhoeae	7.79					7.74		0.151											
Proteus spp.	927	187				464		295	460		845								
Pseudomonas aeruginosa	3,480	1,420	2,080		2,260	2,110	1,790	2,150											
Salmonella Paratyphi	0.0852					0.085								0.000495					
Salmonella Typhi	59.5					54.4								6.21					
Non−typhoidal Salmonella	4.66					4.66													
Serratia spp.	353	139	99.1		62.6	56.4	192	229											
Shigella spp.	3.6					3.6													
Staphylococcus aureus	4,710					2,030			243	64.3		3,640			2,980				
Streptococcus pneumoniae	3,020			319	1,000	168		1,560	933			1,580	1,830						
All pathogens	39,800	10,400	7,620	14,500	9,290	22,700	7,130	17,200	9,360	1,360	7,730	6,240	1,850	6.21	2,980	28.7	0.456	25.6	11.3

Counts
<100k
NA

Bulgaria DALYs (count) associated with bacterial antimicrobial resistance by pathogen−drug combinations, 2019

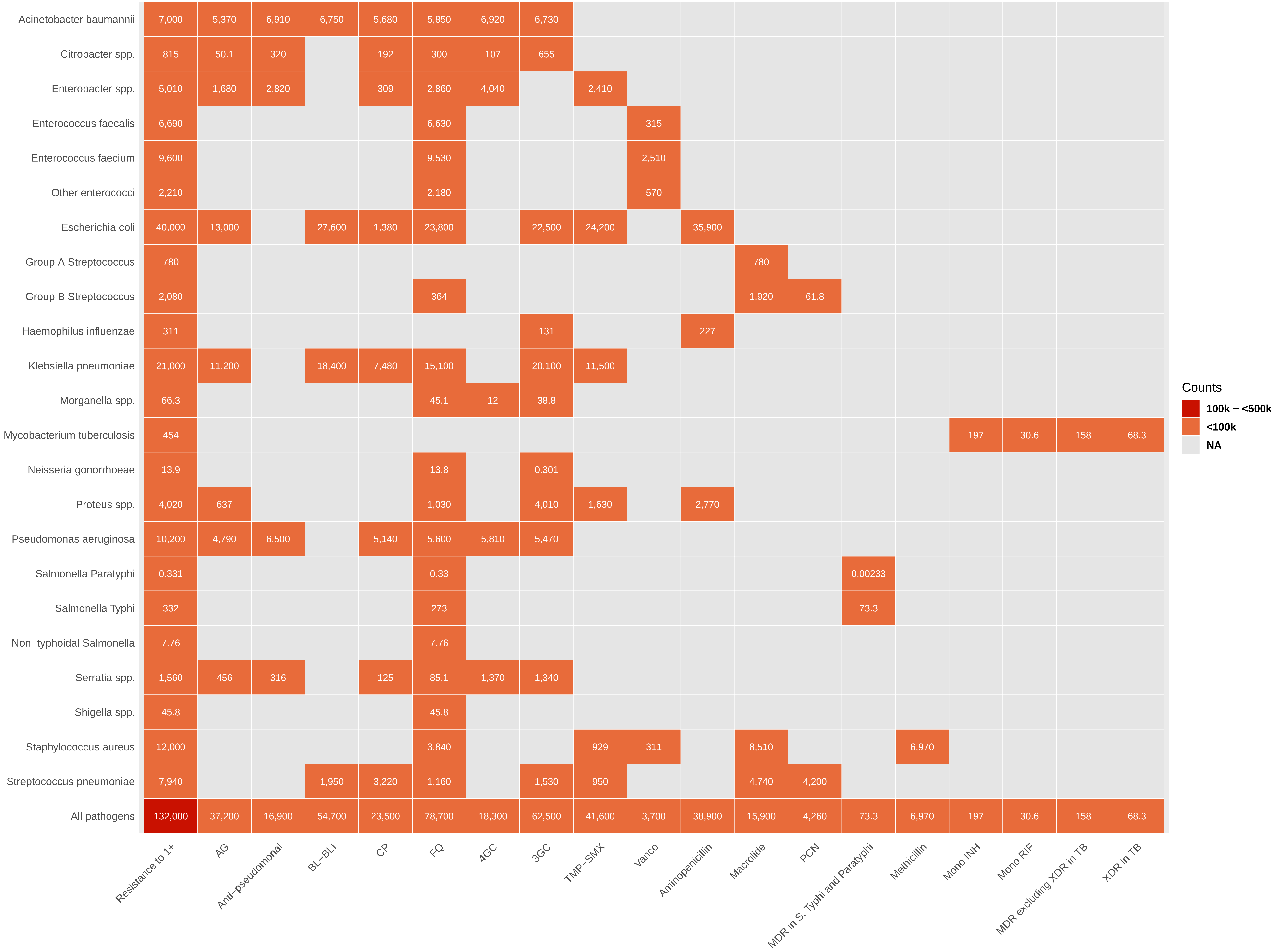

Croatia DALYs (count) associated with bacterial antimicrobial resistance by pathogen−drug combinations, 2019

	Resistance to 1+	AG	Anti−pseudomonal	BL−BLI	CP	FQ	4GC	3GC	TMP−SMX	Vanco	Aminopenicillin	Macrolide	PCN	MDR in S. Typhi and Paratyphi	Methicillin	Mono INH	Mono RIF	MDR excluding XDR in TB	XDR in TB
Acinetobacter baumannii	5,190	4,560	5,100	4,550	5,000	5,040	5,110	5,100											
Citrobacter spp.	364	27.6	150		48.1	90.3	41.1	306											
Enterobacter spp.	2,460	89.6	1,030		271	1,060	2,080		893										
Enterococcus faecalis	1,930					1,890				108									
Enterococcus faecium	3,870					3,840				1,460									
Other enterococci	650					642				62.3									
Escherichia coli	12,200	1,710		7,130	193	5,680		3,240	10,300		9,530								
Group A Streptococcus	168											168							
Group B Streptococcus	831					100						801	10.3						
Haemophilus influenzae	25.1							13.3			14.7								
Klebsiella pneumoniae	5,090	2,060		4,290	745	3,940		3,840	2,590										
Morganella spp.	30.4					4.24	2.11	30.1											
Mycobacterium tuberculosis	31.2															17.9	0.138	9.16	3.95
Neisseria gonorrhoeae	13.9					13.9		0.0218											
Proteus spp.	1,040	71				391		228	472		991								
Pseudomonas aeruginosa	3,860	1,050	1,600		2,410	2,300	2,710	1,860											
Salmonella Paratyphi	0.108					0.108								0.000766					
Salmonella Typhi	23.9					20.4								4.25					
Non−typhoidal Salmonella	4					4													
Serratia spp.	163	25.2	33.5		21.5	33.6	78.8	117											
Shigella spp.	3.39					3.39													
Staphylococcus aureus	6,390					4,000			333	104		3,980			4,170				
Streptococcus pneumoniae	2,660			897	1,070	100		200	1,110			1,800	1,230						
All pathogens	47,000	9,590	7,910	16,900	9,760	29,100	10,000	14,900	15,700	1,740	10,500	6,750	1,240	4.25	4,170	17.9	0.138	9.16	3.95

Counts
<100k
NA

Cyprus DALYs (count) associated with bacterial antimicrobial resistance by pathogen−drug combinations, 2019

	Resistance to 1+	AG	Anti−pseudomonal	BL−BLI	CP	FQ	4GC	3GC	TMP−SMX	Vanco	Aminopenicillin	Macrolide	PCN	MDR in S. Typhi and Paratyphi	Methicillin	Mono INH	Mono RIF	MDR excluding XDR in TB	XDR in TB
Acinetobacter baumannii	396	289	246	322	355	367	312	350											
Citrobacter spp.	42.8	2.52	13.1		4.7	11.8	8.79	36.8											
Enterobacter spp.	172	11.5	121		49.6	68.5	54		42										
Enterococcus faecalis	293					284				20.8									
Enterococcus faecium	776					768				517									
Other enterococci	126					122				28.6									
Escherichia coli	3,110	692		1,340	83.8	1,940		1,570	1,550		2,760								
Group A Streptococcus	48.7											48.7							
Group B Streptococcus	184					18.7						177	5.37						
Haemophilus influenzae	25.7							9.47			20.9								
Klebsiella pneumoniae	1,030	508		742	445	855		756	356										
Morganella spp.	4.42					3.55	0.559	1.85											
Mycobacterium tuberculosis	20															15.4	0.00575	3.68	0.9
Neisseria gonorrhoeae	1.11					1.11		0.0266											
Proteus spp.	193	14.4				102		38.8	90.7		166								
Pseudomonas aeruginosa	513	45.3	277		283	281	178	225											
Salmonella Paratyphi	0.0403					0.0403								0.000315					
Salmonella Typhi	7.61					6.56								1.31					
Non−typhoidal Salmonella	0.00256					0.00256													
Serratia spp.	21.1	2.68	4		5.54	3.33	7.25	16.4											
Shigella spp.	3.83					3.83													
Staphylococcus aureus	1,980					1,180			169	27.1		1,320			1,440				
Streptococcus pneumoniae	471			65.1	272	27.5		111	218			146	295						
All pathogens	9,420	1,570	661	2,470	1,500	6,040	561	3,110	2,430	593	2,940	1,690	300	1.31	1,440	15.4	0.00575	3.68	0.9

Counts
<100k
NA

Czechia DALYs (count) associated with bacterial antimicrobial resistance by pathogen−drug combinations, 2019

	Resistance to 1+	AG	Anti−pseudomonal	BL−BLI	CP	FQ	4GC	3GC	TMP−SMX	Vanco	Aminopenicillin	Macrolide	PCN	MDR in S. Typhi and Paratyphi	Methicillin	Mono INH	Mono RIF	MDR excluding XDR in TB	XDR in TB
Acinetobacter baumannii	8,810	1,660	1,640	8,300	2,320	3,240	1,450	3,290											
Citrobacter spp.	916	56.4	333		202	145	83.2	786											
Enterobacter spp.	3,980	221	1,650		441	638	1,870		2,590										
Enterococcus faecalis	5,230					5,180				137									
Enterococcus faecium	9,190					9,170				2,170									
Other enterococci	1,110					1,070				203									
Escherichia coli	24,300	4,050		20,100	363	11,100		7,160	12,700		20,500								
Group A Streptococcus	1,480											1,480							
Group B Streptococcus	2,240					496						2,040	35.6						
Haemophilus influenzae	120							44.4			89.9								
Klebsiella pneumoniae	13,600	7,620		12,500	283	9,480		10,200	9,720										
Morganella spp.	12.8					5.7	3.52	7.9											
Mycobacterium tuberculosis	119															43.2	1.74	52	21.9
Neisseria gonorrhoeae	22.5					22.4		0.699											
Proteus spp.	2,640	83.1				201		124	1,170		2,610								
Pseudomonas aeruginosa	8,180	2,110	4,030		5,140	5,010	2,640	3,590											
Salmonella Paratyphi	3.89					3.87								0.0305					
Salmonella Typhi	82.6					65.4								19.9					
Non−typhoidal Salmonella	51.8					51.8													
Serratia spp.	323	43.2	60.5		96.5	122	113	185											
Shigella spp.	15.7					15.7													
Staphylococcus aureus	13,800					4,680			786	208		11,900			5,930				
Streptococcus pneumoniae	5,250			753	737	726		452	3,830			1,730	1,170						
All pathogens	101,000	15,800	7,720	41,600	9,590	51,400	6,170	25,800	30,800	2,720	23,200	17,200	1,210	19.9	5,930	43.2	1.74	52	21.9

Counts
100k − <500k
<100k
NA

Denmark DALYs (count) associated with bacterial antimicrobial resistance by pathogen−drug combinations, 2019

	Resistance to 1+	AG	Anti−pseudomonal	BL−BLI	CP	FQ	4GC	3GC	TMP−SMX	Vanco	Aminopenicillin	Macrolide	PCN	MDR in S. Typhi and Paratyphi	Methicillin	Mono INH	Mono RIF	MDR excluding XDR in TB	XDR in TB
Acinetobacter baumannii	2,220	115	743	2,150	160	174	623	1,240											
Citrobacter spp.	182	14	72.6		17.9	52.5	40.3	144											
Enterobacter spp.	1,460	97.8	805		129	120	391		1,020										
Enterococcus faecalis	1,570					1,550				43.2									
Enterococcus faecium	5,060					5,050				841									
Other enterococci	851					717				464									
Escherichia coli	14,700	1,900		6,090	275	4,190		2,540	9,930		12,200								
Group A Streptococcus	101											101							
Group B Streptococcus	1,040					136						990	11.9						
Haemophilus influenzae	195							26.9			185								
Klebsiella pneumoniae	5,210	506		2,700	42.7	1,070		1,120	4,840										
Morganella spp.	10.7					5.9	2.19	6.41											
Mycobacterium tuberculosis	48															23.5	0.0277	19.7	4.79
Neisseria gonorrhoeae	1.55					1.53		0.058											
Proteus spp.	1,250	42.1				40.2		46	980		1,070								
Pseudomonas aeruginosa	1,240	140	417		726	529	449	516											
Salmonella Paratyphi	10.7					10.7								0.0771					
Salmonella Typhi	29.2					25								4.82					
Non−typhoidal Salmonella	0.131					0.131													
Serratia spp.	96.2	12.4	19.2		16.2	17.7	8.56	81.1											
Shigella spp.	23.4					23.4													
Staphylococcus aureus	3,650					1,060			1,030	193		2,160			1,190				
Streptococcus pneumoniae	1,350			230	280	201		549	614			295	559						
All pathogens	40,300	2,830	2,060	11,200	1,650	15,000	1,510	6,280	18,400	1,540	13,400	3,550	571	4.89	1,190	23.5	0.0277	19.7	4.79

Counts
<100k
NA

Estonia DALYs (count) associated with bacterial antimicrobial resistance by pathogen−drug combinations, 2019

	Resistance to 1+	AG	Anti−pseudomonal	BL−BLI	CP	FQ	4GC	3GC	TMP−SMX	Vanco	Aminopenicillin	Macrolide	PCN	MDR in S. Typhi and Paratyphi	Methicillin	Mono INH	Mono RIF	MDR excluding XDR in TB	XDR in TB
Acinetobacter baumannii	1,520	416	1,280	1,250	654	738	1,340	1,420											
Citrobacter spp.	136	8.16	47.2		30.7	11.5	14.6	122											
Enterobacter spp.	520	25.6	334		103	65.4	238		230										
Enterococcus faecalis	686					674				41.4									
Enterococcus faecium	1,180					1,180				86.8									
Other enterococci	169					167				16.7									
Escherichia coli	2,790	360		985	15.9	1,030		603	2,070		2,140								
Group A Streptococcus	68.4											68.4							
Group B Streptococcus	319					32.9						311	6.14						
Haemophilus influenzae	13							3.97			10.4								
Klebsiella pneumoniae	1,620	373		847	34.2	706		638	1,570										
Morganella spp.	4.4					1.08	0.283	4.06											
Mycobacterium tuberculosis	382															68.6	5.72	216	92.6
Neisseria gonorrhoeae	2.33					2.32		0.00337											
Proteus spp.	329	20.3				37.6		26.6	141		324								
Pseudomonas aeruginosa	601	90.4	283		432	247	175	186											
Salmonella Paratyphi	0.246					0.245								0.00194					
Salmonella Typhi	8.91					7.47								1.63					
Non−typhoidal Salmonella	9.72e−56					9.72e−56													
Serratia spp.	76.2	20.1	16.8		17.3	9.78	26.1	59.2											
Shigella spp.	24.1					24.1													
Staphylococcus aureus	1,050					219			93.2	32.4		901			286				
Streptococcus pneumoniae	806			51.4	183	40.6		59.6	734			160	52						
All pathogens	12,300	1,310	1,960	3,140	1,470	5,190	1,800	3,120	4,840	177	2,470	1,440	58.2	1.64	286	68.6	5.72	216	92.6

Counts
- <100k
- NA

Finland DALYs (count) associated with bacterial antimicrobial resistance by pathogen−drug combinations, 2019

	Resistance to 1+	AG	Anti−pseudomonal	BL−BLI	CP	FQ	4GC	3GC	TMP−SMX	Vanco	Aminopenicillin	Macrolide	PCN	MDR in S. Typhi and Paratyphi	Methicillin	Mono INH	Mono RIF	MDR excluding XDR in TB	XDR in TB
Acinetobacter baumannii	1,940	147	857	1,460	126	90.1	1,410	1,670											
Citrobacter spp.	165	12.4	51.6		43.1	28.1	24.9	133											
Enterobacter spp.	997	59.1	705		249	124	369		352										
Enterococcus faecalis	958					869				142									
Enterococcus faecium	4,610					4,610				124									
Other enterococci	153					104				79.7									
Escherichia coli	9,140	1,800		4,060	135	3,170		2,080	4,870		7,430								
Group A Streptococcus	218											218							
Group B Streptococcus	851					120						804	39.1						
Haemophilus influenzae	93.9							26.9			80.1								
Klebsiella pneumoniae	3,020	408		2,070	117	1,100		638	2,230										
Morganella spp.	9.51					5.51	1.05	6.18											
Mycobacterium tuberculosis	105															33.6	0.105	57.2	14.3
Neisseria gonorrhoeae	4.77					4.75		0.0239											
Proteus spp.	1,190	62.4				90.4		167	445		1,170								
Pseudomonas aeruginosa	1,660	107	591		856	952	831	535											
Salmonella Paratyphi	0.575					0.575								0.0045					
Salmonella Typhi	20.5					17								3.95					
Non−typhoidal Salmonella	0.586					0.586													
Serratia spp.	157	13.3	32.7		39.4	22.8	94.3	104											
Shigella spp.	12.1					12.1													
Staphylococcus aureus	2,090					696			369	139		1,040			1,190				
Streptococcus pneumoniae	1,520			114	359	112		241	820			728	678						
All pathogens	28,900	2,610	2,240	7,710	1,920	12,100	2,730	5,610	9,090	485	8,680	2,790	717	3.95	1,190	33.6	0.105	57.2	14.3

Counts
<100k
NA

France DALYs (count) associated with bacterial antimicrobial resistance by pathogen−drug combinations, 2019

	Resistance to 1+	AG	Anti−pseudomonal	BL−BLI	CP	FQ	4GC	3GC	TMP−SMX	Vanco	Aminopenicillin	Macrolide	PCN	MDR in S. Typhi and Paratyphi	Methicillin	Mono INH	Mono RIF	MDR excluding XDR in TB	XDR in TB
Acinetobacter baumannii	24,200	2,840	7,470	23,800	3,070	4,280	5,450	15,700											
Citrobacter spp.	1,980	138	981		213	338	477	1,680											
Enterobacter spp.	13,800	450	8,920		1,950	3,300	6,250		6,520										
Enterococcus faecalis	7,090					6,490				906									
Enterococcus faecium	46,100					46,000				1,110									
Other enterococci	1,880					1,610				561									
Escherichia coli	161,000	18,800		1e+05	4,810	50,400		33,600	92,800		148,000								
Group A Streptococcus	3,340											3,340							
Group B Streptococcus	5,090					1,060						4,410	201						
Haemophilus influenzae	2,380							176			2,340								
Klebsiella pneumoniae	55,500	31,100		43,500	1,570	31,700		38,100	32,200										
Morganella spp.	244					106	17.6	209											
Mycobacterium tuberculosis	1,310															621	18	538	137
Neisseria gonorrhoeae	30.9					30.7		0.477											
Proteus spp.	10,200	995				1,350		1,320	3,310		9,920								
Pseudomonas aeruginosa	34,100	9,530	20,800		19,500	15,300	14,100	16,200											
Salmonella Paratyphi	1.89					1.89								0.0164					
Salmonella Typhi	366					287								91.3					
Non−typhoidal Salmonella	1,070					1,070													
Serratia spp.	1,460	143	474		194	257	173	1,310											
Shigella spp.	182					182													
Staphylococcus aureus	81,200					35,500			5,230	863		58,000			44,400				
Streptococcus pneumoniae	48,900			3,480	15,100	30,200		6,510	6,810			18,300	23,400						
All pathogens	502,000	64,000	38,700	171,000	46,400	229,000	26,400	115,000	147,000	3,440	160,000	84,000	23,600	91.3	44,400	621	18	538	137

Counts
- **500k − <1M**
- **100k − <500k**
- **<100k**
- **NA**

Georgia DALYs (count) associated with bacterial antimicrobial resistance by pathogen−drug combinations, 2019

	Resistance to 1+	AG	Anti−pseudomonal	BL−BLI	CP	FQ	4GC	3GC	TMP−SMX	Vanco	Aminopenicillin	Macrolide	PCN	MDR in S. Typhi and Paratyphi	Methicillin	Mono INH	Mono RIF	MDR excluding XDR in TB	XDR in TB
Acinetobacter baumannii	5,820	3,400	5,690	5,030	4,010	5,290	5,660	5,590											
Citrobacter spp.	387	57.4	94.8		98.3	125	88.3	310											
Enterobacter spp.	2,640	532	1,650		351	691	1,680		1,220										
Enterococcus faecalis	1,960					1,910				136									
Enterococcus faecium	4,140					4,120				766									
Other enterococci	1,390					1,390				146									
Escherichia coli	12,000	3,100		8,180	4,910	6,930		7,400	8,540		9,870								
Group A Streptococcus	212											212							
Group B Streptococcus	1,200					173						1,140	38.7						
Haemophilus influenzae	128							32.8			109								
Klebsiella pneumoniae	10,500	4,210		8,380	4,800	6,180		10,400	4,540										
Morganella spp.	29.4					22.4	4.57	14.9											
Mycobacterium tuberculosis	3,510															723	133	1,840	805
Neisseria gonorrhoeae	27.1					27		0.328											
Proteus spp.	1,230	326				505		330	659		1,160								
Pseudomonas aeruginosa	5,760	1,790	3,380		3,350	3,420	3,230	3,470											
Salmonella Paratyphi	8.73					8.7								0.0712					
Salmonella Typhi	176					161								17.2					
Non−typhoidal Salmonella	8.79					8.79													
Serratia spp.	998	348	287		134	142	771	594											
Shigella spp.	46.4					46.4													
Staphylococcus aureus	7,110					3,070			402	141		5,260			4,190				
Streptococcus pneumoniae	5,360			536	2,340	188		745	4,470			1,300	2,380						
All pathogens	64,700	13,800	11,100	22,100	20,000	34,400	11,400	28,900	19,800	1,190	11,100	7,910	2,410	17.2	4,190	723	133	1,840	805

Counts
<100k
NA

Germany DALYs (count) associated with bacterial antimicrobial resistance by pathogen−drug combinations, 2019

	Resistance to 1+	AG	Anti−pseudomonal	BL−BLI	CP	FQ	4GC	3GC	TMP−SMX	Vanco	Aminopenicillin	Macrolide	PCN	MDR in S. Typhi and Paratyphi	Methicillin	Mono INH	Mono RIF	MDR excluding XDR in TB	XDR in TB
Acinetobacter baumannii	27,700	2,100	10,900	15,000	2,850	4,550	15,000	22,500											
Citrobacter spp.	2,950	250	1,440		374	732	419	2,390											
Enterobacter spp.	19,700	1,090	13,600		3,320	3,530	9,400		6,970										
Enterococcus faecalis	19,300					18,700				1,010									
Enterococcus faecium	85,100					84,900				23,400									
Other enterococci	3,270					2,620				1,240									
Escherichia coli	266,000	37,200		196,000	3,370	115,000		63,400	140,000		246,000								
Group A Streptococcus	2,030											2,030							
Group B Streptococcus	16,100					2,150						15,400	282						
Haemophilus influenzae	1,820							293			1,690								
Klebsiella pneumoniae	70,700	18,200		64,400	1,650	33,300		28,300	32,600										
Morganella spp.	126					52.6	26.4	89.5											
Mycobacterium tuberculosis	1,450															569	26.2	688	169
Neisseria gonorrhoeae	41.3					41.1		1.5											
Proteus spp.	20,400	1,220				1,900		1,810	8,380		20,100								
Pseudomonas aeruginosa	53,400	8,760	30,100		33,300	25,700	24,200	19,400											
Salmonella Paratyphi	2.42					2.43								0.0137					
Salmonella Typhi	578					430								174					
Non−typhoidal Salmonella	265					265													
Serratia spp.	2,700	223	389		418	608	419	2,410											
Shigella spp.	356					356													
Staphylococcus aureus	132,000					67,700			7,610	1,370		101,000			49,300				
Streptococcus pneumoniae	26,500			2,230	6,700	12,900		1,360	5,610			9,470	6,290						
All pathogens	753,000	69,100	56,500	278,000	52,000	375,000	49,400	142,000	201,000	27,000	268,000	128,000	6,580	174	49,300	569	26.2	688	169

Counts
- **500k − <1M**
- **100k − <500k**
- **<100k**
- **NA**

Greece DALYs (count) associated with bacterial antimicrobial resistance by pathogen−drug combinations, 2019

	Resistance to 1+	AG	Anti−pseudomonal	BL−BLI	CP	FQ	4GC	3GC	TMP−SMX	Vanco	Aminopenicillin	Macrolide	PCN	MDR in S. Typhi and Paratyphi	Methicillin	Mono INH	Mono RIF	MDR excluding XDR in TB	XDR in TB
Acinetobacter baumannii	4,560	4,000	4,470	4,480	4,400	4,440	4,470	4,490											
Citrobacter spp.	241	21.5	106		78.1	115	25.5	101											
Enterobacter spp.	2,300	112	1,380		472	1,070	838		1,110										
Enterococcus faecalis	3,580					3,350				584									
Enterococcus faecium	8,290					8,270				3,380									
Other enterococci	1,200					1,070				479									
Escherichia coli	29,000	3,950		12,300	1,110	15,300		10,600	14,800		25,600								
Group A Streptococcus	779											779							
Group B Streptococcus	697					157						580	27.3						
Haemophilus influenzae	579							296			414								
Klebsiella pneumoniae	15,300	8,600		13,100	10,300	12,800		13,600	9,430										
Morganella spp.	22.4					19.8	2.94	5.2											
Mycobacterium tuberculosis	292															129	0.556	129	32.8
Neisseria gonorrhoeae	5					4.91		0.601											
Proteus spp.	2,230	160				910		326	723		2,180								
Pseudomonas aeruginosa	10,800	4,410	6,260		7,840	6,590	5,710	6,160											
Salmonella Paratyphi	0.317					0.317								0.00417					
Salmonella Typhi	77					67.4								11.6					
Non−typhoidal Salmonella	0.0286					0.0286													
Serratia spp.	144	25	49.9		72.8	31.3	30.9	50											
Shigella spp.	12.4					12.4													
Staphylococcus aureus	28,500					16,400			1,290	377		21,000			19,900				
Streptococcus pneumoniae	6,870			438	774	413		609	3,690			3,080	4,060						
All pathogens	115,000	21,300	12,300	30,300	25,100	71,000	11,100	36,300	31,100	4,820	28,200	25,400	4,080	11.6	19,900	129	0.556	129	32.8

Counts
- **100k − <500k**
- **<100k**
- **NA**

Hungary DALYs (count) associated with bacterial antimicrobial resistance by pathogen−drug combinations, 2019

	Resistance to 1+	AG	Anti−pseudomonal	BL−BLI	CP	FQ	4GC	3GC	TMP−SMX	Vanco	Aminopenicillin	Macrolide	PCN	MDR in S. Typhi and Paratyphi	Methicillin	Mono INH	Mono RIF	MDR excluding XDR in TB	XDR in TB
Acinetobacter baumannii	5,480	2,600	4,830	4,350	3,660	4,070	5,220	4,860											
Citrobacter spp.	542	37.2	273		85	107	70.8	465											
Enterobacter spp.	3,350	153	1,930		555	814	1,440		1,910										
Enterococcus faecalis	6,090					6,010				230									
Enterococcus faecium	13,500					13,500				6,140									
Other enterococci	1,720					1,710				102									
Escherichia coli	40,900	10,200		21,400	957	21,800		15,600	23,600		35,800								
Group A Streptococcus	301											301							
Group B Streptococcus	2,940					276						2,870	38.1						
Haemophilus influenzae	102							37.7			77								
Klebsiella pneumoniae	15,700	8,370		11,800	283	10,700		11,800	10,200										
Morganella spp.	48					33.3	3.4	28.2											
Mycobacterium tuberculosis	138															84.6	0.987	37	15.3
Neisseria gonorrhoeae	19.2					18.6		2.33											
Proteus spp.	3,020	111				1,350		825	1,380		2,820								
Pseudomonas aeruginosa	10,300	2,320	5,630		7,610	4,530	4,020	5,280											
Salmonella Paratyphi	0.4					0.398								0.0027					
Salmonella Typhi	194					155								45.3					
Non−typhoidal Salmonella	0.0331					0.0331													
Serratia spp.	495	154	119		199	163	157	206											
Shigella spp.	17.3					17.3													
Staphylococcus aureus	22,400					11,300			997	345		16,700			14,100				
Streptococcus pneumoniae	5,700			486	1,960	1,390		750	3,140			2,100	1,940						
All pathogens	133,000	23,900	12,800	38,000	15,300	77,900	10,900	39,800	41,300	6,810	38,700	21,900	1,970	45.3	14,100	84.6	0.987	37	15.3

Counts
- 100k – <500k
- <100k
- NA

Iceland DALYs (count) associated with bacterial antimicrobial resistance by pathogen−drug combinations, 2019

	Resistance to 1+	AG	Anti−pseudomonal	BL−BLI	CP	FQ	4GC	3GC	TMP−SMX	Vanco	Aminopenicillin	Macrolide	PCN	MDR in S. Typhi and Paratyphi	Methicillin	Mono INH	Mono RIF	MDR excluding XDR in TB	XDR in TB
Acinetobacter baumannii	82.3	10	49.8	72.3	14.3	11.9	35.7	71.4											
Citrobacter spp.	4.63	0.404	2.34		0.715	1.07	0.671	3.61											
Enterobacter spp.	38.6	2.53	29.8		6.62	3.55	12.4		13.8										
Enterococcus faecalis	24.5					22.6				2.92									
Enterococcus faecium	151					151				11.7									
Other enterococci	7.45					6.63				2.02									
Escherichia coli	455	38.8		313	18.9	174		110	253		400								
Group A Streptococcus	8.33											8.33							
Group B Streptococcus	51.6					23.8						39.2	0.903						
Haemophilus influenzae	6.33							0.681			6.04								
Klebsiella pneumoniae	170	37.4		153	4.57	40.6		55.7	97.1										
Morganella spp.	0.494					0.305	0.0447	0.314											
Mycobacterium tuberculosis	3.34															2.75	0.00424	0.467	0.114
Neisseria gonorrhoeae	0.125					0.124		0.000513											
Proteus spp.	41.2	3.86				7.05		7.83	20.1		40.6								
Pseudomonas aeruginosa	80.7	9.95	38.8		48.6	38.9	35.2	22.5											
Salmonella Paratyphi	0.0712					0.0713								0.00065					
Salmonella Typhi	0.992					0.884								0.125					
Non−typhoidal Salmonella	1.92					1.92													
Serratia spp.	3.94	0.608	1.03		1.15	0.784	1.51	2.77											
Shigella spp.	0.803					0.803													
Staphylococcus aureus	210					119			16.2	5.05		122			102				
Streptococcus pneumoniae	93			8.35	36.6	6.24		8.62	59			35.4	24.9						
All pathogens	1,440	104	122	546	131	612	85.5	284	460	21.7	447	205	25.8	0.125	102	2.75	0.00424	0.467	0.114

Counts
<100k
NA

Ireland DALYs (count) associated with bacterial antimicrobial resistance by pathogen−drug combinations, 2019

	Resistance to 1+	AG	Anti−pseudomonal	BL−BLI	CP	FQ	4GC	3GC	TMP−SMX	Vanco	Aminopenicillin	Macrolide	PCN	MDR in S. Typhi and Paratyphi	Methicillin	Mono INH	Mono RIF	MDR excluding XDR in TB	XDR in TB
Acinetobacter baumannii	1,330	46.4	817	1,240	87.4	75.4	828	1,020											
Citrobacter spp.	78.8	7.53	40.1		8.79	15.4	14.1	62.9											
Enterobacter spp.	973	37.6	625		63.9	246	658		422										
Enterococcus faecalis	789					761				56.7									
Enterococcus faecium	2,790					2,780				1,490									
Other enterococci	84.5					66.7				31.7									
Escherichia coli	9,380	1,510		7,060	194	3,940		2,280	4,660		9,090								
Group A Streptococcus	178											178							
Group B Streptococcus	589					101						544	12.6						
Haemophilus influenzae	102							15.8			95								
Klebsiella pneumoniae	3,140	577		2,090	78.2	1,280		1,150	2,690										
Morganella spp.	7.72					4.13	0.874	5.33											
Mycobacterium tuberculosis	46.5															24.9	0.355	17	4.23
Neisseria gonorrhoeae	2.36					2.35		0.0199											
Proteus spp.	702	55.2				52		38.3	212		695								
Pseudomonas aeruginosa	1,210	306	628		643	583	548	473											
Salmonella Paratyphi	0.0718					0.0719								0.000661					
Salmonella Typhi	16.7					14.5								2.58					
Non−typhoidal Salmonella	0.235					0.235													
Serratia spp.	51.2	6.75	15.8		9.95	8.78	6.8	42.1											
Shigella spp.	18.9					18.9													
Staphylococcus aureus	5,710					2,440			343	117		4,650			2,310				
Streptococcus pneumoniae	2,590			168	1,420	265		313	1,660			791	1,160						
All pathogens	29,800	2,540	2,130	10,600	2,500	12,700	2,060	5,410	10,000	1,690	9,880	6,170	1,170	2.58	2,310	24.9	0.355	17	4.23

Counts
- <100k
- NA

Israel DALYs (count) associated with bacterial antimicrobial resistance by pathogen−drug combinations, 2019

	Resistance to 1+	AG	Anti−pseudomonal	BL−BLI	CP	FQ	4GC	3GC	TMP−SMX	Vanco	Aminopenicillin	Macrolide	PCN	MDR in S. Typhi and Paratyphi	Methicillin	Mono INH	Mono RIF	MDR excluding XDR in TB	XDR in TB
Acinetobacter baumannii	2,680	1,700	2,520	2,180	1,870	2,300	2,200	2,430											
Citrobacter spp.	260	15.1	65.3		30	63.6	23.6	233											
Enterobacter spp.	1,110	67.8	614		450	243	420		442										
Enterococcus faecalis	2,110					2,050				156									
Enterococcus faecium	4,090					4,060				1,190									
Other enterococci	546					494				154									
Escherichia coli	19,700	1,150		14,300	742	9,960		7,590	14,100		17,400								
Group A Streptococcus	335											335							
Group B Streptococcus	1,100					259						967	18.1						
Haemophilus influenzae	196							25.2			188								
Klebsiella pneumoniae	6,650	1,650		5,420	497	3,120		5,110	4,050										
Morganella spp.	29.2					23	2.95	13											
Mycobacterium tuberculosis	120															43.7	1.42	60.4	14.7
Neisseria gonorrhoeae	4.37					4.35		0.0633											
Proteus spp.	1,690	48.8				706		441	931		1,650								
Pseudomonas aeruginosa	3,680	263	1,740		2,590	1,970	678	1,030											
Salmonella Paratyphi	0.25					0.25								0.00269					
Salmonella Typhi	59.1					48.6								12.1					
Non−typhoidal Salmonella	5.99					5.99													
Serratia spp.	115	14.4	20		20.2	20.3	13.2	98.9											
Shigella spp.	25.7					25.7													
Staphylococcus aureus	14,800					10,400			511	164		10,400			10,600				
Streptococcus pneumoniae	3,760			799	1,470	286		538	2,100			2,020	1,640						
All pathogens	63,000	4,910	4,960	22,700	7,660	36,000	3,330	17,500	22,100	1,660	19,300	13,700	1,660	12.1	10,600	43.7	1.42	60.4	14.7

Counts
- <100k
- NA

Italy DALYs (count) associated with bacterial antimicrobial resistance by pathogen−drug combinations, 2019

	Resistance to 1+	AG	Anti−pseudomonal	BL−BLI	CP	FQ	4GC	3GC	TMP−SMX	Vanco	Aminopenicillin	Macrolide	PCN	MDR in S. Typhi and Paratyphi	Methicillin	Mono INH	Mono RIF	MDR excluding XDR in TB	XDR in TB
Acinetobacter baumannii	22,200	17,900	20,600	20,500	19,600	18,800	20,500	20,800											
Citrobacter spp.	2,100	123	946		232	484	420	1,830											
Enterobacter spp.	10,800	1,060	7,030		3,270	2,680	3,380		4,590										
Enterococcus faecalis	22,800					22,400				1,490									
Enterococcus faecium	47,200					47,000				13,100									
Other enterococci	5,070					5,010				531									
Escherichia coli	173,000	45,400		105,000	2,950	110,000		78,600	92,000		154,000								
Group A Streptococcus	2,000											2,000							
Group B Streptococcus	11,800					1,210						11,400	179						
Haemophilus influenzae	1,020							138			975								
Klebsiella pneumoniae	65,300	36,300		55,600	28,300	50,000		51,800	41,300										
Morganella spp.	230					208	11.1	93.6											
Mycobacterium tuberculosis	1,010															481	43.9	392	98.2
Neisseria gonorrhoeae	84.8					83.7		7.53											
Proteus spp.	13,300	2,290				8,310		6,900	10,100		12,000								
Pseudomonas aeruginosa	37,300	8,710	19,200		24,200	21,600	15,300	17,400											
Salmonella Paratyphi	1.9					1.9								0.0137					
Salmonella Typhi	302					265								44.8					
Non−typhoidal Salmonella	1.67					1.67													
Serratia spp.	763	219	201		185	217	188	442											
Shigella spp.	130					130													
Staphylococcus aureus	124,000					83,000			3,810	1,240		85,900			78,800				
Streptococcus pneumoniae	19,400			1,540	6,680	2,670		2,300	4,540			13,800	6,880						
All pathogens	559,000	112,000	47,900	182,000	85,500	374,000	39,800	180,000	156,000	16,400	167,000	113,000	7,060	44.9	78,800	481	43.9	392	98.2

Counts
- 500k – <1M
- 100k – <500k
- <100k
- NA

Kazakhstan DALYs (count) associated with bacterial antimicrobial resistance by pathogen−drug combinations, 2019

	Resistance to 1+	AG	Anti−pseudomonal	BL−BLI	CP	FQ	4GC	3GC	TMP−SMX	Vanco	Aminopenicillin	Macrolide	PCN	MDR in S. Typhi and Paratyphi	Methicillin	Mono INH	Mono RIF	MDR excluding XDR in TB	XDR in TB
Acinetobacter baumannii	19,700	15,900	19,300	16,700	15,800	17,900	18,000	18,800											
Citrobacter spp.	1,420	152	320		429	425	316	1,090											
Enterobacter spp.	10,600	1,880	6,910		1,300	2,800	6,310		5,020										
Enterococcus faecalis	8,930					8,720				603									
Enterococcus faecium	18,400					18,300				3,790									
Other enterococci	5,410					5,400				716									
Escherichia coli	52,000	14,100		36,700	6,590	12,200		9,090	36,500		48,200								
Group A Streptococcus	948											948							
Group B Streptococcus	6,600					1,280						6,060	265						
Haemophilus influenzae	737							183			629								
Klebsiella pneumoniae	46,200	19,600		39,900	3,290	32,200		37,200	37,900										
Morganella spp.	123					86.5	20.6	71											
Mycobacterium tuberculosis	22,200															5,560	1,240	10,700	4,660
Neisseria gonorrhoeae	133					132		1.72											
Proteus spp.	4,930	1,200				1,820		1,320	2,810		4,690								
Pseudomonas aeruginosa	29,100	9,520	18,200		17,700	18,100	15,800	17,800											
Salmonella Paratyphi	34					33.9								0.303					
Salmonella Typhi	853					689								202					
Non−typhoidal Salmonella	30.7					30.7													
Serratia spp.	3,570	1,240	1,020		528	525	2,480	2,300											
Shigella spp.	216					216													
Staphylococcus aureus	37,300					21,100			2,320	714		22,400			24,700				
Streptococcus pneumoniae	30,700			2,900	10,200	1,270		4,440	27,400			6,200	10,200						
All pathogens	3e+05	63,600	45,800	96,200	55,700	143,000	42,900	92,400	112,000	5,820	53,500	35,600	10,500	202	24,700	5,560	1,240	10,700	4,660

Counts
100k – <500k
<100k
NA

Kyrgyzstan DALYs (count) associated with bacterial antimicrobial resistance by pathogen−drug combinations, 2019

	Resistance to 1+	AG	Anti−pseudomonal	BL−BLI	CP	FQ	4GC	3GC	TMP−SMX	Vanco	Aminopenicillin	Macrolide	PCN	MDR in S. Typhi and Paratyphi	Methicillin	Mono INH	Mono RIF	MDR excluding XDR in TB	XDR in TB
Acinetobacter baumannii	5,000	3,920	4,930	4,380	3,800	4,590	4,550	4,780											
Citrobacter spp.	499	95.7	87.3		139	255	159	334											
Enterobacter spp.	3,340	588	2,170		472	551	1,880		1,830										
Enterococcus faecalis	2,590					2,510				209									
Enterococcus faecium	4,670					4,650				854									
Other enterococci	1,550					1,550				73.3									
Escherichia coli	17,400	3,750		13,100	2,230	10,100		10,100	12,000		15,900								
Group A Streptococcus	233											233							
Group B Streptococcus	2,580					733						2,250	139						
Haemophilus influenzae	339							171			216								
Klebsiella pneumoniae	15,400	6,880		12,900	1,910	8,010		12,800	13,700										
Morganella spp.	44					33.2	11.9	20.3											
Mycobacterium tuberculosis	14,400															3,560	1,760	6,310	2,760
Neisseria gonorrhoeae	55.7					55		3.84											
Proteus spp.	1,500	355				442		525	924		1,420								
Pseudomonas aeruginosa	8,860	3,600	5,260		4,760	5,740	4,750	5,900											
Salmonella Paratyphi	0.733					0.731								0.00707					
Salmonella Typhi	424					378								56.5					
Non−typhoidal Salmonella	12.7					12.7													
Serratia spp.	1,010	309	353		207	188	645	674											
Shigella spp.	196					196													
Staphylococcus aureus	14,200					7,070			2,470	195		8,550			11,300				
Streptococcus pneumoniae	13,000			1,400	3,390	494		1,610	12,000			2,080	5,010						
All pathogens	107,000	19,500	12,800	31,700	16,900	47,500	12,000	37,000	42,900	1,330	17,500	13,100	5,150	56.5	11,300	3,560	1,760	6,310	2,760

Counts
- **100k − <500k**
- **<100k**
- **NA**

Latvia DALYs (count) associated with bacterial antimicrobial resistance by pathogen−drug combinations, 2019

	Resistance to 1+	AG	Anti−pseudomonal	BL−BLI	CP	FQ	4GC	3GC	TMP−SMX	Vanco	Aminopenicillin	Macrolide	PCN	MDR in S. Typhi and Paratyphi	Methicillin	Mono INH	Mono RIF	MDR excluding XDR in TB	XDR in TB
Acinetobacter baumannii	1,360	887	1,320	1,190	1,140	1,200	1,110	1,310											
Citrobacter spp.	103	6.34	51.6		36.1	22.9	15.8	71.9											
Enterobacter spp.	482	35.3	172		74.5	163	158		343										
Enterococcus faecalis	1,500					1,450				232									
Enterococcus faecium	2,130					2,120				930									
Other enterococci	360					357				27.9									
Escherichia coli	7,160	944		3,390	275	3,280		2,860	4,200		6,140								
Group A Streptococcus	118											118							
Group B Streptococcus	641					51.1						630	7.24						
Haemophilus influenzae	16.1							8.76			9.17								
Klebsiella pneumoniae	3,460	1,310		2,660	96.1	2,140		2,270	2,710										
Morganella spp.	13.8					3.19	1.36	13.1											
Mycobacterium tuberculosis	674															272	0.754	280	121
Neisseria gonorrhoeae	4.35					4.32		0.0709											
Proteus spp.	669	66.2				264		149	289		652								
Pseudomonas aeruginosa	2,230	771	1,220		1,110	1,420	1,290	1,400											
Salmonella Paratyphi	0.0913					0.0909								0.000658					
Salmonella Typhi	44.8					36.5								9.4					
Non−typhoidal Salmonella	7.98					7.98													
Serratia spp.	109	37	24		37.8	10.9	32	71.5											
Shigella spp.	14.4					14.4													
Staphylococcus aureus	2,400					446			215	76.8		2,050			776				
Streptococcus pneumoniae	1,500			157	448	203		105	1,220			324	386						
All pathogens	25,000	4,050	2,790	7,400	3,210	13,200	2,610	8,260	8,970	1,270	6,800	3,120	394	9.4	776	272	0.754	280	121

Counts
<100k
NA

Lithuania DALYs (count) associated with bacterial antimicrobial resistance by pathogen−drug combinations, 2019

	Resistance to 1+	AG	Anti−pseudomonal	BL−BLI	CP	FQ	4GC	3GC	TMP−SMX	Vanco	Aminopenicillin	Macrolide	PCN	MDR in S. Typhi and Paratyphi	Methicillin	Mono INH	Mono RIF	MDR excluding XDR in TB	XDR in TB
Acinetobacter baumannii	1,890	1,590	1,790	1,740	1,750	1,780	1,800	1,810											
Citrobacter spp.	192	10.4	81.1		20.5	46.8	14.7	172											
Enterobacter spp.	1,020	56.2	688		123	124	436		502										
Enterococcus faecalis	294					189				125									
Enterococcus faecium	3,040					2,990				1,500									
Other enterococci	496					493				41									
Escherichia coli	11,500	1,420		5,060	436	4,240		3,410	9,120		9,160								
Group A Streptococcus	403											403							
Group B Streptococcus	874					72.1						857	6.18						
Haemophilus influenzae	23.9							18.6			7.87								
Klebsiella pneumoniae	6,670	2,430		4,730	213	4,420		4,660	6,570										
Morganella spp.	20.2					1.46	1.86	20.1											
Mycobacterium tuberculosis	2,470															490	87.4	1,320	578
Neisseria gonorrhoeae	5.83					5.8		0.0474											
Proteus spp.	972	71.9				427		202	417		937								
Pseudomonas aeruginosa	2,760	599	1,350		1,460	1,320	2,100	1,190											
Salmonella Paratyphi	0.146					0.145								0.00119					
Salmonella Typhi	74.6					58.5								18.5					
Non−typhoidal Salmonella	0.00976					0.00976													
Serratia spp.	223	14.7	19.9		24.7	15.9	126	182											
Shigella spp.	10.8					10.8													
Staphylococcus aureus	3,840					458			311	98.6		3,320			1,350				
Streptococcus pneumoniae	2,670			306	607	245		343	2,000			1,170	1,010						
All pathogens	39,400	6,190	3,930	11,800	4,630	16,900	4,470	12,000	18,900	1,770	10,100	5,740	1,020	18.5	1,350	490	87.4	1,320	578

Counts
<100k
NA

Luxembourg DALYs (count) associated with bacterial antimicrobial resistance by pathogen−drug combinations, 2019

	Resistance to 1+	AG	Anti−pseudomonal	BL−BLI	CP	FQ	4GC	3GC	TMP−SMX	Vanco	Aminopenicillin	Macrolide	PCN	MDR in S. Typhi and Paratyphi	Methicillin	Mono INH	Mono RIF	MDR excluding XDR in TB	XDR in TB
Acinetobacter baumannii	172	5.38	122	151	5.58	22.5	110	152											
Citrobacter spp.	14.1	1.02	7.35		1.64	2.83	2.05	11.8											
Enterobacter spp.	77.9	4.53	60.9		15.8	15.7	25.5		21.7										
Enterococcus faecalis	87.7					79.8				12.7									
Enterococcus faecium	421					420				17.1									
Other enterococci	22.3					21.1				4.3									
Escherichia coli	1,210	154		765	32.1	543		311	623		1,090								
Group A Streptococcus	20.5											20.5							
Group B Streptococcus	90.9					32.2						74	1.89						
Haemophilus influenzae	11.3							1.68			10.7								
Klebsiella pneumoniae	365	131		304	13.4	224		261	121										
Morganella spp.	0.764					0.538	0.0733	0.407											
Mycobacterium tuberculosis	4.57															2.13	0.00458	1.97	0.463
Neisseria gonorrhoeae	0.513					0.507		0.0449											
Proteus spp.	97.8	9.87				26.8		17.4	40.2		96								
Pseudomonas aeruginosa	223	37.7	112		93.4	144	70.1	93.9											
Salmonella Paratyphi	0.148					0.148								0.00116					
Salmonella Typhi	4.65					4.29								0.532					
Non−typhoidal Salmonella	6.4					6.4													
Serratia spp.	10.1	1.13	2.2		2.29	1.94	1.85	8.37											
Shigella spp.	2.22					2.22													
Staphylococcus aureus	669					281			34.2	14.7		539			278				
Streptococcus pneumoniae	155			33.9	55.6	13.9		14.6	86.3			63	60.5						
All pathogens	3,660	345	305	1,250	220	1,840	209	872	926	48.8	1,200	697	62.4	0.533	278	2.13	0.00458	1.97	0.463

Counts
<100k
NA

Malta DALYs (count) associated with bacterial antimicrobial resistance by pathogen−drug combinations, 2019

	Resistance to 1+	AG	Anti−pseudomonal	BL−BLI	CP	FQ	4GC	3GC	TMP−SMX	Vanco	Aminopenicillin	Macrolide	PCN	MDR in S. Typhi and Paratyphi	Methicillin	Mono INH	Mono RIF	MDR excluding XDR in TB	XDR in TB
Acinetobacter baumannii	284	24.4	214	221	17.3	24.9	185	265											
Citrobacter spp.	20.8	1.38	8.59		1.02	5.6	3.15	17.9											
Enterobacter spp.	86.6	6.64	61.2		22.9	20.5	31.8		26.8										
Enterococcus faecalis	70.9					65.3				9.47									
Enterococcus faecium	260					260				14									
Other enterococci	34.9					30.5				11.4									
Escherichia coli	903	215		447	5.05	608		265	415		761								
Group A Streptococcus	37.6											37.6							
Group B Streptococcus	67.1					14						59.5	3.7						
Haemophilus influenzae	19.2							2.68			18.4								
Klebsiella pneumoniae	407	256		310	183	299		353	216										
Morganella spp.	1.31					0.959	0.215	0.633											
Mycobacterium tuberculosis	2.55															2	0.00128	0.445	0.102
Neisseria gonorrhoeae	0.25					0.25		0.000619											
Proteus spp.	70.8	7.15				22.9		22.5	36.8		64.4								
Pseudomonas aeruginosa	208	30.1	157		60.7	76.1	114	109											
Salmonella Paratyphi	0.0217					0.0217								0.00016					
Salmonella Typhi	2.18					1.75								0.52					
Non−typhoidal Salmonella	0.00089					0.00089													
Serratia spp.	7.6	1.3	2.06		1.85	2.36	0.981	5.05											
Shigella spp.	0.758					0.758													
Staphylococcus aureus	811					419			44.9	10.5		633			533				
Streptococcus pneumoniae	330			79.4	117	17.5		21.7	140			218	172						
All pathogens	3,630	542	443	1,060	409	1,870	336	1,060	880	45.3	844	947	176	0.52	533	2	0.00128	0.445	0.102

Counts: <100k; NA

Monaco DALYs (count) associated with bacterial antimicrobial resistance by pathogen−drug combinations, 2019

	Resistance to 1+	AG	Anti−pseudomonal	BL−BLI	CP	FQ	4GC	3GC	TMP−SMX	Vanco	Aminopenicillin	Macrolide	PCN	MDR in S. Typhi and Paratyphi	Methicillin	Mono INH	Mono RIF	MDR excluding XDR in TB	XDR in TB
Acinetobacter baumannii	20.8	13.9	18.1	17	11.6	9.28	16.7	18.8											
Citrobacter spp.	1.76	0.118	0.798		0.25	0.468	0.361	1.46											
Enterobacter spp.	8.14	0.577	5.9		2.24	1.3	2.71		2.54										
Enterococcus faecalis	7.71					7.2				0.856									
Enterococcus faecium	42.3					42.1				12.9									
Other enterococci	1.94					1.71				0.56									
Escherichia coli	141	17.2		101	1.97	71.8		49.7	79.8		125								
Group A Streptococcus	2.59											2.59							
Group B Streptococcus	13					9.01						7.45	0.337						
Haemophilus influenzae	1.55							0.224			1.47								
Klebsiella pneumoniae	40.6	13.4		35.4	1.28	12.5		10.5	23.5										
Morganella spp.	0.0788					0.0524	0.0107	0.0455											
Mycobacterium tuberculosis	1.28															0.776	0.00874	0.391	0.0987
Neisseria gonorrhoeae	0.0175					0.0174		0.000229											
Proteus spp.	9.18	0.789				2.24		2.46	6.38		8.14								
Pseudomonas aeruginosa	26.3	5.04	14		14.8	12.1	8.76	12.5											
Salmonella Paratyphi	0.000818					0.000818								5.47e−06					
Salmonella Typhi	0.445					0.381								0.0965					
Non−typhoidal Salmonella	0.164					0.164													
Serratia spp.	1.01	0.155	0.261		0.208	0.22	0.242	0.807											
Shigella spp.	0.0772					0.0772													
Staphylococcus aureus	100					58.2			3.71	1.39		76.3			36.1				
Streptococcus pneumoniae	31.7			4.08	8.13	4.99		2.23	15			21.1	6.39						
All pathogens	453	51.2	39	158	40.6	234	28.8	98.8	131	15.8	135	107	6.72	0.0965	36.1	0.776	0.00874	0.391	0.0987

Counts
<100k
NA

Montenegro DALYs (count) associated with bacterial antimicrobial resistance by pathogen−drug combinations, 2019

	Resistance to 1+	AG	Anti−pseudomonal	BL−BLI	CP	FQ	4GC	3GC	TMP−SMX	Vanco	Aminopenicillin	Macrolide	PCN	MDR in S. Typhi and Paratyphi	Methicillin	Mono INH	Mono RIF	MDR excluding XDR in TB	XDR in TB
Acinetobacter baumannii	788	557	774	668	617	705	756	749											
Citrobacter spp.	85.6	5.41	31.7		13.8	33.3	6.89	67.9											
Enterobacter spp.	321	40.2	177		53	103	168		195										
Enterococcus faecalis	230					226				12.3									
Enterococcus faecium	490					488				184									
Other enterococci	158					156				32.4									
Escherichia coli	1,510	362		910	119	711		1,080	967		1,280								
Group A Streptococcus	36.2											36.2							
Group B Streptococcus	120					61.1						83.5	1.95						
Haemophilus influenzae	9.38							2.27			8.13								
Klebsiella pneumoniae	894	382		779	89	616		883	630										
Morganella spp.	2.03					1.41	0.232	1.14											
Mycobacterium tuberculosis	4.53															0.817	0.0569	2.55	1.1
Neisseria gonorrhoeae	1.7					1.69		0.0312											
Proteus spp.	140	12.6				43		68	90		126								
Pseudomonas aeruginosa	514	159	267		355	340	259	237											
Salmonella Paratyphi	0.0204					0.0203								0.000169					
Salmonella Typhi	7.3					6.61								0.842					
Non−typhoidal Salmonella	1.1					1.1													
Serratia spp.	41.4	11.8	7.91		9.28	9.36	23.2	25.6											
Shigella spp.	0.412					0.412													
Staphylococcus aureus	830					417			40.8	11.7		505			649				
Streptococcus pneumoniae	453			71.9	176	24.8		65.3	255			249	218						
All pathogens	6,640	1,530	1,260	2,430	1,430	3,940	1,210	3,180	2,180	240	1,420	874	220	0.842	649	0.817	0.0569	2.55	1.1

Counts
- <100k
- NA

Netherlands DALYs (count) associated with bacterial antimicrobial resistance by pathogen−drug combinations, 2019

	Resistance to 1+	AG	Anti−pseudomonal	BL−BLI	CP	FQ	4GC	3GC	TMP−SMX	Vanco	Aminopenicillin	Macrolide	PCN	MDR in S. Typhi and Paratyphi	Methicillin	Mono INH	Mono RIF	MDR excluding XDR in TB	XDR in TB
Acinetobacter baumannii	6,140	360	3,980	4,730	353	556	3,320	5,150											
Citrobacter spp.	217	37.2	75		53.2	104	50.2	88.6											
Enterobacter spp.	2,760	242	1,750		820	235	1,260		905										
Enterococcus faecalis	2,820					2,720				169									
Enterococcus faecium	13,800					13,800				294									
Other enterococci	883					826				179									
Escherichia coli	41,200	4,510		26,100	662	13,900		6,960	25,800		34,700								
Group A Streptococcus	427											427							
Group B Streptococcus	3,330					389						3,180	106						
Haemophilus influenzae	130							79			66.6								
Klebsiella pneumoniae	9,390	1,800		2,550	127	4,460		4,150	8,410										
Morganella spp.	57.6					28.6	7.78	42.2											
Mycobacterium tuberculosis	207															123	1.61	66	16.3
Neisseria gonorrhoeae	5.45					5.41		0.0674											
Proteus spp.	3,330	124				105		230	2,110		3,050								
Pseudomonas aeruginosa	4,060	550	1,980		1,550	2,500	1,310	1,220											
Salmonella Paratyphi	0.831					0.831								0.00492					
Salmonella Typhi	70.2					59.1								13					
Non−typhoidal Salmonella	224					224													
Serratia spp.	285	81.1	42.6		82.2	64.1	26.9	182											
Shigella spp.	41.6					41.6													
Staphylococcus aureus	19,400					5,140			1,560	149		17,400			2,980				
Streptococcus pneumoniae	5,150			514	664	2,200		165	2,820			1,010	695						
All pathogens	114,000	7,700	7,820	33,900	4,310	47,300	5,970	18,300	41,600	792	37,800	22,000	801	13	2,980	123	1.61	66	16.3

Counts
- **100k – <500k**
- **<100k**
- **NA**

North Macedonia DALYs (count) associated with bacterial antimicrobial resistance by pathogen−drug combinations, 2019

	Resistance to 1+	AG	Anti−pseudomonal	BL−BLI	CP	FQ	4GC	3GC	TMP−SMX	Vanco	Aminopenicillin	Macrolide	PCN	MDR in S. Typhi and Paratyphi	Methicillin	Mono INH	Mono RIF	MDR excluding XDR in TB	XDR in TB
Acinetobacter baumannii	3,130	2,510	3,040	2,420	2,750	2,870	2,830	3,000											
Citrobacter spp.	335	19.1	140		67.6	107	51.1	270											
Enterobacter spp.	1,380	169	754		168	860	820		698										
Enterococcus faecalis	791					770				54.6									
Enterococcus faecium	1,820					1,820				570									
Other enterococci	597					589				119									
Escherichia coli	6,660	1,600		4,020	2,970	5,280		5,240	6,070		5,860								
Group A Streptococcus	138											138							
Group B Streptococcus	432					109						378	11.4						
Haemophilus influenzae	46.3							10.2			40.8								
Klebsiella pneumoniae	3,770	1,770		3,280	1,200	3,270		3,670	3,240										
Morganella spp.	11.5					6.74	2.3	7.67											
Mycobacterium tuberculosis	47.7															6.54	0.486	28.3	12.4
Neisseria gonorrhoeae	5.8					5.77		0.115											
Proteus spp.	546	50.1				250		193	321		486								
Pseudomonas aeruginosa	2,260	951	1,280		1,440	1,390	1,260	1,390											
Salmonella Paratyphi	0.0645					0.0643								0.000403					
Salmonella Typhi	38.9					35.2								4.44					
Non−typhoidal Salmonella	3.67					3.67													
Serratia spp.	284	68.6	56.6		39.3	37.3	246	152											
Shigella spp.	6.43					6.43													
Staphylococcus aureus	3,520					1,440			129	43.5		1,590			3,360				
Streptococcus pneumoniae	1,980			261	843	101		405	1,260			812	1,030						
All pathogens	27,800	7,140	5,260	9,980	9,490	18,900	5,220	14,300	11,700	787	6,390	2,910	1,050	4.44	3,360	6.54	0.486	28.3	12.4

Counts
- <100k
- NA

Norway DALYs (count) associated with bacterial antimicrobial resistance by pathogen−drug combinations, 2019

	Resistance to 1+	AG	Anti−pseudomonal	BL−BLI	CP	FQ	4GC	3GC	TMP−SMX	Vanco	Aminopenicillin	Macrolide	PCN	MDR in S. Typhi and Paratyphi	Methicillin	Mono INH	Mono RIF	MDR excluding XDR in TB	XDR in TB
Acinetobacter baumannii	1,470	388	784	1,190	496	86.9	785	1,220											
Citrobacter spp.	74.2	7.93	31.6		11.4	17.3	10.5	58.4											
Enterobacter spp.	653	56.8	463		124	58.7	221		256										
Enterococcus faecalis	641					608				58.3									
Enterococcus faecium	2,920					2,910				154									
Other enterococci	114					87.3				45.9									
Escherichia coli	9,840	1,390		6,010	4,150	2,930		1,460	4,340		7,210								
Group A Streptococcus	174											174							
Group B Streptococcus	851					98						818	31.9						
Haemophilus influenzae	129							11.3			125								
Klebsiella pneumoniae	2,820	590		2,190	39	1,830		685	1,610										
Morganella spp.	13.4					6.22	2.11	10.1											
Mycobacterium tuberculosis	83.5															44.8	0.114	30.7	7.77
Neisseria gonorrhoeae	7.65					7.6		0.195											
Proteus spp.	959	76.7				61.3		76.5	419		952								
Pseudomonas aeruginosa	1,150	197	529		701	476	536	381											
Salmonella Paratyphi	5.15					5.16								0.0369					
Salmonella Typhi	18.1					15.9								2.55					
Non−typhoidal Salmonella	12.3					12.3													
Serratia spp.	65.1	13	19.5		23.9	10.3	8.75	42.4											
Shigella spp.	25.5					25.5													
Staphylococcus aureus	3,420					1,110			339	123		1,270			2,840				
Streptococcus pneumoniae	1,080			117	300	135		124	692			320	343						
All pathogens	26,500	2,720	1,830	9,510	5,840	10,500	1,560	4,070	7,650	380	8,280	2,580	375	2.58	2,840	44.8	0.114	30.7	7.77

Counts
<100k
NA

Poland DALYs (count) associated with bacterial antimicrobial resistance by pathogen−drug combinations, 2019

	Resistance to 1+	AG	Anti−pseudomonal	BL−BLI	CP	FQ	4GC	3GC	TMP−SMX	Vanco	Aminopenicillin	Macrolide	PCN	MDR in S. Typhi and Paratyphi	Methicillin	Mono INH	Mono RIF	MDR excluding XDR in TB	XDR in TB
Acinetobacter baumannii	25,700	16,400	24,600	18,000	20,900	24,200	24,000	24,700											
Citrobacter spp.	2,200	129	1,450		346	962	527	1,010											
Enterobacter spp.	21,800	7,240	20,000		1,830	4,770	15,400		11,200										
Enterococcus faecalis	23,200					22,800				1,710									
Enterococcus faecium	38,800					38,800				15,800									
Other enterococci	6,000					5,940				738									
Escherichia coli	122,000	19,600		80,300	2,360	68,100		35,700	73,400		111,000								
Group A Streptococcus	778											778							
Group B Streptococcus	10,300					843						10,200	141						
Haemophilus influenzae	548							211			402								
Klebsiella pneumoniae	71,400	36,700		67,000	10,600	56,700		56,200	61,600										
Morganella spp.	148					81.4	15.2	105											
Mycobacterium tuberculosis	992															578	56.1	249	109
Neisseria gonorrhoeae	103					102		1.54											
Proteus spp.	11,500	457				3,340		2,110	4,740		11,400								
Pseudomonas aeruginosa	40,400	14,500	22,800		26,100	25,600	19,900	21,000											
Salmonella Paratyphi	1.37					1.37								0.0101					
Salmonella Typhi	644					528								138					
Non−typhoidal Salmonella	24					24													
Serratia spp.	607	213	184		174	172	144	215											
Shigella spp.	26.1					26.1													
Staphylococcus aureus	72,000					24,600			6,100	479		61,200			36,900				
Streptococcus pneumoniae	42,200			5,640	17,300	1,700		7,840	30,500			22,200	14,600						
All pathogens	491,000	95,300	69,100	171,000	79,500	279,000	60,000	149,000	188,000	18,800	122,000	94,300	14,800	138	36,900	578	56.1	249	109

Counts
- 100k – <500k
- <100k
- NA

Portugal DALYs (count) associated with bacterial antimicrobial resistance by pathogen−drug combinations, 2019

	Resistance to 1+	AG	Anti−pseudomonal	BL−BLI	CP	FQ	4GC	3GC	TMP−SMX	Vanco	Aminopenicillin	Macrolide	PCN	MDR in S. Typhi and Paratyphi	Methicillin	Mono INH	Mono RIF	MDR excluding XDR in TB	XDR in TB
Acinetobacter baumannii	5,100	1,550	4,530	4,640	2,700	2,490	4,280	4,740											
Citrobacter spp.	510	29.3	271		48.9	73.2	55.1	466											
Enterobacter spp.	2,970	150	2,350		1,020	401	1,040		690										
Enterococcus faecalis	4,520					4,420				281									
Enterococcus faecium	10,200					10,100				1,570									
Other enterococci	701					611				216									
Escherichia coli	37,600	7,950		20,600	896	17,300		11,100	24,200		32,400								
Group A Streptococcus	372											372							
Group B Streptococcus	3,190					250						3,130	38.5						
Haemophilus influenzae	401							69.7			369								
Klebsiella pneumoniae	17,100	8,480		14,100	4,440	12,300		13,600	11,300										
Morganella spp.	78.9					46.2	24.3	47.8											
Mycobacterium tuberculosis	495															311	6.52	142	36
Neisseria gonorrhoeae	3.23					3.21		0.0152											
Proteus spp.	2,490	154				244		594	1,690		2,070								
Pseudomonas aeruginosa	9,960	2,430	6,310		4,830	5,120	4,070	5,750											
Salmonella Paratyphi	1.49					1.49								0.0106					
Salmonella Typhi	79.7					67.2								14.2					
Non−typhoidal Salmonella	0.0254					0.0254													
Serratia spp.	221	28.5	60.7		40.8	59.2	40.5	172											
Shigella spp.	22.3					22.3													
Staphylococcus aureus	36,900					22,400			1,390	489		28,300			23,800				
Streptococcus pneumoniae	8,370			4,030	3,570	409		1,510	4,010			3,480	3,450						
All pathogens	141,000	20,800	13,500	43,400	17,500	76,400	9,510	38,100	43,300	2,550	34,800	35,300	3,490	14.2	23,800	311	6.52	142	36

Counts
- **100k – <500k**
- **<100k**
- **NA**

Republic of Moldova DALYs (count) associated with bacterial antimicrobial resistance by pathogen−drug combinations, 201

	Resistance to 1+	AG	Anti−pseudomonal	BL−BLI	CP	FQ	4GC	3GC	TMP−SMX	Vanco	Aminopenicillin	Macrolide	PCN	MDR in S. Typhi and Paratyphi	Methicillin	Mono INH	Mono RIF	MDR excluding XDR in TB	XDR in TB
Acinetobacter baumannii	2,520	2,100	2,480	2,170	2,250	2,430	2,450	2,470											
Citrobacter spp.	321	37.5	85.8		55.8	99.5	50.7	257											
Enterobacter spp.	2,100	279	1,190		316	550	1,360		970										
Enterococcus faecalis	3,430					3,360				253									
Enterococcus faecium	5,630					5,620				1,540									
Other enterococci	1,140					1,130				192									
Escherichia coli	18,900	4,040		12,800	1,640	10,600		11,100	13,300		16,500								
Group A Streptococcus	196											196							
Group B Streptococcus	977					119						927	51.3						
Haemophilus influenzae	114							34.4			92								
Klebsiella pneumoniae	11,000	5,990		9,650	724	7,280		9,390	8,850										
Morganella spp.	25.9					10.4	2.4	21.9											
Mycobacterium tuberculosis	5,740															573	172	3,490	1,500
Neisseria gonorrhoeae	10.5					10.5		0.0799											
Proteus spp.	1,410	440				620		270	689		1,340								
Pseudomonas aeruginosa	6,120	2,260	3,210		4,060	3,790	3,680	3,660											
Salmonella Paratyphi	0.52					0.519								0.00282					
Salmonella Typhi	237					190								58					
Non−typhoidal Salmonella	23.5					23.5													
Serratia spp.	590	218	177		86.4	54.9	404	393											
Shigella spp.	20					20													
Staphylococcus aureus	9,520					2,730			403	203		4,700			8,620				
Streptococcus pneumoniae	5,890			650	1,380	208		1,020	5,500			1,660	1,070						
All pathogens	76,000	15,400	7,150	25,300	10,500	38,800	7,940	28,600	29,800	2,190	18,000	7,490	1,120	58	8,620	573	172	3,490	1,500

Counts
- <100k
- NA

Romania DALYs (count) associated with bacterial antimicrobial resistance by pathogen−drug combinations, 2019

	Resistance to 1+	AG	Anti−pseudomonal	BL−BLI	CP	FQ	4GC	3GC	TMP−SMX	Vanco	Aminopenicillin	Macrolide	PCN	MDR in S. Typhi and Paratyphi	Methicillin	Mono INH	Mono RIF	MDR excluding XDR in TB	XDR in TB
Acinetobacter baumannii	13,200	9,970	12,600	8,220	12,300	12,200	12,900	11,700											
Citrobacter spp.	1,690	78.5	1,240		225	241	279	1,320											
Enterobacter spp.	6,190	664	954		1,330	3,690	1,250		4,010										
Enterococcus faecalis	15,900					15,700				1,070									
Enterococcus faecium	25,500					25,100				14,400									
Other enterococci	4,580					4,560				456									
Escherichia coli	83,400	17,600		49,600	1,570	39,500		31,800	30,100		78,700								
Group A Streptococcus	2,130											2,130							
Group B Streptococcus	5,990					594						5,810	36.9						
Haemophilus influenzae	1,330							1,030			524								
Klebsiella pneumoniae	50,300	39,300		47,200	29,100	37,500		40,300	34,000										
Morganella spp.	116					77.2	29	64.9											
Mycobacterium tuberculosis	4,750															1,310	469	2,060	911
Neisseria gonorrhoeae	45.4					44.6		3.25											
Proteus spp.	6,240	350				3,430		3,050	3,070		5,120								
Pseudomonas aeruginosa	37,900	22,900	26,900		28,500	25,500	21,800	22,000											
Salmonella Paratyphi	1.8					1.8								0.014					
Salmonella Typhi	588					478								136					
Non−typhoidal Salmonella	0.196					0.196													
Serratia spp.	3,820	742	693		519	173	3,780	2,180											
Shigella spp.	0.503					0.503													
Staphylococcus aureus	70,200					11,400			2,370	819		58,500			52,500				
Streptococcus pneumoniae	32,900			3,460	16,600	1,750		12,400	11,500			19,400	23,200						
All pathogens	367,000	91,500	42,400	108,000	90,200	182,000	40,000	126,000	85,000	16,700	84,300	85,800	23,300	136	52,500	1,310	469	2,060	911

Counts
- **100k – <500k**
- **<100k**
- **NA**

Russian Federation DALYs (count) associated with bacterial antimicrobial resistance by pathogen−drug combinations, 2019

	Resistance to 1+	AG	Anti−pseudomonal	BL−BLI	CP	FQ	4GC	3GC	TMP−SMX	Vanco	Aminopenicillin	Macrolide	PCN	MDR in S. Typhi and Paratyphi	Methicillin	Mono INH	Mono RIF	MDR excluding XDR in TB	XDR in TB
Acinetobacter baumannii	87,800	77,900	85,800	79,000	77,400	84,500	83,100	86,000											
Citrobacter spp.	7,050	689	4,250		757	2,050	967	4,900											
Enterobacter spp.	88,600	7,710	66,600		14,300	5,790	55,000		35,100										
Enterococcus faecalis	118,000					115,000				8,390									
Enterococcus faecium	194,000					194,000				18,800									
Other enterococci	38,700					38,400				7,070									
Escherichia coli	743,000	213,000		550,000	79,900	453,000		528,000	487,000		678,000								
Group A Streptococcus	6,470											6,470							
Group B Streptococcus	63,800					6,550						61,900	1,780						
Haemophilus influenzae	2,730							1,460			1,590								
Klebsiella pneumoniae	403,000	275,000		391,000	309,000	344,000		373,000	345,000										
Morganella spp.	1,110					356	98.7	996											
Mycobacterium tuberculosis	196,000															36,700	16,800	98,800	43,500
Neisseria gonorrhoeae	366					364		4.56											
Proteus spp.	53,000	29,700				12,000		2,240	24,800		49,000								
Pseudomonas aeruginosa	278,000	157,000	194,000		213,000	199,000	163,000	192,000											
Salmonella Paratyphi	27.4					27.3								0.183					
Salmonella Typhi	7,740					6,060								2,240					
Non−typhoidal Salmonella	721					721													
Serratia spp.	24,800	15,100	11,200		6,950	649	17,400	15,700											
Shigella spp.	1,240					1,240													
Staphylococcus aureus	290,000					194,000			15,100	5,730		127,000			222,000				
Streptococcus pneumoniae	266,000			20,100	28,800	11,100		66,400	247,000			31,700	76,800						
All pathogens	2,870,000	776,000	362,000	1,040,000	731,000	1,670,000	320,000	1,270,000	1,150,000	40,000	729,000	227,000	78,500	2,240	222,000	36,700	16,800	98,800	43,500

Counts
- >= 2M
- 1.5M− <2M
- 1M − <1.5M
- 500k − <1M
- 100k − <500k
- <100k
- NA

San Marino DALYs (count) associated with bacterial antimicrobial resistance by pathogen−drug combinations, 2019

	Resistance to 1+	AG	Anti−pseudomonal	BL−BLI	CP	FQ	4GC	3GC	TMP−SMX	Vanco	Aminopenicillin	Macrolide	PCN	MDR in S. Typhi and Paratyphi	Methicillin	Mono INH	Mono RIF	MDR excluding XDR in TB	XDR in TB
Acinetobacter baumannii	15.7	9.38	13.5	13	7.55	7.44	11.4	14.2											
Citrobacter spp.	1.48	0.0961	0.644		0.394	0.422	0.212	1.14											
Enterobacter spp.	5.48	0.375	3.94		1.45	1.08	1.94		1.71										
Enterococcus faecalis	4.41					4.07				0.538									
Enterococcus faecium	24.6					24.5				4.97									
Other enterococci	1.41					1.29				0.328									
Escherichia coli	77.3	9.3		55.1	1.13	40.5		28.4	44.2		66.6								
Group A Streptococcus	1.56											1.56							
Group B Streptococcus	6.26					3.23						4.35	0.192						
Haemophilus influenzae	0.805							0.167			0.737								
Klebsiella pneumoniae	24.7	6.94		21.9	0.88	5.31		7.44	14.6										
Morganella spp.	0.0663					0.0409	0.00856	0.0422											
Mycobacterium tuberculosis	0.31															0.203	0.0016	0.0847	0.021
Neisseria gonorrhoeae	0.0195					0.0194		0.000244											
Proteus spp.	5.84	0.488				1.35		1.46	3.74		5.37								
Pseudomonas aeruginosa	14	2.41	7.96		7.92	6.32	4.57	6.15											
Salmonella Paratyphi	0.000746					0.000746								5.16e−06					
Salmonella Typhi	0.21					0.18								0.04					
Non−typhoidal Salmonella	0.145					0.145													
Serratia spp.	0.674	0.0842	0.201		0.146	0.147	0.258	0.493											
Shigella spp.	0.0616					0.0616													
Staphylococcus aureus	48.6					28.7			1.86	0.671		29.8			32.5				
Streptococcus pneumoniae	16.7			2.42	4.16	2.9		1.19	7.07			11.6	2.77						
All pathogens	250	29.1	26.3	92.4	23.6	128	18.4	60.7	73.2	6.5	72.7	47.3	2.96	0.04	32.5	0.203	0.0016	0.0847	0.021

Counts
- **<100k**
- **NA**

Serbia DALYs (count) associated with bacterial antimicrobial resistance by pathogen−drug combinations, 2019

Pathogen	Resistance to 1+	AG	Anti−pseudomonal	BL−BLI	CP	FQ	4GC	3GC	TMP−SMX	Vanco	Aminopenicillin	Macrolide	PCN	MDR in S. Typhi and Paratyphi	Methicillin	Mono INH	Mono RIF	MDR excluding XDR in TB	XDR in TB
Acinetobacter baumannii	8,210	6,670	7,890	6,770	7,120	7,650	7,980	7,770											
Citrobacter spp.	1,090	387	259		127	382	258	861											
Enterobacter spp.	5,240	2,040	3,940		2,890	2,470	3,480		1,980										
Enterococcus faecalis	4,700					4,620				251									
Enterococcus faecium	9,710					9,680				2,640									
Other enterococci	2,240					2,220				201									
Escherichia coli	33,500	8,760		20,400	980	20,200		17,200	21,800		28,900								
Group A Streptococcus	1,420											1,420							
Group B Streptococcus	2,240					219						2,160	37.1						
Haemophilus influenzae	183							42.8			160								
Klebsiella pneumoniae	18,900	9,180		16,500	6,310	12,500		18,400	13,200										
Morganella spp.	125					105	11.1	99.5											
Mycobacterium tuberculosis	136															51.7	1.77	57.4	25
Neisseria gonorrhoeae	20.8					20.6		0.435											
Proteus spp.	2,910	1,310				1,610		1,400	1,190		2,510								
Pseudomonas aeruginosa	11,800	4,180	4,970		7,530	7,640	7,280	8,070											
Salmonella Paratyphi	0.224					0.223								0.00143					
Salmonella Typhi	188					162								31.8					
Non−typhoidal Salmonella	9.31					9.31													
Serratia spp.	1,470	450	64.9		74.3	199	1,250	1,160											
Shigella spp.	6.9					6.9													
Staphylococcus aureus	23,100					14,100			888	246		19,600			12,500				
Streptococcus pneumoniae	10,500			2,390	7,130	634		1,290	4,980			5,640	6,200						
All pathogens	138,000	33,000	17,100	46,000	32,200	84,500	20,300	56,300	44,000	3,330	31,600	28,800	6,230	31.8	12,500	51.7	1.77	57.4	25

Counts
100k – <500k
<100k
NA

Slovakia DALYs (count) associated with bacterial antimicrobial resistance by pathogen−drug combinations, 2019

	Resistance to 1+	AG	Anti−pseudomonal	BL−BLI	CP	FQ	4GC	3GC	TMP−SMX	Vanco	Aminopenicillin	Macrolide	PCN	MDR in S. Typhi and Paratyphi	Methicillin	Mono INH	Mono RIF	MDR excluding XDR in TB	XDR in TB
Acinetobacter baumannii	2,660	755	2,110	2,010	1,430	1,620	2,590	2,500											
Citrobacter spp.	287	17.9	109		74.4	63	32.5	231											
Enterobacter spp.	1,870	129	1,020		381	715	914		1,030										
Enterococcus faecalis	1,850					1,820				66.8									
Enterococcus faecium	6,040					6,010				2,500									
Other enterococci	750					733				160									
Escherichia coli	19,800	4,590		11,200	270	13,100		9,430	10,900		16,800								
Group A Streptococcus	494											494							
Group B Streptococcus	1,200					181						1,140	34.1						
Haemophilus influenzae	150							67.9			104								
Klebsiella pneumoniae	11,000	5,970		9,430	962	8,600		7,790	10,500										
Morganella spp.	20.1					13.8	1.93	11.3											
Mycobacterium tuberculosis	23.4															4.14	1.97	12.1	5.14
Neisseria gonorrhoeae	12					11.9		0.728											
Proteus spp.	1,490	70.1				698		453	645		1,420								
Pseudomonas aeruginosa	7,160	2,710	3,840		4,560	5,280	4,560	3,920											
Salmonella Paratyphi	0.111					0.11								0.000887					
Salmonella Typhi	93.6					80.9								15.4					
Non−typhoidal Salmonella	0.0169					0.0169													
Serratia spp.	314	51.4	45.1		64.7	48.7	236	176											
Shigella spp.	7.7					7.7													
Staphylococcus aureus	13,600					7,320			547	186		10,400			8,120				
Streptococcus pneumoniae	4,710			356	1,690	337		750	2,340			3,010	1,830						
All pathogens	73,500	14,300	7,130	23,000	9,440	46,600	8,340	25,300	26,000	2,910	18,300	15,000	1,860	15.4	8,120	4.14	1.97	12.1	5.14

Counts
<100k
NA

Slovenia DALYs (count) associated with bacterial antimicrobial resistance by pathogen−drug combinations, 2019

	Resistance to 1+	AG	Anti−pseudomonal	BL−BLI	CP	FQ	4GC	3GC	TMP−SMX	Vanco	Aminopenicillin	Macrolide	PCN	MDR in S. Typhi and Paratyphi	Methicillin	Mono INH	Mono RIF	MDR excluding XDR in TB	XDR in TB
Acinetobacter baumannii	989	319	692	801	427	522	428	934											
Citrobacter spp.	56.1	5.83	20.5		11	15.7	6.51	41.5											
Enterobacter spp.	538	25.7	296		65.7	83.3	268		291										
Enterococcus faecalis	969					953				54.3									
Enterococcus faecium	2,000					1,990				77.9									
Other enterococci	114					108				23.8									
Escherichia coli	4,960	672		3,110	218	2,380		1,160	2,040		4,480								
Group A Streptococcus	142											142							
Group B Streptococcus	255					58						224	6.52						
Haemophilus influenzae	30.5							5.17			27.9								
Klebsiella pneumoniae	1,900	342		1,110	18.8	1,300		1,210	1,340										
Morganella spp.	4.28					1.96	0.764	3.22											
Mycobacterium tuberculosis	2.13															1.23	0.0105	0.63	0.266
Neisseria gonorrhoeae	3.72					3.69		0.126											
Proteus spp.	449	14.1				179		45.7	199		440								
Pseudomonas aeruginosa	1,250	252	599		693	657	330	600											
Salmonella Paratyphi	0.159					0.158								0.00113					
Salmonella Typhi	11.7					10.3								1.7					
Non−typhoidal Salmonella	0.0141					0.0141													
Serratia spp.	49.7	6.21	7.04		17.7	9	4.07	36.3											
Shigella spp.	1.7					1.7													
Staphylococcus aureus	1,920					792			179	67.4		961			1,390				
Streptococcus pneumoniae	1,120			224	216	151		71.6	788			515	170						
All pathogens	16,800	1,640	1,610	5,240	1,670	9,220	1,040	4,100	4,830	223	4,950	1,840	176	1.71	1,390	1.23	0.0105	0.63	0.266

Counts
<100k
NA

Spain DALYs (count) associated with bacterial antimicrobial resistance by pathogen−drug combinations, 2019

	Resistance to 1+	AG	Anti−pseudomonal	BL−BLI	CP	FQ	4GC	3GC	TMP−SMX	Vanco	Aminopenicillin	Macrolide	PCN	MDR in S. Typhi and Paratyphi	Methicillin	Mono INH	Mono RIF	MDR excluding XDR in TB	XDR in TB
Acinetobacter baumannii	17,300	7,080	12,800	16,400	11,500	11,900	11,900	15,200											
Citrobacter spp.	1,470	111	717		231	308	179	1,230											
Enterobacter spp.	8,910	616	6,190		2,250	2,870	4,980		1,270										
Enterococcus faecalis	14,900					14,600				549									
Enterococcus faecium	37,200					37,100				1,650									
Other enterococci	3,270					3,190				392									
Escherichia coli	132,000	25,700		57,700	2,530	68,800		33,400	69,100		122,000								
Group A Streptococcus	4,540											4,540							
Group B Streptococcus	5,690					1,120						5,090	158						
Haemophilus influenzae	1,080							186			1,010								
Klebsiella pneumoniae	36,500	11,000		30,600	3,330	19,200		22,800	19,600										
Morganella spp.	237					170	23	137											
Mycobacterium tuberculosis	733															504	9.09	174	44.6
Neisseria gonorrhoeae	24.5					24.1		2.69											
Proteus spp.	10,300	338				1,480		838	7,070		9,470								
Pseudomonas aeruginosa	30,300	7,370	15,500		20,500	17,700	13,200	11,600											
Salmonella Paratyphi	2.29					2.3								0.0201					
Salmonella Typhi	226					196								35.9					
Non−typhoidal Salmonella	1,350					1,350													
Serratia spp.	733	89.6	182		146	185	131	576											
Shigella spp.	52.1					52.1													
Staphylococcus aureus	80,100					45,100			3,680	684		54,200			50,400				
Streptococcus pneumoniae	23,300			3,700	12,800	1,710		5,020	10,500			10,800	12,800						
All pathogens	410,000	52,300	35,400	108,000	53,300	227,000	30,500	90,900	111,000	3,280	132,000	74,600	13,000	35.9	50,400	504	9.09	174	44.6

Counts
100k – <500k
<100k
NA

Sweden DALYs (count) associated with bacterial antimicrobial resistance by pathogen−drug combinations, 2019

	Resistance to 1+	AG	Anti−pseudomonal	BL−BLI	CP	FQ	4GC	3GC	TMP−SMX	Vanco	Aminopenicillin	Macrolide	PCN	MDR in S. Typhi and Paratyphi	Methicillin	Mono INH	Mono RIF	MDR excluding XDR in TB	XDR in TB
Acinetobacter baumannii	2,870	167	1,690	2,400	161	237	1,510	2,090											
Citrobacter spp.	125	16.6	19.6		22.1	36.8	24.6	88											
Enterobacter spp.	998	88.4	805		354	93.1	212		92.1										
Enterococcus faecalis	914					838				117									
Enterococcus faecium	6,250					6,240				192									
Other enterococci	230					179				90.6									
Escherichia coli	15,700	2,450		10,500	245	7,320		3,640	7,910		11,400								
Group A Streptococcus	528											528							
Group B Streptococcus	1,660					235						1,580	34.3						
Haemophilus influenzae	95.8							27.5			78.3								
Klebsiella pneumoniae	3,590	576		3,290	76.4	1,750		1,200	1,000										
Morganella spp.	22.5					11.6	3.64	16											
Mycobacterium tuberculosis	194															108	1.29	68.1	16.8
Neisseria gonorrhoeae	7.15					7.09		0.333											
Proteus spp.	2,000	172				139		73.3	654		1,990								
Pseudomonas aeruginosa	2,180	162	817		1,400	967	517	796											
Salmonella Paratyphi	1.03					1.03								0.00731					
Salmonella Typhi	31.7					27.3								4.97					
Non−typhoidal Salmonella	31.6					31.6													
Serratia spp.	226	13.8	97.6		25.8	16.4	16.1	209											
Shigella spp.	110					110													
Staphylococcus aureus	3,190					1,110			721	285		1,660			1,390				
Streptococcus pneumoniae	1,810			245	285	138		128	1,230			518	676						
All pathogens	42,700	3,650	3,430	16,400	2,570	19,500	2,290	8,270	11,600	685	13,500	4,290	710	4.98	1,390	108	1.29	68.1	16.8

Counts
<100k
NA

Switzerland DALYs (count) associated with bacterial antimicrobial resistance by pathogen−drug combinations, 2019

	Resistance to 1+	AG	Anti−pseudomonal	BL−BLI	CP	FQ	4GC	3GC	TMP−SMX	Vanco	Aminopenicillin	Macrolide	PCN	MDR in S. Typhi and Paratyphi	Methicillin	Mono INH	Mono RIF	MDR excluding XDR in TB	XDR in TB
Acinetobacter baumannii	2,510	1,080	1,160	1,910	1,350	421	922	2,140											
Citrobacter spp.	188	14.7	24.6		19.9	26.3	18.8	173											
Enterobacter spp.	926	78.8	644		111	70.8	274		391										
Enterococcus faecalis	375					243				155									
Enterococcus faecium	5,310					5,300				252									
Other enterococci	187					149				69.4									
Escherichia coli	14,600	722		8,690	99.3	5,270		2,680	7,780		12,600								
Group A Streptococcus	286											286							
Group B Streptococcus	1,280					95.2						1,250	23.1						
Haemophilus influenzae	154							18.3			149								
Klebsiella pneumoniae	2,640	273		1,990	128	1,210		978	1,370										
Morganella spp.	17.3					9.39	2.21	12											
Mycobacterium tuberculosis	92.3															27.5	0.215	52	12.6
Neisseria gonorrhoeae	6.13					6.03		0.651											
Proteus spp.	1,520	110				256		125	663		1,500								
Pseudomonas aeruginosa	2,090	272	999		817	1,170	846	981											
Salmonella Paratyphi	0.212					0.212								0.00125					
Salmonella Typhi	56.4					49.1								8.61					
Non−typhoidal Salmonella	26.4					26.4													
Serratia spp.	87.3	13.7	30.3		19.9	14.6	14.8	65.7											
Shigella spp.	46.7					46.7													
Staphylococcus aureus	5,560					2,080			504	192		3,270			3,530				
Streptococcus pneumoniae	2,140			228	567	259		780	1,180			777	372						
All pathogens	40,100	2,560	2,850	12,800	3,110	16,700	2,080	7,950	11,900	668	14,200	5,570	395	8.61	3,530	27.5	0.215	52	12.6

Counts: <100k; NA

Tajikistan DALYs (count) associated with bacterial antimicrobial resistance by pathogen−drug combinations, 2019

	Resistance to 1+	AG	Anti−pseudomonal	BL−BLI	CP	FQ	4GC	3GC	TMP−SMX	Vanco	Aminopenicillin	Macrolide	PCN	MDR in S. Typhi and Paratyphi	Methicillin	Mono INH	Mono RIF	MDR excluding XDR in TB	XDR in TB
Acinetobacter baumannii	17,500	12,300	17,400	15,500	13,800	16,800	17,000	16,900											
Citrobacter spp.	2,210	509	542		853	899	612	1,680											
Enterobacter spp.	11,300	2,750	6,010		2,140	2,020	5,690		7,930										
Enterococcus faecalis	3,420					3,280				353									
Enterococcus faecium	5,330					5,310				1,150									
Other enterococci	3,690					3,690				205									
Escherichia coli	31,400	8,760		20,800	5,430	23,600		21,600	22,500		28,600								
Group A Streptococcus	327											327							
Group B Streptococcus	5,670					1,530						4,930	233						
Haemophilus influenzae	1,640							913			979								
Klebsiella pneumoniae	30,900	12,600		25,600	4,590	17,600		26,600	27,000										
Morganella spp.	107					89.6	19	45.5											
Mycobacterium tuberculosis	17,700															3,330	2,470	8,220	3,650
Neisseria gonorrhoeae	148					147		1.73											
Proteus spp.	2,510	910				1,070		1,060	1,510		2,410								
Pseudomonas aeruginosa	16,700	3,820	7,930		9,570	8,220	7,770	13,800											
Salmonella Paratyphi	5.95					5.92								0.18					
Salmonella Typhi	1,400					1,210								339					
Non−typhoidal Salmonella	100					100													
Serratia spp.	2,160	607	714		410	488	1,290	1,380											
Shigella spp.	1,730					1,730													
Staphylococcus aureus	27,100					11,500			9,540	300		19,800			20,300				
Streptococcus pneumoniae	67,500			14,500	34,600	2,440		8,650	56,600			27,000	32,900						
All pathogens	251,000	42,200	32,600	76,400	71,400	102,000	32,400	92,600	125,000	2,000	31,900	52,100	33,100	339	20,300	3,330	2,470	8,220	3,650

Counts
- 100k – <500k
- <100k
- NA

Turkey DALYs (count) associated with bacterial antimicrobial resistance by pathogen−drug combinations, 2019

	Resistance to 1+	AG	Anti−pseudomonal	BL−BLI	CP	FQ	4GC	3GC	TMP−SMX	Vanco	Aminopenicillin	Macrolide	PCN	MDR in S. Typhi and Paratyphi	Methicillin	Mono INH	Mono RIF	MDR excluding XDR in TB	XDR in TB
Acinetobacter baumannii	59,800	51,500	58,000	55,800	55,500	57,900	56,300	57,600											
Citrobacter spp.	2,490	153	1,010		785	352	168	2,010											
Enterobacter spp.	21,100	1,580	14,200		10,300	1,620	5,150		5,900										
Enterococcus faecalis	7,010					5,620				1,920									
Enterococcus faecium	46,500					46,400				12,700									
Other enterococci	14,600					14,100				8,310									
Escherichia coli	127,000	36,700		90,900	7,400	71,500		77,000	79,200		114,000								
Group A Streptococcus	2,030											2,030							
Group B Streptococcus	27,400					3,260						26,700	93.3						
Haemophilus influenzae	2,510							380			2,340								
Klebsiella pneumoniae	112,000	59,500		105,000	58,900	83,600		86,700	72,600										
Morganella spp.	328					291	79.7	114											
Mycobacterium tuberculosis	6,150															3,260	351	2,370	159
Neisseria gonorrhoeae	393					391		6.56											
Proteus spp.	11,900	435				2,080		2,720	7,850		11,000								
Pseudomonas aeruginosa	56,700	13,300	31,000		38,300	28,400	24,600	27,300											
Salmonella Paratyphi	142					142								1.28					
Salmonella Typhi	3,170					2,960								234					
Non−typhoidal Salmonella	502					502													
Serratia spp.	7,390	1,580	2,480		2,190	2,300	4,770	4,370											
Shigella spp.	419					419													
Staphylococcus aureus	162,000					40,700			4,960	2,250		62,100			159,000				
Streptococcus pneumoniae	99,600			16,100	60,800	3,780		15,300	69,400			50,300	83,200						
All pathogens	771,000	165,000	107,000	268,000	234,000	366,000	91,000	273,000	240,000	25,200	127,000	141,000	83,200	236	159,000	3,260	351	2,370	159

Counts
- 500k − <1M
- 100k − <500k
- <100k
- NA

Turkmenistan DALYs (count) associated with bacterial antimicrobial resistance by pathogen−drug combinations, 2019

	Resistance to 1+	AG	Anti−pseudomonal	BL−BLI	CP	FQ	4GC	3GC	TMP−SMX	Vanco	Aminopenicillin	Macrolide	PCN	MDR in S. Typhi and Paratyphi	Methicillin	Mono INH	Mono RIF	MDR excluding XDR in TB	XDR in TB
Acinetobacter baumannii	6,130	4,730	6,030	5,350	5,160	5,670	5,590	5,910											
Citrobacter spp.	633	109	133		244	333	90.8	404											
Enterobacter spp.	3,680	777	2,010		694	1,050	1,820		2,280										
Enterococcus faecalis	2,040					1,890				277									
Enterococcus faecium	5,270					5,220				2,240									
Other enterococci	1,810					1,810				107									
Escherichia coli	21,300	7,270		15,400	1,600	14,100		13,100	14,800		19,500								
Group A Streptococcus	240											240							
Group B Streptococcus	3,020					1,350						2,210	278						
Haemophilus influenzae	1,050							463			767								
Klebsiella pneumoniae	18,900	7,190		16,300	1,650	9,840		14,500	16,100										
Morganella spp.	51.3					42.1	10.9	19.8											
Mycobacterium tuberculosis	10,900															3,560	242	4,920	2,190
Neisseria gonorrhoeae	50					49.9		0.558											
Proteus spp.	1,660	330				657		652	1,030		1,520								
Pseudomonas aeruginosa	10,500	3,420	5,210		5,440	6,070	5,070	7,690											
Salmonella Paratyphi	0.11					0.11								0.000853					
Salmonella Typhi	553					423								167					
Non−typhoidal Salmonella	9.27					9.27													
Serratia spp.	993	337	310		186	205	596	598											
Shigella spp.	117					117													
Staphylococcus aureus	20,200					9,630			3,810	252		16,800			9,480				
Streptococcus pneumoniae	22,500			4,510	10,000	833		3,220	19,000			10,100	10,300						
All pathogens	131,000	24,200	13,700	41,600	25,000	59,300	13,200	46,500	57,000	2,870	21,800	29,400	10,600	167	9,480	3,560	242	4,920	2,190

Counts
- **100k – <500k**
- **<100k**
- **NA**

Ukraine DALYs (count) associated with bacterial antimicrobial resistance by pathogen−drug combinations, 2019

	Resistance to 1+	AG	Anti−pseudomonal	BL−BLI	CP	FQ	4GC	3GC	TMP−SMX	Vanco	Aminopenicillin	Macrolide	PCN	MDR in S. Typhi and Paratyphi	Methicillin	Mono INH	Mono RIF	MDR excluding XDR in TB	XDR in TB
Acinetobacter baumannii	66,800	56,700	65,500	58,500	58,900	63,100	65,400	65,300											
Citrobacter spp.	7,250	671	3,120		1,090	2,580	1,700	5,390											
Enterobacter spp.	33,800	3,600	20,100		4,420	8,420	21,300		15,300										
Enterococcus faecalis	40,200					39,400				2,920									
Enterococcus faecium	60,600					60,300				21,800									
Other enterococci	15,400					15,200				3,080									
Escherichia coli	180,000	36,900		119,000	12,700	96,400		96,700	123,000		155,000								
Group A Streptococcus	3,030											3,030							
Group B Streptococcus	15,000					1,720						14,500	599						
Haemophilus influenzae	1,270							301			1,090								
Klebsiella pneumoniae	108,000	67,000		98,800	12,800	79,900		83,700	88,100										
Morganella spp.	314					106	27.8	279											
Mycobacterium tuberculosis	113,000															16,100	12,600	58,300	25,800
Neisseria gonorrhoeae	124					123		0.165											
Proteus spp.	16,300	4,300				5,080		2,270	7,650		15,900								
Pseudomonas aeruginosa	71,800	26,700	38,000		50,500	41,900	41,500	46,500											
Salmonella Paratyphi	0.302					0.301								0.00153					
Salmonella Typhi	2,070					1,340								1,010					
Non−typhoidal Salmonella	557					557													
Serratia spp.	8,310	3,170	2,350		1,310	660	5,400	5,680											
Shigella spp.	482					482													
Staphylococcus aureus	43,000					28,300			4,090	2,570		19,900			18,900				
Streptococcus pneumoniae	85,100			7,030	18,000	3,390		16,900	71,300			28,700	34,500						
All pathogens	873,000	199,000	129,000	283,000	160,000	449,000	135,000	323,000	309,000	30,300	172,000	66,200	35,100	1,010	18,900	16,100	12,600	58,300	25,800

Counts
- 500k − <1M
- 100k − <500k
- <100k
- NA

United Kingdom DALYs (count) associated with bacterial antimicrobial resistance by pathogen−drug combinations, 2019

	Resistance to 1+	AG	Anti−pseudomonal	BL−BLI	CP	FQ	4GC	3GC	TMP−SMX	Vanco	Aminopenicillin	Macrolide	PCN	MDR in S. Typhi and Paratyphi	Methicillin	Mono INH	Mono RIF	MDR excluding XDR in TB	XDR in TB
Acinetobacter baumannii	23,400	1,420	765	15,100	1,170	1,770	991	20,800											
Citrobacter spp.	2,760	169	1,760		223	295	223	2,390											
Enterobacter spp.	12,300	908	8,040		3,580	879	6,620		2,560										
Enterococcus faecalis	17,000					15,900				2,040									
Enterococcus faecium	58,900					58,700				20,100									
Other enterococci	3,200					2,680				1,110									
Escherichia coli	195,000	34,500		139,000	2,560	68,400		41,400	116,000		178,000								
Group A Streptococcus	2,650											2,650							
Group B Streptococcus	4,760					1,740						3,400	296						
Haemophilus influenzae	3,380							347			3,290								
Klebsiella pneumoniae	57,600	9,260		41,500	1,930	20,100		22,100	42,100										
Morganella spp.	253					126	42.7	183											
Mycobacterium tuberculosis	937															636	32.8	215	53.6
Neisseria gonorrhoeae	74.6					73.7		2.75											
Proteus spp.	14,600	1,070				1,090		978	10,300		12,900								
Pseudomonas aeruginosa	28,600	4,210	15,600		14,600	14,300	9,150	10,100											
Salmonella Paratyphi	4.81					4.81								0.0363					
Salmonella Typhi	353					302								57.6					
Non−typhoidal Salmonella	3,390					3,390													
Serratia spp.	1,530	157	348		218	315	182	1,340											
Shigella spp.	268					268													
Staphylococcus aureus	95,200					41,300			6,860	2,260		65,300			52,500				
Streptococcus pneumoniae	35,400			4,650	3,740	10,800		2,520	24,200			6,300	8,010						
All pathogens	561,000	51,800	26,500	2e+05	28,000	243,000	17,200	102,000	202,000	25,600	195,000	77,600	8,310	57.6	52,500	636	32.8	215	53.6

Counts
- **500k − <1M**
- **100k − <500k**
- **<100k**
- **NA**

Uzbekistan DALYs (count) associated with bacterial antimicrobial resistance by pathogen−drug combinations, 2019

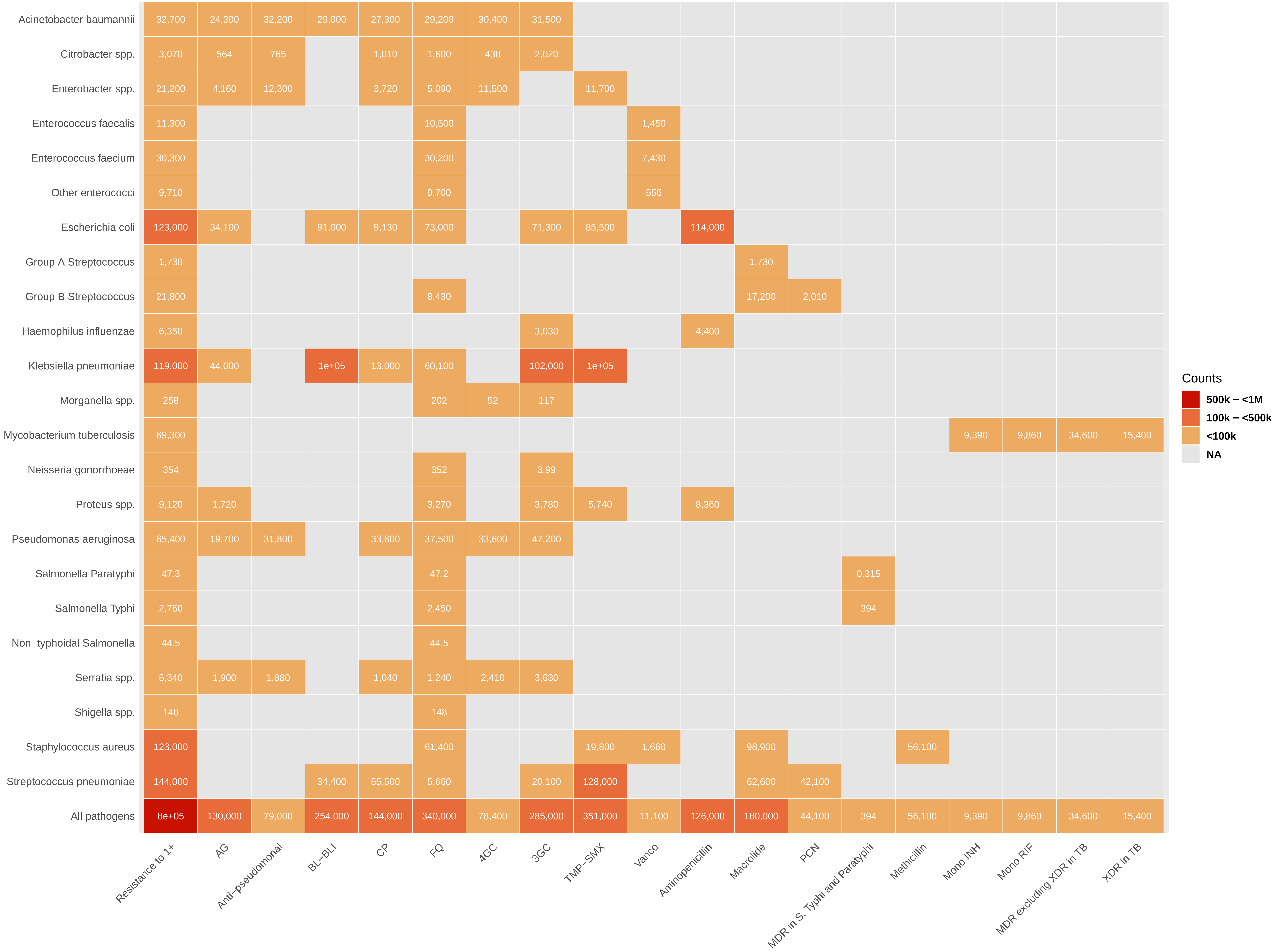

Supplement: Supplementary appendix 3 [file mmc3.pdf]
